# Diastereoselective Radical 1,4-Ester Migration: Radical Cyclizations of Acyclic Esters with SmI_2_

**DOI:** 10.1021/jacs.2c05972

**Published:** 2022-07-20

**Authors:** Charlotte Morrill, Áron Péter, Ilma Amalina, Emma Pye, Giacomo E. M. Crisenza, Nikolas Kaltsoyannis, David J. Procter

**Affiliations:** Department of Chemistry, The University of Manchester, Oxford Road, Manchester, M13 9PL, U.K.

## Abstract

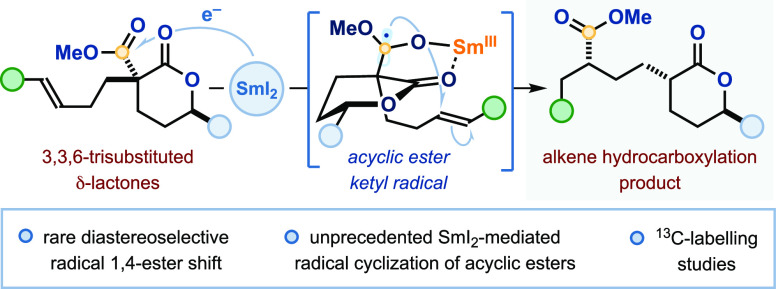

Reductive cyclizations of carbonyl compounds, mediated by samarium(II) diiodide (SmI_2_, Kagan’s reagent), represent an invaluable platform to generate molecular complexity in a stereocontrolled manner. In addition to classical ketone and aldehyde substrates, recent advances in radical chemistry allow the cyclization of lactone and lactam-type substrates using SmI_2_. In contrast, acyclic esters are considered to be unreactive to SmI_2_ and their participation in reductive cyclizations is unprecedented. Here, we report a diastereoselective radical 1,4-ester migration process, mediated by SmI_2_, that delivers stereodefined alkene hydrocarboxylation products via radical cyclization of acyclic ester groups in α-carbomethoxy δ-lactones. Isotopic labeling experiments and computational studies have been used to probe the mechanism of the migration. We propose that a switch in conformation redirects single electron transfer from SmI_2_ to the acyclic ester group, rather than the “more reactive” lactone carbonyl. Our study paves the way for the use of elusive ketyl radicals, derived from acyclic esters, in SmI_2_-mediated reductive cyclizations.

## Introduction

**Scheme 1:**
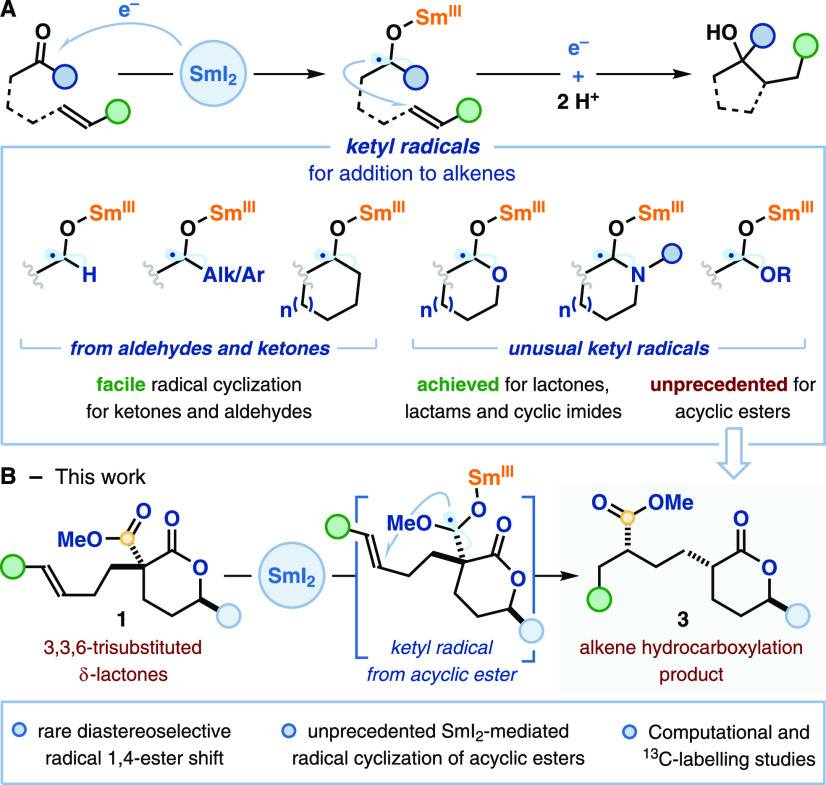
(A) SmI_2_-Mediated Radical Cyclization of Carbonyl Compounds; (B) Radical Cyclization of Acyclic Esters with SmI_2_ Underpins an Unusual Radical 1,4-Ester Shift (This Work)

Radical cyclizations are privileged processes for the regio- and stereocontrolled construction of molecular complexity.^1^ In particular, cyclizations triggered by single electron transfer (SET)^2^ reduction of carbonyl compounds, using the archetypal SET reducing agent samarium(II) diiodide (SmI_2_, Kagan’s reagent),^3^ offer a radical umpolung strategy that couples carbonyl moieties with unsaturated functionalities and delivers decorated cyclic structures (Scheme 1A).^4^ For example, the facile intramolecular addition of ketyl radicals,^5^ generated upon treatment of ketones and aldehydes with SmI_2_,^6^ to alkenes continues to provide effective solutions for the synthesis of high-profile natural products and bioactive molecules.^7^ Recently, our group^8^ and others^9^ have exploited the use of coordinating additives (e.g., H_2_O, phosphoramides, ureas, amines, etc.) to modulate the reactivity of SmI_2_,^10^ and to achieve the SET reduction of more recalcitrant lactam, cyclic imide, and lactone derivatives, thus expanding the scope of SmI_2_-mediated reductive cyclizations to more “unusual” ketyl radicals. Acyclic esters, however, have long been considered to be unreactive to SmI_2_, and as such, they are often used as innocent chelating groups to direct SmI_2_ reactions.^11^ In an attempt to fill this synthetic gap, our group reported the use of SmI_2_·H_2_O·Et_3_N to reduce acyclic esters to the corresponding ketyl radical equivalents.^12^ However, in this system, the enhanced reducing power of SmI_2_ led to over-reduction of the ketyl radical intermediate thus precluding exploitation in radical cyclizations. To date, the reductive cyclization of acyclic esters with SmI_2_ remains unprecedented.^13^

Herein, we present the first SmI_2_-mediated radical cyclizations of acyclic esters: methyl esters embedded within substituted-δ-lactones **1** undergo SET reduction by SmI_2_, to form elusive acyclic ester ketyl radicals (Scheme 1B). These undergo efficient intramolecular addition to a pendant alkene, outcompeting both the unproductive back electron transfer (BET) to the metal center and overreduction of the ketyl radical by SmI_2_. Fragmentation of the resultant spirocyclic tetrahedral intermediate (*vide infra*) provides stereocontrolled access to alkene hydrocarboxylation products **3**. Isotopic labeling studies and DFT calculations have been used to explore the unique diastereoselective radical 1,4-ester migration process.^14^

## Design of Reactivity

**Scheme 2:**
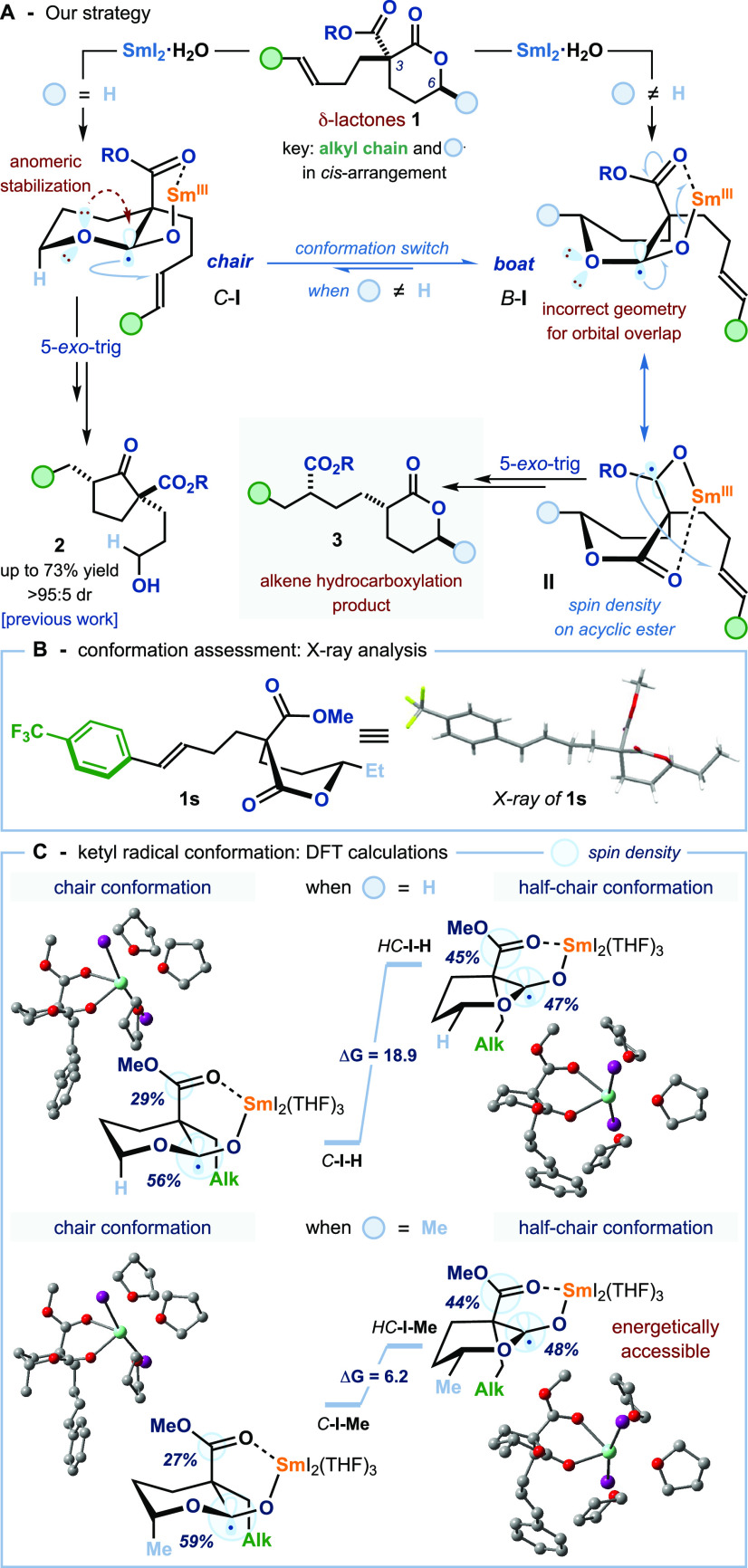
Radical Cyclization of Lactones Enabled by SmI_2_·H_2_O: (A) Our Strategy: Conformational Distortion of Sm(III)-Ketyl Radical Intermediates Drives the Cyclization of Acyclic Esters; (B) Conformation Assessment of δ-Lactones **1** by X-ray Analysis; (C) Computational Studies on Ketyl Radical Anions I (Δ*G* Values Reported in kJ/mol) Percentages refer to distribution of spin density.

We have previously highlighted the important role of conformation in the stabilization of the ketyl radical intermediates in the SmI_2_-mediated reductive cyclizations of lactones.^8a^ We proposed that SET from SmI_2_·H_2_O to 3-carboxyl-3-alkyl-disubstituted δ-lactones, generating Sm(III)-radical intermediate **I**, is facilitated by the ability of the lactone and its ketyl radical anion to access a chair conformation *C*-**I** (Scheme 2A, left-hand side). This conformation grants the correct orbital orientation to permit the anomeric stabilization^15^ of the pseudoaxial radical in **I**, and fosters a diastereoselective 5-*exo*-trig cyclization pathway that delivers pentanone products **2**, after collapse of the resultant tetrahedral intermediate.

In our quest for the first SmI_2_-mediated radical cyclizations of acyclic esters, we envisaged that a remote substituent on the lactone ring in **1** could be used to direct reductive ketyl-radical generation to the acyclic ester group. Specifically, we postulated that, by forcing lactones **1**, or the ketyl radicals formed upon their reduction, into a boat conformation **(***B-***I)**, the correct geometry for orbital overlap between the carbon-centered radical and the lone pair on the adjacent oxygen atom would be disrupted. This would destabilize ketyl radical **I** and drive radical relocation to the acyclic ester moiety, generating Sm(III)-ketyl radical intermediate **II** (Scheme 2A, right-hand side). This switch would convert the former ancillary coordinating group into a noninnocent reactive functionality. We envisaged that the key conformational switch could be achieved by introducing an alkyl group at the C6 position of the lactone ring, *anti* to the ester group. This substituent should favor a boat conformation for the lactone^16^ and the ketyl radical (*B-***I**), by destabilizing chair ketyl radical *C*-**I**. Thus, desired ketyl radical **II** would result from relocation of the spin density and undergo 5-*exo*-trig cyclization, providing hydrocarboxylation products **3** after an unusual radical 1,4-ester migration.

Computational and structural studies on the parent lactones support our proposal that the introduction of an alkyl group at the C6 position of the lactone ring, *anti* to the ester group, can lead to a conformational switch: our DFT calculations indicate that lactone **1-H** preferentially adopts a half-chair conformation while trisubstituted lactone **1a** bearing a C6-Me substituent prefers a boat conformation^18^—the latter being consistent with the X-ray crystal structure obtained for derivative **1s** (Scheme 2B). Our findings are consonant with literature crystallographic data for related lactone structures.^17,18^

Furthermore, computational studies on the ketyl radical intermediates derived from lactones **1-H** and **1a** support our proposal, although the conformational switch is more subtle, involving a switch from a chair to half-chair conformation rather than a switch from chair to boat conformation. Calculations suggest that, while both ketyl radicals favor chair conformations (*C*-**I-H** and *C*-**I-Me**), the presence of the axial C6-Me substituent in *C*-**I-Me** brings it closer in energy to a half-chair conformation *HC*-**I-Me** (Scheme 2C). Crucially, in the energetically accessible half chair conformation *HC*-**I-Me**, anomeric stabilization of the ketyl radical derived from the lactone carbonyl is suboptimal and increased spin density resides at the acyclic ester group; this is consistent with formation of the desired ketyl radical **II** (*cf.*Scheme 2A). Interestingly, the axial C6-Me in *C*-**I-Me** is also likely to block the approach of the alkene moiety to the radical thus disfavoring cyclization.

## Results and Discussion

The feasibility of our design plan was assessed using 6-methyl-substituted δ-lactone **1a** (Table 1). First, **1a** was exposed to SmI_2_·H_2_O, the reagent system previously used for the reductive radical cyclization of lactones and the formation of cyclopentanones **2** (entry 1). Under these conditions, the reaction delivered a complex mixture that contained a 1:1.6 ratio of cyclopentanone **2a** and the desired radical 1,4-ester migration product **3a**, resulting from radical cyclization of the acyclic ester group. The use of HMPA as an additive^10^ (entry 2) led to a cleaner reaction and increased the preference for product **3a**, while increasing the equivalents of SmI_2_ resulted in the complete consumption of starting material **1a** (entry 3). By reducing the amount of both H_2_O and HMPA (entries 4–6), increased chemoselectivity was observed and the amount of **3a** increased. Optimal conditions for the radical cyclization of the acyclic ester group were obtained when the protocol with SmI_2_·H_2_O·HMPA was performed at −78 °C:under these conditions, radical 1,4-ester migration product **3a** was isolated in 76% yield as a 3:1 mixture of diastereoisomers (entry 7). Crucially, the use of tripyrrolidinophosphoric acid triamide (TPPA) as a nontoxic alternative to HMPA, under the conditions reported in entry 7, provided migration product **3a** with similar levels of efficiency and selectivity (66% yield, 4:1 dr).^18^ The diastereomeric mixture obtained for **3a** arises from a protonation event at the C3-carbon of the lactone ring (*vide infra*), whereas the radical 1,4-ester migration, and construction of the new stereocenter, takes place with complete diastereocontrol.

**Table 1:**
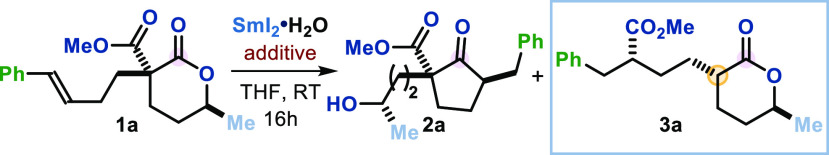

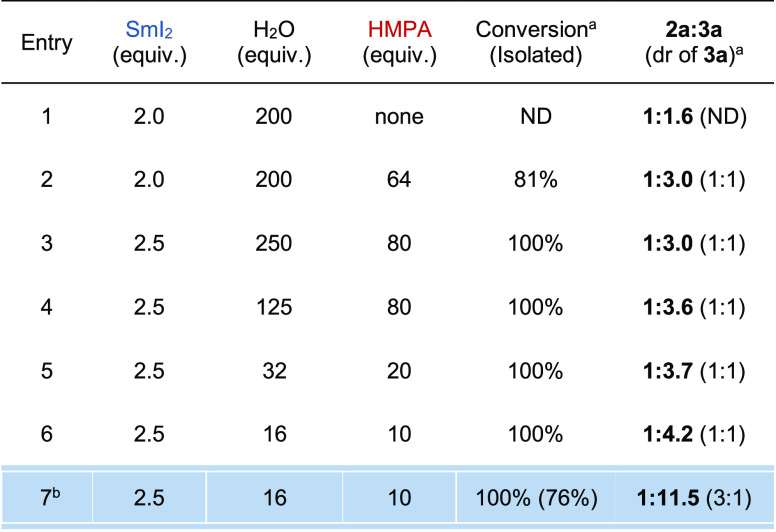
Optimization of the Radical 1,4-Ester Migration

a The conversion of starting material, the products ratio and the diastereoisomeric ratio of **3a** were determined by ^1^H NMR analysis of the crude reaction mixture.

b Reaction performed at −78 °C. The yellow circle within compound **3a** denotes the stereocenter that gives rise to the diastereoisomeric mixture. HMPA: hexamethylphosphoramide. THF: tetrahydrofuran. ND: not determined, due to the complexity of the reaction mixture.

**Figure 1:**
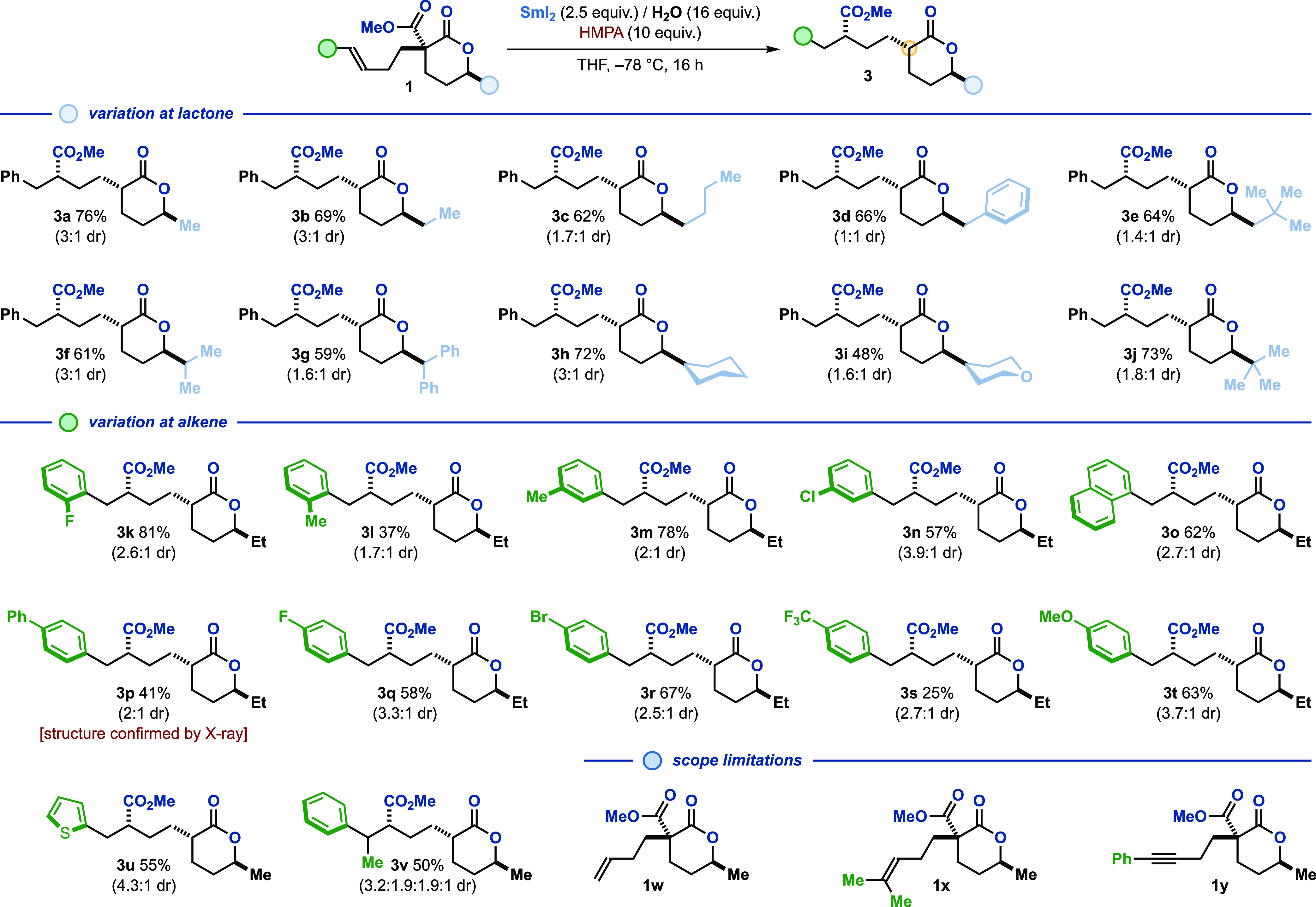
Scope of the method. Reaction conditions: **1** (1 equiv.), SmI_2_ (0.1 M in THF, 2.5 equiv.), H_2_O (16 equiv.), HMPA (10 equiv.), in THF (0.5 mL/0.1 mmol of substrate), at −78 °C under a nitrogen atmosphere. Isolated yields. The diastereoisomeric ratio was determined by ^1^H NMR analysis of the crude reaction mixture. The yellow circle within general product structure **3** denotes the stereocenter at which there is a diastereoisomeric mixture.

To evaluate the scope of the radical 1,4-ester migration, an array of δ-lactones **1**, adorned with different alkyl substituents at the C6-position, was submitted to the optimized Sm(II)-conditions (Figure 1). Primary alkyl substituents (methyl-, ethyl-, *n*-butyl-, benzyl-, and neopentyl-) were well tolerated, and ester migration products **3a**–**e** were obtained in good yield. Likewise, lactones **1f**–**j**, bearing bulkier secondary and tertiary alkyl substituents (including cyclohexyl- and tetrahydropyranyl-groups), efficiently underwent the ester radical cyclization process to deliver products **3f**–**j**. When evaluating the effects on reactivity brought about by substitution on the arene moiety of **1**, we found that both electron-donating (methyl-, naphthyl-, phenyl-, and methoxy-) and electron-withdrawing (fluoro-, chloro-, bromo-, and trifluoromethyl-) functionalities, at all positions of the aromatic ring, were compatible with our SmI_2_-conditions (products **3k**–**t**). The relative stereochemistry of the major and minor diastereoisomers of the product of 1,4-ester migration **3p** was confirmed by X-ray crystallographic analysis.^18^ Furthermore, a heteroaryl substituted olefin and a trisubstituted styrene derivative successfully participated in the 1,4-ester migration protocol to give thiophene derivative **3u** and product **3v**, respectively. C6-Substituted δ-lactones tailored with terminal (**1w**) or α,α-dialkyl-substituted (**1x**) pendant alkenes proved unsuitable for the SmI_2_-mediated 1,4-ester migration reaction. Furthermore, phenylalkynyl-derivative **1y** also failed to deliver the corresponding 1,4-ester migration product. These observations suggest that the generation of a stabilized benzylic radical intermediate—upon ketyl radical cyclization—facilitates the transformation.

**Scheme 3:**
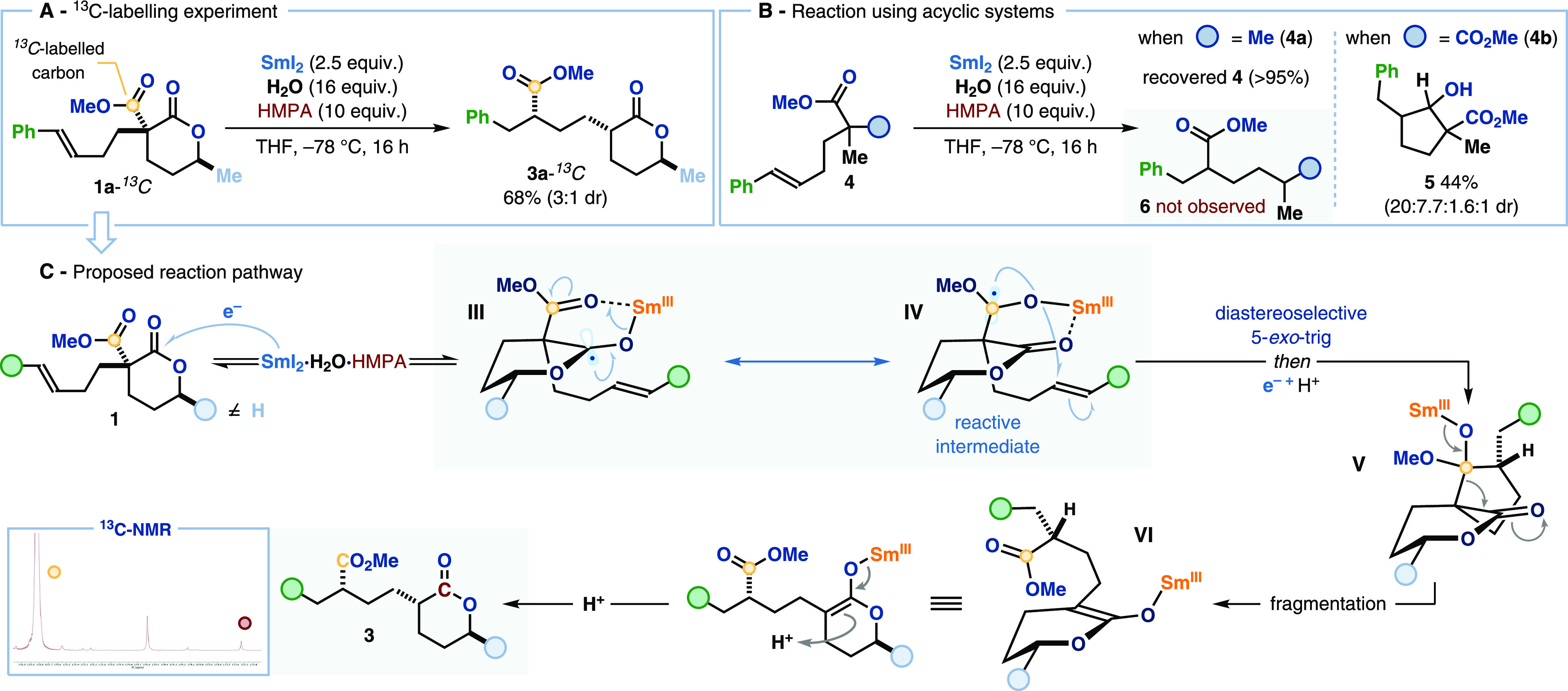
Mechanistic Studies and Proposed Reaction Pathway The diastereoisomeric ratios were determined by ^1^H NMR analysis of the crude reaction mixture.

A ^13^C-isotope labeling experiment was used to track the migration event and shed light on the mechanism of the SmI_2_-mediated radical 1,4-ester shift. Analogue **1a-**^13^*C*, bearing a ^13^C-labeled acyclic ester group, was submitted to the optimized SmI_2_-mediated conditions. Labeled product **3a-**^13^*C* was isolated in 68% yield (Scheme 3A) with ^13^C-enrichment solely at the carboxylic carbon of the migrated ester unit (*cf.*Scheme 3C, bottom left). This confirms that a Sm(III)-ketyl radical, formed from the acyclic ester, engages the alkene moiety in a radical cyclization event.

We next explored the behavior of related acetate and malonate derivatives **4**—in which there are no chemoselectivity challenges—with the aim of further probing the role of the lactone ring in the reactivity of **1**. Exposure of methyl hexenoate derivative **4a** to the standard SmI_2_-conditions gave no reaction (Scheme 3B). Whereas, treatment of malonate **4b** with SmI_2_·H_2_O·HMPA, under standard reaction conditions, gave cyclopentanol derivative **5** in low yield as a mixture of four diastereoisomers. Cyclopentanol **5** is formed by ester radical cyclization, collapse of a tetrahedral intermediate, and further reduction of the cyclopentanone intermediate. In both cases, no 1,4-ester migration product **6** was observed. The latter experiment confirms that the SET reduction of acyclic esters is not limited to lactone-containing substrates; however, the lactone moiety is key to drive productive fragmentation of the spirocyclic intermediate—generated after the radical cyclization event—to give products **3**; 1,4-ester migration in lactones **1** crucially generates a more stable cyclic Sm(III)-enolate species (p*K*_a_ values for δ-valerolactone = 25.2 and ethyl acetate = 27.5, in DMSO).^19^

A plausible mechanistic pathway for the SmI_2_-mediated radical 1,4-ester migration is outlined in Scheme 3C. SET from SmI_2_·H_2_O·HMPA to lactones **1** forms ketyl radicals **III**. The substituent at C6 on the lactone ring disfavors the radical adopting the chair conformation necessary for anomeric stabilization and relocation of the spin density to the acyclic ester gives ketyl radicals **IV**. Facile, diastereoselective 5-*exo*-trig radical cyclization, SET reduction, and protonation of the ensuing radical species generate spirocyclic intermediates **V** that collapse to form cyclic Sm(III)-enolates **VI**. Protonation of **VI** delivers 1,4-ester migration products **3**.

## Conclusion

SmI_2_, in the presence of H_2_O and HMPA, mediates the unprecedented reductive radical cyclization of acyclic esters. Varying the substitution on the lactone ring in α-carbomethoxy δ-lactones allows a switch in conformation that redirects SET from SmI_2_ to the acyclic ester group, rather than to the “more reactive” lactone carbonyl. 5-*Exo*-trig cyclization of the ketyl radical derived from the ester group results in a diastereoselective radical 1,4-ester migration, and the formation of stereodefined alkene hydrocarboxylation products. The protocol tolerates a range of functional groups, and the migration event proceeds with complete diastereocontrol. In addition to describing the first SmI_2_-mediated ketyl-olefin couplings of acyclic esters, and an unusual radical 1,4 ester migration, our studies showcase how control of conformation can be used to alter the chemoselectivity of radical processes.

## Supporting Information Available

Additional experiments, experimental procedures, characterization data, CCDC numbers for X-ray crystal structures, and spectra for all new compounds. (PDF)
