# Supplementary material for: Diastereoselective Radical 1,4-Ester Migration: Radical Cyclizations of Acyclic Esters with SmI2
Source: J Am Chem Soc. 2022 Jul 20;144(30):13946–52. doi: 10.1021/jacs.2c05972 (PMC9377304; doi:10.1021/jacs.2c05972)

Diastereoselective radical 1,4-ester migration: radical cyclizations of acyclic esters with SmI_2

Charlotte Morrill[1], Áron Péter[1], Ilma Amalina[1†], Emma Pye[1†], Giacomo E. M. Crisenza[1], Nikolas Kaltsoyannis[1], and David J. Procter[1]*

1 – Department of Chemistry, The University of Manchester, Oxford Road, Manchester, M13 9PL (UK).
E-mail: david.j.procter@manchester.ac.uk

Supporting information

Table of Contents

1. General Information 3
2. General Procedures 4
   2.1 General Procedure A: Grubbs I Cross Metathesis 4
   2.2 General Procedure B: Michael Addition 4
   2.3 General Procedure C: Grignard addition followed by DMP oxidation 4
   2.4 General Procedure D: Ketone reduction followed by lactonisation 4
   2.5 General Procedure E: SmI_2–H_2O–HMPA–mediated ester migration 4
   2.6 General Procedure F: $NaB(OMe)_4$-mediated Michael addition 5
3. Synthesis and Characterization of Starting Materials 6
4. Synthesis and Characterization of 1,4–Ester Migration Products 36
5. Unsuccessful substrates 50
6. Mechanistic Investigations 51
   5.1 Origin of the Diastereoselectivity 51
   5.2 Experimental Data for Scheme 3A: ^{13}C–labelling experiment 52
      5.2.1 Synthesis of a ^{13}C–labelled substrate 52
      5.2.2 Discussion 54
   5.3 Experimental Data for Scheme 3B: Reaction using a Malonate Derivative 57
7. DFT Studies 59
   6.1 Lowest energy conformations of the lactone substrates 59
      6.1.1 Background 59
      6.1.2 Results 59
      6.1.3 Computational Details 61
      6.1.4 Breakdown of Energy Contributions 61
      6.1.5 Cartesian Coordinates (Å) 62
   6.2 Lowest energy conformations of the lactone-derived ketyl radicals 69
      6.2.1 Computational Details 69
      6.2.2 Breakdown of Energy Contributions 69
      6.2.3 Cartesian Coordinates (Å) 70
8. X–Ray Crystallography Data 77
9. References 79
10. NMR Spectra 81

1. General Information

Experiments were performed under an atmosphere of nitrogen using anhydrous solvents, unless otherwise stated. THF was freshly distilled before use over sodium wire and benzophenone. All other solvents and reagents used were purchased from commercial suppliers and used according to relevant guidelines.

^{1}H NMR spectra were obtained at room temperature on a Bruker 400 or 500 MHz spectrometer. ^{13}C NMR were obtained at 101 or 126 MHz respectively. ^{19}F NMR were obtained at 376 MHz. All NMR spectra were processed using *Mestrenova©* NMR software. Chemical shifts are reported in parts per million (ppm), relative to residual chloroform (δH = 7.27 ppm and δC = 77.0 ppm) as internal standards, and coupling constants (*J*) are reported in Hz. Splitting patterns are reported as follows: singlet (s), doublet (d), triplet (t), quartet (q), heptet (hept), broad singlet (bs), double of doublets (dd), doublet of triplets (dt), doublet of quartets (dq), triplet of triplets (tt), quartet of doublets (qd), doublet of doublets of doublets (ddd), doublet of doublets of triplets (ddt), doublet of triplets of doublets (dtd), doublet of quartets of doublets (dqd), triplet of doublets of doublets (tdd), doublet of doublets of doublets of doublets (dddd) and multiplet (m).

Column chromatography was carried out using 35–70 m, 60 Å silica gel. TLC analysis was carried out on aluminium sheets coated with silica gel 60 F254, 0.2 mm thickness and visualised using potassium permanganate solution, cerium ammonium molybdate, anisaldehyde and/or UV light at 254 nm.

Mass spectra were obtained using positive and negative electrospray ($ES^{\pm}$) or atmospheric pressure chemical ionisation (APCI) methodology.

Infra-red spectra were recorded as evaporated films or neat using a FT/IR spectrometer and values are reported in cm^{-1}.

Melting points were measured on a Stuart Scientific capillary melting point apparatus.

2. General Procedures

2.1 General Procedure A: Grubbs I Cross Metathesis

To a solution of malonate derivative (1.0 eq.) and (substituted) styrene (3.0 eq.) in degassed CH_2Cl_2 (0.25 M) was added Grubbs I catalyst (0.36 g, 0.44 mmol, 5 mol%). The reaction was heated to 35 °C for 18 h, then concentrated. Purification by silica gel column chromatography followed by stirring overnight with decolourising charcoal yielded the desired product.

2.2 General Procedure B: Michael Addition

To a suspension of sodium methoxide (1.2 eq.) in MeOH was added malonate derivative (1.0 eq.) in MeOH. The reaction was stirred for 10 minutes and then α,β–unsaturated ketone (1.2 eq.) was added. The reaction was stirred at room temperature for 16 h, then was quenched with H_2O and the organic solvent was removed under vacuum. The aqueous layer was extracted with EtOAc, the organics were washed with brine, dried ($MgSO_4$) and concentrated. Purification by silica gel column chromatography yielded the desired product.

2.3 General Procedure C: Grignard addition followed by DMP oxidation

To a solution of aldehyde (1.0 eq.) in THF (0.4 M) was added dropwise vinyl magnesium bromide (1.0 M in THF, 1.1 eq.) at –78 °C. After the addition was complete, the reaction was allowed to warm to room temperature and was stirred for 3 h. The reaction was quenched by the addition of NH_4Cl saturated aqueous solution, the aqueous layer was extracted with Et_2O, the combined organics were washed with brine, dried ($MgSO_4$) and concentrated. The crude reaction mixture was dissolved in CH_2Cl_2 (0.25 M), cooled to 0 °C, and Dess–Martin Periodinane (1.2 eq.) was added. The reaction was warmed to room temperature and stirred for 3 h. A 1:1 mixture of saturated aqueous $Na_2S_2O_3$ and $NaHCO_3$ was added, and the mixture was allowed to stir overnight. The aqueous layer was extracted with CH_2Cl_2, the combined organics were dried ($MgSO_4$) and carefully concentrated to avoid loss of the volatile product. The crude product was used in the subsequent step without further purification.

2.4 General Procedure D: Ketone reduction followed by lactonisation

To a solution of samarium diiodide (0.1 M in THF, 2.5 eq.) and deionised water (250 eq.) in THF was added a solution of malonate derivative (1.0 eq.) in THF. The reaction was stirred at room temperature until decolourisation occurred, and then a saturated aqueous solution of Rochelle's salt was added. The aqueous layer was extracted with Et_2O, the combined organics were washed with brine, dried ($MgSO_4$) and concentrated. The crude reaction mixture was dissolved in CH_2Cl_2 and *para*–toluenesulfonic acid (10 mol%) was added. The reaction was stirred at room temperature for 16 h, then $NaHCO_3$ was added. The aqueous layer was extracted with CH_2Cl_2, the combined organics were dried ($MgSO_4$) and concentrated. Purification by silica gel column chromatography yielded the desired product.

2.5 General Procedure E: SmI_2–H_2O–HMPA–mediated ester migration

To a solution of SmI_2 (0.1 M in THF, 2.5 eq.), HMPA (10 eq.) and H_2O (16 eq.) at –78 °C was added a solution of lactone **1** (1.0 eq.) in THF (0.2 M). The reaction was stirred at this temperature until decolourisation occurred (1-16 h, typically 16 h), then saturated aqueous Rochelle's salt solution was added. The aqueous layer was extracted with Et_2O, the combined organics were washed with 1M HCl, $NaHCO_3$, brine, dried ($MgSO_4$) and concentrated. Purification by silica gel column chromatography yielded the desired product.

2.6 General Procedure F: $NaB(OMe)_4$-mediated Michael addition

According to a modified literature procedure[1]: the alkylated dimethyl malonate derivative was dissolved in dry MeCN (0.1 M) under N_2 at r.t. $NaB(OMe)_4$ (0.2 mmol) and methyl vinyl ketone (2.0 eq.) were added sequentially then the mixture was stirred overnight at r.t. Celite was added and the volatiles were evaporated to adsorb the crude product. Purification by silica gel column chromatography yielded the desired product.

3. Synthesis and Characterization of Starting Materials

Dimethyl 2–(but–3–en–1–yl)malonate (S1)[2]

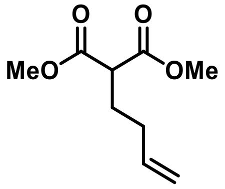

To a suspension of sodium hydride (1.1 g, 26 mmol, 1.2 eq.) in THF (90 mL) at 0 °C was added dropwise dimethyl malonate (3.0 mL, 26 mmol, 1.2 eq.). After stirring at this temperature for 30 minutes, 4–bromo–1–butene (2.2 mL, 22 mmol, 1.0 eq.) was added dropwise. The reaction was then heated to 67 °C for 19 h, and was then quenched by the addition of H_2O (20 mL). The mixture was diluted with Et_2O (200 mL) and the organics were washed with H_2O (50 mL) and brine (50 mL), dried ($MgSO_4$) and concentrated. Purification by silica gel column chromatography (hexane/EtOAc 95:5) yielded the title product as a yellow oil (2.6 g, 14 mmol, 53%). ^{1}H NMR (500 MHz, $CDCl_3$) δ 1.98–2.06 (m, 2H, CHCH_2CH$_2$), 2.10 (t, *J* = 6.9 Hz, 2H, CHCH$_2$CH_2), 3.41 (t, *J* = 7.3 Hz, 1H, C*H*CO$_2$Me), 3.75 (s, 6H, CO$_2$CH_3), 4.98–5.09 (m, 2H, CH=CH_2), 5.77 (ddt, *J* = 16.9, 10.2, 6.6 Hz, 1H, C*H*=CH$_2$) ppm; ^{13}C NMR (101 MHz, $CDCl_3$) δ 27.9 (CH*C*H$_2$CH$_2$), 31.2 (CHCH$_2$*C*H$_2$), 50.9 (*C*HCH$_2$CH$_2$), 52.5 (CO$_2$*C*H$_3$), 116.0 (CH$_2$CH=*C*H$_2$), 136.7 (CH$_2$*C*H=CH$_2$), 169.8 (*C*O$_2$CH$_3$) ppm. Data is consistent with literature.

Dimethyl (*E*)–2–(4–phenylbut–3–en–1–yl)malonate (S2)

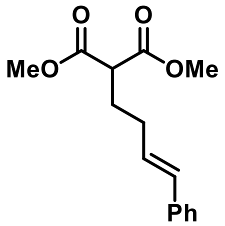

Prepared according to General Procedure A using dimethyl 2–(but–3–en–1–yl)malonate **S1** (3.3 g, 18 mmol, 1.0 eq.), styrene (6.1 mL, 53 mmol, 3.0 eq.) and Grubbs I catalyst (0.36 g, 0.44 mmol, 5 mol%) in CH_2Cl_2 (68 mL). Purification by silica gel column chromatography (hexane/EtOAc 100:0 to 8:2), followed by stirring overnight with decolourising charcoal, yielded the title product as a brown oil (2.2 g, 8.3 mmol, 43%). ^{1}H NMR (400 MHz, $CDCl_3$) δ 2.10 (dt, *J* = 13.2, 6.9 Hz, 2H, CHCH$_2$CH_2), 2.20–2.36 (m, 2H, CHC-H_2CH$_2$)), 3.45 (t, *J* = 7.4 Hz, 1H, C*H*CH$_2$), 3.74 (s, 6H, CO$_2$CH_3), 6.16 (dt, *J* = 15.7, 6.8 Hz, 1H, C*H*=CHAr), 6.41 (d, *J* = 15.9 Hz, 1H, CH=C*H*Ar), 7.22 (ddd, *J* = 9.2, 5.0, 2.8 Hz, 1H, ArC*H*), 7.28–7.38 (m, 4H, ArC*H*) ppm; ^{13}C NMR (101 MHz, $CDCl_3$) δ 28.4 (CH*C*H$_2$CH$_2$), 30.6 (CHCH$_2$*C*H$_2$), 50.9 (*C*HCH$_2$CH$_2$), 53.4 (CO$_2$*C*H$_3$), 126.0 (Ar*C*H), 127.1 (Ar*C*H), 128.5 (*C*H=CHAr), 128.5 (Ar*C*H), 131.4 (CH=*C*HAr), 137.1 (Ar*C*), 169.8 (*C*O$_2$CH$_3$) ppm; IR v_{max} (thin film, cm^{-1}) = 2952, 1731 (C=O), 1434, 1252, 1196, 1152; HRMS calcd. for $C_{15}H_{18}O_4Na$ $[M+Na]^+$ 285.1097, found, 285.1090.

Dimethyl (*E*)–2–(3–oxobutyl)–2–(4–phenylbut–3–en–1–yl)malonate (S3)

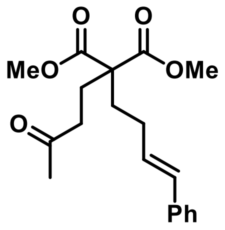

Prepared according to General Procedure B using dimethyl (*E*)–2–(4–phenylbut–3–en–1–yl)malonate **S2** (1.6 g, 6.1 mmol, 1.0 eq.), methyl vinyl ketone (0.61 mL, 7.3 mmol, 1.2 eq.) and sodium methoxide (0.40 g, 7.3 mmol, 1.2 eq.) in MeOH (12 mL). Purification by silica gel column chromatography (hexane/EtOAc 8:2) yielded the title product as a yellow oil (1.2 g, 3.6 mmol, 60%). ^{1}H NMR (400 MHz, $CDCl_3$) δ 1.96–2.05 (m, 4H, $C\mathit{H}_2CH_2C(O)CH_3$ + $CH_2C\mathit{H}_2C(O)CH_3$), 2.11 (s, 3H, $CH_2CH_2C(O)C\mathit{H}_3$), 2.12–2.21 (m, 2H, $CH_2C\mathit{H}_2CH{=}CHAr$), 2.45–2.39 (m, 2H, $C\mathit{H}_2CH_2CH{=}CHAr$), 3.68 (s, 6H, $CO_2C\mathit{H}_3$), 6.10 (dt, *J* = 15.8, 6.5 Hz, 1H, C*H*=CHAr), 6.35 (d, J = 15.9 Hz, 1H, CH=C*H*Ar), 7.12–7.18 (m, 1H, ArC*H*), 7.20–7.25 (m, 2H, ArC*H*), 7.25–7.29 (m, 2H, ArC*H*) ppm; ^{13}C NMR (101 MHz, $CDCl_3$) δ 26.7 ($CH_2\mathit{C}H_2CH{=}CHAr$), 27.7 ($CH_2\mathit{C}H_2C(O)CH_3$), 30.0 ($CH_2CH_2C(O)\mathit{C}H_3$), 33.2 ($\mathit{C}H_2CH_2CH{=}CH$), 38.7 ($\mathit{C}H_2CH_2C(O)CH_3$), 52.5 ($CO_2\mathit{C}H_3$), 56.6 ($\mathit{C}_q$), 125.9 (Ar*C*H), 127.1 (Ar*C*H), 128.5 (Ar*C*H), 128.9 (*C*H=CHAr), 130.6 (CH=*C*HAr), 137.4 (Ar*C*), 171.7 ($\mathit{C}O_2CH_3$), 207.1 ($CH_2CH_2\mathit{C}(O)CH_3$) ppm; IR ν_{max} (thin film, cm^{-1}) = 2929, 2853, 1729 (C=O), 1447, 1221, 965; HRMS calcd. for $C_{19}H_{24}O_5Na$ $[M+Na]^+$ 355.1516, found 355.1509.

Dimethyl (*E*)–2–(3–oxopentyl)–2–(4–phenylbut–3–en–1–yl)malonate (S4)

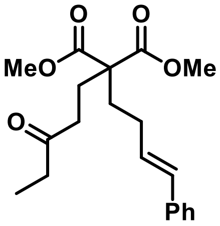

Prepared according to General Procedure B using dimethyl (*E*)–2–(4–phenylbut–3–en–1–yl)malonate **S2** (300 mg, 1.1 mmol, 1.0 eq.), 1–penten–3–one (0.14 mL, 1.4 mmol, 1.2 eq.) and sodium methoxide (74 mg, 1.4 mmol, 1.2 eq.) in MeOH (2.5 mL). Purification by silica gel column chromatography (hexane/EtOAc 9:1 to 8:2) yielded the title product as a colourless oil (280 mg, 0.81 mmol, 71%).^{1}H NMR (500 MHz, $CDCl_3$) δ 1.06 (t, *J* = 7.3 Hz, 3H, $C(O)CH_2C\mathit{H}_3$), 2.06 (dd, *J* = 11.1, 5.1 Hz, 2H, $C\mathit{H}_2CH_2CH{=}CHAr$), 2.11–2.17 (m, 2H, $CH_2C\mathit{H}_2CH{=}CHAr$), 2.23 (t, *J* = 7.8 Hz, 2H, $C\mathit{H}_2CH_2C(O)CH_2CH_3$), 2.41–2.46 (m, 4H, $CH_2C\mathit{H}_2C(O)CH_2CH_3$ + $CH_2CH_2C(O)C\mathit{H}_2CH_3$), 3.73 (s, 6H, $CO_2C\mathit{H}_3$), 6.16 (dt, *J* = 16.3, 6.5 Hz, 1H, C*H*=CHAr), 6.40 (d, *J* = 15.8 Hz, 1H, CH=C*H*Ar), 7.21 (t, *J* = 7.2 Hz, 1H, ArC*H*), 7.27–7.36 (m, 4H, ArC*H*) ppm; ^{13}C NMR (126 MHz, $CDCl_3$) δ 7.8 ($C(O)CH_2\mathit{C}H_3$), 26.8 ($\mathit{C}H_2CH_2C(O)CH_2CH_3$), 27.7 ($CH_2\mathit{C}H_2CH{=}CHAr$), 33.2 ($\mathit{C}H_2CH_2CH{=}CHAr$), 36.0 ($CH_2CH_2C(O)\mathit{C}H_2CH_3$), 37.3 ($CH_2\mathit{C}H_2C(O)CH_2CH_3$), 52.5 ($CO_2\mathit{C}H_3$), 56.7 ($\mathit{C}_q$), 126.0 (Ar*C*H), 127.1 (Ar*C*H), 128.5 (Ar*C*H), 129.0 (*C*H=CHAr), 130.6 (CH=*C*HAr), 137.4 (Ar*C*), 171.7 ($\mathit{C}O_2CH_3$), 209.87 ($CH_2CH_2\mathit{C}(O)CH_2CH_3$) ppm; IR ν_{max} (thin film, cm^{-1}) = 3053, 2186, 1732 (C=O), 1651, 1435, 1083; HRMS calcd. for $C_{20}H_{26}O_5Na$ $[M+Na]^+$ 369.1972, found 369.1657.

Hept–1–en–3–one (S5)

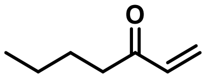

Prepared according to General Procedure C using valeraldehyde (1.06 mL, 10.0 mmol, 1.0 eq.) and vinyl magnesium bromide (11 mL, 1.0 M in THF, 11 mmol, 1.1 eq.) in THF followed by DMP (4.24 g, 10 mmol, 1.5 eq.) in CH_2Cl_2 (33 mL). The crude product was used in the following step without further purification.

Dimethyl (*E*)–2–(3–oxoheptyl)–2–(4–phenylbut–3–en–1–yl)malonate (S6)

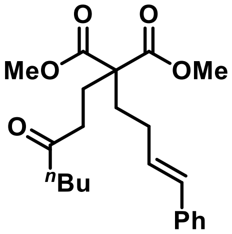

Prepared according to General Procedure B using dimethyl (*E*)–2–(4–phenylbut–3–en–1–yl)malonate **S2** (327 mg, 1.25 mmol, 1.0 eq.), hept–1–en–3–one **S5** (171.4 mg, 1.53 mmol, 1.2 eq.) and sodium methoxide (81 mg, 1.5 mmol, 1.2 eq.) in MeOH (2.6 mL). Purification by silica gel column chromatography (Hexane/EtOAc 9:1) yielded the title product as a yellow oil (202 mg, 0.54 mmol, 43%). ^{1}H NMR (400 MHz, $CDCl_3$) δ 0.90 (t, 3H, *J* = 7.3 Hz, $CH_3(CH_2)_3C(O)$), 1.23–1.34 (m, 2H, $CH_3CH_2CH_2C(O)$), 1.50–1.59 (m, 2H, $CH_3CH_2CH_2CH_2C(O)$), 2.02–2.08 (m, 2H, $CH_2CH_2CH{=}CHAr$), 2.10–2.16 (m, 2H, $CH_2CH_2CH{=}CHAr$), 2.21 (dd, 2H, *J* = 8.9, 6.7 Hz, $CCH_2CH_2C(O)$), 2.41 (dt, 4H, *J* = 9.7, 7.4 Hz, $CCH_2CH_2C(O)CH_2$), 3.72 (s, 6H, 2 x CO_2CH_3), 6.14 (dt, 1H, *J* = 15.8 Hz, 6.5 Hz, C*H*=CHAr) 6.39 (d, 1H, *J* = 15.8 Hz, CH=C*H*Ar), 7.17–7.24 (m, 1H, ArC*H*), 7.27–7.34 (m, 4H, ArC*H*) ppm; ^{13}C NMR (101 MHz, $CDCl_3$) δ 13.8 ($CH_3(CH_2)_3C(O)$), 22.3 ($CH_3CH_2CH_2C(O)$), 25.9 ($CH_3CH_2CH_2CH_2C(O)$), 26.8 ($CH_2CH_2C(O)$), 27.8 ($CH_2CH_2CH{=}CHAr$), 33.2 ($CH_2CH_2CH{=}CHAr$), 37.7 ($CH_2CH_2C(O)$), 42.6 ($CH_3(CH_2)_2CH_2C(O)$), 52.5 (CO_2CH_3), 56.7 (C_q), 126.0 (Ar*C*H), 127.1 (Ar*C*H), 128.5 (Ar*C*H), 129.0 (*C*H=CHAr), 130.6 (CH=*C*HAr), 137.4 (Ar*C*), 171.7 (CO_2CH_3), 209.6 (*C*(O)) ppm; IR ν_{max} (thin film, cm^{-1}) = 2955, 1731, 1497, 1448, 1434, 1209, 1175, 1107, 1071, 967, 745, 587; HRMS calculated for $C_{22}H_{30}O_5$ $[M+H]^+$ 375.2166, found 375.2157.

1–Phenylbut–3–en–2–one (S7)[3]

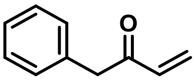

Prepared according to General Procedure C using phenylacetaldehyde (1.2 g, 10 mmol, 1.0 eq.) and vinyl magnesium bromide (11 mL, 1.0 M in THF, 11 mmol, 1.1 eq.) in THF (25 mL) followed by DMP (5.1 g, 12 mmol, 1.2 eq.) in CH_2Cl_2 (38 mL). The crude product was used in the following step without further purification.

Dimethyl (*E*)–2–(3–oxo–4–phenylbutyl)–2–(4–phenylbut–3–en–1–yl)malonate (S8)

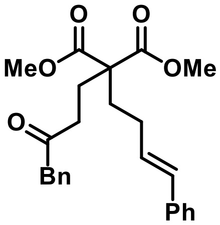

Prepared according to General Procedure B using dimethyl (*E*)–2–(4–phenylbut–3–en–1–yl)malonate **S2** (420 mg, 1.6 mmol, 1.0 eq.), 1–phenylbut–3–en–2–one (240 mg, 1.6 mmol, 1.0 eq.) and sodium methoxide (106 mg, 2.0 mmol, 1.2 eq.) in MeOH (3.5 mL). Purification by silica gel column chromatography

(hexane/EtOAc 9:1) yielded the title product as a yellow oil (277 mg, 0.68 mmol, 41%). ^{1}H NMR (400 MHz, $CDCl_3$) δ 1.97–2.04 (m, 2H, $C\mathit{H}_2CH_2CH$=CHAr), 2.05–2.13 (m, 2H, $CH_2C\mathit{H}_2CH$=CHAr), 2.17–2.23 (m, 2H, $C\mathit{H}_2CH_2C(O)$)), 2.49 (dd, *J* = 8.8, 6.8 Hz, 2H, $CH_2C\mathit{H}_2C(O)$)), 3.68 (s, 6H, $CO_2C\mathit{H}_3$), 3.70 (s, 2H, $C(O)C\mathit{H}_2Ar$), 6.12 (dt, *J* = 15.8, 6.5 Hz, 1H, C*H*=CHAr), 6.36 (d, *J* = 15.8 Hz, 1H, CH=C*H*Ar), 7.13–7.37 (m, 10H, ArC*H*) ppm; ^{13}C NMR (101 MHz, $CDCl_3$) δ 26.8 ($\mathit{C}H_2CH_2C(O)$)), 27.7 ($CH_2\mathit{C}H_2CH$=CHAr), 33.1 ($\mathit{C}H_2CH_2CH$=CHAr), 37.0 ($CH_2\mathit{C}H_2C(O)$)), 50.1 ($C(O)\mathit{C}H_2Ar$), 52.5 ($CO_2\mathit{C}H_3$), 56.6 (C_q), 126.0 (Ar*C*H), 127.1 (Ar*C*H), 128.5 (Ar*C*H), 128.8 (Ar*C*H), 128.9 (*C*H=CHAr), 129.4 (Ar*C*H), 130.6 (CH=*C*HAr), 134.0 (Ar*C*), 137.4 (Ar*C*), 171.6 ($\mathit{C}O_2CH_3$), 206.8 (*C*(O)) ppm; IR ν_{max} (thin film, cm^{-1}) = 2949, 1729 (C=O), 1495, 1454, 1202, 1118, 1072; HRMS calcd. for $C_{25}H_{28}O_5Na$ $[M+Na]^+$ 431.1829, found 431.1818.

5,5–Dimethylhex–1–en–3–one (S9)[4]

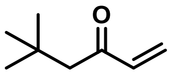

Prepared according to General Procedure C using 3,3–dimethyl butyraldehyde (1.3 mL, 10 mmol, 1.0 eq.) and vinyl magnesium bromide (11 mL, 1.0 M in THF, 11 mmol, 1.1 eq.) in THF (25 mL) followed by DMP (5.1 g, 12 mmol, 1.2 eq.) in CH_2Cl_2 (38 mL). The crude product was used in the following step without further purification.

Dimethyl (*E*)–2–(5,5–dimethyl–3–oxohexyl)–2–(4–phenylbut–3–en–1–yl)malonate (S10)

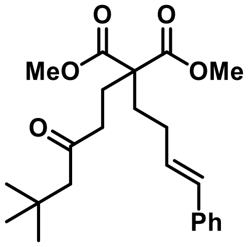

Prepared according to General Procedure B using dimethyl (*E*)–2–(4–phenylbut–3–en–1–yl)malonate **S2** (300 mg, 1.1 mmol 1.0 eq.), 5,5–dimethylhex–1–en–3–one (173 mg, 1.4 mmol, 1.2 eq.) and sodium methoxide (0.074 g, 1.4 mmol, 1.2 eq.) in methanol (2.3 mL). Purification by silica gel column chromatography (hexane/EtOAc 9:1 to CH_2Cl_2) yielded the title product as a colourless oil (176 mg, 0.52 mmol, 40%). ^{1}H NMR (400 MHz, $CDCl_3$) δ 1.01 (s, 9H, $C(C\mathit{H}_3)_3$), 1.99–2.09 (m, 2H, $C\mathit{H}_2CH_2CH$=CHAr), 2.09–2.44 (m, 4H, $CH_2C\mathit{H}_2CH$=CHAr + $C\mathit{H}_2CH_2C(O)$)), 2.30 (s, 2H, $C(O)C\mathit{H}_2C(CH_3)_3$), 2.41 (dd, *J* = 9.1, 6.4 Hz, 2H, $CH_2C\mathit{H}_2C(O)$)), 3.73 (s, 6H, $CO_2C\mathit{H}_3$), 6.15 (dt, *J* = 15.9, 6.3 Hz, 1H, C*H*=CHAr), 6.39 (d, *J* = 15.8 Hz, 1H, CH=C*H*Ar), 7.17–7.24 (m, 1H, ArC*H*), 7.28–7.36 (m, 4H, ArC*H*) ppm; ^{13}C NMR (101 MHz, $CDCl_3$) δ 26.7 ($\mathit{C}H_2CH_2C(O)$)), 27.8 ($CH_2\mathit{C}H_2CH$=CHAr), 29.7 ($C(\mathit{C}H_3)_3$), 31.0 ($\mathit{C}(CH_3)_3$), 33.2 ($\mathit{C}H_2CH_2CH$=CHAr), 40.0 ($CH_2\mathit{C}H_2C(O)$)), 52.5 ($CO_2\mathit{C}H_3$), 55.0 ($C(O)\mathit{C}H_2(C(CH_3)_3)$), 56.6 ($\mathit{C}_q$), 126.0 (Ar*C*H), 127.1 (Ar*C*H), 128.5 (Ar*C*H), 129.0 (*C*H=CHAr), 130.6 (CH=*C*HAr), 137.4 (Ar*C*), 171.7 ($\mathit{C}O_2CH_3$), 209.2 (*C*(O)) ppm; IR ν_{max} (thin film, cm^{-1}) = 2951, 1732 (C=O), 1447, 1363, 1247, 1225, 1174, 1074; HRMS calcd. for $C_{23}H_{32}O_5Na$ $[M+Na]^+$ 411.2142, found 411.2129.

4–Methylpent–1–en–3–one (S11)[5]

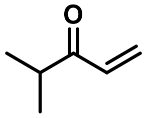

Prepared according to General Procedure C using isobutyraldehyde (0.91 mL, 10 mmol, 1.0 eq.) and vinyl magnesium bromide (11 mL, 1.0 M in THF, 11 mmol, 1.1 eq.) in Et_2O (25 mL) followed by DMP (5.1 g, 12 mmol, 1.2 eq.) in CH_2Cl_2 (38 mL). The crude product was used in the following step without further purification.

Dimethyl (*E*)–2–(4–methyl–3–oxopentyl)–2–(4–phenylbut–3–en–1–yl)malonate (S12)

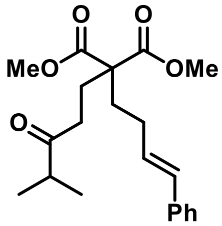

Prepared according to General Procedure B using dimethyl (*E*)–2–(4–phenylbut–3–en–1–yl)malonate **S2** (392 mg, 1.5 mmol, 1.0 eq.), 4–methylpent–1–en–3–one (220 mg, 2.2 mmol, 1.5 eq.) and sodium methoxide (121 mg, 2.2 mmol, 1.5 eq.) in methanol (3.5 mL). Purification by silica gel column chromatography (hexane/EtOAc 9:1) yielded the title product as a colourless oil (248 mg, 0.69 mmol, 46%). ^{1}H NMR (500 MHz, $CDCl_3$) δ 1.10 (d, *J* = 7.0 Hz, 6H, CH(C*H*$_3$)$_2$), 2.04–2.09 (m, 2H, C*H*$_2$CH$_2$CH=CHAr), 2.11–2.18 (m, 2H, CH$_2$C*H*$_2$CH=CHAr), 2.19–2.25 (m, 2H, C*H*$_2$CH$_2$C(O)), 2.45–2.51 (m, 2H, CH$_2$C*H*$_2$C(O)), 2.60 (hept, *J* = 6.9 Hz, 1H, C*H*(CH$_3$)$_2$), 3.73 (s, 6H, CO$_2$C*H*$_3$), 6.16 (dt, *J* = 15.8, 6.6 Hz, 1H, C*H*=CHAr), 6.40 (CH=C*H*Ar), 7.17–7.25 (m, 1H, ArC*H*), 7.29–7.37 (m, 4H, ArC*H*) ppm; ^{13}C NMR (101 MHz, $CDCl_3$) δ 18.3 (CH(*C*H$_3$)$_2$), 26.9 (*C*H$_2$CH$_2$C(O)), 27.8 (CH$_2$*C*H$_2$CH=CHAr), 33.3 (*C*H$_2$CH$_2$CH=CHAr), 35.4 (CH$_2$*C*H$_2$C(O)), 41.0 (*C*H(CH$_3$)$_2$), 52.5 (CO$_2$*C*H$_3$), 56.7 (*C$_q$*), 126.0 (Ar*C*H), 127.1 (Ar*C*H), 128.5 (Ar*C*H), 129.0 (*C*H=CHAr), 130.6 (CH=*C*HAr), 137.4 (Ar*C*), 171.7 (*C*O$_2$CH$_3$), 213.2 (*C*(O)) ppm; IR ν_{max} (thin film, cm^{-1}) = 2951, 1728 (C=O), 1683, 1597, 1447, 1196, 1174, 967; HRMS calcd. for $C_{21}H_{28}O_5Na$ [M+Na]$^+$ 383.1829, found 383.1816.

1,1–Diphenylbut–3–en–2–one (S13)

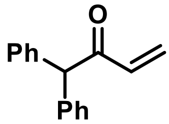

Prepared according to General Procedure C using 2,2–diphenylacetaldehyde (1.78 mL, 10.0 mmol, 1.0 eq.) and vinyl magnesium bromide (11.0 mL, 1.0 M in THF, 11.0 mmol, 1.1 eq.) in THF (25 mL) followed by DMP (1.73 g, 4.08 mmol, 1.5 eq.) in CH_2Cl_2 (14 mL). The crude product was used in the following step without further purification.

Dimethyl (*E*)–2–(3–oxo–4,4–diphenylbutyl)–2–(4–phenylbut–3–en–1–yl)malonate (S14)

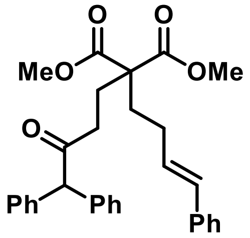

Prepared according to General Procedure B using dimethyl (*E*)–2–(4–phenylbut–3–en–1–yl)malonate **S2** (327 mg, 1.25 mmol, 1.0 eq.), 1,1–diphenylbut–3–en–2–one **ZZ** (333 mg, 1.5 mmol, 1.2 eq.) and sodium methoxide (81 mg, 1.5 mmol, 1.2 eq.) in MeOH (2.5 mL). Purification by silica gel column chromatography (Hexane/EtOAc 9:1) yielded the title product as a yellow oil (400 mg, 0.83 mmol, 66%). ^{1}H NMR (400 MHz, $CDCl_3$) δ 1.90–1.96 (m, 2H, C*H*$_2$CH$_2$CH=CHAr), 1.97–2.04 (m, 2H, CH$_2$C*H*$_2$CH=CHAr), 2.16–2.22 (m, 2H, C*H*$_2$CH$_2$C(O)), 2.49–2.54 (m, 2H, CH$_2$C*H*$_2$C(O)), 3.59 (s, 6H, CO$_2$C*H*$_3$), 5,08 (s, 1H, C*H*(Ph)$_2$), 6.04 (dt, 1H, J = 15.9, 6.4 Hz, C*H*=CHAr) 6.27 (dt, 1H, J = 16.0, 1.3 Hz, CH=C*H*Ar), 7.13–7.25 (m, 10H, ArC*H*), 7.26–7.31 (m, 5H, ArC*H*) ppm; ^{13}C NMR (101 MHz, $CDCl_3$) δ 27.0 (CH$_2$*C*H$_2$CH=CHAr), 27.7 (*C*H$_2$CH$_2$C(O)), 32.9 (*C*H$_2$CH$_2$CH=CHAr), 37.8 (CH$_2$*C*H$_2$C(O)), 52.5 (CO$_2$*C*H$_3$), 56.6 (*C$_q$*), 64.3 (*C*H(Ph)$_2$) 126.0 (Ar*C*H), 127.1 (Ar*C*H), 127.3 (Ar*C*H), 128.5 (*C*H=CHAr), 128.7 (Ar*C*H), 129.0 (Ar*C*H), 128.9 (Ar*C*H), 130.6 (CH=*C*HAr), 137.4 (Ar*C*), 138.2 (Ar*C*), 171.6 (*C*O$_2$CH$_3$), 207.3 (*C*(O)) ppm; IR ν_{max} (thin film, cm^{-1}) = 3027, 2951, 1729, 1598, 1495, 1452, 1434, 1224, 1196, 1175, 1080, 1031, 967, 745, 702; HRMS calculated for $C_{31}H_{32}O_5$ [M+H]$^+$ 485.2323, found 485.2323.

1–Cyclohexylprop–2–en–1–one (S15)[6]

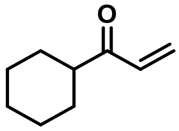

Prepared according to General Procedure C using cyclohexanecarboxaldehyde (1.2 mL, 10 mmol, 1.0 eq.) and vinyl magnesium bromide (11 mL, 1.0 M in THF, 11 mmol, 1.1 eq.) in THF (25 mL), followed by Dess–Martin Periodinane (5.1 g, 12 mmol, 1.2 eq.) in CH_2Cl_2 (38 mL).The crude product was used in the following step without further purification.

Dimethyl (*E*)–2–(3–cyclohexyl–3–oxopropyl)–2–(4–phenylbut–3–en–1–yl)malonate (S16)

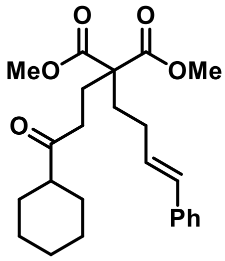

Prepared according to General Procedure B using dimethyl (*E*)–2–(4–phenylbut–3–en–1–yl)malonate **S2** (157 mg, 0.60 mmol, 1.0 eq.), 1–cyclohexylprop–2–en–1–one (100 mg, 0.6 mmol, 1.0 eq.) and sodium methoxide (39 mg, 0.72 mmol, 1.2 eq.) in MeOH (1.3 mL) at 65 °C. Purification by silica gel column chromatography (hexane/EtOAc 95:5) yielded the title product as a pale yellow oil (87 mg, 0.22 mmol, 36%).

^{1}H NMR (500 MHz, $CDCl_3$) δ 1.14–1.41 (m, 5H, 2.5 × CyC*H*$_2$), 1.64–1.71 (m, 1H, 0.5 × CyC*H*$_2$), 1.74–1.87 (m, 4H, 2 × CyC*H*$_2$), 2.03–2.09 (m, 2H, C*H*$_2$CH$_2$CH=CHAr), 2.11–2.15 (m, 2H, CH$_2$C*H*$_2$CH=CHAr), 2.17–2.23 (m, 2H, C*H*$_2$CH$_2$C(O)), 2.33 (tt, *J* = 11.2, 3.4 Hz, 1H, CyC*H*), 2.46 (dd, *J* = 8.9, 6.7 Hz, 2H, CH$_2$C*H*$_2$C(O)), 6.15 (dt, *J* = 15.8, 6.6 Hz, 1H, C*H*=CHAr), 6.40 (d, *J* = 15.9 Hz, 1H, CH=C*H*Ar), 7.18–7.24 (m, 1H, ArC*H*), 7.28–7.35 (m, 4H, ArC*H*) ppm; ^{13}C NMR (101 MHz, $CDCl_3$) δ 25.7 (Cy*C*H$_2$), 25.8 (Cy*C*H$_2$), 26.8 (*C*H$_2$CH$_2$C(O)), 27.8 (CH$_2$*C*H$_2$CH=CHAr), 28.5 (Cy*C*H$_2$), 33.2 (*C*H$_2$CH$_2$CH=CHAr), 35.6 (CH$_2$*C*H$_2$C(O)), 50.9 (Cy*C*H), 52.5 (CO$_2$*C*H$_3$), 56.7 (*C$_q$*), 126.0 (Ar*C*H), 127.1 (Ar*C*H), 128.5 (Ar*C*H), 129.0 (*C*H=CHAr), 130.6 (CH=*C*HAr), 137.4 (Ar*C*), 171.8 (*C*O$_2$CH$_3$), 212.5 (*C*(O)) ppm; IR ν_{max} (thin film, cm^{-1}) = 2933, 1729 (C=O), 1451, 1240, 1196, 1163, 1124, 1090; HRMS calcd. for $C_{24}H_{32}O_5Na$ $[M+Na]^+$ 423.2142, found 423.2131.

1–(Tetrahydro–2*H*–pyran–4–yl)prop–2–en–1–one (S17)

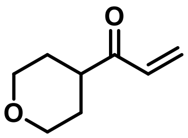

Prepared according to General Procedure C using tetrahydro–2*H*–pyran–4–carbaldehyde (0.52 mL, 5.0 mmol, 1.0 eq.) and vinyl magnesium bromide (5.5 mL, 1.0 M in THF, 5.5 mmol, 1.1 eq.) in THF followed by DMP (3.12 g, 7.4 mmol, 1.5 eq.) in CH_2Cl_2 (25 mL). The crude product was used in the following step without further purification.

Dimethyl (*E*)–2–(3–oxo–3–(tetrahydro–2*H*–pyran–4–yl)propyl)–2–(4–phenylbut–3–en–1–yl) malonate (S18)

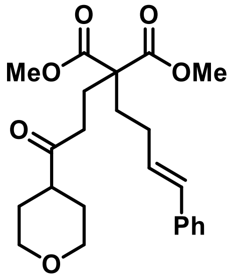

Prepared according to General Procedure B using dimethyl (*E*)–2–(4–phenylbut–3–en–1–yl)malonate **S2** (262 mg, 1.17 mmol, 1.0 eq.), 1–(tetrahydro–2*H*–pyran–4–yl)prop–2–en–1–one **S17** (196 mg, 1.4 mmol, 1.2 eq.) and sodium methoxide (76 mg, 1.4 mmol, 1.2 eq.) in MeOH (2.5 mL). Purification by silica gel column chromatography (Hexane/EtOAc 9:1) yielded the title product as a yellow oil (249 mg, 0.63 mmol, 54%). ^{1}H NMR (400 MHz, $CDCl_3$) δ 1.64–1.78 (m, 4H, (C*H*$_2$CH$_2$OCH$_2$C*H*$_2$), 2.02–2.09 (m, 2H, C*H*$_2$CH$_2$CH=CHAr), 2.10–2.18 (m, 2H, CH$_2$C*H*$_2$CH=CHAr), 2.21 (dd, 2H, *J* = 8.5, 6.8 Hz, C*H*$_2$CH$_2$C(O)), 2.46–2.51 (m, 2H, CH$_2$C*H*$_2$C(O)), 2.58–2.52 (m, 1H, C*H*C(O)), 3.40 (td, 2H, *J* = 11.3, 3.0 Hz, CH$_2$OC*H*$_2$), 3.72 (s, 6H, CO$_2$C*H*$_3$), 3.99 (ddd, 2H, *J* = 11.6, 4.2, 2.6 Hz, C*H*$_2$OCH$_2$), 6.14 (dt, 1H, *J* = 15.8 Hz, 6.6 Hz, C*H*=CHAr) 6.39 (d, 1H, *J* = 16.0 Hz, CH=C*H*Ar), 7.23–7.17 (m, 1H, ArC*H*), 7.34–7.28 (m, 4H, ArC*H*) ppm; ^{13}C NMR (101 MHz, $CDCl_3$) δ 26.7 (*C*H$_2$CH$_2$C(O)), 27.8 (CH$_2$*C*H$_2$CH=CHAr), 28.2 (*C*H$_2$CH$_2$OCH$_2$*C*H$_2$), 33.4 (*C*H$_2$CH$_2$CH=CHAr), 35.4 (CH$_2$*C*H$_2$C(O)), 47.6 (*C*HC(O)), 52.5 (CO$_2$*C*H$_3$), 56.7 (*C$_q$*), 67.2 (*C*H$_2$O*C*H$_2$), 126.0 (Ar*C*H), 127.1 (Ar*C*H), 128.5 (Ar*C*H), 128.9 (*C*H=CHAr), 130.7 (CH=*C*HAr), 137.4 (Ar*C*), 171.7 (*C*O$_2$CH$_3$), 210.4 (*C*(O)) ppm; IR ν_{max} (thin film, cm^{-1}) = 2953, 2847, 1729, 1710, 1445,

1387, 1239, 1197, 1176, 1118, 1093, 1019, 968, 746; HRMS calculated for $C_{23}H_{31}O_6$ $[M+H]^+$ 403.2115 found 403.2111.

4,4–Dimethylpent–1–en–3–one (S19)[7]

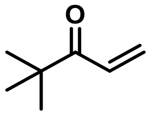

Prepared according to General Procedure C using pivaldehyde (1.1 mL, 10 mmol, 1.0 eq.) and vinyl magnesium bromide (11 mL, 1.0 M in THF, 11 mmol, 1.1 eq.) in Et_2O (25 mL) followed by DMP (5.1 g, 12 mmol, 1.2 eq.) in CH_2Cl_2 (38 mL). The crude product was used in the following step without further purification.

Dimethyl (*E*)–2–(4,4–dimethyl–3–oxopentyl)–2–(4–phenylbut–3–en–1–yl)malonate (S20)

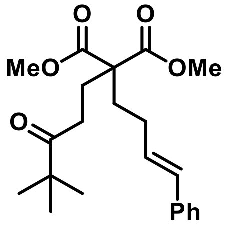

Prepared according to a modified version of General Procedure B using dimethyl (*E*)–2–(4–phenylbut–3–en–1–yl)malonate **S2** (351 mg, 1.3 mmol, 1.0 eq.), 4,4–dimethylpent–1–en–3–one (226 mg, 2.0 mmol, 1.5 eq.) and K_2CO_3 (272 mg, 2.0 mmol, 1.5 eq.) in 1,4–dioxane (3.4 mL) at 100 °C. Purification by silica gel column chromatography (hexane/EtOAc 9:1) yielded the title product as a yellow oil (415 mg, 1.1 mmol, 83%). ^{1}H NMR (400 MHz, $CDCl_3$) δ 1.14 (s, 9H, C(C*H*$_3$)$_3$), 2.03–2.11 (m, 2H, C*H*$_2$CH$_2$CH=CHAr), 2.11–2.23 (m, 4H, CH$_2$C*H*$_2$CH=CHAr + C*H*$_2$CH$_2$C(O)), 2.46–2.53 (m, 2H, CH$_2$C*H*$_2$C(O)), 3.73 (s, 6H, CO$_2$C*H*$_3$), 6.16 (dt, *J* = 15.8, 6.6 Hz, 1H, C*H*=CHAr), 6.40 (d, *J* = 15.7 Hz, 1H, CH=C*H*Ar), 7.17–7.23 (m, 1H, ArC*H*), 7.27–7.36 (m, 4H, ArC*H*) ppm; ^{13}C NMR (101 MHz, $CDCl_3$) δ 26.4 (C(*C*H$_3$)$_3$), 27.4 (*C*H$_2$CH$_2$C(O)), 27.8 (CH$_2$*C*H$_2$CH=CHAr), 31.5 CH$_2$*C*H$_2$C(O)), 33.3 (*C*H$_2$CH$_2$CH=CHAr), 44.3 (*C*(CH$_3$)$_3$), 52.5 (CO$_2$*C*H$_3$), 56.8 (C_q), 126.0 (Ar*C*H), 127.1 (Ar*C*H), 128.5 (Ar*C*H), 129.1 (*C*H=CHAr), 130.6 (CH=*C*HAr), 137.4 (Ar*C*), 171.8 (*C*O$_2$CH$_3$), 214.6 (*C*(O)) ppm; IR ν_{max} (thin film, cm^{-1}) = 2973, 1732 (C=O), 1694, 1434, 1202, 1178, 967; HRMS calcd. for $C_{22}H_{30}O_5Na$ $[M+Na]^+$ 397.1985, found 397.1974.

Dimethyl 2–(but–3–en–1–yl)–2–(3–oxopentyl)malonate (S21)

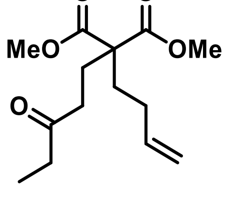

Prepared according to General Procedure B using dimethyl 2–(but–3–en–1–yl)malonate **S1** (3.4 g, 18 mmol, 1.0 eq.), 1–penten–3–one (2.2 mL, 22 mmol, 1.2 eq.) and sodium methoxide (1.2 g, 22 mmol, 1.2 eq.) in

methanol (57 mL). Purification by silica gel column chromatography (PE/EtOAc 9:1) yielded the title product as a colourless oil (2.6 g, 9.7 mmol, 53%). ^{1}H NMR (400 MHz, $CDCl_3$) δ 1.06 (t, *J* = 7.3 Hz, 3H, C(O)CH_2C*H_3*), 1.93–2.01 (m, 4H, C*H_2*$CH_2CH{=}CH_2$ + CH_2C*H_2*$CH{=}CH_2$), 2.16–2.23 (m, 2H, C*H_2*CH_2C(O)), 2.38–2.48 (m, 4H, CH_2C*H_2*C(O) + C(O)C*H_2*CH_3), 3.72 (s, 6H, CO_2C*H_3*), 4.95–5.09 (m, 2H, CH=C*H_2*), 5.71–5.84 (m, 1H, C*H*=CH_2) ppm; ^{13}C NMR (101 MHz, $CDCl_3$) δ 7.8 (C(O)CH_2*C*H_3), 26.8 (C*H_2*CH_2C(O)), 28.4 (CH_2*C*H_2CH=CH), 32.8 (C*H_2*CH_2CH=CH), 36.0 (C(O)*C*H_2CH_3), 37.3 (CH_2*C*H_2C(O)), 52.5 (CO_2*C*H_3), 115.2 (CH=*C*H_2), 137.3 (*C*H=CH_2), 171.7 (*C*O_2CH_3), 209.9 (*C*(O)) ppm; IR ν_{max} (thin film, cm^{-1}) = 2953, 1729 (C=O), 1641, 1455, 1111, 1048, 1021; HRMS calcd. for $C_{14}H_{22}O_5Na$ $[M+Na]^+$ 293.1359, found 293.1346.

Dimethyl (*E*)–2–(4–(2–fluorophenyl)but–3–en–1–yl)–2–(3–oxopentyl)malonate (S22)

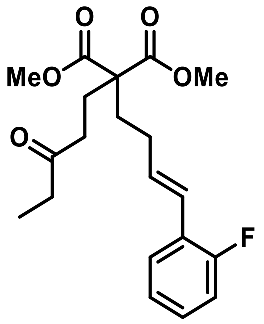

Prepared according to General Procedure A using dimethyl 2–(but–3–en–1–yl)–2–(3–oxopentyl)malonate **S21** (250 mg, 0.92 mmol, 1.0 eq.), 2–fluorostyrene (0.33 mL, 3.0 mmol, 3.0 eq.) and Grubbs I catalyst (38 mg, 0.046 mmol, 5 mol%) in CH_2Cl_2 (4 mL). Purification by silica gel column chromatography (hexane/EtOAc 100:0 to 9:1) yielded the title product as a yellow oil (151 mg, 0.41 mmol, 45%). ^{1}H NMR (500 MHz, $CDCl_3$) δ 1.06 (t, *J* = 7.3 Hz, 3H, C(O)CH_2C*H_3*), 2.02–2.10 (m, 2H, C*H_2*CH_2CH=CH), 2.13–2.20 (m, 2H, CH_2C*H_2*CH=CH), 2.23 (dd, *J* = 8.9, 6.7 Hz, 2H, C*H_2*CH_2C(O)), 2.41–2.47 (m, 4H, C(O)C*H_2*CH_3 + CH_2C*H_2*C(O)), 3.73 (s, 6H, CO_2C*H_3*), 6.24 (dt, *J* = 16.0, 6.6 Hz, 1H, C*H*=CHAr), 6.54 (d, *J* = 16.0 Hz, 1H, CH=C*H*Ar), 7.01 (ddd, *J* = 10.9, 8.2, 1.3 Hz, 1H, ArC*H*), 7.07 (td, *J* = 7.5, 1.2 Hz, 1H, ArC*H*), 7.18 (m, 1H, ArC*H*), 7.40 (td, *J* = 7.7, 1.8 Hz, 1H, ArC*H*) ppm; ^{13}C NMR (126 MHz, $CDCl_3$) δ 7.8 (C(O)CH_2*C*H_3), 26.8 (*C*H_2CH_2C(O)), 28.2 (CH_2*C*H_2CH=CH), 33.1 (*C*H_2CH_2CH=CH), 36.0 (C(O)*C*H_2CH_3), 37.3 (CH_2*C*H_2C(O)), 52.5 (CO_2*C*H_3), 56.7 (C_q), 115.7 (d, *J* = 22.3 Hz, Ar*C*H), 123.1 (CH=*C*HAr), 124.0 (d, *J* = 12.2 Hz, Ar*C*H), 125.1 (d, *J* = 12.2 Hz, Ar*C*H), 127.1 (*C*H=CHAr), 128.3 (d, *J* = 8.4 Hz, Ar*C*H), 131.8 (d, *J* = 4.8 Hz, Ar*C*), 160.0 (d, *J* = 248.5 Hz, Ar*C*F), 171.7 (*C*O_2CH_3), 209.9 (*C*(O)) ppm; ^{19}F NMR (376 MHz, $CDCl_3$) δ –118.6 ppm; IR ν_{max} (thin film, cm^{-1}) = 2952, 1730 (C=O), 1487, 1455, 1227, 1194, 1090; HRMS calcd. for $C_{20}H_{25}O_5FNa$ $[M+Na]^+$ 387.1578, found 387.1561.

Dimethyl (*E*)–2–(3–oxopentyl)–2–(4–(o–tolyl)but–3–en–1–yl)malonate (S23)

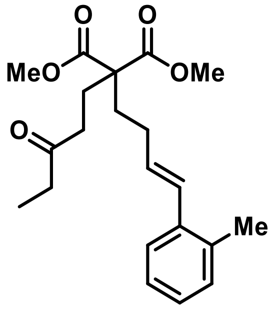

Prepared according to General Procedure A using dimethyl 2–(but–3–en–1–yl)–2–(3–oxopentyl)malonate **S21** (270 mg, 1.0 mmol, 1.0 eq.), 1–methylstyrene (0.39 mL, 3.0 mmol, 3.0 eq.) and Grubbs I catalyst (41 mg, 0.05 mmol, 5 mol%) in CH_2Cl_2 (4 mL). Purification by silica gel column chromatography (Hexane/EtOAc 10:0 to 9:1) yielded the title product as a dark oil (107 mg, 0.30 mmol, 30%). ^{1}H NMR (500 MHz, $CDCl_3$) δ 1.05 (t, 3H, *J* = 7.3 Hz, C(O)$CH_2C\mathit{H}_3$), 2.03–2.09 (m, 2H, $C\mathit{CH}_2CH_2$CH=CH), 2.11–2.19 (m, 2H, $CH_2C\mathit{H}_2$CH=CH), 2.22 (dd, 2H, *J* = 8.9, 6.7 Hz, $CC\mathit{H}_2CH_2$C(O)), 2.32 (s, 3H, $C\mathit{H}_3$Ar), 2.43 (q, 4H, *J* = 7.1 Hz, C(O)$C\mathit{H}_2CH_3$ + $CCH_2C\mathit{H}_2$C(O)), 3.72 (s, 6H, $CO_2C\mathit{H}_3$), 6.01 (dt, 1H, *J* = 15.7, 6.6 Hz, C*H*=CHAr), 6.58 (d, 1H, *J* = 15.7, 1.7 Hz, CH=C*H*Ar), 7.10–7.16 (m, 3H, ArC*H*), 7.35–7.40 (m, 1H, ArC*H*) ppm; ^{13}C NMR (126 MHz, $CDCl_3$) δ 7.8 (C(O)$CH_2\mathit{C}H_3$), 19.8 ($\mathit{C}H_3$Ar), 26.9 ($C\mathit{C}H_2CH_2$C(O)), 28.1 ($CH_2\mathit{C}H_2$CH=CH), 33.4 ($C\mathit{C}H_2CH_2$CH=CH), 36.0 (C(O)$\mathit{C}H_2CH_3$), 37.4 ($CCH_2\mathit{C}H_2$C(O)), 52.5 ($CO_2\mathit{C}H_3$), 56.7 (C_q), 125.4 (Ar*C*H), 126.0 (Ar*C*H), 127.0 (Ar*C*H), 128.5 (CH=*C*HAr), 130.2 (Ar*C*H), 130.4 (*C*H=CHAr), 135.0 (Ar*C*Me), 136.5 (Ar*C*), 171.7 ($\mathit{C}O_2CH_3$), 209.9 (*C*(O)) ppm; IR ν_{max} (thin film, cm^{-1}) = 2955, 2160, 1731, 1435, 1376, 1201, 1114, 969, 804, 753; HRMS calculated for $C_{21}H_{28}O_5Na$ $[M+Na]^+$: 383.1829, found 383.1815.

Dimethyl (*E*)–2–(3–oxopentyl)–2–(4–(m–tolyl)but–3–en–1–yl)malonate (S24)

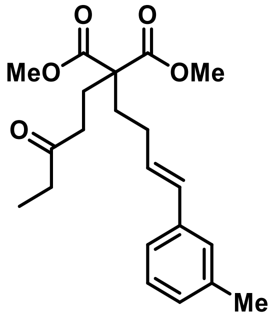

Prepared according to General Procedure A using dimethyl 2–(but–3–en–1–yl)–2–(3–oxopentyl)malonate **S21** (300 mg, 1.1 mmol, 1.0 eq.), 3–methylstyrene (0.44 mL, 3.3 mmol, 3.0 eq.) and Grubbs I catalyst (45 mg, 0.055 mmol, 5 mol%) in CH_2Cl_2 (4 mL). Purification by silica gel column chromatography (Hexane/EtOAc 100:0 to 9:1) yielded the title product as a yellow oil (136 mg, 0.38 mmol, 34%). ^{1}H NMR (500 MHz, $CDCl_3$) δ 1.05 (t, 3H, *J* = 7.3 Hz, C(O)$CH_2C\mathit{H}_3$), 2.02–2.08 (m, 2H, $C\mathit{H}_2CH_2$CH=CH), 2.09–2.15 (m, 2H, $CH_2C\mathit{H}_2$CH=CH), 2.22 (dd, 2H, *J* = 8.9, 6.6 Hz, $C\mathit{H}_2CH_2$C(O)), 2.33 (s, 3H, $C\mathit{H}_3$Ar) 2.39–2.46 (m, 4H, C(O)$C\mathit{H}_2CH_3$ + $CCH_2C\mathit{H}_2$C(O)), 3.72 (s, 6H, $CO_2C\mathit{H}_3$), 6.13 (dt, 1H, *J* = 15.9, 6.5 Hz, C*H*=CHAr), 6.36 (d, 1H, *J* = 15.8 Hz, CH=C*H*Ar), 7.02 (d, 1H, *J* = 7.4 Hz, ArC*H*), 7.10–7.21 (m, 3H, ArC*H*) ppm; ^{13}C NMR (126 MHz, $CDCl_3$) δ 7.8 (C(O)$CH_2\mathit{C}H_3$), 21.4 ($\mathit{C}H_3$Ar), 26.8 ($\mathit{C}H_2CH_2$C(O)), 27.7 ($CH_2\mathit{C}H_2$CH=CH), 33.2 ($\mathit{C}H_2CH_2$CH=CH), 36.0 ($CH_2\mathit{C}H_2$C(O)), 37.3 (C(O)$\mathit{C}H_2CH_3$), 52.5 ($CO_2\mathit{C}H_3$), 56.7 (C_q), 123.1 (Ar*C*H), 126.7 (Ar*C*H), 127.9 (Ar*C*H), 128.4 (Ar*C*H), 128.8 (*C*H=CHAr), 130.7 (CH=*C*HAr), 137.4 (Ar*C*Me), 138.0 (Ar*C*), 171.7 ($\mathit{C}O_2CH_3$), 209.9 (*C*(O)) ppm; IR ν_{max} (thin film, cm^{-1}) = 2953, 1729, 1603, 1434, 1376, 1222,

1196, 1176, 1109, 1038, 966, 890, 775, 694; HRMS calculated for $C_{21}H_{29}O_5$ $[M+H]^+$ 361.2010, found 361.2005.

Dimethyl (*E*)–2–(4–(3–chlorophenyl)but–3–en–1–yl)–2–(3–oxopentyl)malonate (S25)

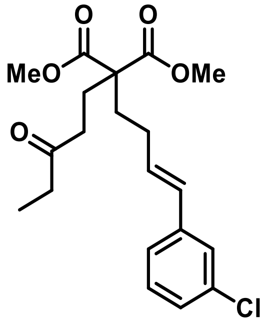

Prepared according to General Procedure A using dimethyl 2–(but–3–en–1–yl)–2–(3–oxopentyl)malonate **S21** (400 mg, 1.5 mmol, 1.0 eq.), 3–chlorostyrene (0.56 mL, 4.4 mmol, 3.0 eq.) and Grubbs I catalyst (61 mg, 0.074 mmol, 5 mol%) in CH_2Cl_2 (7 mL). Purification by silica gel column chromatography (hexane/EtOAc 100:0 to 9:1) yielded the title product as a colourless oil (169 mg, 0.43 mmol, 29%). ^{1}H NMR (400 MHz, $CDCl_3$) δ 1.06 (t, *J* = 7.4 Hz, 3H, C(O)$CH_2C\mathit{H_3}$), 2.01–2.09 (m, 2H, C$\mathit{H_2}$$CH_2$CH=CH), 2.13–2.17 (m, 2H, CH_2C$\mathit{H_2}$CH=CH), 2.22 (dd, *J* = 8.9, 6.6 Hz, 2H, C$\mathit{H_2}$$CH_2$C(O)), 2.38–2.48 (m, 4H, C(O)C$\mathit{H_2}$$CH_3$ + CH_2C$\mathit{H_2}$C(O)), 3.73 (s, 6H, CO_2C$\mathit{H_3}$), 6.17 (dt, *J* = 15.8, 6.5 Hz, 1H, C*H*=CHAr), 6.34 (d, *J* = 16.0 Hz, 1H, CH=C*H*Ar), 7.15–7.25 (m, 3H, ArC*H*), 7.32 (t, *J* = 1.8 Hz, 1H, ArC*H*) ppm; ^{13}C NMR (101 MHz, $CDCl_3$) δ 7.8 (C(O)CH_2*C*H_3), 26.8 (C*C*$H_2$$CH_2$C(O)), 27.7 ($CH_2$*C*$H_2$CH=CH), 33.1 (*C*$H_2$$CH_2$CH=CH), 36.0 (C(O)*C*$H_2$$CH_3$), 37.3 ($CH_2$*C*$H_2$C(O)), 52.5 ($CO_2$*C*$H_3$), 56.6 ($C_q$), 124.2 (Ar*C*H), 126.0 (Ar*C*H), 127.1 (Ar*C*H), 129.4 (CH=*C*HAr), 129.7 (Ar*C*H), 130.7 (*C*H=CHAr), 134.5 (Ar*C*Cl), 139.3 (Ar*C*), 171.7 (*C*$O_2$$CH_3$), 209.9 (*C*(O)) ppm; IR ν_{max} (thin film, cm^{-1}) = 3012, 1732 (C=O), 1435, 1254, 1197, 1117; HRMS calcd. for $C_{20}H_{25}O_5ClNa$ $[M+Na]^+$ 403.1283, found 403.1273.

Dimethyl (*E*)–2–(4–(naphthalen–1–yl)but–3–en–1–yl)–2–(3–oxopentyl)malonate (S26)

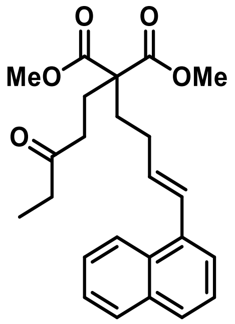

Prepared according to General Procedure A using dimethyl 2–(but–3–en–1–yl)–2–(3–oxopentyl)malonate **S21** (230 mg, 0.85 mmol, 1.0 eq.), 2–vinylnapthalene (394 mg, 2.6 mmol, 3.0 eq.) and Grubbs I catalyst (35 mg, 0.043 mmol, 5 mol%) in CH_2Cl_2 (3.5 mL). Purification by silica gel column chromatography (hexane/EtOAc 100:0 to 9:1) yielded the title product as a yellow solid (153 mg, 0.39 mmol, 45%). ^{1}H NMR (500 MHz, $CDCl_3$) δ 1.06 (t, *J* = 7.5 Hz, 3H, C(O)$CH_2C\mathit{H_3}$), 2.08–2.14 (m, 2H, C$\mathit{H_2}$$CH_2$CH=CH), 2.19–2.27 (m, 4H, C$\mathit{H_2}$$CH_2$C(O) + CH_2C$\mathit{H_2}$CH=CH), 2.42–2.47 (m, 4H, C(O)C$\mathit{H_2}$$CH_3$ + CH_2C$\mathit{H_2}$C(O)), 3.73 (s, 6H, CO_2C$\mathit{H_3}$), 6.29 (dt, *J* = 15.8, 6.6 Hz, 1H, C*H*=CHAr), 6.56 (d, *J* = 15.8 Hz, 1H, CH=C*H*Ar), 7.39–7.52 (m,

2H, ArC*H*), 7.56 (dd, *J* = 9.1, 1.6 Hz, 1H, ArC*H*), 7.67 (s, 1H, ArC*H*), 7.78 (dd, *J* = 10.6, 8.5 Hz, 3H, ArC*H*) ppm; ^{13}C NMR (126 MHz, $CDCl_3$) δ 7.8 ($C(O)CH_2\mathit{C}H_3$), 26.8 ($\mathit{C}H_2CH_2C(O)$), 27.9 ($CH_2\mathit{C}H_2CH{=}CH$), 33.2 ($\mathit{C}H_2CH_2CH{=}CH$), 36.0 ($C(O)\mathit{C}H_2CH_3$), 37.4 ($CH_2\mathit{C}H_2C(O)$), 52.5 ($CO_2\mathit{C}H_3$), 56.7 (C_q), 123.4 (Ar*C*H), 125.6 (Ar*C*H), 126.0 (Ar*C*H), 126.2 (Ar*C*H), 127.6 (Ar*C*H), 127.9 (Ar*C*H), 128.1 (Ar*C*H), 129.5 (*C*H=CHAr), 130.8 (CH=*C*HAr), 132.8 (Ar*C*), 133.7 (Ar*C*), 134.9 (Ar*C*), 171.8 ($\mathit{C}O_2CH_3$), 209.9 (*C*(O)) ppm; IR ν_{max} (thin film, cm^{-1}) = 3061, 2166, 1732 (C=O), 1274, 1090; HRMS calcd. for $C_{24}H_{28}O_5Na$ $[M+Na]^+$ 419.1829, found 419.1818.

Dimethyl (*E*)–2–(4–([1,1'–biphenyl]–4–yl)but–3–en–1–yl)–2–(3–oxopentyl)malonate (S27)

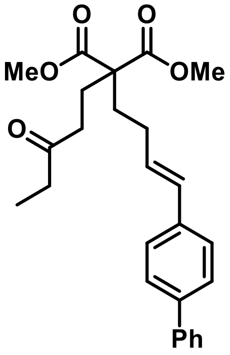

Prepared according to General Procedure A using dimethyl 2–(but–3–en–1–yl)–2–(3–oxopentyl)malonate **S21** (324 mg, 1.2 mmol, 1.0 eq.), 4–phenylstyrene (0.65 mL, 3.6 mmol, 3.0 eq.) and Grubbs I catalyst (49 mg, 0.06 mmol, 5 mol%) in CH_2Cl_2 (4.4 mL). Purification by silica gel column chromatography (Hexane/EtOAc 10:0 to 9:1) yielded the title product as a grey oil (171 mg, 0.41 mmol, 34%). ^{1}H NMR (400 MHz, CD_2Cl_2) δ 1.02 (t, 3H, *J* = 7.3 Hz, $CH_2C\mathit{H}_3$), 2.02–2.08 (m, 2H, $C\mathit{H}_2CH_2CH{=}CH$), 2.10–2.20 (m, 4H, *J* = 14.7, 8.7, 5.9 Hz, $CH_2C\mathit{H}_2CH{=}CHAr$ + $CC\mathit{H}_2CH_2C(O)$), 2.37–2.45 (m, 4H, $CCH_2C\mathit{H}_2C(O)$ + $C(O)C\mathit{H}_2CH_3$), 3.70 (s, 6H, $CO_2C\mathit{H}_3$), 6.23 (dt, 1H, *J* = 15.9, 6.6 Hz, C*H*=CHAr), 6.44 (d, 1H, *J* = 15.9 Hz, CH=C*H*Ar), 7.33 (t, 1H, *J* = 7.1, ArC*H*), 7.40–7.46 (m, 4H, ArC*H*), 7.55 (d, 2H, *J* = 8.3 Hz, ArC*H*), 7.60 (d, 2H, *J* = 7.3 Hz, ArC*H*) ppm; ^{13}C NMR (126 MHz, CD_2Cl_2) δ 7.5 ($CH_2C\mathit{H}_3$), 26.6 ($C\mathit{C}H_2CH_2C(O)$), 27.7 ($CH_2\mathit{C}H_2CH{=}CH$), 32.8 ($\mathit{C}H_2CH_2CH{=}CH$), 35.8 ($C(O)C\mathit{H}_2CH_3$), 37.1 ($CCH_2\mathit{C}H_2C(O)$), 52.3 ($O\mathit{C}H_3$), 56.6 (C_q), 126.3 (Ar*C*H), 126.7 (Ar*C*H) 127.1 (Ar*C*H), 127.2 (Ar*C*H), 128.7 (Ar*C*H), 129.5 ($CH_2CH_2CH{=}\mathit{C}HAr$), 129.9 ($CH_2CH_2\mathit{C}H{=}CHAr$), 136.6 (Ar*C*), 139.6 (Ar*C*), 140.6 (Ar*C*), 171.6 ($\mathit{C}O_2CH_3$), 210.3 ($\mathit{C}(O)CH_2CH_3$) ppm; IR ν_{max} (thin film, cm^{-1}) = 3737, 3727, 3704, 2920, 2850, 2362, 1732, 1448, 1258, 1105, 797, 758, 683; HRMS calculated for $C_{26}H_{30}O_5Na$ $[M+Na]^+$ 445.1985, found 445.1972.

Dimethyl (*E*)–2–(4–(4–fluorophenyl)but–3–en–1–yl)–2–(3–oxopentyl)malonate (S28)

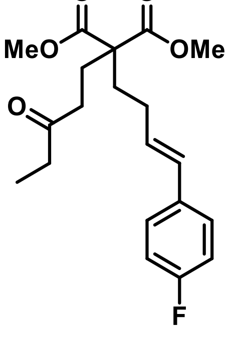

Prepared according to General Procedure A using dimethyl 2–(but–3–en–1–yl)–2–(3–oxopentyl)malonate **S21** (300 mg, 1.1 mmol, 1.0 eq.), 4–fluorostyrene (0.39 mL, 3.3 mmol, 3.0 eq.) and Grubbs I catalyst (45 mg, 0.055 mmol, 5 mol%) in CH_2Cl_2 (4 mL). Purification by silica gel column chromatography (Hexane/EtOAc 10:0 to 9:1) yielded the title product as a grey oil (171 mg, 0.18 mmol, 16%). ^{1}H NMR (400 MHz, $CDCl_3$) δ 1.05 (td, 3H, *J* = 7.4, 0.9 Hz, $CH_2C\mathit{H}_3$), 2.04 (dt, 2H, *J* = 11.1, 3.5 Hz, $C\mathit{H}_2CH_2CH$=CH), 2.12 (td, 2H, *J* = 8.1, 7.5, 2.5, $CH_2C\mathit{H}_2CH$=CH), 2.21 (dd, 2H, *J* = 8.8, 6.8 Hz, $CC\mathit{H}_2CH_2C$(O)), 2.38–2.46 (m, 4H, $CCH_2C\mathit{H}_2C$(O) + C(O)$C\mathit{H}_2CH_3$), 3.72 (s, 6H, $CO_2C\mathit{H}_3$), 6.05 (dt, 1H, *J* = 15.8, 6.5 Hz, C*H*=CHAr), 6.35 (d, *J* = 15.8 Hz, 1H, CH=C*H*Ar), 6.97 (ddd, 2H, *J* = 8.7, 7.7, 0.9 Hz, ArC*H*), 7.27–7.31 (m, 2H, ArC*H*) ppm; ^{13}C NMR (126 MHz, $CDCl_3$) δ 7.8 ($\mathit{C}H_3CH_2C$(O)), 26.8 ($C\mathit{C}H_2CH_2C$(O)), 27.7 ($CH_2\mathit{C}H_2CH$=CH), 33.2 ($\mathit{C}H_2CH_2CH$=CH), 36.0 ($CH_3\mathit{C}H_2C$(O)), 37.4 ($CCH_2\mathit{C}H_2C$(O)), 52.5 ($CO_2\mathit{C}H_3$), 56.6 (C_q), 115.4 (d, 2H, *J* = 21.5 Hz, Ar*C*H), 127.4 (d, 2H, *J* = 7.9 Hz, Ar*C*H), 128.7 (*C*H=CHAr), 129.5 (CH=*C*HAr), 133.6 (d, *J* = 3.4 Hz, Ar*C*), 162.0 (d, *J* = 245.9, Ar*C*F), 171.6 ($CO_2\mathit{C}H_3$), 209.9 (C(O)CH_2CH_3) ppm; ^{19}F NMR (471 MHz, $CDCl_3$) δ –115.4 ppm; IR $v_{\max}$ (thin film, cm^{-1}) = 3727, 363, 2952, 2358, 1731, 1601, 1508, 1458, 1225, 1093, 967, 849, 668; HRMS calculated for $C_{20}H_{25}FO_5Na$ $[M+Na]^+$ 387.1578, found 387.1558.

Dimethyl (*E*)–2–(4–(4–bromophenyl)but–3–en–1–yl)–2–(3–oxopentyl)malonate (S29)

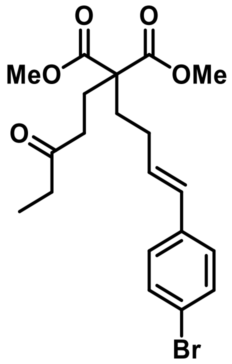

Prepared according to General Procedure A using dimethyl 2–(but–3–en–1–yl)–2–(3–oxopentyl)malonate **S21** (300 mg, 1.1 mmol, 1.0 eq.), 4–bromostyrene (0.43 mL, 3.3 mmol, 3.0 eq.) and Grubbs I catalyst (45 mg, 0.055 mmol, 5 mol%) in CH_2Cl_2 (4 mL). Purification by silica gel column chromatography (hexane/EtOAc 100:0 to 9:1) yielded the title product as a colourless oil (147 mg, 0.35 mmol, 31%). ^{1}H NMR (500 MHz, $CDCl_3$) δ 1.06 (t, *J* = 7.4 Hz, 3H, C(O)$CH_2C\mathit{H}_3$), 2.03–2.08 (m, 2H, $C\mathit{H}_2CH_2CH$=CH), 2.13 (dd, *J* = 9.8, 5.9 Hz, 2H, $CH_2C\mathit{H}_2CH$=CH), 2.22 (dd, *J* = 8.8, 6.8 Hz, 2H, $C\mathit{H}_2CH_2C$(O)), 2.40–2.46 (m, 4H, C(O)$C\mathit{H}_2CH_3$ + $CH_2C\mathit{H}_2C$(O)), 3.72 (s, 6H, $CO_2C\mathit{H}_3$), 6.14 (dt, *J* = 15.9, 6.5 Hz, 1H, C*H*=CHAr), 6.33 (d, *J* = 15.9 Hz, 1H, CH=C*H*Ar), 7.19 (d, *J* = 8.2 Hz, 2H, ArC*H*), 7.41 (d, *J* = 8.2 Hz, 2H, ArC*H*) ppm; ^{13}C NMR (126 MHz, $CDCl_3$) δ 7.8 (C(O)$CH_2\mathit{C}H_3$), 26.8 ($\mathit{C}H_2CH_2C$(O)), 27.7 ($CH_2\mathit{C}H_2CH$=CH), 33.1 ($\mathit{C}H_2CH_2CH$=CH), 36.0 (C(O)$\mathit{C}H_2CH_3$), 37.3 ($CH_2\mathit{C}H_2C$(O)), 52.5 ($CO_2\mathit{C}H_3$), 56.6 (C_q), 120.8 (Ar*C*Br), 127.5 (Ar*C*H), 129.5 (CH=*C*HAr), 129.9 (*C*H=CHAr), 131.6 (Ar*C*H), 136.4 (Ar*C*), 171.7 ($\mathit{C}O_2CH_3$), 209.9 (*C*(O)) ppm; IR v_{max} (thin film, cm^{-1}) = 2940, 2043, 1729 (C=O), 1435, 1265, 1194, 1130; HRMS calcd. for $C_{20}H_{25}O_5BrNa$ $[M+Na]^+$ 447.0778, found 477.0765.

Dimethyl (*E*)–2–(3–oxopentyl)–2–(4–(4–(trifluoromethyl)phenyl)but–3–en–1–yl)malonate (S30)

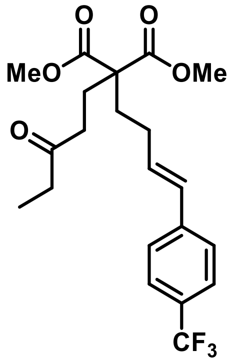

Prepared according to General Procedure A using dimethyl 2–(but–3–en–1–yl)–2–(3–oxopentyl)malonate **S21** (300 mg, 1.1 mmol, 1.0 eq.), 4–trifluoromethylstyrene (0.49 mL, 3.3 mmol, 3.0 eq.) and Grubbs I catalyst (45 mg, 0.055 mmol, 5 mol%) in CH_2Cl_2 (4 mL). Purification by silica gel column chromatography (Hexane/EtOAc 10:0 to 9:1) yielded the title product as a dark oil (172 mg, 0.41 mmol, 38%). ^{1}H NMR (400 MHz, $CDCl_3$) δ 1.05 (t, 3H, *J* = 7.3 Hz, CH_2CH_3), 2.03–2.09 (m, 2H, CH_2CH_2CH=CH), 2.14–2.19 (m, 2H, CH_2CH_2CH=CH), 2.19–2.25 (m, 2H, CCH_2CH_2C(O)) 2.39–2.47 (m, 4H, CCH_2CH_2C(O) + CH_2CH_3), 3.72 (s, 6H, CO_2CH_3), 6.25 (dt, 1H, *J* = 15.8, 6.5 Hz, C*H*=CHAr), 6.42 (d, 1H, *J* = 16.0 Hz, CH=C*H*Ar), 7.41 (d, 2H, *J* = 8.1 Hz, ArC*H*), 7.54 (d, 2H, *J* = 8.2 Hz, ArC*H*) ppm; ^{13}C NMR (126 MHz, $CDCl_3$) δ 7.8 (C(O)CH_2CH_3), 26.8 (CCH_2CH_2C(O)), 27.8 (CH_2CH_2CH=CH), 33.0 (CH_2CH_2CH=CH), 36.0 (C(O)CH_2CH_3), 37.3 (CCH_2CH_2C(O)), 52.6 (CO_2CH_3), 56.6 (C_q), 125.5 (d, *J* = 3.8 Hz, 2 × ArC*H*), 126.1 (2 × ArC*H*), 128.9 (q, *J* = 32.5 Hz, Ar*C*CF_3), 129.5 (CH=*C*HAr), 131.9 (*C*H=CHAr), 140.9 (Ar*C*), 171.6 (*C*O_2CH_3), 209.9 (*C*(O)) ppm, *C*F_3 is not observed; ^{19}F NMR (471 MHz, $CDCl_3$) δ –62.5 ppm; IR ν_{max} (thin film, cm^{-1}) = 2954, 1731, 1614, 1435, 1325, 1164, 1120, 1067, 1016, 969, 860, 815, 652; HRMS calculated for $C_{21}H_{25}F_3O_5Na$ [M+Na]$^+$ 437.1546, found 437.1524.

Dimethyl (*E*)–2–(4–(4–methoxyphenyl)but–3–en–1–yl)–2–(3–oxopentyl)malonate (S31)

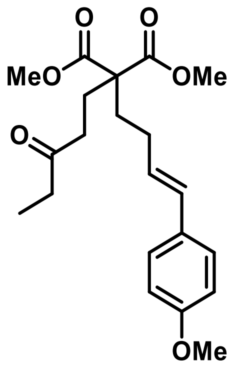

Prepared according to General Procedure A using dimethyl 2–(but–3–en–1–yl)–2–(3–oxopentyl)malonate **S21** (250 mg, 0.92 mmol, 1.0 eq.), 4–methoxystyrene (0.39 mL, 3.0 mmol, 3.0 eq.) and Grubbs I catalyst (38 mg, 0.046 mmol, 5 mol%) in CH_2Cl_2 (4 mL). Purification by silica gel column chromatography (hexane/EtOAc 100:0 to 9:1) yielded the title product as a brown oil (171 mg, 0.45 mmol, 49%). ^{1}H NMR (400 MHz, $CDCl_3$) δ 1.06 (t, *J* = 7.4 Hz, 3H, C(O)CH_2CH_3), 2.00–2.07 (m, 2H, CH_2CH_2CH=CH), 2.09–2.15 (m, 2H, CH_2CH_2CH=CH), 2.18–2.26 (m, 2H, CH_2CH_2C(O)), 2.38–2.46 (m, 4H, C(O)CH_2CH_3 + CH_2CH_2C(O)), 3.72 (s, 3H, CO_2CH_3), 3.81 (s, 3H, ArOCH_3), 6.00 (dt, *J* = 15.8, 6.5 Hz, 1H, C*H*=CHAr), 6.34 (d, *J* = 15.8 Hz, 1H, CH=C*H*Ar), 6.84 (d, *J* = 8.7 Hz, 2H, ArC*H*), 7.26 (d, *J* = 8.7 Hz, 2H, ArC*H*) ppm; ^{13}C NMR (101 MHz, $CDCl_3$) δ 7.8 (C(O)CH_2CH_3), 26.8 (CH_2CH_2C(O)), 27.7 (CH_2CH_2CH=CH), 33.3 (CH_2CH_2CH=CH), 36.0 (C(O)CH_2CH_3), 37.4 (CH_2CH_2C(O)), 52.5 (CO_2CH_3), 55.3 (ArOCH_3), 56.7 (C_q),

114.0 (Ar*C*H), 126.8 (*C*H=CHAr), 127.1 (Ar*C*H), 130.0 (CH=*C*HAr), 171.8 (*C*O_2CH_3), 210.0 (*C*(O)) ppm; IR ν_{max} (thin film, cm^{-1}) = 2951, 1729 (C=O), 1607, 1511, 1246, 1176, 1032; HRMS calcd. for $C_{21}H_{28}O_6Na$ $[M+Na]^+$ 399.1778, found 399.1764.

***Rac*–methyl (3*R*,6*S*)–6–methyl–2–oxo–3–((*E*)–4–phenylbut–3–en–1–yl)tetrahydro–2*H*–pyran–3–carboxylate (1a)**

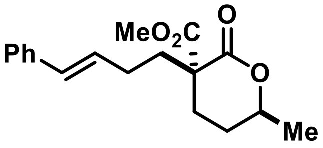

Prepared according to general procedue D using of dimethyl (*E*)–2–(3–oxobutyl)–2–(4–phenylbut–3–en–1–yl)malonate **S3** (1.0 g, 3.0 mmol, 1.0 eq.), SmI_2 (76 mL, 0.1 M in THF, 7.6 mmol, 2.5 eq.) and H_2O (14 mL, 760 mmol, 250 eq.) in THF, followed by *p*TSA (30 mg, 0.15 mmol, 5 mol%) in CH_2Cl_2 (35 mL). Purification by silica gel column chromatography (hexane/EtOAc 95:5) yielded the title product as a 3:1 mixture of diastereoisomers (606 mg, 2.0 mmol, 66%). The major diastereoisomer could be isolated and was obtained as a colourless oil. ^{1}H NMR (500 MHz, $CDCl_3$) δ 1.38 (d, *J* = 6.2 Hz, 3H, CO_2CHC*H_3*), 1.65 (ddt, *J* = 14.1, 10.5, 8.0 Hz, 1H, CO_2CHC*H_a*H_b), 1.84 (ddd, *J* = 13.7, 8.3, 5.0 Hz, 1H, C(O)CC*H_a*H_b), 1.98–2.06 (m, 2H, CO_2CHCH_a*H_b* + C*H_a*H_bCH_2CH=CHAr), 2.16 (ddd, *J* = 13.5, 11.3, 4.7 Hz, 1H, CH_a*H_b*CH_2CH=CHAr), 2.19–2.27 (m, 1H, CH_2C*H_a*H_bCH=CHAr), 2.27–2.36 (m, 1H, CH_2CH_a*H_b*CH=CHAr), 2.56 (dt, *J* = 14.0, 8.0 Hz, 1H, C(O)CCH_a*H_b*), 3.79 (s, 3H, CO_2C*H_3*), 4.36 (dqd, *J* = 10.0, 6.1, 3.7 Hz, 1H, CO_2C*H*), 6.18 (dt, *J* = 15.8, 6.6 Hz, 1H, C*H*=CHAr), 6.42 (d, *J* = 15.9 Hz, 1H, CH=C*H*Ar), 7.18–7.23 (m, 1H, ArC*H*), 7.28–7.35 (m, 4H, ArC*H*) ppm; ^{13}C NMR (126 MHz, $CDCl_3$) δ 21.4 (CO_2CH*C*H_3), 27.0 (CO_2CH*C*H_2), 27.8 (C(O)C*C*H_2), 28.0 (*C*H_2CH_2CH=CHAr), 36.1 (*C*H_2CH=CHAr), 52.8 (CO_2*C*H_3), 53.1 (*C_q*), 74.8 (CO_2*C*H), 126.0 (Ar*C*H), 127.1 (Ar*C*H), 128.5 (Ar*C*H), 129.1 (*C*H=CHAr), 130.7 (CH=*C*HAr), 137.5 (Ar*C*), 170.8 (*C*O_2CH), 171.8 (*C*O_2CH_3) ppm; IR ν_{max} (thin film, cm^{-1}) = 2207, 1732 (C=O), 1434, 1260, 1065, 985; HRMS calcd. for $C_{18}H_{22}O_4Na$ $[M+Na]^+$ 325.1410, found 325.1397.

***Rac*–methyl (3*R*,6*S*)–6–ethyl–2–oxo–3–((*E*)–4–phenylbut–3–en–1–yl)tetrahydro–2*H*–pyran–3–carboxylate (1b)**

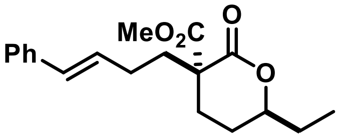

Prepared according to General Procedure D using dimethyl (*E*)–2–(3–oxopentyl)–2–(4–phenylbut–3–en–1–yl)malonate **S4** (200 mg, 0.58 mmol, 1.0 eq.), SmI_2 (14 mL, 0.1 M in THF, 1.4 mmol, 2.5 eq.), and H_2O (2.60 mL, 144 mmol, 250 eq.) in THF, followed by *p*TSA (5.5 mg, 0.029 mmol, 5 mol%) in CH_2Cl_2 (5 mL). Purification by silica gel column chromatography (hexane/EtOAc 9:1) yielded the title product as a white solid, as a 7.3:1 mixture of diastereoisomers (107 mg, 0.34 mmol, 58%). ^{1}H NMR (400 MHz, $CDCl_3$) δ 1.00 (t, *J* = 7.5 Hz, 3H, CH_2C*H_3*), 1.58–1.88 (m, 4H, C*H_2*CH_3 + CO_2CHC*H_a*H_b + C(O)CC*H_a*H_b), 1.95–2.07 (m, 2H, CO_2CHCH_a*H_b* + C*H_a*H_bCH=CHAr), 2.10–2.37 (m, 3H, CH_a*H_b*CH=CHAr + C*H_2*CH_2CH=CHAr), 2.55 (dt, *J* = 13.9, 8.0 Hz, C(O)CCH_a*H_b*), 3.79 (s, 3H, CO_2C*H_3*), 4.12 (dddd, *J* = 10.7, 6.8, 5.5, 3.9 Hz, 0.89H, CO_2C*H* from major diastereoisomer), 4.23–4.30 (m, 0.11H, CO_2C*H* from minor diastereoisomer), 6.18 (dt, *J* = 15.8, 6.6 Hz, 1H, C*H*=CHAr), 6.42 (d, *J* = 16.0 Hz, 1H, CH=C*H*Ar), 7.18–7.24 (m, 1H, ArC*H*), 7.28–

7.38 (m, 4H, ArC*H*) ppm; ^{13}C NMR (101 MHz, $CDCl_3$) δ 9.4 (CH_2*C*H_3), 25.5 (CO_2CH*C*H_2), 27.1 (C(O)C*C*H_2), 28.1 (*C*H_2CH_2CH=CHAr), 28.6 (*C*H_2CH_3), 36.0 (*C*H_2CH=CHAr), 53.0 (*C_q*), 53.1 (CO_2*C*H_3), 79.8 (CO_2*C*H), 126.0 (Ar*C*H), 127.1 (Ar*C*H), 128.5 (Ar*C*H), 129.1 (*C*H=CHAr), 130.7 (CH=*C*HAr), 137.5 (Ar*C*), 170.9 (*C*O_2CH), 171.8 (*C*O_2CH_3) ppm; IR v_{max} (thin film, cm^{-1}) = 2956, 2170, 1728 (C=O), 1446, 1265, 1124; HRMS calcd. for $C_{19}H_{24}O_4Na$ [M+Na]$^+$ 339.1567, found 339.1557.

Rac–methyl (3_R_,6_S_)–6–butyl–2–oxo–3–((_E_)–4–phenylbut–3–en–1–yl)tetrahydro–2_H_–pyran–3–carboxylate (1c)

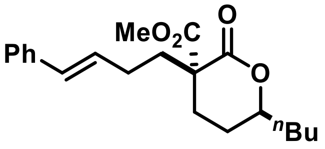

Prepared according to General Procedure D using dimethyl (*E*)–2–(3–oxoheptyl)–2–(4–phenylbut–3–en–1–yl)malonate **S6** (202 mg, 0.54 mmol, 1.0 eq.), SmI_2 (13.5 mL, 0.1 M in THF, 1.35 mmol, 2.5 eq.) and H_2O (2.4 mL, 135 mmol, 250 eq.) in THF, followed by *p*TSA (4.6 mg, 0.024 mmol, 5 mol%) in CH_2Cl_2 (4 mL). Purification by silica gel column chromatography (Hexane/EtOAc 9:1) yielded the title product as a white solid, as a 4.5:1 mixture of diastereoisomers (119 mg, 0.34 mmol, 72%). M.p. 45–46 °C. ^{1}H NMR (500 MHz, $CDCl_3$) δ 0.87–0.90 (m, 3H, C*H_3*(CH_2)$_2CH_2$CH), 1.33 (dtd, 4H, *J* = 13.8, 7.3, 3.7 Hz, CH_3(C*H_2*)$_2CH_2$CH), 1.41–1.50 (m, 1H, C(O)CC*H_a*H_b), 1.64–1.75 (m, 2H, $CH_3(CH_2)_2$C*H_2*CH), 1.81 (ddd, 1H, *J* = 13.7, 8.2, 4.9 Hz, CO_2CHC*H_a*H_b), 1.95–2.05 (m, 2H, C*H_2*CH_2CH=CHAr), 2.10–2.18 (m, 1H, CO_2CHCH_a*H_b*), 2.23 (dddd, 1H, *J* = 8.2, 6.1, 4.1, 1.9 Hz, CH_2C*H_a*H_bCH=CHAr), 2.26–2.36 (m, 1H, CH_2CH_a*H_b*CH=CHAr), 2.54 (dt, 1H, *J* = 13.9, 8.0 Hz, C(O)CCH_a*H_b*), 3.77 (s, 3H, OC*H_3*), 4.16 (dddd, 0.86H, *J* = 11.1, 7.5, 5.0, 3.9 Hz, CO_2C*H* from major diastereomer), 4.26–4.34 (m, 0.14H, CO_2C*H*, from minor diastereomer), 6.17 (dt, 1H, *J* = 15.8, 6.6 Hz, C*H*=CHAr), 6.41 (d, 1H, *J* = 15.9 Hz, CH=C*H*Ar), 7.17–7.22 (m, 1H, ArC*H*), 7.28–7.34 (m, 4H, ArC*H*) ppm; ^{13}C NMR (126 MHz, $CDCl_3$) δ 13.9 (*C*$H_3(CH_2)_2CH_2$CH), 22.4 (CH_3(*C*H_2)$_2CH_2$CH), 26.0 (C(O)CH*C*H_2), 27.1 (CH_3(*C*H_2)$_2CH_2$CH), 27.1 (C(O)C*C*H_2) 28.1 (*C*H_2CH_2CH=CHAr), 35.3 ($CH_3(CH_2)_2$*C*H_2CH), 36.0 (CH_2*C*H_2CH=CHAr), 53.0 (*C*$_q$), 53.1 (CO_2*C*H_3), 78.5 (CO_2*C*H from major diastereomer), 82.2 (CO_2*C*H from minor diastereomer), 126.0 (Ar*C*H), 126.0 (Ar*C*H), 127.1 (Ar*C*H), 127.1 (Ar*C*H), 128.5 (Ar*C*H), 129.1 (*C*H=CHAr), 130.7 (CH=*C*HAr), 130.7 (Ar*C*), 170.1 (*C*O_2CH_3), 171.8 (*C*O_2CH) ppm; IR v_{max} (thin film, cm^{-1}) = 2954, 2863, 1728, 1496, 1447, 1236, 1176, 1093, 965, 744, 694; HRMS calculated for $C_{21}H_{28}O_4Na$ [M+Na]$^+$ 367.1880, found 367.1873.

Rac–methyl (3_R_,6_R_)–6–benzyl–2–oxo–3–((_E_)–4–phenylbut–3–en–1–yl)tetrahydro–2_H_–pyran–3–carboxylate (1d)

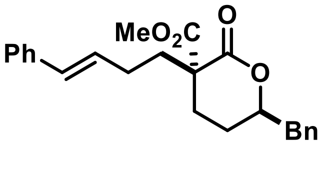

Prepared according to General Procedure D using dimethyl (*E*)–2–(3–oxo–4–phenylbutyl)–2–(4–phenylbut–3–en–1–yl)malonate **S8** (200 mg, 0.49 mmol, 1.0 eq.), SmI_2 (12 mL, 0.1 M in THF, 1.2 mmol, 2.5 eq.), and H_2O (2.2 mL, 122 mmol, 250 eq.) in THF, followed by *p*TSA (4.7 mg, 0.025 mmol, 5 mol%) in CH_2Cl_2 (5 mL). Purification by silica gel column chromatography (hexane/EtOAc 9:1) yielded the title product as a colourless oil, as a 7.3:1 mixture of diastereoisomers (115 mg, 0.30 mmol, 62%). ^{1}H NMR (400 MHz,

$CDCl_3$) δ 1.68 (ddt, *J* = 13.9, 10.5, 8.0 Hz, 1H, $CO_2CHC\mathit{H_a}H_b$), 1.82 (ddd, *J* = 13.9, 7.8, 4.9 Hz, 1H, $C(O)CC\mathit{H_a}H_b$), 1.90–2.03 (m, 2H, $CO_2CHCH_a\mathit{H_b}$ + $C\mathit{H_a}H_bCH_2CH{=}CHAr$), 2.05–2.34 (m, 3H, $CH_a\mathit{H_b}CH_2CH{=}CHAr$ + $CH_2C\mathit{H_2}CH{=}CHAr$), 2.48 (dt, *J* = 13.9, 7.9 Hz, 1H, $C(O)CCH_a\mathit{H_b}$), 2.88 (dd, *J* = 14.0, 6.8 Hz, 1H, $C\mathit{H_a}H_bAr$), 3.09 (dd, *J* = 13.9, 5.7 Hz, 1H, $CH_a\mathit{H_b}Ar$), 3.76 (s, 3H, $CO_2C\mathit{H_3}$), 4.45 (dddd, *J* = 10.5, 6.7, 5.8, 3.8 Hz, 0.88H, $CO_2C\mathit{H}$ from major diastereoisomer), 4.50–4.57 (m, 0.12H, $CO_2C\mathit{H}$ from minor diastereoisomer), 6.16 (dt, *J* = 15.8, 6.6 Hz, 1H, $C\mathit{H}{=}CHAr$), 6.40 (d, *J* = 15.8 Hz, 1H, $CH{=}C\mathit{H}Ar$), 7.18–7.26 (m, 4H, ArC*H*), 7.29–7.36 (m, 6H, ArC*H*) ppm; ^{13}C NMR (101 MHz, $CDCl_3$) δ 25.2 ($CO_2CH\mathit{C}H_2$), 27.1 ($C(O)\mathit{C}CH_2$), 28.0 ($CH_2\mathit{C}H_2CH{=}CHAr$), 36.0 ($\mathit{C}H_2CH_2CH{=}CHAr$), 41.7 ($\mathit{C}H_2Ar$), 53.0 ($C_q$), 53.1 ($CO_2\mathit{C}H_3$), 79.1 ($CO_2\mathit{C}H$), 126.0 (Ar*C*H), 127.0 (Ar*C*H), 127.1 (Ar*C*H), 128.5 (Ar*C*H), 129.1 (Ar*C*H), 129.5 (*C*H=CHAr), (Ar*C*H), 130.7 (CH=*C*HAr), 136.1 (Ar*C*), 137.5 (Ar*C*), 170.4 ($\mathit{C}O_2CH$), 171.7 ($\mathit{C}O_2CH_3$) ppm; IR ν_{max} (thin film, cm^{-1}) = 2950, 1732 (C=O), 1455, 1365, 1163, 1103; HRMS calcd. for $C_{24}H_{26}O_4Na$ $[M+Na]^+$ 401.1723, found 401.1709.

***Rac*–methyl (3*R*,6*R*)–6–neopentyl–2–oxo–3–((*E*)–4–phenylbut–3–en–1–yl)tetrahydro–2*H*–pyran–3–carboxylate (1e)**

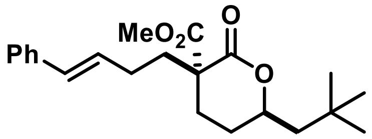

Prepared according to General Procedure D using dimethyl (*E*)–2–(5,5–dimethyl–3–oxohexyl)–2–(4–phenylbut–3–en–1–yl)malonate **S10** (150 mg, 0.39 mmol, 1.0 eq.), SmI_2 (9.7 mL, 0.1 M in THF, 0.97 mmol, 2.5 eq.), and H_2O (1.7 mL, 97 mmol, 250 eq.) in THF, followed by *p*TSA (3.7 mg, 0.02 mmol, 5 mol%) in CH_2Cl_2 (4 mL). Purification by silica gel column chromatography (hexane/EtOAc 9:1) yielded the title product as a colourless oil, as a 8.6:1 mixture of diastereoisomers (113 mg, 0.32 mmol, 81%). ^1H NMR (400 MHz, $CDCl_3$) δ 0.96 (s, 9H, $C(C\mathit{H_3})_3$), 1.36 (dd, *J* = 14.7, 2.7 Hz, 1H, $C\mathit{H_a}H_bC(CH_3)_3$), 1.62–1.82 (m, 3H, $CH_a\mathit{H_b}C(CH_3)_3$ + $C(O)CC\mathit{H_a}H_b$ + $CO_2CHC\mathit{H_a}H_b$), 1.89–2.03 (m, 2H, $CO_2CHCH_a\mathit{H_b}$ + $C\mathit{H_a}H_bCH_2CH{=}CHAr$), 2.08–2.26 (m, 2H, $CH_a\mathit{H_b}CH_2CH{=}CHAr$ + $CH_2C\mathit{H_a}H_bCH{=}CHAr$), 2.29–2.43 (m, 1H, $CH_2CH_a\mathit{H_b}CH{=}CHAr$), 2.59 (ddd, *J* = 13.4, 8.7, 6.8 Hz, 1H, $C(O)CCH_a\mathit{H_b}$), 3.80 (s, 3H, $CO_2C\mathit{H_3}$), 4.27 (dddd, *J* = 10.6, 7.9, 3.7, 2.7 Hz, 0.9H, $CO_2C\mathit{H}$ from major diastereoisomer), 4.42 (ddt, *J* = 11.1, 7.2, 3.5 Hz, 0.1H, $CO_2C\mathit{H}$ from minor diastereoisomer), 6.18 (dt, *J* = 15.8, 6.5 Hz, 1H, $C\mathit{H}{=}CHAr$), 6.42 (d, *J* = 15.9 Hz, 1H, $CH{=}C\mathit{H}Ar$), 7.18–7.23 (m, 1H, ArC*H*), 7.28–7.35 (m, 4H, ArC*H*) ppm; ^{13}C NMR (101 MHz, $CDCl_3$) δ 27.0 ($C(O)C\mathit{C}H_2$), 27.7 ($CO_2CH\mathit{C}H_2$), 28.1 ($CH_2\mathit{C}H_2CH{=}CHAr$), 29.9 ($C(\mathit{C}H_3)_3$), 30.1 ($\mathit{C}(CH_3)_3$), 36.0 ($\mathit{C}H_2CH_2CH{=}CHAr$), 48.9 ($\mathit{C}H_2C(CH_3)_3$), 52.7 ($CO_2\mathit{C}H_3$) 53.0 ($C_q$), 75.8 ($CO_2\mathit{C}H$), 126.0 (Ar*C*H), 127.1 (Ar*C*H), 128.5 (Ar*C*H), 129.2 (*C*H=CHAr), 130.7 (CH=*C*HAr), 137.5 (Ar*C*), 170.9 ($\mathit{C}O_2CH$), 171.9 ($\mathit{C}O_2CH_3$) ppm; IR ν_{max} (thin film, cm^{-1}) = 29116, 2184, 1728 (C=O), 1460, 1140, 1086, 1039; HRMS calcd. for $C_{22}H_{30}O_4Na$ $[M+Na]^+$ 381.2036, found 381.2023.

***Rac*–methyl (3*R*,6*R*)–6–isopropyl–2–oxo–3–((*E*)–4–phenylbut–3–en–1–yl)tetrahydro–2*H*–pyran–3–carboxylate (1f)**

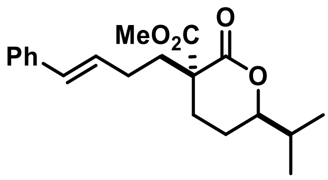

Prepared according to General Procedure D using dimethyl (*E*)–2–(4–methyl–3–oxopentyl)–2–(4–phenylbut–3–en–1–yl)malonate **S12** (200 mg, 0.55 mmol, 1.0 eq.), SmI_2 (134 mL, 0.1 M in THF, 1.4 mmol, 2.5 eq.), and H_2O (2.5 mL, 139 mmol, 250 eq.) in THF, followed by *p*TSA (5.2 mg, 0.028 mmol, 5 mol%) in CH_2Cl_2 (5 mL). Purification by silica gel column chromatography (hexane/EtOAc 9:1) yielded the title product as a white solid, as a 5.3:1 mixture of diastereoisomers (130 mg, 0.39 mmol, 72%). ^{1}H NMR (400 MHz, $CDCl_3$) δ 0.96 (d, *J* = 6.8 Hz, 3H, CH(C*H₃*)CH₃), 1.01 (d, *J* = 6.7 Hz, 3H, CH(CH₃)C*H₃*), 1.69 (ddt, *J* = 13.7, 11.0, 8.1 Hz, 1H, $CO_2CHCH_aH_b$ with *H$_a$*), 1.79–1.91 (m, 2H, C*H*(CH₃)₂ + C(O)CC*H$_a$*H$_b$), 1.92–2.06 (m, 2H, CO_2CHCH_a*H$_b$* + C*H$_a$*H$_b$CH₂CH=CHAr), 2.15 (td, *J* = 12.3, 11.6, 4.6 Hz, 1H, CH$_a$*H$_b$*CH₂CH=CHAr), 2.23 (dtd, *J* = 11.5, 6.5, 3.1 Hz, 1H, CH₂C*H$_a$*H$_b$CH=CHAr), 2.27–2.35 (m, 1H, CH₂CH$_a$*H$_b$*CH=CHAr), 2.54 (dt, *J* = 13.7, 8.0 Hz, 1H, C(O)CCH$_a$*H$_b$*), 3.78 (s, 3H, CO₂C*H₃*), 3.92 (ddd, *J* = 10.7, 6.1, 3.9 Hz, 0.84H, CO₂C*H* from major diastereoisomer), 4.10–4.16 (m, 0.16H, CO₂C*H* from minor diastereoisomer), 6.18 (dt, *J* = 15.7, 6.6 Hz, 1H, C*H*=CHAr), 6.42 (d, *J* = 15.8 Hz, 1H, CH=C*H*Ar), 7.17–7.23 (m, 1H, ArC*H*), 7.28–7.34 (m, 4H, ArC*H*) ppm; ^{13}C NMR (126 MHz, $CDCl_3$) δ 17.8 (CH(*C*H₃)CH₃), 17.9 (CH(CH₃)*C*H₃), 22.9 (CO₂CH*C*H₂), 27.2 (C(O)C*C*H₂), 28.1 (*C*H₂CH₂CH=CHAr), 32.6 (*C*H(CH₃)₂), 36.0 (*C*H₂CH=CHAr), 53.0 (CO₂*C*H₃), 53.1 (*C$_q$*), 83.1 (CO₂*C*H), 126.0 (Ar*C*H), 127.1 (Ar*C*H), 128.5 (Ar*C*H), 129.2 (*C*H=CHAr), 130.7 (CH=*C*HAr), 137.5 (Ar*C*), 171.0 (*C*O₂CH), 171.8 (*C*O₂CH₃) ppm; IR ν_{max} (thin film, cm^{-1}) = 2963, 1727 (C=O), 1457, 1247, 1192, 908, 731; HRMS calcd. for $C_{20}H_{26}O_4Na$ [M+Na]$^+$ 353.1723, found 353.1711.

Rac–methyl (3*R*,6*R*)–6–benzhydryl–2–oxo–3–((*E*)–4–phenylbut–3–en–1–yl)tetrahydro–2*H*–pyran–3–carboxylate (1g)

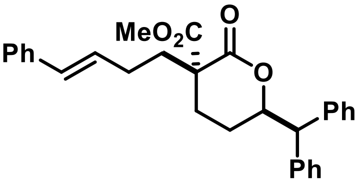

Prepared according to General Procedure D using dimethyl (*E*)–2–(3–oxo–4,4–diphenylbutyl)–2–(4–phenylbut–3–en–1–yl)malonate **S14** (198 mg, 0.41 mmol, 1.0 eq.), SmI_2 (10.3 mL, 0.1 M in THF, 1.03 mmol, 2.5 eq.), and H_2O (103 mL, 103 mmol, 250 eq.) in THF, followed by treatment with *p*TSA (3.6 mg, 0.019 mmol, 5 mol%) in CH_2Cl_2 (1.9 mL) to give the crude product as 2.6:1 mixture of diastereoisomers. Purification by silica gel column chromatography (Hexane/EtOAc 9:1) yielded the title product as a colourless oil (102 mg, 0.22 mmol, 59%). ^{1}H NMR (500 MHz, $CDCl_3$) δ 1.63–1.71 (m, 1H, C(O)CC*H$_a$*H$_b$), 1.72–1.82 (m, 1H, C(O)CCH$_a$*H$_b$*), 1.82–1.98 (m, 2H, C*H₂*CH₂CH=CHAr), 2.07 (ddd, 1H, *J* = 13.7, 11.4, 4.8 Hz, CO₂CHC*H$_a$*H$_b$), 2.12–2.21 (m, 1H, CH₂C*H$_a$*H$_b$CH=CHAr), 2.22–2.33 (m, 1H, CH₂CH$_a$*H$_b$*CH=CHAr), 2.51 (dt, 1H, *J* = 13.4, 7.9 Hz, CO₂CHCH$_a$*H$_b$*), 3.82 (s, 3H, CO₂C*H₃*), 4.06 (d, 1H, *J* = 7.4 Hz, CH(Ph)₂), 4.96 (ddd, 0.82H, *J* = 10.8, 7.5, 3.6 Hz, CO₂C*H* from major diastereomer), 5.05 (ddd, 0.18H, *J* = 10.9, 6.9, 3.8 Hz, CO₂C*H* from minor diastereomer), 6.13 (dt, 1H, *J* = 15.8, 6.6 Hz, C*H*=CHAr), 6.38 (d, 1H, *J* = 15.8 Hz, CH=C*H*Ar), 7.17–7.24 (m, 5H, ArC*H*), 7.26–7.31 (m, 10H, ArC*H*) ppm; ^{13}C NMR (126 MHz, $CDCl_3$) δ 25.0 (*C*H₂CH₂CH=CHAr), 27.3 (C(O)C*C*H₂), 28.0 (CH₂*C*H₂CH=CHAr), 35.8 (CO₂CH*C*H₂), 53.0 (CO₂*C*H₃), 53.2 (*C$_q$*), 56.4 (*C*H(Ph)₂), 79.3 (CO₂*C*HCH(Ph)₂), 126.0 (Ar*C*H), 126.7 (Ar*C*H), 126.9 (Ar*C*H), 127.1 (Ar*C*H), 128.3 (Ar*C*H), 128.4 (Ar*C*H), 128.5 (Ar*C*H), 128.6 (Ar*C*H) 128.7 (Ar*C*H), 128.8 (Ar*C*H), 129.1 (*C*H=CHAr), 130.7 (CH=*C*HAr), 137.4 (Ar*C*), 140.1 (Ar*C*), 140.8 (Ar*C*), 170.2 (*C*O₂CH), 171.8 (*C*O₂CH₃) ppm; IR ν_{max} (thin film, cm^{-1}) = 3026, 2951, 1728, 1598, 1495, 1451, 1237, 170, 1096, 1031, 966, 921, 745, 697; HRMS calculated for $C_{30}H_{30}O_4Na$ [M+Na]$^+$ 477.2036, found 477.2026.

Rac–methyl (3_R_,6_R_)–6–cyclohexyl–2–oxo–3–((_E_)–4–phenylbut–3–en–1–yl)tetrahydro–2_H_–pyran–3–carboxylate (1h)

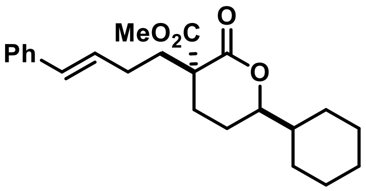

Prepared according to General Procedure D using dimethyl (*E*)–2–(3–cyclohexyl–3–oxopropyl)–2–(4–phenylbut–3–en–1–yl)malonate **S16** (80 mg, 0.20 mmol, 1.0 eq.), SmI_2 (5.0 mL, 0.1 M in THF, 0.50 mmol, 2.5 eq.), and H_2O (0.90 mL, 50 mmol, 250 eq.) in THF, followed by *p*TSA (2 mg, 0.010 mmol, 5 mol%) in CH_2Cl_2 (1 mL) to give the title product as a 5.0:1 mixture of diastereoisomers. Purification by silica gel column chromatography (hexane/EtOAc 9:1) yielded the title product (55 mg, 0.15 mmol, 74%). The major diastereoisomer could be isolated and was obtained as a yellow oil. ^1H NMR (400 MHz, $CDCl_3$) δ 0.98–1.25 (m, 4H, CyCH_2), 1.50–1.58 (m, 1H, CyC*H*), 1.63–1.86 (m, 7H, $CO_2CHCH_aH_b$ + C(O)CCH_aH_b + CyCH_2), 1.90–2.04 (m, 3H, $CO_2CHCH_aH_b$ + C$H_aH_bCH_2$CH=CHAr + CyCH_2), 2.10–2.38 (m, 3H, CH_2CH_2CH=CHAr + $CH_aH_bCH_2$CH=CHAr), 2.53 (dt, *J* = 13.6, 8.0 Hz, 1H, C(O)CCH_aH_b), 3.78 (s, 3H, CO_2CH_3), 3.93 (ddd, *J* = 10.5, 6.2, 3.8 Hz, 1H, CO_2C*H*), 6.18 (dt, *J* = 15.8, 6.5 Hz, 1H, C*H*=CHAr), 6.42 (d, *J* = 15.9 Hz, 1H, CH=C*H*Ar), 7.19–7.24 (m, 1H, ArC*H*), 7.28–7.35 (m, 4H, ArC*H*) ppm; ^{13}C NMR (101 MHz, $CDCl_3$) δ 23.0 (CyCH_2), 25.7 (CyCH_2), 25.9 (CyCH_2), 26.3 (CyCH_2), 27.3 (C(O)CCH_2), 28.0 (CyCH_2), 28.1 (CH_2CH_2CH=CHAr), 28.3 (CO_2CHCH_2), 36.0 (CH_2CH_2CH=CHAr), 42.3 (Cy*C*H), 53.0 (CO_2CH_3), 53.0 (C_q), 82.6 (CO_2*C*H), 126.0 (Ar*C*H), 127.1 (Ar*C*H), 128.5 (Ar*C*H), 129.2 (*C*H=CHAr), 130.7 (CH=*C*HAr), 137.5 (Ar*C*), 171.0 (CO_2CH), 171.8 (CO_2CH_3) ppm; IR v_{max} (thin film, cm^{-1}) = 2925, 2853, 1729 (C=O), 1447, 1234, 1175, 966; HRMS calcd. for $C_{23}H_{30}O_4Na$ $[M+Na]^+$ 393.2036, found 393.2019.

Rac–methyl (2_R_,5_R_)–6–oxo–5–((_E_)–4–phenylbut–3–en–1–yl)octahydro–2_H_,2'_H_–[2,4'–bipyran]–5–carboxylate (1i)

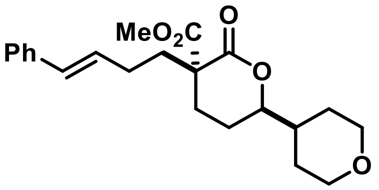

Prepared according to General Procedure D using dimethyl (*E)*–2–(3–oxo–3–(tetrahydro–2H–pyran–4–yl)propyl)–2–(4–phenylbut–3–en–1–yl) malonate **S18** (190 mg, 0.48 mmol, 1.0 eq.), SmI_2 (12 mL, 0.1 M in THF, 1.2 mmol, 2.5 eq.), and H_2O (2.2 mL mL, 120 mmol, 250 eq.) in THF, followed by treatment with *p*TSA (5.8 mg, 0.031 mmol, 5 mol%) in CH_2Cl_2 (3.1 mL). Purification by silica gel column chromatography (Hexane/EtOAc 9:1) yielded the title product as a yellow oil, as a 1.9:1 mixture of diastereoisomers (107 mg, 0.22 mmol, 36%). ^1H NMR (500 MHz, $CDCl_3$) δ 1.36–1.47 (m, 2H, *J* = 20.5, 12.2, 4.3 Hz, $O(CH_2)_2CH_2CH_2$CH), 1.49–1.53 (m, 1H, $O(CH_2)_2CH_2CH_aH_b$CH), 1.64–1.71 (m, 1H, $O(CH_2)_2CH_2CH_aH_b$CH), 1.75–1.88 (m, 3H, CH_2CH_2CH=CHAr + $CO_2CHCH_aH_b$), 1.90 (q, 1H, *J* = 1.6 Hz, C(O)CCH_aH_b), 1.95–2.03 (m, 1H, $CO_2CHCH_aH_b$), 2.10–2.19 (m, 1H, $O(CH_2)_2CH_2CH_2$C*H*), 2.20–2.33 (m, 2H, CH_2CH_2CH=CHAr), 2.55 (dt, 1H, *J* = 14.0, 8.0 Hz, C(O)CCH_aH_b), 3.36 (tdd, 2H, *J* = 11.9, 5.5, 2.2, CH_2OCH_2), 3.76 (s, 3H, CO_2CH_3), 3.92 (ddd, 0.75H, *J* = 10.4, 7.1, 3.8, Hz, CO_2C*H* from major diastereomer), 3.97–4.03 (m, 2H, CH_2OCH_2), 4.07–4.13 (m, 0.25H, CO_2C*H* from minor diastereomer), 6.12 (m, 1H, C*H*=CHAr), 6.41 (dd, 1H, *J* = 16.0, 1.6 Hz, CH=C*H*Ar), 7.17–7.23 (m, 1H, ArC*H*), 7.28–7.34 (m,

4H, ArC*H*) ppm; ^{13}C NMR (126 MHz, $CDCl_3$) δ 23.0 (CO_2CHCH_2), 27.0 C(O)CCH_2), 27.9 (O(CH_2)$_2$(CH_2)$_2$CH), 28.1 (CH_2CH_2CH=CHAr), 28.8 (O(CH_2)$_2$(CH_2)$_2$CH), 36.0 (O(CH_2)$_2$(CH_2)$_2$CH), 40.0 (CH_2CH_2CH=CHAr), 53.0 (CO_2CH_3), 53.1 (C_q), 67.4 (CH_2OCH_2), 67.6 (CH_2OCH_2), 81.7 (CO_2CHCH from major diastereomer), 85.2 (CO_2CHCH form minor diastereomer), 126.0 (ArCH), 126.0 (ArCH), 127.1 (ArCH), 128.5 (ArCH), 129.0 (CH=CHAr), 130.6 (CH=CHAr), 130.9 (ArCH), 137.4 (ArC), 170.6 (CO_2CH_3), 171.6 (CO_2CH)) ppm; IR ν_{max} (thin film, cm^{-1}) = 2951, 2845, 1728, 1494, 1446, 1239, 1192, 1141, 1092, 1019, 966, 745, 695; HRMS calculated for $C_{22}H_{28}O_5Na$ [M+Na]$^+$ 395.1829, found 395.1823.

Rac–methyl (3R,6R)–6–(tert–butyl)–2–oxo–3–((E)–4–phenylbut–3–en–1–yl)tetrahydro–2H–pyran–3–carboxylate (1j)

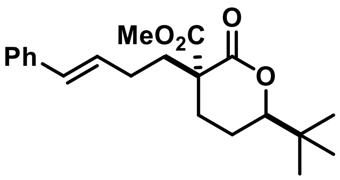

Prepared according to General Procedure D using dimethyl (*E*)–2–(4,4–dimethyl–3–oxopentyl)–2–(4–phenylbut–3–en–1–yl)malonate **S20** (300 mg, 0.80 mmol, 1.0 eq.), SmI_2 (20 mL, 0.1 M in THF, 2.0 mmol, 2.5 eq.), and H_2O (2.6 mL, 200 mmol, 250 eq.) in THF, followed by *p*TSA (7.6 mg, 0.04 mmol, 5 mol%) in CH_2Cl_2 (8 mL) to give the title product as a 9.0:1 mixture of diastereoisomers. Purification by silica gel column chromatography (hexane/EtOAc 9:1 to CH_2Cl_2) yielded the title product (69 mg, 0.20 mmol, 25%). The major diastereoisomer could be isolated and was obtained as a yellow oil. ^{1}H NMR (400 MHz, $CDCl_3$) δ 0.97 (s, 9H, C(CH_3)$_3$), 1.65–1.74, (m, 1H, CH_aH_bCHC(CH_3)$_3$), 1.82 (ddd, *J* = 13.8, 8.0, 4.2 Hz, 1H, C(O)CCH_aH_b), 1.88–2.05 (m, 2H, CH_aH_bCHC(CH_3)$_3$ + C$H_aH_b$$CH_2CH$=CHAr), 2.11–2.37 (m, 3H, CH_2CH_2CH=CHAr + C$H_aH_b$$CH_2CH$=CHAr), 2.54 (dt, *J* = 13.8, 8.2 Hz, 1H, C(O)CCH_aH_b), 3.79 (s, 3H, CO_2CH_3), 3.82 (dd, *J* = 15.9, 6.5 Hz, 1H, CO_2C*H*), 6.18 (dt, *J* = 15.9, 6.5 Hz, 1H, C*H*=CHAr), 6.42 (d, *J* = 15.9 Hz, 1H, CH=C*H*Ar), 7.14–7.23 (m, 1H, ArC*H*), 7.28–7.36 (m, 4H, ArC*H*) ppm; ^{13}C NMR (101 MHz, $CDCl_3$) δ 20.9 (CH_2CHC(CH_3)$_3$), 25.3 (C(CH_3)$_3$), 27.4 (C(O)CCH_2), 28.1 (CH_2CH=CHAr), 34.2 (*C*(CH_3)$_3$), 36.0 (CH_2CH_2CH=CHAr), 52.8 (C_q), 53.0 (CO_2CH_3), 85.7 (CO_2CH), 126.0 (ArCH), 127.1 (ArCH), 128.5 (ArCH), 129.2 (CH=CHAr), 130.7 (CH=CHAr), 137.5 (ArC), 171.0 (CO_2CH), 171.8 (CO_2CH_3) ppm; IR ν_{max} (thin film, cm^{-1}) = 2955, 1730 (C=O), 1447, 1250, 1172, 965; HRMS calcd. for $C_{21}H_{28}O_4$ [M+Na]$^+$ 367.1880, found 367.1868.

Rac–methyl (3R,6S)–6–ethyl–3–((E)–4–(2–fluorophenyl)but–3–en–1–yl)–2–oxotetrahydro–2H–pyran–3–carboxylate (1k)

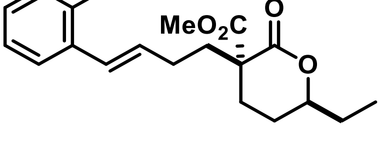

Prepared according to General Procedure D using dimethyl (*E*)–2–(4–(2–fluorophenyl)but–3–en–1–yl)–2–(3–oxopentyl)malonate **S22** (100 mg, 0.27 mmol, 1.0 eq.), SmI_2 (8.2 mL, 0.1 M in THF, 0.82 mmol, 2.5 eq.), and H_2O (1.5 mL, 82 mmol, 250 eq.) in THF, followed by *p*TSA (2.6 mg, 0.014 mmol, 5 mol%) in CH_2Cl_2 (5 mL). Purification by silica gel column chromatography (hexane/EtOAc 9:1) yielded the title product as a colourless oil, as a 5.2:1 mixture of diastereoisomers (71 mg, 0.21 mmol, 79%). ^{1}H NMR (500 MHz, $CDCl_3$)

δ 1.00 (t, J = 7.5 Hz, 3H, CH_2CH_3), 1.59–1.70 (m, 2H, $CH_aH_bCH_3$ + $CO_2CHCH_aH_b$), 1.70–1.79 (m, 1H, $CH_aH_bCH_3$), 1.83 (ddd, J = 13.7, 8.2, 5.0 Hz, 1H, C(O)CCH_aH_b), 1.96–2.08 (m, 2H, $CH_aH_bCH_2CH$=CHAr + $CO_2CHCH_aH_b$), 2.16 (ddd, J = 13.6, 11.4, 4.9 Hz, 1H, $CH_aH_bCH_2CH$=CHAr), 2.21–2.30 (m, 1H, $CH_2CH_aH_bCH$=CHAr), 2.31–2.39 (m, 1H, $CH_2CH_aH_bCH$=CHAr), 2.55 (dt, J = 14.0, 8.0 Hz, 1H, C(O)CCH_aH_b), 3.79 (s, 3H, CO_2CH_3), 4.12 (dddd, J = 10.6, 6.8, 5.6, 3.8 Hz, 0.84H, CO_2CH from major diastereoisomer), 4.26–4.33 (m, 0.16H, CO_2CH from minor diastereoisomer), 6.26 (dt, J = 16.0, 6.8 Hz, 1H, *CH*=CHAr), 6.57 (d, J = 16.1 Hz, 1H, CH=*CH*Ar), 7.01 (ddd, J = 10.9, 8.2, 1.3 Hz, 1H, Ar*CH*), 7.07 (td, J = 7.4, 1.2 Hz, 1H, Ar*CH*), 7.17 (tdd, J = 7.4, 5.1, 1.8 Hz, 1H, Ar*CH*), 7.40 (td, J = 7.7, 1.8 Hz, 1H, Ar*CH*) ppm; ^{13}C NMR (126 MHz, $CDCl_3$) δ 9.4 (CH_2CH_3), 25.5 (CO_2CHCH_2), 27.0 (C(O)CCH_2), 28.5 (CH_2CH_2CH=CHAr), 28.6 (CH_2CH_3), 35.9 (CH_2CH_2CH=CHAr), 53.0 (CO_2CH_3), 53.1 (C_q), 79.8 (CO_2CH), 115.6 (d, J = 22.2 Hz, Ar*C*H), 123.1 (CH=*C*HAr), 124.0 (d, J = 3.4 Hz, Ar*C*H), 125.1 (d, J = 12.2 Hz, Ar*C*), 127.1 (d, J = 4.0 Hz, Ar*C*H), 128.3 (d, J = 8.4 Hz, Ar*C*H), 131.9 (d, J = 4.0 Hz, *C*H=CHAr), 160.0 (d, J = 248.6 Hz, Ar*C*F), 170.9 (*C*O_2CH), 171.8 (*C*O_2CH_3) ppm; ^{19}F NMR (376 MHz, $CDCl_3$) δ –118.7 ppm; IR ν_{max} (thin film, cm^{-1}) = 2950, 1727 (C=O), 1489, 1455, 1227, 1192, 1091; HRMS calcd. for $C_{19}H_{23}O_4FNa$ $[M+Na]^+$ 357.1473, found 347.1458.

***Rac*–methyl (3*R*,6*S*)–6–ethyl–2–oxo–3–((*E*)–4–(o–tolyl)but–3–en–1–yl)tetrahydro–2*H*–pyran–3–carboxylate (1l)**

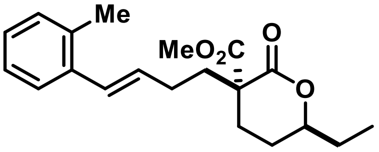

Prepared according to General Procedure D using dimethyl (*E*)–2–(3–oxopentyl)–2–(4–(o–tolyl)but–3–en–1–yl)malonate **S23** (107 mg, 0.3 mmol, 1.0 eq.), SmI_2 (7.5 mL, 0.1 M in THF, 0.75 mmol, 2.5 eq.), and H_2O (1.35 mL, 75 mmol, 250 eq.) in THF, followed by treatment with *p*TSA (2.5 mg,0.013 mmol, 5 mol%) in CH_2Cl_2 (1.3 mL) to give the title product as a single diastereoisomer. Purification by silica gel column chromatography (Hexane/EtOAc 9:1) yielded the title product as a pale oil (38 mg, 0.12 mmol, 44%). ^{1}H NMR (400 MHz, $CDCl_3$) δ 0.99 (t, 3H, J = 7.5 Hz, CH_3CH_2CH), 1.59–1.68 (m, 2H, J = 17.5, 8.4, 4.8, 2.2 Hz, CH_3CH_2CH), 1.70–1.77 (m, 1H, C(O)CCH_aH_b), 1.84 (ddd, 1H, J = 13.9, 8.1, 5.1, $CO_2CHCH_aH_b$), 1.96–2.07 (m, 2H, CH_2CH_2CH=CHAr), 2.15 (ddd, 2H, J = 16.2, 8.4, 4.7 Hz, CH_2CH_2CH=CHAr), 2.22–2.29 (m, 1H, CO_2CHCH_2), 2.33 (s, 3H, CH_3Ar), 2.55 (dt, 1H, J = 14.0, 7.9 Hz, C(O)CCH_2), 3.78 (s, 3H, OCH_3), 4.11 (dddd, 1H, J = 10.7, 6.8, 5.6, 3.8 Hz, CO_2CH), 6.03 (dt, 1H, J = 15.7, 6.6 Hz, CH_2CH_2CH=CHAr), 6.60 (d, 1H, J = 15.5 Hz, CH_2CH_2CH=*CH*Ar), 7.08–7.18 (m, 3H, Ar*CH*), 7.34–7.40 (m, 1H, Ar*CH*) ppm; ^{13}C NMR (101 MHz, $CDCl_3$) δ 9.4 (CH_2CH_3), 19.8 (ArCH_3), 25.5 (CH_2CH_2CH=CHAr), 27.1 (C(O)CCH_aH_b), 28.4 ($CO_2CHCH_aH_b$), 28.6 (CH_2CH_3), 36.2 (CH_2CH_2CH=CHAr), 53.1 (OCH_3), 53.1 (C_q), 79.8 (CO_2CH), 125.5 (Ar*C*H), 126.0 (Ar*C*H), 127.0 (Ar*C*H), 128.6 (CH_2CH_2CH=CHAr), 130.2 (Ar*C*H), 130.5 (CH_2CH_2CH=*C*HAr), 135.0 (Ar*C*), 136.7 (Ar*C*), 170.9 (*C*O_2CH), 171.8 (*C*O_2CH_3) ppm; IR ν_{max} (thin film, cm^{-1}) = 2951, 1729, 1484, 1458, 1241, 1191, 1093, 966, 749; HRMS calculated for $C_{20}H_{27}O_4$ $[M+H]^+$ 331.1904, found 331.1899.

Rac–methyl (3R,6S)–6–ethyl–2–oxo–3–((E)–4–(m–tolyl)but–3–en–1–yl)tetrahydro–2H–pyran–3–carboxylate (1m)

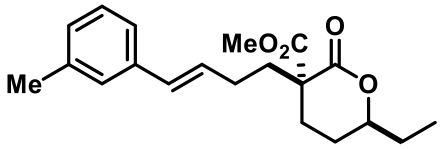

Prepared according to General Procedure D using dimethyl (*E*)–2–(3–oxopentyl)–2–(4–(*m*–tolyl)but–3–en–1–yl)malonate **S24** (136 mg, 0.38 mmol, 1.0 eq.), SmI_2 (9.5 mL, 0.1 M in THF, 0.95 mmol, 2.5 eq.), and H_2O (1.7 mL, 95 mmol, 250 eq.) in THF, followed by treatment with *p*TSA (3.5 mg, 0.019 mmol, 5 mol%) in CH_2Cl_2 (1.9 mL) to give the title product as a single diastereoisomer. Purification by silica gel column chromatography (Hexane/EtOAc 9:1) yielded the title product as a yellow oil (43 mg, 0.13 mmol, 36%). ^1H NMR (400 MHz, $CDCl_3$) δ 0.99 (t, 3H, *J* = 7.4 Hz, CH_2CH_3), 1.59–1.68 (m, 2H, CH_2CH_3), 1.69–1.76 (m, 1H, $CO_2CHCH_aH_b$), 1.81 (ddd, 1H, *J* = 17.5, 8.4, 4.4 Hz, $C(O)CCH_aH_b$), 1.99 (ddt, 2H, *J* = 13.2, 10.7, 2.9 Hz, CH_2CH_2CH=CHAr), 2.10–2.24 (m, 2H, CH_2CH_2CH=CHAr), 2.25–2.30 (m, 1H, $CO_2CHCH_aH_b$), 2.33 (s, 3H, $ArCH_3$), 2.54 (dt, 1H, *J* = 13.9, 8.0 Hz, $C(O)CCH_aH_b$), 3.78 (s, 3H, OCH_3), 4.07–4.15 (m, 1H, CO_2CH), 6.15 (dt, 1H, *J* = 15.8, 6.6 Hz, CH_2CH_2CH=CHAr), 6.38 (d, 1H, *J* = 15.9 Hz, CH_2CH_2CH=CHAr), 7.01 (d, 1H, *J* = 7.4 Hz, ArCH), 7.20–7.10 (m, 3H, ArCH) ppm; ^{13}C NMR (101 MHz, $CDCl_3$) δ 9.4 (CH_2CH_3), 21.4 ($ArCH_3$), 25.5 (CH_2CH_2CH=CHAr), 27.0 ($C(O)CCH_2$), 28.1 (CO_2CHCH_2), 28.6 (CH_2CH_3), 36.1 (CH_2CH_2CH=CHAr), 53.0 (OCH_3), 53.1 (C_q), 79.8 (CO_2CH), 123.1 (ArCH), 126.7 (ArCH), 127.8 (ArCH), 128.4 (ArCH), 128.9 (CH_2CH_2CH=CHAr), 130.7 (CH_2CH_2CH=CHAr), 137.4 (ArC), 138.0 (ArC), 170.9 (CO_2CH), 171.8 (CO_2CH_3) ppm; IR ν_{max} (thin film, cm^{-1}) = 2951, 1729, 1603, 1455, 1240, 1191, 1091, 965, 777, 693; HRMS calculated for $C_{20}H_{27}O_4$ $[M+H]^+$ 331.1904, found 331.1900.

Rac–methyl (3R,6S)–3–((E)–4–(3–chlorophenyl)but–3–en–1–yl)–6–ethyl–2–oxotetrahydro–2H–pyran–3–carboxylate (1n)

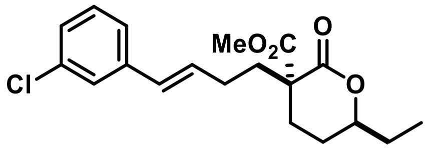

Prepared according to General Procedure D using dimethyl (*E*)–2–(4–(3–chlorophenyl)but–3–en–1–yl)–2–(3–oxopentyl)malonate **S25** (130 mg, 0.33 mmol, 1.0 eq.), SmI_2 (8.2 mL, 0.1 M in THF, 0.82 mmol, 2.5 eq.), and H_2O (1.5 mL, 83 mmol, 250 eq.) in THF, followed by *p*TSA (3.0 mg, 0.017 mmol, 5 mol%) in CH_2Cl_2 (5 mL). Purification by silica gel column chromatography (hexane/EtOAc 9:1) yielded the title product as a 4.3:1 mixture of diastereoisomers (94 mg, 0.27 mmol, 81%). The major diastereoisomer could be isolated and was obtained as a colourless oil. ^1H NMR (400 MHz, $CDCl_3$) δ 1.00 (t, *J* = 7.4 Hz, 3H, CH_2CH_3), 1.56–1.71 (m, 2H, $CH_aH_bCH_3$ + $CO_2CHCH_aH_b$), 1.70–1.89 (m, 2H, $CH_aH_bCH_3$ + $C(O)CCH_aH_b$), 1.95–2.06 (m, 2H, $CO_2CHCH_aH_b$ + $CH_aH_bCH_2CH$=CHAr), 2.14 (tdd, *J* = 11.3, 4.9, 1.8 Hz, 1H, $CH_aH_bCH_2CH$=CHAr), 2.19–2.26 (m, 1H, $CH_2CH_aH_bCH$=CHAr), 2.27–2.37 (m, 1H, $CH_2CH_aH_bCH$=CHAr), 2.55 (ddd, *J* = 13.9, 8.9, 7.0 Hz, 1H, $C(O)CCH_aH_b$), 3.79 (s, 3H, CO_2CH_3), 4.06–4.17 (m, 1H, CO_2CH), 6.20 (dt, *J* = 15.7, 6.5 Hz, 1H, CH=CHAr), 6.36 (d, *J* = 15.8 Hz, 1H, CH=CHAr), 7.15–7.24 (m, 3H, ArCH), 7.27–7.33 (m, 1H, ArCH) ppm; ^{13}C NMR (101 MHz, $CDCl_3$) δ 9.4 (CH_2CH_3), 25.5 (CO_2CHCH_2), 27.1 ($C(O)CCH_2$), 28.0 (CH_2CH_2CH=CHAr), 28.6 (CH_2CH_3), 35.9 (CH_2CH_2CH=CHAr), 53.0 (C_q), 53.1 (CO_2CH_3), 79.8 (CO_2CH), 124.2 (ArCH), 126.0 (ArCH), 127.0 (ArCH), 129.5 (CH=CHAr), 129.7 (ArCH), 130.8 (CH=CHAr), 134.5

(Ar*C*Cl), 139.4 (Ar*C*), 170.8 (*C*O_2CH), 171.7 (*C*O_2CH_3) ppm; IR ν_{max} (thin film, cm^{-1}) = 2945, 1729 (C=O), 1455, 1272, 1245, 1193, 1090; HRMS calcd. for $C_{19}H_{23}O_4ClNa$ $[M+Na]^+$ 373.1177, found 373.1164.

Rac–methyl (3*R*,6*S*)–6–ethyl–3–((*E*)–4–(naphthalen–2–yl)but–3–en–1–yl)–2–oxotetrahydro–2*H*–pyran–3–carboxylate (1o)

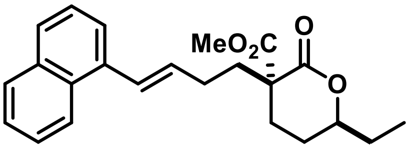

Prepared according to General Procedure D using dimethyl (*E*)–2–(4–(naphthalen–1–yl)but–3–en–1–yl)–2–(3–oxopentyl)malonate **S26** (120 mg, 0.29 mmol, 1.0 eq.), SmI_2 (7.3 mL, 0.1 M in THF, 0.73 mmol, 2.5 eq.), and H_2O (1.3 mL, 73 mmol, 250 eq.) in THF, followed by *p*TSA (2.8 mg, 0.015 mmol, 5 mol%) in CH_2Cl_2 (4 mL). Purification by silica gel column chromatography (hexane/EtOAc 9:1) yielded the title product as white crystals, as a 6.4:1 mixture of diastereoisomers (65 mg, 0.018 mmol, 61%). ^{1}H NMR ($CDCl_3$, 400 MHz) δ 1.01 (t, *J* = 7.5 Hz, 3H, CH_2CH_3), 1.61–1.81 (m, 3H, CH_2CH_3 + $CO_2CHCH_aH_b$), 1.81–1.90 (m, 1H, C(O)CCH_aH_b), 1.98–2.13 (m, 2H, C(O)CCH_aH_b + $CH_aH_bCH_2$CH=CHAr), 2.14–2.45 (m, 3H, $CH_aH_bCH_2$CH=CHAr + CH_2CH=CHAr), 2.57 (dt, *J* = 14.0, 7.9 Hz, 1H, C(O)CCH_aH_b), 3.81 (s, 3H, CO_2CH_3), 4.14 (dddd, *J* = 10.6, 6.8, 5.5, 3.8 Hz, 0.85H, CO_2C*H* from major diastereoisomer), 4.25–4.33 (m, 0.15H, CO_2C*H* from minor diastereoisomer), 6.19 (dt, *J* = 15.8, 6.7 Hz, 1H, C*H*=CHAr), 6.81 (d, *J* = 15.8 Hz, 1H, CH=C*H*Ar), 7.11–7.25 (m, 4H, ArC*H*), 7.29 (s, 1H, ArC*H*), 7.35 (dd, *J* = 7.8, 1.5 Hz, 1H, ArC*H*), 7.50 (dd, *J* = 7.7, 1.5 Hz, 1H, ArC*H*) ppm; ^{13}C NMR (101 MHz, $CDCl_3$) δ 9.4 (CH_2CH_3), 25.5 (CO_2CHCH_2), 27.1 (C(O)CCH_2), 28.3 (CH_2CH=CHAr), 28.6 (CH_2CH_3), 35.9 (CH_2CH_2CH=CHAr), 53.0 (CO_2CH_3), 53.1 (C_q), 79.8 (CO_2*C*H), 126.7 (Ar*C*H), 126.8 (ArC*H*), 126.9 (CH=*C*HAr), 126.9 (Ar*C*H), 128.1 (Ar*C*H), 129.6 (Ar*C*H), 132.2 (Ar*C*), 135.4 (Ar*C*), 135.5 (Ar*C*), 170.8 (*C*O_2CH), 171.8 (*C*O_2CH_3) ppm; IR ν_{max} (thin film, cm^{-1}) = 2951, 1728 (C=O), 1470, 1241, 1190, 1118, 1032; HRMS calcd. for $C_{23}H_{26}O_4Na$ $[M+Na]^+$ 389.1723, found 389.1710.

Rac–methyl (3*R*,6*S*)–3–((*E*)–4–([1,1'–biphenyl]–4–yl)but–3–en–1–yl)–6–ethyl–2–oxotetrahydro–2*H*–pyran–3–carboxylate (1p)

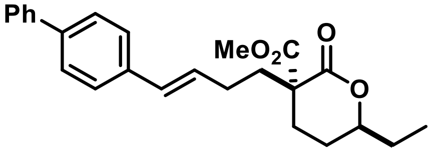

Prepared according to General Procedure D using dimethyl (*E*)–2–(4–([1,1'–biphenyl]–4–yl)but–3–en–1–yl)–2–(3–oxopentyl)malonate **S27** (171 mg, 0.41 mmol, 1.0 eq.), SmI_2 (10.3 mL, 0.1 M in THF, 1.03 mmol, 2.5 eq.), and H_2O (1.85 mL, 103 mmol, 250 eq.) in THF, followed by treatment with *p*TSA (4.4 mg, 0.023 mmol, 5 mol%) in CH_2Cl_2 (2.3 mL) to give the title product as a single diastereoisomer. Purification by silica gel column chromatography (Hexane/EtOAc 9:1) yielded the title major diastereoisomer product as a yellow oil (70.7 mg, 0.18 mmol, 39%). ^{1}H NMR (400 MHz, $CDCl_3$) δ 0.99 (t, 3H, *J* = 7.5 Hz, CH_2CH_3), 1.58–1.69 (m, 2H, CH_2CH_3), 1.74 (td, 1H, *J* = 15.7, 15.0, 7.8 Hz, $CH_aH_bCH_2$CH=CHAr), 1.83 (ddd, 1H, *J* = 13.7, 8.2, 5.1 Hz, C(O)CCH_aH_b), 1.97–2.06 (m, 2H, CO_2CHCH_2), 2.16 (ddd, 1H, *J* = 13.5, 10.8, 4.8 Hz, $CH_2CH_aH_b$CH=CHAr), 2.24 (tt, 1H, *J* = 12.4, 5.6 Hz, $CH_aH_bCH_2$CH=CHAr), 2.29–2.38 (m, 1H, $CH_2CH_aH_b$CH=CHAr), 2.55 (dt, 1H, *J* = 13.9, 8.0 Hz, C(O)CCH_aH_b), 3.79 (s, 3H, OCH_3), 4.12 (dq, 1H, *J* =

10.7, 6.0, CO_2CH), 6.22 (dt, 1H, J = 15.8, 6.7 Hz, CH_2CH_2CH=CHAr), 6.45 (d, 1H, J = 15.8 Hz, CH_2CH_2CH=C*H*Ar), 7.33 (t, 1H, J = 7.4 Hz, ArC*H*), 7.38–7.45 (m, 4H, ArC*H*), 7.53 (d, 2H, J = 8.2 Hz, ArC*H*), 7.59 (d, 2H, J = 7.3 Hz, ArC*H*) ppm; ^{13}C NMR (101 MHz, $CDCl_3$) δ 9.4 (CH_2CH_3), 25.5 (CO_2CHCH_2), 27.1 (C(O)CCH_2), 28.1 (CH_2CH_2CH=CHAr), 28.6 (CH_2CH_3), 36.0 (CH_2CH_2CH=CHAr), 53.0 (OCH_3), 53.2 (C_q), 79.8 (CO_2CH), 126.4 (ArCH), 126.9 (ArCH), 127.2 (ArCH), 127.2 (ArCH), 128.8 (ArCH), 129.3 (CH=CHAr), 130.2 (CH=CHAr), 136.5 (ArC), 139.8 (ArC), 140.8 (ArC), 170.9 (CO_2CH), 171.8 (CO_2CH_3) ppm; IR ν_{max} (thin film, cm^{-1}) = 2917, 2849, 1732, 1615, 1458, 1326, 1240, 1164, 1121, 1067, 1016, 969, 862, 816, 745; HRMS calculated for $C_{25}H_{28}O_4Na$ $[M+Na]^+$ 415.1880, found 415.1863.

***Rac*–methyl (3*R*,6*S*)–6–ethyl–3–((*E*)–4–(4–fluorophenyl)but–3–en–1–yl)–2–oxotetrahydro–2*H*–pyran–3–carboxylate (1q)**

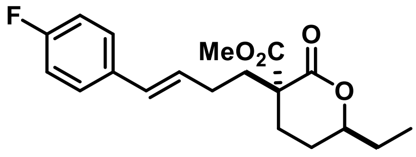

Prepared according to General Procedure D using dimethyl (*E*)–2–(4–(4–fluorophenyl)but–3–en–1–yl)–2–(3–oxopentyl)malonate **S28** (88 mg, 0.24 mmol, 1.0 eq.), SmI_2 (6 mL, 0.1 M in THF, 0.6. mmol, 2.5 eq.), and H_2O (1.1 mL, 60 mmol, 250 eq.) in THF, followed by treatment with *p*TSA (2.1 mg, 0.011 mmol, 5 mol%) in CH_2Cl_2 (1.1 mL) to give the product as a single diastereoisomer. Purification by silica gel column chromatography (Hexane/EtOAc 9:1) yielded the product as a yellow oil (15 mg, 0.046 mmol, 21%). ^1H NMR (400 MHz, $CDCl_3$) δ 0.98 (t, 3H, J = 7.4 Hz, CH_2CH_3), 1.58–1.68 (m, 2H, CH_2CH_3), 1.73 (q, 1H, J = 7.4 Hz, C(O)CCH_aH_b), 1.77–1.86 (m, 1H, J = 14.5, 8.6, 5.4 Hz, $CO_2CHCH_aH_b$), 1.99 (ddd, 2H, J = 13.2, 9.8, 4.1 Hz, CH_2CH_2CH=CHAr), 2.08–2.24 (m, 2H, CH_2CH_2CH=CHAr), 2.31 (ddd, 1H, J = 17.5, 9.0, 5.6 Hz, C(O)CCH_aH_b), 2.54 (dt, 1H, J = 14.9, 7.9 Hz, $CO_2CHCH_aH_b$), 3.77 (s, 3H, CO_2CH_3), 4.11 (dq, 1H, J = 10.6, 5.2 Hz, CO_2CH), 6.08 (dt, 1H, J = 15.2, 6.4 Hz, C*H*=CHAr), 6.37 (d, 1H, J = 15.8 Hz, CH=C*H*Ar), 6.94–7.01 (m, 2H, ArC*H*), 7.27–7.30 (m, 2H, ArC*H*) ppm; ^{13}C NMR (101 MHz, $CDCl_3$) δ 9.4 (CH_2CH_3), 25.5 (CH_2CH_2CH=CHAr), 27.1 (CH_2CH_3), 28.0 (C(O)CCH_2), 28.6 (CO_2CHCH_2), 36.0 (CH_2CH_2CH=CHAr), 53.0 (CO_2CH_3), 53.1 (C_q), 79.8 (CO_2CH), 115.4 (d, J = 21.5 Hz, ArCH), 127.4 (d, J = 7.9 Hz, ArCH), 128.9 (CH=CHAr), 129.5 (CH=CHAr), 133.6 (ArC), 170.9 (CO_2CH), 171.8 (CO_2CH_3) ppm, ArCF is not observed; ^{19}F NMR (471 MHz, $CDCl_3$) δ –115.4 ppm; IR ν_{max} (thin film, cm^{-1}) = 3727, 2952, 2358, 1729, 1601, 1508, 1458, 1226, 1192, 1158, 1092, 968, 852, 821 668; HRMS calculated for $C_{19}H_{23}FO_4Na$ $[M+Na]^+$ 357.1473, found 357.1457.

***Rac*–methyl (3*R*,6*S*)–3–((*E*)–4–(4–bromophenyl)but–3–en–1–yl)–6–ethyl–2–oxotetrahydro–2*H*–pyran–3–carboxylate (1r)**

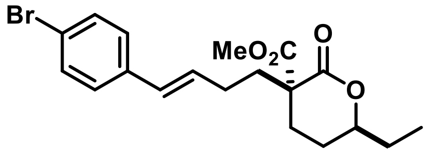

Prepared according to General Procedure D using dimethyl (*E*)–2–(4–(4–bromophenyl)but–3–en–1–yl)–2–(3–oxopentyl)malonate **S29** (136 mg, 0.32 mmol, 1.0 eq.), SmI_2 (8.0 mL, 0.1 M in THF, 0.80 mmol, 2.5 eq.), and H_2O (1.4 mL, 80 mmol, 250 eq.) in THF, followed by *p*TSA (3.0 mg, 0.016 mmol, 5 mol%) in CH_2Cl_2 (4 mL). Purification by silica gel column chromatography (hexane/EtOAc 9:1) yielded the title product as a

colourless oil, as a 6.2:1 mixture of diastereoisomers (78 mg, 0.20 mmol, 62%). ^{1}H NMR (400 MHz, $CDCl_3$) δ 0.99 (t, *J* = 7.5 Hz, 3H, CH_2CH_3), 1.52–1.86 (m, 4H, CH_2CH_3 + $CO_2CHCH_aH_b$ + $C(O)CCH_aH_b$), 1.94–2.06 (m, 2H, $CO_2CHCH_aH_b$ + $CH_aH_bCH_2CH$=CHAr), 2.09–2.35 (m, 3H, $CH_aH_bCH_2CH$=CHAr + CH_2CH=CHAr), 2.54 (dt, *J* = 13.9, 7.9 Hz, 1H, $C(O)CCH_aH_b$), 3.78 (s, 3H, CO_2CH_3), 4.02–4.20 (m, 0.9H, CO_2CH from major diastereoisomer), 4.22–4.30 (m, 0.1H, CO_2CH from minor diastereoisomer), 6.17 (dt, *J* = 15.8, 6.5 Hz, 1H, C*H*=CHAr), 6.36 (d, *J* = 15.9 Hz, 1H, CH=C*H*Ar), 7.19 (d, *J* = 8.5 Hz, 2H, ArC*H*), 7.42 (d, *J* = 8.5 Hz, 2H, ArC*H*) ppm; ^{13}C NMR (101 MHz, $CDCl_3$) δ 9.4 (CH_2CH_3), 25.5 (CO_2CHCH_2), 27.1 ($C(O)CCH_2$), 28.1 (CH_2CH=CHAr), 28.6 (CH_2CH_3), 35.9 (CH_2CH_2CH=CHAr), 53.0 (CO_2CH_3), 53.1 (C_q), 79.8 (CO_2CH), 120.7 (Ar*C*Br), 127.5 (Ar*C*H), 129.6 (CH=*C*HAr), 130.0 (*C*H=CHAr), 131.6 (Ar*C*H), 136.4 (Ar*C*), 170.8 (CO_2CH), 171.8 (CO_2CH_3) ppm; IR ν_{max} (thin film, cm^{-1}) = 2949, 1732 (C=O), 1455, 1385, 1250, 1039;HRMS calcd. for $C_{19}H_{23}O_4BrNa$ $[M+Na]^+$ 417.0672, found 417.0662.

Rac–methyl (3_R_,6_S_)–6–ethyl–2–oxo–3–((_E_)–4–(4–(trifluoromethyl)phenyl)but–3–en–1–yl)tetrahydro–2_H_–pyran–3–carboxylate (1s)

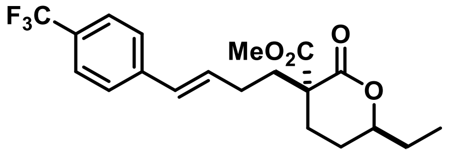

Prepared according to General Procedure D using dimethyl (*E*)–2–(3–oxopentyl)–2–(4–(4–(trifluoromethyl)phenyl)but–3–en–1–yl)malonate **S30** (172 mg, 0.41 mmol, 1.0 eq.), SmI_2 (10.3 mL, 0.1 M in THF, 1.03 mmol, 2.5 eq.), and H_2O (1.85 mL, 103 mmol, 250 eq.) in THF, followed by treatment with *p*TSA (4 mg, 0.021 mmol, 5 mol%) in CH_2Cl_2 (2.1 mL) to give the title product as a single diastereoisomer. Purification by silica gel column chromatography (Hexane/EtOAc 9:1) yielded the title product as a white solid (30.1 mg, 0.078 mmol, 19%). M.p. 91–92 °C. ^{1}H NMR (400 MHz, $CDCl_3$) δ 0.99 (t, 3H, *J* = 7.4 Hz, CH_2CH_3), 1.59–1.69 (m, 2H, CH_2CH_3), 1.73 (dd, 1H, *J* = 14.5, 7.3 Hz, $CO_2CHCH_aH_b$), 1.81 (ddd, 1H, *J* = 16.2, 7.9, 3.8 Hz, $C(O)CCH_aH_b$), 1.95–2.06 (m, 2H, *J* = 17.8, 8.5, 3.4 Hz, CH_2CH_2CH=CHAr), 2.14 (td, 1H, *J* = 12.3, 11.4, 4.8 Hz, $CO_2CHCH_aH_b$), 2.19–2.29 (m, 1H, $CH_2CH_aH_bCH$=CHAr), 2.35 (tt, 1H, *J* = 11.3, 5.6 Hz, $CH_2CH_aH_bCH$=CHAr), 2.54 (dt, 1H, *J* = 14.0, 7.9 Hz, $C(O)CCH_aH_b$), 3.78 (s, 3H, OCH_3), 4.11 (ddd, 1H, *J* = 10.6, 8.5, 5.0 Hz, CO_2CH), 6.28 (dt, 1H, *J* = 15.8, 6.5 Hz, C*H*=CHAr), 6.44 (d, 1H, *J* = 15.9 Hz, CH=C*H*Ar), 7.40 (d, 2H, *J* = 8.1 Hz, ArC*H*), 7.53 (d, 2H, *J* = 8.1 Hz, ArC*H*) ppm; ^{13}C NMR (101 MHz, $CDCl_3$) δ 9.4 (CH_2CH_3), 25.5 (CH_2CH_3), 27.1 ($C(O)CCH_2$), 28.1 (CH_2CH_2CH=CHAr), 28.6 (CO_2CHCH_2), 35.8 (CH_2CH_2CH=CHAr), 52.9 (OCH_3), 53.2 (C_q), 79.8 (CO_2CH), 125.5 (q, *J* = 3.8 Hz, 2 x Ar*C*H), 126.1 (2 x Ar*C*H), 129.5 (*C*H=CHAr), 131.9 (CH=*C*HAr), 140.9 (Ar*C*), 170.1 (CO_2CH), 171.7 (CO_2CH_3) ppm, ArCCF_3 and CF_3 are not observed; ^{19}F NMR (471 MHz, $CDCl_3$) δ –62.5 ppm; IR ν_{max} (thin film, cm^{-1}) = 3727, 3625, 2920, 2850, 2359, 1732, 1326, 1260, 1123, 1067, 683, 654; HRMS calculated for $C_{20}H_{23}F_3O_4Na$ $[M+Na]^+$ 407.1441, found 407.1431.

Rac–methyl (3_R_,6_S_)–6–ethyl–3–((_E_)–4–(4–methoxyphenyl)but–3–en–1–yl)–2–oxotetrahydro–2_H_–pyran–3–carboxylate (1t)

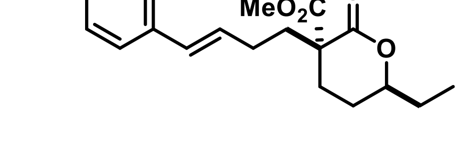

Prepared according to General Procedure D using dimethyl (*E*)–2–(4–(4–methoxyphenyl)but–3–en–1–yl)–2–(3–oxopentyl)malonate **S31** (120 mg, 0.33 mmol, 1.0 eq.), SmI_2 (9.9 mL, 0.1 M in THF, 0.99 mmol, 2.5 eq.), and H_2O (1.8 mL, 99 mmol, 250 eq.) in THF, followed by *p*TSA (3.1 mg, 0.017 mmol, 5 mol%) in CH_2Cl_2 (4 mL). Purification by silica gel column chromatography (hexane/EtOAc 9:1) yielded the title product as a 4.2:1 mixture of diastereoisomers (83 mg, 0.24 mmol, 73%). The major diastereoisomer could be isolated and was obtained as a colourless oil. ^{1}H NMR (400 MHz, $CDCl_3$) δ 0.99 (t, *J* = 7.5 Hz, 3H, CH_2CH_3), 1.59–1.70 (m, 2H, $CH_aH_bCH_3$ + $CO_2CHCH_aH_b$), 1.70–1.77 (m, 1H, $CH_aH_bCH_3$), 1.78–1.87 (m, 1H, C(O)CCH_aH_b), 1.96–2.05 (m, 2H, $CH_aH_bCH_2$CH=CHAr + $CO_2CHCH_aH_b$), 2.07–2.35 (m, 3H, $CH_aH_bCH_2$CH=CHAr + CH_2CH_2CH=CHAr), 2.55 (dt, *J* = 14.0, 8.0 Hz, 1H, C(O)CCH_aH_b), 3.78 (s, 3H, CO_2CH_3), 3.81 (s, 3H, ArOCH_3), 4.11 (dddd, *J* = 10.6, 6.8, 5.5, 3.8 Hz, 1H, CO_2CH), 6.03 (dt, *J* = 15.8, 6.6 Hz, 1H, C*H*=CHAr), 6.36 (d, *J* = 15.9 Hz, 1H, CH=C*H*Ar), 6.84 (d, *J* = 8.0 Hz, 2H, ArC*H*), 7.26 (d, *J* = 8.0 Hz, 2H, ArC*H*) ppm; ^{13}C NMR (101 MHz, $CDCl_3$) δ 9.4 (CH_2CH_3), 25.5 (CO_2CHCH_2), 27.0 (C(O)CCH_2), 28.0 (CH_2CH_2CH=CHAr), 28.6 (CH_2CH_3), 36.2 (CH_2CH_2CH=CHAr), 53.0 (C_q), 53.1 (CO_2CH_3), 55.3 (ArOCH_3), 79.8 (CO_2CH), 113.9 (Ar*C*H), 127.0 (*C*H=CHAr), 127.1 (Ar*C*H), 130.1 (CH=*C*HAr), 130.3 (Ar*C*), 158.8 (Ar*C*OCH_3), 170.9 (*C*O_2CH), 171.8 (*C*O_2CH_3); IR ν_{max} (thin film, cm^{-1}) = 2951, 1727 (C=O), 1606, 1510, 1455, 1244, 1174, 1091, 1030; HRMS calcd. for $C_{20}H_{26}O_5Na$ $[M+Na]^+$ 369.1659, found 369.1658.

***Rac*-methyl (3*R*,6*S*)-3-(but-3-en-1-yl)-6-methyl-2-oxotetrahydro-2H-pyran-3-carboxylate (1w)**

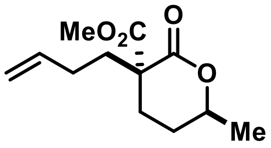

Prepared according to General Procedure D using dimethyl 2-(but-3-en-1-yl)-2-(3-oxobutyl)malonate[8] (2.0 mmol, 512 mg). Purification by silica gel column chromatography (hexane/Et_2O 6:4) yielded the title product as a 13:1 mixture of diastereoisomers (322 mg, 1.42 mmol, 71%). ^{1}H NMR (400 MHz, $CDCl_3$) δ 6.00 – 5.60 (m, 1H, CH_2=C*H*), 5.11 – 4.83 (m, 2H, CH_2=CH), 4.33 (dqd, *J* = 10.0, 6.2, 3.8 Hz, 1H, C*H*CH_3), 3.76 (s, 3H, OCH_3), 2.51 (dt, *J* = 14.0, 8.0 Hz, 1H, $CH_aH_bCH_2CHCH_3$), 2.17 – 1.85 (m, 5H, CH_2=CHCH_2, CH_2=CHCH_2CH_2, $CH_aH_bCHCH_3$), 1.78 (ddd, *J* = 14.0, 8.3, 4.9 Hz, 1H, $CH_aH_bCHCH_3$), 1.69 – 1.50 (m, 1H, $CH_aH_bCH_2CHCH_3$), 1.35 (d, *J* = 6.2 Hz, 3H, CHCH_3). ^{13}C NMR (101 MHz, $CDCl_3$) δ 171.9 (*C*O_2Me), 170.9 (*C*O_2CH), 137.5 (CH_2=*C*H), 115.4 (*C*H_2=CH), 74.9 (*C*HCH_3), 53.2 (OCH_3), 52.9 (*C*q), 35.7 (CH_2=CHCH_2CH_2), 28.8 (CH_2=CHCH_2), 27.9 (CH_2CHCH_3), 27.0 ($CH_2CH_2CHCH_3$), 21.5 (CHCH_3). HRMS calcd. for $C_{12}H_{18}O_4Na$ $[M+Na]^+$ 249.1097, found 249.1101. IR ν_{max} (thin film, cm^{-1}) = 3078, 2978, 2954, 2937, 1725, 1449.

***Rac*-methyl (3*S*,6*S*)-6-methyl-2-oxo-3-(3-oxopropyl)tetrahydro-2H-pyran-3-carboxylate (S33)**

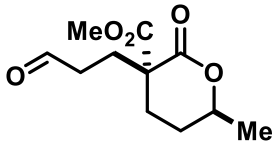

1w (1.0 eq., 3.00 mmol, 680 mg) was dissolved in MeOH (15 ml) and CH_2Cl_2 (15 ml) under air. The solution was cooled to –78 °C then O_3/O_2 gas mix was bubbled through the mixture until it became blue. Next, O_2 was bubbled through the mixture until the blue colour disappeared. PPh_3 (1.5 eq., 4.50 mmol, 1180 mg) was

added and the mixture was allowed to warm to r.t. overnight. The volatiles were removed *in vacuo* then the crude product was purified by silica gel column chromatography (hexane/Et_2O 1:9) yielding the title product as a >20:1 mixture of diastereoisomers (624 mg, 2.73 mmol, 91%). ^{1}H NMR (400 MHz, $CDCl_3$) δ 9.75 (t, *J* = 1.1 Hz, 1H, C*H*O), 4.34 (dqd, *J* = 10.0, 6.2, 3.8 Hz, 1H, C*H*CH$_3$), 3.77 (s, 3H, OC*H*$_3$), 2.72 (dddd, *J* = 18.5, 9.5, 5.8, 1.0 Hz, 1H, CHOCH_aH$_b$), 2.57 (dddd, *J* = 18.4, 9.4, 5.7, 1.1 Hz, 1H, CHOCH$_a$$H_b$), 2.47 (dt, *J* = 13.7, 7.9 Hz, 1H, CH_aH$_b$CH$_2$CHCH$_3$), 2.24 (ddd, *J* = 15.0, 9.3, 5.7 Hz, 1H, CHOCH$_2$CH_aH$_b$), 2.13 (ddd, *J* = 14.7, 9.5, 5.7 Hz, 1H, CHOCH$_2$CH$_a$$H_b$), 2.04 – 1.91 (m, 1H, C$H_aH_b$CHCH$_3$), 1.76 (ddd, *J* = 13.6, 7.8, 4.7 Hz, 1H, CH$_a$$H_bCH_2$CHCH$_3$), 1.64 (ddt, *J* = 16.0, 10.7, 3.8 Hz, 1H, CH$_a$$H_b$CHCH$_3$), 1.36 (d, *J* = 6.2 Hz, 3H, CHC*H*$_3$). ^{13}C NMR (101 MHz, $CDCl_3$) δ 200.8 (*C*HO), 171.7 (*C*O$_2$Me), 170.5 (*C*O$_2$CH), 75.4 (*C*HCH$_3$), 53.3 (O*C*H$_3$), 52.3 (*C*q), 39.7 (CHO*C*H$_2$), 28.4 (CHOCH$_2$*C*H$_2$), 28.1 (*C*H$_2$CH$_2$CHCH$_3$), 27.5 (*C*H$_2$CHCH$_3$), 21.5 (CH*C*H$_3$). HRMS calcd. for $C_{11}H_{16}O_5Na$ $[M+Na]^+$ 251.0890, found 251.0888. IR ν_{max} (thin film, cm^{-1}) = 2979, 2955, 2939, 2847, 2730, 1716, 2437, 1388.

Rac-methyl (3R,6S)-6-methyl-2-oxo-3-(4-(thiophen-2-yl)but-3-en-1-yl)tetrahydro-2H-pyran-3-carboxylate (1u)

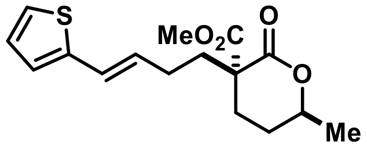

Triphenyl(thiophen-2-ylmethyl)phosphonium bromide[9] (1.3 eq., 0.78 mmol, 343 mg) was suspended in dry THF (2 ml) under N_2, then cooled to 0 °C. *n*-BuLi (1.3 eq., 0.78 mmol, 0.32 ml, 2.4 M in hexanes) was added dropwise at 0 °C, then stirring was continued for 30 min before **S33** (1 eq., 0.6 mmol, 137 mg) in dry THF (2 ml) was added dropwise. The reaction mixture was stirred for 2.5 h at 0 °C before warming to r.t. Stirring was continued for 13 h; TLC indicated complete consumption of **S33**. The reaction mixture was filtered through cotton wool, washed with Et_2O then evaporated. The crude product was purified by consecutive silica gel column chromatography (50% Et_2O in hexanes then 25% EtOAc in hexanes) yielding the title product as a 3.9:1 mixture of diastereomers with 1.4:1 ratio of *E*:*Z* isomers (32 mg, 0.104 mmol, 17%). ^{1}H NMR (400 MHz, $CDCl_3$) δ 7.24 (dd, *J* = 4.9, 1.3 Hz, 1H, ArC*H*, *Z*-alkene, major diastereomer), 7.08 (d, *J* = 5.1 Hz, 1H, ArC*H*, *E*-isomer, major diastereomer), 6.99 (dt, *J* = 6.1, 3.6 Hz, 2H, 2 × ArC*H*), 6.91 (dd, *J* = 5.1, 3.4 Hz, 1H, ArC*H*, *E*-isomer, major diastereomer), 6.85 (d, *J* = 3.5 Hz, 1H, ArC*H*, *E*-isomer, major diastereomer), 6.58 – 6.46 (m, 2H, 2 × ArC$_q$C*H*=CH), 6.00 (dt, *J* = 15.6, 6.7 Hz, 1H, ArC$_q$CH=C*H*, *E*-isomer, major diastereomer), 5.58 – 5.42 (m, 1H, ArC$_q$CH=C*H*, *Z*-isomer, major diastereomer), 4.45 (ddt, *J* = 11.1, 6.1, 3.0 Hz, 1H, C*H*CH$_3$, both alkene isomers, minor diastereomer), 4.34 (dddd, *J* = 12.4, 10.3, 6.2, 4.1 Hz, 2H, 2 × C*H*CH$_3$, both alkene isomers, major diastereomer), 3.77 (s, 6H, 2 × OCH$_3$, both alkene isomers, minor diastereomer), 3.76 (s, 6H, 2 × OCH$_3$, both alkene isomers, major diastereomer), 2.55 (tt, *J* = 13.9, 8.0 Hz, 2H, C*H*$_2$), 2.47 – 2.35 (m, 2H, C*H*$_2$), 2.33 – 2.07 (m, 4H, C*H*$_2$), 2.07 – 1.90 (m, 4H, C*H*$_2$), 1.89 – 1.74 (m, 2H, C*H*$_2$), 1.73 – 1.54 (m, 2H, C*H*$_2$), 1.38 (d, *J* = 6.3 Hz, 6H, 2 × CHC*H*$_3$, minor diastereomer, both alkene isomers), 1.35 (d, *J* = 6.2 Hz, 6H, 2 × CHC*H*$_3$, major diastereomer, both alkene isomers). ^{13}C NMR (101 MHz, $CDCl_3$) δ 171.8 (*C*O$_2$Me, *E*-alkene, major diastereomer), 171.8 (*C*O$_2$Me, *Z*-alkene, major diastereomer), 170.9 (*C*O$_2$CH, *Z*-alkene, major diastereomer), 170.8 (CO$_2$CH, *E*-alkene, major diastereomer), 142.7 (Ar*C*$_q$, *E*-alkene, major diastereomer), 140.3 (Ar*C*$_q$, *Z*-alkene, major diastereomer), 129.1 (CH=*C*HCH$_2$, *E*-alkene, major diastereomer), 129.0 (CH=*C*HCH$_2$, *Z*-alkene, major diastereomer), 127.6 (Ar*C*H, *Z*-alkene, major diastereomer),127.3 (Ar*C*H, *E*-alkene, major diastereomer), 126.9 (Ar*C*H, *Z*-alkene, major diastereomer), 125.3 (Ar*C*H, *Z*-alkene, major diastereomer), 124.7 (Ar*C*H, *E*-alkene, major diastereomer), 124.1 (Ar*C*H, *E*-alkene, major diastereomer), 123.5 (Ar*C*H, *E*-alkene, major diastereomer),

122.7 (Ar*C*H, *Z*-alkene, major diastereomer), 78.7 (CO_2*C*H, minor diastereomer, both alkene isomers), 74.9 (CO_2*C*H, major diastereomer, both alkene isomers), 53.2 (O*C*H_3, *E*-alkene, major diastereomer), 52.8 (Cq, *E*-alkene, major diastereomer), 36.0 (*C*H_2, both alkene isomers, major diastereomer), 27.9 (*C*H_2, both alkene isomers, major diastereomer), 27.8 (*C*H_2, *Z*-alkene, major diastereomer), 27.8 (*C*H_2, *E*-alkene, major diastereomer), 27.1 (*C*H_2, *E*-alkene, major diastereomer), 27.0 (*C*H_2, *Z*-alkene, major diastereomer), 21.5 (CH*C*H_3, both alkene isomers, major diastereomer). HRMS calcd. for $C_{16}H_{20}O_4SNa$ $[M+Na]^+$ 331.0975, found 331.0965. IR v_{max} (thin film, cm^{-1}) = 2977, 2951, 2935, 1728, 1448, 1435.

Note: The diastereomeric ratio was assigned based on the characteristic ^{1}H NMR peak of OC*H*CH_3. Similarly, the *E*/*Z* ratio was assigned based on the coupling constant and the shift of the CH=C*H*CH_2 peaks in the ^{1}H NMR.

Rac-methyl (3*R*,6*S*)-6-methyl-2-oxo-3-(4-phenylpent-3-en-1-yl)tetrahydro-2H-pyran-3-carboxylate (1v)

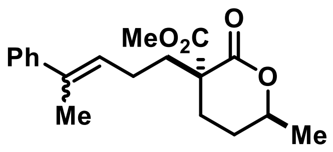

Triphenyl(1-phenylethyl)phosphonium bromide[10] (1.2 eq., 0.36 mmol, 161 mg) was suspended in dry THF (1 ml) under N_2, then cooled to 0 °C. *n*-BuLi (1.2 eq., 0.36 mmol, 0.15 ml, 2.4 M in hexanes) was added dropwise at 0 °C, then stirring was continued for 30 min before **S33** (1 eq., 0.3 mmol, 68.5 mg) in dry THF (0.5 ml) was added dropwise. The reaction mixture was stirred for 10 min at 0 °C before warming to r.t. Stirring was continued for 13 h; TLC indicated complete consumption of **S33**. The reaction mixture was filtered through cotton wool, washed with Et_2O then evaporated. Purification by silica gel column chromatography (50% Et_2O in hexanes) yielded the title product (30 mg, 0.095 mmol, 32%). ^{1}H NMR (400 MHz, $CDCl_3$, major diastereomer – alkene isomer unassigned) δ 7.43 – 7.18 (m, 5H, ArC*H*), 5.76 – 5.68 (m, 1H, C_q=C*H*), 4.41 – 4.30 (m, 1H, CH_3C*H*), 3.78 (s, 3H, OC*H*$_3$), 2.56 (dt, *J* = 14.0, 8.0 Hz, 1H, C*H*$_a$H$_b$), 2.30 – 2.16 (m, 2H, C_q=CHC*H*$_2$), 2.15 – 2.05 (m, 1H, C*H*$_a$H$_b$), 2.03 (dd, *J* = 1.4, 0.7 Hz, 3H, C_qC*H*$_3$), 2.07 – 1.91 (m, 2H, CH_3CHC*H*$_a$H$_b$, CH$_a$*H*$_b$), 1.84 (ddd, *J* = 14.0, 8.2, 4.9 Hz, 1H, CH$_a$*H*$_b$), 1.70 – 1.59 (m, 1H, CH_3CHCH$_a$*H*$_b$), 1.37 (d, *J* = 6.2 Hz, 3H, CHC*H*$_3$). ^{13}C NMR (126 MHz, $CDCl_3$, major diastereomer – alkene isomer unassigned) δ 171.9 (*C*O_2Me), 170.9 (*C*O_2CH), 143.7 (*C*$_q$), 136.1 (*C*$_q$), 128.3 (Ar*C*H), 126.8 (Ar*C*H), 126.7 (ArC$_q$C=*C*H), 125.7 (Ar*C*H), 74.9 (*C*HCH_3), 53.2 (O*C*H_3), 53.0 (*C*$_q$$CO_2$), 36.3 (*C*$H_2$), 27.9 ($CH_3$CH*C*$H_2$), 27.1 (*C*$H_2$), 24.1 ($C_q$=CH*C*$H_2$), 21.5 (CH*C*$H_3$), 15.9 ($C_q$*C*$H_3$). HRMS calcd. for $C_{19}H_{24}O_4Na$ $[M+Na]^+$ 339.1567, found 339.1557.

Note: The d.r. of the isolated product cannot be accurately determined by ^{1}H NMR analysis because of the presence of the cis and trans alkene isomers. However, the major diastereoisomer has been assigned based on comparison of its ^{13}C NMR to other lactone substrates. The *E*/*Z* ratio of the major diastereomer could not be determined because of the presence of diastereoisomers.

Rac-methyl (3*R*,6*S*)-6-methyl-3-(4-methylpent-3-en-1-yl)-2-oxotetrahydro-2H-pyran-3-carboxylate (1x)

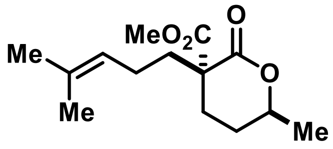

Isopropyltriphenylphosphonium iodide (1.2 eq., 0.36 mmol, 156 mg) was suspended in dry THF (0.5 ml) under N_2, then cooled to 0 °C. *n*-BuLi (1.2 eq., 0.36 mmol, 0.15 ml, 2.4 M in hexanes) was added dropwise at 0 °C, then stirring was continued for 30 min before **S33** (1 eq., 0.3 mmol, 68.5 mg) in dry THF (0.5 ml) was added dropwise. The reaction mixture was stirred for 10 min at 0 °C before warming to r.t. Stirring was continued for 3 h; TLC indicated complete consumption of **S33**. The reaction mixture was filtered through cotton wool, washed with Et_2O then evaporated. The crude product was purified by silica gel column chromatography (40% Et_2O in hexanes) yielding the title product as a 10:1 mixture of diastereoisomers (11 mg, 0.043 mmol, 14%). ^{1}H NMR (400 MHz, $CDCl_3$) δ 5.08 (tdt, *J* = 7.1, 3.2, 1.4 Hz, 1H), 4.33 (dqd, *J* = 10.0, 6.2, 3.8 Hz, 1H), 3.76 (s, 3H), 2.51 (dt, *J* = 14.0, 8.0 Hz, 1H), 2.09 – 1.92 (m, 4H), 1.88 – 1.74 (m, 2H), 1.66 (d, *J* = 1.4 Hz, 3H), 1.61 – 1.52 (m, 4H), 1.35 (d, *J* = 6.2 Hz, 3H). ^{13}C NMR (101 MHz, $CDCl_3$) δ 172.0, 171.0, 132.9, 123.1, 74.9, 53.1, 53.0, 36.6, 27.9, 26.9, 25.8, 23.3, 21.5, 17.8. HRMS calcd. for $C_{14}H_{22}O_4Na$ $[M+Na]^+$ 277.1410, found 277.1402.

Dimethyl 2-(3-methylbut-3-en-1-yl)-2-(3-oxobutyl)malonate (S34)

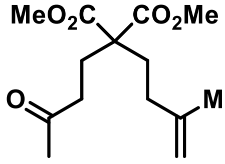

Prepared according to General procedure F using dimethyl 2-(3-methylbut-3-en-1-yl)malonate[11] (1.0 eq., 16.4 mmol, 3.29 g). Purification by silica gel column chromatography (hexane/EtOAc 8:2) yielded the title product as a white solid (3.61 g, 13.5 mmol, 82%). ^{1}H NMR (400 MHz, $CDCl_3$) δ 4.76 – 4.65 (m, 2H, C=CH_2), 3.72 (s, 6H, OCH_3), 2.44 (dd, *J* = 8.9, 6.8 Hz, 2H, CH_3COCH_2), 2.20 – 2.14 (m, 2H, $CH_3COCH_2CH_2$), 2.13 (s, 3H, CH_3COCH$_2$), 2.05 – 1.95 (m, 2H, CCH$_2$CH_2), 1.92 – 1.82 (m, 2H, CCH_2CH$_2$), 1.71 (t, *J* = 1.1 Hz, 3H, CCH_3). ^{13}C NMR (101 MHz, $CDCl_3$) δ 207.3 (CH$_3$*C*OCH$_2$), 171.9 (*C*O$_2$Me), 144.7 (C_q=CH$_3$), 110.5 (CH$_3$C$_q$=*C*H$_2$), 56.8 (C_q), 52.6 (O*C*H$_3$), 38.8 (CH$_3$CO*C*H$_2$), 32.3 (C*C*H$_2$CH$_2$), 32.0 (CCH$_2$*C*H$_2$), 30.1 (*C*H$_3$COCH$_2$), 26.8 (CH$_3$COCH$_2$*C*H$_2$), 22.7 (C$_q$=*C*H$_3$). HRMS calcd. for $C_{14}H_{23}O_5$ $[M+H]^+$ 271.1540, found 271.1544. IR v_{max} (thin film, cm^{-1}) = 2954, 1732, 1651, 1451, 1435, 1177.

***Rac*-methyl (3*R*,6*S*)-6-methyl-3-(3-methylbut-3-en-1-yl)-2-oxotetrahydro-2H-pyran-3-carboxylate (S35)**

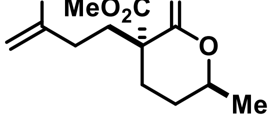

Prepared according to General Procedure D using **S34** (1.0 eq., 3.0 mmol, 811 mg). Purification by silica gel column chromatography (hexane/Et_2O 6:4) yielded the title product as a 6.5:1 mixture of diastereoisomers (402 mg, 1.67 mmol, 56%). ^{1}H NMR (400 MHz, $CDCl_3$, major diasteromer) δ 4.79 – 4.64 (m, 2H, CH_2=C(CH$_3$)CH$_2$), 4.34 (dqd, *J* = 10.0, 6.2, 3.7 Hz, 1H, C*H*CH$_3$), 3.76 (s, 3H, CO$_2$CH_3), 2.52 (dt, *J* = 14.0,

8.0 Hz, 1H, $CH_3CHCH_2CH_aH_b$), 2.16 – 2.07 (m, 1H, $CH_2=C(CH_3)CH_2CH_aH_b$), 2.07 – 1.89 (m, 4H, $CH_2=C(CH_3)CH_2CH_aH_b$, $CH_2=C(CH_3)CH_2$, $CH_3CHCH_aH_b$,), 1.78 (ddd, *J* = 14.0, 8.2, 4.9 Hz, 1H, $CH_3CHCH_2CH_aH_b$), 1.72 (t, *J* = 1.1 Hz, 3H, $CH_2=C(CH_3)$), 1.65 – 1.57 (m, 1H, $CH_3CHCH_aH_b$), 1.35 (d, *J* = 6.2 Hz, 3H, CH_3CH). ^{13}C NMR (101 MHz, $CDCl_3$, major diasteromer) δ 171.9 (CO_2Me), 170.9 (CO_2CH), 144.8 ($CH_2=C(CH_3)CH_2$), 110.5 ($CH_2=C(CH_3)CH_2$), 74.9 (CO_2CH), 53.2 (CO_2CH_3), 52.9 ($C_qCO_2CH_3$), 34.8 ($CH_2=C(CH_3)CH_2CH_2$), 32.5 ($CH_2=C(CH_3)CH_2CH_2$), 27.9 (CH_3CHCH_2), 27.0 ($CH_3CHCH_2CH_2$), 22.7 ($CH_2=C(CH_3)CH_2CH_2$), 21.5 ($CHCH_3$). HRMS calcd. for $C_{13}H_{20}O_4Na$ $[M+Na]^+$ 263.1254, found 263.1260. IR ν_{max} (thin film, cm^{-1}) = 2976, 2953, 2937, 1725, 1649, 1448.

Dimethyl 2-(3-oxobutyl)-2-(4-phenylbut-3-yn-1-yl)malonate (S36)

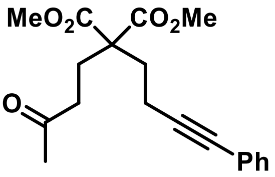

Prepared according to General Procedure F using dimethyl 2-(4-phenylbut-3-yn-1-yl)malonate[12] (1.0 eq., 9.81 mmol, 2.56 g). Purification by silica gel column chromatography (30% EtOAc in hexanes) yielded the title product as a yellow oil (2.63 g, 7.95 mmol, 81%). ^1H NMR (500 MHz, $CDCl_3$) δ 7.41 – 7.33 (m, 2H, Ar*H*), 7.30 – 7.23 (m, 3H, Ar*H*), 3.72 (s, 6H, OCH_3), 2.47 (dd, *J* = 8.8, 6.8 Hz, 2H, CH_2COMe), 2.39 (dd, *J* = 8.9, 7.1 Hz, 2H, $CCCH_2$), 2.27 – 2.18 (m, 4H, $CH_2C_q(CO_2Me)_2CH_2$), 2.12 (s, 3H, CH_2COCH_3). ^{13}C NMR (126 MHz, $CDCl_3$) δ 207.1 (*C*OMe), 171.4 (CO_2Me), 131.6 (Ar*C*H), 128.3 (Ar*C*H), 127.9 (Ar*C*H), 123.6 (ArC_q), 88.6 (ArC_qC*C*), 81.3 (ArC_q*C*C), 56.4 (C_qCO_2Me), 52.7 (OCH_3), 38.8 (CH_2COMe), 32.7 ($CCCH_2CH_2$), 30.0 (CH_2COCH_3), 26.8 ($CH_2CH_2COCH_3$), 15.1 ($CCCH_2$). HRMS calcd. for $C_{19}H_{22}O_5Na$ $[M+Na]^+$ 353.1359, found 353.1350. IR ν_{max} (thin film, cm^{-1}) = 2953, 1720, 1598, 1491, 1434.

***Rac*-methyl (3*R*,6*S*)-6-methyl-2-oxo-3-(4-phenylbut-3-yn-1-yl)tetrahydro-2H-pyran-3-carboxylate (1y)**

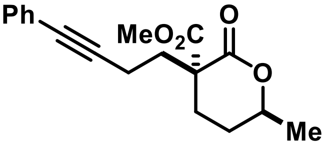

Prepared according to General Procedure D using **S36** (1.0 eq., 2.0 mmol, 661 mg). Purification by silica gel column chromatography (35% EtOAc in hexanes) yielded the title product as a >20:1 mixture of diastereoisomers (331 mg, 1.11 mmol, 55%). ^1H NMR (400 MHz, $CDCl_3$) δ 7.42 – 7.33 (m, 2H, Ar*H*), 7.31 – 7.23 (m, 3H, Ar*H*), 4.35 (dddd, *J* = 12.3, 10.1, 6.2, 3.8 Hz, 1H, C*H*), 3.78 (s, 3H, OCH_3), 2.69 – 2.41 (m, 3H, ArC_qCCCH_2, $CHCH_2CH_aH_b$), 2.39 – 2.26 (m, 1H, $ArC_qCCCH_2CH_aH_b$), 2.17 (ddd, *J* = 13.9, 9.4, 6.4 Hz, 1H, $ArC_qCCCH_2CH_aH_b$), 2.08 – 1.94 (m, 1H, $CHCH_aH_b$), 1.87 (ddd, *J* = 14.0, 8.1, 4.9 Hz, 1H, $CHCH_2CH_aH_b$), 1.66 (ddt, *J* = 14.1, 10.7, 8.0 Hz, 1H, $CHCH_aH_b$), 1.36 (d, *J* = 6.2 Hz, 3H, $CHCH_3$). ^{13}C NMR (101 MHz, $CDCl_3$) δ 171.5 (CO_2Me), 170.6 (CO_2CH), 131.6 (Ar*C*H), 128.3 (Ar*C*H), 127.9 (Ar*C*H), 123.7 (ArC_q), 88.8 (ArC_qC*C*), 81.3(ArC_q*C*C), 75.1 (*C*H), 53.4 (OCH_3), 52.6 (C_q), 35.5 ($ArC_qCCCH_2CH_2$), 27.7 ($CHCH_2$), 27.1 ($CHCH_2CH_2$), 21.5 ($CHCH_3$), 15.4 (ArC_qCCCH_2). HRMS calcd. for $C_{18}H_{20}O_4Na$ $[M+Na]^+$ 323.1254, found 323.1246. IR ν_{max} (thin film, cm^{-1}) = 2978, 2952, 2932, 1725, 1490, 1442.

4. Synthesis and Characterization of 1,4–Ester Migration Products

***Rac*–methyl (*R*)–2–benzyl–4–((3*S*,6*S*)–6–methyl–2–oxotetrahydro–2*H*–pyran–3–yl)butanoate (3a)**

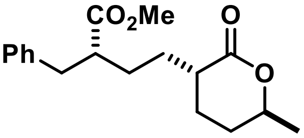

Prepared according to General Procedure E using SmI_2 (2.5 mL, 0.1 M in THF, 0.25 mmol, 2.5 eq.), HMPA (0.17 mL, 1.0 mmol, 10 eq.), H_2O (29 µL, 1.6 mmol, 16 eq.) and *rac*–methyl (3*R*,6*S*)–6–methyl–2–oxo–3–((*E*)–4–phenylbut–3–en–1–yl)tetrahydro–2*H*–pyran–3–carboxylate **1a** (30 mg, 0.10 mmol, 1.0 eq.). Purification by silica gel column chromatography (hexane/EtOAc 85:15) yielded the title product as a colourless oil, as a 3:1 mixture of diastereoisomers (23 mg, 0.076 mmol, 76%). ^{1}H NMR (400 MHz, $CDCl_3$) δ 1.35 (apparent dd, *J* = 6.3, 2.6 Hz, 3H, $CHC\mathit{H_3}$), 1.42–1.65 (m, 4H, $CO_2CHCH_2C\mathit{H_a}H_b$ + $CO_2CHC\mathit{H_a}H_b$ + $C\mathit{H_a}H_bCH_2CHCO_2CH_3$ + $CH_2C\mathit{H_a}H_bCHCO_2CH_3$), 1.68–1.80 (m, 1H, $CH_2CH_a\mathit{H_b}CHCO_2CH_3$), 1.84–2.11 (m, 3H, $CO_2CHCH_a\mathit{H_b}$ + $CH_a\mathit{H_b}CH_2CHCO_2CH_3$ + $CO_2CHCH_2CH_a\mathit{H_b}$), 2.30 (dddd, *J* = 11.1, 8.1, 6.7, 4.5 Hz, 0.75H, $C\mathit{H}CO_2CH$ from major diastereoisomer), 2.41 (ddt, *J* = 10.0, 7.6, 3.7 Hz, 0.25H, $C\mathit{H}CO_2CH$ from minor diastereoisomer), 2.62–2.72 (m, 1H, $C\mathit{H}CH_2Ar$), 2.78 (dd, *J* = 13.5, 6.6 Hz, 1H, $CHC\mathit{H_a}H_bAr$), 2.96 (dd, *J* = 13.5, 7.7 Hz, 1H, $CHCH_a\mathit{H_b}Ar$), 3.61 (s, 3H, $CO_2C\mathit{H_3}$), 4.39 (m, 1H, $CO_2C\mathit{H}$), 7.15 (dd, *J* = 6.9, 1.5 Hz, 2H, $ArC\mathit{H}$), 7.18–7.23 (m, 1H, $ArC\mathit{H}$), 7.24–7.30 (m, 2H, $ArC\mathit{H}$) ppm; ^{13}C NMR (101 MHz, $CDCl_3$) δ 22.1 ($CH\mathit{C}H_3$), 25.6 ($CO_2CHCH_2\mathit{C}H_2$), 29.2 ($CH_2\mathit{C}H_2CHCO_2CH_3$), 29.7 ($\mathit{C}H_2CH_2CHCO_2CH_3$) 30.7 ($CO_2CH\mathit{C}H_2$), 38.5 ($\mathit{C}H_2Ar$), 40.4 ($\mathit{C}HCO_2CH$), 47.4 ($\mathit{C}HCH_2Ar$), 51.5 ($CO_2\mathit{C}H_3$), 77.7 ($CO_2\mathit{C}H$), 126.4 (Ar*C*H), 128.4 (Ar*C*H), 128.8 (Ar*C*H), 139.0 (Ar*C*), 173.4 ($\mathit{C}O_2CH$), 175.7 ($\mathit{C}O_2CH_3$) ppm; IR v_{max} (thin film, cm^{-1}) = 2940, 1733 (C=O), 1435, 1162, 1098; HRMS calcd. for $C_{18}H_{24}O_4Na$ $[M+Na]^+$ 327.1567, found 327.1551.

Note: When the reaction was repeated using tripyrrolidinophosphoric acid triamide (TPPA), a nontoxic alternative to the carcinogenic and mutagenic HMPA, the desired product was isolated in 66% yield with 4:1 d.r.

***Rac*–methyl (1*S*,3*S*)–3–benzyl–1–(3–hydroxybutyl)–2–oxocyclopentane–1–carboxylate (2a)**

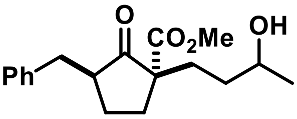

Prepared according to a modified version of General Procedure E using SmI_2 (1.7 mL, 0.1 M in THF, 0.17 mmol, 2.5 eq.), H_2O (0.30 mL, 17 mmol, 250 eq.) and dimethyl (*E*)–2–(3–oxobutyl)–2–(4–phenylbut–3–en–1–yl)malonate **S3** (20 mg, 0.066 mmol, 1.0 eq.). Purification by silica gel column chromatography (hexane/EtOAc 8:2) yielded the title product as a colourless oil (5 mg, 0.016 mmol, 29%). ^{1}H NMR (500 MHz, $CDCl_3$) δ 1.15 (d, *J* = 6.2 Hz, 3H, $CHC\mathit{H_3}$), 1.25–1.44 (m, 2H, $CH_3CH(OH)C\mathit{H_2}$), 1.51–1.64 (m, 2H, $C(O)CHC\mathit{H_a}H_b$ + $C(O)CHCH_2C\mathit{H_a}H_b$), 1.86–1.94 (m, 2H, $C(O)CHCH_a\mathit{H_b}$ + $CH_3CH(OH)CH_2C\mathit{H_a}H_b$), 2.05–2.11 (m, 1H, $CH_3CH(OH)CH_2CH_a\mathit{H_b}$), 2.36 (ddd, *J* = 13.4, 10.3, 7.2 Hz, 1H, $C(O)CHCH_2CH_a\mathit{H_b}$) 2.61–2.71 (m, 2H, $C\mathit{H}CH_2Ar$ + $CHC\mathit{H_a}H_bAr$), 3.13 (dd, *J* = 13.4, 3.8 Hz, 1H, $CHCH_a\mathit{H_b}Ar$), 3.68–3.74 (m, 1H, $C\mathit{H}OH$), 3.71 (s, 3H, $CO_2C\mathit{H_3}$), 7.16–7.18 (m, 2H, $ArC\mathit{H}$), 7.20–7.23 (m, 1H, $ArC\mathit{H}$), 7.27–7.30 (m, 2H, $ArC\mathit{H}$) ppm, O*H* not observed; ^{13}C NMR (126 MHz, $CDCl_3$) δ 23.4 ($CH\mathit{C}H_3$), 25.2 ($CH_3CH(OH)CH_2\mathit{C}H_2$), 29.0 ($C(O)CH\mathit{C}H_2$), 30.7 ($C(O)CHCH_2\mathit{C}H_2$), 34.0 ($CH_3CH(OH)\mathit{C}H_2$), 35.5 ($\mathit{C}H_2Ar$), 50.9 ($C(O)\mathit{C}H$), 52.6 ($CO_2\mathit{C}H_3$), 59.9 ($C_q$), 67.9 (*C*HOH), 126.4 (Ar*C*H), 128.4 (Ar*C*H), 129.2 (Ar*C*H), 139.3 (Ar*C*), 172.1

($\mathit{C}O_2CH_3$), 214.5 (C(O)) ppm; IR ν_{max} (thin film, cm^{-1}) = 2953, 1728 (C=O), 1433, 1222, 1174, 966; HRMS calcd. for $C_{18}H_{25}O_4$ [M+H]$^+$ 305.1747, found 305.1744.

***Rac*–methyl (*R*)–2–benzyl–4–((3*S*,6*S*)–6–ethyl–2–oxotetrahydro–2*H*–pyran–3–yl)butanoate (3b)**

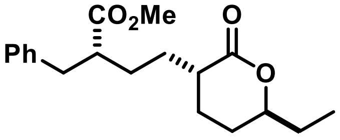

Prepared according to General Procedure E using SmI_2 (2.5 mL, 0.1 M in THF, 0.25 mmol, 2.5 eq.), HMPA (0.17 mL, 1.0 mmol, 10 eq.), H_2O (29 µL, 1.6 mmol, 16 eq.) and *rac*–methyl (3*R*,6*S*)–6–ethyl–2–oxo–3–((*E*)–4–phenylbut–3–en–1–yl)tetrahydro–2*H*–pyran–3–carboxylate **1b** (32 mg, 0.10 mmol, 1.0 eq.). Purification by silica gel column chromatography (hexane/EtOAc 85:15) yielded the title product as a colourless oil, as a 3:1 mixture of diastereoisomers (22 mg, 0.069 mmol, 69%). ^{1}H NMR (500 MHz, $CDCl_3$) δ 0.98 (t, *J* = 7.5 Hz, 3H, $CH_2C\mathit{H}_3$), 1.36–1.79 (m, 7H, $C\mathit{H}_2CHCO_2CH_3$ + $C\mathit{H}_2CH_3$ + $CO_2CHC\mathit{H}_aH_b$ + $CO_2CHCH_2C\mathit{H}_aH_b$ + $C\mathit{H}_aH_bCH_2CHCO_2CH_3$), 1.83–2.08 (m, 3H, $CO_2CHCH_a\mathit{H}_b$ + $CO_2CHCH_2CH_a\mathit{H}_b$ + $CH_a\mathit{H}_bCH_2CHCO_2CH_3$), 2.26–2.34 (m, 0.75H, $C\mathit{H}CO_2CH$ from major diastereoisomer), 2.37–2.46 (m, 0.25H, $C\mathit{H}CO_2CH$ from minor diastereoisomer), 2.64–2.72 (m, 1H, $C\mathit{H}CO_2CH_3$), 2.76 (dd, *J* = 13.5, 6.9 Hz, 1H, $C\mathit{H}_aH_bAr$), 2.96 (dd, *J* = 13.6, 6.9 Hz, 1H, $CH_a\mathit{H}_bAr$), 3.61 (s, 3H, $CO_2C\mathit{H}_3$), 4.14–4.22 (m, 1H, $CO_2C\mathit{H}$), 7.13–7.16 (m, 2H, ArC*H*), 7.18–7.23 (m, 1H, ArC*H*), 7.25–7.30 (m, 2H, ArC*H*) ppm; ^{13}C NMR (126 MHz, $CDCl_3$) δ 9.2 ($CH_2\mathit{C}H_3$), 25.5 ($\mathit{C}H_2CH_2CHCO_2CH_3$), 28.3 ($CO_2CHCH_2\mathit{C}H_2$), 29.1 ($\mathit{C}H_2CH_3$), 29.2 ($\mathit{C}H_2CHCO_2CH_3$), 29.8 ($CO_2CH\mathit{C}H_2$), 38.5 ($\mathit{C}H_2Ar$), 40.8 ($\mathit{C}HCO_2CH$), 47.4 ($\mathit{C}HCO_2CH_3$), 51.5 ($CO_2\mathit{C}H_3$), 82.3 ($CO_2\mathit{C}H$), 126.4 (Ar*C*H), 128.4 (Ar*C*H), 128.8 (Ar*C*H), 139.1 (Ar*C*), 173.5 ($\mathit{C}O_2CH$), 175.8 ($\mathit{C}O_2CH_3$) ppm; IR ν_{max} (thin film, cm^{-1}) = 2942, 1725 (C=O), 1435, 1371, 1246, 1163, 1090; HRMS calcd. for $C_{19}H_{26}O_4Na$ [M+Na]$^+$ 341.1723, found 341.1708.

***Rac*–methyl (*R*)–2–benzyl–4–((3*S*,6*S*)–6–butyl–2–oxotetrahydro–2*H*–pyran–3–yl)butanoate (3c)**

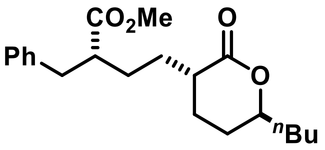

Prepared according to General Procedure E using SmI_2 (2.75 mL, 0.1 M in THF, 0.275 mmol, 2.5 eq.), HMPA (0.19 mL, 1.1 mmol, 10 eq.), H_2O (30 µL, 1.7 mmol, 16 eq.) and *rac*–methyl (3*R*,6*S*)–6–butyl–2–oxo–3–((*E*)–4–phenylbut–3–en–1–yl)tetrahydro–2*H*–pyran–3–carboxylate **1c** (38 mg, 0.11 mmol, 1.0 eq.) in THF (0.5 mL). Purification by silica gel column chromatography (Hexane/EtOAc 9:1) yielded the title product as a yellow oil, as a 1.7:1 mixture of diastereoisomers (24 mg, 0.068 mmol, 62%). ^{1}H NMR (400 MHz, $CDCl_3$) δ 0.90 (t, 3H, *J* = 6.9 Hz, $CH_2C\mathit{H}_3$), 1.29–1.38 (m, 3H, $C\mathit{H}_2CH_3$ + $C(O)CHC\mathit{H}_aH_bCH_2$), 1.38–1.60 (m, 6H, $C(O)CHCH_2C\mathit{H}_2$ + $CH_3O(O)CCHC\mathit{H}_2CH_2$ + $CH_3O(O)CCHCH_2C\mathit{H}_2$), 1.62–1.78 (m, 2H, $C\mathit{H}_2CH_2CH_2CH_3$), 1.82–2.04 (m, 3H, $C\mathit{H}_2CH_2CH_3$ + $C(O)CHCH_a\mathit{H}_bCH_2$) 2.24–2.34 (m, 0.63H, $C\mathit{H}CO_2CH$ from major diastereoisomer), 2.34–2.44 (m, 0.37H, $C\mathit{H}CO_2CH$ from minor diastereoisomer), 2.67 (ddd, 1H, *J* = 12.7, 9.8, 5.8 Hz, $CH_3O_2CC\mathit{H}$), 2.77 (td, 1H, *J* = 13.4, 12.4, 6.5 Hz, $CH_3O_2CCHC\mathit{H}_aH_b$), 2.94 (td, 1H, *J* = 13.7, 7.0 Hz, $CH_3O(O)CCHCH_a\mathit{H}_b$), 3.60 (s, 3H, $CO_2C\mathit{H}_3$), 4.22 (m, 1H, $CO_2C\mathit{H}$), 7.14 (d, 2H, *J* = 7.0 Hz, ArC*H*), 7.20 (t, 1H, *J* = 7.3 Hz, ArC*H*), 7.27–7.30 (m, 2H, ArC*H*) ppm; ^{13}C NMR (101 MHz, $CDCl_3$) δ 14.0 ($CH_2\mathit{C}H_3$), 22.5 ($\mathit{C}H_2CH_3$), 25.6 ($CH_3O(O)CCH\mathit{C}H_2CH_2$), 26.9 ($CH_2\mathit{C}H_2CH_2CH_3$), 28.8 ($C(O)CH\mathit{C}H_2CH_2$), 29.2 ($CH_3O(O)CCHCH_2\mathit{C}H_2$), 29.8 (($C(O)CHCH_2\mathit{C}H_2$), 36.0 ($\mathit{C}H_2CH_2CH_2CH_3$), 38.5 ($\mathit{C}HCH_2Ar$), 40.8

(CHCO$_2$*C*H), 47.5 (C*H*CO$_2$CH$_3$), 51.6 (CO$_2$*C*H$_3$), 81.4 (C*H*CO$_2$*C*H), 126.4 (Ar*C*H), 128.4 (Ar*C*H), 128.8 (Ar*C*H), 139.0 (Ar*C*), 173.5 (*C*O$_2$CH), 175.8 (*C*O$_2$CH$_3$) ppm; IR $\nu_{\max}$ (thin film, cm^{-1}) = 2952, 2862, 1732, 1495, 1455, 1377, 1248, 1164, 1105, 745; HRMS calculated for $C_{21}H_{30}O_4Na$ [M+Na]$^+$ 369.2036, found 369.2023.

***Rac*–methyl (*R*)–2–benzyl–4–((3*S*,6*R*)–6–benzyl–2–oxotetrahydro–2*H*–pyran–3–yl)butanoate (3d)**

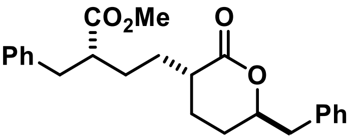

Prepared according to General Procedure E using SmI$_2$ (2.5 mL, 0.1 M in THF, 0.25 mmol, 2.5 eq.), HMPA (0.17 mL, 1.0 mmol, 10 eq.), H$_2$O (29 µL, 1.6 mmol, 16 eq.) and *rac*–methyl (3*R*,6*R*)–6–benzyl–2–oxo–3–((*E*)–4–phenylbut–3–en–1–yl)tetrahydro–2*H*–pyran–3–carboxylate **1d** (38 mg, 0.10 mmol, 1.0 eq.). Purification by silica gel column chromatography (hexane/EtOAc 85:15) yielded the title product as a colourless oil, as a 1:1 mixture of diastereoisomers (25 mg, 0.066 mmol, 66%). ^{1}H NMR (500 MHz, CDCl$_3$) δ 1.34–1.77 (m, 5H, C*H*$_2$CHCO$_2$CH$_3$ + C*H$_a$*H$_b$CH$_2$CHCO$_2$CH$_3$ + CO$_2$CHC*H$_a$*H$_b$ + CO$_2$CHCH$_2$C*H$_a$*H$_b$), 1.81–2.04 (m, 3H, CH$_a$*H$_b$*CH$_2$CHCO$_2$CH$_3$ + CO$_2$CHCH$_a$*H$_b$* + CO$_2$CHCH$_2$CH$_a$*H$_b$*), 2.23–2.31 (m, 0.5H, C*H*CO$_2$CH from one diastereoisomer), 2.34–2.44 (m, 0.5H, C*H*CO$_2$CH from one diastereoisomer), 2.63–2.70 (m, 1H, C*H*CO$_2$CH$_3$), 2.77 (apparent td, *J* = 13.3, 8.6 Hz, 1H, CH(CO$_2$CH$_3$)C*H$_a$*H$_b$Ar), 2.85 (apparent td, *J* = 14.1, 7.2 Hz, 1H, CO$_2$CHC*H$_a$*H$_b$Ar), 2.95 (apparent dt, *J* = 13.4, 8.9 Hz, 1H, CH(CO$_2$CH$_3$)CH$_a$*H$_b$*Ar), 3.08 (apparent dt, *J* = 13.4, 6.5 Hz, 1H, CO$_2$CHCH$_a$*H$_b$*Ar), 3.60 (s, 3H, CO$_2$C*H$_3$*), 4.40 (m, 1H, CO$_2$C*H*), 7.14 (d, *J* = 7.6 Hz, 2H, ArC*H*), 7.18–7.27 (m, 5H, ArC*H*), 7.27–7.34 (m, 3H, ArC*H*) ppm; ^{13}C NMR (126 MHz, CDCl$_3$) δ 25.4 (*C*H$_2$CH$_2$CHCO$_2$CH$_3$ from one diastereoisomer), 26.1 (*C*H$_2$CH$_2$CHCO$_2$CH$_3$ from one diastereoisomer), 28.0 (CO$_2$CH*C*H$_2$ from one diastereoisomer), 28.3 (CO$_2$CH*C*H$_2$ from one diastereoisomer), 29.2 (*C*H$_2$CHCO$_2$CH$_3$), 29.7 (CO$_2$CHCH$_2$*C*H$_2$), 38.0 (*C*HCO$_2$CH from one diastereoisomer), 38.3 (CH(CO$_2$CH$_3$)*C*H$_2$Ar from one diastereoisomer), 38.5 (CH(CO$_2$CH$_3$)*C*H$_2$Ar from one diastereoisomer) 40.7 (*C*HCO$_2$CH from one diastereoisomer), 41.6 (CO$_2$CH*C*H$_2$Ar from one diastereoisomer), 42.5 (CO$_2$CH*C*H$_2$Ar from one diastereoisomer), 47.4 (*C*HCO$_2$CH$_3$ from one diastereoisomer), 47.5 (*C*HCO$_2$CH$_3$ from one diastereoisomer), 51.5 (CO$_2$*C*H$_3$), 78.5 (CO$_2$*C*H from one diastereoisomer), 81.6 (CO$_2$*C*H from one diastereoisomer), 126.4 (Ar*C*H from one diastereoisomer), 126.4 (Ar*C*H from one diastereoisomer), 126.8 (Ar*C*H from one diastereoisomer), 126.8 (Ar*C*H from one diastereoisomer), 128.4 (Ar*C*H from one diastereoisomer), 128.4 (Ar*C*H from one diastereoisomer), 128.5 (Ar*C*H from one diastereoisomer), 128.6 (Ar*C*H from one diastereoisomer), 128.8 (Ar*C*H), 129.5 (Ar*C*H from one diastereoisomer), 129.6 (Ar*C*H from one diastereoisomer), 136.3 (Ar*C* from one diastereoisomer), 136.6 (Ar*C* from one diastereoisomer), 139.0 (Ar*C* from one diastereoisomer), 139.1 (Ar*C* from one diastereoisomer), 173.2 (*C*O$_2$CH from one diastereoisomer), 174.8 (*C*O$_2$CH from one diastereoisomer), 175.7 (*C*O$_2$CH$_3$ from one diastereoisomer), 175.7 (*C*O$_2$CH$_3$ from one diastereoisomer) ppm; IR ν_{max} (thin film, cm^{-1}) = 2922, 1729 (C=O), 1454, 1375, 1251, 1164, 1080; HRMS calcd. for $C_{24}H_{28}O_4Na$ [M+Na]$^+$ 403.1880, found 403.1866.

***Rac*–methyl (*R*)–2–benzyl–4–((3*S*,6*R*)–6–neopentyl–2–oxotetrahydro–2*H*–pyran–3–yl)butanoate (3e)**

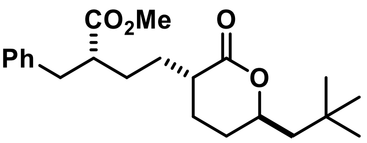

Prepared according to General Procedure E using SmI_2 (2.5 mL, 0.1 M in THF, 0.25 mmol, 2.5 eq.), HMPA (0.17 mL, 1.0 mmol, 10 eq.), H_2O (29 µL, 1.6 mmol, 16 eq.) and *rac*–methyl (3*R*,6*R*)–6–neopentyl–2–oxo–3–((*E*)–4–phenylbut–3–en–1–yl)tetrahydro–2*H*–pyran–3–carboxylate **1e** (36 mg, 0.10 mmol, 1.0 eq.). Purification by silica gel column chromatography (hexane/EtOAc 9:1) yielded the title product as a colourless oil, as a 1.5:1 mixture of diastereoisomers (23 mg, 0.064 mmol, 64%). ^{1}H NMR (500 MHz, $CDCl_3$) δ 0.97 (s, 9H, $C(C\textit{H}_3)_3$), 1.32–1.79 (m, 7H, $C\textit{H}_2C(CH_3)_3$ + $CO_2CHC\textit{H}_a H_b$ + $CO_2CHCH_2C\textit{H}_a H_b$ + $C\textit{H}_2CH_2CHCO_2CH_3$ + $CH_2C\textit{H}_a H_bCHCO_2CH_3$), 1.81–1.92 (m, 1.4H $CO_2CHCH_a\textit{H}_b$ + $CH_2CH_a\textit{H}_bCHCO_2CH_3$ from minor diastereoisomer), 1.93–2.10 (m, 1.6H, $CO_2CHCH_2CH_a\textit{H}_b$ + $CH_2CH_a\textit{H}_bCHCO_2CH_3$ from major diastereoisomer), 2.24–2.32 (m, 0.4H, $C\textit{H}CO_2CH$ from minor diastereoisomer), 2.39–2.46 (m, 0.6H, $C\textit{H}CO_2CH$ from major diastereoisomer), 2.63–2.72 (m, 1H, $C\textit{H}CH_2Ar$), 2.78 (dd, *J* = 13.6, 6.7 Hz, 1H, $C\textit{H}_a H_bAr$), 2.96 (apparent dt, *J* = 13.6, 8.0 Hz, 1H, $CH_a\textit{H}_bAr$), 3.61 (s, 3H, $CO_2C\textit{H}_3$), 4.33–4.39 (m, 1H, $CO_2C\textit{H}$), 7.15 (dd, *J* = 7.2, 2.6 Hz, 2H, ArC*H*), 7.20 (m, 1H, ArC*H*), 7.25–7.31 (m, 2H, ArC*H*) ppm; ^{13}C NMR (126 MHz, $CDCl_3$) δ 25.7 ($CO_2CHCH_2\textit{C}H_2$), 28.3 ($CO_2CH\textit{C}H_2$ from one diastereoisomer), 28.5 ($CO_2CH\textit{C}H_2$ from one diastereoisomer), 29.2 ($CH_2\textit{C}H_2CHCO_2CH_3$), 29.9 ($C(\textit{C}H_3)_3$), 30.1 ($CH_2\textit{C}H_2CHCO_2CH_3$ from one diastereoisomer), 30.1 ($\textit{C}H_2CH_2CHCO_2CH_3$ from one diastereoisomer), 30.7 ($\textit{C}H_2CH_2CHCO_2CH_3$ from one diastereoisomer), 37.8 ($\textit{C}(CH_3)_3$), 38.2 ($\textit{C}H_2Ar$ from one diastereoisomer), 38.5 ($\textit{C}H_2Ar$ from one diastereoisomer), 40.4, ($\textit{C}HCO_2CH$), 47.4 ($\textit{C}HCH_2Ar$ from one diastereoisomer), 47.6 ($\textit{C}HCH_2Ar$ from one diastereoisomer), 49.0 ($\textit{C}H_2C(CH_3)_3$ from one diastereoisomer), 50.1 ($\textit{C}H_2C(CH_3)_3$ from one diastereoisomer), 51.5 ($CO_2\textit{C}H_3$ from one diastereoisomer), 51.6 ($CO_2\textit{C}H_3$ from one diastereoisomer), 75.4 ($CO_2\textit{C}H$ from one diastereoisomer), 79.1 ($CO_2\textit{C}H$ from one diastereoisomer), 126.4 (Ar*C*H from one diastereoisomer), 126.4 (Ar*C*H from one diastereoisomer), 128.4 (Ar*C*H from one diastereoisomer), 128.4 (Ar*C*H from one diastereoisomer), 128.4 (Ar*C*H from one diastereoisomer), 128.5 (Ar*C*H from one diastereoisomer), 139.0 (Ar*C* from one diastereoisomer), 139.1 (Ar*C* from one diastereoisomer), 173.3 ($\textit{C}O_2CH$ from one diastereoisomer), 175.2 ($\textit{C}O_2CH$ from one diastereoisomer), 175.7 ($\textit{C}O_2CH_3$ from one diastereoisomer), 175.8 ($\textit{C}O_2CH_3$ from one diastereoisomer) ppm; IR ν_{max} (thin film, cm^{-1}) = 2949, 1728 (C=O), 1454, 1364, 1247, 1162, 1075; HRMS calcd. for $C_{22}H_{32}O_4Na$ $[M+Na]^+$ 383.2193, found 383.2180.

***Rac*–methyl (*R*)–2–benzyl–4–((3*S*,6*R*)–6–isopropyl–2–oxotetrahydro–2*H*–pyran–3–yl)butanoate (3f)**

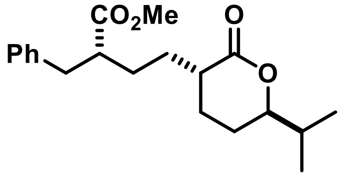

Prepared according to General Procedure E using SmI_2 (2.5 mL, 0.1 M in THF, 0.25 mmol, 2.5 eq.), HMPA (0.17 mL, 1.0 mmol, 10 eq.), H_2O (29 µL, 1.6 mmol, 16 eq.) and *rac*–methyl (3*R*,6*R*)–6–isopropyl–2–oxo–3–((*E*)–4–phenylbut–3–en–1–yl)tetrahydro–2*H*–pyran–3–carboxylate **1f** (33 mg, 0.10 mmol, 1.0 eq.). Purification by silica gel column chromatography (hexane/EtOAc 85:15) yielded the title product as a colourless oil, as a 3:1 mixture of diastereoisomers (20 mg, 0.061 mmol, 61%). ^{1}H NMR (500 MHz, $CDCl_3$) δ 0.95 (d, *J* = 6.9 Hz, 3H, $CHC\textit{H}_3$), 0.98 (d, *J* = 6.8 Hz, 3H, $CHC\textit{H}_3$), 1.41–1.67 (m, 4H, $CO_2CHC\textit{H}_2$ + $CO_2CHCH_2C\textit{H}_a H_b$ + $CH_2C\textit{H}_a H_bCHCO_2CH_3$), 1.69–1.78 (m, 1H, $CH_2CH_a\textit{H}_bCHCO_2CH_3$), 1.82–1.90 (m, 2H, $CH_a\textit{H}_bCH_2CHCO_2CH_3$ + $CO_2CHCH_2CH_a\textit{H}_b$), 1.95–2.08 (m, 2H, $C\textit{H}_a H_bCH_2CHCO_2CH_3$ + $C\textit{H}(CH_3)_2$), 2.28 (dddd, *J* = 11.1, 8.2, 6.6, 4.5 Hz, 0.75H, $C\textit{H}CO_2CH$ from major diastereoisomer), 2.37–2.47 (m, 0.25H, $C\textit{H}CO_2CH$ from minor diastereoisomer), 2.65–2.71 (m, 1H, $C\textit{H}CH_2Ar$), 2.77 (dd, *J* = 13.6, 6.9 Hz, 1H, $C\textit{H}_a H_bAr$), 2.96 (dd, *J* = 13.6, 8.1 Hz, 1H, $CH_a\textit{H}_bAr$), 3.61 (s, 3H, $CO_2C\textit{H}_3$), 3.98 (ddd, *J* = 11.4, 6.2, 3.3 Hz, 0.25H, $CO_2C\textit{H}$ from minor diastereoisomer), 4.03 (ddd, *J* = 11.5, 5.6, 3.1 Hz, 0.75H, $CO_2C\textit{H}$ from major

diastereoisomer), 7.12–7.17 (m, 2H, ArC*H*), 7.18–7.23 (m, 1H, ArC*H*), 7.25–7.31 (m, 2H, ArC*H*) ppm; ^{13}C NMR (126 MHz, $CDCl_3$) δ 17.6 ($CH\mathit{C}H_3$), 17.7 ($CH\mathit{C}H_3$), 25.4 ($CO_2CHCH_2\mathit{C}H_2$), 25.6 ($\mathit{C}H_2CH_2CHCO_2CH_3$) 29.2 ($CH_2\mathit{C}H_2CHCO_2CH_3$), 29.8 ($CO_2CH\mathit{C}H_2$), 32.9 ($\mathit{C}H(CH_3)_2$), 38.5 ($\mathit{C}H_2Ar$), 40.9 ($\mathit{C}HCO_2CH$), 47.4 ($\mathit{C}HCH_2Ar$), 51.5 ($CO_2\mathit{C}H_3$), 86.0 ($CO_2\mathit{C}H$), 126.4 (Ar*C*H), 128.4 (Ar*C*H), 128.9 (Ar*C*H), 139.1 (Ar*C*), 173.6 ($\mathit{C}O_2CH$), 175.8 ($\mathit{C}O_2CH_3$) ppm; IR ν_{max} (thin film, cm^{-1}) = 2955, 2924, 1731 (C=O), 1453, 1245, 700; HRMS calcd. for $C_{20}H_{28}O_4Na$ $[M+Na]^+$ 355.1880, found 355.1868**.**

***Rac*–methyl (*R*)–4–((3*S*,6*R*)–6–benzhydryl–2–oxotetrahydro–2*H*–pyran–3–yl)–2–benzylbutanoate (3g)**

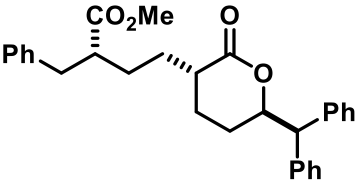

Prepared according to General Procedure E using SmI_2 (2.5 mL, 0.1 M in THF, 0.25 mmol, 2.5 eq.), HMPA (0.17 mL, 1.0 mmol, 10 eq.), H_2O (29 µL, 1.6 mmol, 16 eq.) and *rac*–methyl (3*R*,6*R*)–6–benzhydryl–2–oxo–3–((*E*)–4–phenylbut–3–en–1–yl)tetrahydro–2*H*–pyran–3–carboxylate **1g** (45 mg, 0.1 mmol, 1.0 eq.) in THF (0.5 mL). Purification by silica gel column chromatography (Hexane/EtOAc 9.8:0.2 to 9.5:0.5) yielded the title product as a yellow oil, as a 1.6:1 mixture of diastereoisomers (11 mg, 0.029 mmol, 24%). ^{1}H NMR (400 MHz, $CDCl_3$) δ 1.44–1.64 (m, 3H, $C(O)CHC\mathit{H_a}H_bCH_2$ + $CH_3O(O)CCHC\mathit{H_a}H_bCH_2$ + $CH_3O_2CCHCH_2C\mathit{H_a}H_b$), 1.66–1.75 (m, 1H, $C(O)CHCH_2C\mathit{H_a}H_b$), 1.75–1.87 (m, 2H, $C(O)CHCH_2CH_a\mathit{H_b}$ + $CH_3O_2CCHCH_a\mathit{H_b}CH_2$), 1.89–2.07 (m, 2H, $C(O)CHCH_a\mathit{H_b}CH_2$ + $CH_3O_2CCHCH_2CH_a\mathit{H_b}$), 2.24 (ddt, 0.47H, *J* = 25.0, 9.2, 4.9 Hz, $C\mathit{H}CO_2CH$ from minor diastereoisomer), 2.47 (dq, 0.52H, *J* = 16.7, 9.8, 8.5 Hz, $C\mathit{H}CO_2CH$ from major diastereoisomer), 2.66 (tt, 1H, *J* = 8.3, 5.4 Hz, $C\mathit{H}CO_2CH_3$), 2.72–2.81 (m, 1H, CH_aH_bAr), 2.93 (dt, 1H, *J* = 13.6, 8.1 Hz, CH_aH_bAr), 3.6 (s, 3H, $OC\mathit{H}_3$), 4.02–4.07 (m, 1H, $C\mathit{H}(Ph)_2$), 4.98 (ddd, 1H, *J* = 11.2, 8.1, 3.3 Hz, $CO_2C\mathit{H}CH$), 7.13 (dt, 2H, *J* = 6.8, 2.5 Hz, ArC*H*), 7.17–7.25 (m, 4H, ArC*H*), 7.30 (dt, 9H, *J* = 12.5, 3.3 Hz, ArC*H*) ppm; ^{13}C NMR (101 MHz, $CDCl_3$) δ 23.3 ($C(O)CH\mathit{C}H_2CH_2$), 25.4 ($CH_3O(O)CCH\mathit{C}H_2CH_2$ from one diastereomer), 25.8 ($CH_3O(O)CCH\mathit{C}H_2CH_2$ from one diastereomer), 27.9 ($CH_3O(O)CCHCH_2\mathit{C}H_2$ from one diastereomer), 28.3 ($CH_3O(O)CCHCH_2\mathit{C}H_2$ from one diastereomer), 29.1 ($C(O)CHCH_2\mathit{C}H_2$ from one diastereomer), 29.8 ($C(O)CHCH_2\mathit{C}H_2$ from one diastereomer), 37.9 ($C\mathit{H}CO_2$), 38.2 ($CH\mathit{C}H_2Ar$ from one diastereomer), 38.5 ($CH\mathit{C}H_2Ar$ from one diastereomer), 47.4 ($\mathit{C}HCO_2CH_3$ from one diastereomer) 47.5 ($\mathit{C}HCO_2CH_3$ from one diastereomer), 51.5 ($CO_2\mathit{C}H_3$), 56.5 ($\mathit{C}HCHCO_2$ from one diastereomer), 57.2 ($\mathit{C}HCHCO_2$ from one diastereomer), 79.0 ($CO_2\mathit{C}H$ one diastereomer), 82.0 ($CO_2\mathit{C}H$ one diastereomer), 126.4 (Ar*C*H from one diastereomer), 126.4 (Ar*C*H from one diastereomer), 126.8 (Ar*C*H), 128.8 (Ar*C*H from one diastereomer), 127.0 (Ar*C*H from one diastereomer), 128.4 (Ar*C*H from one diastereomer), 128.4 (Ar*C*H from one diastereomer), 128.5 (Ar*C*H from one diastereomer), 128.5 (Ar*C*H from one diastereomer), 128.7 (Ar*C*H from one diastereomer), 128.7 (Ar*C*H from one diastereomer), 128.8 (Ar*C*H), 128.8 (Ar*C*H), 128.9 (Ar*C*H), 139.0 (Ar*C* from one diastereomer), 139.1 (Ar*C* from one diastereomer), 140.1 (Ar*C* from one diastereomer), 140.3 (Ar*C* from one diastereomer), 141.0 (Ar*C* from one diastereomer), 141.2 (Ar*C* from one diastereomer), 174.5 ($\mathit{C}O_2CH$ from one diastereomer), 174.6 ($\mathit{C}O_2CH$ from one diastereomer), 175.6 ($\mathit{C}O_2CH_3$ from one diastereomer), 175.7 ($\mathit{C}O_2CH_3$ from one diastereomer) ppm; IR ν_{max} (thin film, cm^{-1}) = 3027, 2925, 1732, 1599, 1495, 1452, 1376, 1166, 1087, 1031, 746, 701; HRMS calculated for $C_{30}H_{32}O_4Na$ $[M+Na]^+$ 479.2193, found 479.2173.

***Rac*–methyl (*R*)–2–benzyl–4–((3*S*,6*R*)–6–cyclohexyl–2–oxotetrahydro–2*H*–pyran–3–yl)butanoate (3h)**

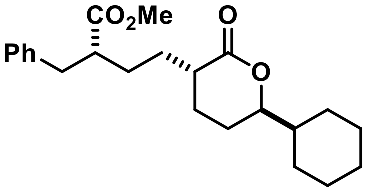

Prepared according to General Procedure E using SmI_2 (2.5 mL, 0.1 M in THF, 0.25 mmol, 2.5 eq.), HMPA (0.17 mL, 1.0 mmol, 10 eq.), H_2O (29 µL, 1.6 mmol, 16 eq.) and *rac*–methyl (3*R*,6*R*)–6–cyclohexyl–2–oxo–3–((*E*)–4–phenylbut–3–en–1–yl)tetrahydro–2*H*–pyran–3–carboxylate **1h** (37 mg, 0.10 mmol, 1.0 eq.). Purification by silica gel column chromatography (hexane/EtOAc 9:1) yielded the title product as a colourless oil, as a 3:1 mixture of diastereoisomers (27 mg, 0.072 mmol, 72%). ^{1}H NMR (500 MHz, $CDCl_3$) δ 1.00–1.29 (m, 6H, 3 x CyCH_2), 1.39–1.61 (m, 5H, CH_aH$_b$CHCO$_2$CH$_3$ + CO$_2$CHCH$_2$CH_aH$_b$ + CyC*H* + CyCH_2), 1.64–1.81 (m, 5H, CO$_2$CHCH_aH$_b$ + CH_aH$_b$CH$_2$CHCO$_2$CH$_3$ + CH$_a$$H_b$CHCO$_2CH_3$ + 0.5 x CyCH_2), 1.82–1.93 (m, 2H, CO$_2$CHCH$_a$$H_b$ + 0.5 x CyCH_2), 1.94–2.06 (m, 2H, CO$_2$CHCH$_2$CH$_a$$H_b$ + CH$_a$$H_bCH_2$CHCO$_2CH_3$), 2.22–2.32 (m, 0.75H, C*H*CO$_2$CH from major diastereoisomer), 2.37–2.45 (m, 0.25H, C*H*CO$_2$CH from minor diastereoisomer), 2.64–2.71 (m, 1H, C*H*CO$_2$CH$_3$), 2.76 (dd, *J* = 13.6, 6.8 Hz, 1H, CH_aH$_b$Ar), 2.96 (dd, *J* = 13.6, 8.0 Hz, 1H, CH$_a$$H_b$Ar), 3.61 (s, 3H, CO$_2CH_3$), 3.96–4.01 (m, 1H, CO$_2$C*H*), 7.15 (d, *J* = 7.5 Hz, 2H, ArC*H*), 7.20 (dd, *J* = 8.6, 5.9 Hz, 1H, ArC*H*), 7.25–7.29 (m, 2H, ArC*H*) ppm; ^{13}C NMR (126 MHz, $CDCl_3$) δ 25.6 (Cy*C*H$_2$), 25.6 (*C*H$_2$CH$_2$CHCO$_2$CH$_3$), 25.9 (Cy*C*H$_2$), 26.0 (Cy*C*H$_2$), 26.3 (Cy*C*H$_2$), 28.0 (Cy*C*H$_2$), 28.1 (CO$_2$CH*C*H$_2$), 29.2 (*C*H$_2$CHCO$_2$CH$_3$), 29.8 (CO$_2$CHCH$_2$*C*H$_2$), 38.5 (*C*H$_2$Ar), 41.0 (*C*HCO$_2$CH), 42.8 (Cy*C*H), 47.4 (*C*HCO$_2$CH$_3$), 51.5 (CO$_2$*C*H$_3$), 85.5 (CO$_2$*C*H), 126.4 (Ar*C*H), 128.4 (Ar*C*H), 128.8 (Ar*C*H), 139.0 (Ar*C*), 173.6 (*C*O$_2$CH), 175.7 (*C*O$_2$CH$_3$) ppm; IR ν_{max} (thin film, cm^{-1}) = 2925, 2853, 1731 (C=O), 1451, 1164; HRMS calcd. for $C_{23}H_{32}O_4Na$ [M+Na]$^+$ 395.2193, found 395.2186.

***Rac*–Methyl (*R*)–2–benzyl–4–((2*R*,5*S*)–6–oxooctahydro–2*H*,2'*H*–[2,4'–bipyran]–5–yl)butanoate (3i)**

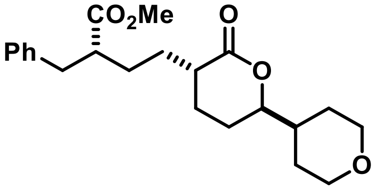

Prepared according to General Procedure E using SmI_2 (2.5 mL, 0.1 M in THF, 0.25 mmol, 2.5 eq.), HMPA (0.17 mL, 1.0 mmol, 10 eq.), H_2O (29 µL, 1.6 mmol, 16 eq.) and *rac*–methyl (2*R*,5*R*)–6–oxo–5–((*E*)–4–phenylbut–3–en–1–yl)octahydro–2*H*,2'*H*–[2,4'–bipyran]–5–carboxylate **1i** (37 mg, 0.1 mmol, 1.0 eq.) in THF (0.5 mL). Purification by silica gel column chromatography (Hexane/EtOAc 8:2) yielded the title product as a yellow oil, as a 1.6:1 mixture of diastereoisomers (18 mg, 0.048 mmol, 48%). ^{1}H NMR (400 MHz, $CDCl_3$) δ 1.38–1.61 (m, 7H, CH_2OCH$_2$ + CH$_2$OCH_2 + CH$_3$O(O)CCHCH_2CH$_2$ + CO$_2$CHC*H*), 1.67–2.04 (m, 6H, CH$_3$O(O)CCHCH$_2$CH_2 + CO$_2$CHCH_2CH$_2$ + CO$_2$CHCH$_2$CH_2), 2.28 (ddd, 0.6H, *J* = 11.0, 7.9, 5.2 Hz, C*H*CO$_2$CH from major diastereoisomer), 2.34–2.47 (m, 0.4H, C*H*CO$_2$CH from minor diastereoisomer), 2.67 (dq, 1H, *J* = 8.2, 4.2 Hz, C*H*CO$_2$CH$_3$), 2.77 (td, 1H, *J* = 13.6, 6.6 Hz, CH_aH$_b$Ar), 2.94 (td, 1H, *J* = 13.8, 7.8 Hz, CH$_a$$H_b$Ar), 3.36 (tdd, 2H, *J* = 11.9, 4.2, 2.3 Hz, CH_2OCH$_2$), 3.60 (s, 3H, OCH_3), 3.97–4.04 (m, 3H, CH$_2$OCH_2 + CO$_2$C*H*), 7.14 (d, 2H, *J* = 7.1 Hz, ArC*H*), 7.17–7.23 (m, 1H, ArC*H*), 7.28 (d, 2H, *J* = 7.1 Hz, ArC*H*) ppm; ^{13}C NMR (101 MHz, $CDCl_3$) δ 25.4 (CO$_2$CH*C*H$_2$), 25.6 (O(CH$_2$)$_2$(*C*H$_2$)$_2$), 27.7 (O(CH$_2$)$_2$(*C*H$_2$)$_2$), 28.6 (ArCH$_2$CH*C*H$_2$CH$_2$), 29.2 (C(O)CH*C*H$_2$CH$_2$), 29.7 (CO$_2$CH*C*H$_2$), 38.5 (CH*C*H$_2$Ar), 40.4 (CO$_2$*C*H from major diastereomer), 40.4 (CO$_2$*C*H from minor diastereomer), 41.0 (CH*C*HCH$_2$CH$_2$), 47.4 (*C*HCO$_2$CH$_3$), 51.6 (CO$_2$*C*H$_3$), 67.5 (*C*H$_2$OCH$_2$), 67.7 (CH$_2$O*C*H$_2$), 84.5 (CO$_2$*C*H),

126.4 (Ar*C*), 128.4 (Ar*C*H), 128.8 (Ar*C*H), 139.0 (Ar*C*), 173.2 (*C*O_2CH), 175.7 (*C*O_2CH_3) ppm; IR ν_{max} (thin film, cm^{-1}) = 2948, 2853, 1729, 1495, 1454, 1373, 1240, 1192, 1165, 1091, 1019, 984, 874, 747, 701; HRMS calculated for $C_{22}H_{30}O_5Na$ $[M+Na]^+$ 397.1985, found 397.1973.

Rac–methyl (_R_)–2–benzyl–4–((3_S_,6_R_)–6–(tert–butyl)–2–oxotetrahydro–2_H_–pyran–3–yl)butanoate (3j)

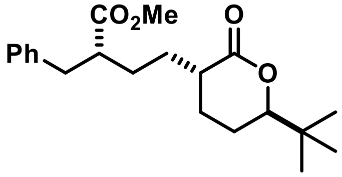

Prepared according to General Procedure E using SmI_2 (2.5 mL, 0.1 M in THF, 0.25 mmol, 2.5 eq.), HMPA (0.17 mL, 1.0 mmol, 10 eq.), H_2O (29 µL, 1.6 mmol, 16 eq.) and *rac*–methyl (3*R*,6*R*)–6–(tert–butyl)–2–oxo–3–((*E*)–4–phenylbut–3–en–1–yl)tetrahydro–2*H*–pyran–3–carboxylate **1j** (34 mg, 0.10 mmol, 1.0 eq.). Purification by silica gel column chromatography (hexane/EtOAc 9:1) yielded the title product as a colourless oil, as a 1.8:1 mixture of diastereoisomers (25 mg, 0.073 mmol, 73%). ^1H NMR (500 MHz, $CDCl_3$) δ 0.95 (s, 9H, C(C*H*$_3$)$_3$), 1.38–1.62 (m, 4H, CO_2CHCH_2C*H*$_a$H$_b$ + CO_2CHC*H*$_a$H$_b$ + C*H*$_a$H$_b$$CH_2CHCO_2CH_3$ + CH_2C*H*$_a$H$_b$$CHCO_2CH_3$), 1.68–1.77 (m, 1H, CH_2CH_a*H*$_b$$CHCO_2CH_3$), 1.86–1.93 (m, 1H, CO_2CHCH_a*H*$_b$), 1.96–2.02 (m, 2H, $CO_2CHCH_2CH_a$*H*$_b$ + CH_a*H*$_b$$CH_2CHCO_2CH_3$), 2.22–2.31 (m, 1H, C*H*CO_2CH), 2.68 (m, 1H, C*H*CH_2Ar), 2.76 (dd, *J* = 13.6, 7.0 Hz, 1H, C*H*$_a$H$_b$Ar), 2.97 (dd, *J* = 13.5, 8.0 Hz, 1H, CH_a*H*$_b$Ar), 3.61 (s, 3H, CO_2C*H*$_3$), 3.91 (dd, *J* = 11.6, 3.1 Hz, 1H, CO_2C*H*), 7.13–7.17 (m, 2H, ArC*H*), 7.21 (t, *J* = 7.4 Hz, 1H, ArC*H*), 7.26–7.31 (m, 2H, ArC*H*) ppm; ^{13}C NMR (126 MHz, $CDCl_3$) δ 23.6 (CO_2CH*C*H_2), 25.4 (C(*C*H_3)$_3$), 25.6 (CO_2CHCH_2*C*H_2), 29.2 (CH_2*C*$H_2CHCO_2CH_3$), 29.8 (*C*$H_2CH_2CHCO_2CH_3$), 34.5 (*C*(CH_3)$_3$), 38.5 (*C*H_2Ar), 40.9 (*C*HCO_2CH), 47.4 (*C*HCH_2Ar), 51.5 (CO_2*C*H_3 from one diastereoisomer), 51.6 (CO_2*C*H_3 from one diastereoisomer), 88.7 (CO_2*C*H from one diastereoisomer), 88.8 (CO_2*C*H from one diastereoisomer), 126.4 (Ar*C*H), 128.4 (Ar*C*H), 128.9 (Ar*C*H), 139.1 (Ar*C*), 173.7 (*C*O_2CH), 175.8 (*C*O_2CH_3) ppm; IR ν_{max} (thin film, cm^{-1}) = 2954, 2871, 1731 (C=O), 1454, 1251, 1174, 1090; HRMS calcd. for $C_{21}H_{30}O_4Na$ $[M+Na]^+$ 369.2036, found 369.2028.

Rac–methyl (_R_)–4–((3_S_,6_S_)–6–ethyl–2–oxotetrahydro–2_H_–pyran–3–yl)–2–(2–fluorobenzyl) butanoate (3k)

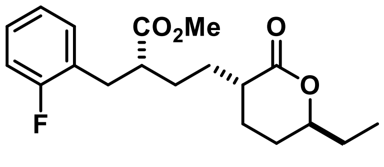

Prepared according to General Procedure E using SmI_2 (2.5 mL, 0.1 M in THF, 0.25 mmol, 2.5 eq.), HMPA (0.17 mL, 1.0 mmol, 10 eq.), H_2O (29 µL, 1.6 mmol, 16 eq.) and *rac*–methyl (3*R*,6*S*)–6–ethyl–3–((*E*)–4–(2–fluorophenyl)but–3–en–1–yl)–2–oxotetrahydro–2*H*–pyran–3–carboxylate **1k** (33 mg, 0.10 mmol, 1.0 eq.). Purification by silica gel column chromatography (hexane/EtOAc 9:1) yielded the title product as a colourless oil, as a 2.6:1 mixture of diastereoisomers (27 mg, 0.081 mmol, 81%). ^1H NMR (400 MHz, $CDCl_3$) δ 0.95–1.04 (m, 3H, CH_2C*H*$_3$), 1.42–1.81 (m, 7H, C*H*$_2CH_3$ + CH_2C*H*$_2CHCO_2CH_3$ + CO_2CHC*H*$_a$H$_b$$CH_2$ + CO_2CHCH_2C*H*$_a$H$_b$ + C*H*$_a$H$_b$$CH_2CHCO_2CH_3$), 1.83–2.11 (m, 3H, CO_2CHCH_a*H*$_b$$CH_2$ + $CO_2CHCH_2CH_a$*H*$_b$ + CH_a*H*$_b$$CH_2CHCO_2CH_3$), 2.28–2.36 (m, 0.72H, C*H*CO_2CH from major

diastereoisomer), 2.39–2.49 (m, 0.28H, $CHCO_2CH$ from minor diastereoisomer), 2.69–2.78 (m, 1H, $CHCO_2CH_3$), 2.84–2.94 (m, 2H, CH_2Ar), 3.61 (s, 3H, CO_2CH_3), 4.13–4.25 (m, 1H, CO_2CH), 6.97–7.08 (m, 2H, ArC*H*), 7.10–7.25 (m, 2H, ArC*H*) ppm; ^{13}C NMR (101 MHz, $CDCl_3$) δ 9.2 (CH_2CH_3), 25.6 ($CH_2CH_2CHCO_2CH_3$), 28.3 ($CH_2CH_2CHCO_2CH_3$), 29.1 (CH_2CH_3), 29.3 ($CO_2CHCH_2CH_2$), 29.7 ($CO_2CHCH_2CH_2$), 31.8 (d, *J* = 2.0 Hz, CH_2Ar), 40.8 ($CHCO_2CH$), 47.4 ($CHCH_2Ar$), 51.6 (CO_2CH_3), 82.6 (CO_2CH), 115.3 (d, *J* = 22.1 Hz, ArCH), 124.0 (d, *J* = 3.7 Hz, ArCH), 126.0 (d, *J* = 15.7 Hz, ArCH), 128.3 (d, *J* = 8.2 Hz, ArCH), 131.2 (d, *J* = 4.5 Hz, ArC), 161.2 (d, *J* = 245.2 Hz, ArCF), 173.5 (CO_2CH), 175.5 (CO_2CH_3) ppm; ^{19}F NMR (376 MHz, $CDCl_3$) δ –118.0 ppm; IR ν_{max} (thin film, cm^{-1}) = 2933, 1729 (C=O), 1492, 1372, 1229, 1163, 1105; HRMS calcd. for $C_{19}H_{25}O_4FNa$ $[M+Na]^+$ 359.1629, found 359.1614.

Rac–methyl (_R_)–4–((3_S_,6_S_)–6–ethyl–2–oxotetrahydro–2_H_–pyran–3–yl)–2–(2–methylbenzyl) butanoate (3l)

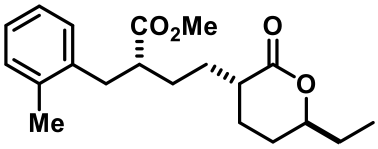

Prepared according to General Procedure E using SmI_2 (2.5 mL, 0.1 M in THF, 0.25 mmol, 2.5 eq.), HMPA (0.17 mL, 1.0 mmol, 10 eq.), H_2O (29 µL, 1.6 mmol, 16 eq.) and *rac*–methyl (3*R*,6*S*)–6–ethyl–2–oxo–3–((*E*)–4–(o–tolyl)but–3–en–1–yl)tetrahydro–2*H*–pyran–3–carboxylate **1l** (33 mg, 0.1 mmol, 1.0 eq.) in THF (0.5 mL). Purification by silica gel column chromatography (Hexane/EtOAc 9:1) yielded the title product as a yellow oil, as a 1.7:1 mixture of diastereoisomers (12 mg, 0.037 mmol, 37%). ^{1}H NMR (400 MHz, MeOD) δ 1.01 (td, 3H, *J* = 7.5, 2.6 Hz, CH_2CH_3), 1.51–1.59 (m, 2H, CH_2CH_3), 1.66 (dqt, 3H, *J* = 10.2, 5.3, 2.6 Hz, $CH_2CH_aH_bCHCO_2CH_3$ + $C(O)CHCH_2CH_2$), 1.72–1.83 (m, 1H, $C(O)CHCH_aH_bCH_2$), 1.85–1.94 (m, 1H, $CH_2CH_aH_bCHCO_2CH_3$), 1.94–2.02 (m, 2H, $CH_2CH_2CHCO_2CH_3$), 2.05–2.19 (m, 1H, $C(O)CHCH_aH_bCH_2$), 2.32 (s, 3H, $ArCH_3$), 2.43 (ddd, 0.63H, *J* = 13.0, 6.6, 3.5 Hz, $CHCO_2CH$ from major diastereoisomer), 2.58 (dq, 0.37H, *J* = 9.5, 6.5 Hz, $CHCO_2CH$ from minor diastereoisomer), 2.70 (tdd, 1H, *J* = 7.8, 6.0, 4.3 Hz, $CHCO_2CH_3$), 2.83 (ddd, 1H, *J* = 13.8, 6.2, 2.8 Hz, $CH_aH_bCHCO_2CH_3$), 2.93 (ddd, 1H, *J* = 12.7, 8.9, 3.6 Hz, $CH_aH_bCHCO_2CH_3$), 3.59 (s, 3H, CO_2CH_3), 4.21–4.35 (m, 1H, CO_2CH), 7.06–7.17 (m, 4H, ArC*H*) ppm; ^{13}C NMR (101 MHz, MeOD) δ 8.1 (CH_2CH_3), 18.1 ($ArCH_3$), 24.9 ($CH_2CH_2CHCO_2CH_3$), 27.8 ($C(O)CHCH_2CH_2$), 28.7 (CH_2CH_3), 29.2 ($CH_2CH_2CHCO_2CH_3$), 29.3 ($C(O)CHCH_2CH_2$), 35.5 ($ArCH_2CHCO_2CH_3$), 40.3 ($CHCO_2$), 46.2 ($CHCO_2CH_3$), 50.6 (CO_2CH_3), 83.1 (CO_2CH), 125.5 (ArCH), 126.2 (ArCH), 129.1 (ArCH), 129.9 (ArCH), 135.8 (ArC), 137.1 (ArC), 175.0 (CO_2CH), 176.2 (CO_2CH_3) ppm; IR ν_{max} (thin film, cm^{-1}) = 3727, 2930, 2364, 1732, 1456, 1163, 1102, 775, 701, 669; HRMS calculated for $C_{20}H_{29}O_4$ $[M+H]^+$ 333.2060, found 333.2059.

Rac–methyl (_R_)–4–((3_S_,6_S_)–6–ethyl–2–oxotetrahydro–2_H_–pyran–3–yl)–2–(3–methylbenzyl) butanoate (3m)

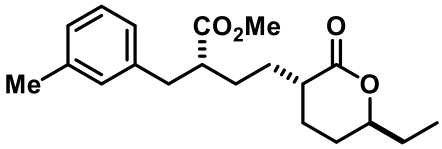

Prepared according to General Procedure E using SmI_2 (2.5 mL, 0.1 M in THF, 0.25 mmol, 2.5 eq.), HMPA (0.17 mL, 1.0 mmol, 10 eq.), H_2O (29 µL, 1.6 mmol, 16 eq.) and *rac*–methyl (3*R*,6*S*)–6–ethyl–2–oxo–3–

((*E*)–4–(*m*–tolyl)but–3–en–1–yl)tetrahydro–2*H*–pyran–3–carboxylate **1m** (33 mg, 0.1 mmol, 1.0 eq.) in THF (0.5 mL). Purification by silica gel column chromatography (Hexane/EtOAc 9:1) yielded the title product as a yellow oil, as a 2.0:1 mixture of diastereoisomers (11 mg, 0.033 mmol, 34%). ^{1}H NMR (400 MHz, MeOD–*d₄*) δ 1.01 (td, 3H, *J* = 7.5, 2.9 Hz, CH_2CH_3), 1.50–1.58 (m, 2H, CH_2CH_3), 1.62–1.69 (m, 3H, $CH_2CH_aH_bCHCO_2CH_3$ + C(O)$CHCH_2CH_2$), 1.72–1.82 (m, 1H, C(O)$CHCH_aH_bCH_2$), 1.86–1.93 (m, 1H, $CH_2CH_aH_bCHCO_2CH_3$), 2.01–1.93 (m, 2H, $CH_2CH_2CHCO_2CH_3$), 2.10 (dq, 1H, *J* = 13.0, 9.1, 7.9 Hz, C(O)$CHCH_aH_bCH_2$), 2.31 (s, 3H, ArCH_3), 2.37–2.46 (m, 0.66H, $CHCO_2CH$ from major diastereoisomer), 2.52–2.62 (m, 0.34H, $CHCO_2CH$ from minor diastereoisomer), 2.65–2.72 (m, 1H, $CHCO_2CH_3$), 2.72–2.80 (m, 1H, $CH_aH_bCHCO_2CH_3$), 2.88 (ddd, 1H, *J* = 13.3, 8.6, 4.7 Hz, $CH_aH_bCHCO_2CH_3$), 3.61 (s, 3H, CO_2CH_3), 4.20–4.35 (m, 1H, CO_2CH), 6.94–7.03 (m, 3H, ArC*H*), 7.15 (td, 1H, *J* = 7.5, 2.1 Hz, ArC*H*) ppm; ^{13}C NMR (101 MHz, MeOD–*d₄*) δ 8.1 (CH_2CH_3), 20.0 (ArCH_3), 24.8 ($CH_2CH_2CHCO_2CH_3$), 27.8 (C(O)$CHCH_2CH_2$), 28.7 (CH_2CH_3), 28.8 ($CH_2CH_2CHCO_2CH_3$), 29.1 (CO_2CHCH_2), 38.0 (CHCH_2Ar), 40.3 ($CHCO_2$), 47.5 ($CHCO_2CH_3$), 50.6 (CO_2CH_3), 83.1 (CO_2CH), 125.5 (Ar*C*H), 126.7 (Ar*C*H), 127.9 (Ar*C*H), 129.2 (Ar*C*H), 137.7 (Ar*C*), 139.0 (Ar*C*), 174.9 (CO_2CH), 176.1 (CO_2CH_3) ppm; IR ν_{max} (thin film, cm^{-1}) = 2930, 2363, 1733, 1458, 1375, 1165, 743, 668, 661; HRMS calculated for $C_{20}H_{29}O_4$ $[M+H]^+$ 333.2060, found 333.2056.

***Rac*–methyl (*R*)–2–(3–chlorobenzyl)–4–((3*S*,6*S*)–6–ethyl–2–oxotetrahydro–2*H*–pyran–3–yl) butanoate (3n)**

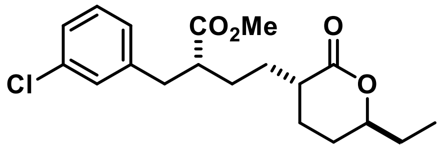

Prepared according to General Procedure E using SmI_2 (2.5 mL, 0.1 M in THF, 0.25 mmol, 2.5 eq.), HMPA (0.17 mL, 1.0 mmol, 10 eq.), H_2O (29 µL, 1.6 mmol, 16 eq.) and *rac*–methyl (3*R*,6*S*)–3–((*E*)–4–(3–chlorophenyl)but–3–en–1–yl)–6–ethyl–2–oxotetrahydro–2*H*–pyran–3–carboxylate **1n** (35 mg, 0.10 mmol, 1.0 eq.). Purification by silica gel column chromatography (hexane/EtOAc 85:15) yielded the title product as a yellow oil, as a 3.9:1 mixture of diastereoisomers (20 mg, 0.057 mmol, 57%). ^{1}H NMR (400 MHz, $CDCl_3$) δ 0.99 (t, *J* = 7.5 Hz, 3H, CH_2CH_3), 1.46–1.79 (m, 7H, CH_2CH_3 + $CO_2CHCH_aH_b$ + $CO_2CHCH_2CH_aH_b$ + $CH_aH_bCH_2CHCO_2CH_3$ + $CH_2CHCO_2CH_3$), 1.83–2.08 (m, 3H, $CO_2CHCH_aH_b$ + $CO_2CHCH_2CH_aH_b$ + $CH_aH_bCH_2CHCO_2CH_3$), 2.26–2.37 (m, 0.8H, $CHCO_2CH$ from major diastereoisomer), 2.39–2.48 (m, 0.2H, $CHCO_2CH$ from minor diastereoisomer), 2.62–2.69 (m, 1H, $CHCO_2CH_3$), 2.74 (dd, *J* = 13.6, 6.6 Hz, 1H, CH_aH_bAr), 2.93 (dd, *J* = 13.6, 8.2 Hz, 1H, CH_aH_bAr), 3.62 (s, 3H, CO_2CH_3), 4.13–4.24 (m, 1H, CO_2CH), 7.00–7.18 (m, 1H, ArC*H*), 7.13–7.24 (m, 3H, ArC*H*) ppm; ^{13}C NMR (101 MHz, $CDCl_3$) δ 9.2 (CH_2CH_3), 25.6 ($CH_2CH_2CHCO_2CH_3$), 28.3 ($CH_2CH_2CHCO_2CH_3$), 29.1 (CH_2CH_3), 29.3 ($CO_2CHCH_2CH_2$), 29.7 (CO_2CHCH_2), 38.0 (CH_2Ar), 40.8 ($CHCO_2CH$), 47.2 ($CHCH_2$Ar), 51.6 (CO_2CH_3), 82.6 (CO_2CH), 126.7 (Ar*C*H), 127.0 (Ar*C*H), 129.0 (Ar*C*H), 129.7 (Ar*C*H), 134.2 (Ar*C*Cl), 141.1 (Ar*C*), 173.4 (CO_2CH), 175.4 (CO_2CH_3) ppm; IR ν_{max} (thin film, cm^{-1}) = 2202, 1733 (C=O), 1538, 1422, 1264, 1089; HRMS calcd. for $C_{19}H_{25}O_4ClNa$ $[M+Na]^+$ 375.1334, found 375.1319.

***Rac*–methyl (*R*)–4–((3*S*,6*S*)–6–ethyl–2–oxotetrahydro–2*H*–pyran–3–yl)–2–(naphthalen–1–ylmethyl)butanoate (3o)**

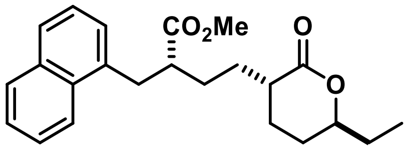

Prepared according to General Procedure E using SmI_2 (2.5 mL, 0.1 M in THF, 0.25 mmol, 2.5 eq.), HMPA (0.17 mL, 1.0 mmol, 10 eq.), H_2O (29 µL, 1.6 mmol, 16 eq.) and *rac*–methyl (3*R*,6*S*)–6–ethyl–3–((*E*)–4–(naphthalen–2–yl)but–3–en–1–yl)–2–oxotetrahydro–2*H*–pyran–3–carboxylate **1o** (37 mg, 0.10 mmol, 1.0 eq.). Purification by silica gel column chromatography (hexane/EtOAc 9:1) yielded the title product as a colourless oil, as a 2.7:1 mixture of diastereoisomers (23 mg, 0.062 mmol, 62%). ^{1}H NMR (400 MHz, $CDCl_3$) δ 0.95–1.05 (m, 3H, CH_2CH_3), 1.40–1.85 (m, 7H, CH_2CH_3 + $CH_2CH_2CHCO_2CH_3$ + $CO_2CHCH_aH_b$ + $CO_2CHCH_2CH_aH_b$ + $CH_aH_bCH_2CHCO_2CH_3$), 1.85–2.10 (m, 3H, $CO_2CHCH_aH_b$ + $CO_2CHCH_2CH_aH_b$ + $CH_aH_bCH_2CHCO_2CH_3$), 2.26–2.35 (m, 0.73H, $CHCO_2CH$ from major diastereoisomer), 2.37–2.48 (m, 0.27H, $CHCO_2CH$ from minor diastereoisomer), 2.75–2.84 (m, 1H, $CHCH_2Ar$), 2.93 (dd, *J* = 13.6, 6.8 Hz, 1H, CH_aH_bAr), 3.14 (dd, *J* = 13.6, 7.9 Hz, 1H, CH_aH_bAr), 3.60 (s, 3H, CO_2CH_3), 4.12–4.21 (m, 1H, CO_2CH), 7.30 (dd, *J* = 8.4, 1.8 Hz, 1H, ArC*H*), 7.45 (tt, *J* = 6.8, 5.2 Hz, 2H, ArC*H*), 7.61 (d, *J* = 2.0 Hz, 1H, ArC*H*), 7.73–7.84 (m, 3H, ArC*H*) ppm; ^{13}C NMR (101 MHz, $CDCl_3$) δ 9.2 (CH_2CH_3), 25.5 ($CH_2CH_2CHCO_2CH_3$), 28.3 ($CO_2CHCH_2CH_2$), 29.1 (CH_2CH_3), 29.3 ($CH_2CH_2CHCO_2CH_3$), 29.8 (CO_2CHCH_2), 38.7 (CH_2Ar), 40.8 ($CHCO_2CH$), 47.4 ($CHCH_2Ar$), 51.6 (CO_2CH_3), 82.6 (CO_2CH), 125.5 (Ar*C*H), 126.0 (Ar*C*H), 127.2 (Ar*C*H), 127.3 (Ar*C*H), 127.6 (Ar*C*H), 127.6 (Ar*C*H), 128.1 (Ar*C*H), 132.2 (Ar*C*), 133.5 (Ar*C*), 136.6 (Ar*C*), 173.5 (CO_2CH), 175.8 (CO_2CH_3) ppm; IR ν_{max} (thin film, cm^{-1}) = 2953, 1729 (C=O), 1455, 1239, 1191, 1092, 963; HRMS calcd. for $C_{23}H_{28}O_4Na$ $[M+Na]^+$ 391.1880, found 391.1863.

***Rac*–methyl (*R*)–2–([1,1'–biphenyl]–4–ylmethyl)–4–((3*S*,6*S*)–6–ethyl–2–oxotetrahydro–2*H*–pyran–3–yl)butanoate (3p)**

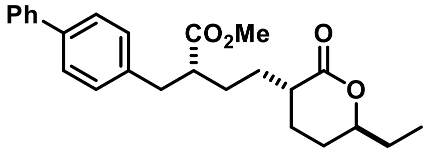

Prepared according to General Procedure E using SmI_2 (2.5 mL, 0.1 M in THF, 0.25 mmol, 2.5 eq.), HMPA (0.17 mL, 1.0 mmol, 10 eq.), H_2O (29 µL, 1.6 mmol, 16 eq.) and *rac*–methyl (3*R*,6*S*)–3–((*E*)–4–([1,1'–biphenyl]–4–yl)but–3–en–1–yl)–6–ethyl–2–oxotetrahydro–2*H*–pyran–3–carboxylate **1p** (39 mg, 0.1 mmol, 1.0 eq.). Purification by silica gel column chromatography (Hexane/EtOAc 9:1) yielded the title product as a white solid, as a 2:1 mixture of diastereoisomers (16 mg, 0.041 mmol, 41%). M.p. 111–113 °C. ^{1}H NMR (400 MHz, $CDCl_3$) δ 0.98 (td, 3H, *J* = 7.5, 4.4 Hz, (CH_2CH_3), 1.45–1.81 (m, 8H, $CH_2CH_2CHCO_2CH_3$ + $C(O)CHCH_2CH_2$ + CH_2CH_3 + $CH_aH_bCH_2CHCO_2CH_3$ + $C(O)CHCH_aH_bCH_2$), 1.86–1.95 (m, 1H, $CH_aH_bCH_2CHCO_2CH_3$), 1.96–2.06 (m, 1H, $C(O)CHCH_aH_bCH_2$), 2.27–2.36 (m, 0.67H, $CHCO_2CH$ from major diastereoisomer), 2.44 (dt, 0.33H, *J* = 15.2, 7.1 Hz, $CHCO_2CH$ from minor diastereoisomer), 2.71 (tdt, 1H, *J* = 8.1, 4.7, 2.1 Hz, $CHCO_2CH_3$), 2.82 (ddd, 1H, *J* = 13.6, 10.1, 6.5 Hz, CH_2CHCO_2Me), 2.99 (ddd, 2H, *J* = 14.8, 8.3, 6.8 Hz, CH_2CHCO_2Me), 3.60 (s, 3H, CO_2CH_3), 4.18 (dt, 1H, *J* = 9.3, 2.7 Hz, CO_2CH), 7.22 (d, 2H, *J* = 8.1, ArC*H*), 7.31–7.35 (m, 1H, ArC*H*), 7.43 (t, 2H, *J* = 7.6 Hz, ArC*H*), 7.51 (d, 2H, *J* = 8.2 Hz, ArC*H*), 7.56–7.59 (m, 2H, ArC*H*) ppm; ^{13}C NMR (101 MHz, $CDCl_3$) δ 9.2 (CH_2CH_3), 25.6 ($CH_2CH_2CHCO_2CH_3$), 28.3 ($C(O)CHCH_2CH_2$), 29.1 (CH_2CH_3), 29.3 ($C(O)CHCH_2CH_2$), 29.8 ($CH_2CH_2CHCO_2CH_3$), 38.1 ($CHCH_2Ar$), 40.8 ($C(O)CH$), 47.4 ($CHCO_2CH_3$), 51.6 (CO_2CH_3), 82.6 (CO_2CH), 127.0 (Ar*C*H), 127.1 (Ar*C*H), 127.1 (Ar*C*H), 128.7 (Ar*C*H), 129.3 (Ar*C*H), 138.1 (Ar*C*), 139.3

(Ar*C*), 140.9 (Ar*C*), 173.5 ($\mathit{C}O_2CH$), 175.7 ($\mathit{C}O_2CH_3$) ppm; IR ν_{max} (thin film, cm^{-1}) = 3732, 2924, 2334, 1733, 683, 668; HRMS calculated for $C_{25}H_{30}O_4Na$ $[M+Na]^+$ 417.2036, found 417.2023.

Rac–methyl (_R_)–4–((3_S_,6_S_)–6–ethyl–2–oxotetrahydro–2_H_–pyran–3–yl)–2–(4–fluorobenzyl) butanoate (3q)

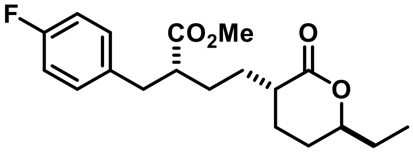

Prepared according to General Procedure E using SmI_2 (2.5 mL, 0.1 M in THF, 0.25 mmol, 2.5 eq.), HMPA (0.17 mL, 1.0 mmol, 10 eq.), H_2O (29 µL, 1.6 mmol, 16 eq.) and *rac*–methyl (3*R*,6*S*)–6–ethyl–3–((*E*)–4–(4–fluorophenyl)but–3–en–1–yl)–2–oxotetrahydro–2*H*–pyran–3–carboxylate **1q** (33 mg, 0.1 mmol, 1.0 eq.) in THF (0.5 mL). Purification by silica gel column chromatography (Hexane/EtOAc 9.8:0.2 to 9.5:0.5) yielded the title product as a yellow oil, as a 3.3:1 mixture of diastereoisomers (19 mg, 0.058 mmol, 58%). ^1H NMR (400 MHz, $CDCl_3$) δ 0.98 (dtd, 3H, *J* = 7.5, 4.6, 2.3 Hz, $CH_2C\mathit{H}_3$), 1.45–1.77 (m, 8H, $C\mathit{H}_2CH_3$ + $C(O)CHC\mathit{H}_aH_bCH_2$ + $CH_2C\mathit{H}_aH_bCHCO_2CH_3$ + $C(O)CHCH_2C\mathit{H}_2$ + $C\mathit{H}_2CH_2CHCO_2CH_3$), 1.90–2.02 (m, 2H, $C(O)CHCH_a\mathit{H}_bCH_2$ + $CH_2CH_a\mathit{H}_bCHCO_2CH_3$), 2.26–2.35 (m, 0.75H, $C\mathit{H}CO_2CH$ from major diastereoisomer), 2.42 (dq, 0.25H, *J* = 9.3, 7.0 Hz, $C\mathit{H}CO_2CH$ from minor diastereoisomer), 2.63 (tddd, 1H, *J* = 8.3, 6.1, 4.6, 1.5 Hz, $C\mathit{H}CO_2CH_3$), 2.74 (dd, 1H, *J* = 13.7, 6.8 Hz, $C\mathit{H}_aH_bCHCO_2Me$), 2.90 (dt, 2H, *J* = 13.7, 6.9 Hz, $CH_a\mathit{H}_bCHCO_2Me$), 3.60 (s, 3H, $CO_2C\mathit{H}_3$), 4.13–4.23 (m, 1H, $CO_2C\mathit{H}$), 6.95 (tt, 2H, *J* = 8.6, 1.8 Hz, ArC*H*), 7.10 (ddd, 2H, *J* = 8.8, 5.4, 1.7 Hz, ArC*H*) ppm; ^{13}C NMR (101 MHz, $CDCl_3$) δ 9.2 ($CH_2\mathit{C}H_3$), 25.6 ($C(O)CH\mathit{C}H_2CH_2$), 28.3 ($C(O)CHCH_2\mathit{C}H_2$), 29.1 ($\mathit{C}H_2CH_3$), 29.3 ($\mathit{C}H_2CH_2CHCO_2CH_3$), 29.7 ($CH_2\mathit{C}H_2CHCO_2CH_3$), 37.6 ($CH\mathit{C}H_2Ar$), 40.8 ($CHCO_2\mathit{C}H$), 47.6 ($C\mathit{H}CO_2CH_3$), 51.6 ($CO_2\mathit{C}H_3$), 82.6 ($CO_2C\mathit{H}$), 115.2 (d, *J* = 21.2 Hz, 2 x Ar*C*H), 130.2 (d, *J* = 7.9 Hz, 2 x Ar*C*H), 134.7 (Ar*C*), 161.5 (d, *J* = 244.2 Hz, Ar*C*F), 173.4 ($\mathit{C}O_2CH$), 175.5 ($\mathit{C}O_2CH_3$) ppm; ^{19}F (471 MHz, $CDCl_3$) δ ppm –116.7; IR ν_{max} (thin film, cm^{-1}) = 2949, 1728, 1601, 1510, 1446, 1370, 1220, 1159, 1101, 1056, 825, 758, 668; HRMS calculated for $C_{19}H_{26}O_4F$ $[M+H]^+$ 337.1810, found 337.1801.

Rac–methyl (_R_)–2–(4–bromobenzyl)–4–((3_S_,6_S_)–6–ethyl–2–oxotetrahydro–2_H_–pyran–3–yl) butanoate (3r)

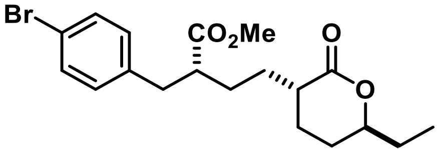

Prepared according to General Procedure E using SmI_2 (2.5 mL, 0.1 M in THF, 0.25 mmol, 2.5 eq.), HMPA (0.17 mL, 1.0 mmol, 10 eq.), H_2O (29 µL, 1.6 mmol, 16 eq.) and *rac*–methyl (3*R*,6*S*)–3–((*E*)–4–(4–bromophenyl)but–3–en–1–yl)–6–ethyl–2–oxotetrahydro–2*H*–pyran–3–carboxylate **1r** (39 mg, 0.10 mmol, 1.0 eq.). Purification by silica gel column chromatography (hexane/EtOAc 9:1) yielded the title product as a colourless oil, as a 2.5:1 mixture of diastereoisomers (27 mg, 0.067 mmol, 67%). ^1H NMR (500 MHz, $CDCl_3$) δ 0.99 (t, *J* = 7.5 Hz, 3H, $CH_2C\mathit{H}_3$), 1.44–1.66 (m, 5H, $C\mathit{H}_2CHCO_2CH_3$ + $C\mathit{H}_aH_bCH_2CHCO_2CH_3$ + $CO_2CHC\mathit{H}_aH_bCH_2$ + $CO_2CHCH_2C\mathit{H}_aH_b$), 1.66–1.78 (m, 2H, $C\mathit{H}_2CH_3$), 1.81–2.09 (m, 3H, $CH_a\mathit{H}_bCH_2CHCO_2CH_3$ + $CO_2CHCH_a\mathit{H}_bCH_2$ + $CO_2CHCH_2CH_a\mathit{H}_b$), 2.27–2.35 (m, 0.71H, $C\mathit{H}CO_2CH$ from major diastereoisomer), 2.38–2.48 (m, 0.29H, $C\mathit{H}CO_2CH$ from minor diastereoisomer), 2.59–2.69 (m, 1H,

C*H*CO_2CH_3), 2.73 (dd, *J* = 13.7, 6.4 Hz, 1H, CH_aH_bAr), 2.90 (dd, *J* = 13.7, 8.4 Hz, 1H, CH_aH_bAr), 3.61 (s, 3H, CO_2CH_3), 4.13–4.22 (m, 1H, CO_2C*H*), 7.03 (d, *J* = 8.4 Hz, 2H, ArC*H*), 7.40 (d, *J* = 8.4 Hz, 2H, ArC*H*) ppm; ^{13}C NMR (126 MHz, $CDCl_3$) δ 9.2 (CH_2CH_3), 25.6 ($CH_2CH_2CHCO_2CH_3$), 28.3 ($CH_2CH_2CHCO_2CH_3$), 29.1 (CH_2CH_3), 29.3 ($CO_2CHCH_2CH_2$), 29.7 ($CO_2CHCH_2CH_2$), 37.8 (CH_2Ar), 40.8 (*C*HCO_2CH), 47.3 (*C*HCH_2Ar), 51.6 (CO_2CH_3), 82.6 (CO_2*C*H), 120.3 (Ar*C*Br), 130.6 (Ar*C*H), 131.5 (Ar*C*H), 138.0 (Ar*C*), 173.4 (*C*O_2CH), 175.4 (*C*O_2CH_3) ppm; IR ν_{max} (thin film, cm^{-1}) = 2950, 2926, 1730 (C=O), 1488, 1246, 1193, 1102; HRMS calcd. for $C_{19}H_{25}O_4$BrNa $[M+Na]^+$ 419.0828, found 419.0815.

Rac–methyl (_R_)–4–((3_S_,6_S_)–6–ethyl–2–oxotetrahydro–2_H_–pyran–3–yl)–2–(4–(trifluoromethyl)benzyl) butanoate (3s)

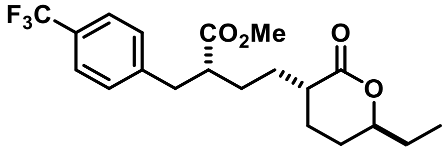

Prepared according to General Procedure E using SmI_2 (2 mL, 0.1 M in THF, 0.2 mmol, 2.5 eq.), HMPA (0.14 mL, 0.8 mmol, 10 eq.), H_2O (20 µL, 1.28 mmol, 16 eq.) and *rac*–methyl (3*R*,6*S*)–6–ethyl–2–oxo–3–((*E*)–4–(4–(trifluoromethyl)phenyl)but–3–en–1–yl)tetrahydro–2*H*–pyran–3–carboxylate **1s** (30 mg, 0.08 mmol, 1.0 eq.) in THF (0.5 mL). Purification by silica gel column chromatography (Hexane/EtOAc 9.5:0.5) yielded the title product as a yellow oil, as a 2.7:1 mixture of diastereoisomers (7.6 mg, 0.02 mmol, 25%). ^{1}H NMR (400 MHz, $CDCl_3$) δ 0.98 (t, 3H, *J* = 7.4 Hz, CH_2CH_3), 1.46–1.65 (m, 6H, CH_2CH_3 + C(O)CHC$H_aH_b$$CH_2$ + CH_2C$H_aH_b$$CHCO_2CH_3$ + C$H_aH_b$$CH_2CHCO_2CH_3$ + C(O)CHCH_2CH_aH_b), 1.66–1.77 (m, 2H, C$H_aH_b$$CH_2CHCO_2CH_3$ + CH_2C$H_aH_b$$CHCO_2CH_3$), 1.90–2.02 (m, 2H, C(O)CHC$H_aH_b$$CH_2$ + C(O)CHCH_2CH_aH_b), 2.30 (qd, 0.72H, *J* = 7.2, 6.3, 3.1 Hz, C*H*CO_2CH from major diastereoisomer), 2.38–2.54 (m, 0.28H, C*H*CO_2CH from minor diastereoisomer), 2.69 (tdd, 1H, *J* = 8.6, 6.2, 4.4 Hz, C*H*CO_2CH_3), 2.84 (dt, 1H, *J* = 13.6, 6.6 Hz, CH_2CHCO_2Me), 2.99 (qd, 2H, *J* = 9.5, 9.0, 4.6 Hz, CH_2CHCO_2Me), 3.60 (s, 3H, CO_2CH_3), 4.18 (qd, 1H, *J* = 5.9, 2.6 Hz, CO_2C*H*), 7.27 (d, 2H, *J* = 5.3 Hz, ArC*H*), 7.53 (d, 2H, *J* = 8.0 Hz, ArC*H*) ppm; ^{13}C NMR (101 MHz, $CDCl_3$) δ 9.2 (CH*C*H_3), 25.6 (C(O)CH*C*H_2CH_2), 28.3 (C(O)CHCH_2*C*H_2), 29.1 (*C*H_2CH_3), 29.5 (*C*$H_2CH_2CHCO_2CH_3$), 29.7 (CH_2*C*$H_2CHCO_2CH_3$), 38.1 (*C*H_2Ar), 40.8 (C(O)*C*H), 47.2 (*C*HCO_2CH_3), 51.7 (CO_2*C*H_3), 82.6 (CO_2C*H*), 125.4 (dt, 2H, *J* = 7.0, 3.8 Hz, Ar*C*H), 128.8 (d, *J* = 32.4 Hz, Ar*C*CF_3), 129.2 (d, 2H, *J* = 2.9 Hz, Ar*C*H), 143.2 (Ar*C*), 173.4 (*C*O_2CH), 175.2 (*C*O_2CH_3) ppm, *C*F_3 is not observed; ^{19}F NMR (471 MHz, $CDCl_3$) δ –62.4 ppm; IR ν_{max} (thin film, cm^{-1}) = 2925, 2154, 1732, 1458, 1325, 1163, 1122, 1067, 1019, 750; HRMS calculated for $C_{20}H_{25}F_3O_4$Na $[M+Na]^+$ 409.1597, found 409.1581.

Rac–methyl (_R_)–4–((3_S_,6_S_)–6–ethyl–2–oxotetrahydro–2_H_–pyran–3–yl)–2–(4–methoxybenzyl) butanoate (3t)

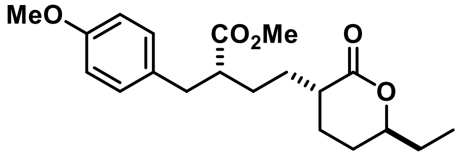

Prepared according to General Procedure E using SmI_2 (2.5 mL, 0.1 M in THF, 0.25 mmol, 2.5 eq.), HMPA (0.17 mL, 1.0 mmol, 10 eq.), H_2O (29 µL, 1.6 mmol, 16 eq.) and *rac*–methyl (3*R*,6*S*)–6–ethyl–3–((*E*)–4–(4–methoxyphenyl)but–3–en–1–yl)–2–oxotetrahydro–2*H*–pyran–3–carboxylate **1t** (35 mg, 0.10 mmol, 1.0 eq.).

Purification by silica gel column chromatography (hexane/EtOAc 85:15) yielded the title product as a yellow oil, as a 3.7:1 mixture of diastereoisomers (22 mg, 0.063 mmol, 63%). ^{1}H NMR (400 MHz, $CDCl_3$) δ 0.92–1.95 (m, 3H, $CH_2C\mathit{H_3}$), 1.41–1.76 (m, 7H, $C\mathit{H_2}CH_3$ + $CO_2CHC\mathit{H_a}H_b$ + $CO_2CHCH_2C\mathit{H_a}H_b$ + $C\mathit{H_a}H_bCH_2CHCO_2CH_3$ + $C\mathit{H_2}CHCO_2CH_3$), 1.84–2.01 (m, 3H, $CO_2CHCH_a\mathit{H_b}$ + $CO_2CHCH_2CH_a\mathit{H_b}$ + $CH_a\mathit{H_b}CH_2CHCO_2CH_3$), 2.24–2.34 (m, 0.79H, $C\mathit{H}CO_2CH$ from major diastereoisomer), 2.38–2.48 (m, 0.21H, $C\mathit{H}CO_2CH$ from minor diastereoisomer), 2.59–2.66 (m, 1H, $C\mathit{H}CO_2CH_3$), 2.71 (dd, *J* = 13.6, 6.8 Hz, 1H, $C\mathit{H_a}H_bAr$), 2.89 (dd, *J* = 13.6, 7.7 Hz, 1H, $CH_a\mathit{H_b}Ar$), 3.61 (s, 3H, $CO_2C\mathit{H_3}$), 3.78 (s, 3H, $ArOC\mathit{H_3}$), 4.13–4.22 (m, 1H, $CO_2C\mathit{H}$), 6.76–6.85 (m, 2H, $ArC\mathit{H}$), 6.98–7.13 (m, 2H, $ArC\mathit{H}$) ppm; ^{13}C NMR (101 MHz, $CDCl_3$) δ 9.2 ($CH_2\mathit{C}H_3$), 25.5 ($CO_2CHCH_2\mathit{C}H_2$), 28.2 ($CO_2CH\mathit{C}H_2$), 29.1 ($\mathit{C}H_2CHCO_2CH_3$), 29.2 ($\mathit{C}H_2CH_3$), 29.8 ($\mathit{C}H_2CH_2CHCO_2CH_3$), 37.6 ($\mathit{C}H_2Ar$), 40.8 ($\mathit{C}HCO_2CH$), 47.7 ($\mathit{C}HCO_2CH_3$), 51.5 ($CO_2\mathit{C}H_3$), 55.2 ($ArO\mathit{C}H_3$), 82.6 ($CO_2\mathit{C}H$), 113.8 ($Ar\mathit{C}H$), 129.8 ($Ar\mathit{C}H$), 131.1 ($Ar\mathit{C}$), 158.2 ($Ar\mathit{C}OCH_3$), 173.5 ($\mathit{C}O_2CH$), 175.8 ($\mathit{C}O_2CH_3$) ppm; IR v_{max} (thin film, cm^{-1}) = 2956, 2178, 1733 (C=O), 1443, 1271, 1217, 1193; HRMS calcd. for $C_{20}H_{28}O_5Na$ $[M+Na]^+$ 371.1829, found 371.1815.

***Rac*-methyl (*R*)-4-((3*S*,6*S*)-6-methyl-2-oxotetrahydro-2H-pyran-3-yl)-2-(thiophen-2-ylmethyl)butanoate (3u)**

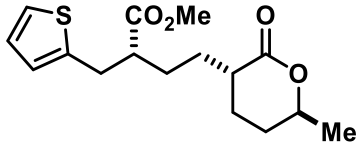

Prepared according to General Procedure E using SmI_2 (2.2 ml, 0.1 M in THF, 0.22 mmol, 2.5 eq.) HMPA (0.15 mL, 0.88 mmol, 10 eq.), H_2O (25 µL, 1.41 mmol, 16 eq.) and **1u** (27 mg, 0.088 mmol, 1.0 eq.). Purification by silica gel column chromatography (hexanes/EtOAc 75:25) yielded the title copound as a colourless oil, as a 4.3:1 mixture of diastereoisomers (15 mg, 0.048 mmol, 55%). ^{1}H NMR (400 MHz, $CDCl_3$, major diastereomer) δ 7.12 (dt, *J* = 5.2, 1.7 Hz, 1H, $ArC\mathit{H}$), 6.95 – 6.86 (m, 1H, $ArC\mathit{H}$), 6.78 (dd, *J* = 3.4, 1.2 Hz, 1H, $ArC\mathit{H}$), 4.40 (m, 1H, $CO_2C\mathit{H}CH_3$), 3.65 (s, 3H, $CO_2C\mathit{H_3}$), 3.17 (ddd, *J* = 14.8, 7.9, 0.9 Hz, 1H, $ArC_qC\mathit{H_a}H_b$), 3.08 – 2.91 (m, 1H, $ArC_qCH_a\mathit{H_b}$), 2.69 (tdt, *J* = 8.6, 6.4, 4.1 Hz, 1H, $C\mathit{H}CO_2Me$), 2.37 – 2.26 (m, 1H, $C\mathit{H}CO_2CH$), 2.04 – 1.85 (m, 3H, $C\mathit{H_a}H_bCH_2CHMe$, $C\mathit{H_a}H_bCHCH_3$, $C\mathit{H_a}H_bCH_2CHCO_2Me$), 1.80 – 1.67 (m, 1H, $ArC_qCH_2CHC\mathit{H_a}H_b$), 1.67 – 1.57 (m, 1H, $ArC_qCH_2CHCH_a\mathit{H_b}$), 1.56 – 1.45 (m, 3H, $CH_a\mathit{H_b}CH_2CHMe$, $CH_a\mathit{H_b}CHCH_3$, $CH_a\mathit{H_b}CH_2CHCO_2Me$), 1.35 (d, *J* = 6.2 Hz, 3H, $CHC\mathit{H_3}$). ^{13}C NMR (101 MHz, $CDCl_3$, major diastereomer) δ 175.4 ($\mathit{C}O_2Me$), 173.5 ($\mathit{C}O_2CH$), 141.4 ($Ar\mathit{C}_q$), 127.0 ($Ar\mathit{C}H$), 125.7 ($Ar\mathit{C}H$), 124.0 ($Ar\mathit{C}H$), 77.9 ($CO_2\mathit{C}H$), 51.8 ($CO_2\mathit{C}H_3$), 47.8 ($\mathit{C}HCO_2Me$), 40.5 ($\mathit{C}HCO_2CH$), 32.3 ($ArC_q\mathit{C}H_2$), 30.8 ($\mathit{C}H_2CHCH_3$), 29.7 ($\mathit{C}H_2CH_2CHCO_2Me$), 29.1 ($ArC_qCH_2CH\mathit{C}H_2$), 25.8 ($\mathit{C}H_2CH_2CHMe$), 22.2 ($CH\mathit{C}H_3$). HRMS calcd. for $C_{16}H_{23}O_4S$ $[M+H]^+$ 311.1312, found 311.1308. IR v_{max} (thin film, cm^{-1}) = 2949, 2868, 1727, 1436, 1386.

***Rac*-methyl 4-(6-methyl-2-oxotetrahydro-2H-pyran-3-yl)-2-(1-phenylethyl)butanoate (3v)**

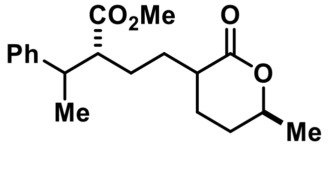

Prepared according to General Procedure E using SmI_2 (2.4 mL, 0.1 M in THF, 0.24 mmol, 2.5 eq.), HMPA (0.165 mL, 0.95 mmol, 10 eq.), H_2O (27 µL, 1.52 mmol, 16 eq.) and **1v** (30 mg, 0.095 mmol, 1.0 eq.).

Purification by silica gel column chromatography (hexanes/EtOAc 70:30) yielded the title product as a yellow oil, as a 3.2:2.0:1.9:1 mixture of diastereoisomers (15 mg, 0.047 mmol, 50%). ^{1}H NMR (400 MHz, $CDCl_3$, major diastereomer) δ 7.37 – 7.11 (m, 5H, ArC*H*), 4.55 – 4.21 (m, 1H, OC*H*), 3.42 (s, 3H, OCH_3), 3.08 – 2.96 (m, 1H, C*H*), 2.63 – 2.49 (m, 1H, *C*HCO_2CH), 2.34 – 2.20 (m, 1H, C*H*), 2.04 – 1.87 (m, 3H, C$H_a$$H_b$, C$H_a$$H_b$, C$H_a$$H_b$), 1.86 – 1.70 (m, 2H, C$H_2$), 1.58 – 1.37 (m, 3H, C$H_a$$H_b$, C$H_a$$H_b$, C$H_a$$H_b$), 1.35 (d, J = 6.2 Hz, 3H, CH_3), 1.29 (d, J = 6.8 Hz, 3H, CH_3). ^{13}C NMR (101 MHz, $CDCl_3$, major diastereomer) δ 175.3 (*C*O_2CH_3), 173.6 (*C*O_2CH), 144.6 (ArC_q), 128.4 (Ar*C*H), 127.5 (Ar*C*H), 126.6 (Ar*C*H), 77.8 (O*C*H), 53.1 (*C*HCO_2CH), 51.4 (O*C*H_3), 42.2 (*C*H), 40.5 (*C*H), 30.8 (*C*H_2), 30.2 (*C*H_2), 27.0 (*C*H_2), 25.8 (*C*H_2), 22.2 (*C*H_3), 18.4 (*C*H_3). HRMS calcd. for $C_{19}H_{27}O_4$ $[M+H]^+$ 319.1904, found 319.1905. IR ν_{max} (thin film, cm^{-1}) = 3027, 2949, 2932, 2873, 1727, 1453, 1386.

5. Unsuccessful substrates

The following substrates gave no product of 1,4-ester migration.

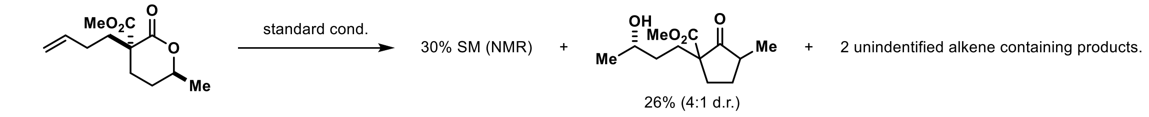

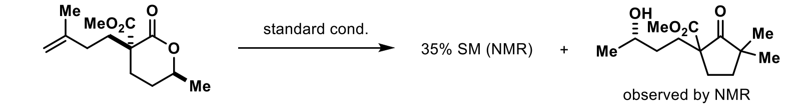

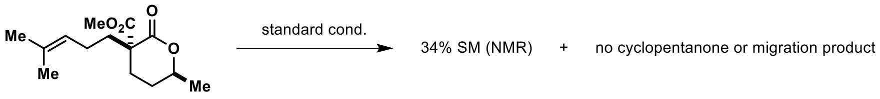

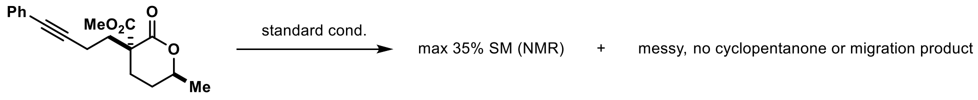

6. Mechanistic Investigations

5.1 Origin of the Diastereoselectivity

Influence of different proton sources on reactivity:

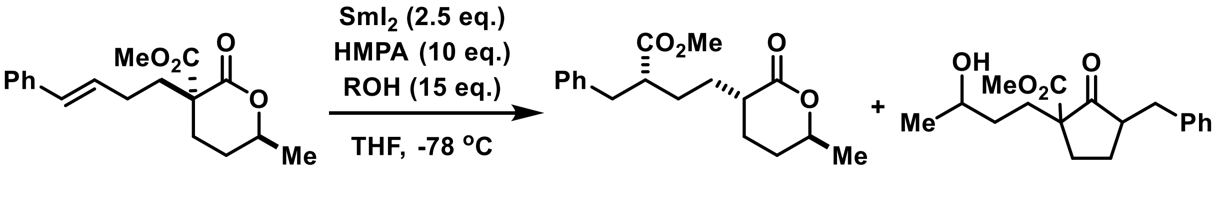

1a 3a 2a

Entry	ROH	Conversion (%)	3a:2a	dr of 3a
**1**	H_2O	100 (76)	11.5:1	3:1
**2**	MeOH	100	7.2:1	3:1
**3**	HFIP	100	14:1	1:1
**4**	^{i}PrOH	67	5.4:1	N.D.
**5**	Ethylene glycol	52	6.2:1	N.D.

Table S1

Different proton sources were tested in the benchmark reaction of lactone **1a**, in the presence of SmI_2 and HMPA, to evaluate their influence on the cyclization protocol. The study, summarized in Table S1, highlighted that the nature of the proton source influences both the reactivity, and the chemo- and the diastereoselectivity of the process. In particular, the diverse diastereoisomeric ratios observed in the reactions using water, methanol and HIPF (entries 1-3) suggest that the diastereoisomeric mixture, observed for product **3a**, arises from the protonation of the samarium(III)-enolate intermediate (c.f. Scheme 3 within the main manuscript). Whereas, the ester radical migration step occurs with complete stereocontrol.

X-ray analysis on product 3p:

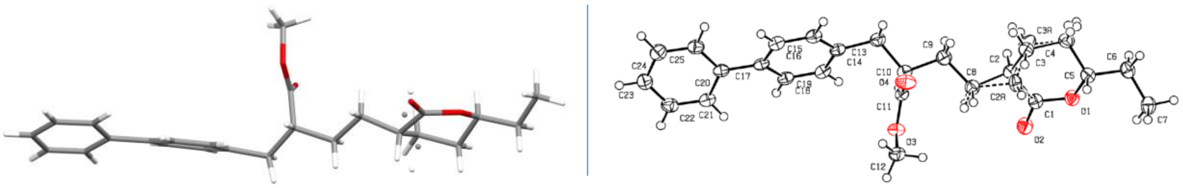

Figure S1

X-ray crystallographic analysis of a crystal containing a 2:1 diastereoisomeric mixture of **3p** (see section 7, for crystallographic data) reveals structural uncertainty at the position alpha-to-the lactone carbonyl (Figure S1). This evidence also indicates that the stereocentre at which there is a diastereoisomeric mixture is the C3-site of the lactone ring within products **3**.

5.2 Experimental Data for Scheme 3A: ^{13}C–labelling experiment

5.2.1 Synthesis of a ^{13}C–labelled substrate

(*E*)–6–Methyl–3–(4–phenylbut–3–en–1–yl)tetrahydro–2*H*–pyran–2–one (S32)

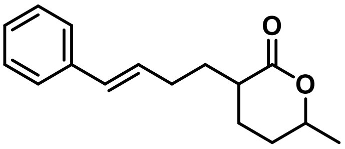

Prepared according to a modified literature procedure:[13] to a flame–dried flask was added diisopropylamine (1.27 mL, 919 mg, 9.09 mmol, 1.1 eq.) and dry THF (16.5 mL). The solution was cooled to 0 °C and *n*BuLi (3.63 mL, 2.5 M in THF, 1.1 eq., 9.09 mmol) was added dropwise. The resulting solution was stirred at 0 °C for 15 min and then cooled to –78 °C. A solution of 6–methyltetrahydro–2*H*–pyran–2–one (943 mg, 0.906 ml, 8.26 mmol, 1.0 eq.) in dry THF (33 mL) was added dropwise over 30 minutes. After stirring for a further 30 minutes, DMPU (4.09 mL, 4.34 g, 33.9 mmol, 4.1 eq.) was added dropwise and the solution was stirred for another 15 min at that temperature. A solution of (*E*)–(4–iodobut–1–en–1–yl)benzene[8] (2.35 g, 9.09 mmol, 1.1 eq.) in THF (8.3 mL) was added dropwise and the reaction was stirred at –78 °C for 3 h, then was allowed to warm to room temperature and was stirred overnight. The reaction was quenched by addition of sat. aq. NH_4Cl (50 mL) and the aqueous layer was extracted with EtOAc (3 x 50 mL). The organic layers were combined, dried ($MgSO_4$) and concentrated. Purification by silica gel column chromatography (hexane/EtOAc 75:25) yielded the title product as a white solid, as a 1.2:1 mixture of diastereoisomers (900 mg, 3.68 mmol, 45%). Major diastereoisomer: ^{1}H NMR (400 MHz, $CDCl_3$) δ 1.35 (d, *J* = 6.2 Hz, 3H, C*H*$_3$) 1.49–1.67 (m, 3H, CH_a*H*$_b$$CHCH_3$, C*H*$_a$$H_b$$CH_2CHCH_3$, CH=$CHCH_2$C*H*$_a$$H_b$), 1.88–2.00 (m, 1H, C*H*$_a$$H_b$$CHCH_3$), 2.09 (app. dddd, *J* = 18.2, 9.7, 4.4, 2.4 Hz, 2H, CH_a*H*$_b$$CH_2CHCH_3$, CH=$CHCH_2CH_a$*H*$_b$), 2.30–2.39 (m, 2H, CH=CHC*H*$_2$), 2.46–2.54 (m, 1H, C*H*C(O)), 4.42 (dddt, *J* = 11.6, 9.3, 5.8, 3.0 Hz, 1H, OC*H*CH_3), 6.19 (app. dtd, *J* = 15.0, 6.8, 1.1 Hz, 1H, ArCH=C*H*), 6.31–6.49 (app. d, *J* = 15.9 Hz, 1H, ArC*H*=CH), 7.15–7.24 (m, 1H, ArC*H*), 7.25–7.38 (m, 4H, ArC*H*), ppm; ^{13}C NMR (101 MHz, $CDCl_3$) δ 21.1 (*C*H_3), 23.5 (*C*H_2), 28.6 (*C*H_2*)*, 30.4 (*C*H_2), 30.4 (*C*H_2), 37.2 (*C*HC(O)O), 74. 137.6 (Ar*C*), 3 (C(O)O*C*HCH_3), 126.1 (Ar*C*H), 127.2 (Ar*C*H), 128.6 (Ar*C*H), 129.7 (ArCH=*C*H), 130.8 (Ar*C*H=CH), 175.6 (*C(*O)) ppm; HRMS calcd. for $C_{16}H_{21}O_2$ $[M+H]^+$ 245.1536, found 245.1526.

Note: The more toxic HMPA is not necessary and can be substituted by DMPU, maintaining a similar reaction efficiency as to the original study.[13] However, the absence of DMPU, or adding DMPU to LDA before deprotonating the substrate, resulted in significantly lower yields for the desired product.

***Rac*–methyl (3*R*,6*S*)–6–methyl–2–oxo–3–((*E*)–4–phenylbut–3–en–1–yl)tetrahydro–2*H*–pyran–3–carboxylate–*^{13}C* (1a–*^{13}C*) and *Rac*–methyl (3*R*,6*R*)–6–methyl–2–oxo–3–((*E*)–4–phenylbut–3–en–1–yl)tetrahydro–2*H*–pyran–3–carboxylate–*^{13}C* (1a'–*^{13}C*)**

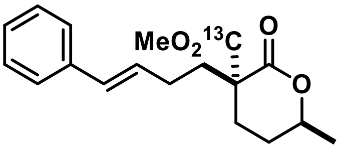

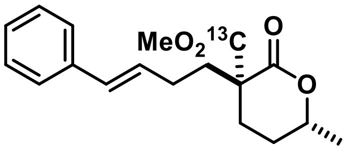

To a flame–dried flask was added was added diisopropylamine (0.14 mL, 101 mg, 1.0 mmol, 2.0 eq.) and dry THF (2 ml) under N_2. The solution was cooled to 0 °C, then *n*-BuLi (0.4 mL, 2.5 M in THF, 1.0 mmol,

2.0 eq.,) was added dropwise and the solution was stirred at 0 °C for 15 min. The solution was then cooled to –78 °C and (*E*)–6–methyl–3–(4–phenylbut–3–en–1–yl)tetrahydro–2*H*–pyran–2–one **S32** (122 mg, 0.5 mmol, 1.0 eq.) in THF (2.5 mL was added dropwise over 5 minutes. The mixture was stirred for 1 h at –78 °C and $^{13}CO_2$ was bubbled through the reaction mixture using a balloon and a reaction bubbler to saturate the solution with $^{13}CO_2$ and to replace N_2 in the headspace. After 5 min of bubbling, the bubbler was removed but the balloon was left in place to maintain an atmosphere of $^{13}CO_2$. After stirring for 30 min at –78 °C, $TMSCHN_2$ (2.5 mL, 2.0 M in hexanes, 5.0 mmol, 10 eq.) and dry MeOH (0.61 mL, 15 mmol, 30 eq.) were added and the reaction was stirred for another 2 h at –78 °C before it was allowed to slowly warm to room temperature overnight. The reaction was quenched by the addition of glacial acetic acid until the yellow colour of $TMSCHN_2$ disappeared and the bubbling stopped, then sat. aq. $NaHCO_3$ solution (25 ml) was added. The aqueous phase was extracted with EtOAc (3 × 50 ml). The organic layers were combined, dried ($MgSO_4$) and concentrated. The crude product was purified by silica gel column chromatography (Hexane/Acetone 85:15 to hexane/Et_2O 1:1) to yield separate isolated samples of the title products **1a–*^{13}C*** (0.094 mmol, 28.5 mg, 19%) as a colourless oil and **1a'–*^{13}C*** (0.104 mmol, 31.6 mg, 21%) as a colourless oil.

1a–*^{13}C*: ^1H NMR (400 MHz, $CDCl_3$) δ 1.29 (d, *J* = 6.2 Hz, 3H, CHC*H*$_3$) 1.56, (ddt, *J* = 14.1, 10.6, 8.0 Hz, 1H, OCHC*H*$_a$H$_b$), 1.69–1.81 (m, 1H, OCHCH$_2$C*H*$_a$H$_b$), 1.87–1.99 (m, 2H, CH=CHCH$_2$C*H*$_a$H$_b$ + OCHCH$_a$*H*$_b$), 2.02–2.10 (m, 1H, CH=CHCH$_2$CH$_a$*H*$_b$), 2.10–2.18 (m, 1H, CH=CHC*H*$_a$H$_b$), 2.18–2.29 (m, 1H, CH=CHCH$_a$*H*$_b$), 2.47 (dtd, *J* = 13.2, 8.0, 4.9 Hz, 1H, OCHCH$_2$CH$_a$*H*$_b$), 3.70 (d, *J* = 3.9 Hz, 3H, OC*H*$_3$), 4.21–4.34 (m, 1H, OC*H*CH$_3$), 6.10 (dt, *J* = 15.8, 6.5 Hz, 1H, ArCH=C*H*), 6.34 (dd, *J* = 16.0, 1.6 Hz, 1H, ArC*H*=CH), 7.08–7.15 (m, 1H, ArC*H*), 7.18–7.28 (m, 4H, ArC*H*) ppm; ^{13}C NMR (101 MHz, $CDCl_3$) δ 21.5 (CH*C*H$_3$), 27.1 (d, *J* = 1.5 Hz, OCHCH$_2$*C*H$_2$), 27.8 (OCH*C*H$_2$), 28.1 (d, *J* = 1.9 Hz CH=CH*C*H$_2$), 36.1 (d, *J* = 1.6 Hz CH=CHCH$_2$*C*H$_2$), 52.9 (d, *J* = 55.8 Hz, *C*$_q$), 53.2 (d, *J* = 2.9 Hz CO$_2$*C*H$_3$), 74.9 (O*C*H), 126.1 (Ar*C*H), 127.2 (Ar*C*H), 128.6 (Ar*C*H), 129.2 (ArCH=*C*H), 130.8 (Ar*C*H=CH), 137.5 (Ar*C*), 170.9 (d, *J* = 1.6 Hz, *C*(O)OCH), 171.8 (*C*O$_2$CH$_3$) ppm; HRMS calcd. for $C_{17}{}^{13}CH_{23}O_4$ $[M+H]^+$ 304.1624, found 304.1622.

1a'–*^{13}C*: ^1H NMR (400 MHz, $CDCl_3$) δ 1.31 (d, *J* = 6.3 Hz, 3H, OCHC*H*$_3$), 1.62 (tdd, *J* = 14.0, 11.4, 3.6 Hz, 1H, CH=CHC*H*$_a$H$_b$), 1.77–1.93 (m, 2H, CO$_2$CHC*H*$_2$), 1.93–2.04 (m, 1H, CH=CHCH$_2$C*H*$_a$H$_b$), 2.07–2.23 (m, 3H, CH=CHCH$_2$CH$_a$*H*$_b$ + CO$_2$CHCH$_2$C*H*$_2$), 2.24–2.37 (m, 1H, CH=CHCH$_a$*H*$_b$), 3.69 (d, *J* = 3.9 Hz, 3H, CO$_2$C*H*$_3$), 4.38 (ddp, *J* = 12.5, 6.3, 3.2 Hz, 1H, OC*H*CH$_3$), 6.09 (dt, *J* = 15.7, 6.6 Hz, 1H, ArCH=C*H*), 6.34 (d, *J* = 15.9 Hz, 1H, ArC*H*=CH), 7.07–7.16 (m, 1H, ArC*H*), 7.18–7.28 (m, 4H, ArC*H*) ppm; ^{13}C NMR (101 MHz, $CDCl_3$) δ 22.1 (CH*C*H$_3$), 28.4 (d, *J* = 2.8 Hz, *C*H$_2$), 28.4 (*C*H$_2$), 29.3 (d, *J* = 1.7 Hz, *C*H$_2$), 36.0 (d, *J* = 1.8 Hz, *C*H$_2$), 53.1 (d, *J* = 2.9 Hz, *C*$_q$), 53.7 (d, *J* = 55.7 Hz, CO$_2$*C*H$_3$), 78.6 (O*C*H), 126.1 (Ar*C*H), 127.2 (Ar*C*H), 128.6 (Ar*C*H), 129.2 (ArCH=*C*H), 130.9 (Ar*C*H=CH), 137.5 (Ar*C*), 169.9 (d, *J* = 1.9 Hz, *C*O$_2$CH), 172.2 (*C*O$_2$CH$_3$) ppm; HRMS calcd. for $C_{17}{}^{13}CH_{23}O_4$ $[M+H]^+$ 304.1624, found 304.1622.

***Rac*–methyl (*R*)–2–benzyl–4–((3*S*,6*S*)–6–methyl–2–oxotetrahydro–2*H*–pyran–3–yl)butanoate–1–*^{13}C* (3a–*^{13}C*)**

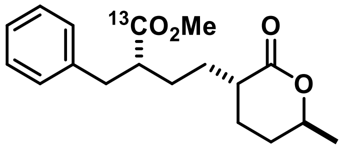

Prepared according to General Procedure E using SmI_2 (2.35 mL, 0.1 M in THF, 0.235 mmol, 2.5 eq.), HMPA (0.16 mL, 0.939 mmol, 10 eq.), H_2O (27 µL, 1.5 mmol, 16 eq.) and *rac*–methyl (3*R*,6*S*)–6–methyl–2–oxo–3–((*E*)–4–phenylbut–3–en–1–yl)tetrahydro–2*H*–pyran–3–carboxylate–^{13}C **1a–*^{13}C*** (28.9 mg, 0.0939

mmol, 1.0 eq.). Purification by silica gel column chromatography yielded the title product as a colourless oil, as a 3:1 mixture of diastereoisomers (19.4 mg, 0.0636 mmol, 68%). Major diastereoisomer: ^{1}H NMR (400 MHz, $CDCl_3$) δ 1.37 (d, *J* = 6.3 Hz, 3H, $CHCH_3$), 1.43–1.62 (m, 4H, $ArCH_2CHCH_aH_b$ + $CO_2CHCH_2CH_aH_b$ + $CO_2CHCH_aH_b$ + $CH_aH_bCH_2CHCO_2CH_3$), 1.75 (ddd, *J* = 11.8, 8.2, 3.8 Hz, 1H, $ArCH_2CHCH_aH_b$), 2.11–1.88 (m, 3H, $CO_2CHCH_aH_b$ + $CH_aH_bCH_2CHCO_2CH_3$ + $CO_2CHCH_2CH_aH_b$), 2.31 (ddtd, *J* = 11.0, 6.7, 4.4, 2.5 Hz, 1H, $CHCO_2CH$), 2.69 (dqd, *J* = 11.5, 8.3, 7.5, 5.5 Hz, 1H, $CHCO_2CH_3$), 2.79 (app. ddt, *J* = 13.2, 9.9, 6.3 Hz, 1H, $ArCH_aH_b$), 2.96 (app. dtd, *J* = 15.2, 7.7, 3.3 Hz, 1H, $ArCH_aH_b$), 3.62 (d, *J* = 3.9 Hz, 3H, CO_2CH_3), 4.41 (ddtt, *J* = 15.5, 12.4, 6.2, 2.9 Hz, 1H, OC*H*), 7.13–7.18 (m, 2H, ArC*H*), 7.19–7.25 (m, 1H, ArC*H*), 7.25–7.37 (m, 2H, ArC*H*) ppm; ^{13}C NMR (101 MHz, $CDCl_3$) δ 22.2 ($CHCH_3$), 25.7 ($CO_2CHCH_2CH_2$), 29.3 (d, *J* = 1.5 Hz, $ArCH_2CHCH_2$), 29.8 (d, *J* = 1.8 Hz, $ArCH_2CHCH_2CH_2$), 30.8 (CO_2CHCH_2), 38.6 (d, *J* = 1.5 Hz, $ArCH_2$), 40.5 ($CHCO_2CH$), 47.5 (d, *J* = 56.9 Hz, $CHCO_2CH_3$), 51.6 (d, *J* = 2.8 Hz, CO_2CH_3), 77.9 (CO_2CH), 126.5 (ArCH), 128.5 (ArCH), 128.9 (ArCH), 139.1 (d, *J* = 2.4 Hz, ArC), 173.5 (CO_2CH), 175.8 (CO_2CH_3), ppm; HRMS calcd. for $C_{17}{}^{13}CH_{25}O_4$ $[M+H]^+$ 306.1781, found 306.1770.

5.2.2 Discussion

A plausible alternative mechanism for the reaction is outlined in Figure S2. This mechanism would proceed through cyclisation of a radical species formed from the lactone carbonyl, rather than the acyclic ester. Following cyclisation of the radical onto the alkene acceptor, the hemiacetal could collapse to give alkoxide **III**. Lactonisation onto the ester group would give spirocyclic intermediate **IV**, by elimination of a methoxy group, which could then attack the ketone carbonyl to give **V**. Collapse of this intermediate by extrusion of the lactone enolate followed by protonation would generate product **3'**. Conformational analysis suggests that the major diastereoisomer formed by this mechanism would be opposite to the experimentally observed selectivity, however, further investigation is needed to confirm cyclisation, and therefore migration of the acyclic ester group. ^{13}C–Labelling studies allow the two possible mechanisms to be distinguished; the acyclic ester functionality in the product originates from the ester carbonyl in our proposed mechanism, whereas in mechanism shown in Figure S2 it would arise from the lactone carbonyl in the substrate.

Figure S2: Alternative mechanism

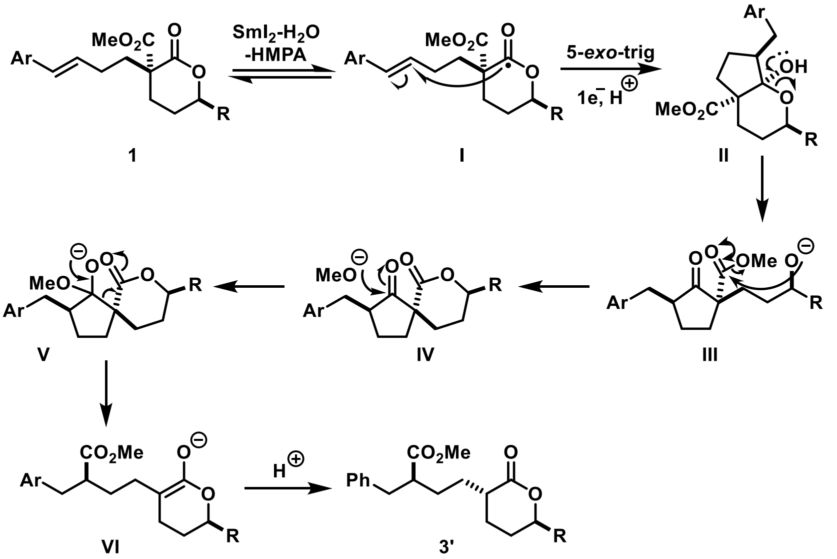

By analysing the ^{1}H and ^{13}C NMR spectra of the ^{13}C labelled product **3a–*^{13}C*** we were able to confirm that the labelled carbon in the product is at the acyclic ester. Therefore, we conclude that the mechanism shown

in Scheme 3C is in operation. Assignment of the position of the labelled carbon was achieved based on: (1) a doublet for the ester CH_3 peak in the ^{1}H NMR, (2) a doublet for the carbon adjacent to the ester in the ^{13}C NMR, and (3) a singlet for the carbon adjacent to the lactone in the ^{13}C NMR. Observations (1) and (2) indicate that the ester migration is indeed in operation, and observation (3) rules out a scenario in which both mechanisms are in action.

(1) The OCH_3 of the methyl ester in **3a–*^{13}C*** appears as a doublet in the ^{1}H NMR spectrum, indicating a high abundance of ^{13}C at the Me ester's carbonyl position.

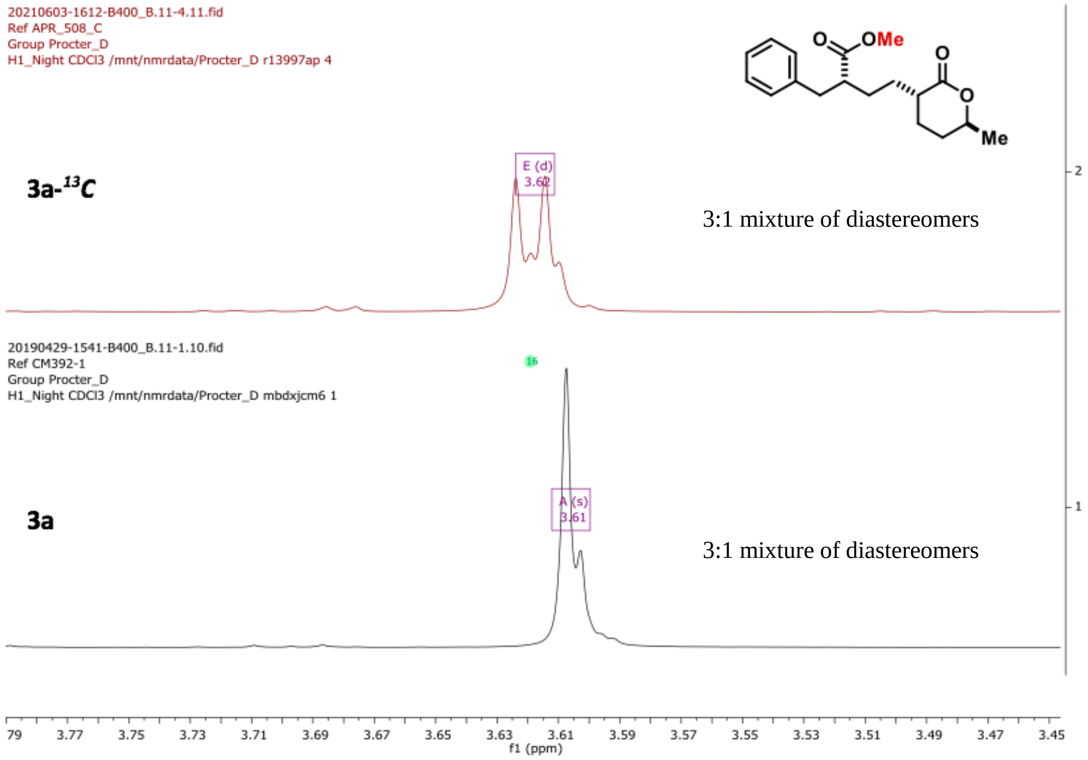

(2) In the ^{13}C NMR spectrum of **3a–*^{13}C*** the carbon adjacent to the Me ester's carbonyl carbon appears as a doublet with a large coupling constant (J = 56.9 Hz) indicative of a ^{13}C labelled carbon on an adjacent atom.[15] The labelled compound's multiplicity–edited ^{1}H–^{13}C HSQC spectrum shows that both peaks in the ^{13}C spectra correspond to the same proton in the ^{1}H spectrum, and no other proton shows correlation with these ^{13}C peaks.

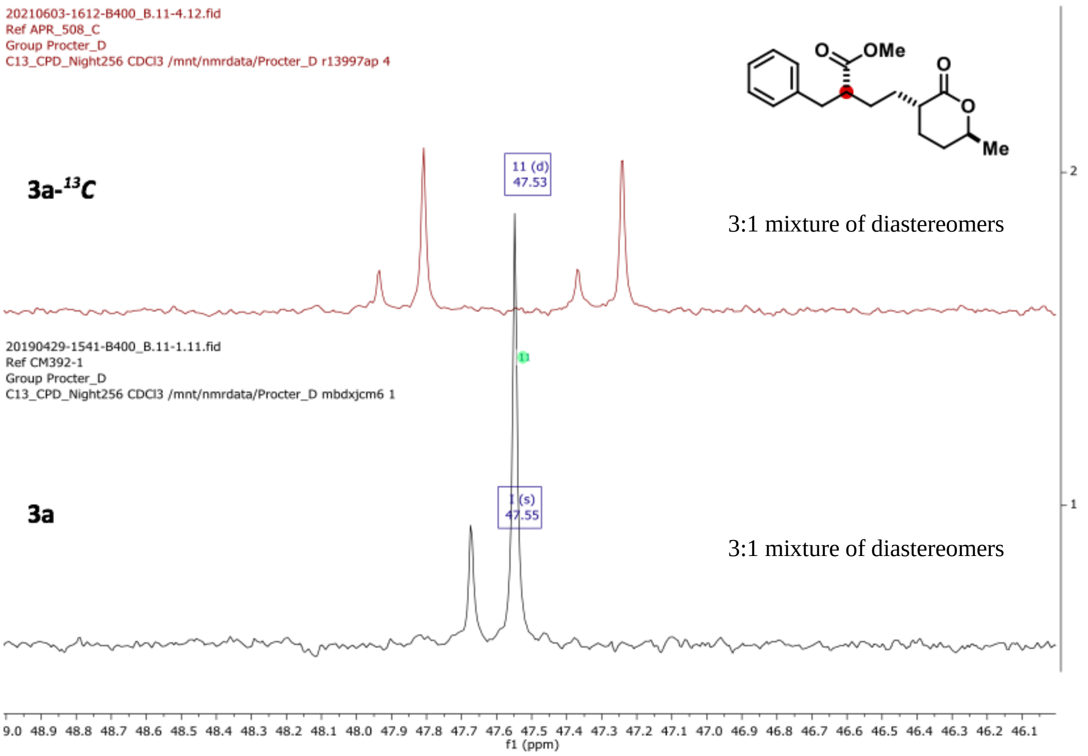

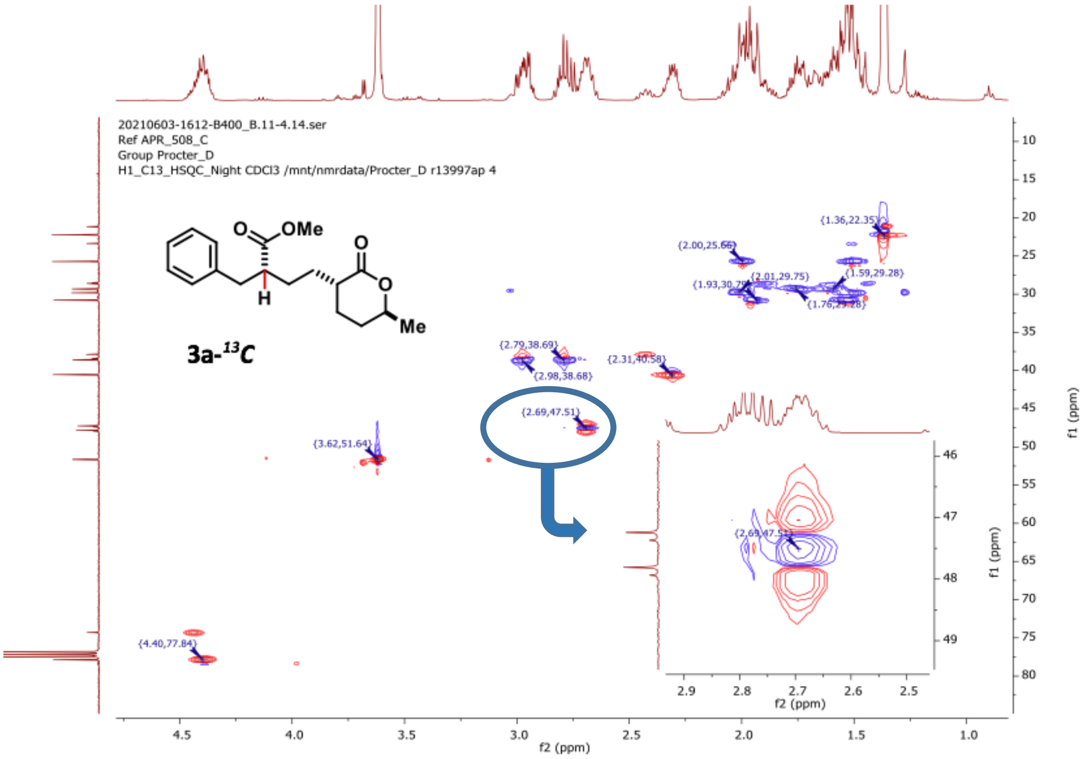

(3) The carbon atom adjacent to the lactone carbonyl appears as a singlet in the ^{13}C spectrum confirming that ^{13}C is only present at a low abundance at the lactone functionality.

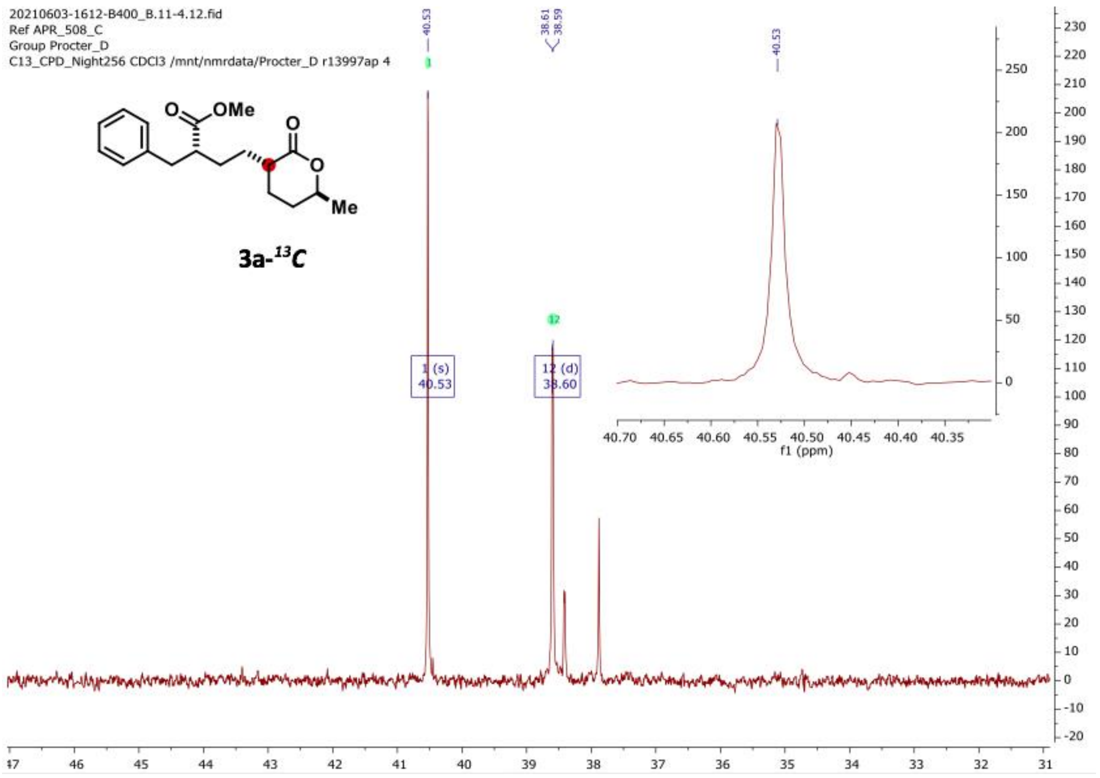

5.3 Experimental Data for Scheme 3B: Reaction using a Malonate Derivative

Dimethyl (*E*)–2–methyl–2–(4–phenylbut–3–en–1–yl)malonate (4)

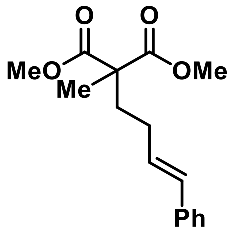

To a solution of NaH (21 mg, 0.84 mmol, 1.2 eq.) in THF (0.3 mL) was added dropwise a solution of dimethyl (*E*)–2–(4–phenylbut–3–en–1–yl)malonate **S2** (0.20 g, 0.76 mmol, 1.0 eq.) in THF (0.3 mL). After 30 minutes, methyl iodide (0.10 mL, 1.5 mmol, 2.0 eq.) was added and the reaction was stirred for a further 18 h. The reaction was quenched by the addition of sat. aq. NH_4Cl (1 mL) and the aqueous was extracted with EtOAc (3 × 3 mL). The combined organic layers were washed with brine (3 mL), dried ($MgSO_4$) and concentrated. Purification by silica gel column chromatography (hexane/EtOAc 95:5) yielded the title product as a colourless oil (118 mg, 0.430 mmol, 56%). ^{1}H NMR (400 MHz, $CDCl_3$) δ 1.48 (s, 3H, CCH_3), 2.01–2.13 (m, 2H, CH_2CH_2CH=CHAr), 2.13–2.30 (m, 2H, CH_2CH_2CH=CHAr), 3.73 (s, 6H, CO_2CH_3), 6.18 (dt, *J* = 15.8, 6.6 Hz, 1H, C*H*=CHAr), 6.41 (d, *J* = 15.9 Hz, 1H, CH=C*H*Ar), 7.18–7.24 (m, 1H, ArC*H*), 7.28–7.36 (m, 4H, ArC*H*) ppm; ^{13}C NMR (101 MHz, $CDCl_3$) δ 20.1 (CCH_3), 28.0 (CH_2CH_2CH=CHAr), 35.2 (CH_2CH_2CH=CHAr), 52.5 (CO_2CH_3), 53.4 (C_q), 126.0 (Ar*C*H), 127.0 (Ar*C*H), 128.5 (Ar*C*H), 129.4 (*C*H=CHAr), 130.5 (CH=*C*HAr), 137.5 (Ar*C*), 172.7 (CO_2CH_3) ppm; IR ν_{max} (thin film, cm^{-1}) = 2997, 1732 (C=O), 1435, 1234, 1112, 966; HRMS calcd. for $C_{16}H_{20}O_4Na$ $[M+Na]^+$ 299.1254, found 299.1240.

Methyl 3–benzyl–2–hydroxy–1–methylcyclopentane–1–carboxylate (5)

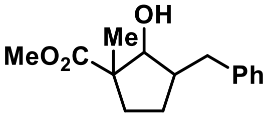

Prepared according to General Procedure E using SmI_2 (7.5 mL, 0.1 M in THF, 0.75 mmol, 2.5 eq.), HMPA (0.51 mL, 3.0 mmol, 10 eq.), H_2O (87 µL, 4.8 mmol, 16 eq.) and dimethyl (*E*)–2–methyl–2–(4–phenylbut–3–en–1–yl)malonate **4** (83 mg, 0.3 mmol 1.0 eq.). Purification by silica gel column chromatography (hexane/Et_2O 60:40) yielded an isolated sample of the major diastereoisomer of the title product as a colourless oil (21.7 mg, 0.0875 mmol, 29%), a second fraction containing the three minor diastereoisomers, as a 7.7:1.6:1 mixture (11.0 mg, 0.0443 mmol, 15%) in addition to a third fraction containing recovered starting material (36 mg, 0.129 mmol, 43%). Major diastereoisomer: ^1H NMR (400 MHz, $CDCl_3$) δ 1.25–1.32 (m, 1H $CCH_2CH_aH_b$), 1.33 (s, 3H, CCH_3), 1.49 (ddd, *J* = 13.7, 9.2, 4.8 Hz, 1H, CCH_aH_b), 1.67–1.79 (m, 1H, $CCH_2CH_aH_b$), 2.11–2.25 (m, 2H, C*H*CHOH + CCH_aH_b), 2.49 (dd, *J* = 13.5, 9.6 Hz, 1H, CH_aH_bAr), 3.02 (d, *J* = 8.6 Hz, 1H, O*H*), 3.07 (dd, *J* = 13.5, 4.8 Hz, 1H, CH_aH_bAr), 3.43 (t, *J* = 8.5 Hz, 1H, C*H*OH), 3.70 (s, 3H, CO_2CH_3), 7.19 (d, *J* = 7.3 Hz, 3H, ArC*H*), 7.24–7.32 (m, 2H, ArC*H*) ppm; ^{13}C NMR (101 MHz, $CDCl_3$) δ 23.4 (CCH_3), 26.4 (CCH_2CH_2), 33.6 (CCH_2), 40.2 (CH_2Ar), 47.9 (*C*HCHOH), 52.1 (CO_2CH_3), 52.2 (C_q), 85.7 (*C*HOH), 126.0 (Ar*C*H), 128.4 (Ar*C*H), 129.0 (Ar*C*H), 141.0 (Ar*C*), 177.9 (*C*(O)OCH_3) ppm; HRMS calcd. for $C_{15}H_{20}O_3Na$ $[M+Na]^+$ 271.1305, found 271.1297.

Methyl (E)-2,2-dimethyl-6-phenylhex-5-enoate (S37)

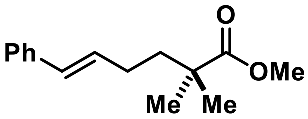

To a solution of i-Pr_2NH (1.2 eq., 3.6 mmol, 364 mg, 0.5 ml) in dry THF (10 ml) under dry N_2 was added n-BuLi (1.2 eq., 3.6 mmol, 1.56 ml, 2.3 M in hexanes) at –78 °C. The mixture was stirred for 30 min before methyl isobutyrate (1.0 eq., 3.0 mmol, 306 mg, 0.34 ml) was added neat, then stirring was continued for another 30 min at –78 °C. Next, (*E*)–(4–iodobut–1–en–1–yl)benzene[14] (1.2 eq., 3.6 mmol, 929 mg) in THF (2 ml) was added dropwise, the mixture was stirred at –78 °C for 30 min before warming to r.t. and stirring for a further 5 h. The reaction mixture was quenched by H_2O (25 ml) and Et_2O (25 ml). The phases were separated then the aqueous phase was extracted by Et_2O (2 × 25 ml). The combined organic layers were dried ($MgSO_4$) and concentrated. Purification by silica gel column chromatography (hexanes/Et_2O 95:5) yielded the title product as a colourless oil (549 mg, 2.36 mmol, 79%). ^1H NMR (400 MHz, $CDCl_3$) δ 7.37 – 7.26 (m, 4H, Ar*H*), 7.23 – 7.16 (m, 1H, Ar*H*), 6.38 (dt, *J* = 16.0, 1.6 Hz, 1H, C*H*=$CHCH_2$), 6.18 (dt, *J* = 15.8, 6.8 Hz, 1H, CH=C*H*CH_2), 3.66 (s, 3H, OCH_3), 2.25 – 2.09 (m, 2H, CH=$CHCH_2$), 1.80 – 1.64 (m, 2H, CH=$CHCH_2CH_2$), 1.22 (s, 6H, $C(CH_3)_2$). ^{13}C NMR (101 MHz, $CDCl_3$) δ 178.4 (CO_2Me), 137.8 (Ar*C*), 130.4 (CH=*C*HCH_2), 130.1 (*C*H=$CHCH_2$), 128.6 (Ar*C*H), 127.0 (Ar*C*H), 126.0 (Ar*C*H), 51.9 (OCH_3), 42.3 (*C*$(Me)_2$), 40.4 ($CH_2C(Me)_2$), 28.8 (CH=$CHCH_2$), 25.4 ($C(CH_3)_2$). HRMS calcd. for $C_{15}H_{21}O_2$ $[M+H]^+$ 233.1536, found 223.1535. IR v_{max} (thin film, cm^{-1}) = 3025, 2972, 2948, 1728, 1473, 1448.

7. DFT Studies

6.1 Lowest energy conformations of the lactone substrates

6.1.1 Background

In order to establish the lowest energy conformation of the lactone substrates **1-H** and **1a**, six initial geometry optimisations were run for each substrate – two chair-, two half-chair- and two boat conformations – in order to account for the 6 major possible conformations of the lactone substrates.

6.1.2 Results

In order to obtain the lowest energy conformation of each lactone, several more geometry optimisations were calculated in order to fine-tune the six initial calculations. As a result, for both **1-H** and **1a**, two half chair (**1-Hh1** + **1-Hh2** and **1ah1** + **1ah2**, respectively) conformations could be located (one more energetically favourable than the other), whilst only one chair (**1-Hc** and **1ac**, respectively) conformation could be located for both lactones. Similarly, only one boat conformation could be located for lactone **1a**, whilst two were located for lactone **1-H** (**1ab1** and **1-Hb1** + **1-Hb2**, respectively). Thermodynamic corrections to the SCF energies identified half chair **1-Hh1** as the lowest energy conformation of lactone **1-H**, whilst boat **1ab1** was identified as the lowest energy conformation of lactone **1a**.

1-H:

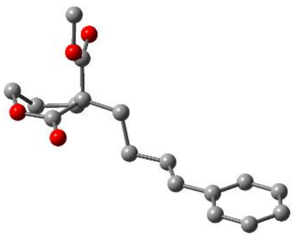

1-Hb1

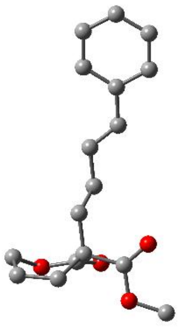

1-Hb2

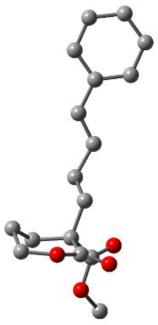

1- Hh2

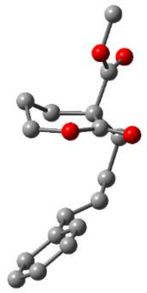

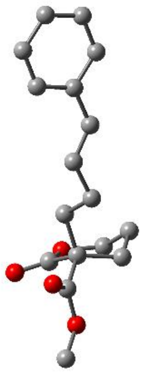

1-Hh1 (Lowest Energy) **1-Hc**

***1a*:**

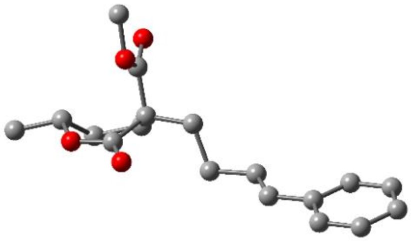

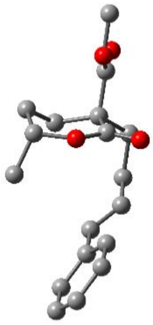

1ab1 (Lowest Energy) **1ah1**

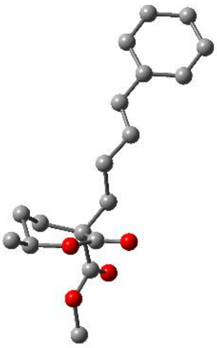

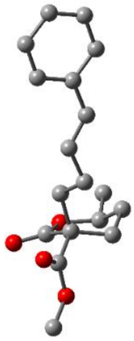

1ah2 **1ac**

6.1.3 Computational Details

In this study, geometry optimisation calculations were performed (using Gaussian 16, Revision C.01.)[16] using the PBE0 functional[17] and Tom Dunning's correlation-consistent double-zeta + polarisation basis sets for C, H and O.[18] The geometry optimisation calculations were run with the Grimme's D3 model[19] with Becke-Johnson damping factors.[20] Harmonic vibrational frequencies calculations were performed, to confirm the stationary points as true minima and to provide thermodynamic corrections to the SCF energies. Further single point energy calculations using Tom Dunning's correlation-consistent triple-zeta + polarisation basis sets for C, H and O[18] and corrections for solvation (PCM in THF (ε = 7.6)[21]) were then run on the optimised geometries.

6.1.4 Breakdown of Energy Contributions

Table S.1: *Determination of the most stable boat and half chair conformations for each lactone. Breakdown of energy contributions detailing the evolution of relative energies as the successive corrections to the initial SCF energy are included. Energies in kJ mol^{-1}:*

ΔG: Free energy at 298.15 K and 1 atm

$\Delta G_{cc\text{-}pVTZ}$: Free energy from cc-pVTZ single point calculations, with thermodynamic corrections from the cc-pVDZ frequencies calculations

$\Delta G_{Finalcc\text{-}pVTZ+Solv}$: Free energy from cc-pVTZ single point calculations (with corrections for THF solvent), with thermodynamic corrections from the cc-pVDZ frequencies calculations. These are the data used to select the most stable conformation.

Label	ΔG	$\Delta G_{cc\text{-}pVTZ}$	$\Delta G_{Finalcc\text{-}pVTZ+Solv}$
1-Hb1* vs *1-Hb2	-11.8	-11.7	-9.9
1-Hh1* vs *1-Hh2	-10.6	-5.2	-0.3
1ah1 vs 1ah2	-1.6	+2.6	+7.5

Table S.2: *Determination of the overall most stable conformation for each lactone. The most stable boat, half chair and chair conformations were used for these calculations. Breakdown of energy contributions detailing the evolution of relative energies as the successive corrections to the initial SCF energy are included. Energies in kJ mol^{-1}:*

ΔG: Free energy at 298.15 K and 1 atm

$\Delta G_{cc\text{-}pVTZ}$: Free energy from cc-pVTZ single point calculations, with thermodynamic corrections from the cc-pVDZ frequencies calculations

$\Delta G_{Finalcc\text{-}pVTZ+Solv}$: Free energy from cc-pVTZ single point calculations (with corrections for THF solvent), with thermodynamic corrections from the cc-pVDZ frequencies calculations.

Label	ΔG	$\Delta G_{cc\text{-}pVTZ}$	$\Delta G_{Final cc\text{-}pVTZ+Solv}$
1-Hb1 **vs** ***1-Hh1***	+4.8	+0.5	-1.9
1-Hb1 **vs** ***1-Hc***	-5.8	-4.7	-2.2
1-Hh1 vs 1-Hc	-10.6	-5.2	-0.3
1ab1 vs 1ah2	-3.0	-2.8	-0.7
1ab1 vs 1ac	-5.0	-3.8	-1.5
1ah2 vs 1ac	-2.0	-1.1	-0.9

6.1.5 Cartesian Coordinates (Å) and computed energies (in Hartrees) for all optimised structures.

1-Hb1

SCF Energy = -959.764477

Free Energy (298K) = -959.474948

Free Energy (cc-pVTZ, PCM(THF)) = -959.7399411

C -3.950538 -1.805285 -1.026820

C -2.667351 -1.074273 -1.440232

C -2.122031 -0.163870 -0.324743

C -4.564643 -1.163443 0.197886

H -1.897580 -1.803175 -1.723266

H -2.846495 -0.426927 -2.309988

H -4.880058 -0.124275 0.001008

H -3.733245 -2.855932 -0.776893

H -4.685335 -1.813935 -1.845618

C -2.386690 -0.748480 1.064519

O -1.566223 -0.829314 1.939083

O -3.639242 -1.196479 1.286768

C -2.827247 1.195519 -0.417161

O -3.313702 1.659745 -1.419269

O -2.782159 1.838833 0.752997

C -3.343693 3.150315 0.753242

H -3.230155 3.519827 1.777816

H -2.808250 3.798288 0.045172

H -4.404843 3.116133 0.468302

C -0.623453 0.161676 -0.496871

H -0.510485 0.684496 -1.462232

H -0.337799 0.871030 0.293325

C 0.332236 -1.032074 -0.442668

H 0.243284 -1.523578 0.535548

H 0.055510 -1.770643 -1.215397

C 1.743857 -0.599358 -0.673631

H 1.964569 -0.173513 -1.660000

C 2.712562 -0.676970 0.249658

H 2.449355 -1.090193 1.230401

C 4.111258 -0.261055 0.107998

C 4.957957 -0.344808 1.223290

C	4.654114	0.221040	-1.094538
C	6.293081	0.042265	1.147984
H	4.553793	-0.719817	2.166920
C	5.986176	0.607620	-1.171825
H	4.026467	0.290016	-1.984825
C	6.814243	0.521684	-0.051075
H	6.929789	-0.032037	2.031983
H	6.386223	0.978407	-2.117923
H	7.860833	0.825315	-0.115783
H	-5.443996	-1.714781	0.554736

1-Hb2

SCF Energy = -959.7602542

Free Energy (298K) = -959.470446

Free Energy (cc-pVTZ, PCM(THF)) = -959.7361735

C	2.247151	0.115045	-0.336818
C	3.106399	0.739542	-1.452551
C	3.236982	2.263097	-1.314307
C	2.833877	2.754518	0.070034
H	1.749191	2.935152	0.133445
H	4.100705	0.277299	-1.405666
H	2.675156	0.465701	-2.427764
H	2.614272	2.790052	-2.053739
H	4.277516	2.554134	-1.518853
H	3.335446	3.696901	0.323315
O	3.202142	1.850491	1.113127
C	2.780944	0.574002	1.022216
O	2.861992	-0.149863	1.978641
C	2.405478	-1.406769	-0.347284
O	1.498893	-2.199940	-0.399975
O	3.695297	-1.757411	-0.314215
C	3.936162	-3.158836	-0.218172
H	5.025493	-3.274304	-0.219652
H	3.508944	-3.552707	0.714793
H	3.487016	-3.690717	-1.068704
C	0.753857	0.453714	-0.515351
H	0.375445	-0.197431	-1.317767
H	0.649105	1.488155	-0.877768
C	-0.111878	0.252900	0.729121
H	0.008975	-0.777328	1.091979
H	0.242568	0.920910	1.535197
C	-1.550409	0.537907	0.446533
H	-1.796680	1.572615	0.177626
C	-2.518666	-0.388669	0.471369
H	-2.232394	-1.417059	0.720739
C	-3.943387	-0.192292	0.186662
C	-4.783186	-1.314438	0.129288
C	-4.518032	1.070480	-0.032977
C	-6.141771	-1.187090	-0.146443
H	-4.354663	-2.304813	0.302200
C	-5.873629	1.199799	-0.307957
H	-3.896787	1.966332	0.020913
C	-6.694159	0.071906	-0.368064
H	-6.772527	-2.077531	-0.187027
H	-6.298413	2.192229	-0.473181
H	-7.759354	0.177304	-0.582494

1-Hh1

SCF Energy = -959.7710306

Free Energy (298K) = -959.476764

Free Energy (cc-pVTZ, PCM(THF)) = -959.7391995

C	-1.780285	0.548077	-0.093456
C	-0.037510	-1.405921	-1.356607
C	-1.243085	-0.884634	-2.100935
C	-1.541212	0.521475	-1.615938
H	0.860283	-0.810988	-1.595246

H	0.176151	-2.455483	-1.598774
H	-1.022338	-0.888416	-3.179625
H	-2.103346	-1.550166	-1.933652
H	-0.678554	1.163996	-1.846553
H	-2.412261	0.963182	-2.124566
O	-0.200583	-1.386396	0.064543
C	-0.893207	-0.430269	0.697104
O	-0.851579	-0.387832	1.902429
C	-3.226751	0.103319	0.144500
O	-4.171791	0.845952	0.226664
O	-3.330234	-1.232377	0.198090
C	-4.649148	-1.731269	0.417376
H	-5.319317	-1.426767	-0.398965
H	-4.550884	-2.821545	0.452884
H	-5.049353	-1.348173	1.366249
C	-1.635962	1.960441	0.500035
H	-2.475790	2.566266	0.131423
H	-1.766362	1.862726	1.587595
C	-0.309138	2.670370	0.204875
H	-0.253567	2.983612	-0.850294
H	-0.303471	3.599991	0.800151
C	0.881688	1.837557	0.548285
H	0.940278	1.482855	1.582037
C	1.823704	1.456502	-0.327907
H	1.779629	1.855642	-1.349660
C	2.933463	0.537994	-0.052101
C	4.068573	0.554409	-0.876019
C	2.883698	-0.392830	0.999237
C	5.136983	-0.306377	-0.642277
H	4.112962	1.263546	-1.706781
C	3.951707	-1.252543	1.230326
H	1.986728	-0.468039	1.617058
C	5.084263	-1.211306	0.416168
H	6.014715	-0.271654	-1.291023
H	3.893056	-1.973776	2.048041
H	5.918255	-1.891543	0.599907

1-Hh2

SCF Energy = -959.7635496

Free Energy (298K) = -959.472742

Free Energy (cc-pVTZ, PCM(THF)) = -959.7390863

C	2.190993	-0.094790	-0.032427
C	2.499047	-0.964140	-1.259867
C	2.286637	-2.426133	-0.916288
H	1.877489	-0.648022	-2.109976
H	3.547594	-0.804600	-1.554935
H	1.238897	-2.616615	-0.633178
H	2.510359	-3.071818	-1.779554
C	2.871041	-0.615759	1.238394
O	3.079637	0.106046	2.177445
O	3.124396	-1.932599	1.352750
C	2.720251	1.321628	-0.235914
O	2.066349	2.332654	-0.186810
O	4.035238	1.300079	-0.493540
C	4.644116	2.582853	-0.622903
H	4.537368	3.150474	0.312138
H	5.701108	2.390788	-0.837322
H	4.179365	3.153109	-1.439670
C	0.688623	-0.033842	0.318045
H	0.579672	0.717181	1.113923
H	0.376897	-0.996702	0.753396
C	-0.239786	0.315164	-0.845874
H	-0.247696	-0.491106	-1.597036
H	0.142009	1.226550	-1.334880
C	-1.634034	0.556030	-0.368073
H	-1.759849	1.403753	0.315206
C	-2.691230	-0.197127	-0.702542
H	-2.524679	-1.038661	-1.386172
C	-4.079381	-0.028635	-0.261987

C -5.054040 -0.917923 -0.738113

C -4.488697 0.984310 0.620936

C -6.386734 -0.804873 -0.351726

H -4.754520 -1.712812 -1.426015

C -5.817962 1.098802 1.007569

H -3.756828 1.693107 1.012072

C -6.775715 0.205412 0.524050

H -7.125168 -1.510683 -0.737639

H -6.112490 1.894287 1.695256

H -7.819299 0.298651 0.830483

C 3.215578 -2.798337 0.215009

H 4.263533 -2.778943 -0.131719

H 3.009039 -3.803239 0.607134

1-Hc

SCF Energy = -959.7635496

Free Energy (298K) = -959.472742

Free Energy (cc-pVTZ, PCM(THF)) = -959.7390863

C 3.215520 -2.798323 0.215200

C 2.191026 -0.094776 -0.032404

C 2.499190 -0.964158 -1.259804

C 2.286677 -2.426136 -0.916191

H 4.263497 -2.779016 -0.131469

H 1.877756 -0.648019 -2.109994

H 3.547784 -0.804671 -1.554730

H 1.238907 -2.616530 -0.633141

H 2.510429 -3.071889 -1.779396

C 2.871062 -0.615663 1.238433

O 3.079738 0.106197 2.177428

O 3.124328 -1.932527 1.352880

C 2.720163 1.321673 -0.236011

O 2.066148 2.332637 -0.187026

O 4.035153 1.300239 -0.493610

C 4.643925 2.583056 -0.623095

H 4.537286 3.150687 0.311953

H 5.700896 2.391052 -0.837669

H 4.179004 3.153263 -1.439797

C 0.688626 -0.033910 0.318007

H 0.579577 0.717307 1.113687

H 0.376978 -0.996688 0.753592

C -0.239759 0.314697 -0.846050

H -0.247708 -0.491884 -1.596874

H 0.142094 1.225866 -1.335417

C -1.633995 0.555818 -0.368338

H -1.759801 1.403933 0.314456

C -2.691196 -0.197527 -0.702369

H -2.524692 -1.039331 -1.385679

C -4.079326 -0.028831 -0.261824

C -5.054277 -0.917399 -0.738694

C -4.488337 0.983614 0.621813

C -6.386984 -0.804055 -0.352439

H -4.754983 -1.711929 -1.427108

C -5.817615 1.098399 1.008314

H -3.756197 1.691713 1.013703

C -6.775676 0.205779 0.523984

H -7.125655 -1.509289 -0.738954

H -6.111901 1.893474 1.696579

H -7.819266 0.299232 0.830330

H 3.008906 -3.803194 0.607363

1ab1

SCF Energy = -999.0406092

Free Energy (298K) = -998.725131

Free Energy (cc-pVTZ, PCM(THF)) = -999.0006837

C -3.682669 -1.476886 -1.247013

C -2.380872 -0.741784 -1.581258

C -1.822163 0.047188 -0.383946

C	-4.332276	-0.913838	0.002648
H	-1.626773	-1.459214	-1.928524
H	-2.538755	-0.012444	-2.387843
H	-4.556639	0.159402	-0.136390
H	-3.489925	-2.545814	-1.057073
H	-4.392199	-1.421519	-2.085832
C	-2.141308	-0.639968	0.944482
O	-1.344514	-0.824534	1.825852
O	-3.414062	-1.051740	1.103484
C	-5.594073	-1.632770	0.416107
H	-5.979054	-1.221852	1.359580
H	-6.367003	-1.520287	-0.357846
H	-5.393781	-2.704737	0.562052
C	-2.469805	1.438056	-0.370857
O	-2.912396	2.008621	-1.337869
O	-2.427952	1.974851	0.852057
C	-2.938025	3.302969	0.955520
H	-2.834799	3.577541	2.010599
H	-2.361296	3.987686	0.317942
H	-3.992592	3.336741	0.646992
C	-0.308241	0.321542	-0.502274
H	-0.153599	0.912060	-1.421733
H	-0.010232	0.955007	0.345774
C	0.596020	-0.912605	-0.524048
H	0.474051	-1.467748	0.415757
H	0.297604	-1.582570	-1.349528
C	2.027360	-0.526202	-0.711117
H	2.277382	-0.039788	-1.661867
C	2.981492	-0.712919	0.211734
H	2.689695	-1.183512	1.157931
C	4.398412	-0.350013	0.111404
C	5.226791	-0.543373	1.226838
C	4.976479	0.185259	-1.051577
C	6.578062	-0.210548	1.190123
H	4.795219	-0.961384	2.139763
C	6.324720	0.517834	-1.090335
H	4.363676	0.338621	-1.941666
C	7.134229	0.323119	0.030339
H	7.199908	-0.370092	2.073433
H	6.752123	0.931499	-2.006216
H	8.193591	0.584455	-0.004128

1ah1

SCF Energy = -999.0450135

Free Energy (298K) = -998.724602

Free Energy (cc-pVTZ, PCM(THF)) = -998.9975883

C	1.809504	0.591548	0.156277
C	0.514710	-1.956450	0.908728
C	1.595353	-1.310412	1.763645
C	1.559452	0.199353	1.619718
H	0.784903	-3.005913	0.713805
H	1.433149	-1.610948	2.809771
H	2.583500	-1.695541	1.469780
H	0.576383	0.583566	1.927862
H	2.315748	0.685396	2.254205
O	0.409526	-1.384944	-0.413698
C	0.933587	-0.218490	-0.807537
O	0.714257	0.159036	-1.933173
C	3.276066	0.268664	-0.146604
O	4.205698	0.954109	0.198942
O	3.416117	-0.890006	-0.803085
C	4.760418	-1.256522	-1.111955
H	5.348606	-1.379245	-0.191398
H	4.690454	-2.203580	-1.657480
H	5.234300	-0.484918	-1.734272
C	1.626667	2.099702	-0.096580
H	2.472361	2.613502	0.382395
H	1.719859	2.257907	-1.181176

C 0.310154 2.712673 0.394178
H 0.269264 2.729035 1.495450
H 0.314066 3.768843 0.071635
C -0.894807 2.020723 -0.151143
H -0.943494 1.930096 -1.240391
C -1.858525 1.459908 0.593399
H -1.825058 1.593043 1.682816
C -2.975101 0.658184 0.081342
C -4.138411 0.509543 0.849723
C -2.895369 -0.014587 -1.149486
C -5.204775 -0.260731 0.394385
H -4.205729 1.013907 1.817230
C -3.961378 -0.784282 -1.602151
H -1.974430 0.033487 -1.734877
C -5.121910 -0.908186 -0.836845
H -6.104518 -0.358206 1.005595
H -3.879189 -1.306355 -2.557761
H -5.954072 -1.517788 -1.194605
C -0.863992 -1.915382 1.538004
H -1.149354 -0.891399 1.817717
H -1.616894 -2.286378 0.829352
H -0.884497 -2.545601 2.439878

1ah2

SCF Energy = -999.0400592

Free Energy (298K) = -998.723995

Free Energy (cc-pVTZ, PCM(THF)) = -999.0004312

C -2.079405 0.506330 -1.440387
C -2.527952 1.904733 -1.062246
H -1.164809 0.531616 -2.050848
H -2.861253 0.052710 -2.068303
H -1.711423 2.476259 -0.588416
H -2.844230 2.462610 -1.957112
C -2.375980 0.167761 1.131025
O -2.043733 -0.320641 2.180640
O -3.231286 1.198739 1.136373
C -1.827311 -0.387840 -0.198259
C -2.570763 -1.705787 -0.401781
O -2.067433 -2.779371 -0.600998
O -3.899951 -1.502639 -0.350366
C -4.698580 -2.673382 -0.514472
H -4.458011 -3.413796 0.261047
H -5.737881 -2.340472 -0.419682
H -4.525214 -3.125094 -1.501615
C -0.338839 -0.699510 -0.004758
H 0.016990 -1.228182 -0.902030
H -0.245836 -1.399394 0.837042
C 0.521617 0.534965 0.257968
H 0.454664 1.248146 -0.580407
H 0.135470 1.050604 1.155785
C 1.953101 0.173079 0.483225
H 2.146197 -0.485954 1.337477
C 2.969231 0.580801 -0.289754
H 2.736110 1.234091 -1.139467
C 4.390666 0.256160 -0.135991
C 5.308821 0.779958 -1.058003
C 4.886518 -0.559798 0.894293
C 6.669663 0.502851 -0.960601
H 4.941767 1.417333 -1.866570
C 6.243973 -0.837446 0.993201
H 4.200723 -0.983724 1.629800
C 7.144508 -0.308310 0.066775
H 7.362638 0.923629 -1.692132
H 6.606052 -1.474567 1.802804
H 8.210558 -0.528820 0.148003
C -3.679390 1.813964 -0.085389
H -4.469333 1.166384 -0.506111
C -4.261194 3.148954 0.313455

H	-4.707618	3.645917	-0.560222
H	-5.039725	3.015365	1.077136
H	-3.478717	3.801725	0.728820

1ac

SCF Energy = -999.0398254

Free Energy (298K) = -998.723244

Free Energy (cc-pVTZ, PCM(THF)) = -999.0000963

C	3.177091	-2.451772	-0.045360
C	2.039159	0.243388	-0.072407
C	2.376086	-0.515324	-1.362193
C	2.224766	-2.006655	-1.134865
H	4.213246	-2.313416	-0.405494
H	1.737685	-0.162260	-2.184698
H	3.415059	-0.289768	-1.646904
H	1.188859	-2.263844	-0.857741
H	2.461267	-2.567810	-2.051978
C	2.758448	-0.343830	1.145357
O	2.954658	0.312140	2.134562
C	2.981039	-3.883851	0.392536
H	3.148525	-4.567067	-0.453061
H	3.684502	-4.140030	1.196468
H	1.957214	-4.033032	0.767667
O	3.064838	-1.653204	1.152811
C	2.498728	1.694597	-0.168424
O	1.799458	2.666287	-0.029666
O	3.808876	1.756896	-0.443768
C	4.354761	3.073525	-0.476081
H	4.237782	3.557884	0.503530
H	5.415747	2.950432	-0.719593
H	3.849909	3.685211	-1.237195
C	0.539863	0.204268	0.292961
H	0.403651	0.884703	1.146097
H	0.279755	-0.803842	0.653128
C	-0.416852	0.597510	-0.833189
H	-0.386431	-0.141723	-1.649867
H	-0.090341	1.566828	-1.244678
C	-1.818132	0.717491	-0.331010
H	-1.985899	1.495434	0.422673
C	-2.833986	-0.063462	-0.724691
H	-2.626604	-0.830737	-1.480713
C	-4.225270	-0.013747	-0.265025
C	-5.158326	-0.893792	-0.832880
C	-4.677221	0.874874	0.724541
C	-6.492150	-0.889651	-0.434098
H	-4.825059	-1.593304	-1.603849
C	-6.007690	0.880509	1.123810
H	-3.977598	1.570607	1.190907
C	-6.923957	-0.001111	0.547230
H	-7.197768	-1.585199	-0.893131
H	-6.335738	1.580061	1.895483
H	-7.968549	0.006122	0.864158

6.2 Lowest energy conformations of the lactone-derived ketyl radicals

6.2.1 Computational Details

In this study, geometry optimisation calculations were performed (using Gaussian 16, Revision C.01.)[16] using the PBE0 functional[17] and Tom Dunning's correlation-consistent double-zeta + polarisation basis sets for C, H and O.[18] For Samarium and Iodine, Stuttgart-Koln effective core potentials and associated valence basis sets were used.[22,23] The geometry optimisation calculations were run with the Grimme's D3 model[19] with Becke-Johnson damping factors.[20] Harmonic vibrational frequencies calculations were performed, to confirm the stationary points as true minima and to provide thermodynamic corrections to the SCF energies. Further single point energy calculations using Tom Dunning's correlation-consistent triple-zeta + polarisation basis sets for C, H and O[18] and corrections for solvation (PCM in THF (ε = 7.6)[21]) were then run on the optimised geometries. The ball and stick structures in the main text were created using GaussView 6.1.1.[24]

Our previous calculations[25] on systems of this type suggest that reactivity takes place *via* the quintet spin state, with antiparallel coupling of ligand radical anion to Sm(III) $4f^5$ metal centre. Hence all calculations have been performed with a spin multiplicity of 5.

6.2.2 Breakdown of Energy Contributions

Table S.3: *Breakdown of energy contributions detailing the evolution of relative energies as the successive corrections to the initial SCF energy are included. Energies in kJ mol^{-1}:*

ΔE: SCF energy computed from the PBE0/cc-pVDZ geometry optimisations

ΔG: Free energy at 298.15 K and 1 atm

$\Delta G_{cc\text{-}pVTZ}$: Free energy from cc-pVTZ single point calculations, with thermodynamic corrections from the cc-pVDZ frequencies (at 298.15 K) calculations

$\Delta G_{Finalcc\text{-}pVTZ+Solv(298.15\,K)}$: Free energy from cc-pVTZ single point calculations (with corrections for THF solvent), with thermodynamic corrections from the cc-pVDZ frequencies (at 298.15 K) calculations. These are the data used in the main article

$\Delta G_{Finalcc\text{-}pVTZ+Solv(195.15\,K)}$: Free energy from cc-pVTZ single point calculations (with corrections for THF solvent), with thermodynamic corrections from the cc-pVDZ frequencies (at 195.15 K) calculations.

Label	ΔE	ΔG	$\Delta G_{cc\text{-}pVTZ}$	$\Delta G_{Finalcc\text{-}pVTZ+Solv(298.15\,K)}$	$\Delta G_{Finalcc\text{-}pVTZ+Solv(195.15\,K)}$
***C*-I-Me vs *HC*-I-Me**	+16.4	-7.7	-2.85	-6.2	-9.1
***C*-I-H vs *HC*-I-H**	+28.2	-21.1	-16.5	-18.9	-21.5

6.2.3 **Cartesian Coordinates (Å)** and computed energies (in Hartrees) for all optimised structures.

C-***I-Me***

SCF Energy = -2376.7816685

Free Energy (298K) = -2376.143004

Free Energy (298K, cc-pVTZ, PCM(THF)) = -2376.604424

Sm	-1.027724	-0.095422	0.006330
I	-1.843750	1.125401	-2.713633
I	-0.687671	-1.522517	2.720790
O	-2.821520	-1.815643	-0.572994
C	-4.018588	-1.602120	-1.354349
C	-4.698490	-2.959968	-1.448651
C	-4.195519	-3.670365	-0.195877
C	-2.762191	-3.186902	-0.133743
O	-0.011504	-2.146691	-0.951825
C	0.014270	-2.347281	-2.380387
C	1.433234	-2.780995	-2.679472
C	1.751673	-3.637927	-1.458431
C	1.096450	-2.851748	-0.336994
O	-3.224285	0.690386	0.907001
C	-3.747611	2.016430	0.676768
C	-5.181495	1.953558	1.158866
C	-5.053651	1.024987	2.361826
C	-4.054439	-0.005374	1.865616
H	-4.635011	-0.859666	-0.822597
H	-3.728009	-1.171249	-2.321613
H	-2.112744	-3.754169	-0.822228
H	-2.312825	-3.193757	0.868725
H	-4.360606	-3.496756	-2.349286
H	-5.792558	-2.872613	-1.499228
H	-4.267720	-4.765339	-0.250952
H	-4.750178	-3.334503	0.695370
H	-0.716848	-3.135583	-2.636505
H	-0.291222	-1.408461	-2.862937
H	1.773262	-2.095393	0.084990
H	0.697521	-3.465268	0.481372
H	2.088718	-1.896426	-2.716191
H	1.516468	-3.319839	-3.633316
H	2.827171	-3.772491	-1.282113
H	1.290206	-4.633352	-1.560515
H	-3.146923	2.729035	1.262789
H	-3.626547	2.236081	-0.392831
H	-4.550733	-0.837261	1.339553
H	-3.402392	-0.421278	2.645205
H	-5.828343	1.505137	0.387205
H	-5.582021	2.945886	1.408121
H	-6.002592	0.565723	2.671182
H	-4.641973	1.573026	3.223987
O	-0.728107	2.139742	0.694803
C	0.260824	2.913040	0.693326
C	1.653315	2.448953	1.035448
O	0.154601	4.141793	0.189259
C	2.701564	3.565585	1.026369
C	1.592650	1.730668	2.395128
C	1.883140	1.467694	-0.117097
C	-1.085217	4.474837	-0.440653
C	3.059737	4.047240	-0.372110
H	3.602238	3.172027	1.520570

H	2.340675	4.402791	1.642787
H	1.313262	2.482106	3.152568
H	0.769693	1.003169	2.373068
C	2.860376	1.006919	2.852624
O	2.400386	1.892500	-1.295130
O	1.120489	0.433093	-0.234967
H	-1.883943	4.558283	0.310949
H	-0.917162	5.448069	-0.915409
H	-1.358150	3.716647	-1.188812
C	3.435587	2.891209	-1.285981
H	3.904135	4.752857	-0.325527
H	2.207315	4.584203	-0.808822
H	3.643763	1.714931	3.169753
H	2.573076	0.440889	3.757401
C	3.413946	0.056580	1.843636
H	3.436989	3.247435	-2.326946
H	2.678037	-0.551524	1.310855
C	4.723633	-0.130615	1.604403
H	5.456896	0.423444	2.203811
C	5.271437	-1.011616	0.570986
C	6.559111	-1.552259	0.708465
C	4.555241	-1.298785	-0.604753
C	7.102734	-2.375883	-0.272759
H	7.136580	-1.324778	1.608096
C	5.104167	-2.114961	-1.589202
H	3.579417	-0.833372	-0.762265
C	6.376559	-2.664547	-1.427255
H	8.103919	-2.791104	-0.138304
H	4.542465	-2.305341	-2.506850
H	6.805627	-3.299861	-2.204504
C	4.781518	2.262872	-0.982339
H	4.822833	1.870181	0.042834
H	4.977012	1.425054	-1.665848
H	5.581143	3.008896	-1.108959

HC-***I-Me***

SCF Energy = -2376.7754126

Free Energy (298K) = -2376.140085

Free Energy (298K, cc-pVTZ, PCM(THF)) = -2376.60206

Sm	-0.937417	-0.507464	0.002695
I	1.834279	-1.826743	0.178992
I	-3.920304	0.236629	-0.231850
O	-1.743776	-2.780383	-0.737425
C	-1.054169	-3.623555	-1.686133
C	-1.593253	-5.015311	-1.430055
C	-3.047163	-4.716277	-1.080257
C	-2.914096	-3.464449	-0.232437
O	-1.546367	-1.805483	2.052442
C	-0.770364	-2.829512	2.693261
C	-0.253183	-2.168423	3.965233
C	-1.305660	-1.078494	4.265313
C	-2.302673	-1.211886	3.118846
O	-0.679638	-0.730333	-2.479761
C	0.474356	-0.185216	-3.145593
C	-0.021433	1.046587	-3.911864
C	-1.555213	0.947940	-3.829981
C	-1.785193	-0.476796	-3.360702
H	-1.295581	-3.275942	-2.703795
H	0.023700	-3.506171	-1.511230
H	-2.734620	-3.704078	0.827094

H	-3.764172	-2.771735	-0.297927
H	-1.074525	-5.473880	-0.572736
H	-1.473443	-5.676367	-2.299471
H	-3.550695	-5.530171	-0.540548
H	-3.624120	-4.503892	-1.994531
H	-1.443267	-3.678039	2.915906
H	0.013109	-3.144482	1.993615
H	-2.715185	-0.269500	2.745945
H	-3.132866	-1.896979	3.365798
H	0.731417	-1.719352	3.773528
H	-0.142468	-2.896161	4.780948
H	-0.844099	-0.082327	4.242266
H	-1.794640	-1.213163	5.240236
H	1.224356	0.026875	-2.375050
H	0.868156	-0.964406	-3.820322
H	-1.739104	-1.202764	-4.194382
H	-2.710731	-0.624583	-2.789055
H	0.339111	1.025226	-4.950499
H	0.332720	1.972789	-3.442247
H	-2.055235	1.154479	-4.786380
H	-1.940423	1.643824	-3.071477
C	0.386479	5.289553	0.000706
C	0.732345	4.435238	1.209405
C	0.531169	2.932306	0.930972
C	-0.984023	4.923205	-0.527415
H	1.777874	4.604371	1.505257
H	0.107388	4.735525	2.059861
H	-1.721262	5.014574	0.290381
H	1.123937	5.148128	-0.808758
H	0.395717	6.357887	0.267269
C	-0.155185	2.670476	-0.393078
O	-0.251177	1.482005	-0.841698
O	-1.009959	3.548502	-0.951034
C	-1.429794	5.740664	-1.716656
H	-2.395734	5.374119	-2.091348
H	-1.542351	6.797744	-1.434139
H	-0.692199	5.670253	-2.530737
C	-0.528194	2.283662	1.796508
O	-0.782676	1.038273	1.738274
O	-1.412038	3.132731	2.357696
C	-2.632978	2.575247	2.831676
H	-3.234912	3.429158	3.164040
H	-3.155484	2.027086	2.033401
H	-2.447496	1.902711	3.683550
C	1.854937	2.159757	1.026036
H	2.309930	2.417093	1.997620
H	1.674831	1.076271	1.044667
C	2.821840	2.475502	-0.115070
H	2.398879	2.094252	-1.057844
H	2.922688	3.570481	-0.227395
C	4.170257	1.875180	0.117068
H	4.770620	2.320167	0.919996
C	4.636081	0.804836	-0.540014
H	3.986089	0.339118	-1.288585
C	5.919419	0.132225	-0.320382
C	6.075129	-1.192838	-0.754051
C	7.007747	0.754083	0.311593
C	7.267449	-1.880235	-0.547127
H	5.233689	-1.690460	-1.242118
C	8.199579	0.068637	0.517237

H 6.923224 1.795398 0.629017

C 8.335766 -1.253264 0.091069

H 7.362180 -2.914119 -0.885908

H 9.035558 0.572882 1.006986

H 9.273830 -1.788625 0.250834

C-***I-H***

SCF Energy = -2337.50959

Free Energy (298K) = -2336.897298

Free Energy (298K, cc-pVTZ, PCM(THF)) = -2337.348044

Sm -0.999984 0.086477 0.003711

I -1.731386 -0.974817 2.814885

I -0.761265 1.375540 -2.788101

O -2.805550 1.793498 0.524680

C -3.993030 1.587599 1.323224

C -4.780914 2.891129 1.247560

C -4.241618 3.545455 -0.021481

C -2.783175 3.145884 0.032490

O 0.019121 2.212905 0.814862

C 0.105260 2.465115 2.232651

C 1.568160 2.766806 2.474627

C 1.917470 3.570004 1.225449

C 1.143075 2.836353 0.142761

O -3.184858 -0.825748 -0.799353

C -3.642582 -2.161092 -0.484630

C -5.059952 -2.222725 -1.015742

C -4.956826 -1.348748 -2.261364

C -4.058684 -0.224265 -1.781468

H -4.533588 0.733496 0.887619

H -3.686363 1.307948 2.340152

H -2.213689 3.777156 0.736631

H -2.270313 3.133489 -0.938568

H -4.559878 3.523895 2.121281

H -5.865697 2.717802 1.226305

H -4.380861 4.635060 -0.045967

H -4.715745 3.117959 -0.919678

H -0.536423 3.331746 2.475689

H -0.274304 1.578962 2.760337

H 1.735452 2.029768 -0.310970

H 0.750235 3.480987 -0.654768

H 2.136289 1.823507 2.505813

H 1.737136 3.312856 3.412949

H 2.993126 3.596984 1.005164

H 1.560962 4.607425 1.328277

H -2.978281 -2.875382 -0.993875

H -3.547824 -2.293596 0.602088

H -4.636217 0.571580 -1.281827

H -3.431520 0.228314 -2.561016

H -5.764879 -1.783242 -0.291315

H -5.381078 -3.252599 -1.224161

H -5.925181 -0.979495 -2.626052

H -4.470309 -1.903472 -3.079215

O -0.612904 -2.175066 -0.532019

C 0.415553 -2.896894 -0.503467

C 1.766855 -2.406838 -0.949478

O 0.402053 -4.073912 0.123404

C 2.852260 -3.491563 -0.914741

C 1.641098 -1.775834 -2.345673

C 2.000920 -1.320738 0.103437

C -0.794318 -4.413735 0.828141

C 3.390092 -3.768460 0.481009

H 3.685950 -3.143431 -1.542755

H 2.456924 -4.406625 -1.379770

H 1.350514 -2.566026 -3.057714

H 0.813774 -1.053150 -2.334961

C 2.904755 -1.068820 -2.847781

O 2.686326 -1.579965 1.240758

O 1.175095 -0.339793 0.208777

H -1.613209 -4.605570 0.118894

H -0.556190 -5.330621 1.378943

H -1.084546 -3.607681 1.517502

C 3.799752 -2.465385 1.127240

H 4.262067 -4.439508 0.427458

H 2.626419 -4.260407 1.101369

H 3.653554 -1.791705 -3.210474

H 2.600309 -0.469449 -3.723998

C 3.528324 -0.169903 -1.831999

H 4.592914 -1.964941 0.545552

H 4.155612 -2.605988 2.156037

H 2.861716 0.564304 -1.371293

C 4.840039 -0.156105 -1.523981

H 5.514071 -0.832464 -2.064105

C 5.457179 0.683662 -0.497455

C 6.823873 0.997960 -0.567553

C 4.723945 1.164046 0.602732

C 7.430564 1.790647 0.401733

H 7.412994 0.619017 -1.406622

C 5.333919 1.951261 1.574036

H 3.677636 0.873819 0.715609

C 6.687413 2.276297 1.476946

H 8.493286 2.028866 0.319469

H 4.750578 2.297580 2.430623

H 7.163356 2.890824 2.243506

HC-***I-H***

SCF Energy = -2337.4988665

Free Energy (298K) = -2336.889278

Free Energy (298K, cc-pVTZ, PCM(THF)) = -2337.34084

Sm -0.982836 -0.362544 -0.002449

I 1.727499 -1.793254 0.223941

I -3.928692 0.515186 -0.256843

O -1.922778 -2.657611 -0.478071

C -1.308195 -3.633915 -1.348591

C -1.959808 -4.950986 -0.979928

C -3.376883 -4.503845 -0.637023

C -3.119981 -3.211490 0.114222

O -1.607270 -1.422911 2.177377

C -0.851860 -2.410808 2.894825

C -0.263885 -1.646333 4.074020

C -1.271915 -0.498887 4.304475

C -2.306025 -0.697251 3.200183

O -0.822220 -0.838264 -2.454433

C 0.318235 -0.395618 -3.213320

C -0.176235 0.775609 -4.069335

C -1.709470 0.710815 -3.943066

C -1.949338 -0.654470 -3.325365

H -1.522316 -3.353045 -2.392279

H -0.223422 -3.592293 -1.181423

H	-2.919646	-3.392920	1.181959
H	-3.915383	-2.459004	0.025975
H	-1.468036	-5.387822	-0.095804
H	-1.911211	-5.683294	-1.797597
H	-3.938003	-5.230216	-0.033068
H	-3.950012	-4.307450	-1.557330
H	-1.550627	-3.201650	3.224583
H	-0.107496	-2.830149	2.207221
H	-2.689643	0.222333	2.748077
H	-3.156130	-1.318578	3.533023
H	0.723684	-1.249998	3.798848
H	-0.138513	-2.293246	4.953207
H	-0.779529	0.475224	4.184263
H	-1.737128	-0.532984	5.299557
H	1.107213	-0.138358	-2.497700
H	0.658041	-1.246342	-3.828487
H	-1.938879	-1.461930	-4.081624
H	-2.859838	-0.725090	-2.716421
H	0.159236	0.661713	-5.110282
H	0.202391	1.730911	-3.685323
H	-2.230260	0.827897	-4.903471
H	-2.067956	1.485927	-3.250935
C	0.638560	5.339119	-0.546740
C	0.949622	4.580265	0.733617
C	0.665666	3.069901	0.593329
C	-0.744819	4.974586	-1.029543
H	2.004762	4.718439	1.011233
H	0.346862	4.988454	1.554891
H	-1.497467	5.205765	-0.256457
H	1.369378	5.095380	-1.336572
H	0.690079	6.426156	-0.378163
C	-0.040698	2.719516	-0.701504
O	-0.207328	1.499872	-1.031073
O	-0.847068	3.586216	-1.347587
C	-0.418038	2.561517	1.519077
O	-0.733603	1.331623	1.582771
O	-1.247738	3.503631	2.007717
C	-2.486397	3.057146	2.549332
H	-3.038704	3.968606	2.806380
H	-3.049522	2.463116	1.814083
H	-2.318733	2.460019	3.459053
C	1.948622	2.241906	0.759780
H	2.420120	2.566345	1.703097
H	1.713814	1.175590	0.880411
C	2.926152	2.398608	-0.404839
H	2.490681	1.932855	-1.302762
H	3.065985	3.470839	-0.634102
C	4.252513	1.781033	-0.101439
H	4.866272	2.291254	0.651075
C	4.682799	0.629662	-0.633993
H	4.018282	0.106155	-1.329652
C	5.941949	-0.056974	-0.332555
C	6.053643	-1.426544	-0.615313
C	7.048878	0.594266	0.234246
C	7.220812	-2.126551	-0.324202
H	5.197686	-1.946429	-1.052232
C	8.215646	-0.103891	0.523749
H	6.999167	1.666755	0.433125
C	8.307692	-1.468940	0.248455
H	7.281249	-3.194295	-0.545428

H 9.066749 0.423253 0.960481

H 9.226142 -2.014603 0.473848

H -1.019637 5.506911 -1.950560

8. X–Ray Crystallography Data

Crystal data and structure refinement for **1s** (CCDC 2113457)

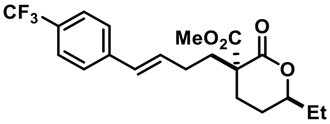

1s

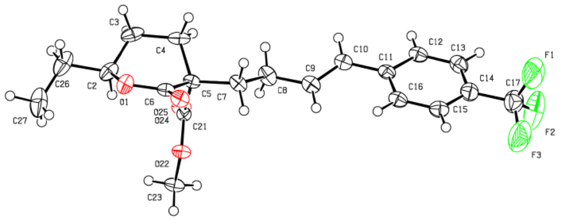

**Empirical formula**	$C_{20}H_{23}F_3O_4$
**Formula weight**	384.38
**Temperature (K)**	168
**Space group**	C 2/c
**a (Å)**	25.4807 (15)
**b (Å)**	5.9380 (3)
**c (Å)**	25.0707 (14)
**α (°)**	90
**β (°)**	91.600 (5)
**γ (°)**	90
**Volume ($Å^3$)**	3791.8 (4)
**Z**	8
**d_{cale} (g, cm^{-1})**	1.347
**λ (Å)**	0.71073
**μ (mm^{-1})**	0.112
**R_1, wR_2 (all data)**	0.0709; 0.1715

Crystal data and structure refinement for **3p** (CCDC 2113458)

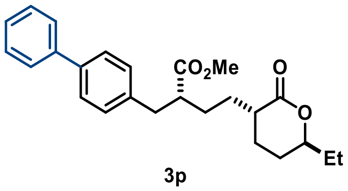

3p

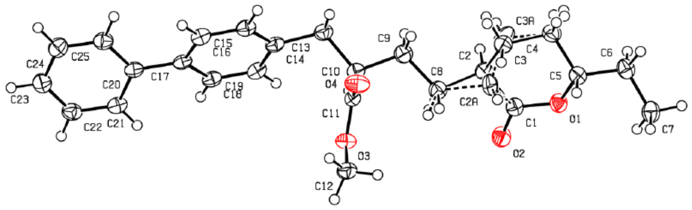

**Empirical formula**	$\mathbf{C_{25}H_{30}O_4}$
**Formula weight**	394.49
**Temperature (K)**	100
**Space group**	P 21/n
**a (Å)**	8.07151 (17)
**b (Å)**	10.8691 (3)
**c (Å)**	24.5220 (7)
**α (°)**	90
**β (°)**	97.472 (12)
**γ (°)**	90
**Volume (Å^3)**	2133.05 (10)
**Z**	4
**d_{cale} (g, cm^{-1})**	1.228
**λ (Å)**	1.54184
**μ (mm^{-1})**	0.653
**R_1, wR_2 (all data)**	0.0418; 0.1090

9. References

(1) Campaña, A. G.; Fuentes, N.; Gómez-Bengoa, E.; Mateo, C.; Oltra, J. E.; Echavarren, A. M.; Cuerva, J. M. Sodium Tetramethoxyborate: An Efficient Catalyst for Michael Additions of Stabilized Carbon Nucleophiles. *J. Org. Chem.* **2007**, *72* (21), 8127–8130. https://doi.org/10.1021/jo701354c.

(2) Nagasawa, S.; Sasano, Y.; Iwabuchi, Y. Synthesis of 1,3-Cycloalkadienes from Cycloalkenes: Unprecedented Reactivity of Oxoammonium Salts. *Angew. Chemie Int. Ed.* **2016**, *55* (42), 13189–13194. https://doi.org/10.1002/anie.201607752.

(3) Lin, J.; Zhu, T.; Jia, M.; Ma, S. A Pd-Catalyzed Ring Opening Coupling Reaction of 2,3-Allenylic Carbonates with Cyclopropanols. *Chem. Commun.* **2019**, *55* (31), 4523–4526. https://doi.org/10.1039/C9CC00979E.

(4) Groaning, M. D.; Brengel, G. P.; Meyers, A. I. Allyldimethyltritylsilane. Synthesis of Cyclopentanols, Oxetanes, and Tetrahydrofurans by Reaction with Electron Deficient Olefins. *J. Org. Chem.* **1998**, *63* (16), 5517–5522. https://doi.org/10.1021/jo9805652.

(5) Feng, T.; Tian, M.; Zhang, X.; Fan, X. Tunable Synthesis of Functionalized Cyclohexa-1,3-Dienes and 2-Aminobenzophenones/Benzoate from the Cascade Reactions of Allenic Ketones/Allenoate with Amines and Enones. *J. Org. Chem.* **2018**, *83* (9), 5313–5322. https://doi.org/10.1021/acs.joc.8b00473.

(6) Tang, M.; Li, Y.; Han, S.; Liu, L.; Ackermann, L.; Li, J. Rhodium(III)-Catalyzed C–H Alkylation/Nucleophilic Addition Domino Reaction. *European J. Org. Chem.* **2019**, *2019* (4), 660–664. https://doi.org/10.1002/ejoc.201801535.

(7) Felker, I.; Pupo, G.; Kraft, P.; List, B. Design and Enantioselective Synthesis of Cashmeran Odorants by Using "Enol Catalysis." *Angew. Chemie Int. Ed.* **2015**, *54* (6), 1960–1964. https://doi.org/10.1002/anie.201409591.

(8) Lim, H. N.; Dong, G. Catalytic Intramolecular Ketone Alkylation with Olefins by Dual Activation. *Angew. Chemie Int. Ed.* **2015**, *54* (50), 15294–15298. https://doi.org/10.1002/anie.201507741.

(9) Effenberger, F.; Wezstein, M. Synthesis of Aryl-Terminated Polyenaldehydes and Polyenetriethoxysilanes for Preparation of Self-Assembled Monolayers on Silicon Surfaces. *Synthesis (Stuttg).* **2001**, *2001* (09), 1368–1376. https://doi.org/10.1055/s-2001-15224.

(10) Hüggenberg, W.; Seper, A.; Oppel, I. M.; Dyker, G. Multifold Photocyclization Reactions of Styrylcalix[4]Arenes. *European J. Org. Chem.* **2010**, *2010* (35), 6786–6797. https://doi.org/10.1002/ejoc.201001108.

(11) Heinrich, C. F.; Durand, D.; Starck, J.; Michelet, V. Ruthenium Metathesis: A Key Step To Access a New Cyclic Tetrasubstituted Olefin Platform. *Org. Lett.* **2020**, *22* (18), 7064–7067. https://doi.org/10.1021/acs.orglett.0c01344.

(12) Gasperini, D.; Maggi, L.; Dupuy, S.; Veenboer, R. M. P.; Cordes, D. B.; Slawin, A. M. Z.; Nolan, S. P. Gold(I)-Catalysed Cyclisation of Alkynoic Acids: Towards an Efficient and Eco-Friendly Synthesis of γ-, δ- and ϵ-Lactones. *Adv. Synth. Catal.* **2016**, *358* (23), 3857–3862. https://doi.org/10.1002/adsc.201600575.

(13) Yang, X.-H.; Yue, H.-T.; Yu, N.; Li, Y.-P.; Xie, J.-H.; Zhou, Q.-L. Iridium-Catalyzed Asymmetric Hydrogenation of Racemic α-Substituted Lactones to Chiral Diols. *Chem. Sci.* **2017**, *8* (3), 1811–1814. https://doi.org/10.1039/C6SC04609F.

(14) Fisher, M. J.; Overman, L. E. Intramolecular N-(Acyloxy)Iminium Ion-Alkyne Cycloadditions. A New Route to Bicyclic .Alpha.-Amino Ketones. *J. Org. Chem.* **1990**, *55* (5), 1447–1459. https://doi.org/10.1021/jo00292a014.

(15) Webb, G. *Annual Reports on NMR Spectroscopy, Volume 11*; 1981.

(16) Frisch, M. J.; Trucks, G. W.; Schlegel, H. B.; Scuseria, G. E.; Robb, M. A.; Cheeseman, J. R.; Scalmani, G.; Barone, V.; Petersson, G. A.; Nakatsuji, H.; Li, X.; Caricato, M.; Marenich, A. V.; Bloino, J.; Janesko, B. G.; Gomperts, R.; Mennucci, B.; Hratchian, H. P.; Ortiz, J. V.; Izmaylov, A. F.; Sonnenberg, J. L.; Williams-Young, D.; Ding, F.; Lipparini, F.; Egidi, F.; Goings, J.; Peng, B.; Petrone, A.; Henderson, T.; Ranasinghe, D.; Zakrzewski, V. G.; Gao, J.; Rega, N.; Zheng, G.; Liang, W.; Hada, M.; Ehara, M.; Toyota, K.; Fukuda, R.; Hasegawa, J.; Ishida, M.; Nakajima, T.; Honda, Y.; Kitao, O.; Nakai, H.; Vreven, T.; Throssell, K.; Jr. Montgomery, J. A.; Peralta, J. E.; Ogliaro, F.; Bearpark, M. J.; Heyd, J. J.; Brothers, E. N.; Kudin, K. N.; Staroverov, V. N.; Keith, T. A.; Kobayashi, R.; Normand, J.; Raghavachari, K.; Rendell, A. P.; Burant, J. C.; Iyengar, S. S.; Tomasi, J.; Cossi, M.; Millam, J. M.; Klene, M.; Adamo, C.; Cammi, R.; Ochterski, J. W.; Martin, R. L.; Morokuma, K.; Farkas, O.; Foresman, J. B.; Fox, D. J. Gaussian 16, Revision C.01. Gaussian, Inc., Wallingford CT 2016.

(17) Adamo, C.; Barone, V. Toward Reliable Density Functional Methods without Adjustable Parameters: The PBE0 Model. *J. Chem. Phys.* **1999**, *110* (13), 6158–6170. https://doi.org/10.1063/1.478522.

(18) Dunning, T. H. Gaussian Basis Sets for Use in Correlated Molecular Calculations. I. The Atoms Boron through Neon and Hydrogen. *J. Chem. Phys.* **1989**, *90* (2), 1007–1023. https://doi.org/10.1063/1.456153.

(19) Grimme, S.; Antony, J.; Ehrlich, S.; Krieg, H. A Consistent and Accurate Ab Initio Parametrization of Density Functional Dispersion Correction (DFT-D) for the 94 Elements H-Pu. *J. Chem. Phys.* **2010**, *132* (15), 154104. https://doi.org/10.1063/1.3382344.

(20) Johnson, E. R.; Becke, A. D. A Post-Hartree-Fock Model of Intermolecular Interactions: Inclusion of Higher-Order Corrections. *J. Chem. Phys.* **2006**, *124* (17), 174104. https://doi.org/10.1063/1.2190220.

(21) Tomasi, J.; Mennucci, B.; Cammi, R. Quantum Mechanical Continuum Solvation Models. *Chem. Rev.* **2005**, *105* (8), 2999–3094. https://doi.org/10.1021/cr9904009.

(22) Cao, X.; Dolg, M. Segmented Contraction Scheme for Small-Core Lanthanide Pseudopotential Basis Sets. *J. Mol. Struct. THEOCHEM* **2002**, *581* (1–3), 139–147. https://doi.org/10.1016/S0166-1280(01)00751-5.

(23) Martin, J. M. L.; Sundermann, A. Correlation Consistent Valence Basis Sets for Use with the Stuttgart–Dresden–Bonn Relativistic Effective Core Potentials: The Atoms Ga–Kr and In–Xe. *J. Chem. Phys.* **2001**, *114* (8), 3408–3420. https://doi.org/10.1063/1.1337864.

(24) Dennington, R.; Keith, T.; Millam, J. GaussView Version 6.1.1. Semichem Inc. Shawnee Mission, KS 2019.

(25) Agasti, S.; Beattie, N. A.; McDouall, J. J. W.; Procter, D. J. SmI 2 -Catalyzed Intermolecular Coupling of Cyclopropyl Ketones and Alkynes: A Link between Ketone Conformation and Reactivity. *J. Am. Chem. Soc.* **2021**, *143* (9), 3655–3661. https://doi.org/10.1021/jacs.1c01356.

10. NMR Spectra

Dimethyl 2–(but–3–en–1–yl)malonate (S1)

^{1}H NMR (500 MHz, $CDCl_3$)

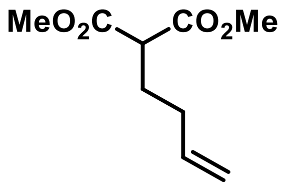

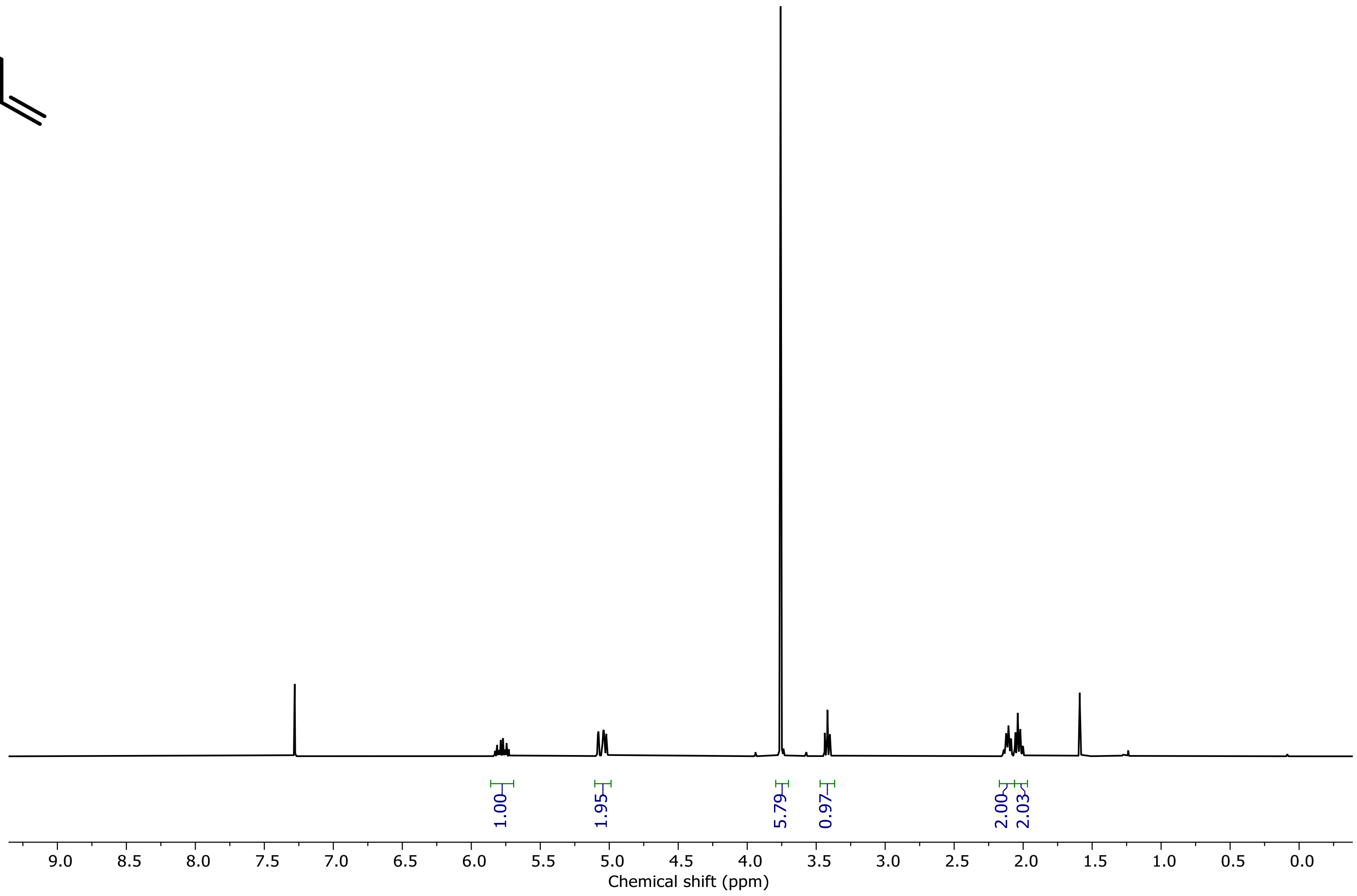

Dimethyl (*E*)–2–(4–phenylbut–3–en–1–yl)malonate (S2)

^1H NMR (400 MHz, $CDCl_3$)

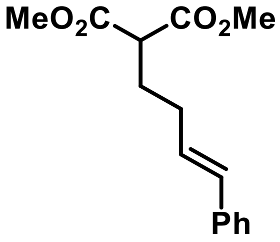

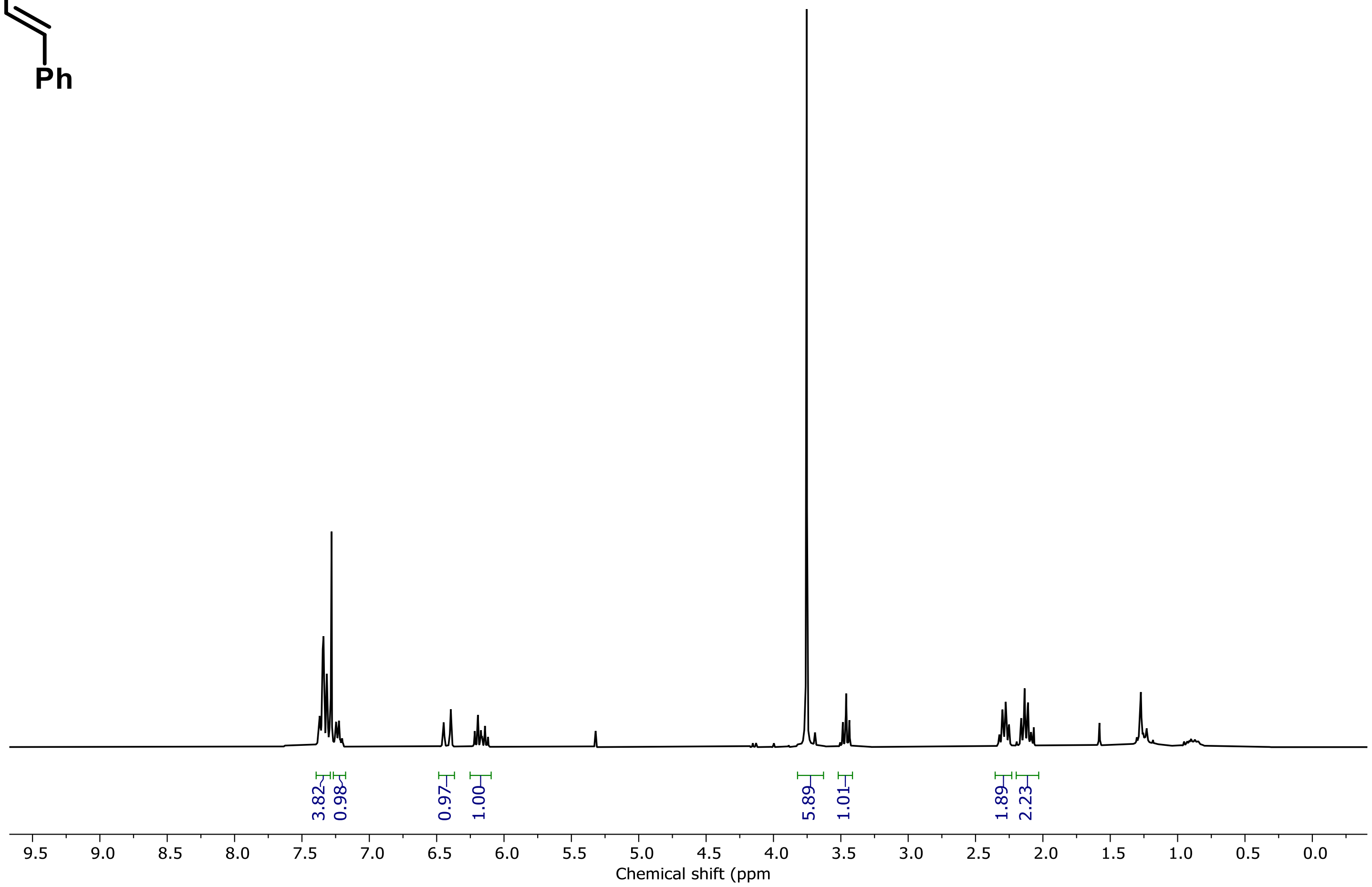

^{13}C NMR (101 MHz, $CDCl_3$)

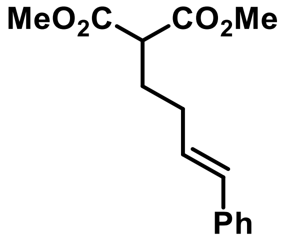

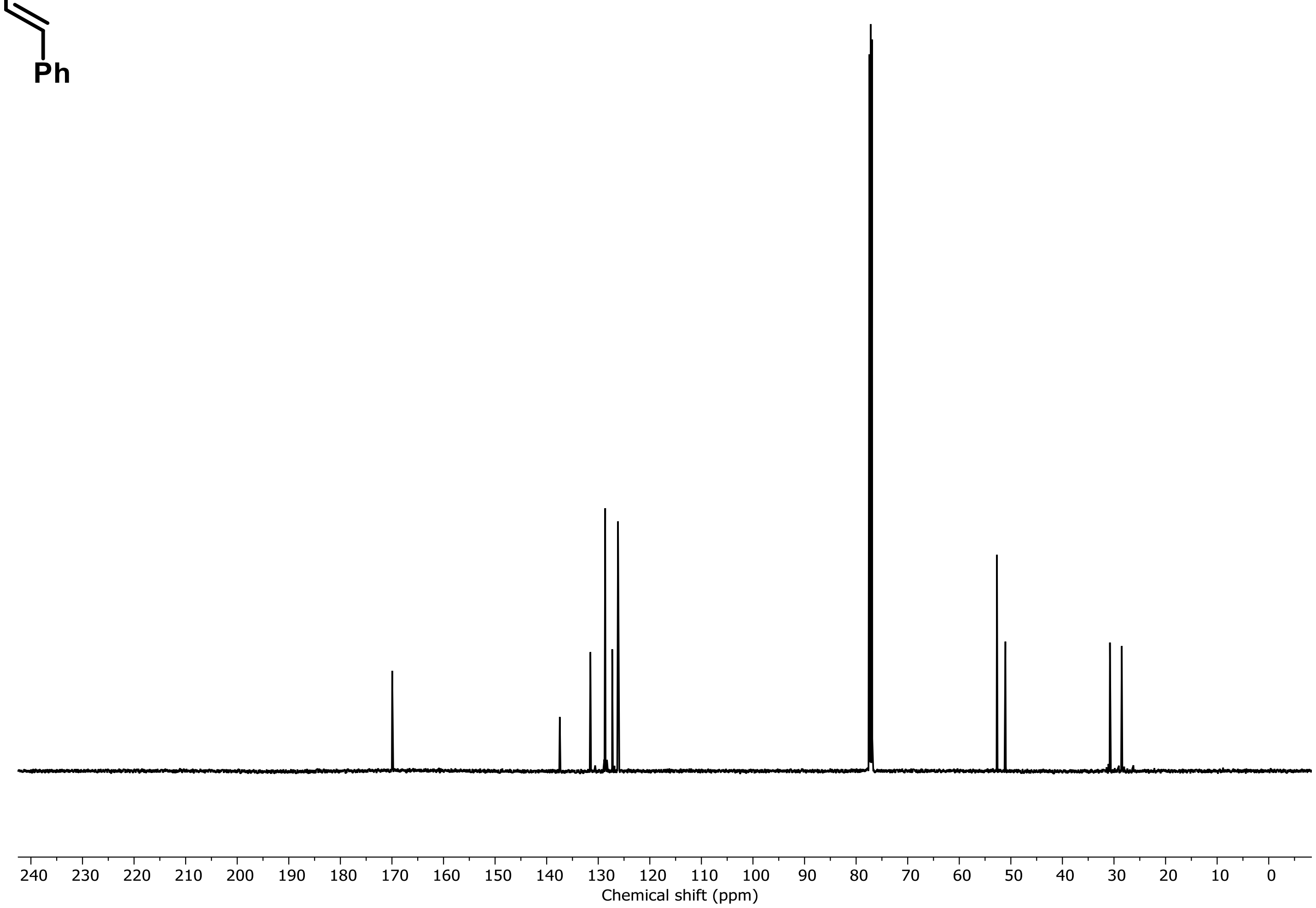

Dimethyl (*E*)–2–(3–oxobutyl)–2–(4–phenylbut–3–en–1–yl)malonate (S3)

^{1}H NMR (400 MHz, $CDCl_3$)

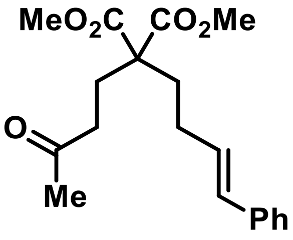

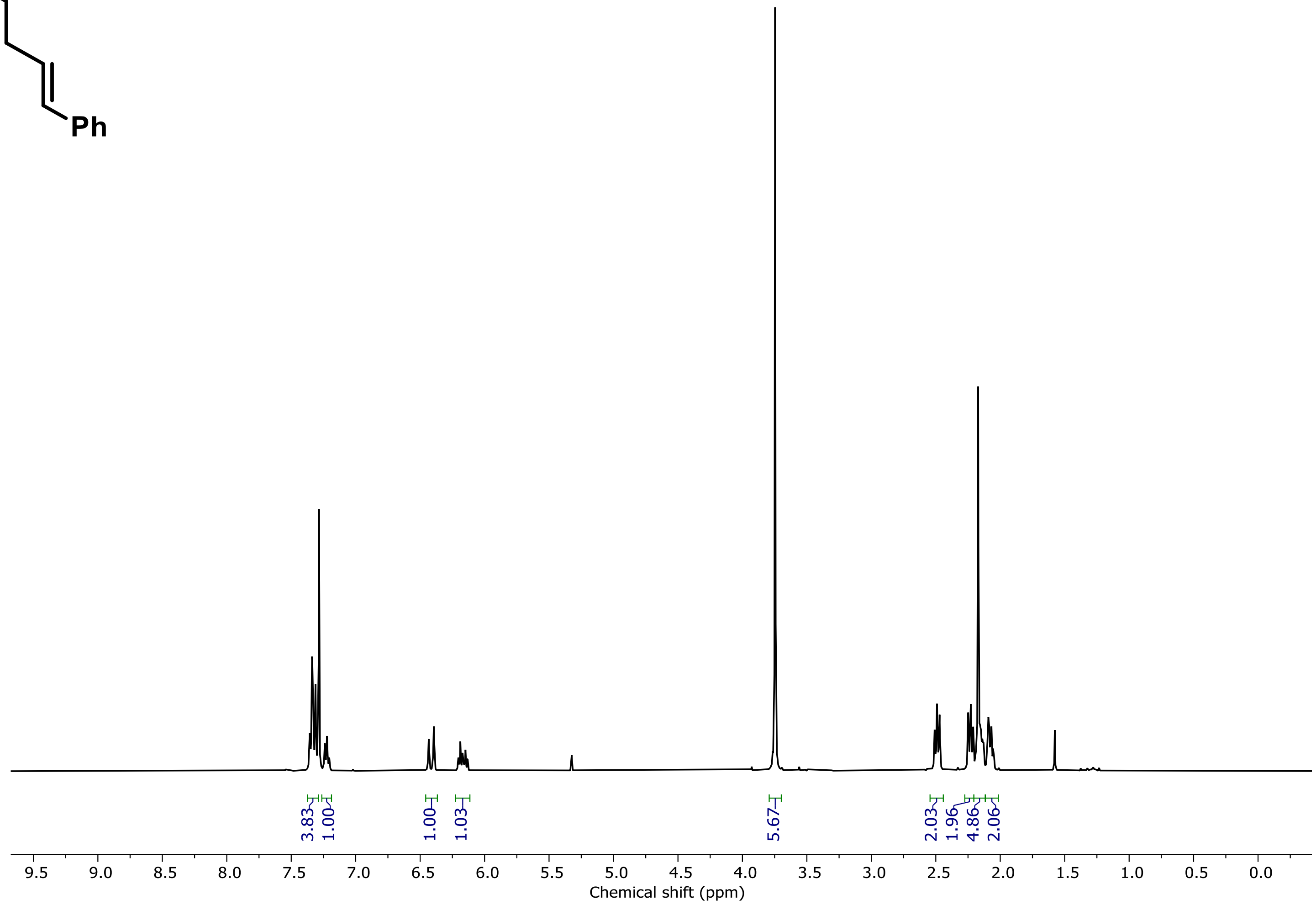

^{13}C NMR (101 MHz, $CDCl_3$)

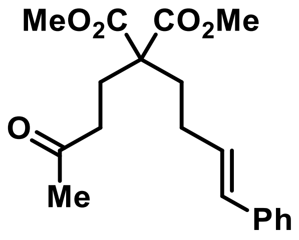

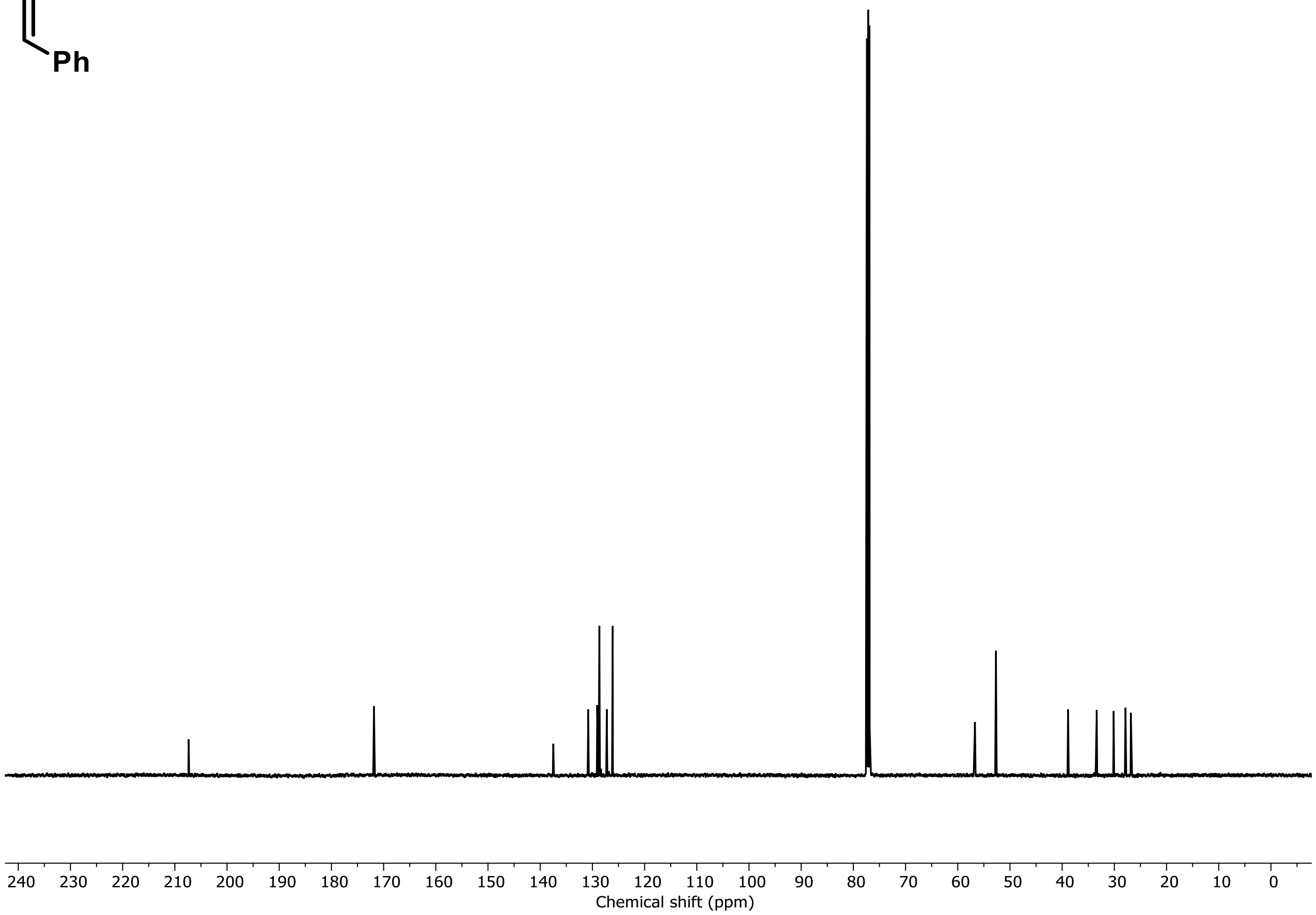

Dimethyl (*E*)–2–(3–oxopentyl)–2–(4–phenylbut–3–en–1–yl)malonate (S4)

^{1}H NMR (500 MHz, $CDCl_3$)

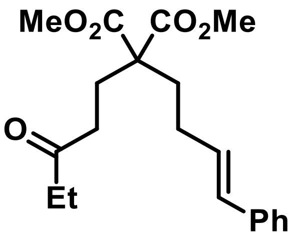

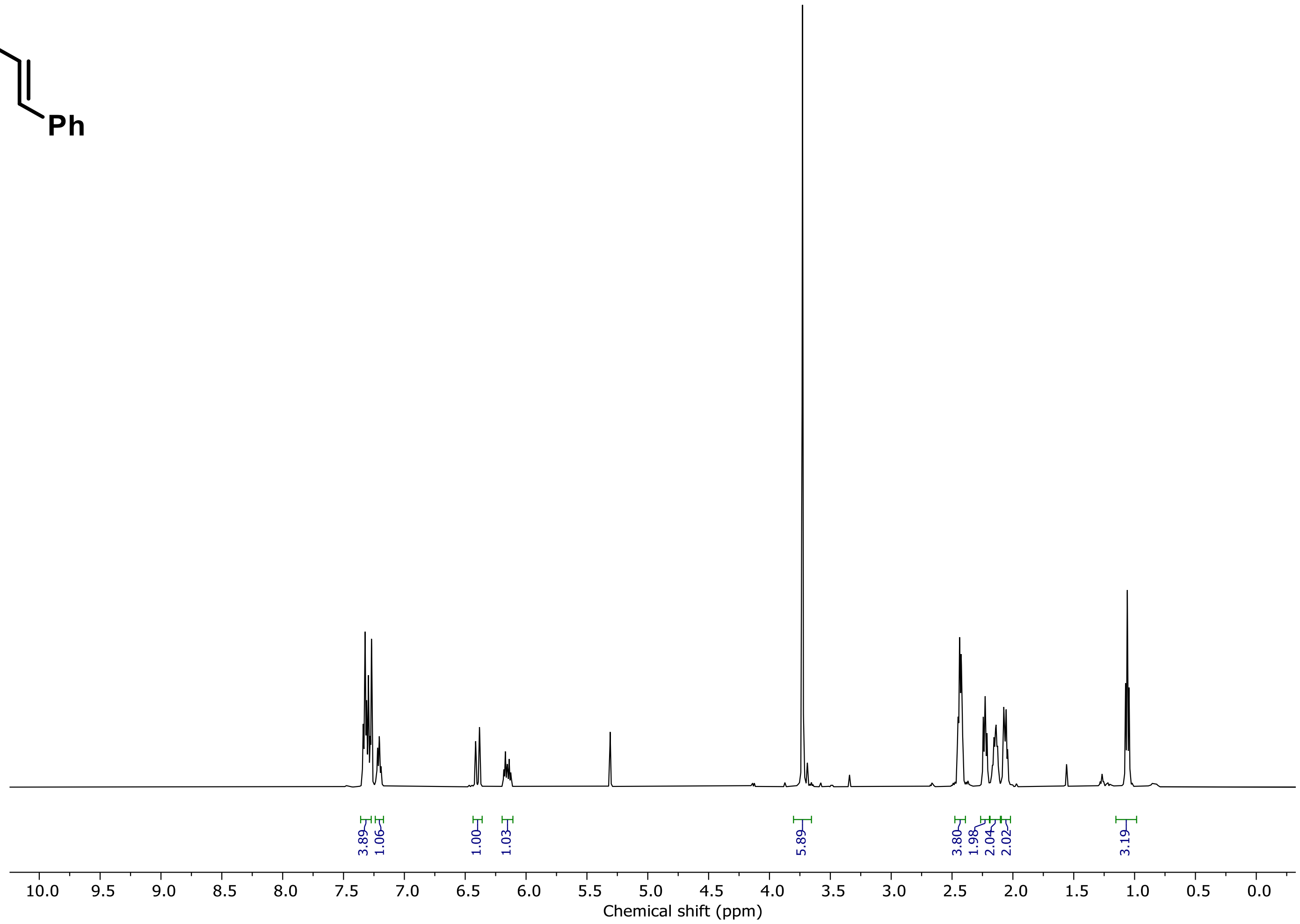

^{13}C NMR (126 MHz, $CDCl_3$)

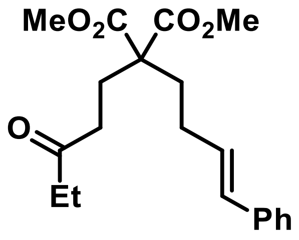

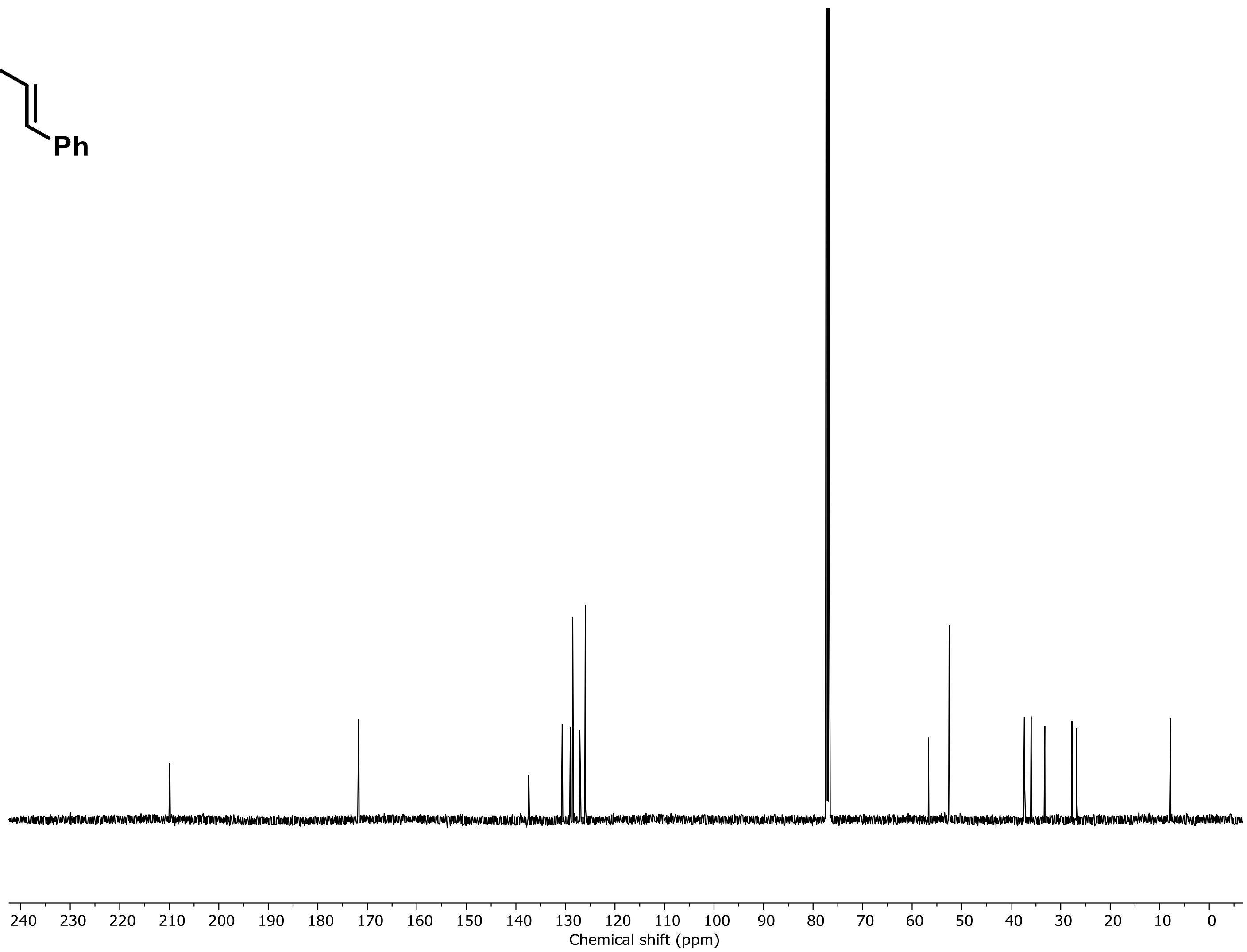

Dimethyl (*E*)–2–(3–oxoheptyl)–2–(4–phenylbut–3–en–1–yl)malonate (S6)

^{1}H NMR (400 MHz, $CDCl_3$)

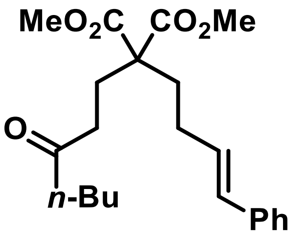

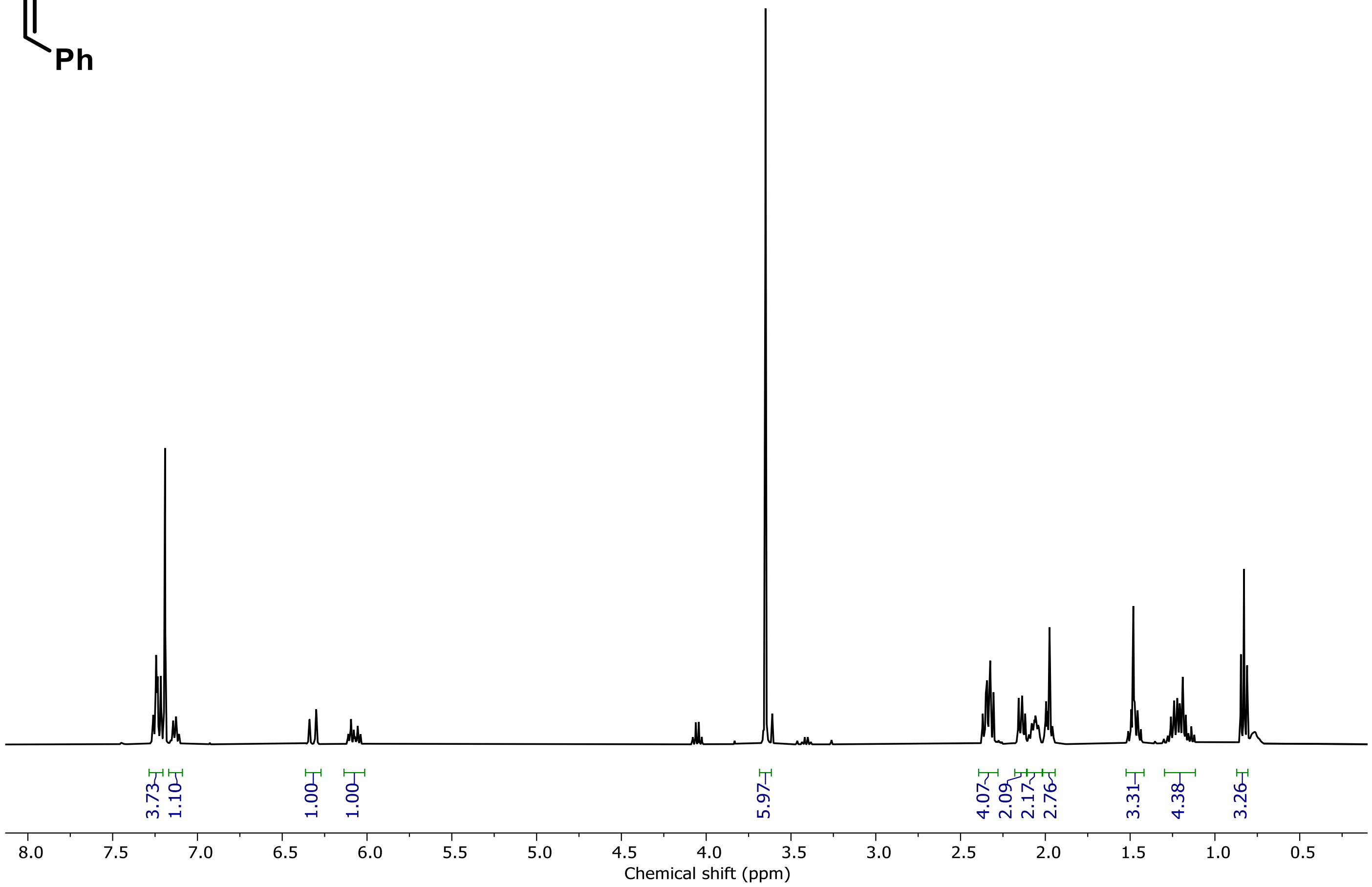

^{13}C NMR (101 MHz, $CDCl_3$)

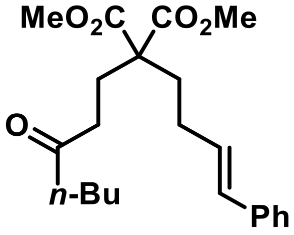

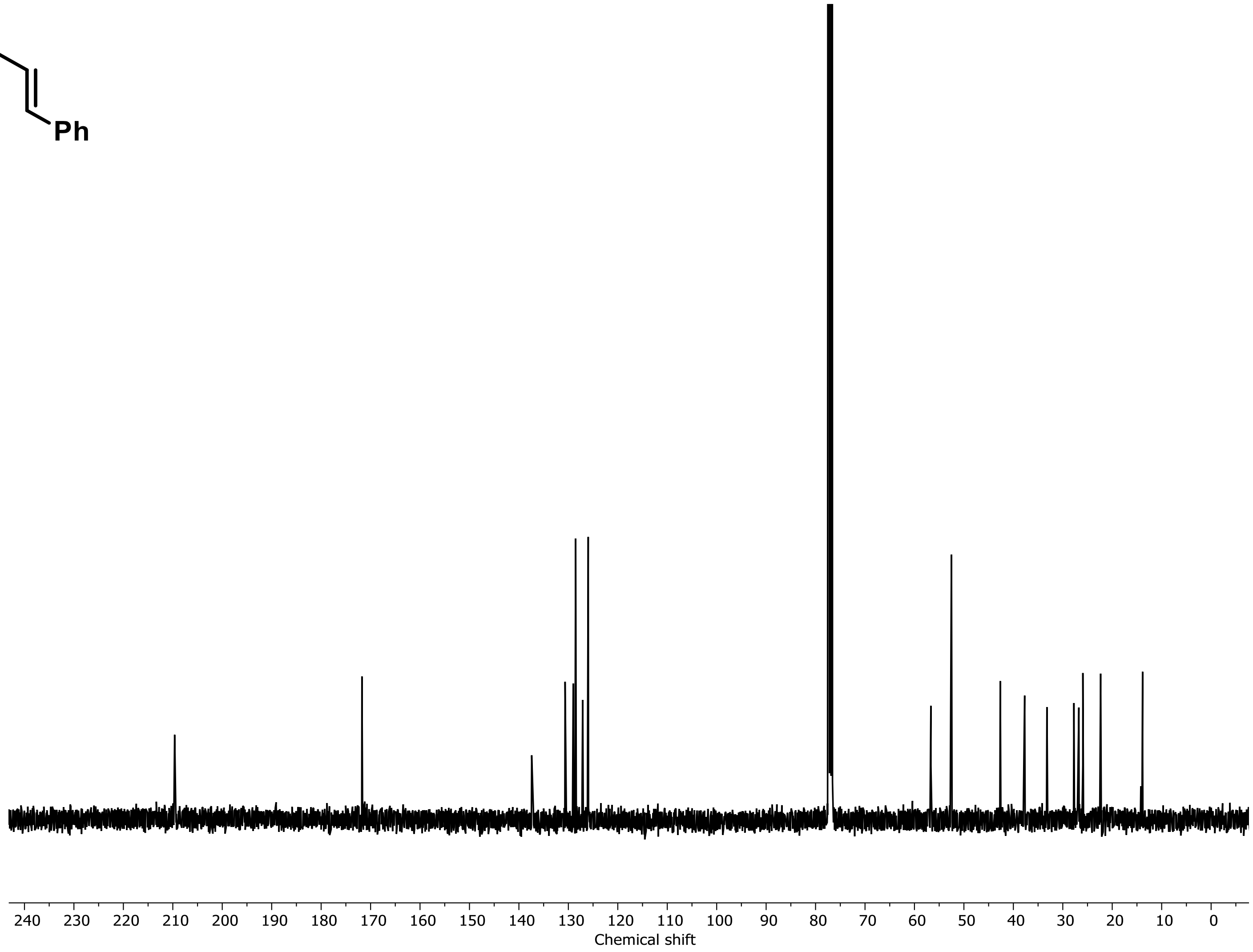

Dimethyl (*E*)–2–(3–oxo–4–phenylbutyl)–2–(4–phenylbut–3–en–1–yl)malonate (S8)

^{1}H NMR (400 MHz, $CDCl_3$)

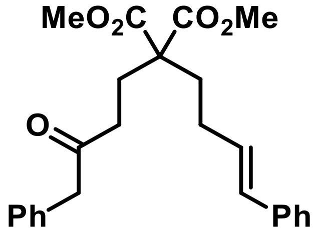

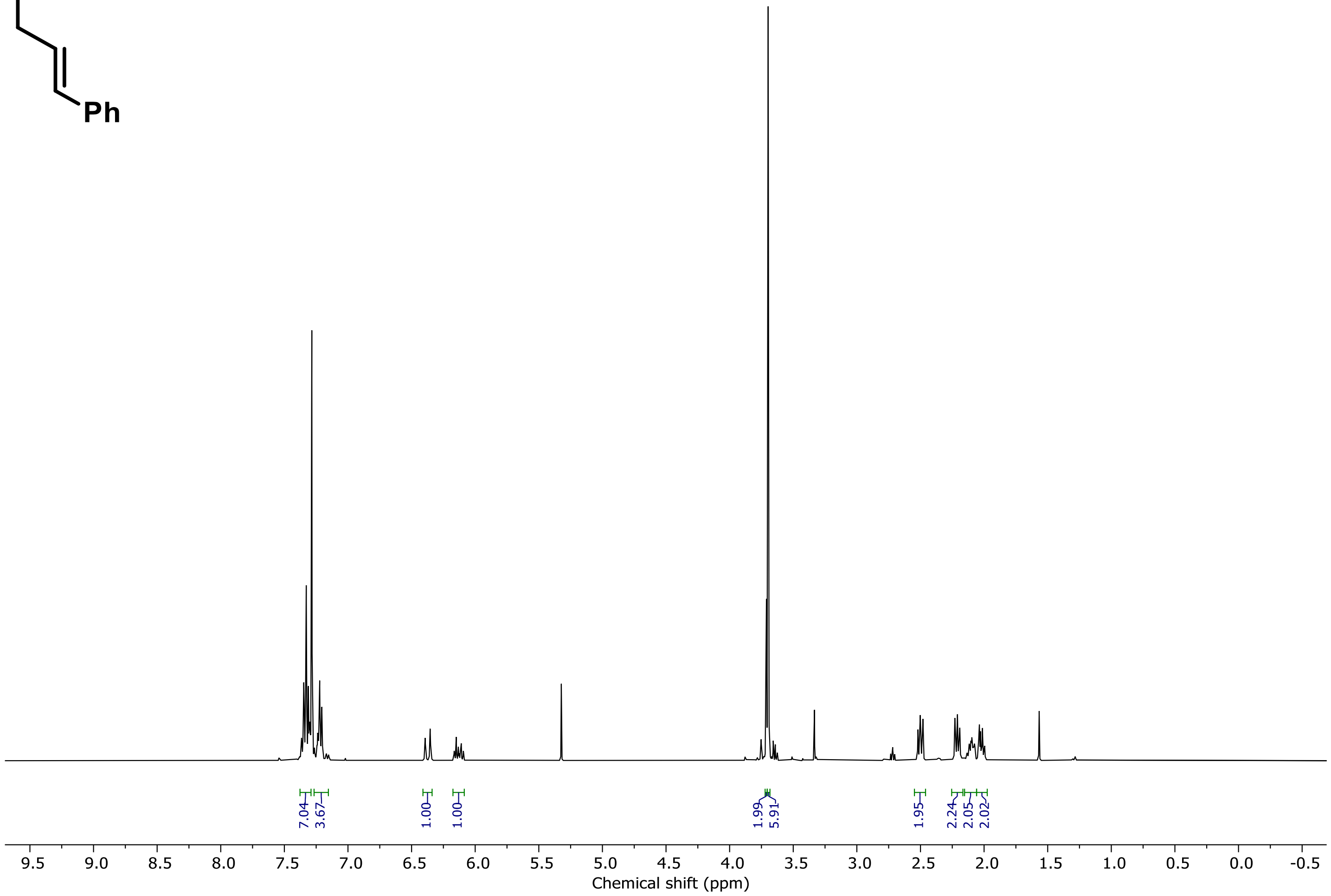

^{13}C NMR (101 MHz, $CDCl_3$)

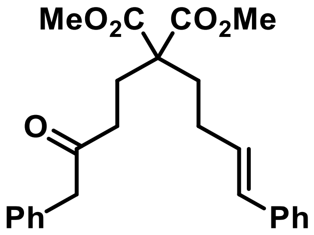

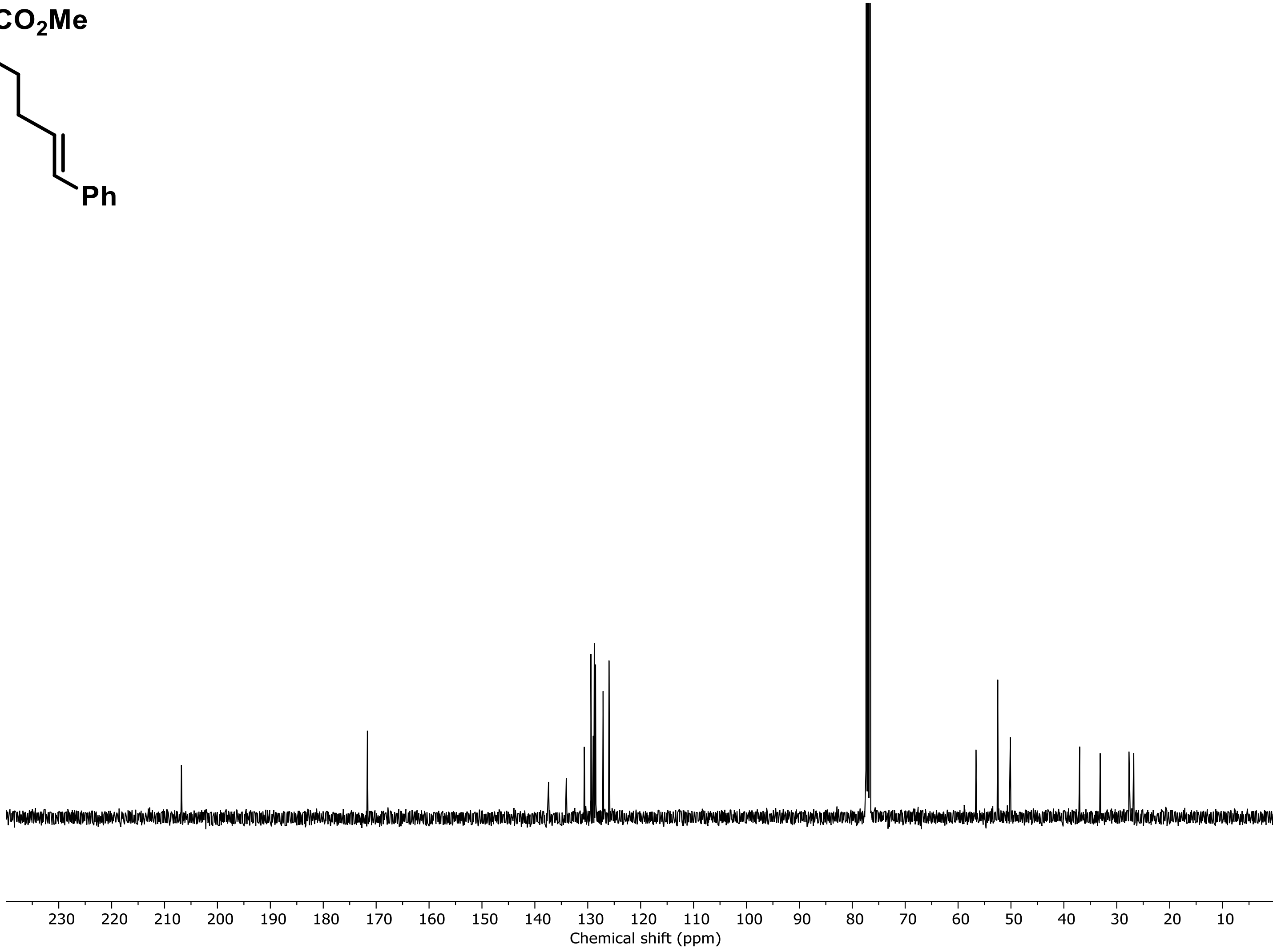

Dimethyl (*E*)–2–(5,5–dimethyl–3–oxohexyl)–2–(4–phenylbut–3–en–1–yl)malonate (S10)

^{1}H NMR (400 MHz, $CDCl_3$)

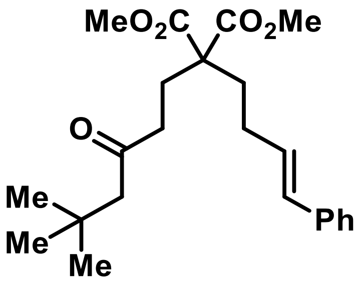

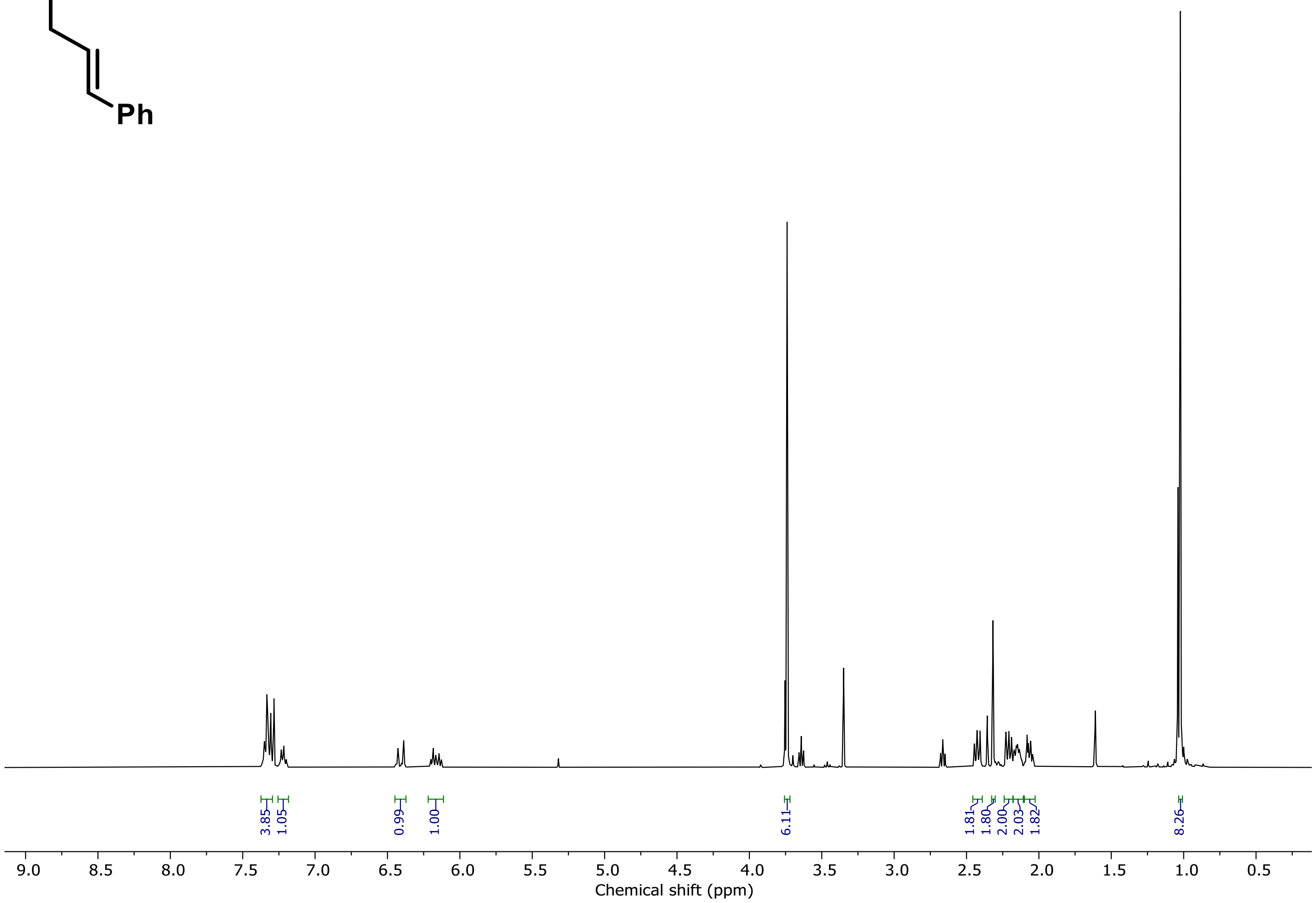

^{13}C NMR (101 MHz, $CDCl_3$)

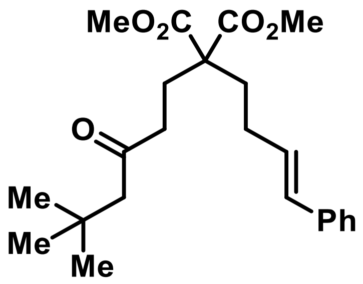

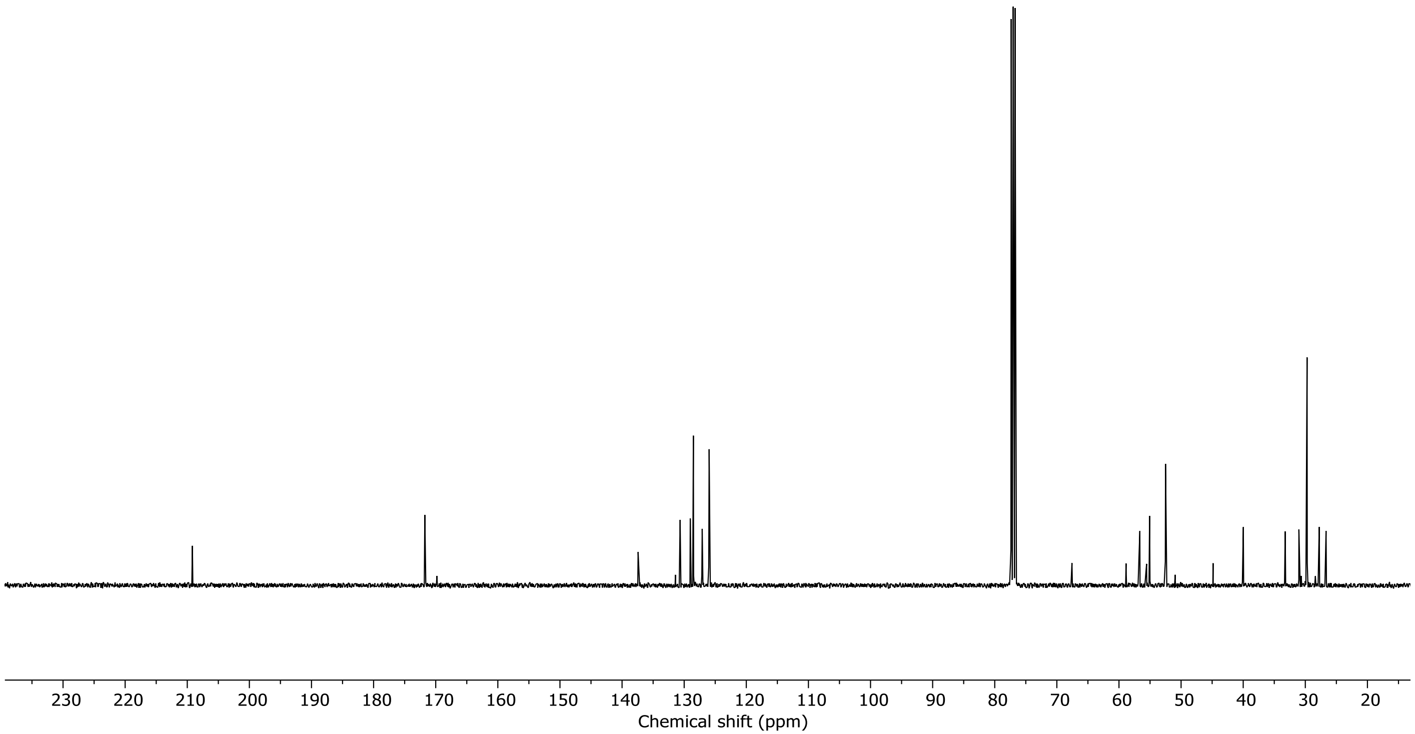

Dimethyl (*E*)–2–(4–methyl–3–oxopentyl)–2–(4–phenylbut–3–en–1–yl)malonate (S12)

^{1}H NMR (500 MHz, $CDCl_3$)

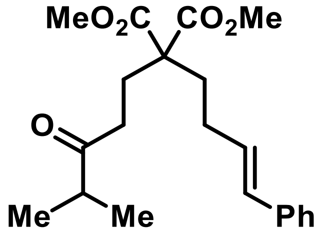

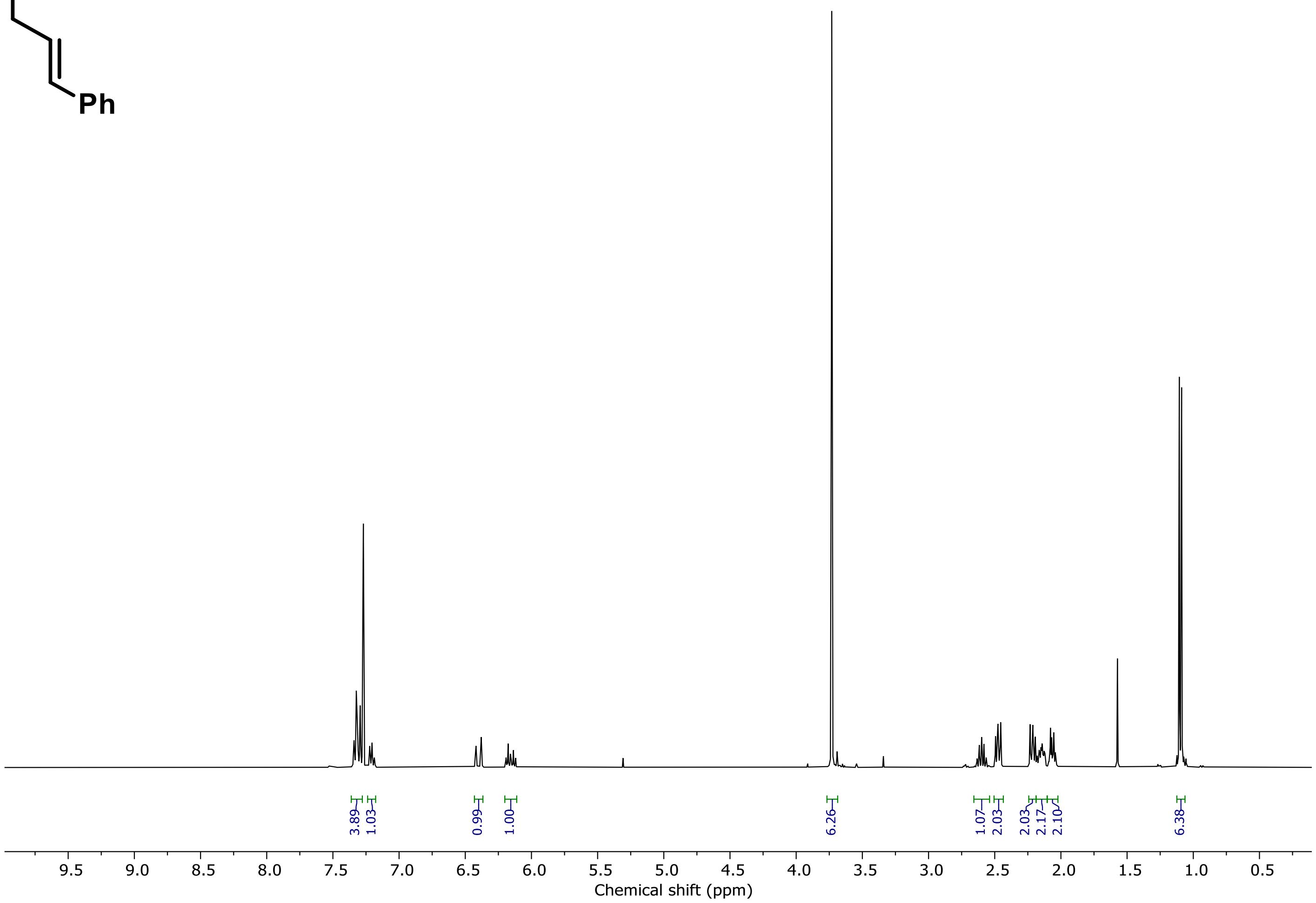

^{13}C NMR (101 MHz, $CDCl_3$)

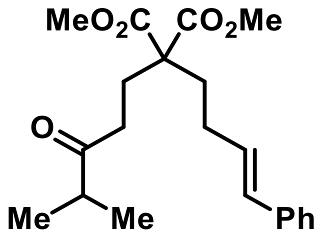

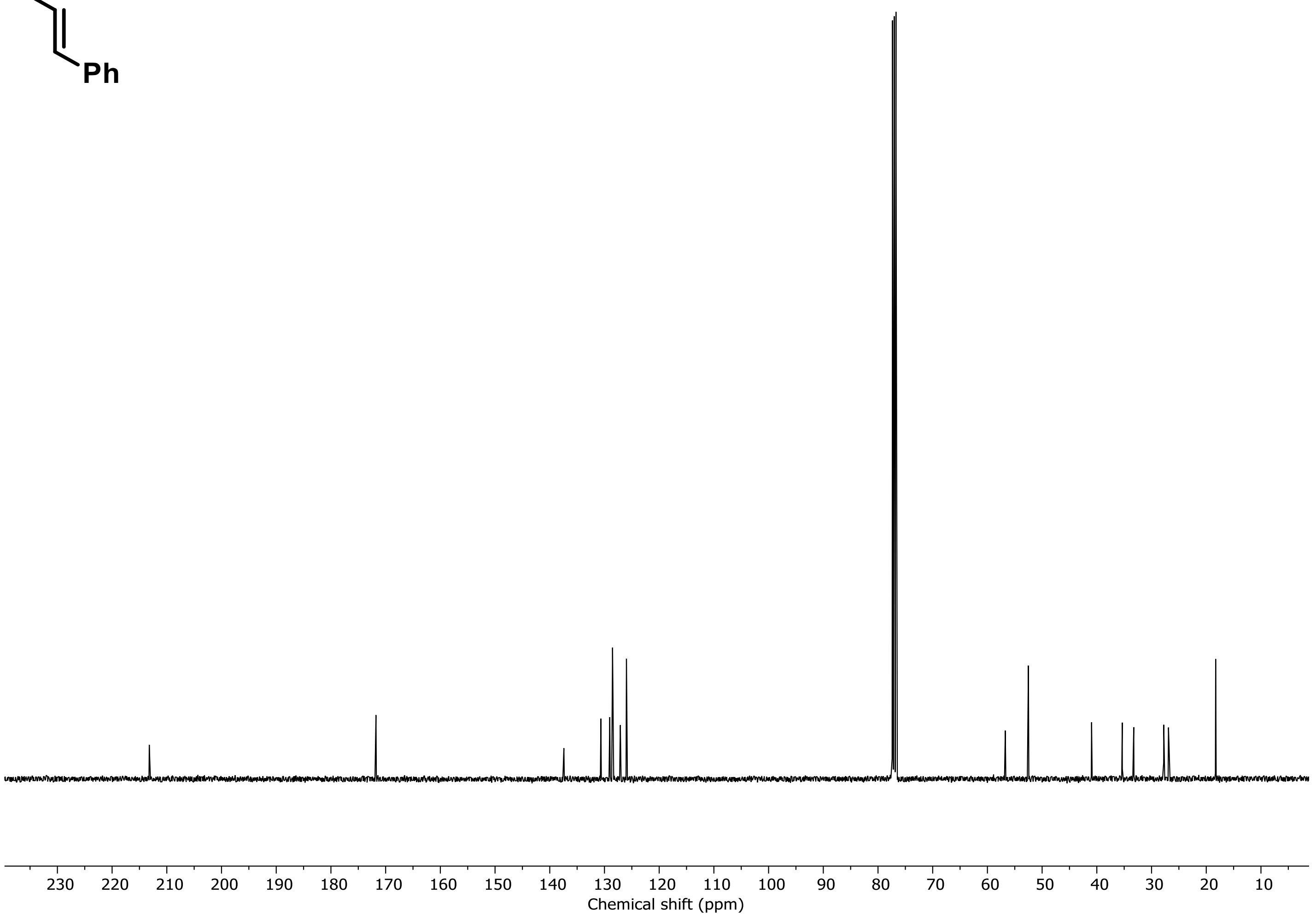

Dimethyl (*E*)–2–(3–oxo–4,4–diphenylbutyl)–2–(4–phenylbut–3–en–1–yl)malonate (S14)

^{1}H NMR (400 MHz, $CDCl_3$)

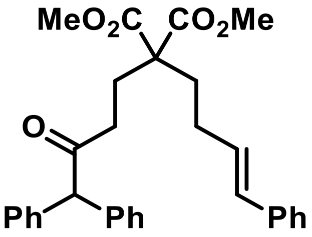

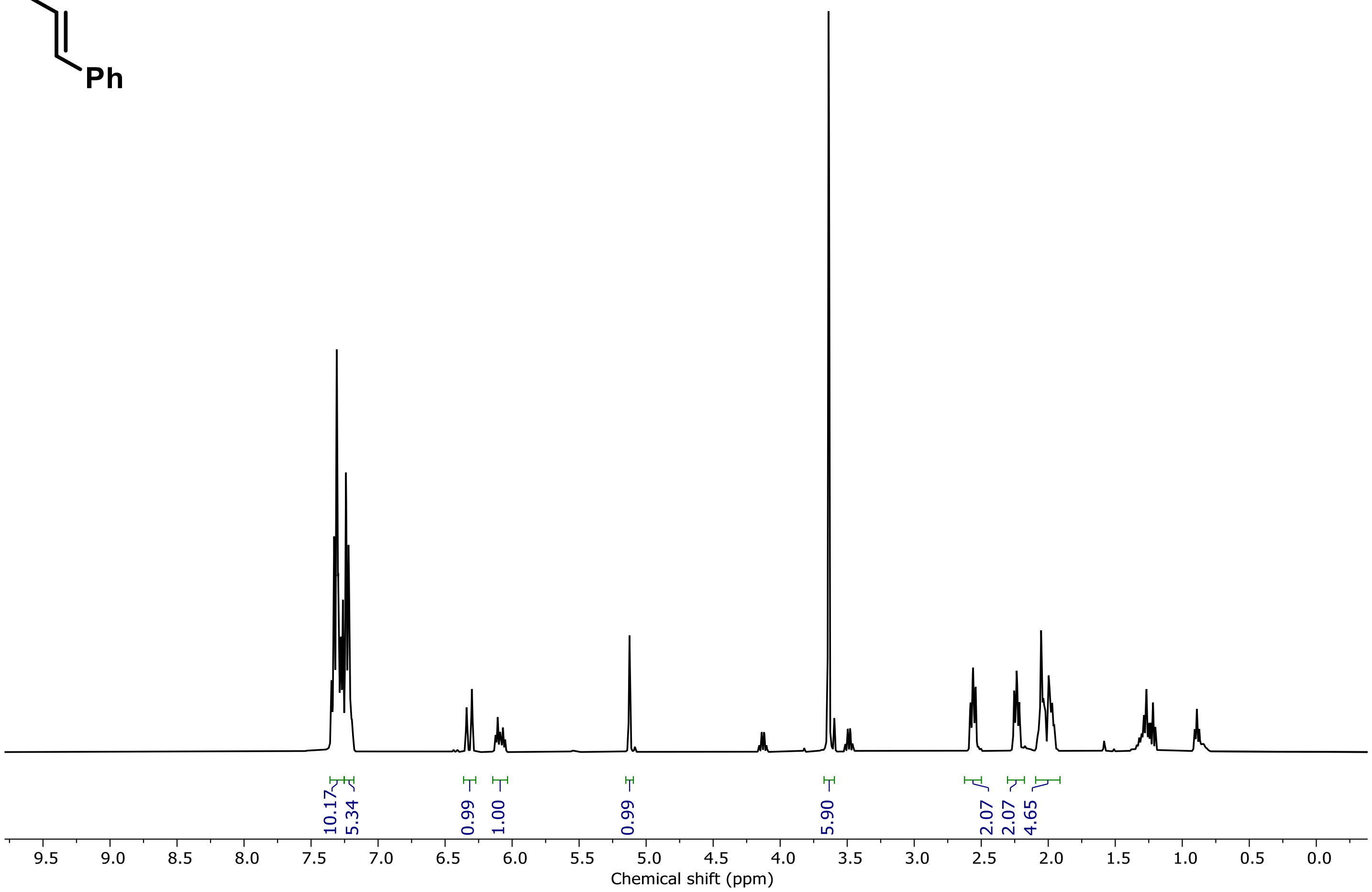

^{13}C NMR (101 MHz, $CDCl_3$)

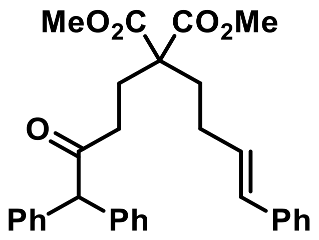

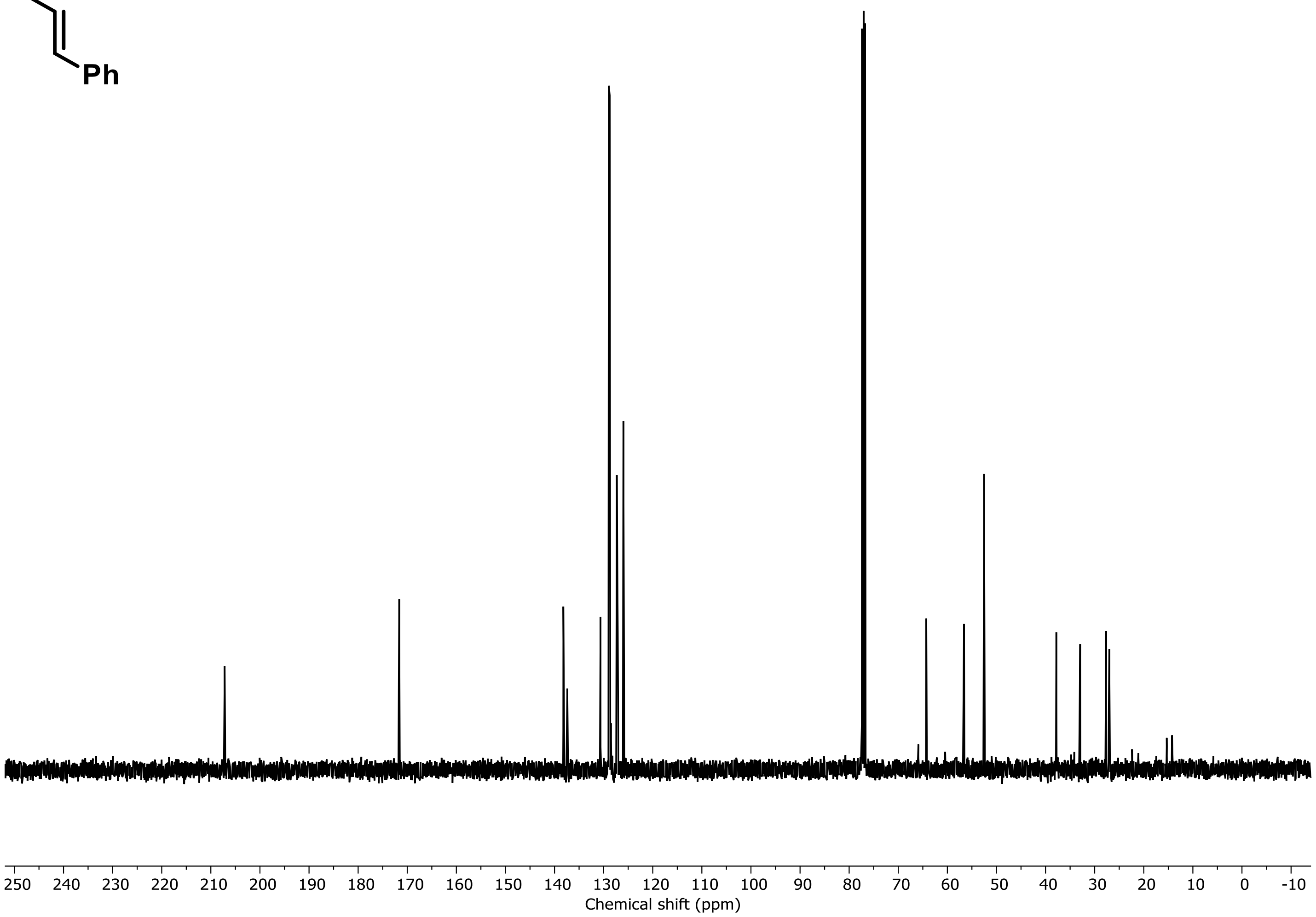

Dimethyl (*E*)–2–(3–cyclohexyl–3–oxopropyl)–2–(4–phenylbut–3–en–1–yl)malonate (S16)

^{1}H NMR (500 MHz, $CDCl_3$)

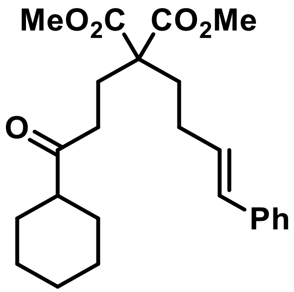

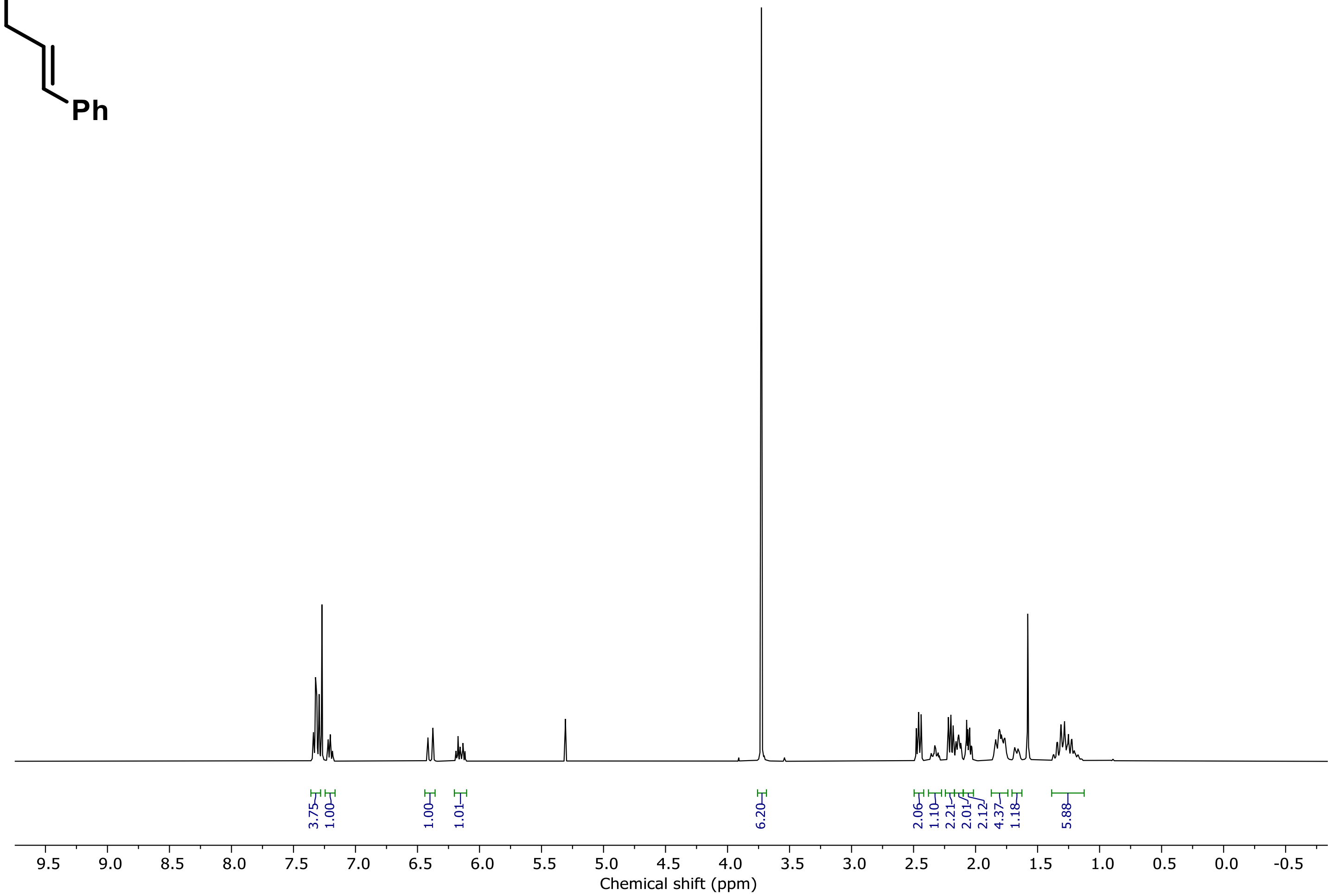

^{13}C NMR (101 MHz, $CDCl_3$)

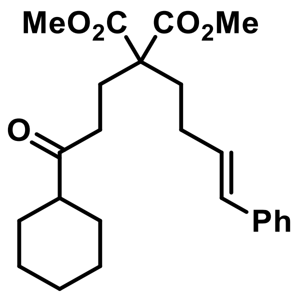

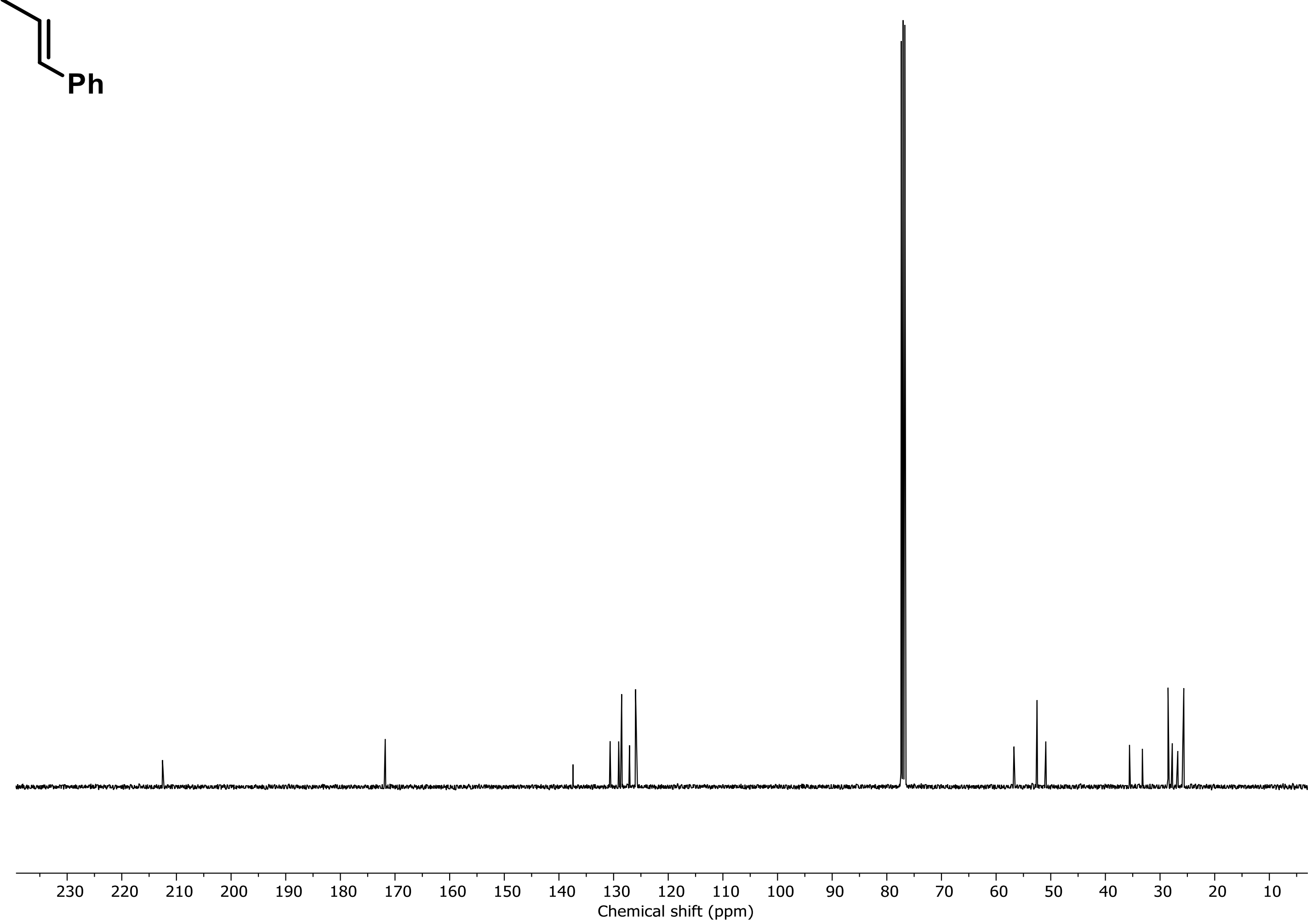

Dimethyl (*E*)–2–(3–oxo–3–(tetrahydro–2*H*–pyran–4–yl)propyl)–2–(4–phenylbut–3–en–1–yl) malonate (S18)

^{1}H NMR (400 MHz, $CDCl_3$)

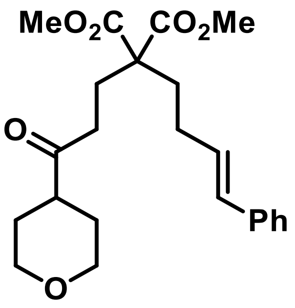

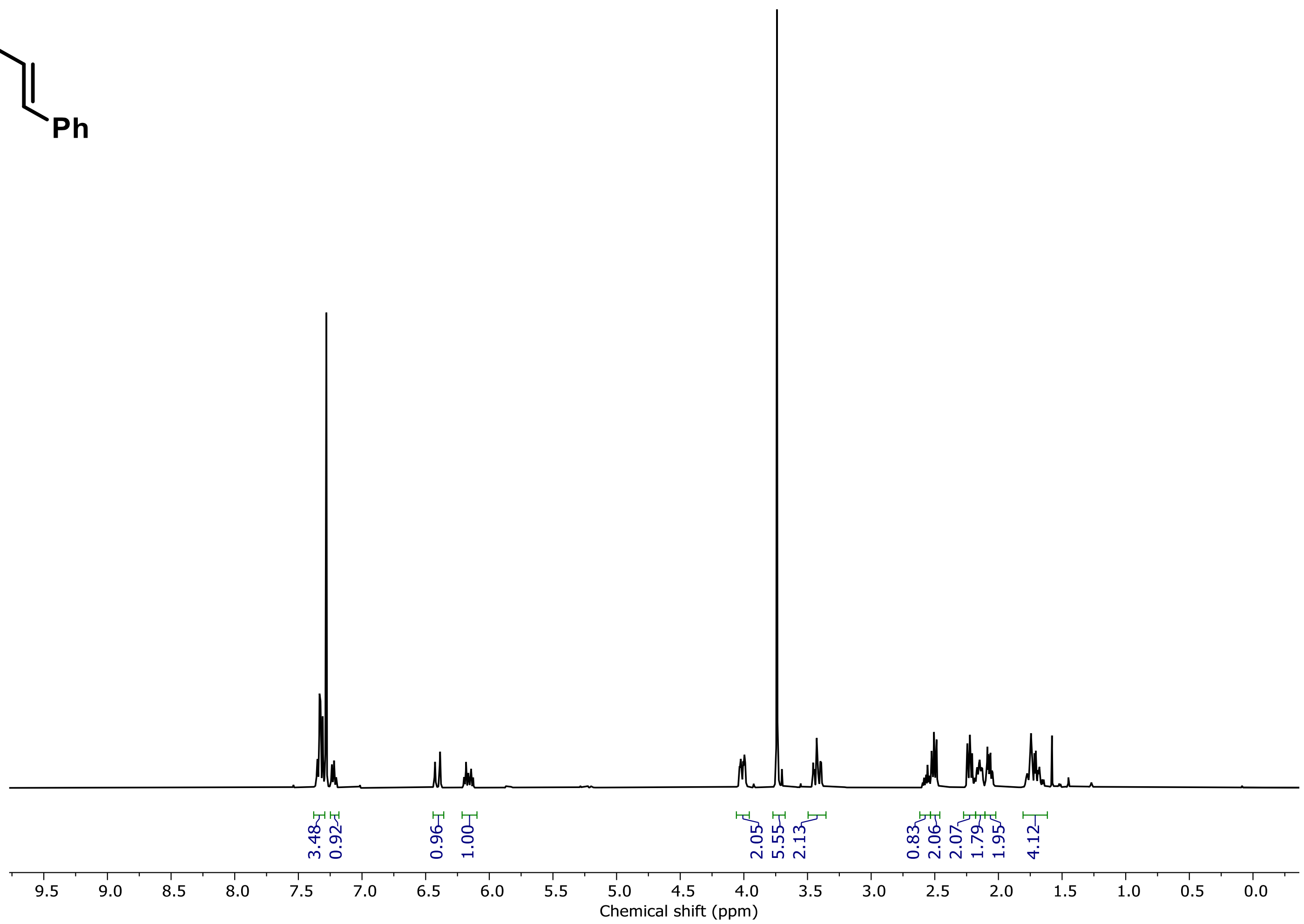

^{13}C NMR (101 MHz, $CDCl_3$)

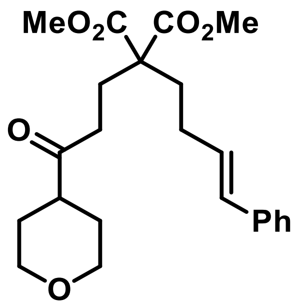

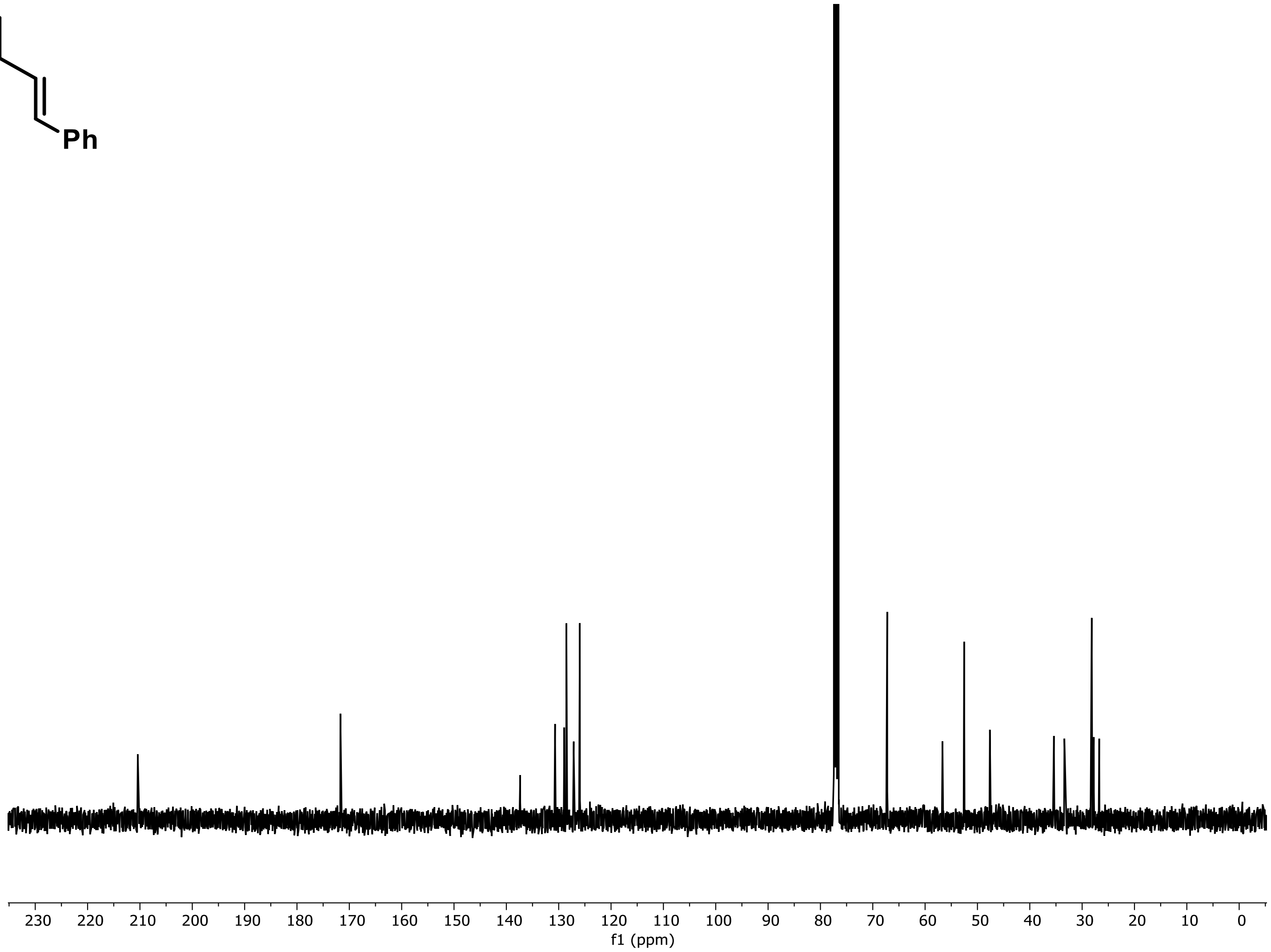

Dimethyl (*E*)–2–(4,4–dimethyl–3–oxopentyl)–2–(4–phenylbut–3–en–1–yl)malonate (S20)

^{1}H NMR (400 MHz, $CDCl_3$)

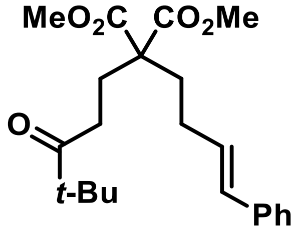

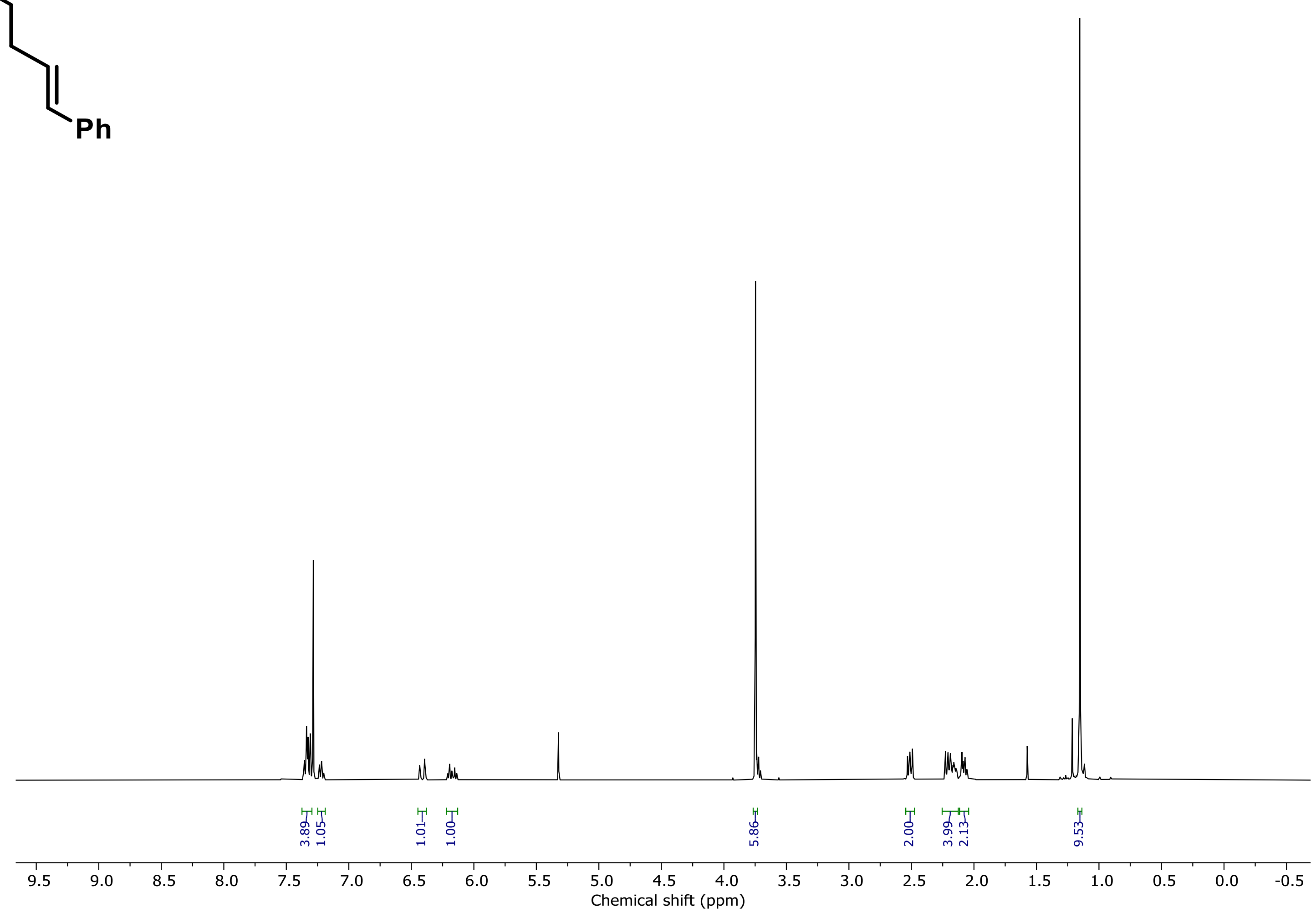

^{13}C NMR (101 MHz, $CDCl_3$)

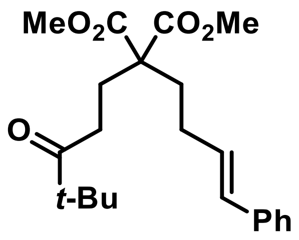

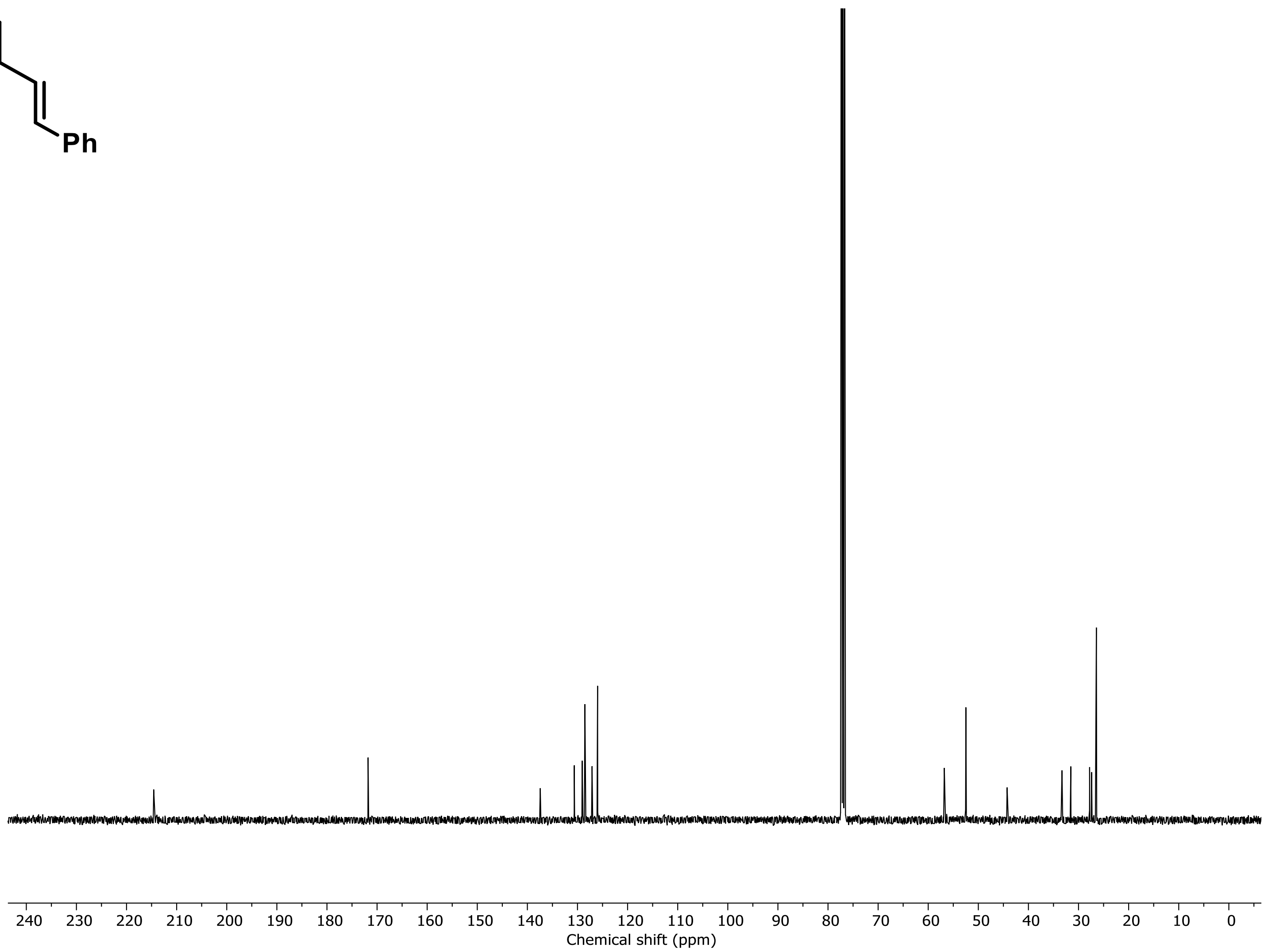

Dimethyl 2–(but–3–en–1–yl)–2–(3–oxopentyl)malonate (S21)

^{1}H NMR (400 MHz, $CDCl_3$)

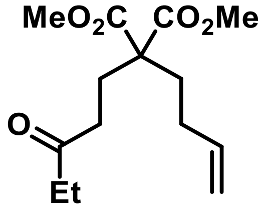

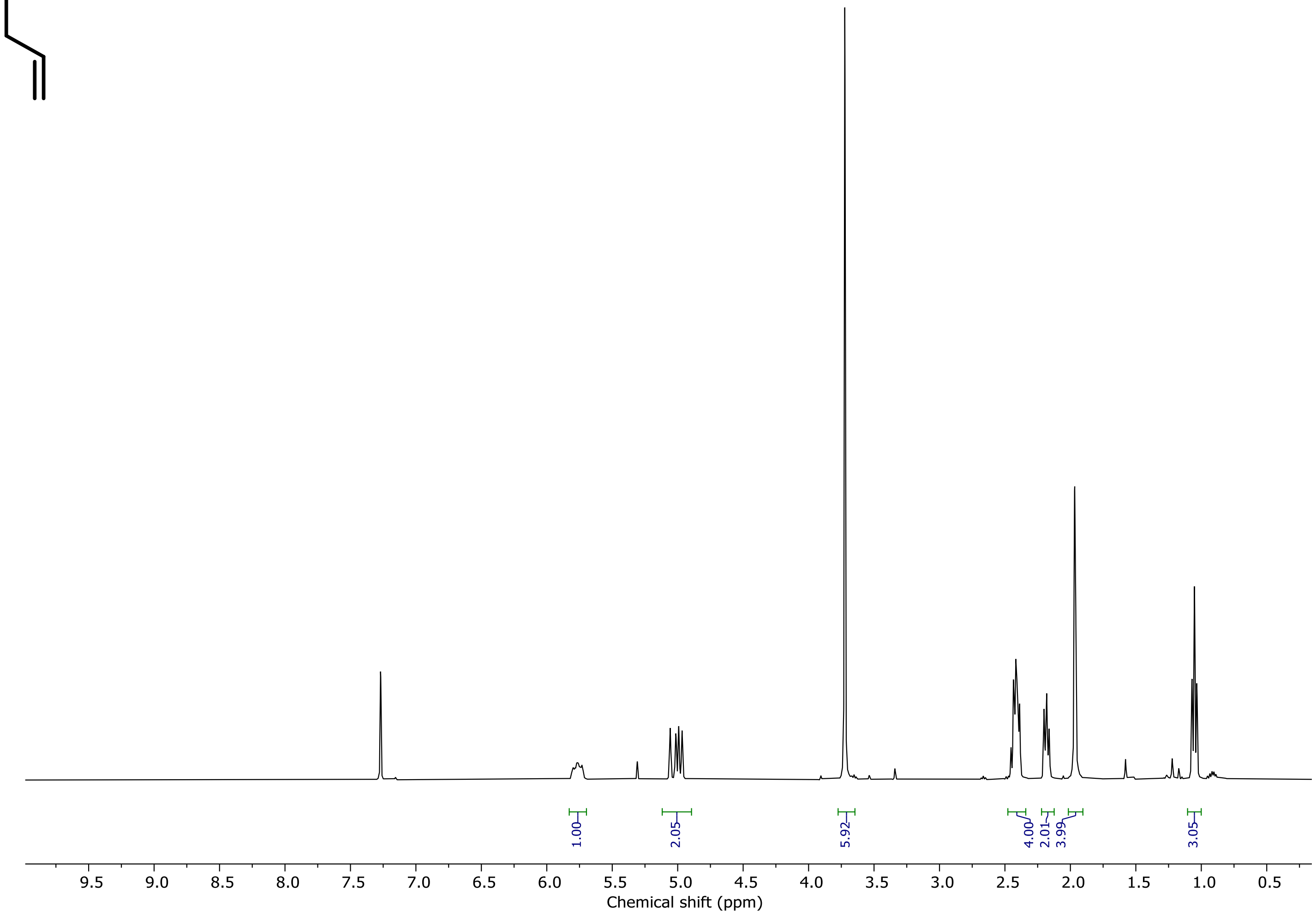

^{13}C NMR (101 MHz, $CDCl_3$)

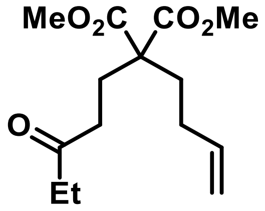

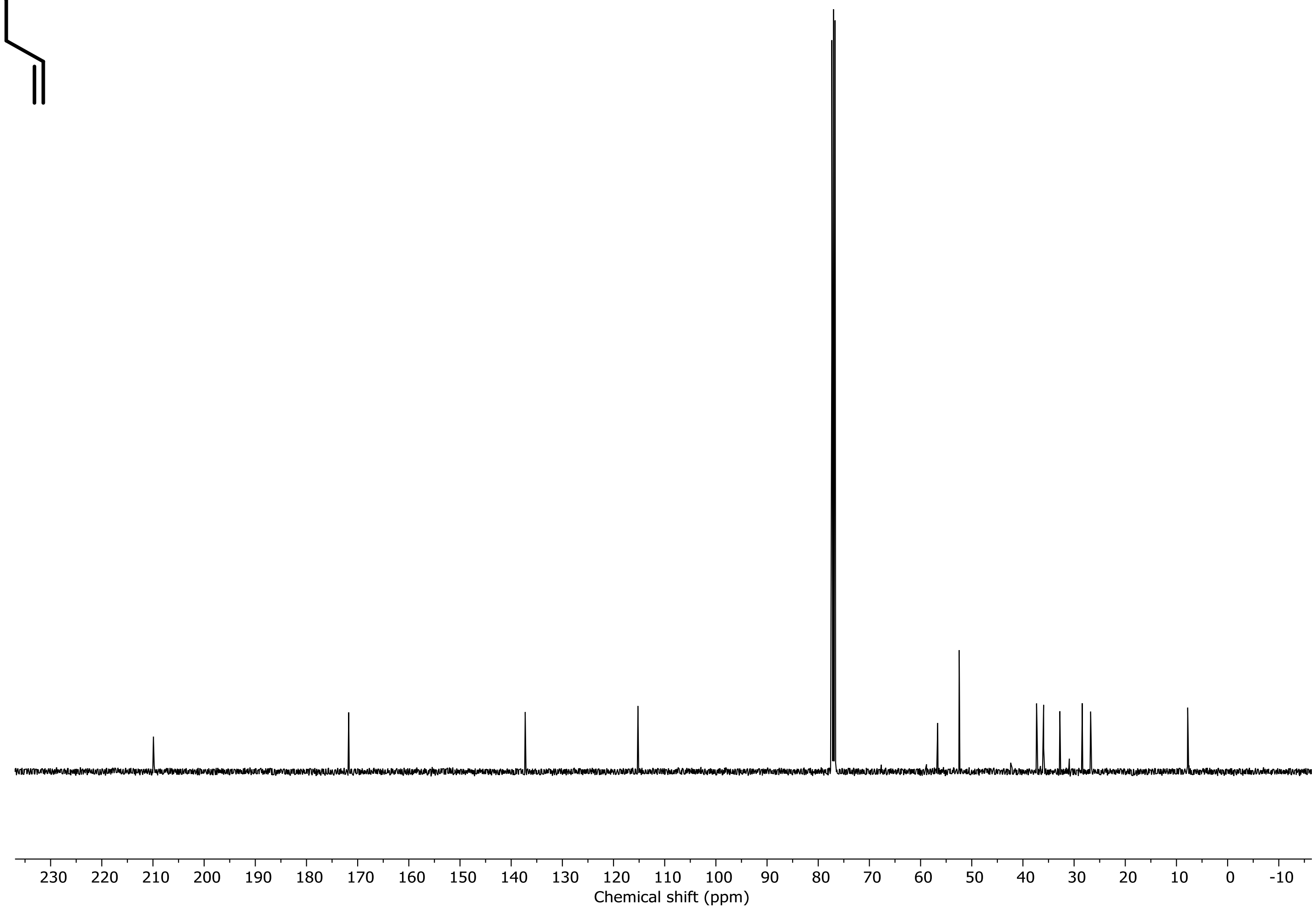

Dimethyl (*E*)–2–(4–(2–fluorophenyl)but–3–en–1–yl)–2–(3–oxopentyl)malonate (S22)

^{1}H NMR (500 MHz, $CDCl_3$)

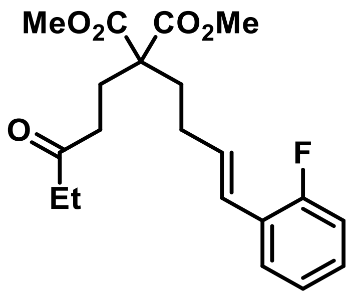

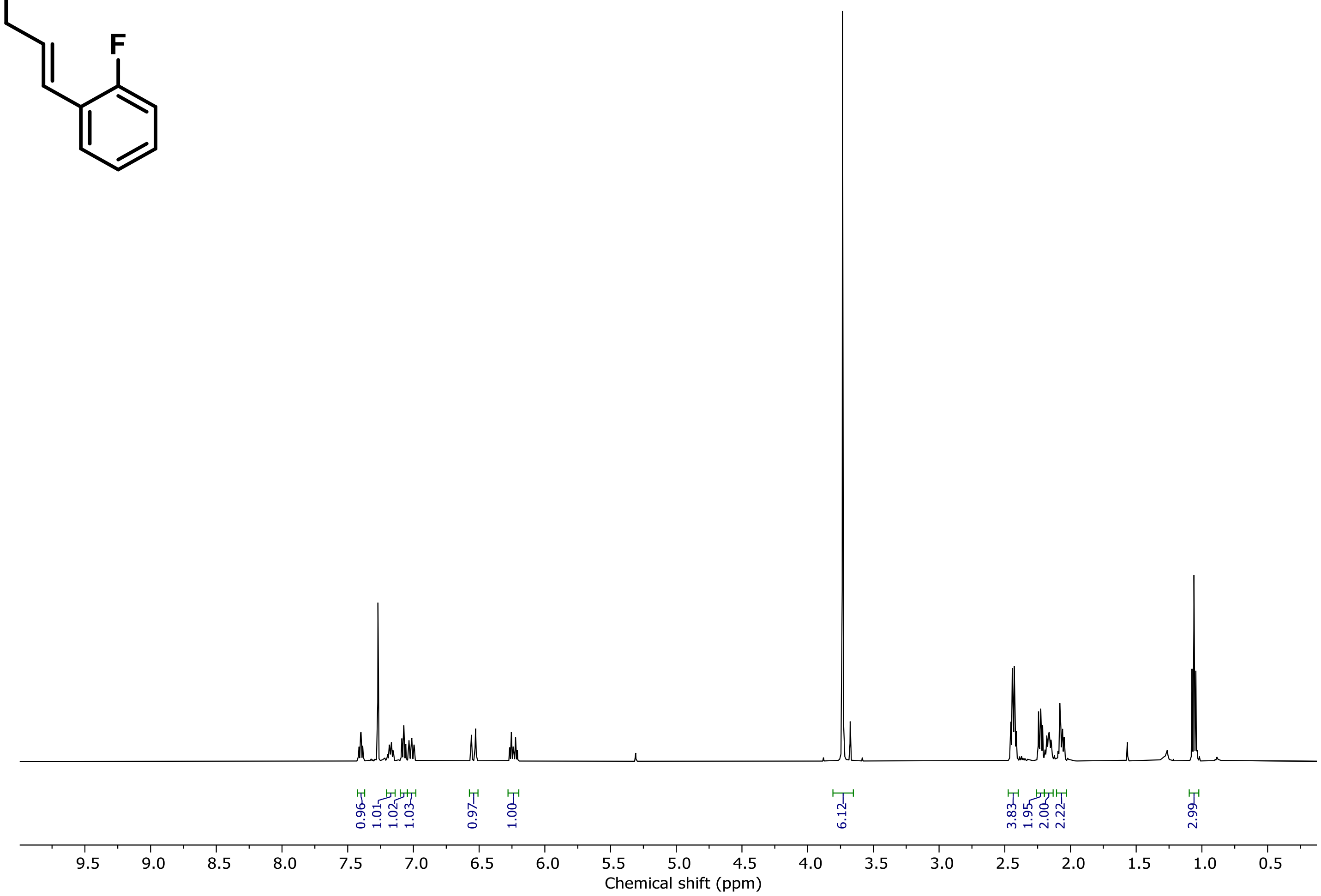

^{13}C NMR (126 MHz, $CDCl_3$)

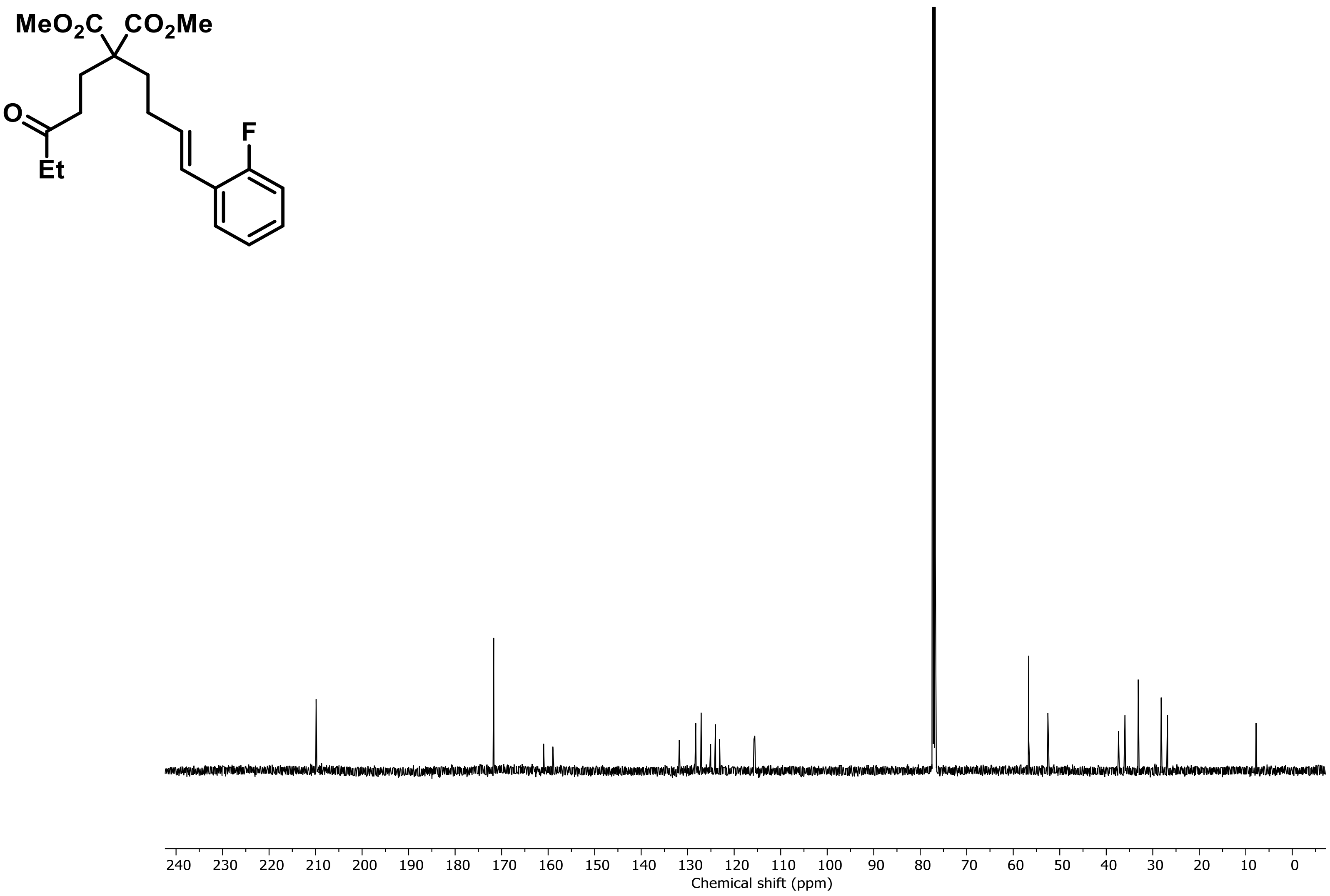

^{19}F NMR (376 MHz, $CDCl_3$)

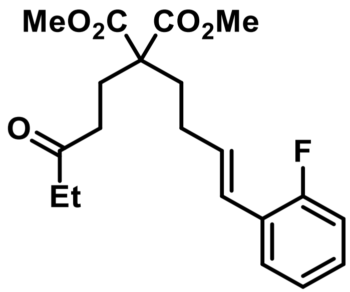

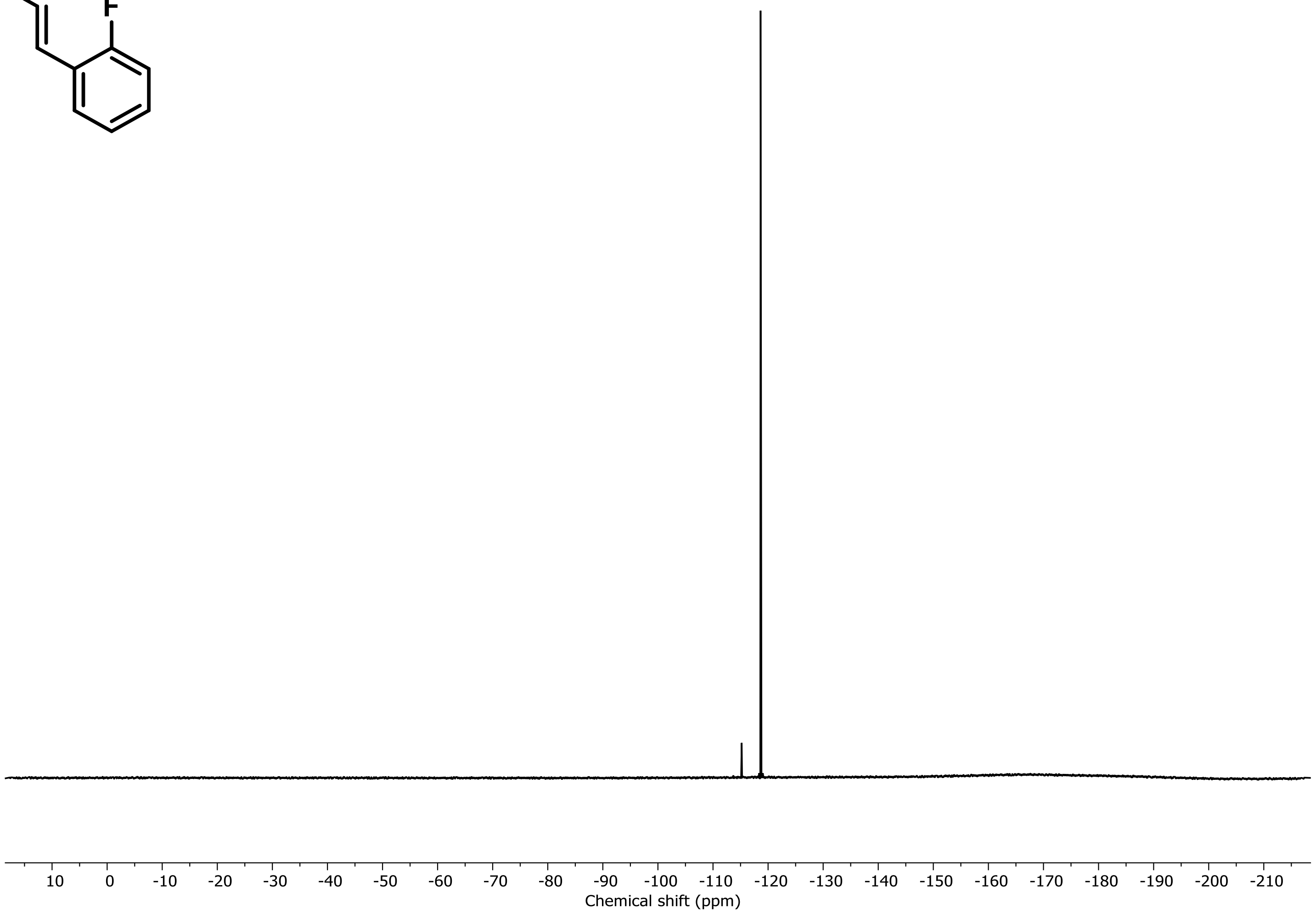

Dimethyl (*E*)–2–(3–oxopentyl)–2–(4–(o–tolyl)but–3–en–1–yl)malonate (S23)

^{1}H NMR (400 MHz, $CDCl_3$)

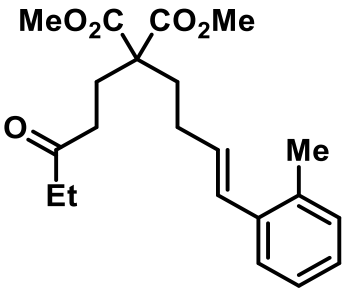

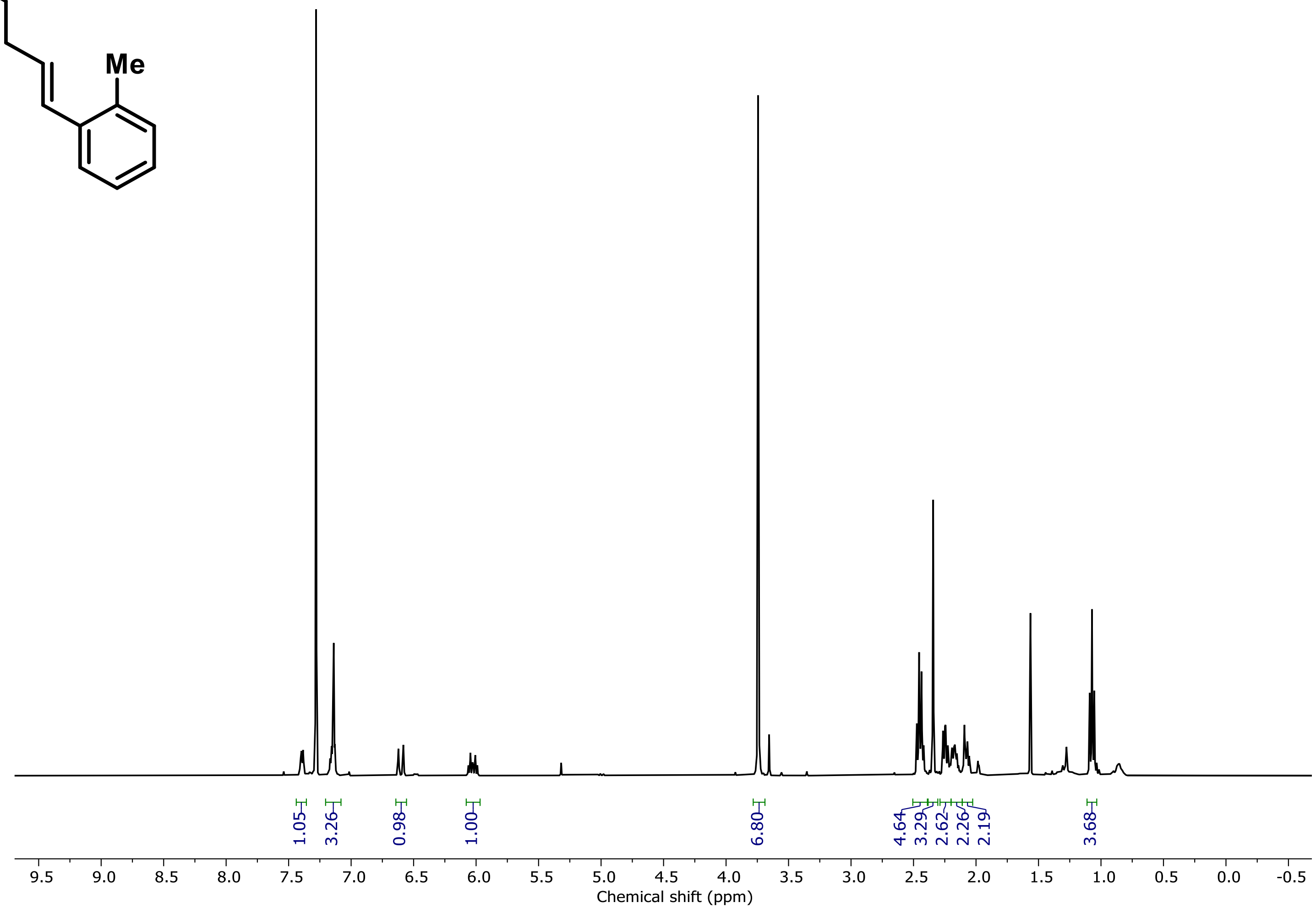

^{13}C NMR (101 MHz, $CDCl_3$)

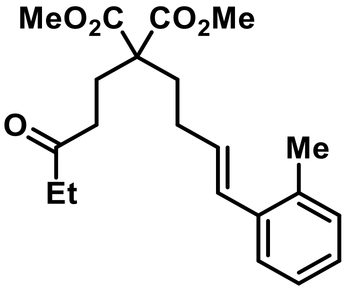

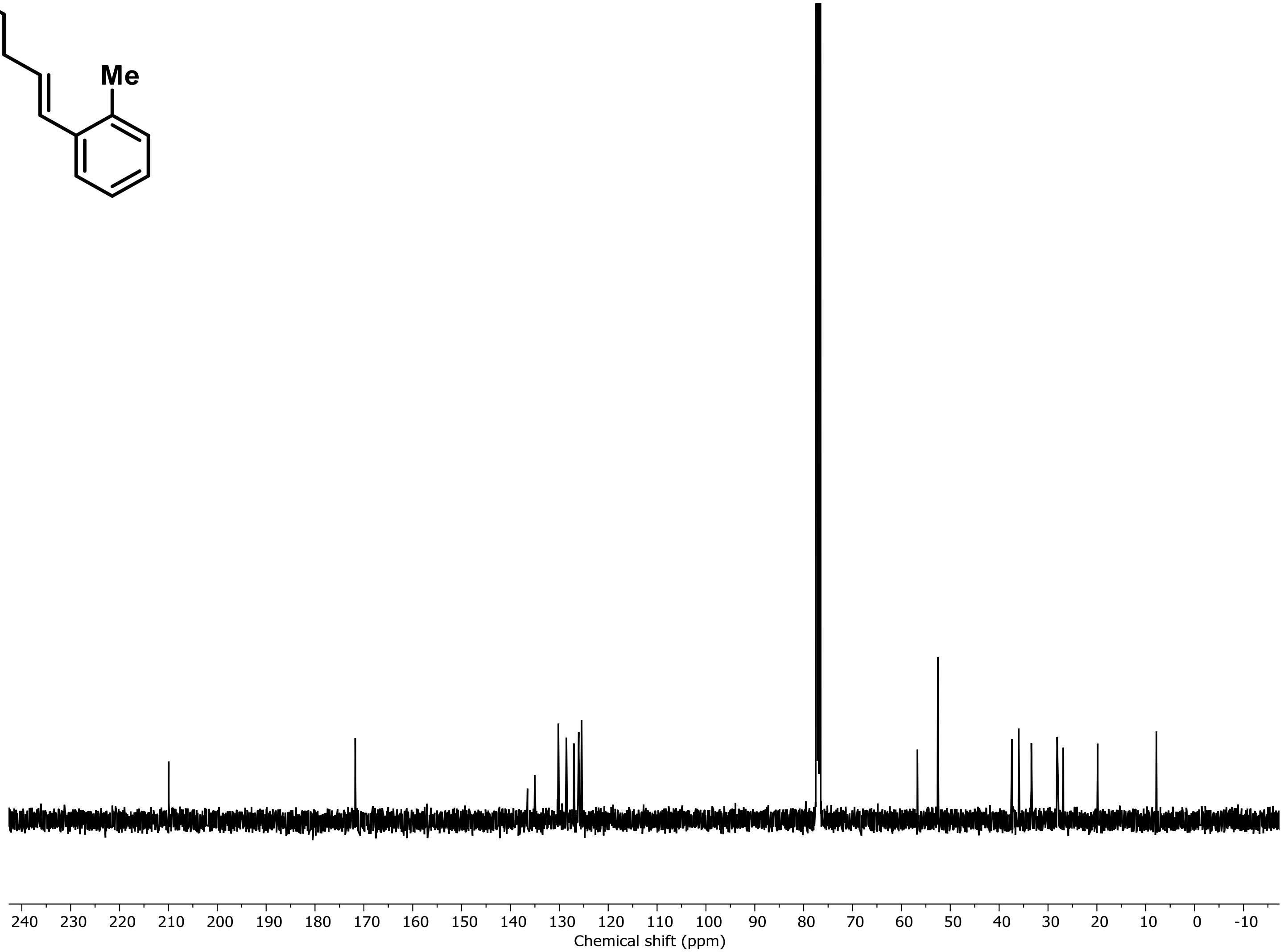

Dimethyl (*E*)–2–(3–oxopentyl)–2–(4–(m–tolyl)but–3–en–1–yl)malonate (S24)

^{1}H NMR (400 MHz, $CDCl_3$)

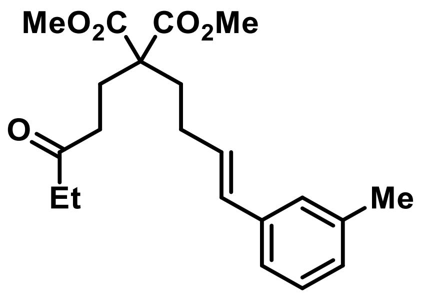

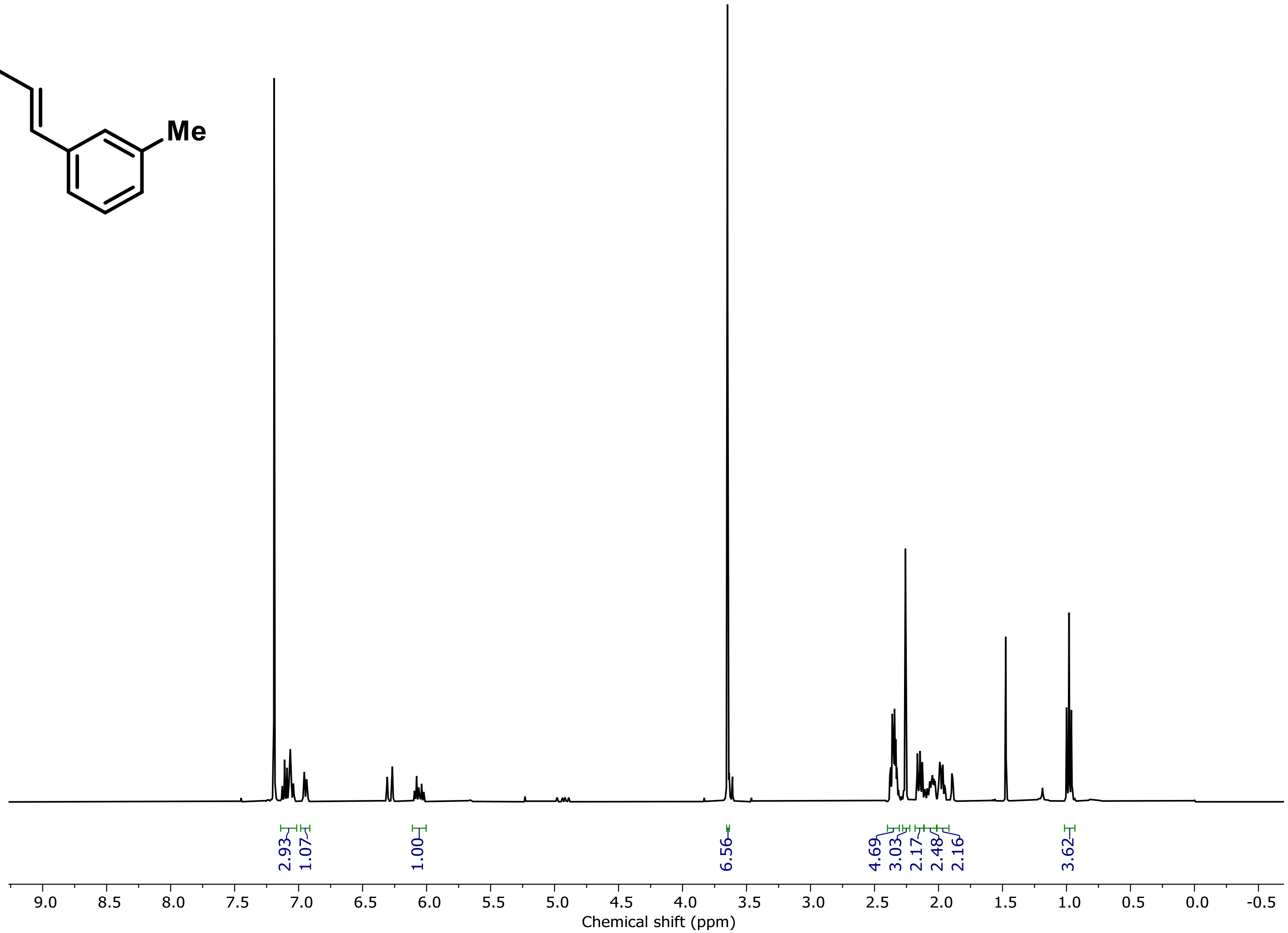

^{13}C NMR (101 MHz, $CDCl_3$)

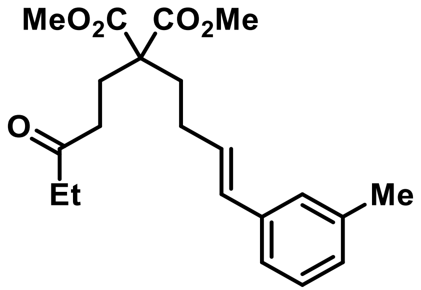

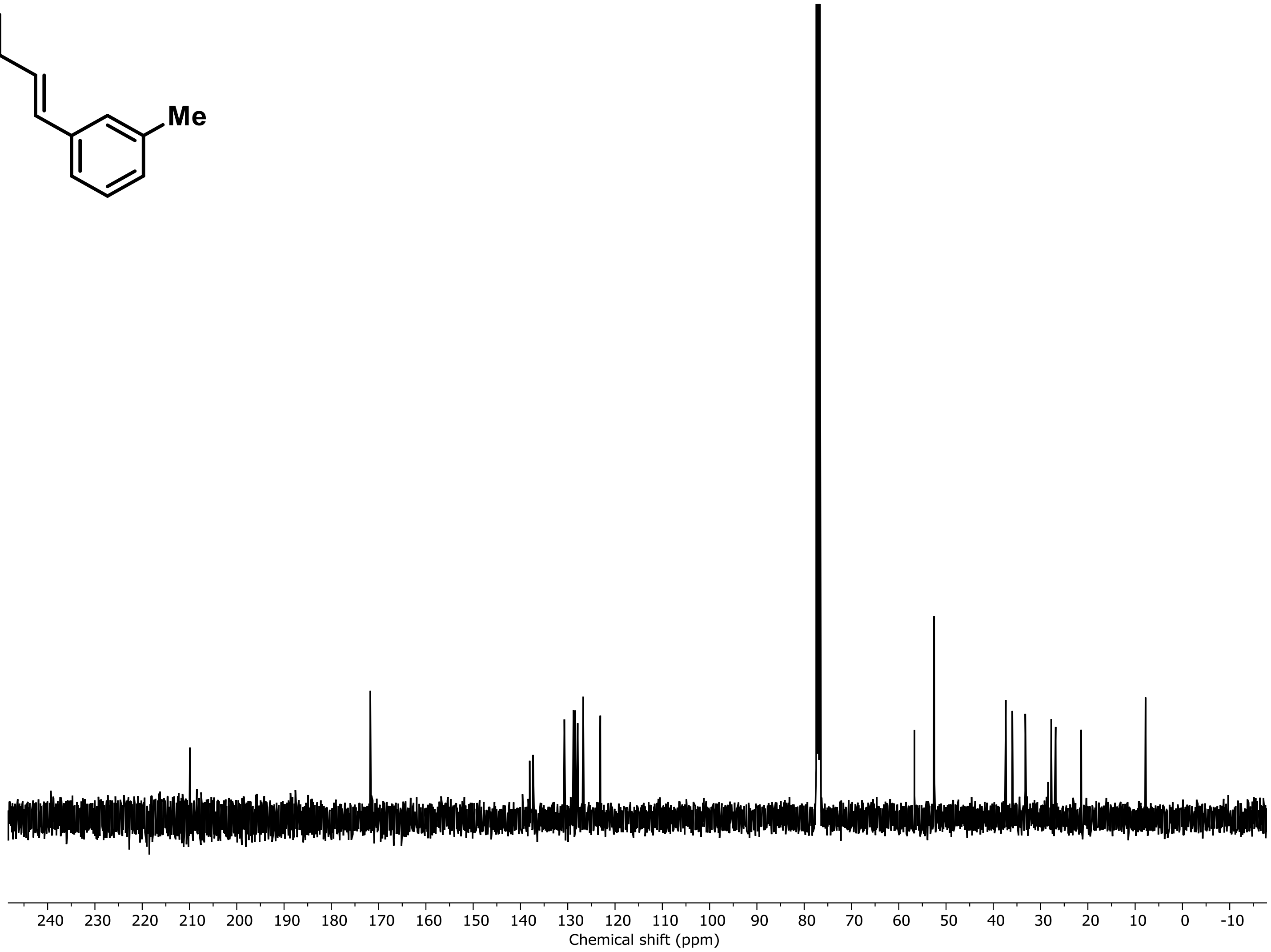

Dimethyl (*E*)–2–(4–(3–chlorophenyl)but–3–en–1–yl)–2–(3–oxopentyl)malonate (S25)

^{1}H NMR (400 MHz, $CDCl_3$)

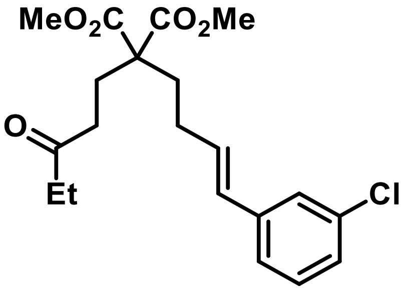

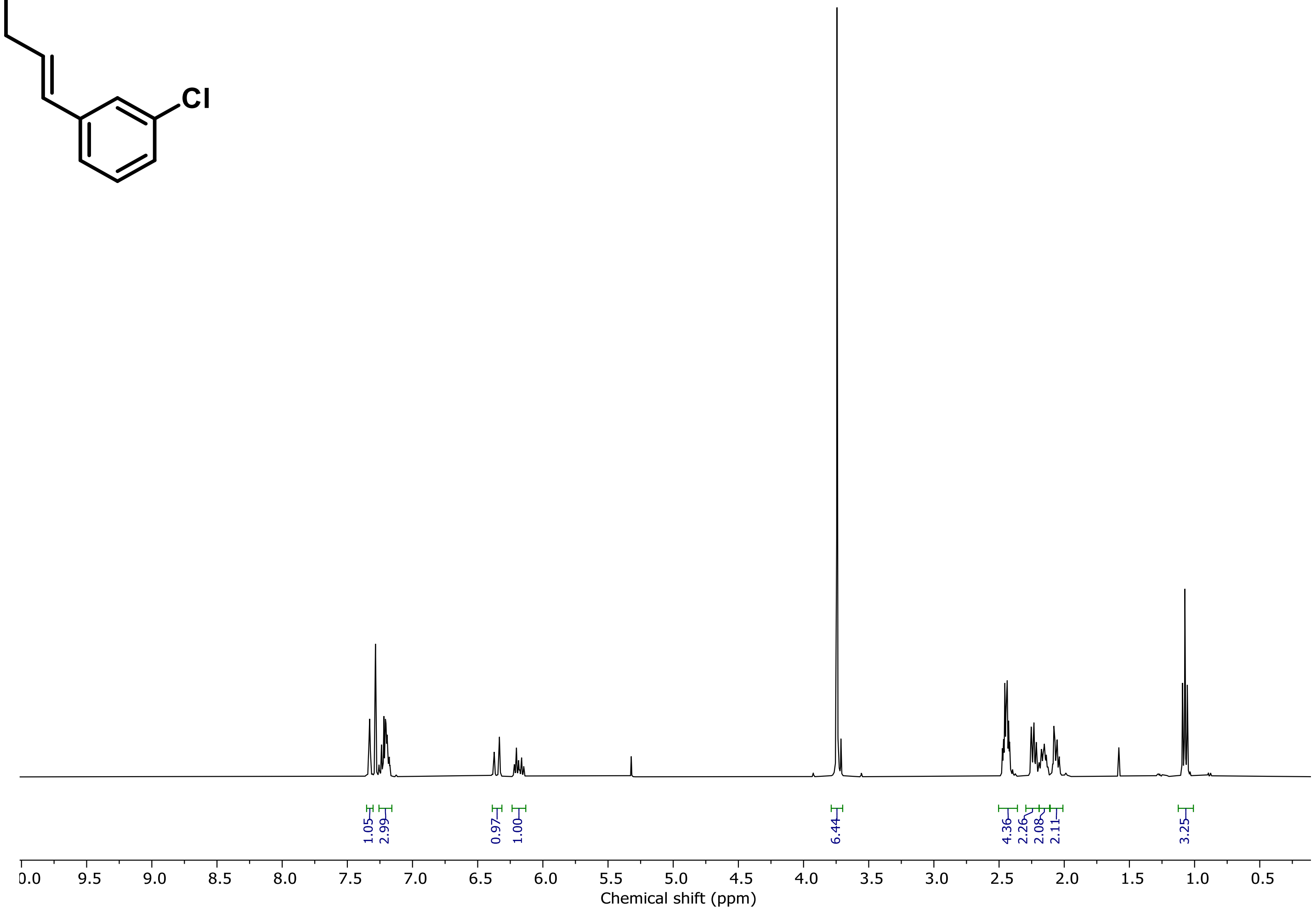

^{13}C NMR (101 MHz, $CDCl_3$)

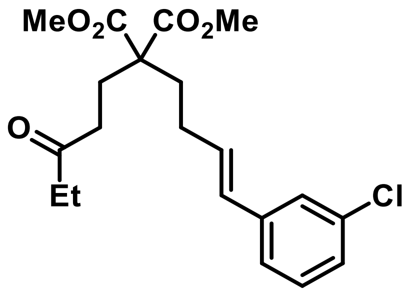

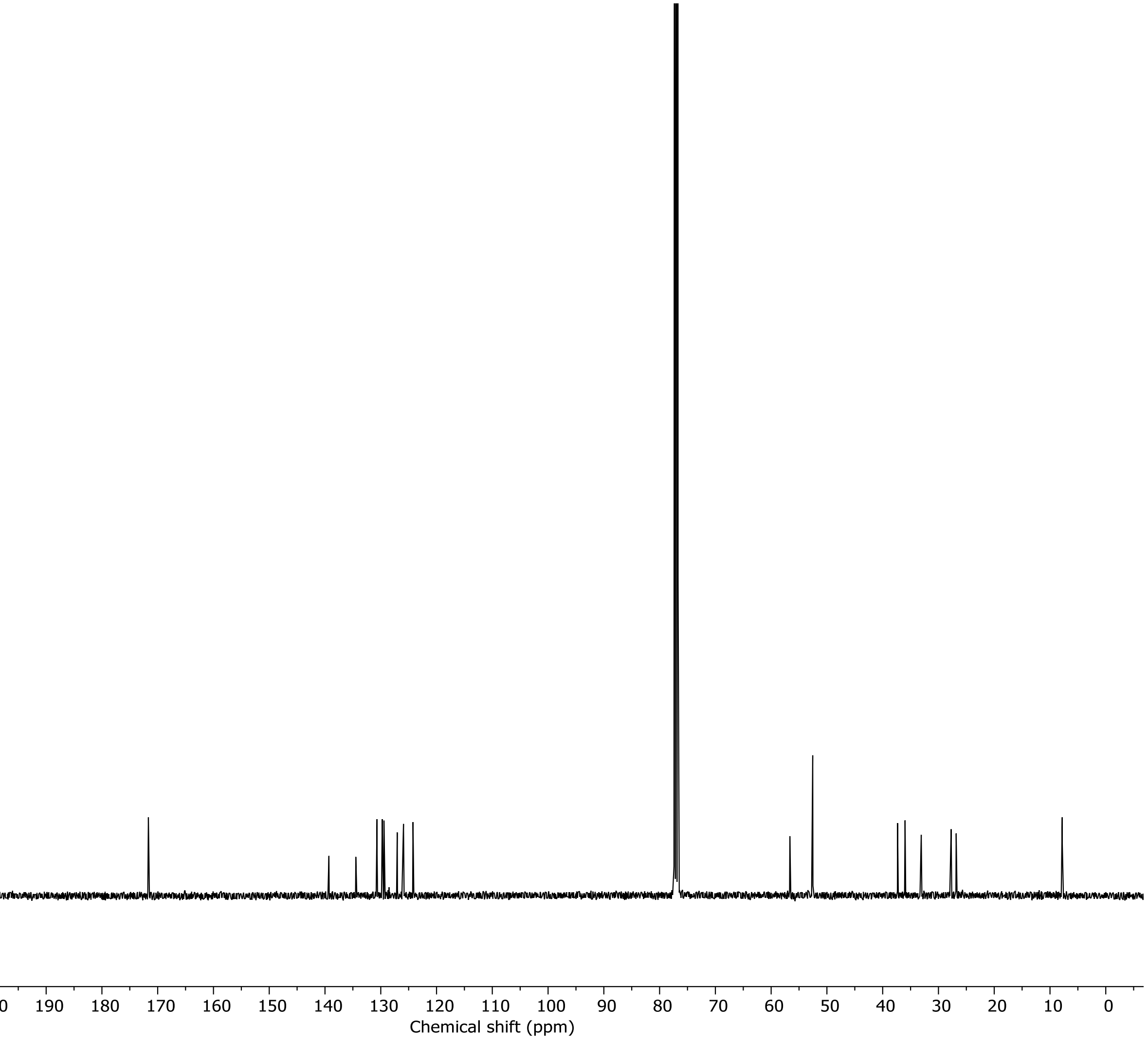

Dimethyl (*E*)–2–(4–(naphthalen–1–yl)but–3–en–1–yl)–2–(3–oxopentyl)malonate (S26)

^{1}H NMR (500 MHz, $CDCl_3$)

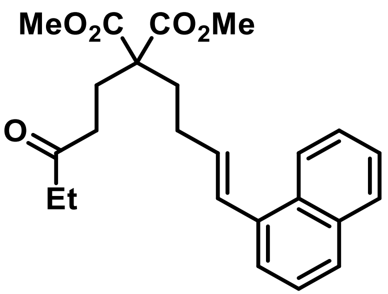

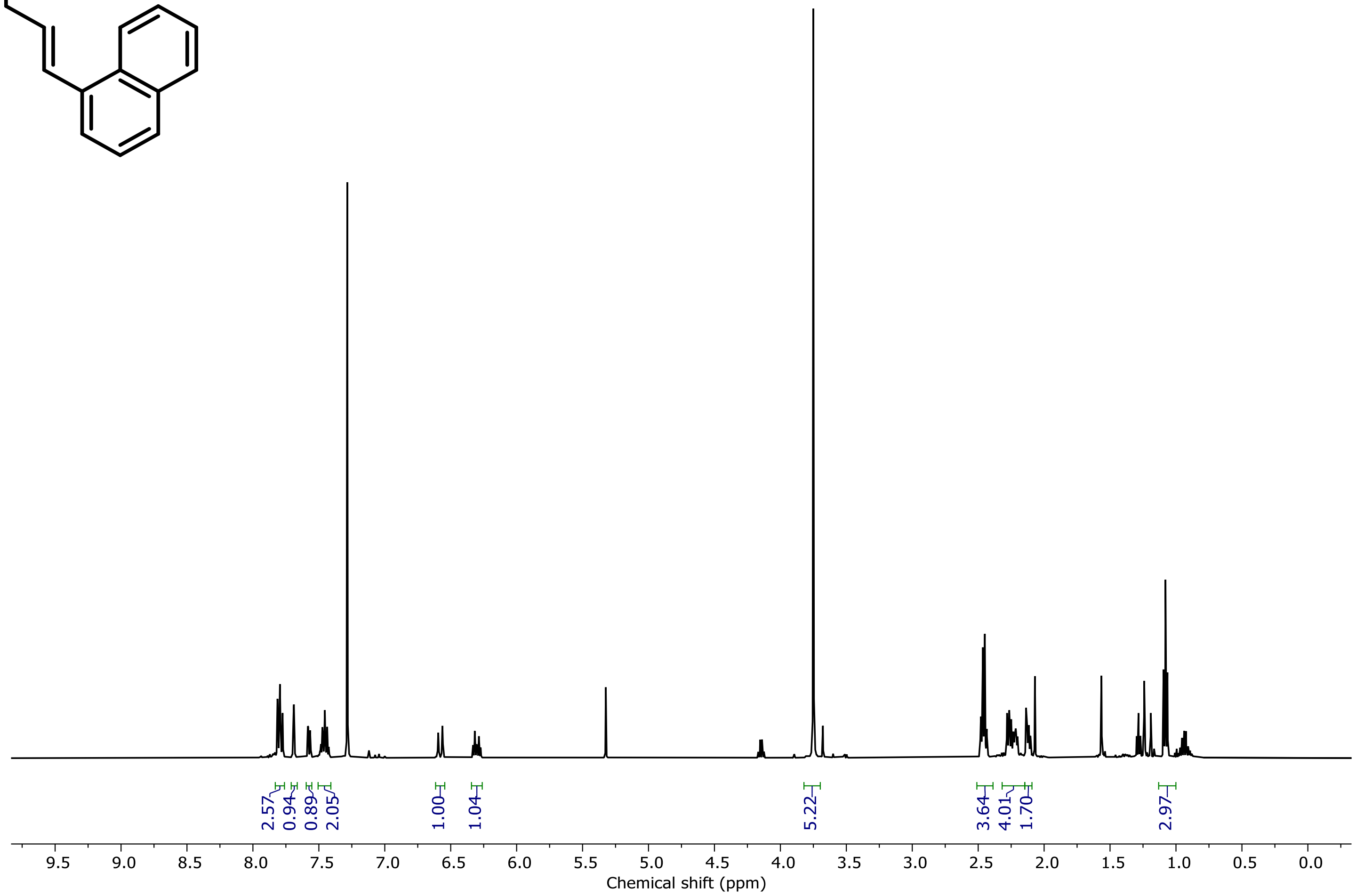

^{13}C NMR (126 MHz, $CDCl_3$)

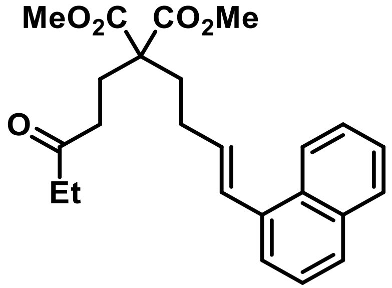

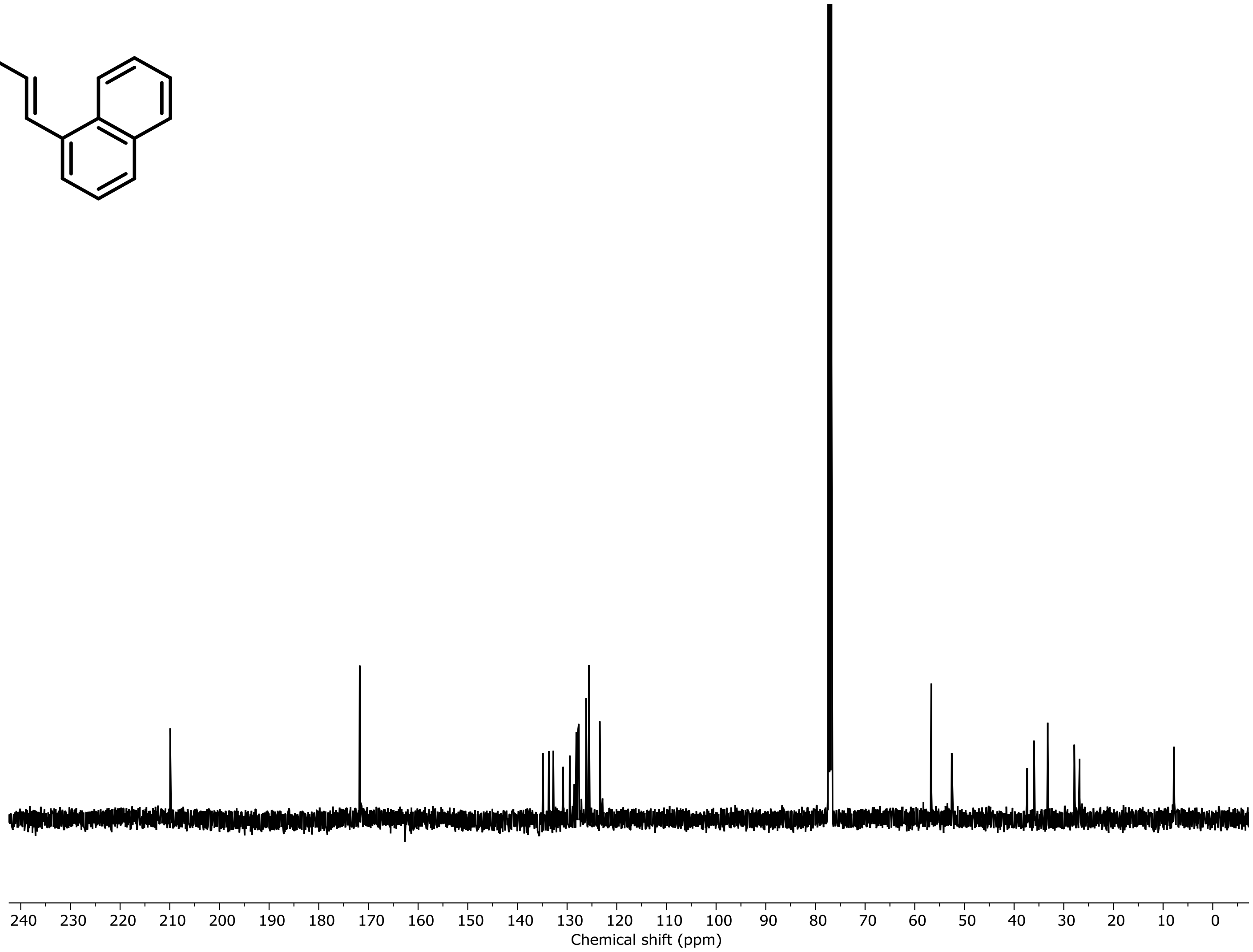

Dimethyl (*E*)–2–(4–([1,1'–biphenyl]–4–yl)but–3–en–1–yl)–2–(3–oxopentyl)malonate (S27)

^{1}H NMR (400 MHz, $CDCl_3$)

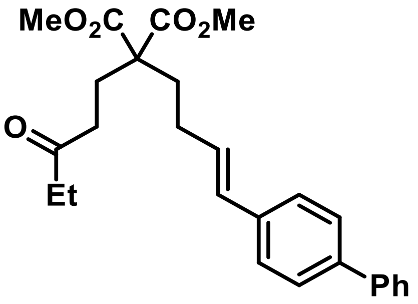

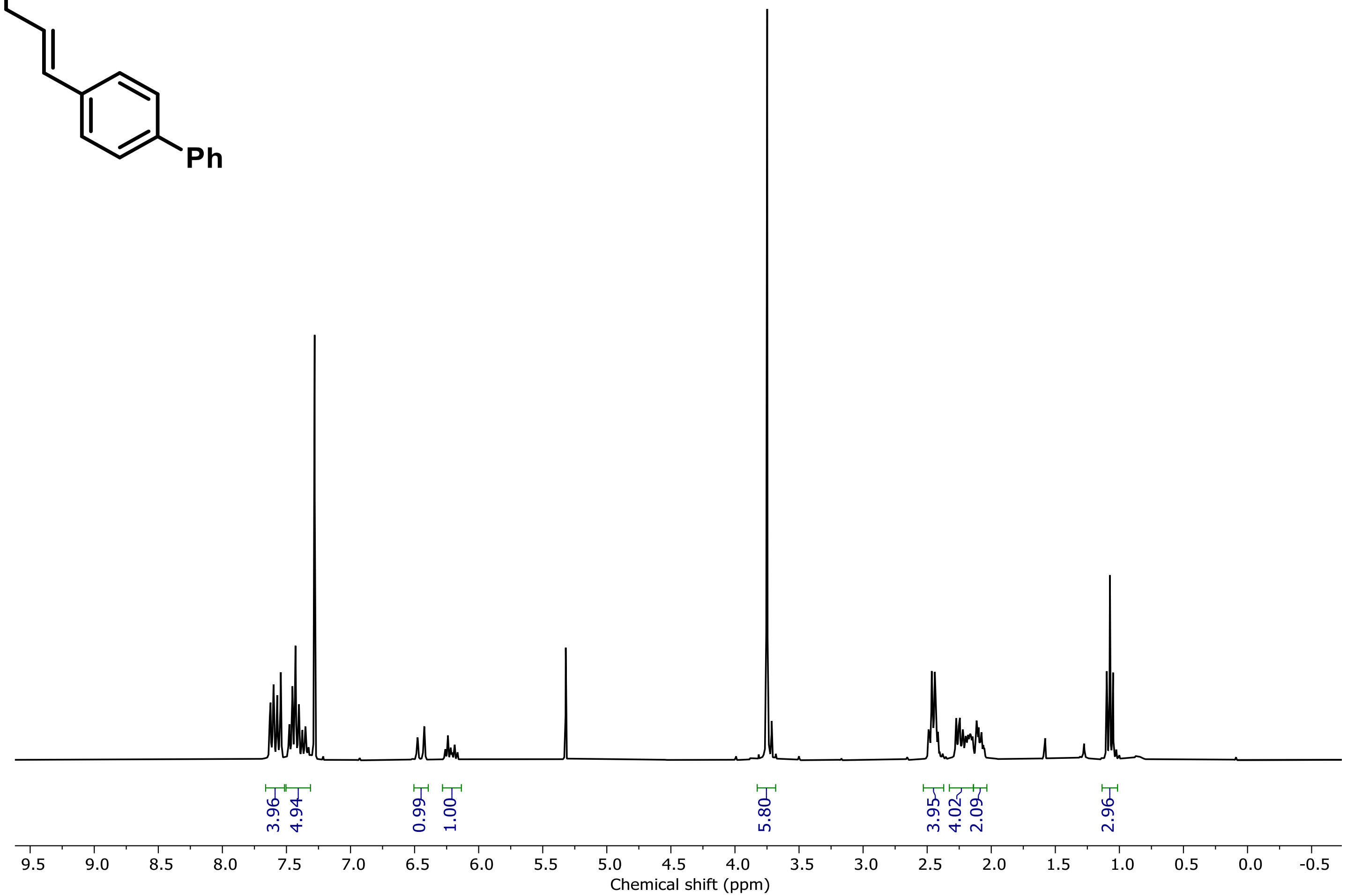

^{13}C NMR (126 MHz, CD_2Cl_2)

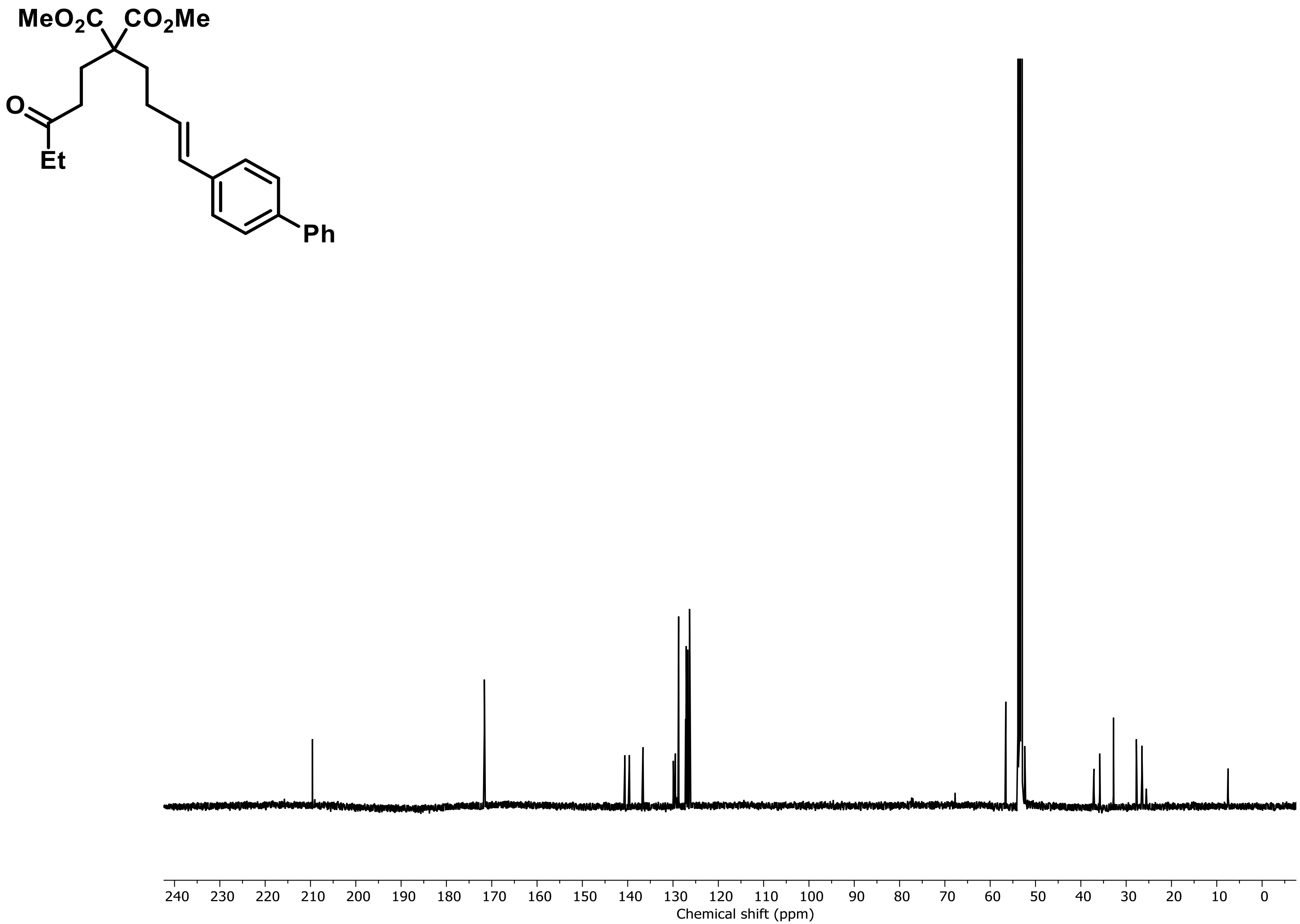

Dimethyl (*E*)–2–(4–(4–fluorophenyl)but–3–en–1–yl)–2–(3–oxopentyl)malonate (S28)

^{1}H NMR (400 MHz, $CDCl_3$)

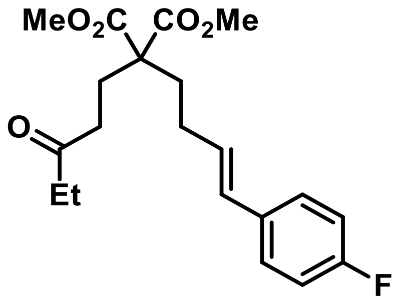

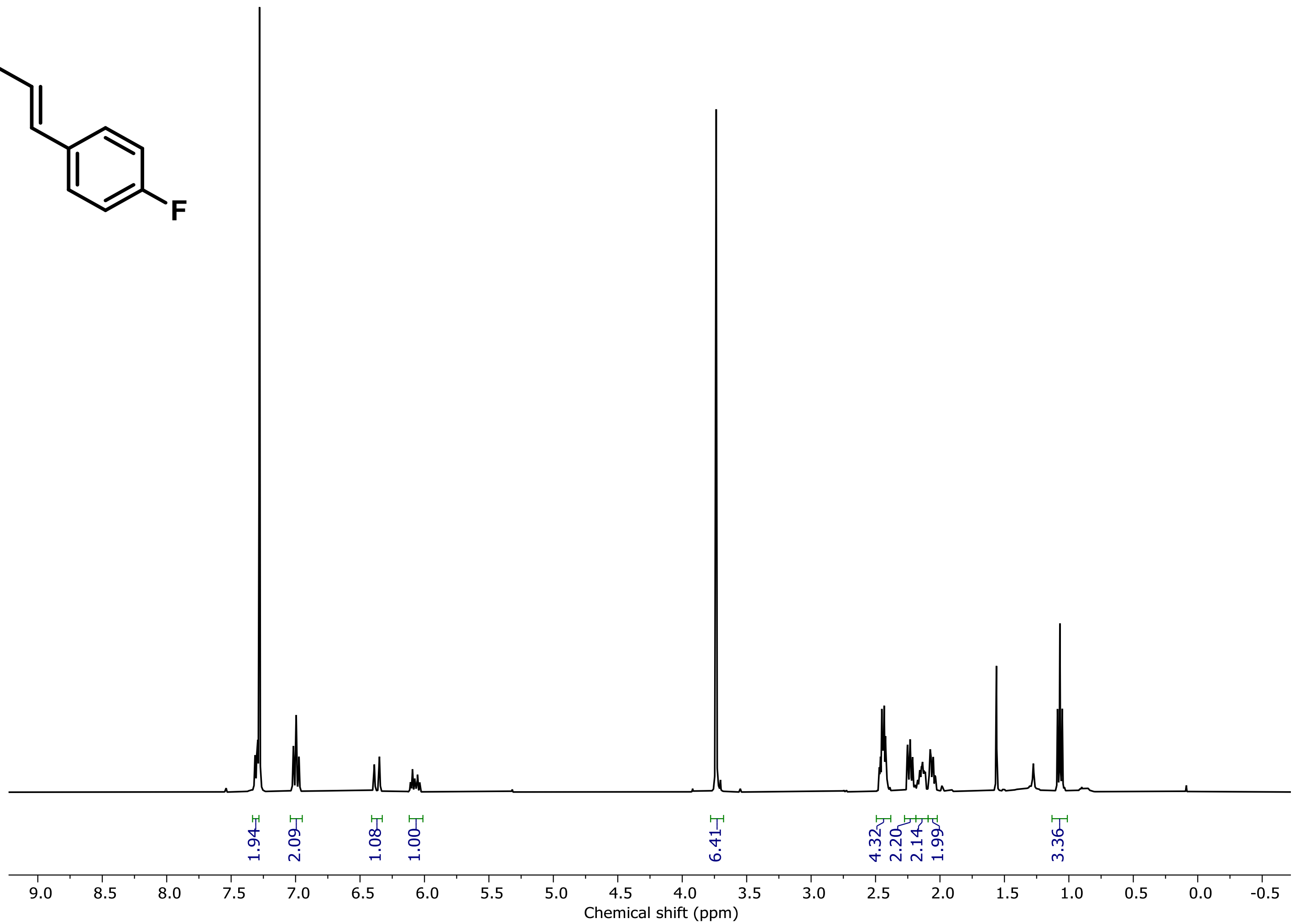

^{13}C NMR (126 MHz, $CDCl_3$)

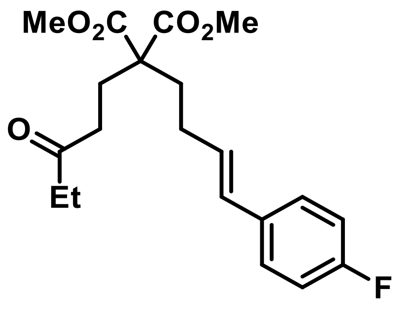

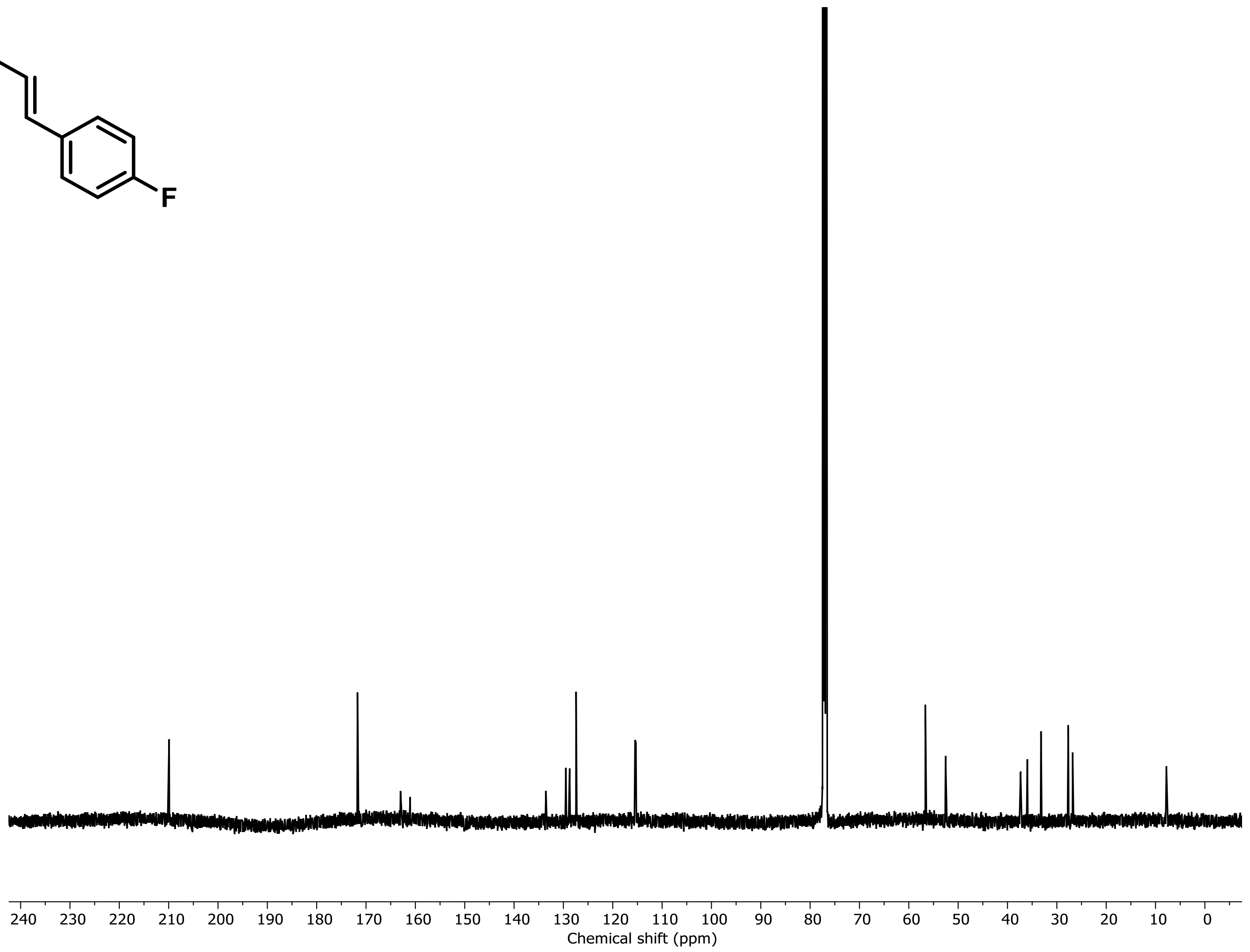

^{19}F NMR (471 MHz, $CDCl_3$)

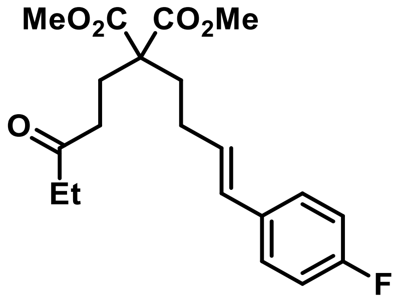

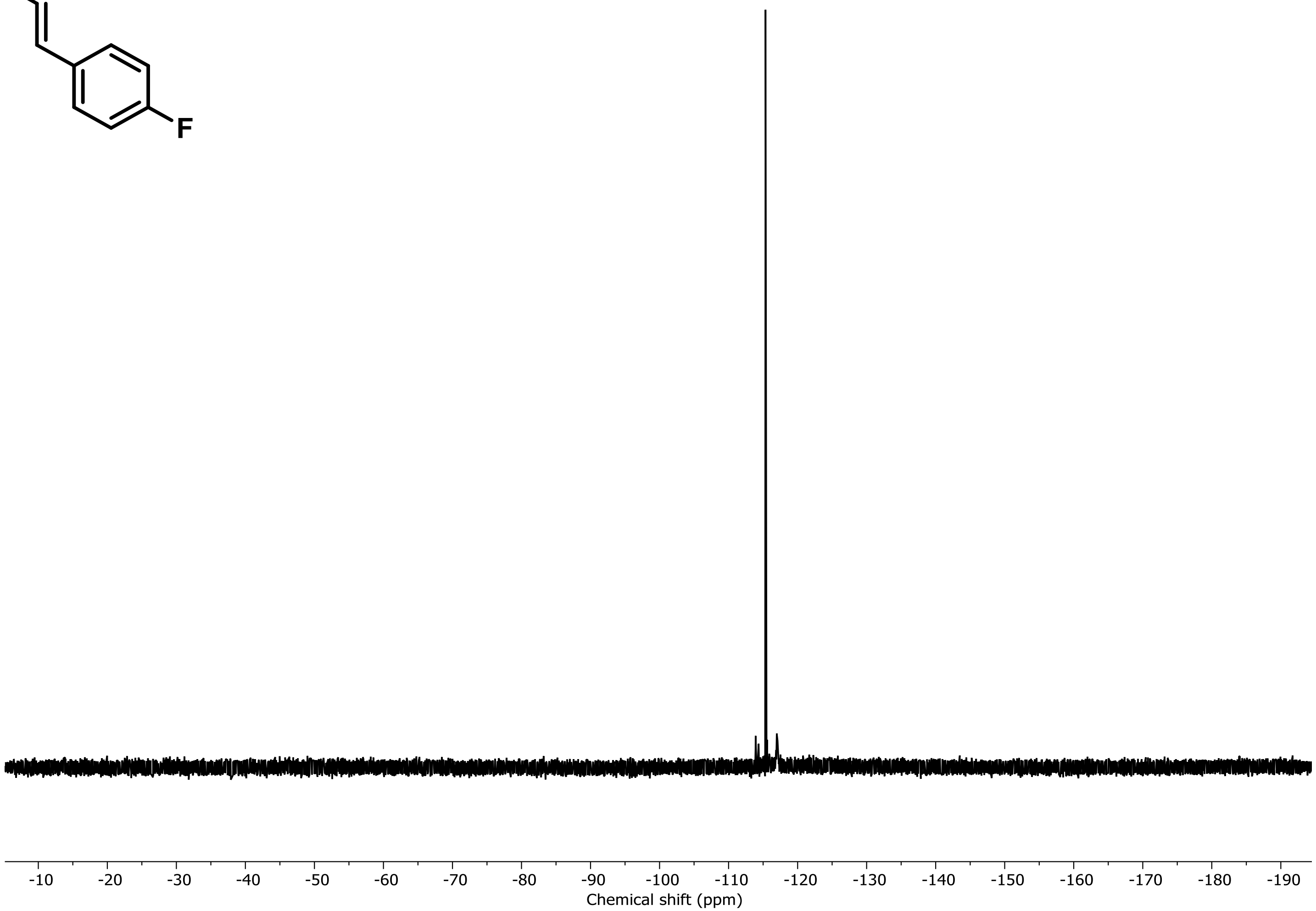

Dimethyl (*E*)–2–(4–(4–bromophenyl)but–3–en–1–yl)–2–(3–oxopentyl)malonate (S29)

^{1}H NMR (500 MHz, $CDCl_3$)

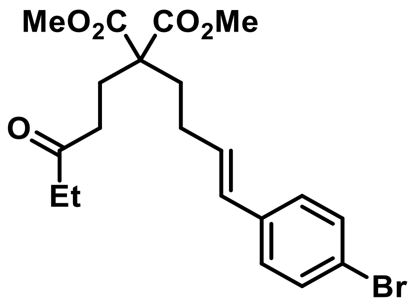

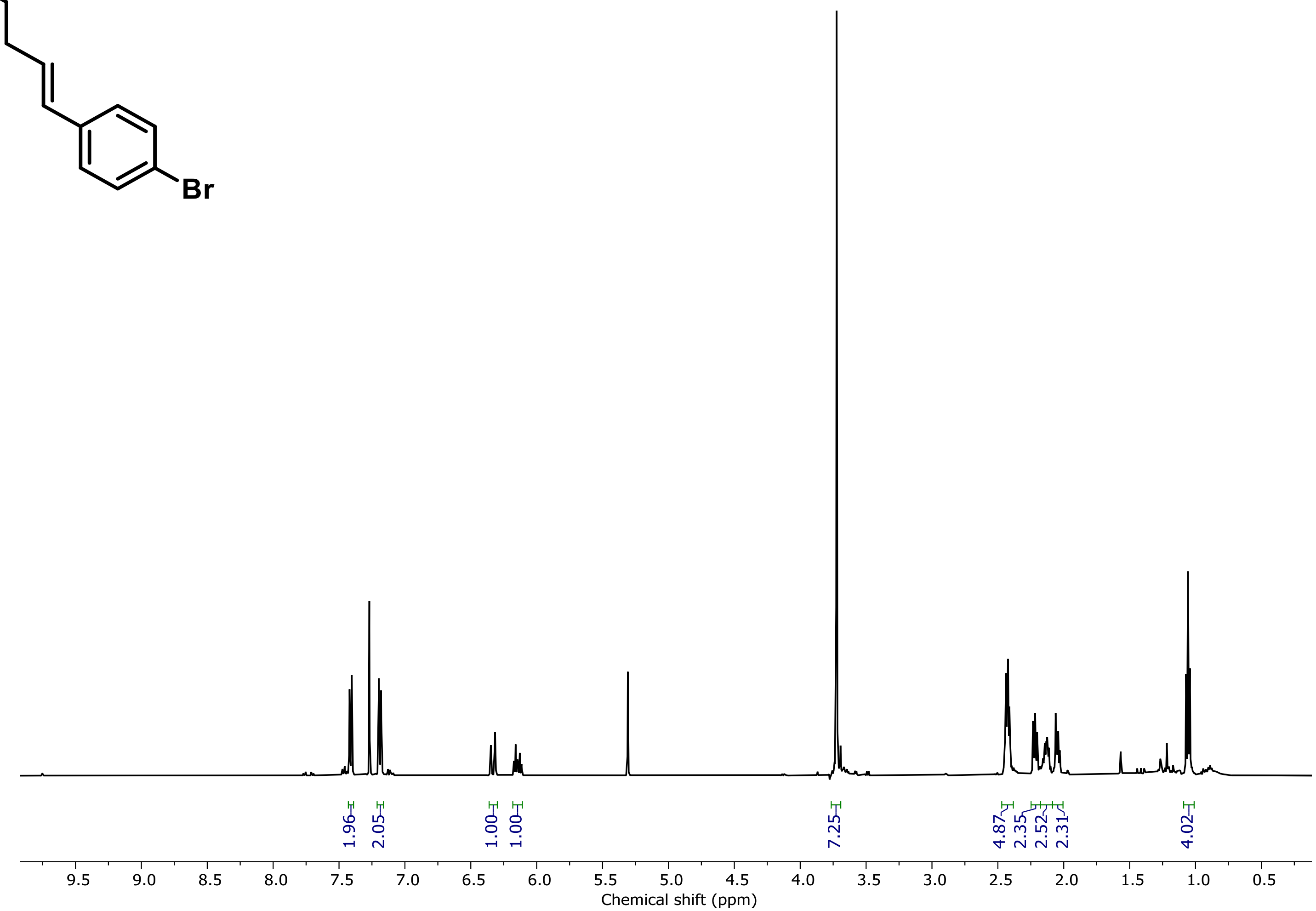

^{13}C NMR (126 MHz, $CDCl_3$)

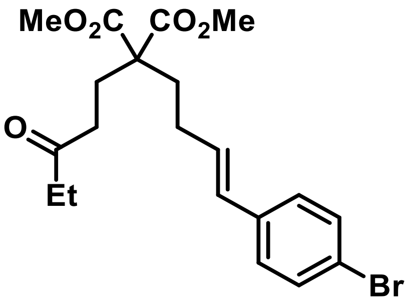

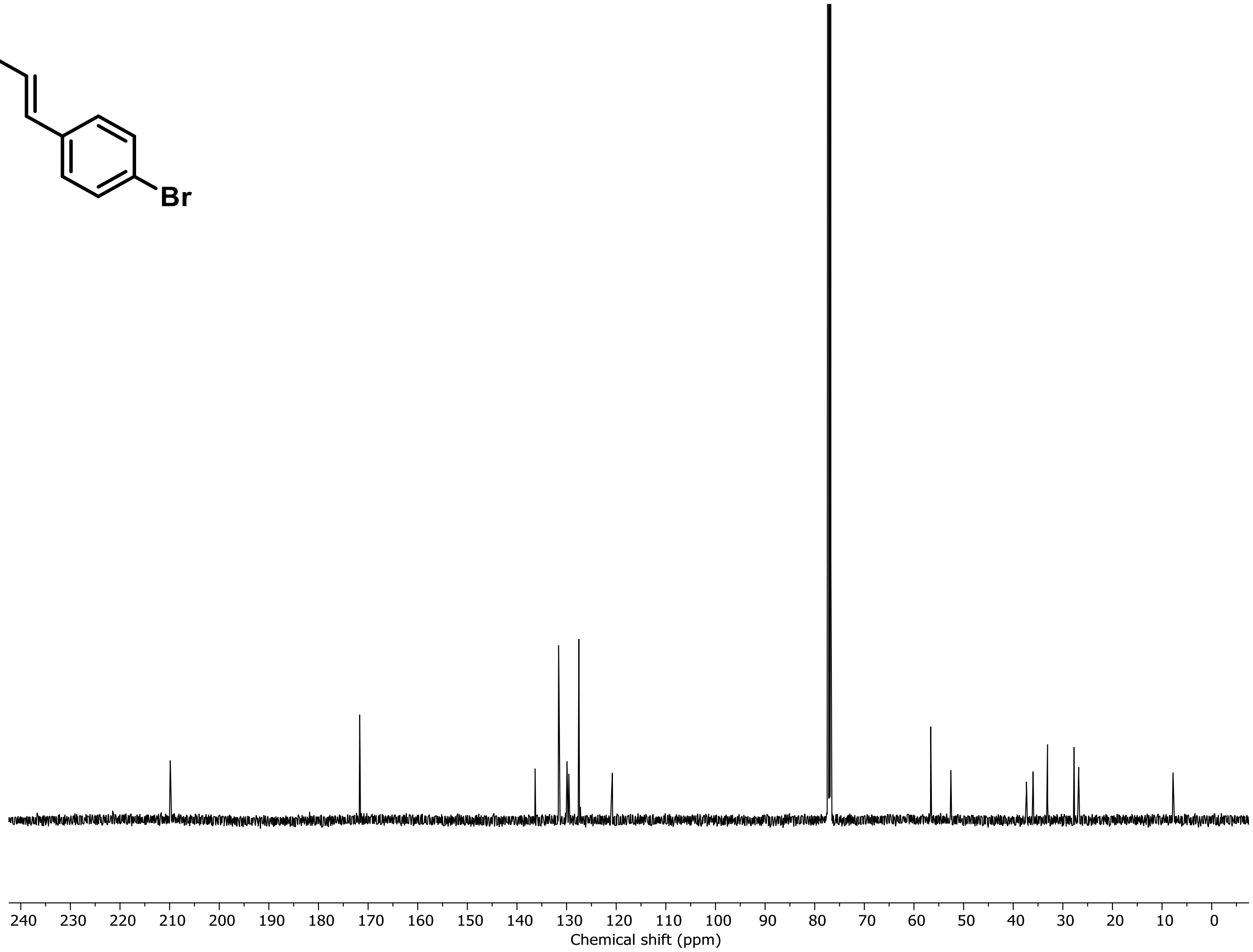

Dimethyl (*E*)–2–(3–oxopentyl)–2–(4–(4–(trifluoromethyl)phenyl)but–3–en–1–yl)malonate (S30)

^{1}H NMR (400 MHz, $CDCl_3$)

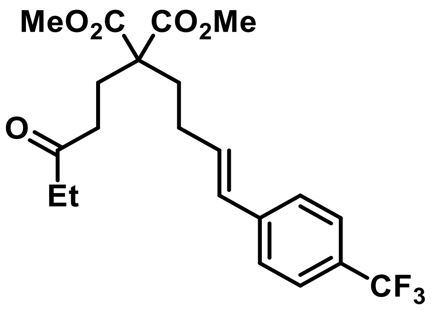

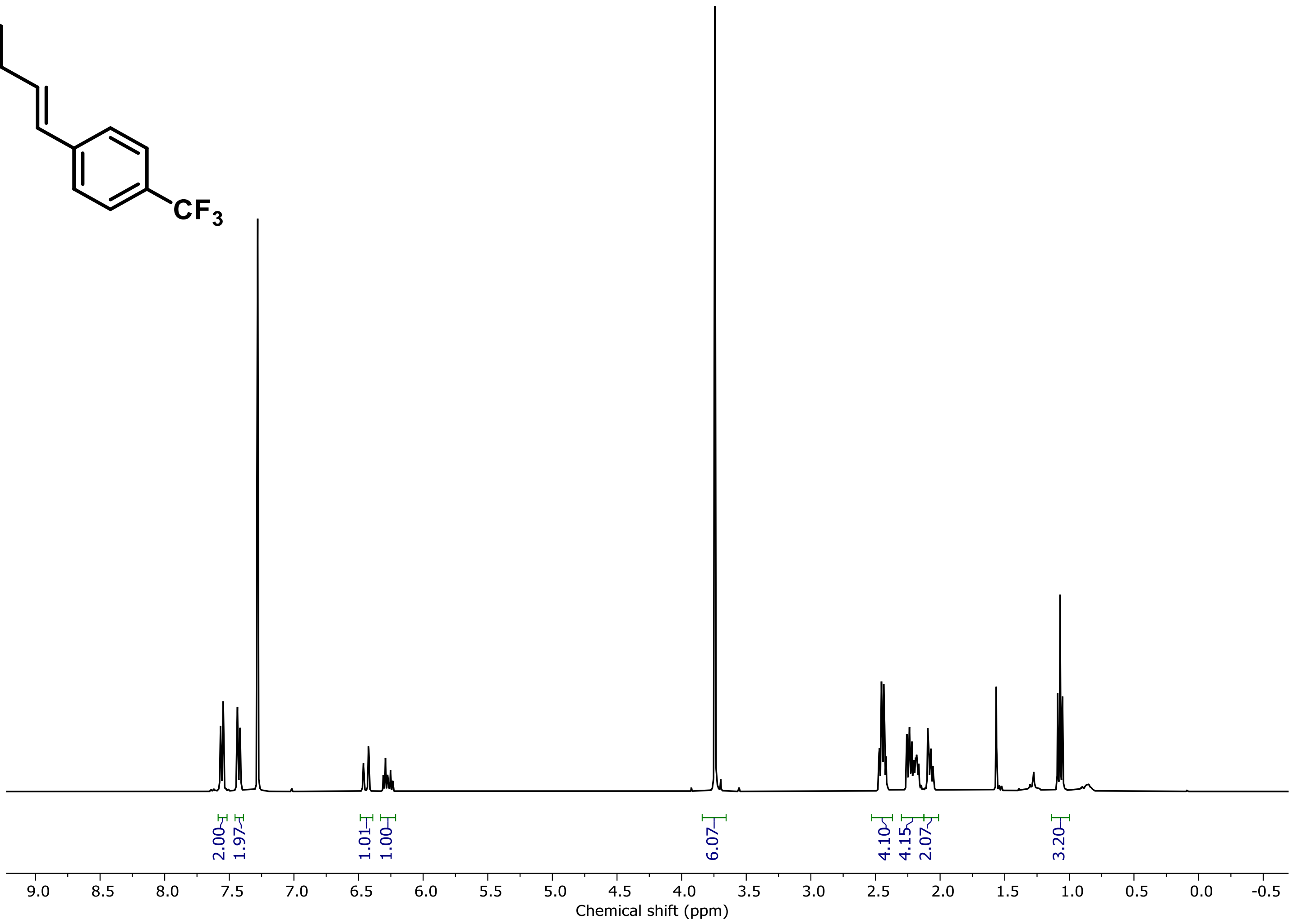

^{13}C NMR (101 MHz, $CDCl_3$)

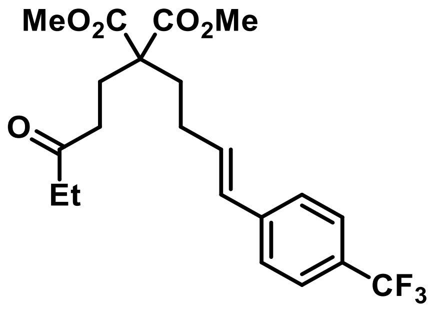

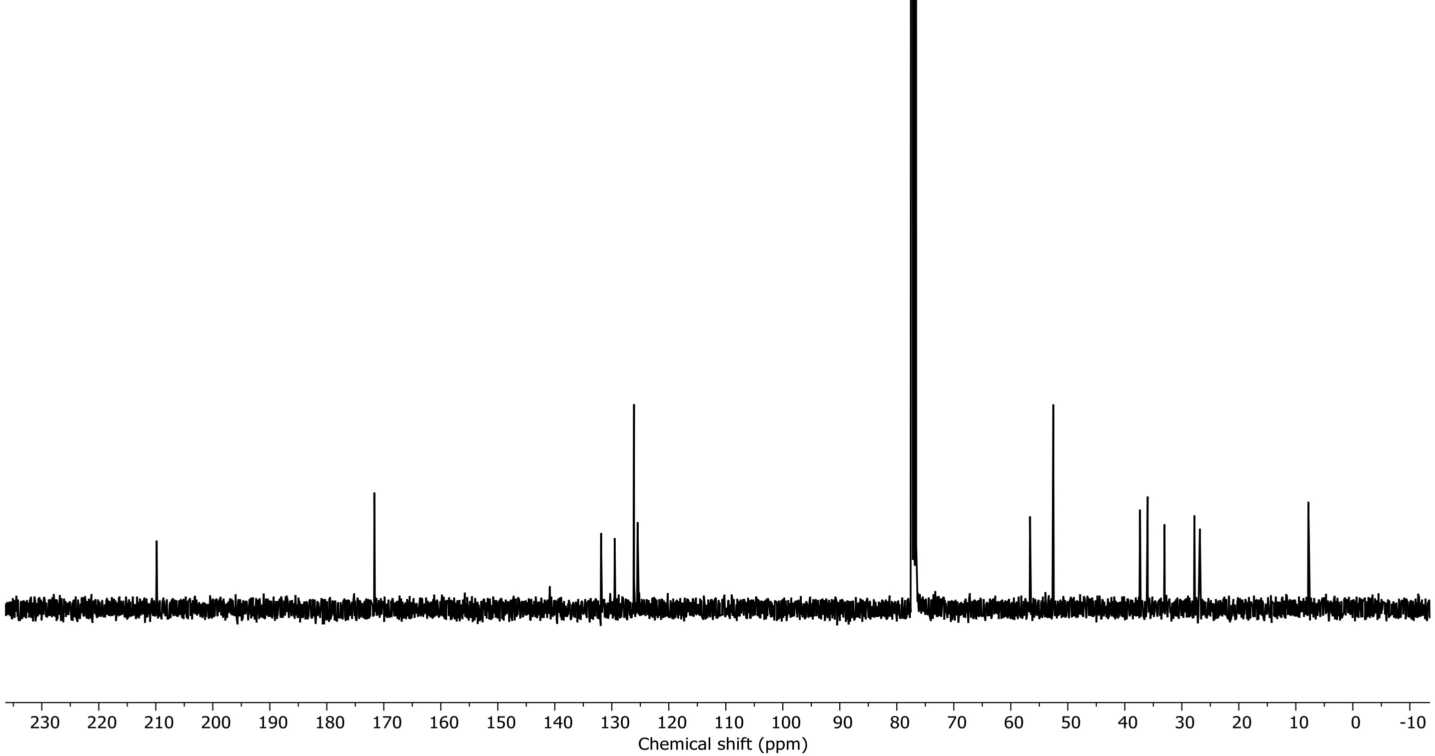

^{19}F NMR (471 MHz, $CDCl_3$)

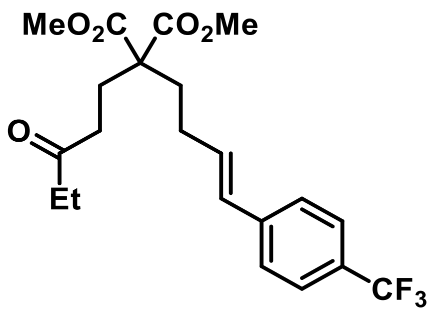

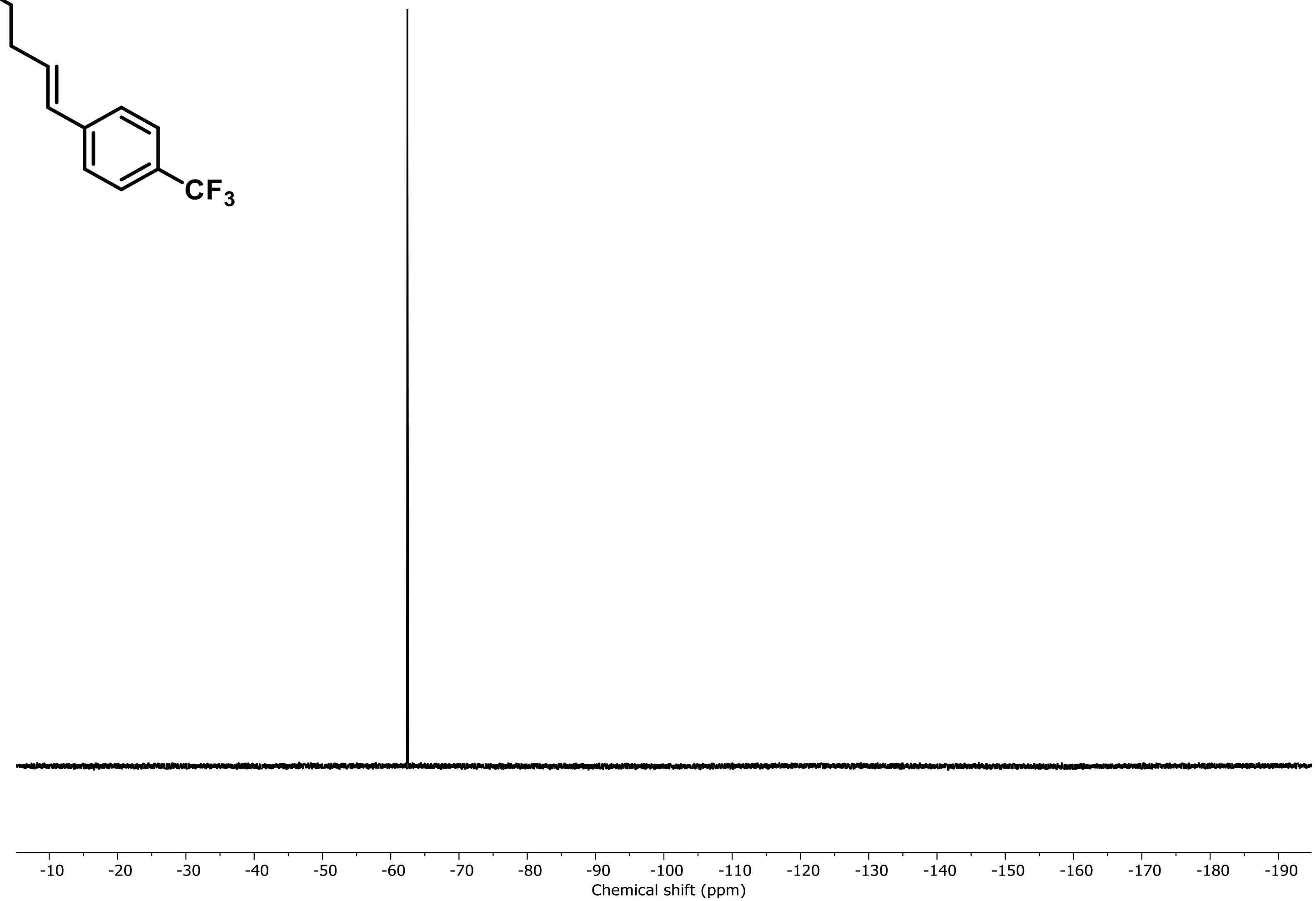

Dimethyl (*E*)–2–(4–(4–methoxyphenyl)but–3–en–1–yl)–2–(3–oxopentyl)malonate (S31)

^{1}H NMR (400 MHz, $CDCl_3$)

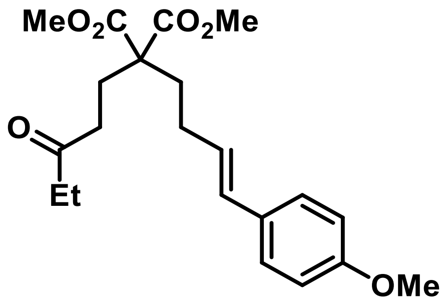

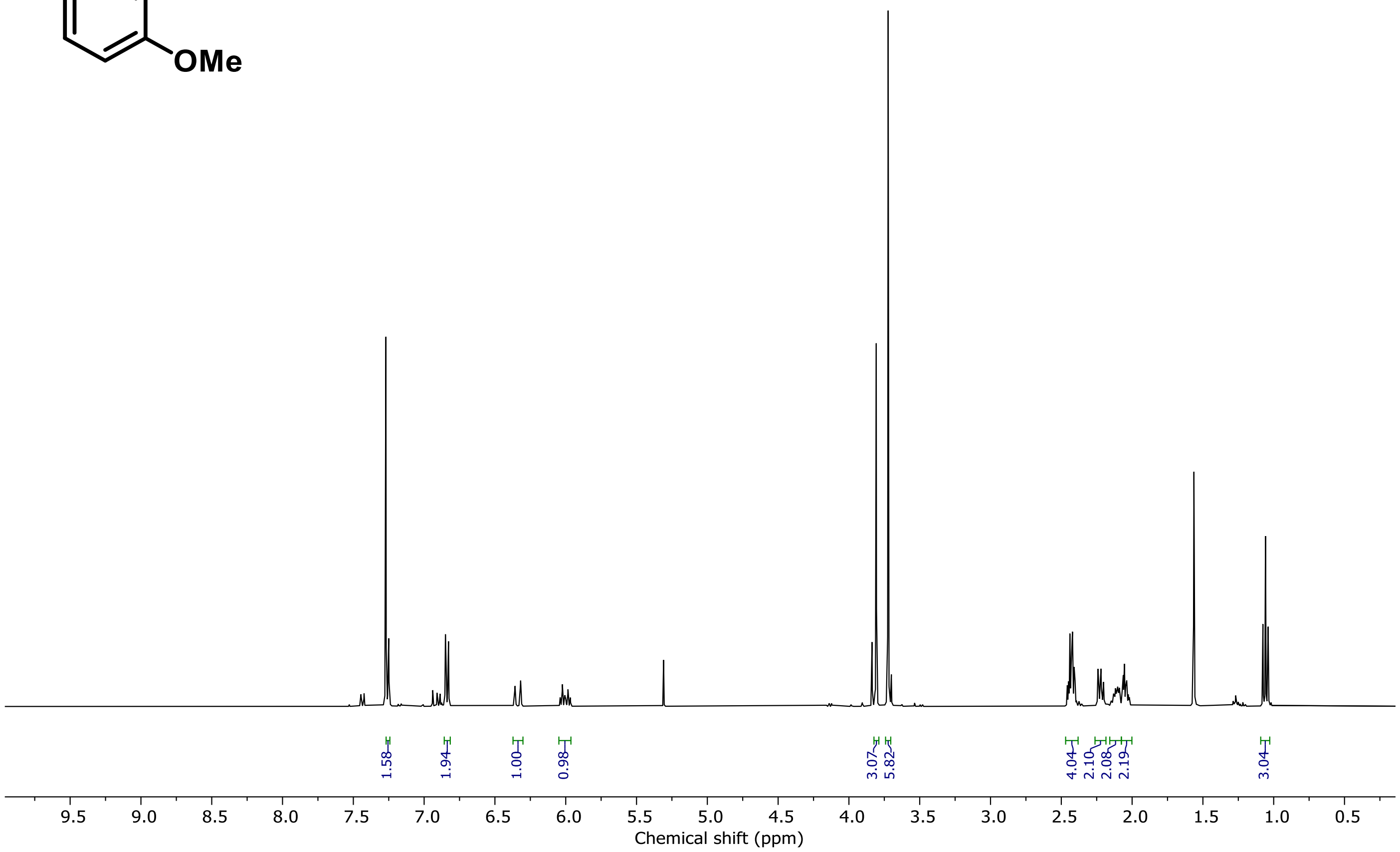

^{13}C NMR (101 MHz, $CDCl_3$)

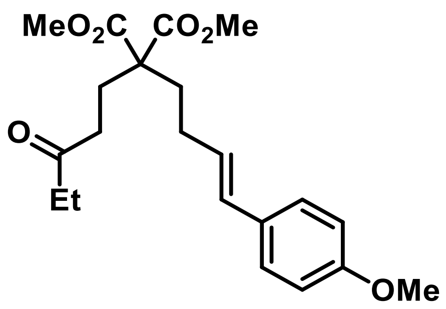

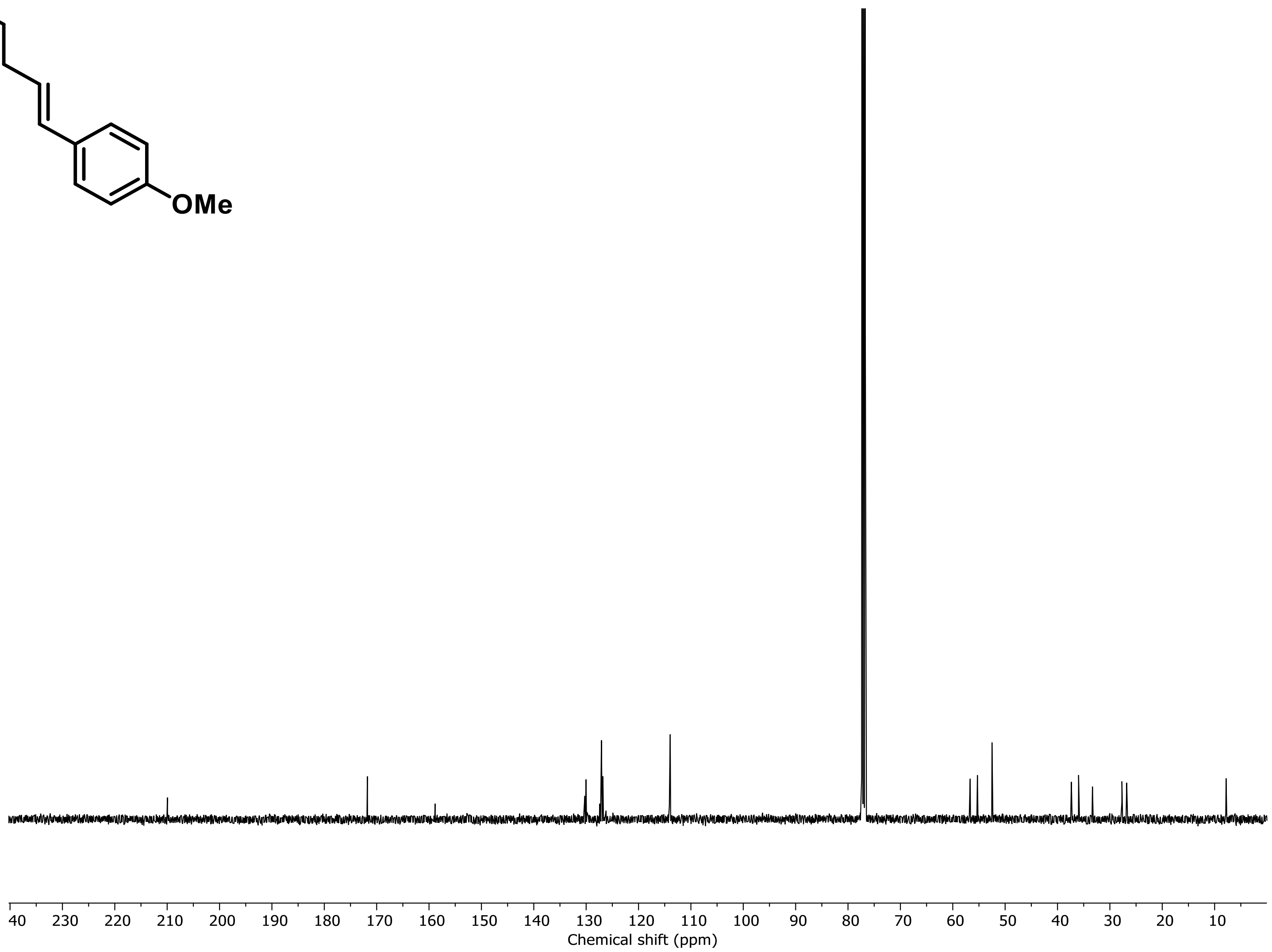

***Rac*–methyl (3*R*,6*S*)–6–methyl–2–oxo–3–((*E*)–4–phenylbut–3–en–1–yl)tetrahydro–2*H*–pyran–3–carboxylate (1a)**

^{1}H NMR (500 MHz, $CDCl_3$)

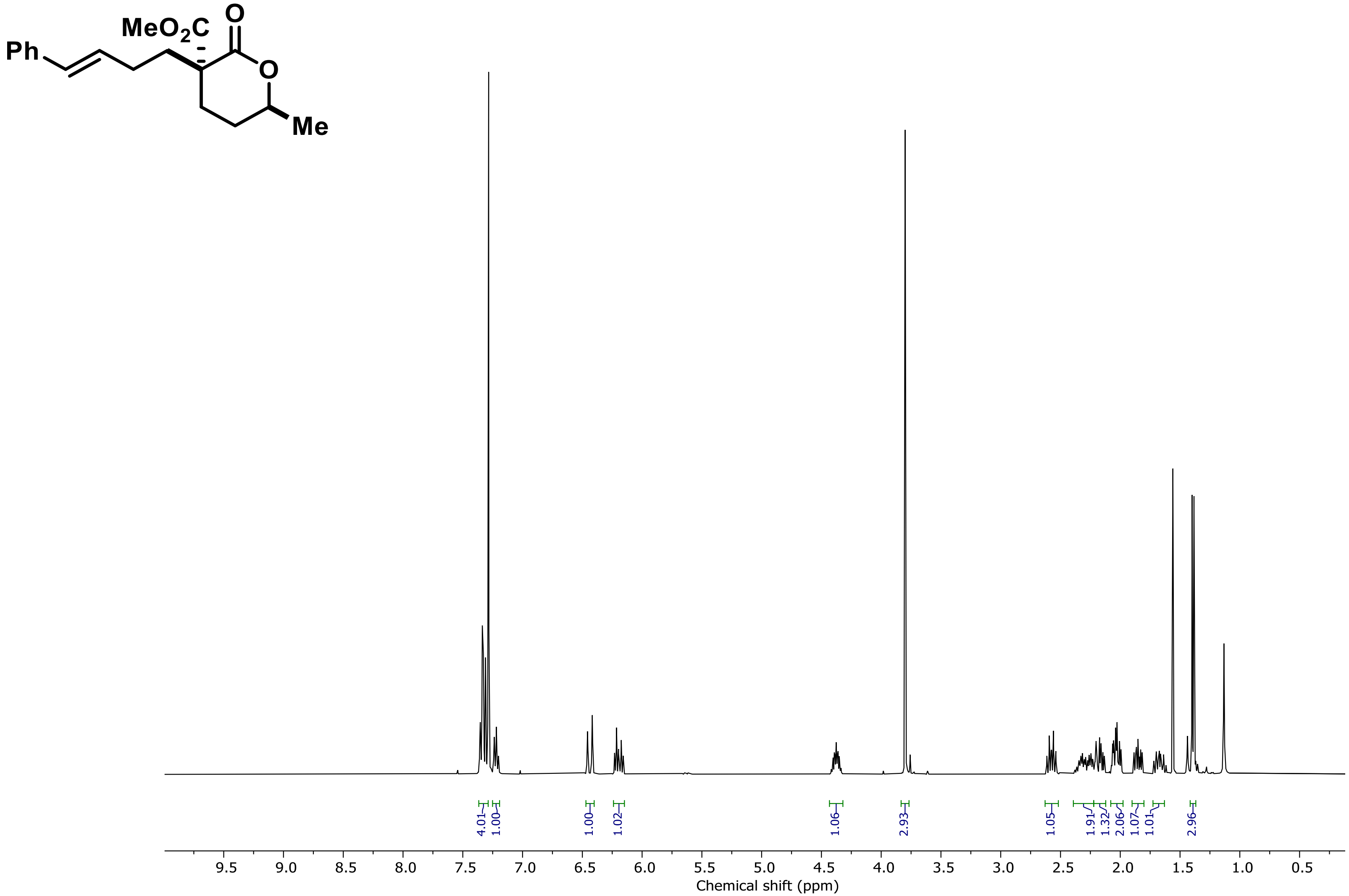

^{13}C NMR (126 MHz, $CDCl_3$)

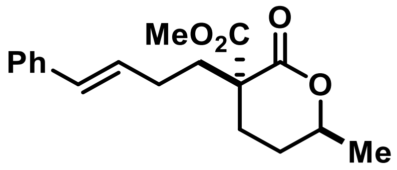

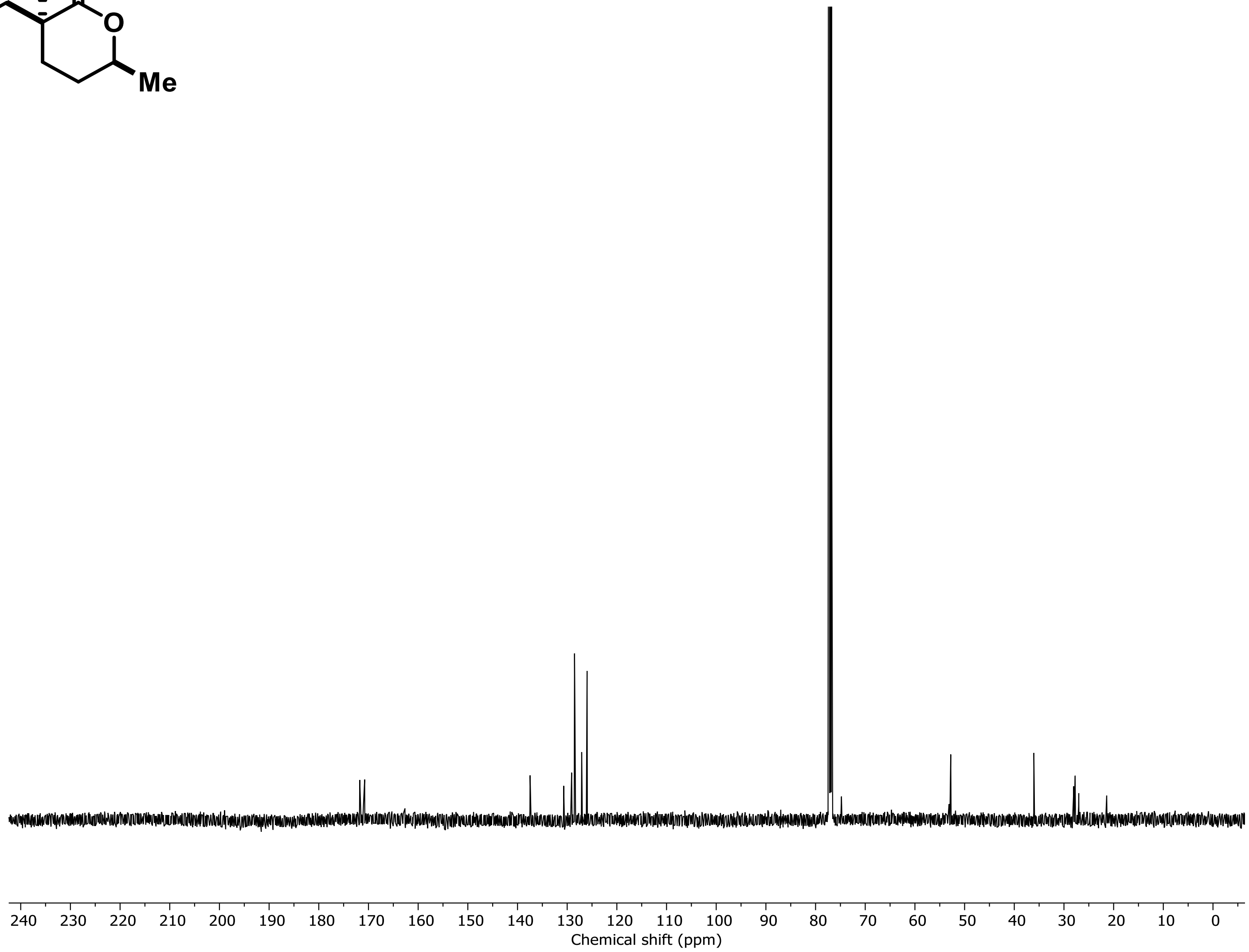

***Rac*–methyl (3*R*,6*S*)–6–ethyl–2–oxo–3–((*E*)–4–phenylbut–3–en–1–yl)tetrahydro–2*H*–pyran–3–carboxylate (1b)**

^{1}H NMR (400 MHz, $CDCl_3$) 7.3:1 d.r.

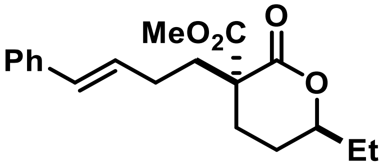

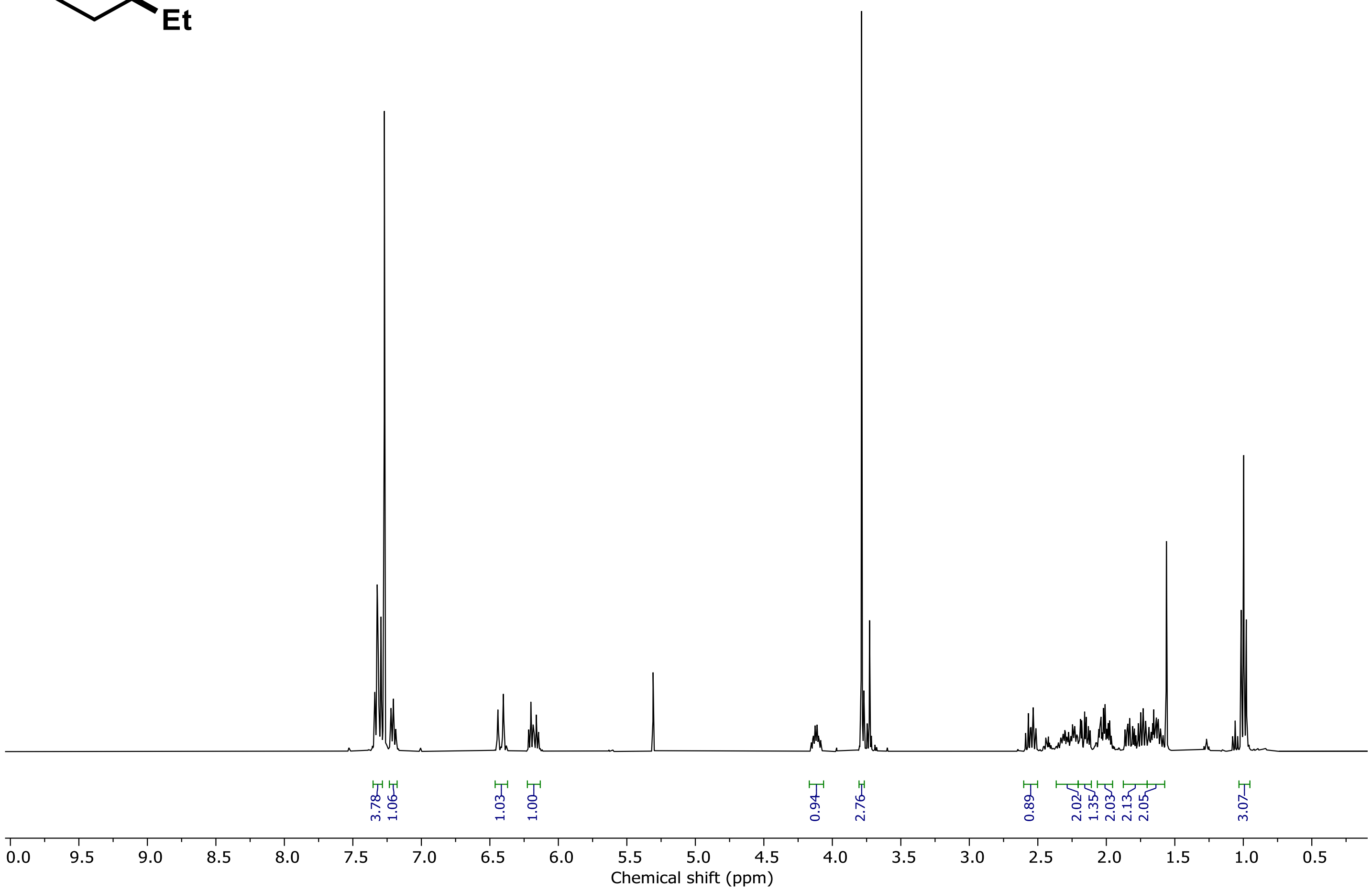

^{13}C NMR (101 MHz, $CDCl_3$) 7.3:1 d.r.

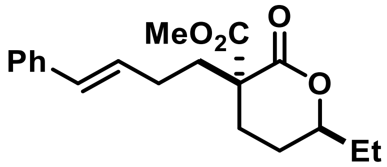

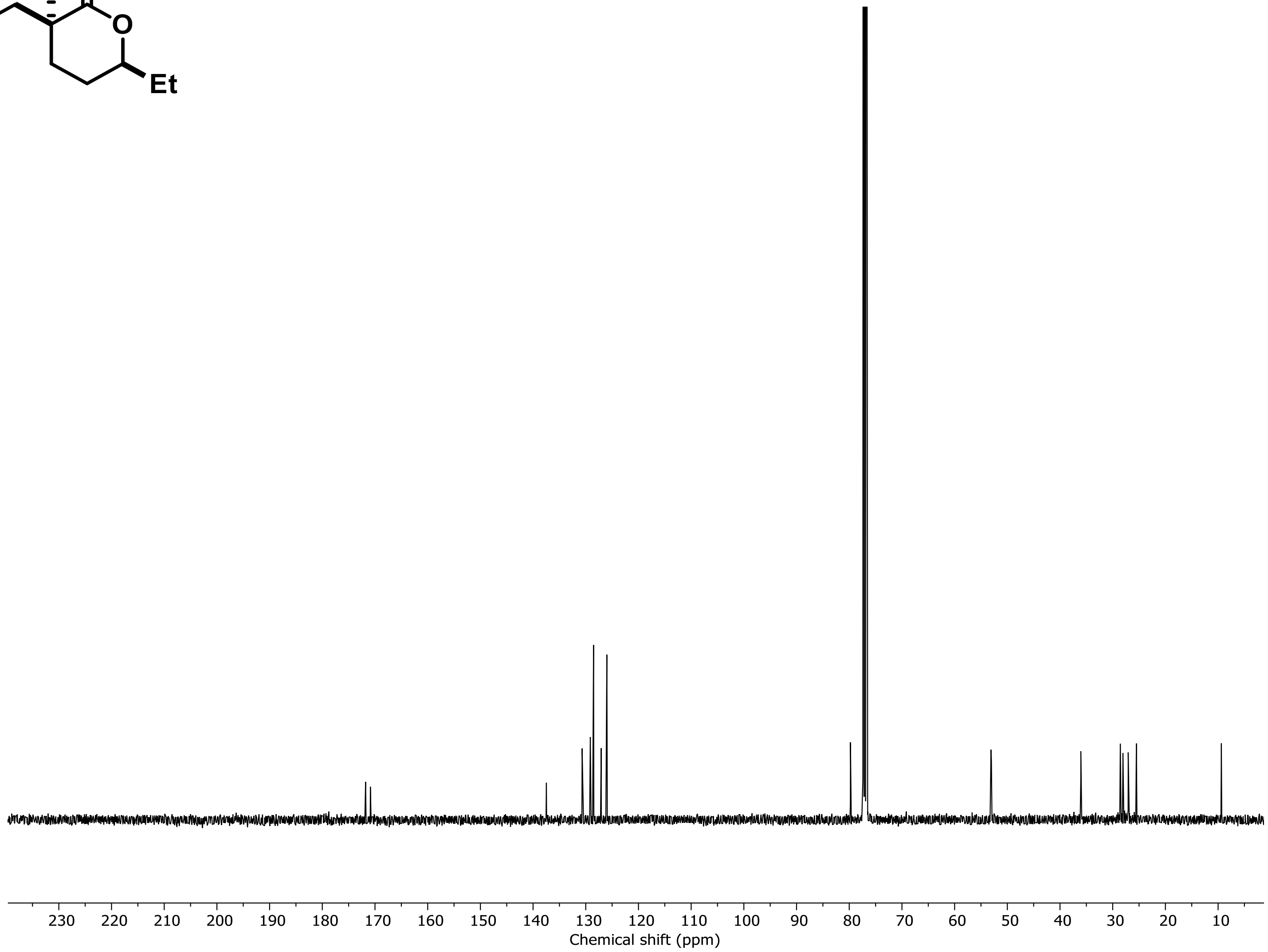

***Rac*–methyl (3*R*,6*S*)–6–butyl–2–oxo–3–((*E*)–4–phenylbut–3–en–1–yl)tetrahydro–2*H*–pyran–3–carboxylate (1c)**

^{1}H NMR (500 MHz, $CDCl_3$) 4.5:1 d.r.

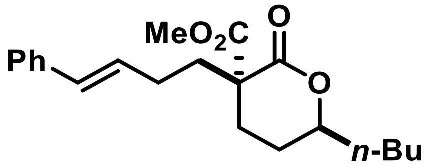

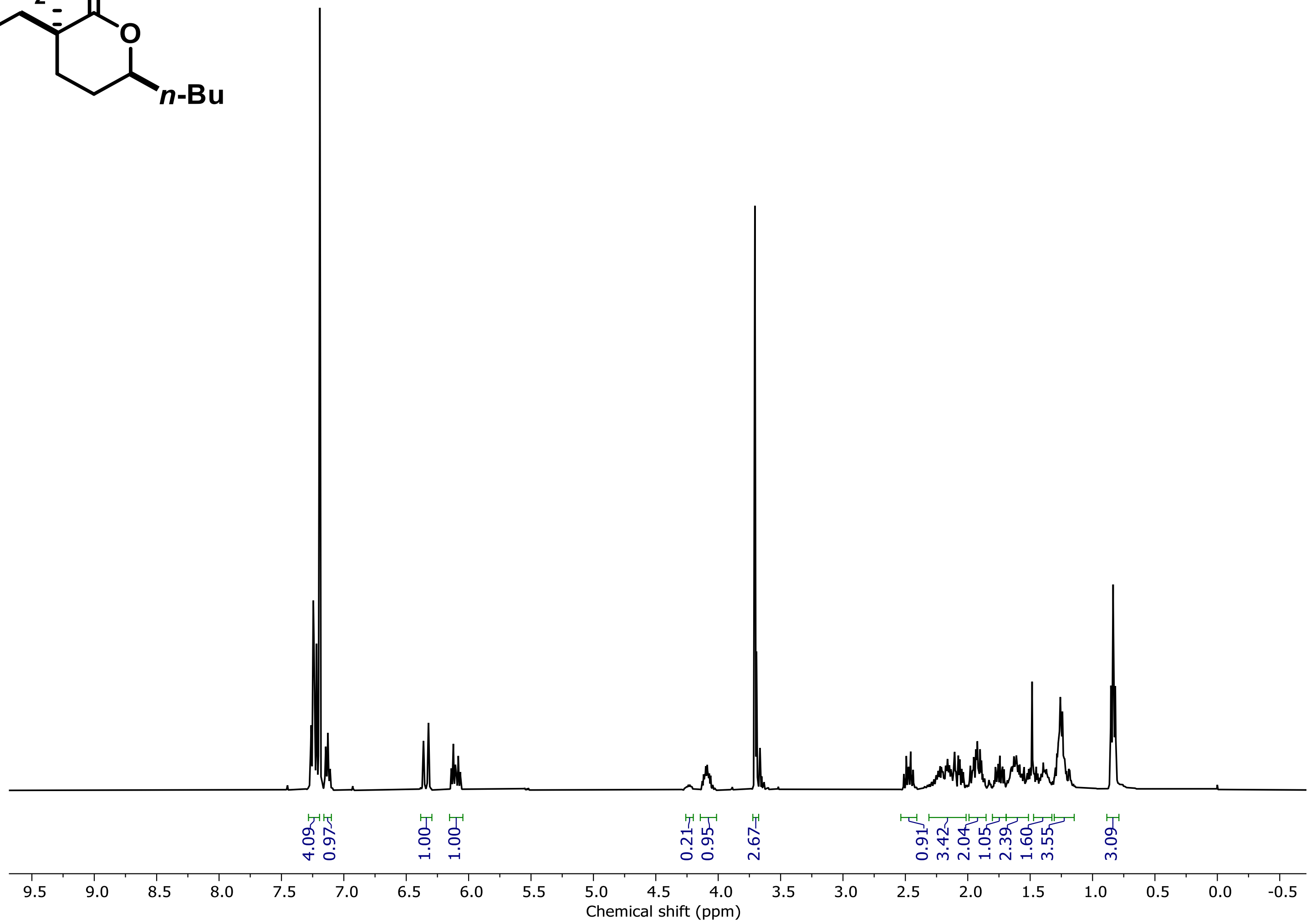

^{13}C NMR (126 MHz, $CDCl_3$) 4.5:1 d.r.

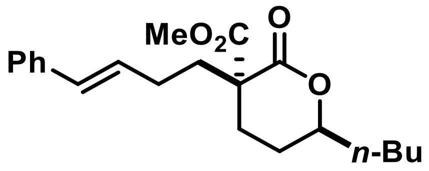

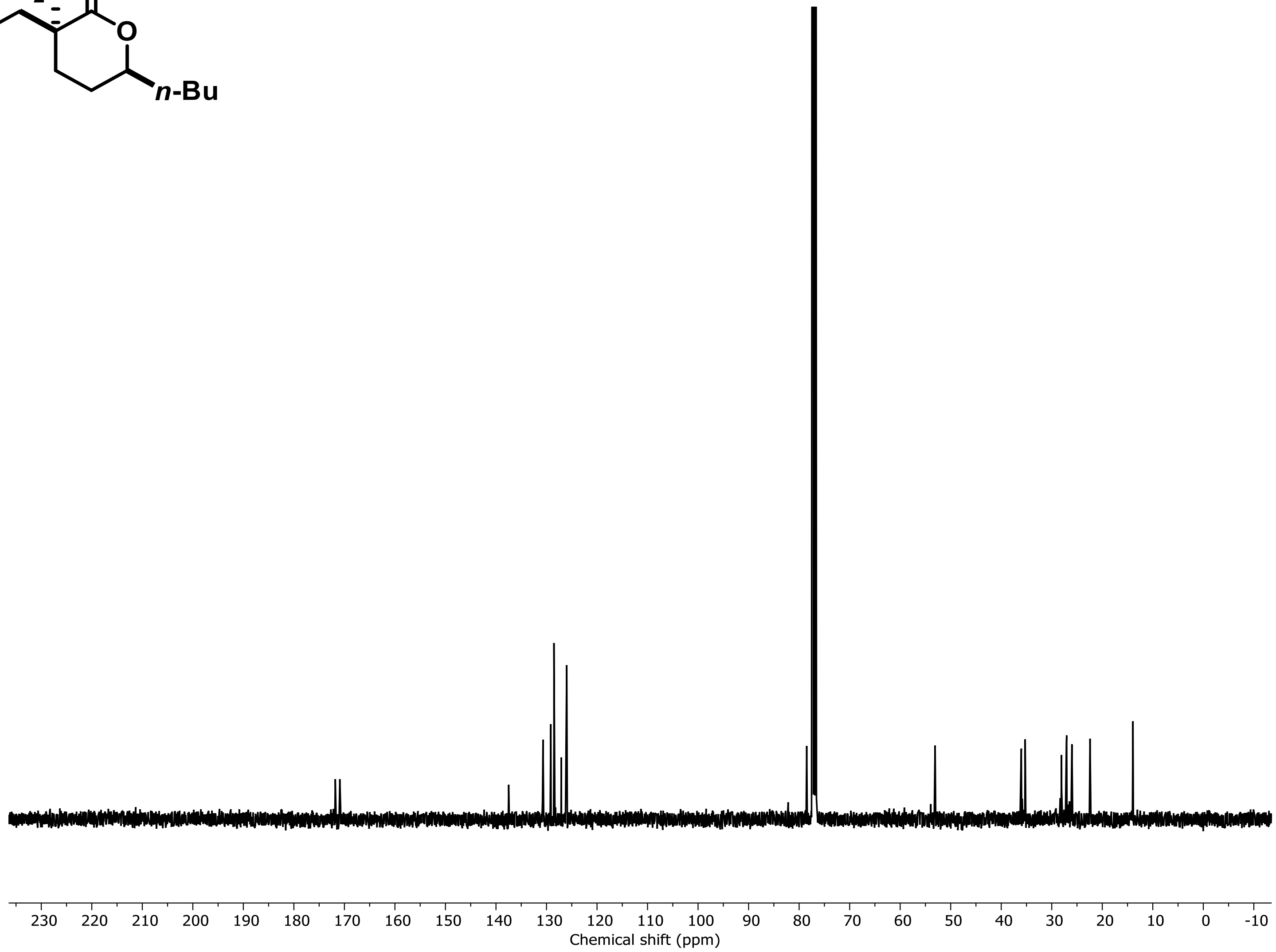

***Rac*–methyl (3*R*,6*R*)–6–benzyl–2–oxo–3–((*E*)–4–phenylbut–3–en–1–yl)tetrahydro–2*H*–pyran–3–carboxylate (1d)**

^{1}H NMR (400 MHz, $CDCl_3$) 7.3:1 d.r.

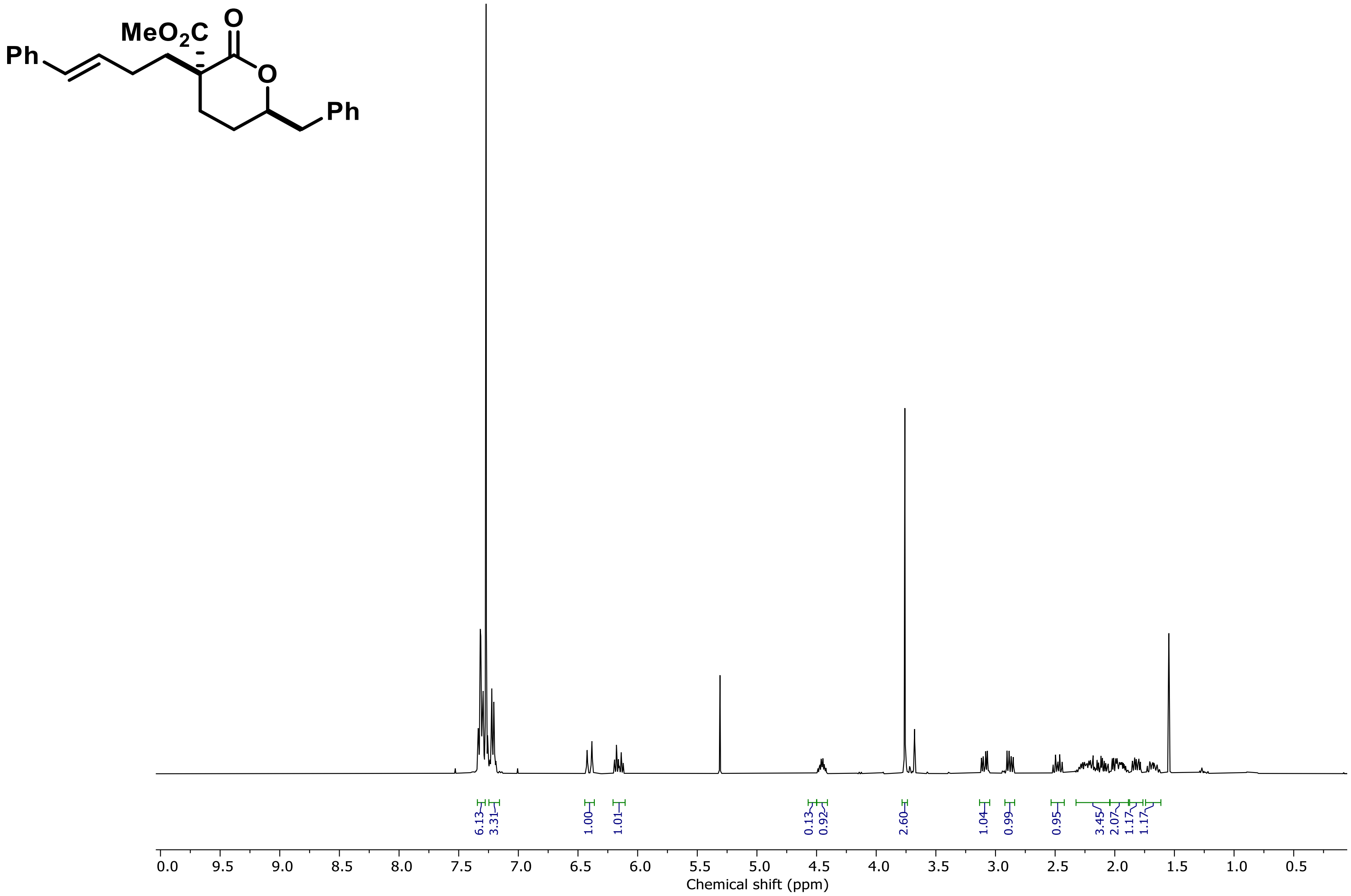

^{13}C NMR (101 MHz, $CDCl_3$) 7.3:1 d.r.

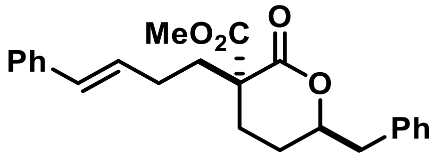

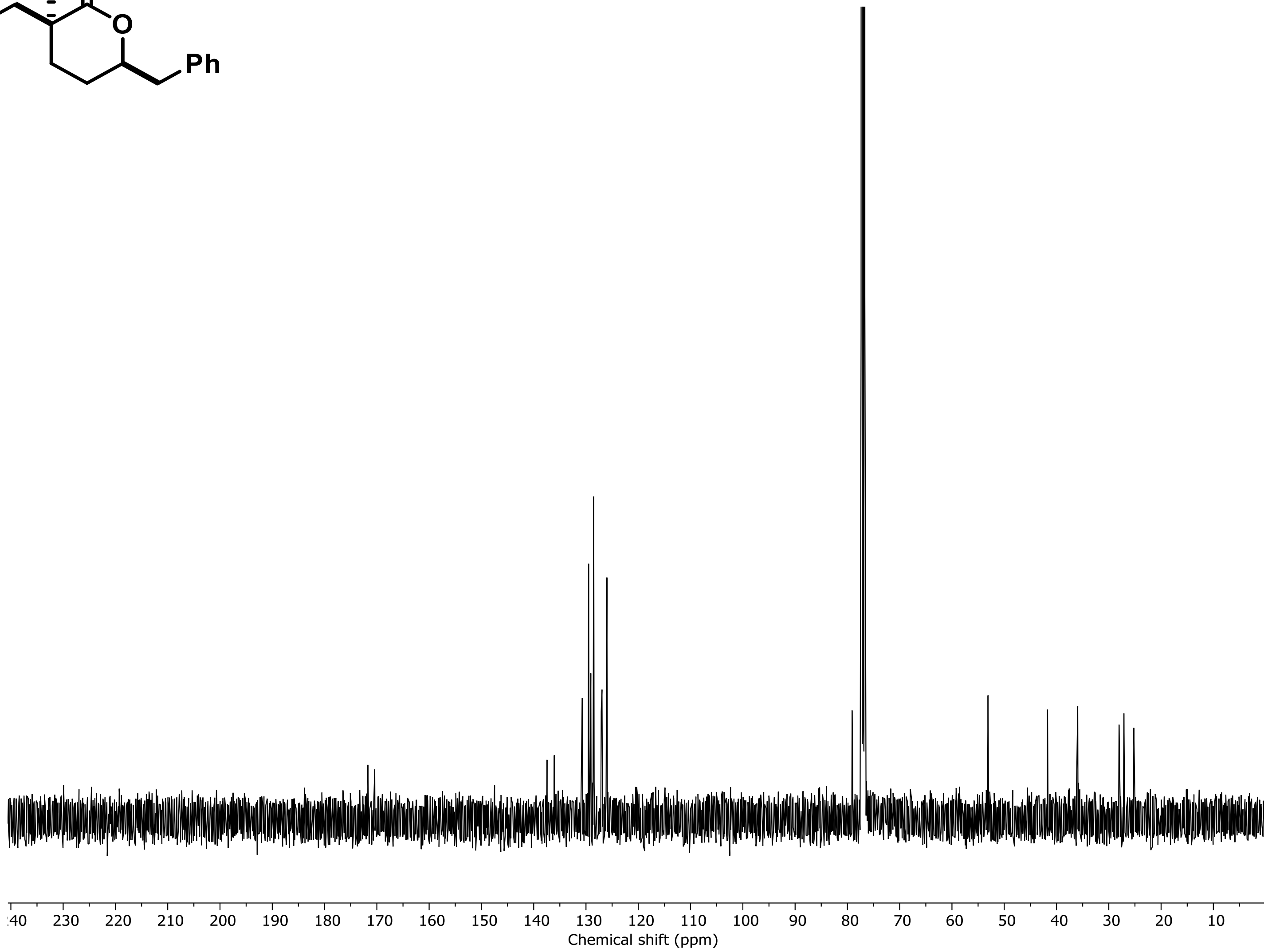

***Rac*–methyl (3*R*,6*R*)–6–neopentyl–2–oxo–3–((*E*)–4–phenylbut–3–en–1–yl)tetrahydro–2*H*–pyran–3–carboxylate (1e)**

^{1}H NMR (400 MHz, $CDCl_3$) 8.6:1 d.r.

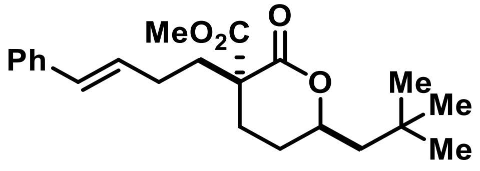

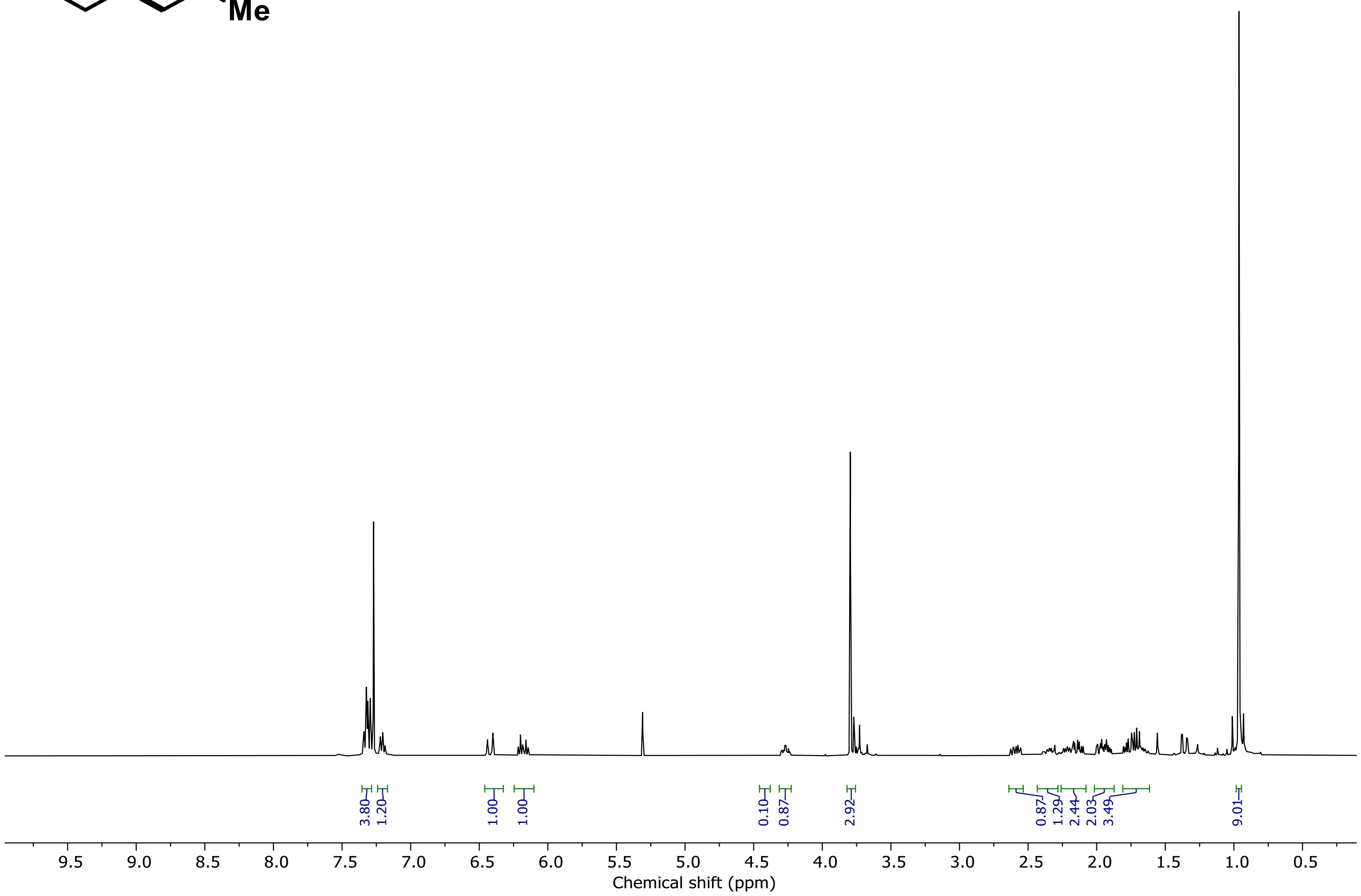

^{13}C NMR (101 MHz, $CDCl_3$) 8.6:1 d.r.

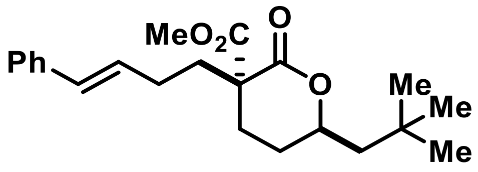

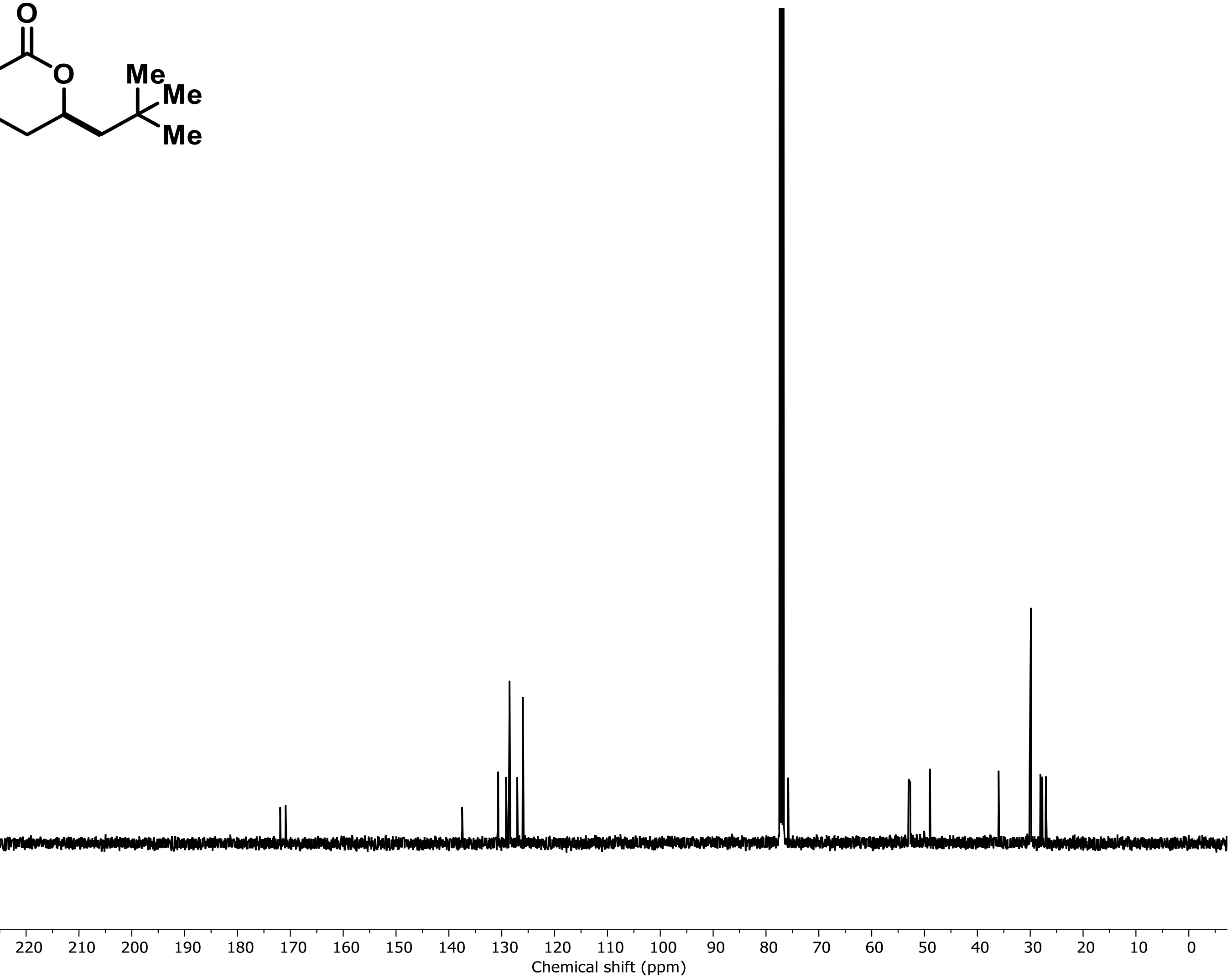

***Rac*–methyl (3*R*,6*R*)–6–isopropyl–2–oxo–3–((*E*)–4–phenylbut–3–en–1–yl)tetrahydro–2*H*–pyran–3–carboxylate (1f)**

^{1}H NMR (400 MHz, $CDCl_3$) 5.3:1 d.r.

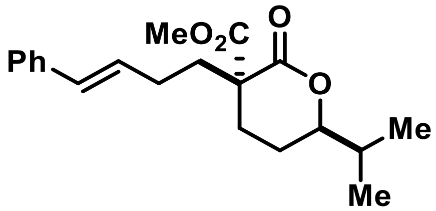

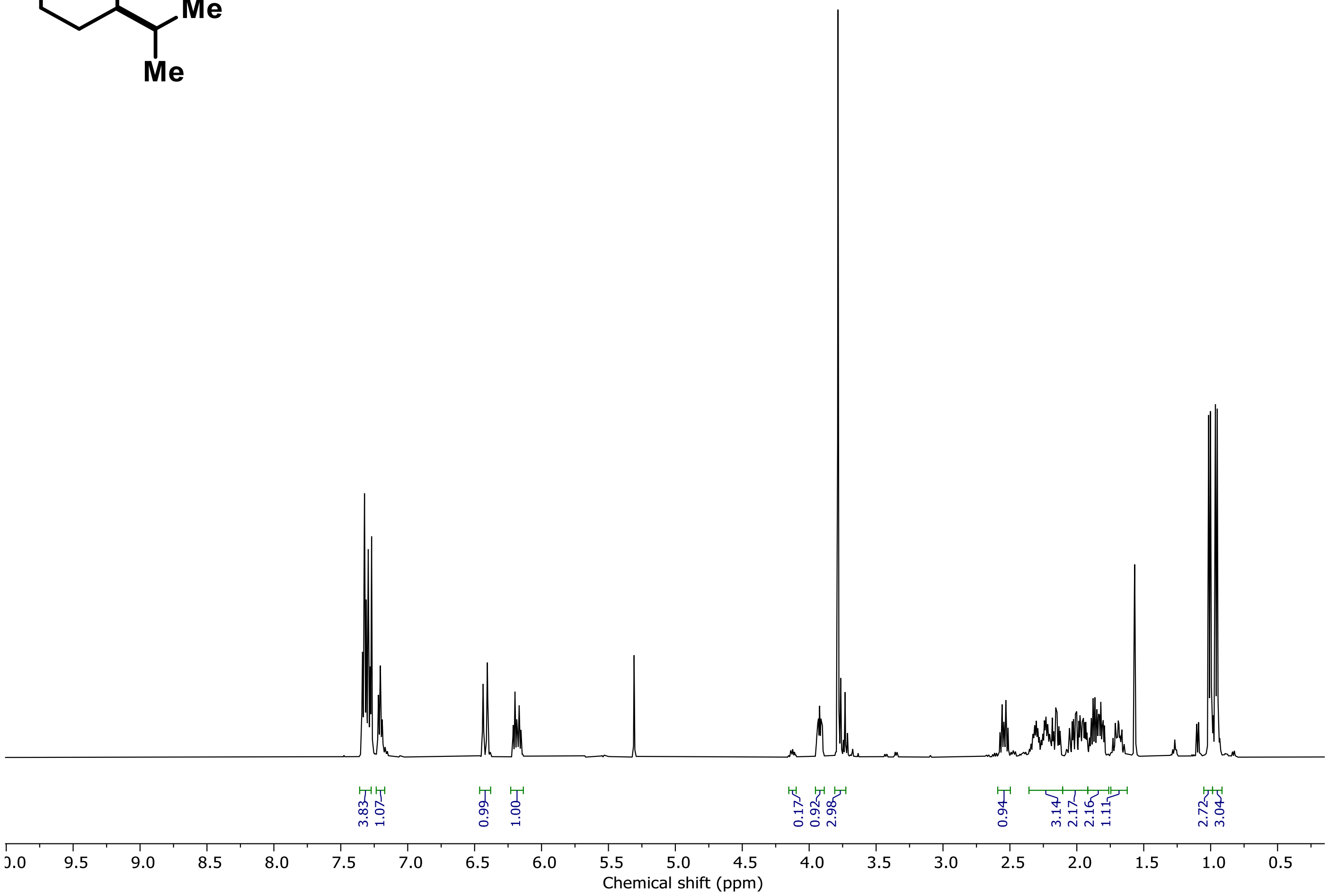

^{13}C NMR (126 MHz, $CDCl_3$) 5.3:1 d.r.

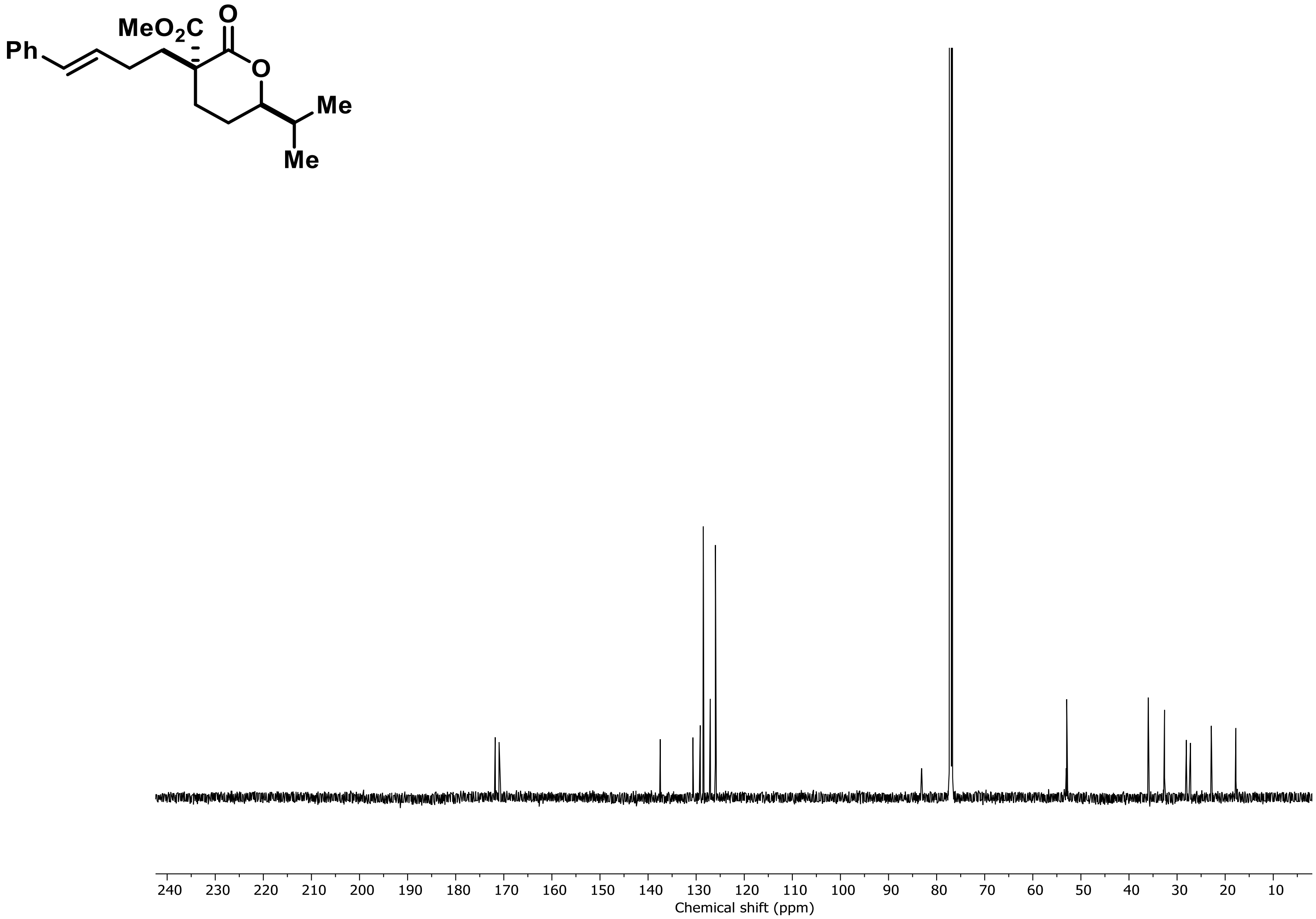

***Rac*–methyl (3*R*,6*R*)–6–benzhydryl–2–oxo–3–((*E*)–4–phenylbut–3–en–1–yl)tetrahydro–2*H*–pyran–3–carboxylate (1g)**

^{1}H NMR (400 MHz, $CDCl_3$) 2.6:1 d.r.

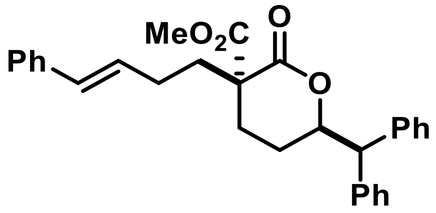

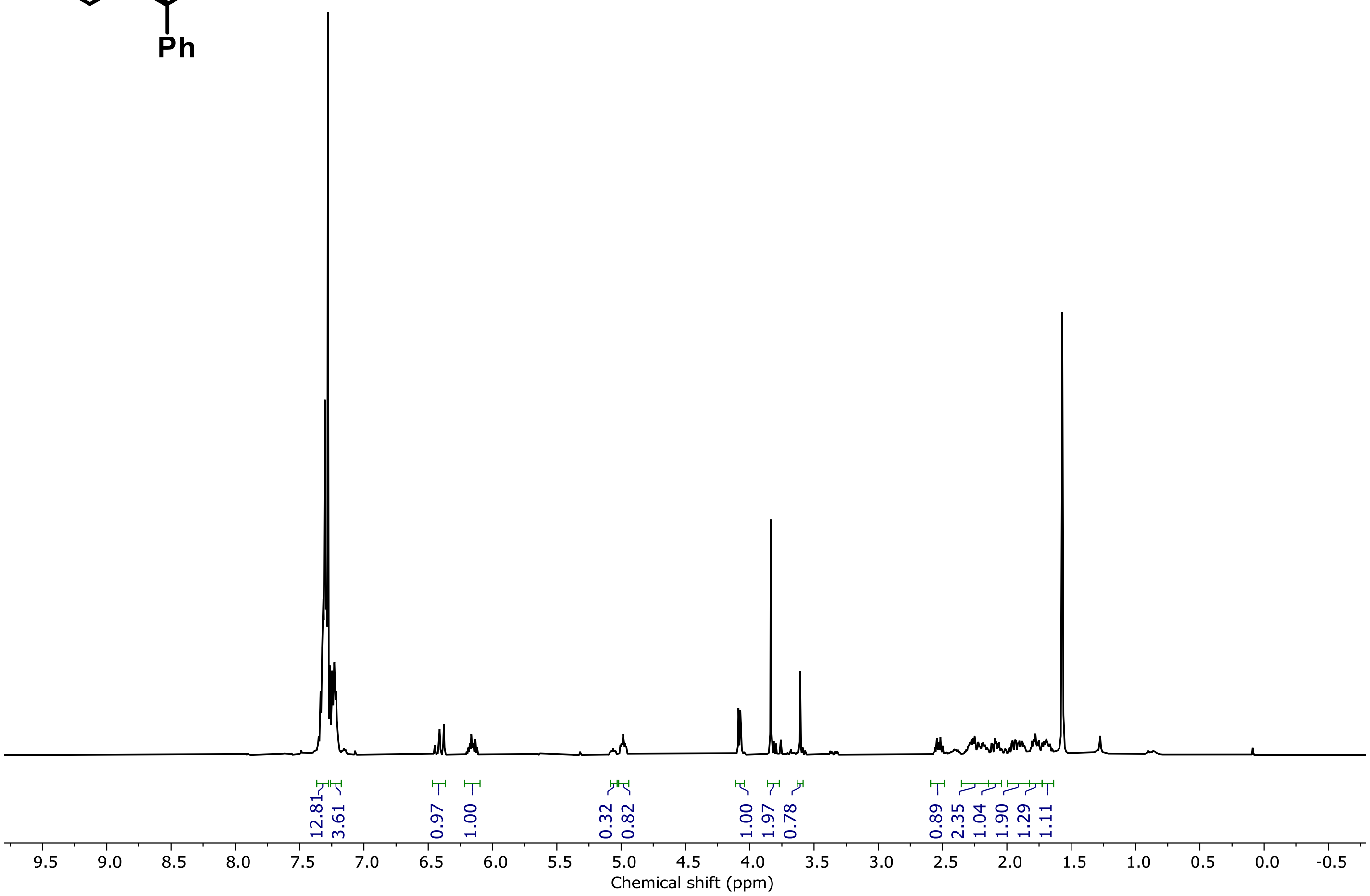

^{13}C NMR (101 MHz, $CDCl_3$) 2.6:1 d.r.

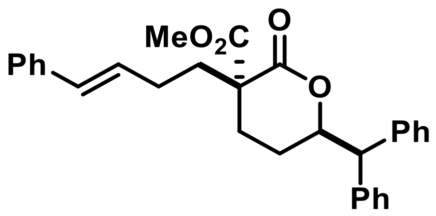

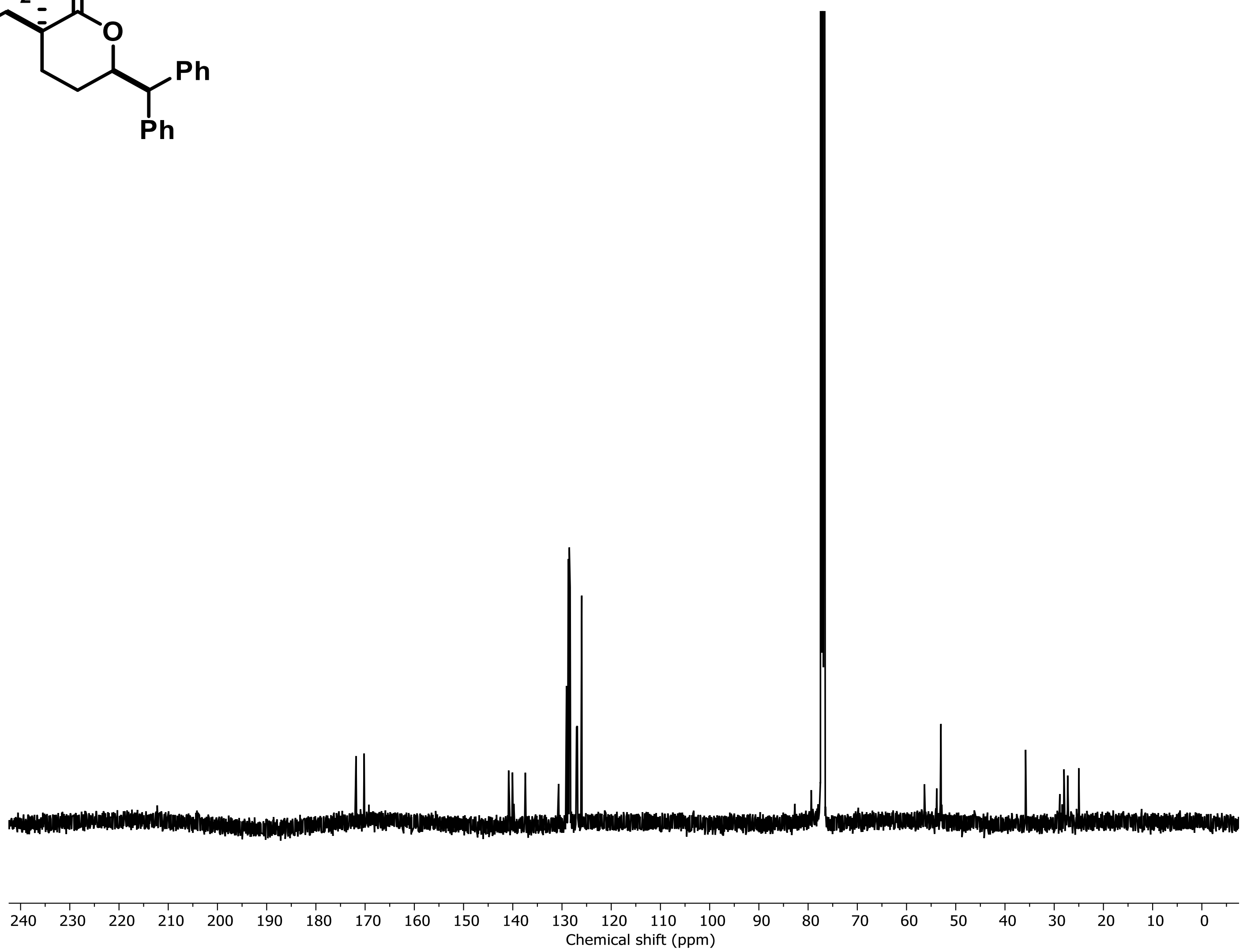

***Rac*–methyl (3*R*,6*R*)–6–cyclohexyl–2–oxo–3–((*E*)–4–phenylbut–3–en–1–yl)tetrahydro–2*H*–pyran–3–carboxylate (1h)**

^{1}H NMR (400 MHz, $CDCl_3$)

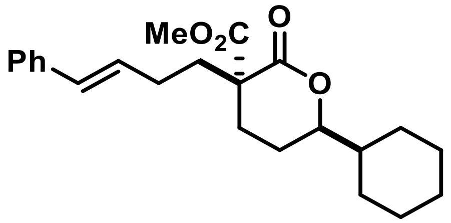

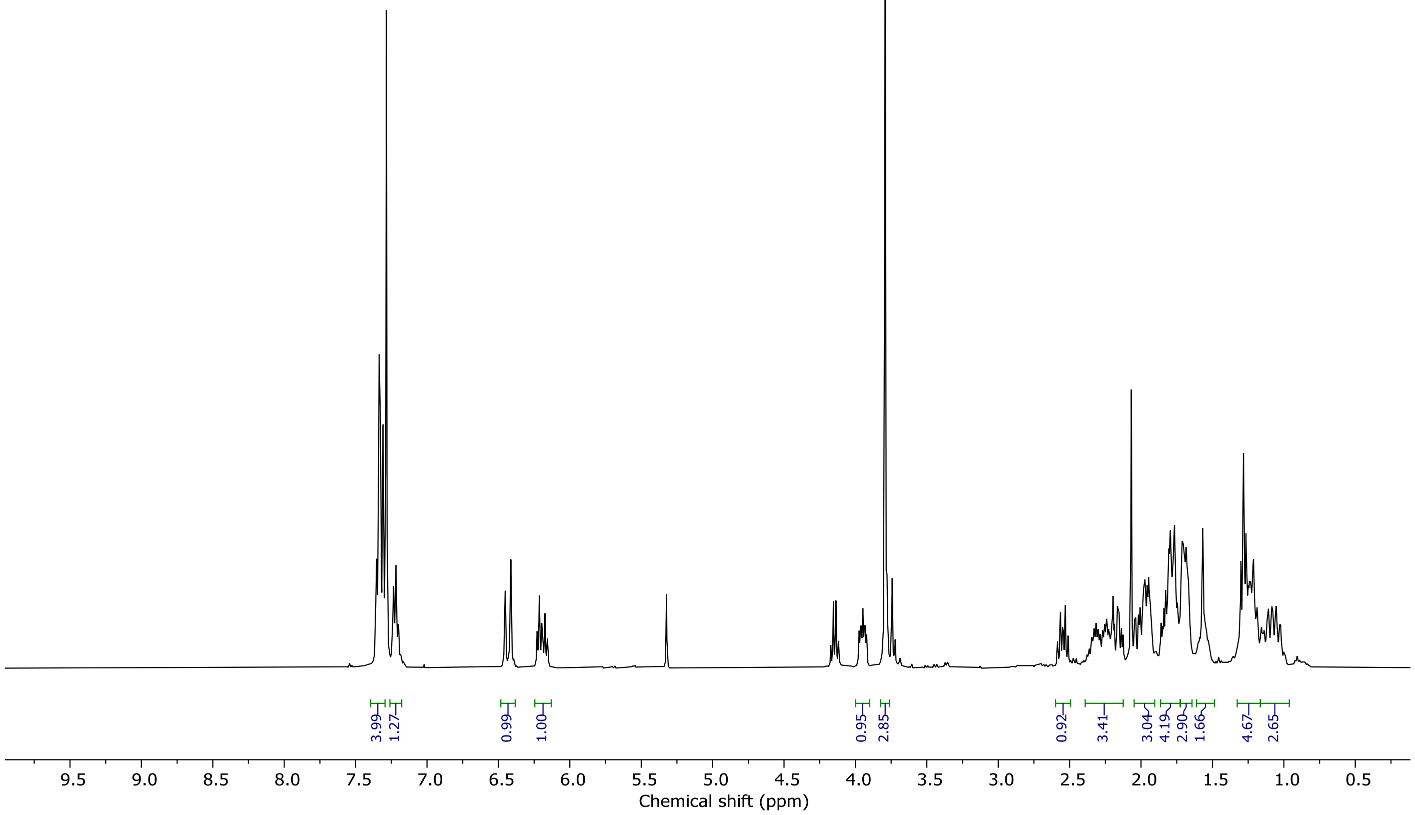

^{13}C NMR (101 MHz, $CDCl_3$)

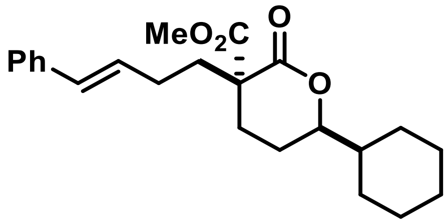

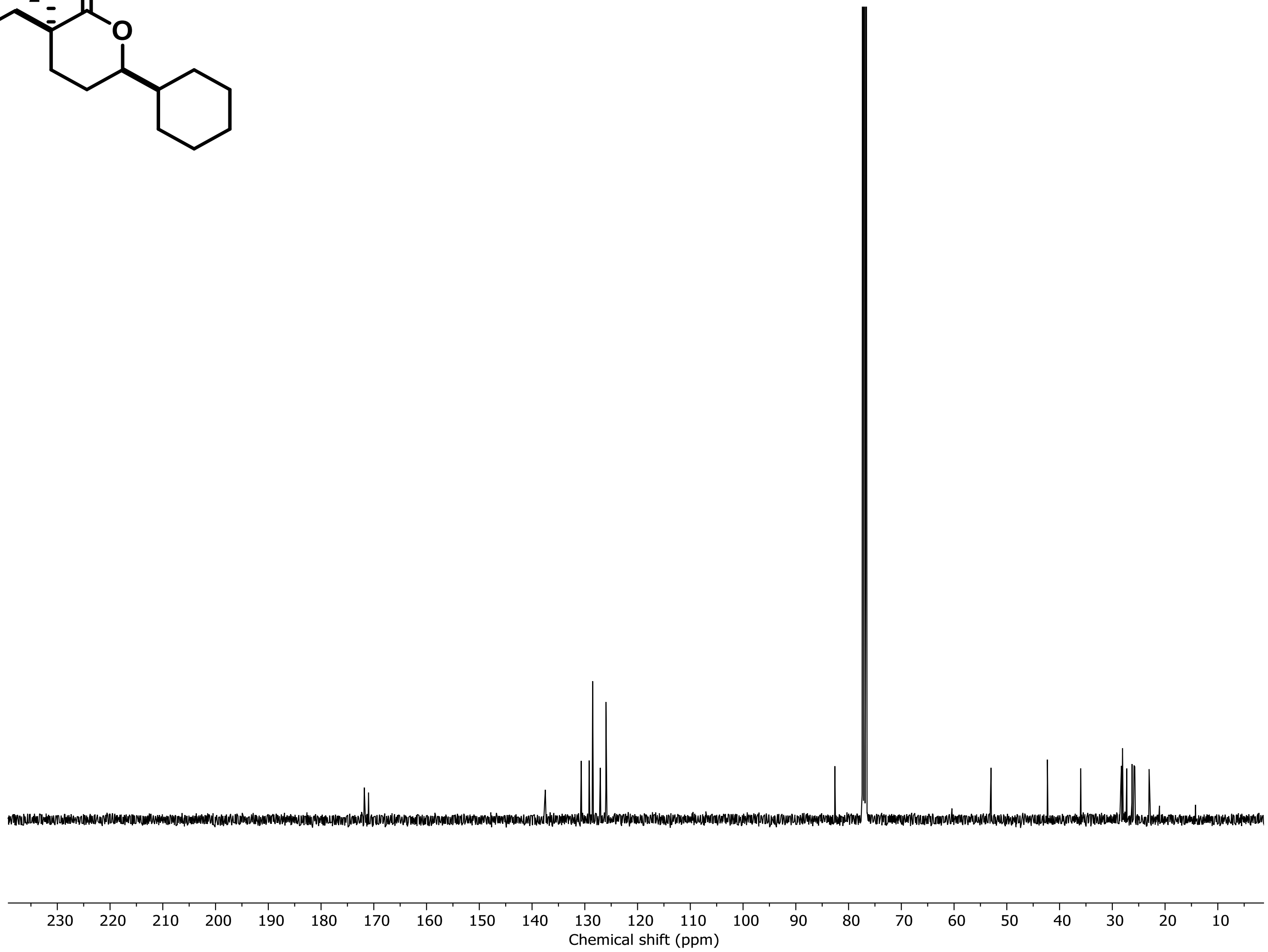

***Rac*–methyl (2*R*,5*R*)–6–oxo–5–((*E*)–4–phenylbut–3–en–1–yl)octahydro–2*H*,2'*H*–[2,4'–bipyran]–5–carboxylate (1i)**

^{1}H NMR (500 MHz, $CDCl_3$) 1.9:1 d.r.

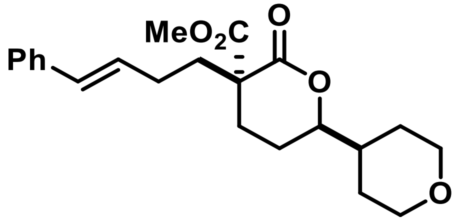

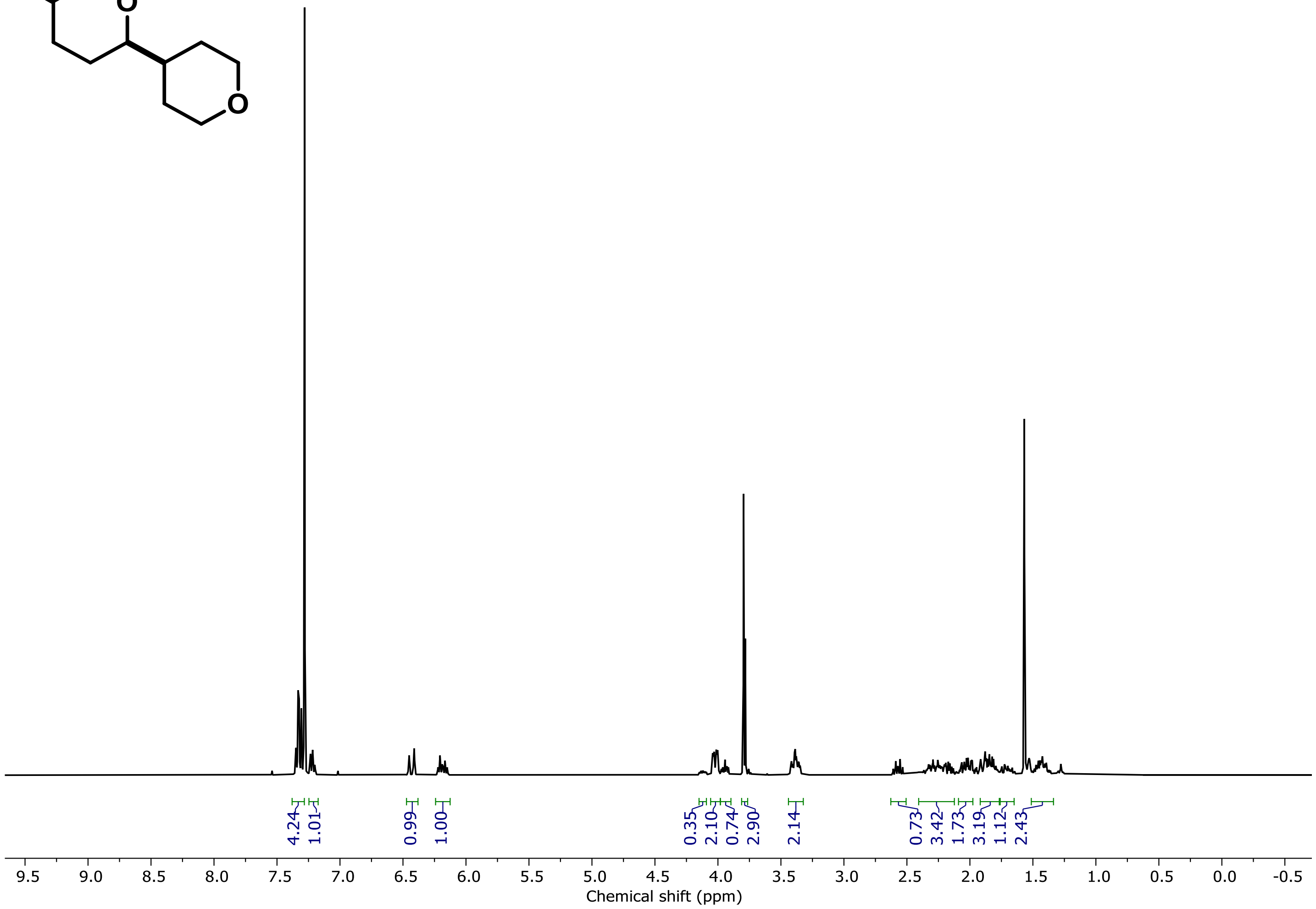

^{13}C NMR (126 MHz, $CDCl_3$) 1.9:1 d.r.

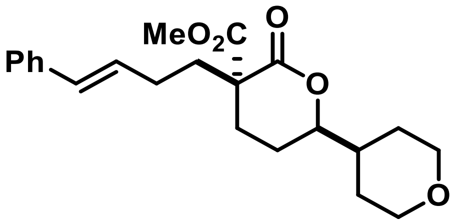

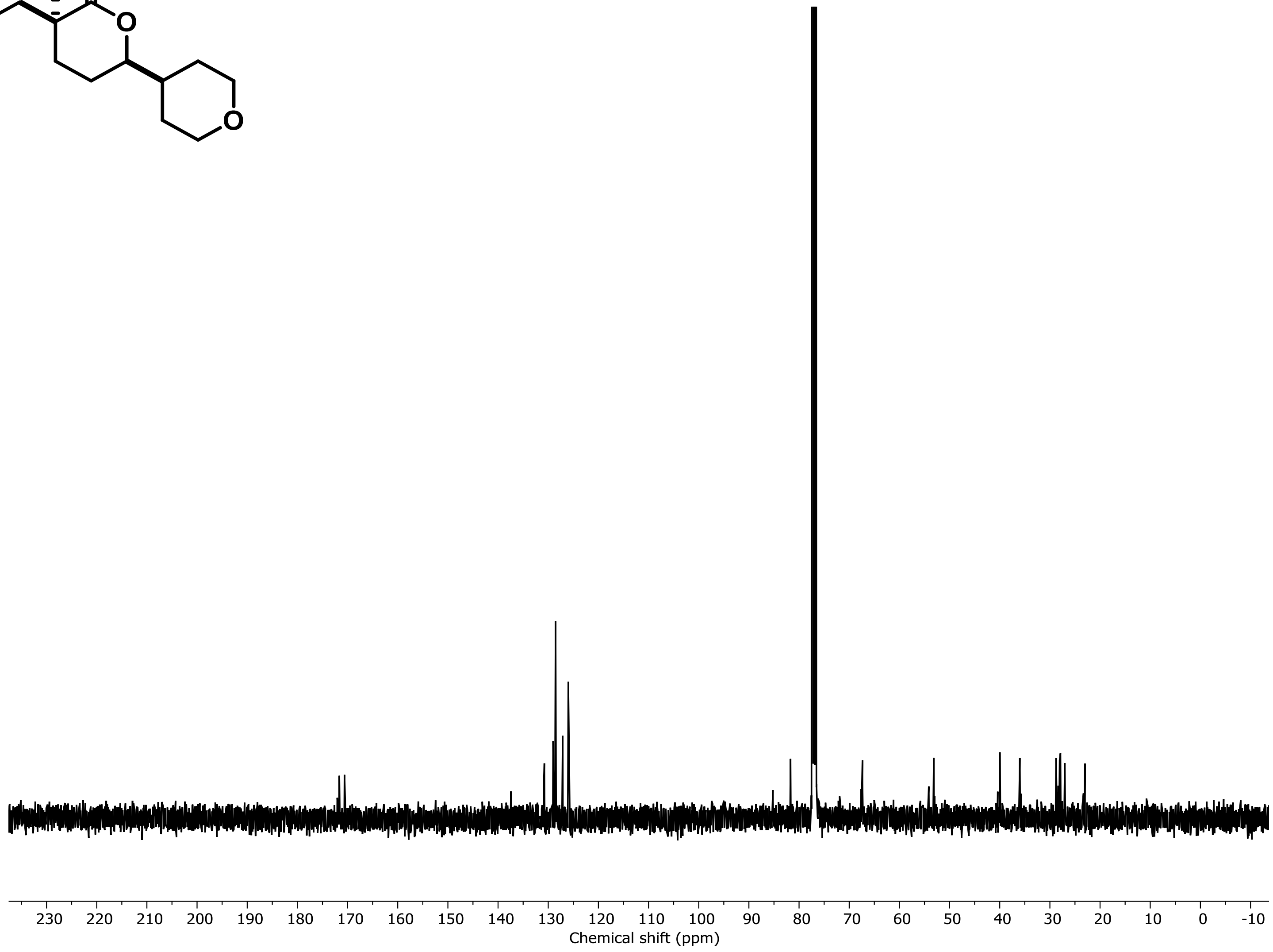

***Rac*–methyl (3*R*,6*R*)–6–(tert–butyl)–2–oxo–3–((*E*)–4–phenylbut–3–en–1–yl)tetrahydro–2*H*–pyran–3–carboxylate (1j)**

^{1}H NMR (400 MHz, $CDCl_3$)

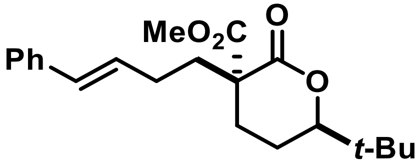

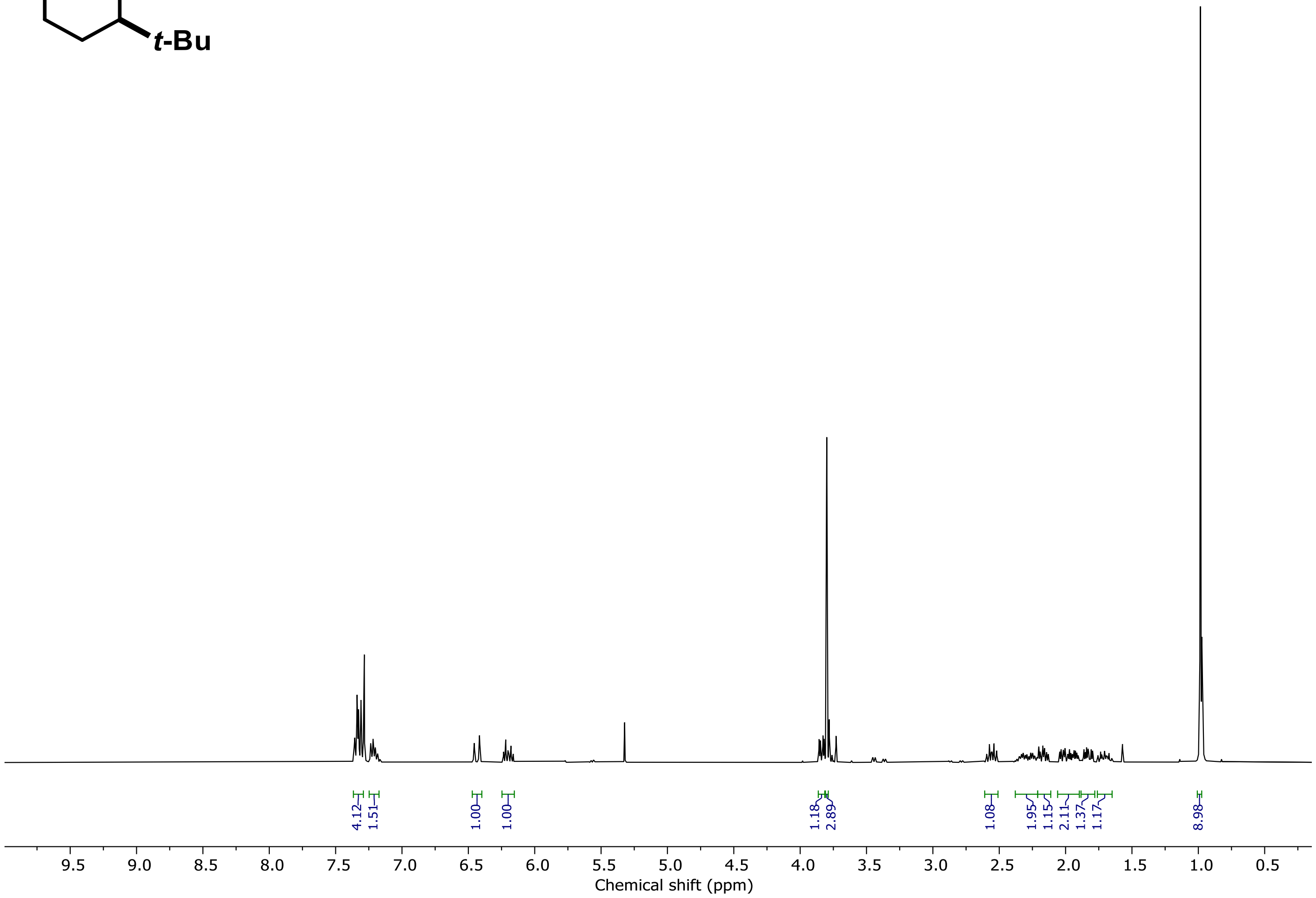

^{13}C NMR (101 MHz, $CDCl_3$)

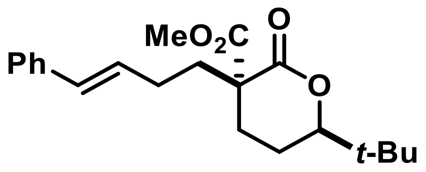

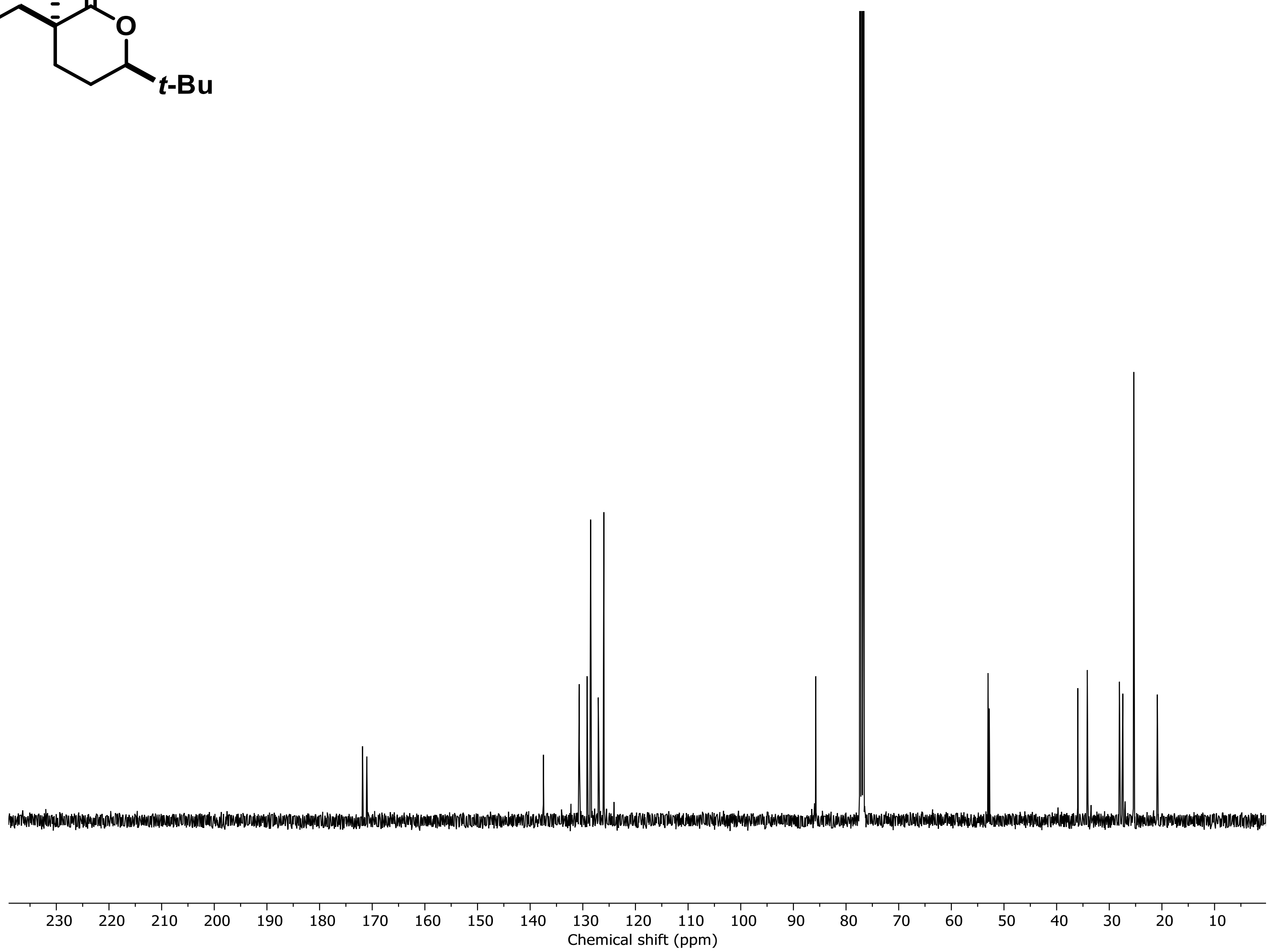

***Rac*–methyl (3*R*,6*S*)–6–ethyl–3–((*E*)–4–(2–fluorophenyl)but–3–en–1–yl)–2–oxotetrahydro–2*H*–pyran–3–carboxylate (1k)**

^{1}H NMR (500 MHz, $CDCl_3$) 5.2:1 d.r.

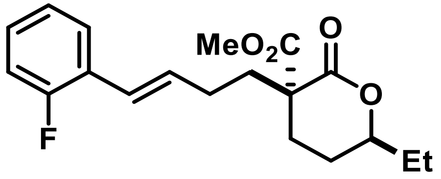

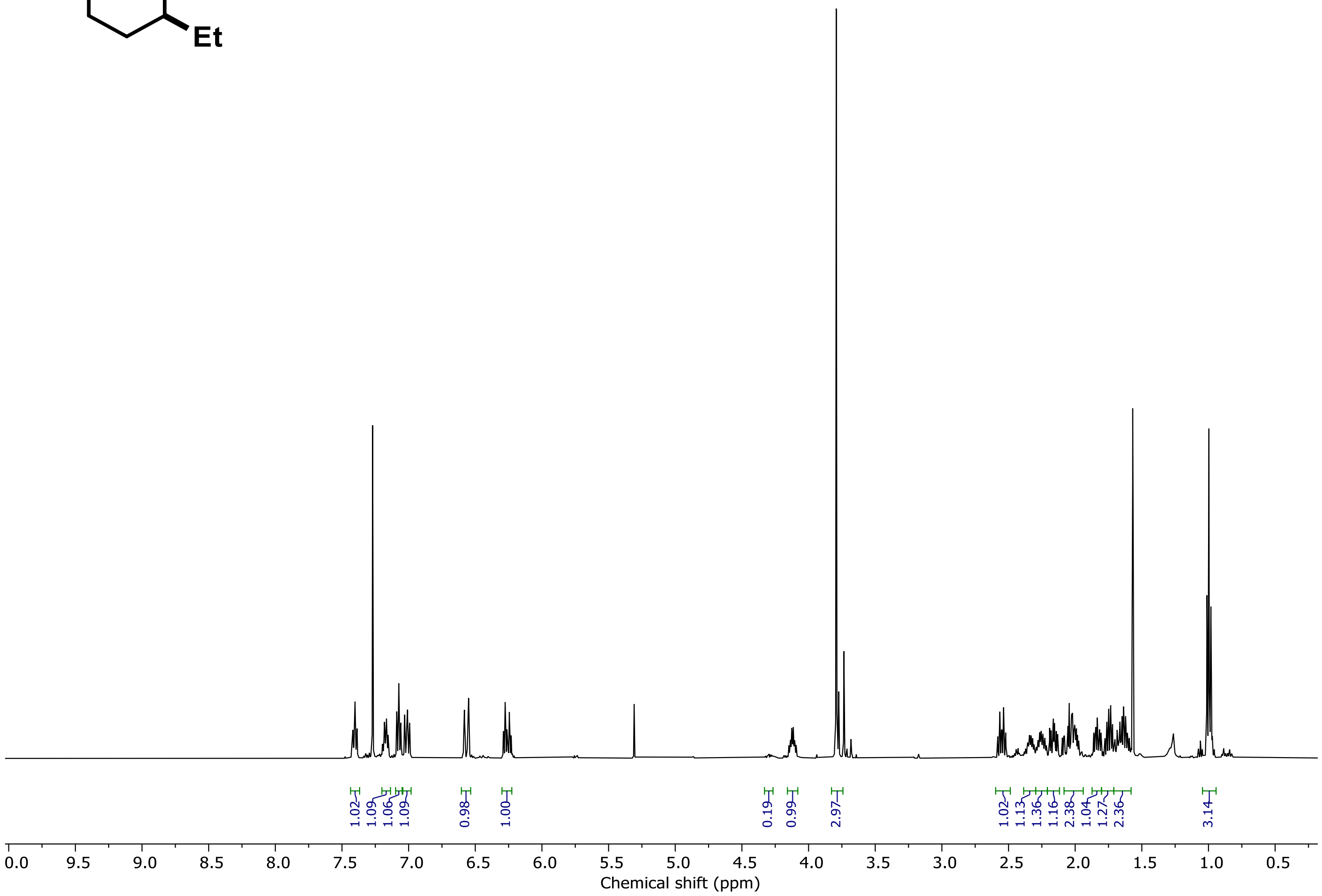

^{13}C NMR (126 MHz, $CDCl_3$) 5.2:1 d.r.

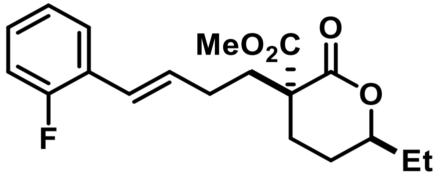

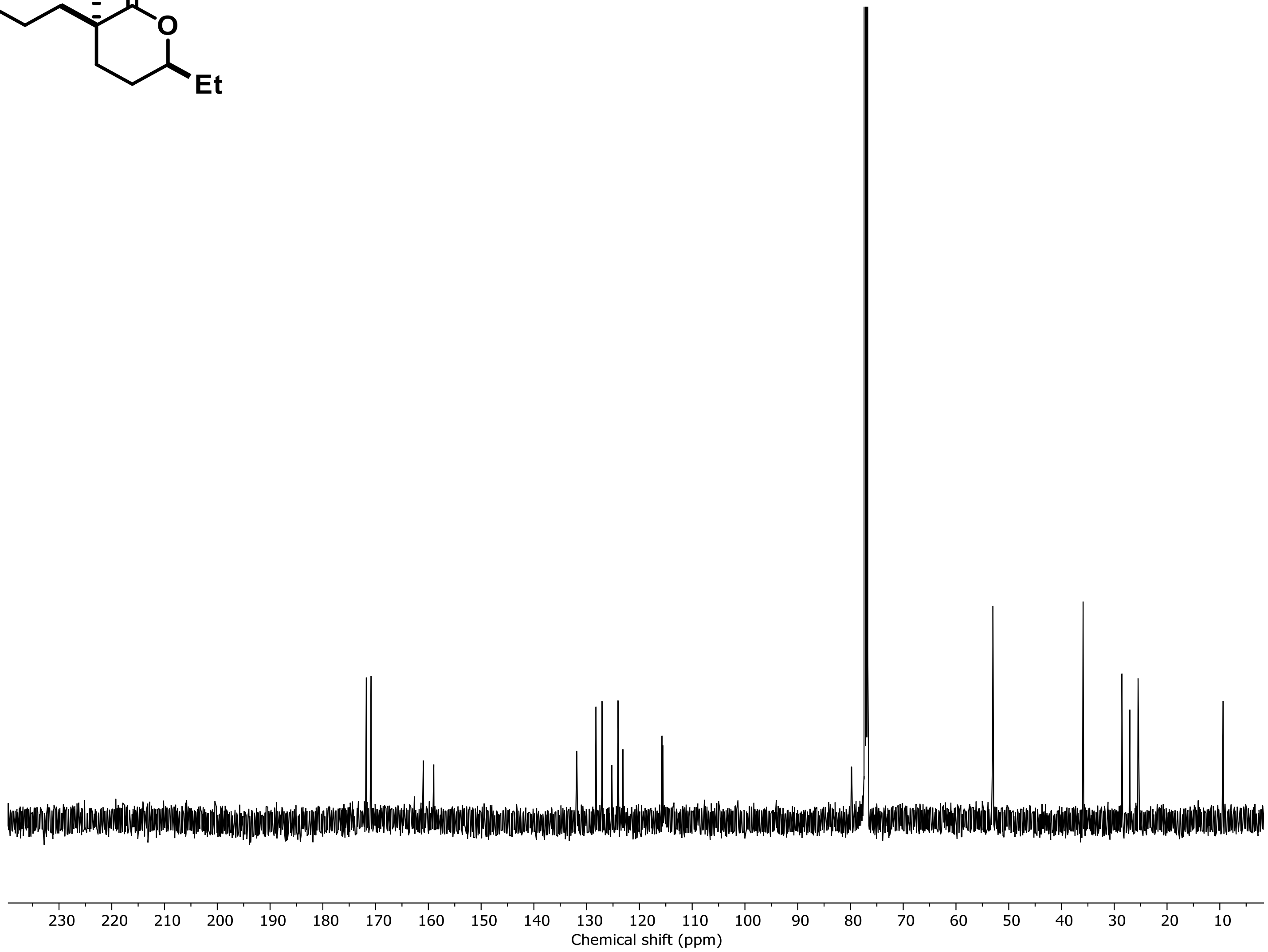

^{19}F NMR (376 MHz, $CDCl_3$) 5.2:1 d.r.

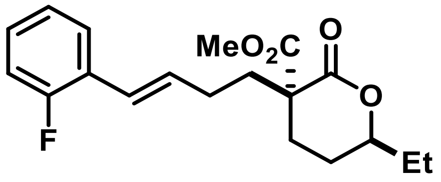

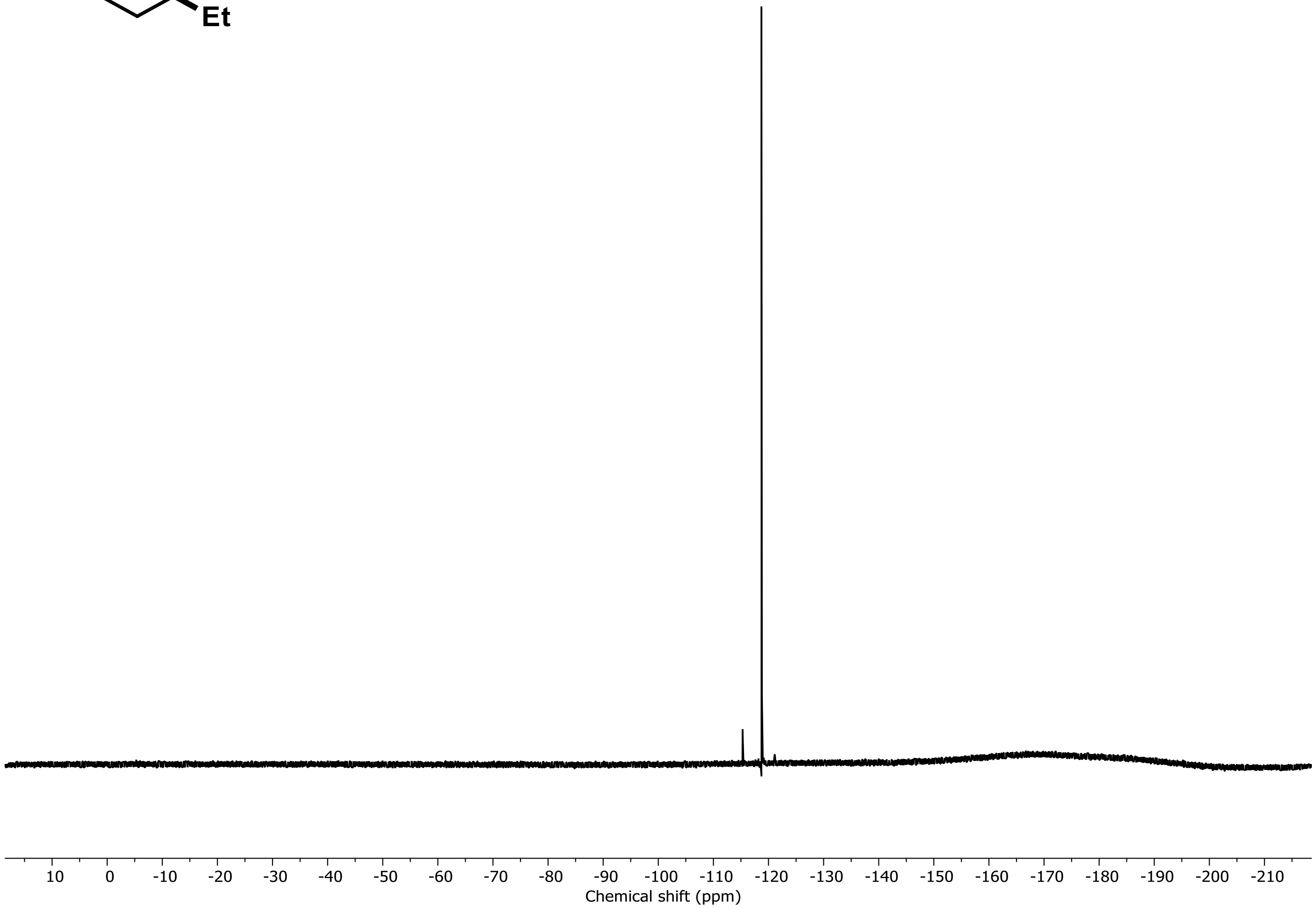

Rac–methyl (3_R_,6_S_)–6–ethyl–2–oxo–3–((_E_)–4–(o–tolyl)but–3–en–1–yl)tetrahydro–2_H_–pyran–3–carboxylate (1l)

^{1}H NMR (400 MHz, $CDCl_3$)

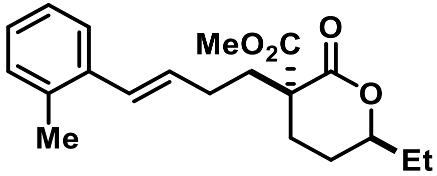

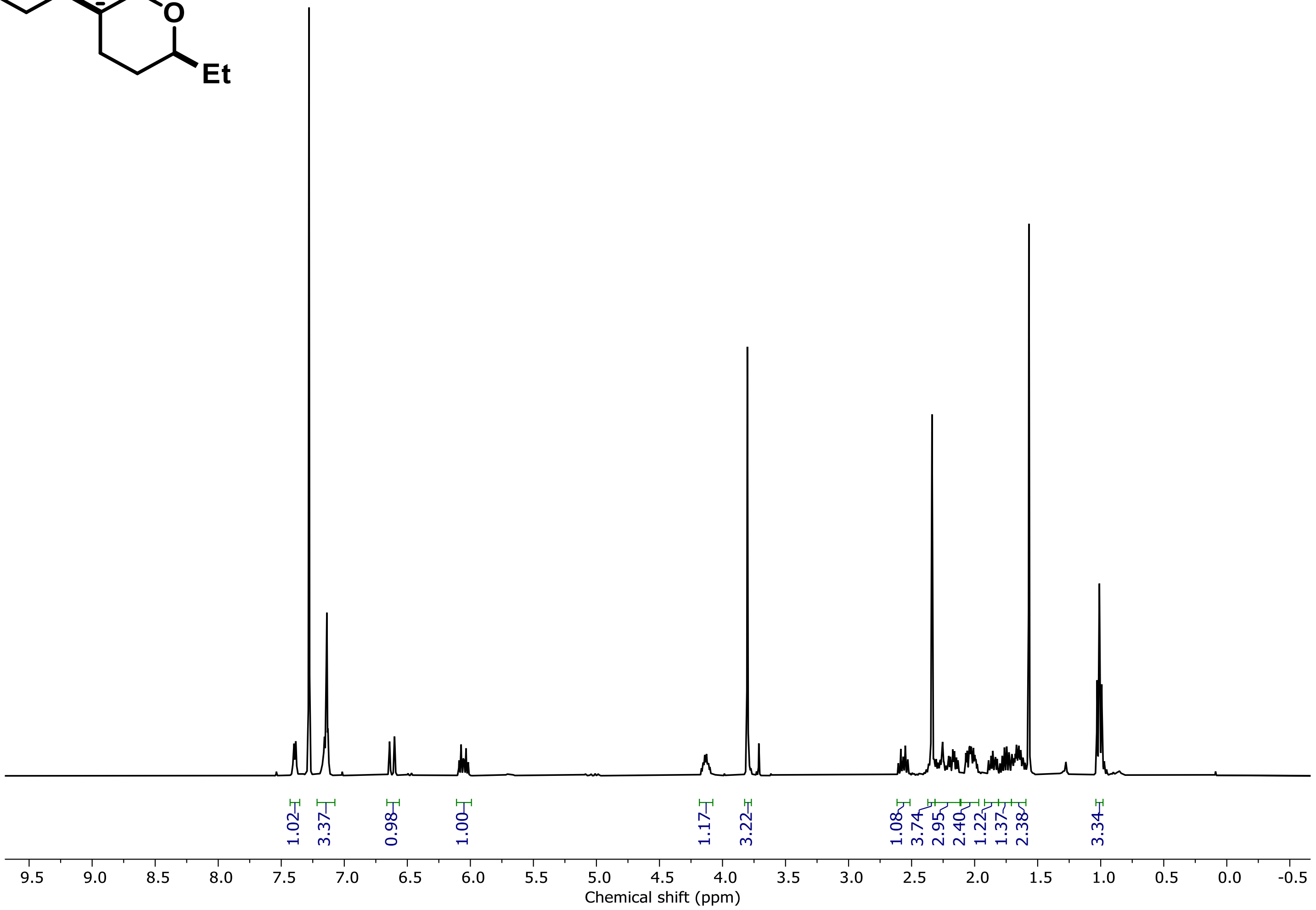

^{13}C NMR (101 MHz, $CDCl_3$)

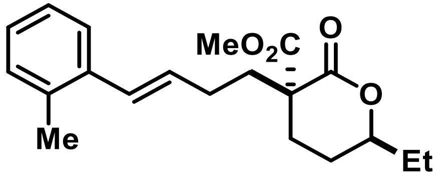

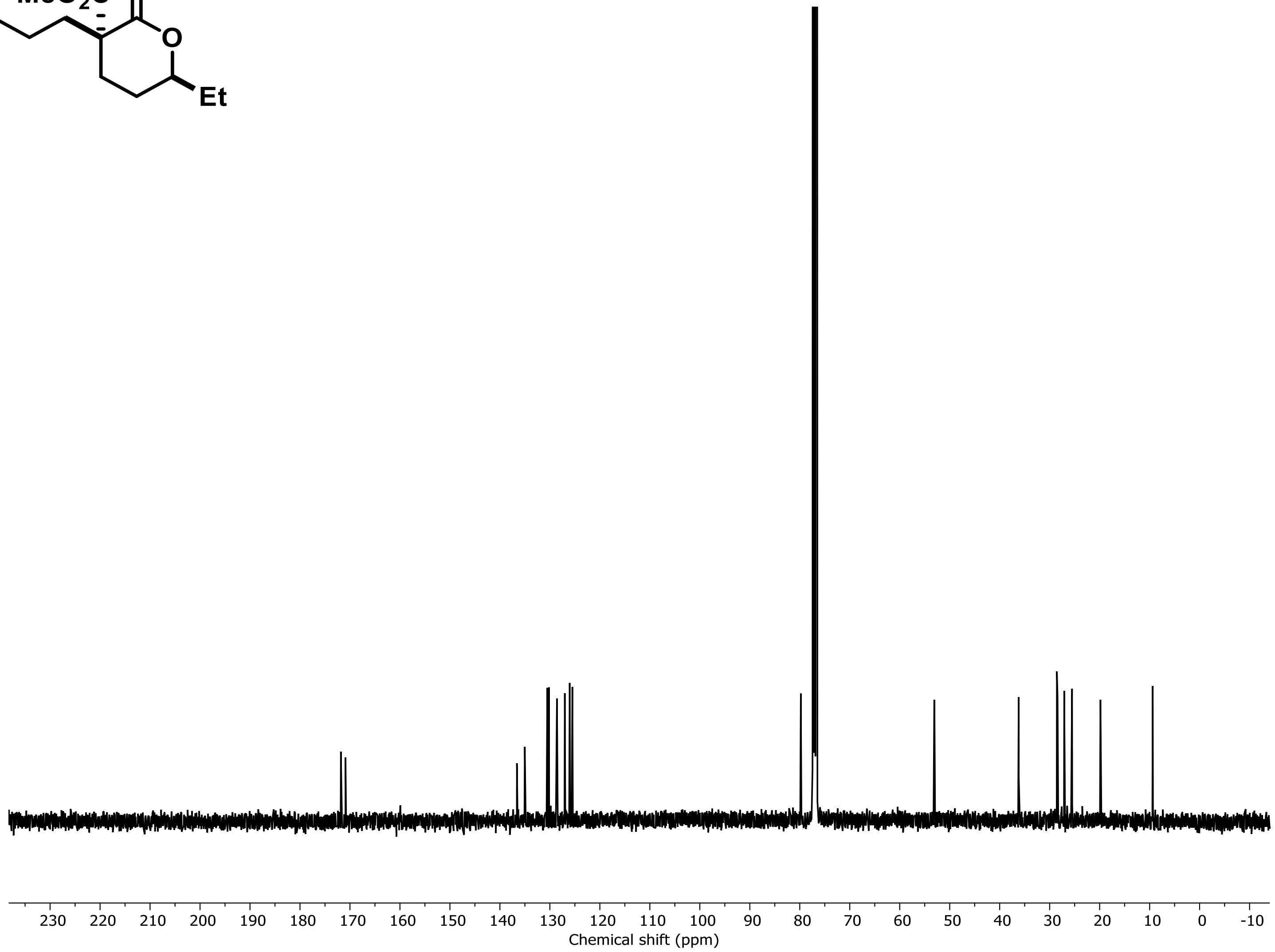

***Rac*–methyl (3*R*,6*S*)–6–ethyl–2–oxo–3–((*E*)–4–(m–tolyl)but–3–en–1–yl)tetrahydro–2*H*–pyran–3–carboxylate (1m)**

^{1}H NMR (400 MHz, $CDCl_3$)

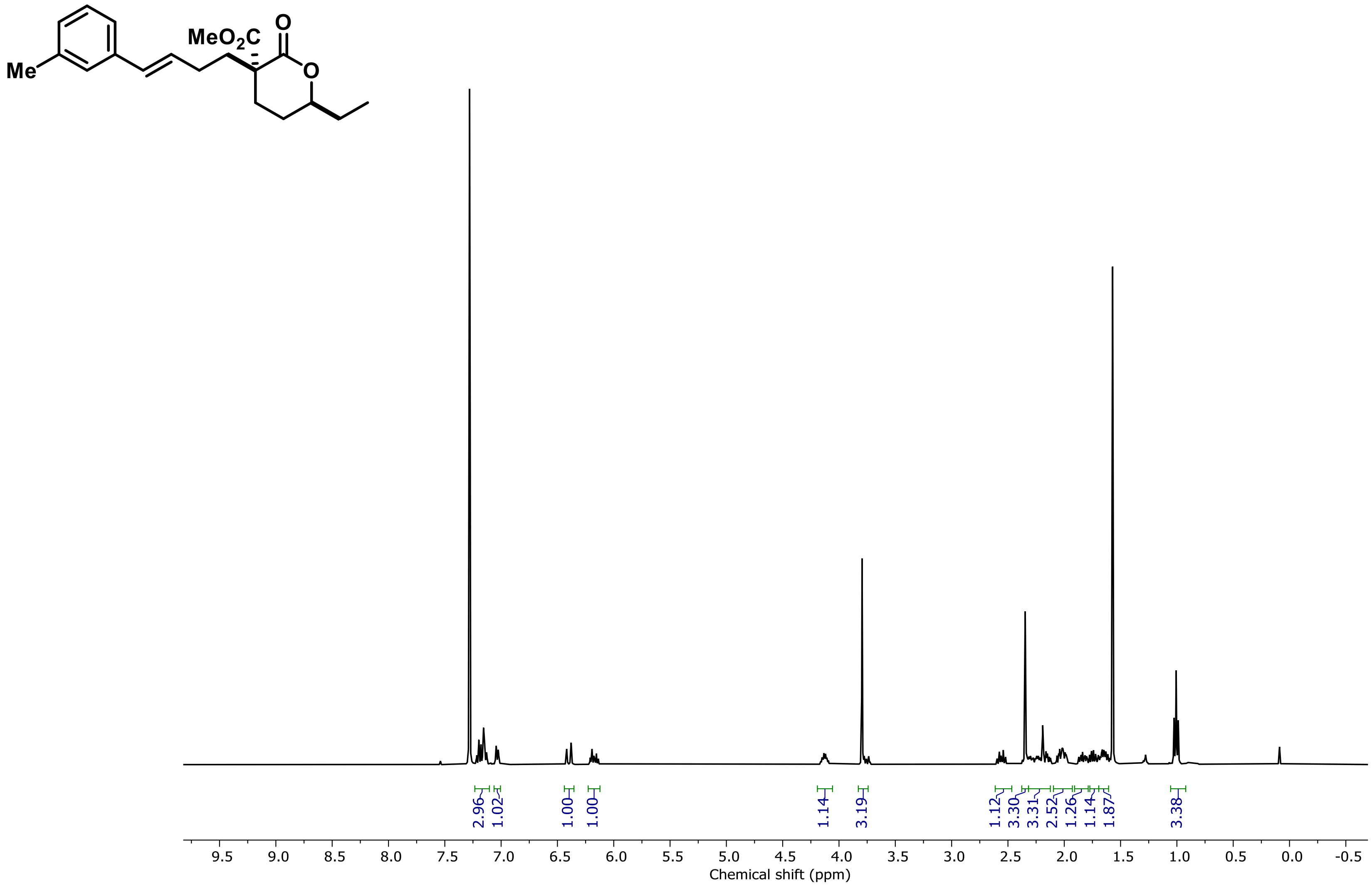

^{13}C NMR (101 MHz, $CDCl_3$)

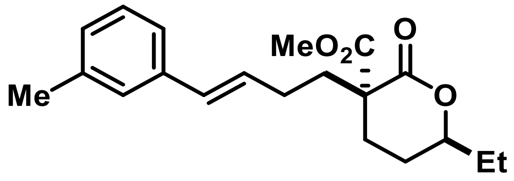

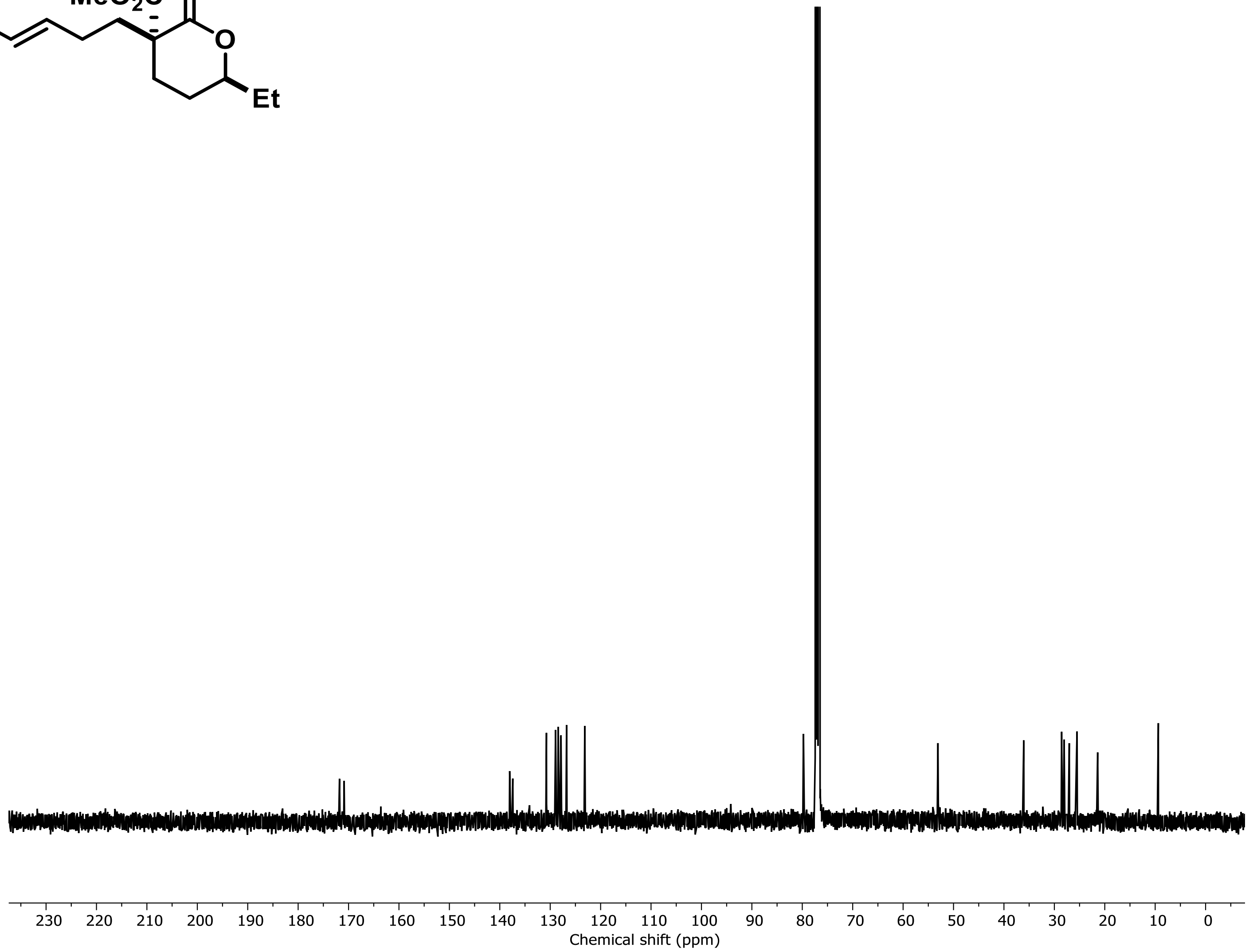

***Rac*–methyl (3*R*,6*S*)–3–((*E*)–4–(3–chlorophenyl)but–3–en–1–yl)–6–ethyl–2–oxotetrahydro–2*H*–pyran–3–carboxylate (1n)**

^{1}H NMR (400 MHz, $CDCl_3$)

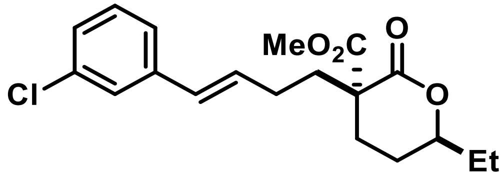

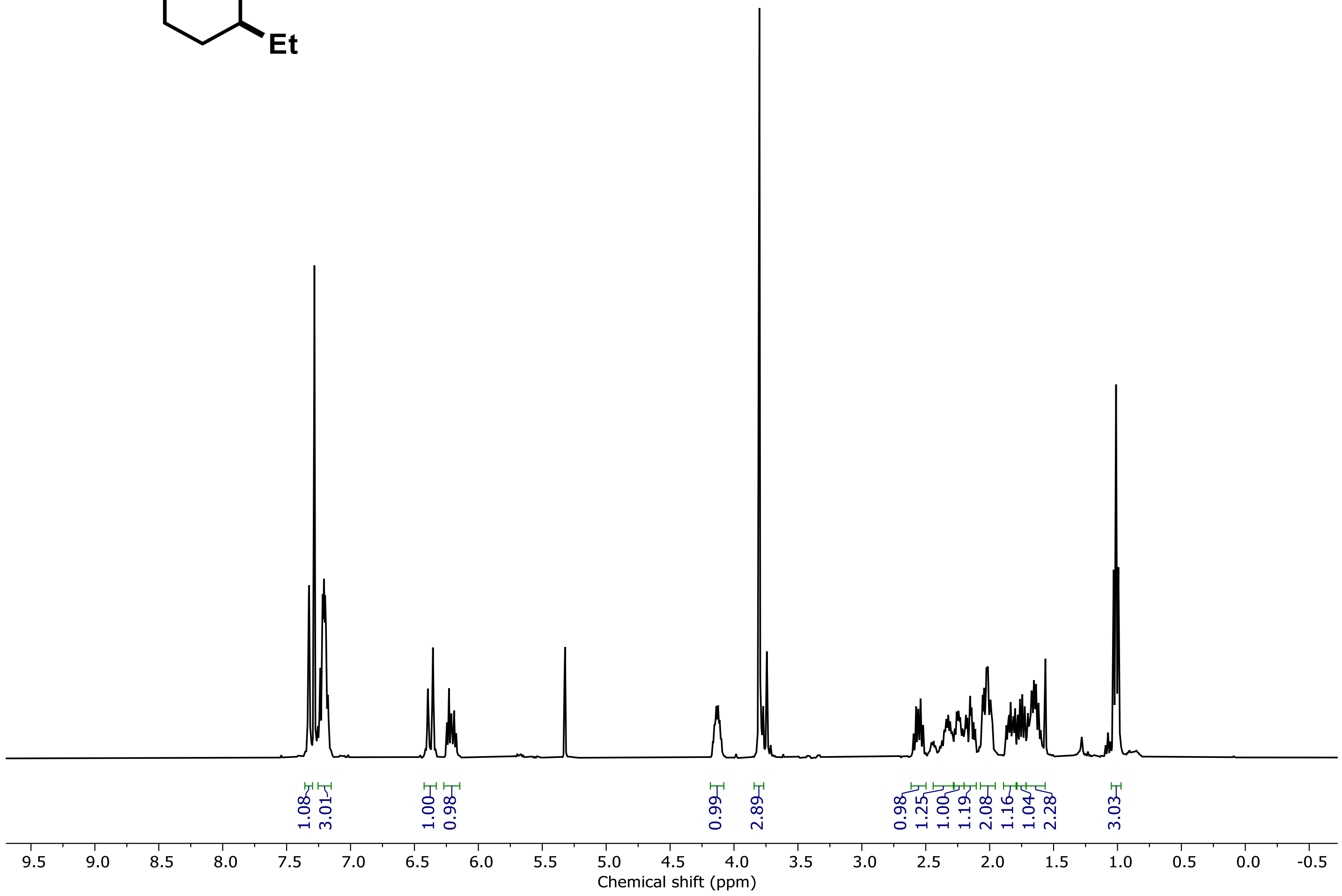

^{13}C NMR (101 MHz, $CDCl_3$)

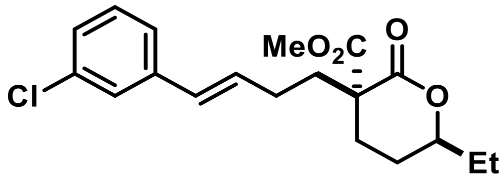

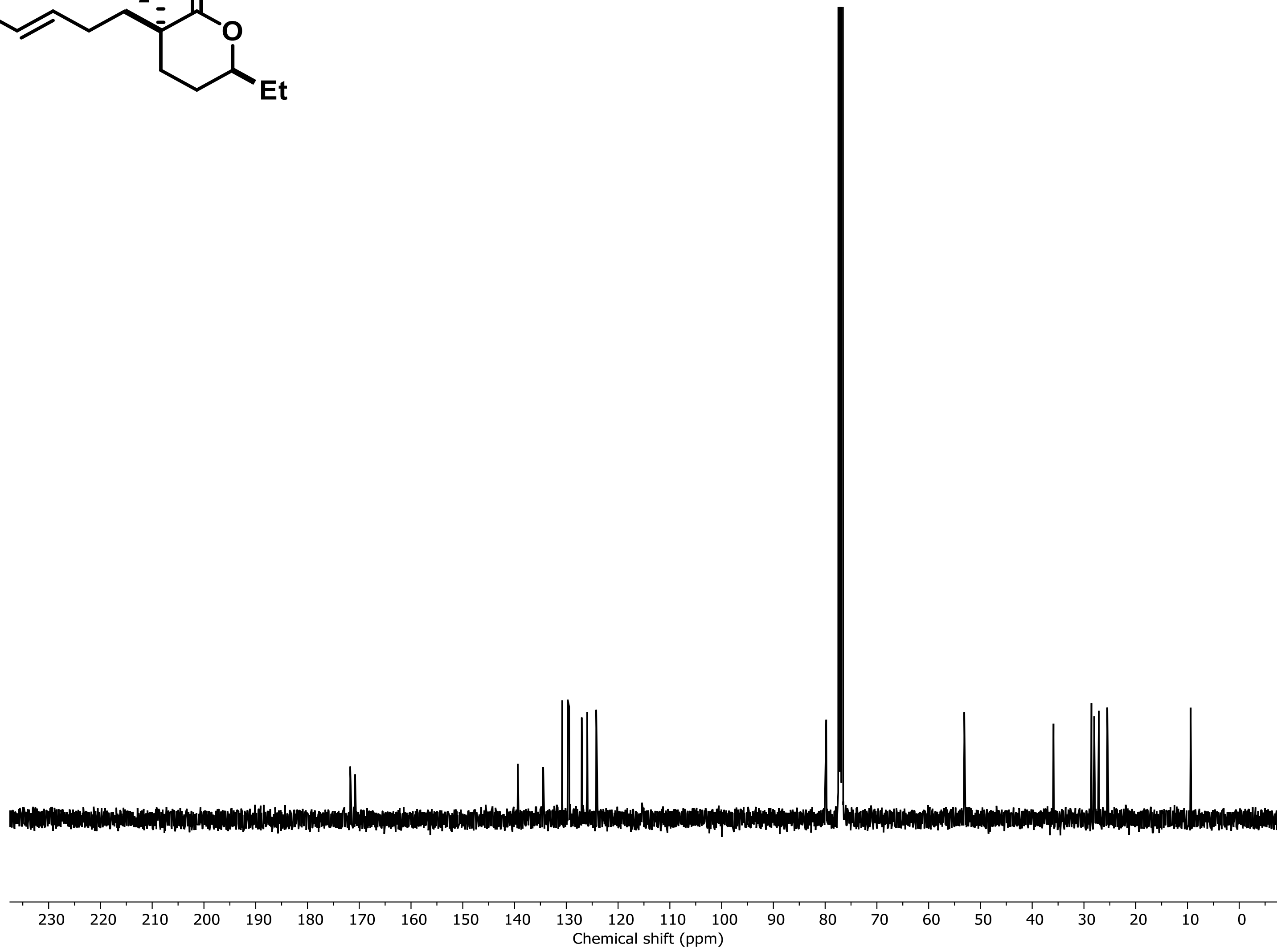

Rac–methyl (3_R_,6_S_)–6–ethyl–3–((_E_)–4–(naphthalen–2–yl)but–3–en–1–yl)–2–oxotetrahydro–2_H_–pyran–3–carboxylate (1o)

^{1}H NMR (400 MHz, $CDCl_3$) 6.4:1 d.r.

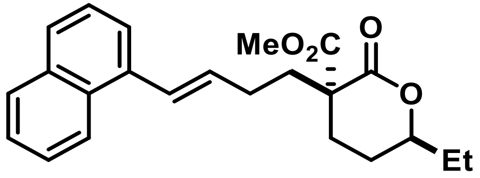

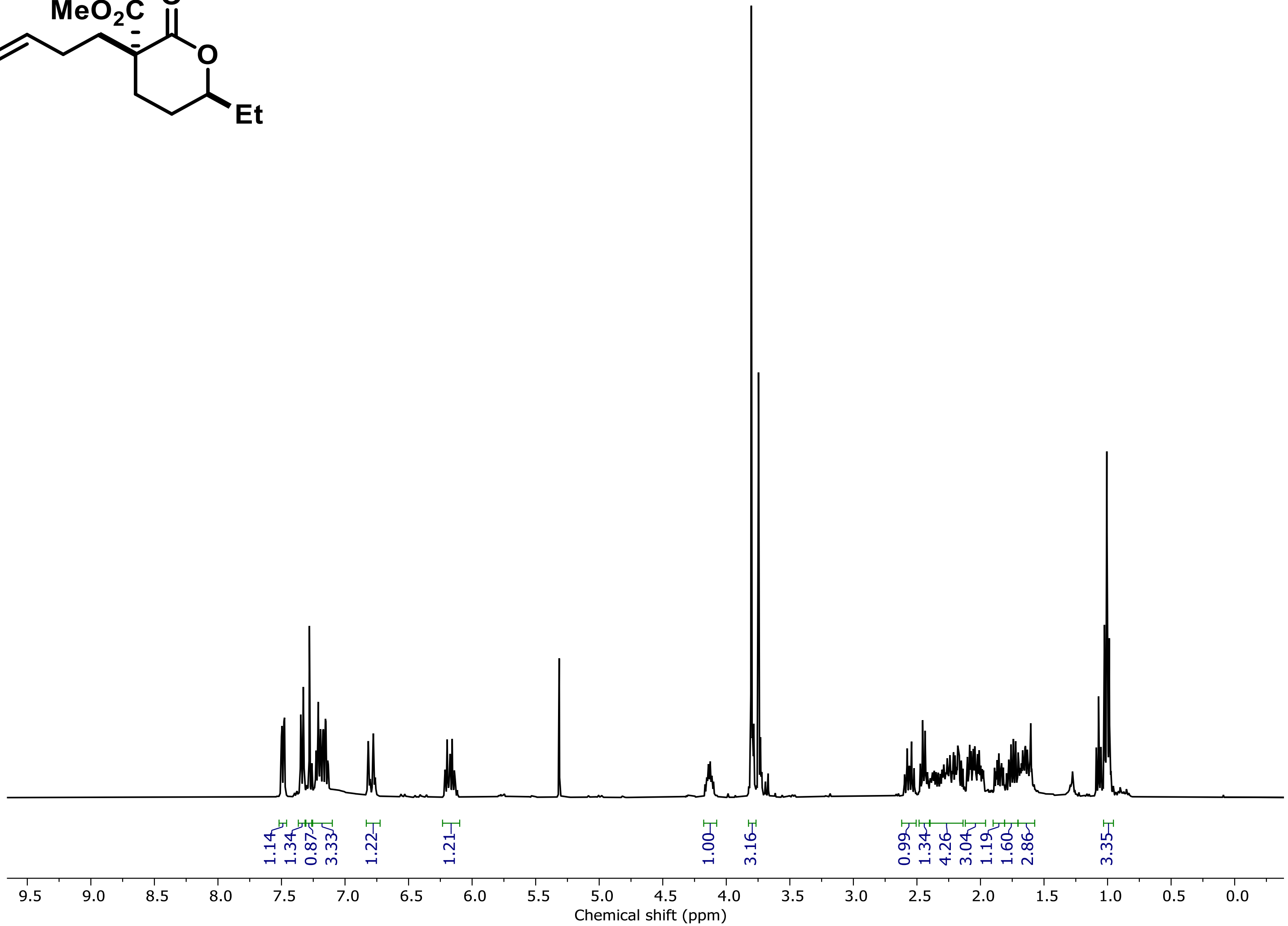

^{13}C NMR (101 MHz, $CDCl_3$) 6.4:1 d.r.

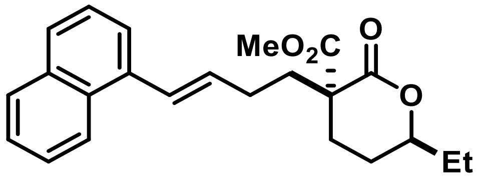

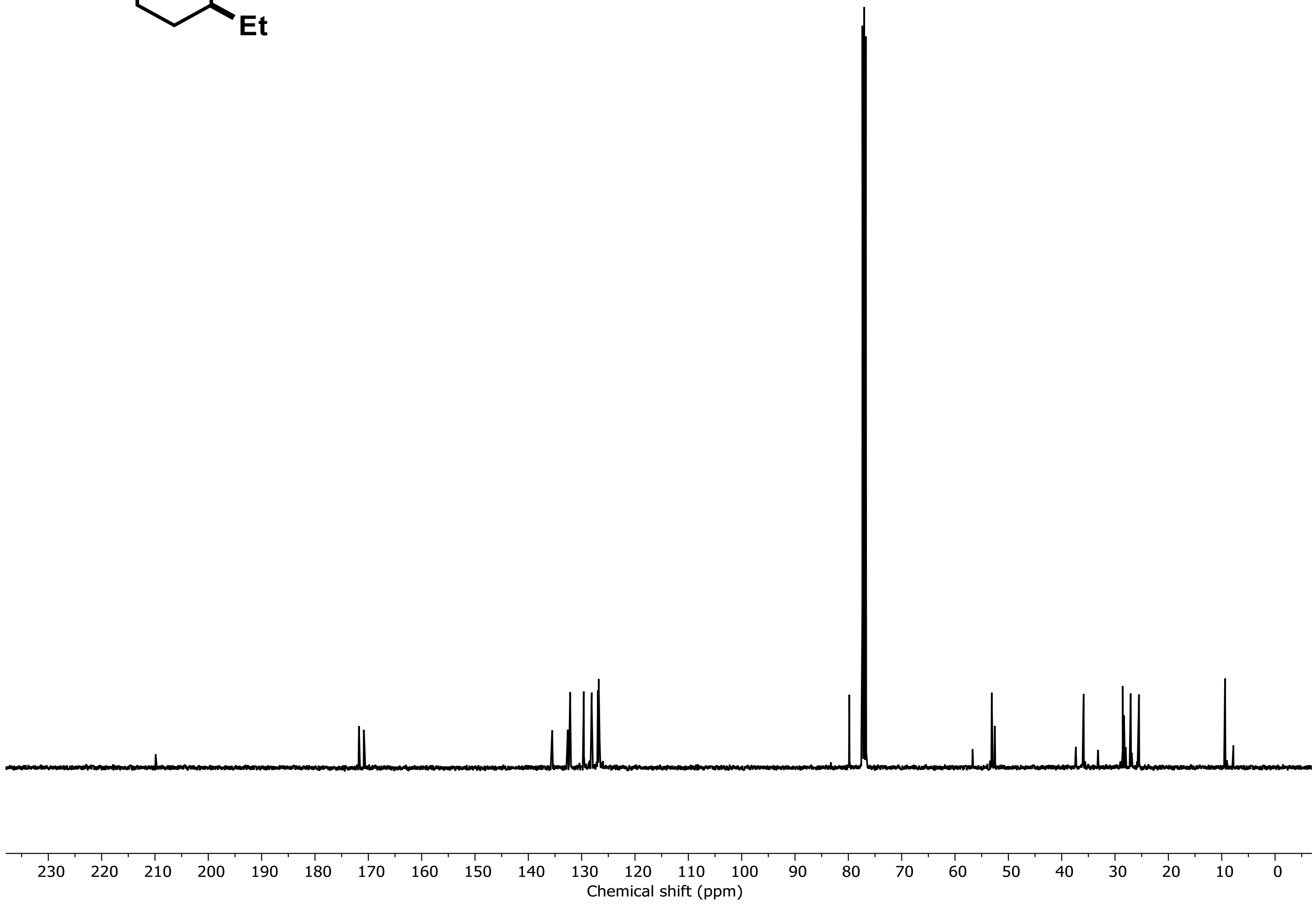

Rac–methyl (3*R*,6*S*)–3–((*E*)–4–([1,1'–biphenyl]–4–yl)but–3–en–1–yl)–6–ethyl–2–oxotetrahydro–2*H*–pyran–3–carboxylate (1p)

^{1}H NMR (400 MHz, $CDCl_3$)

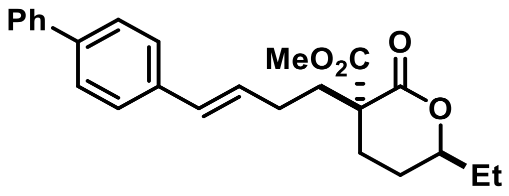

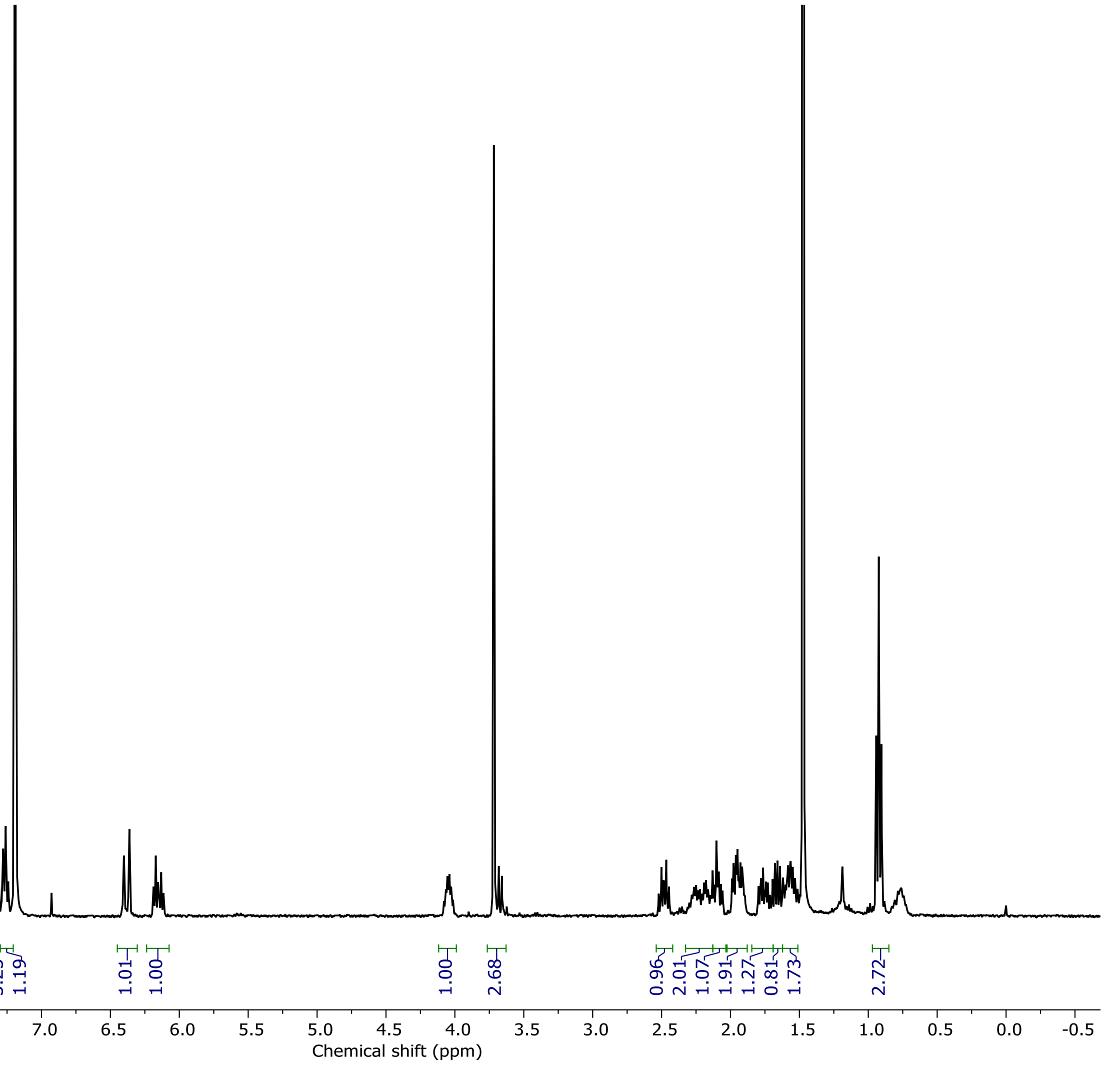

^{13}C NMR (126 MHz, $CDCl_3$)

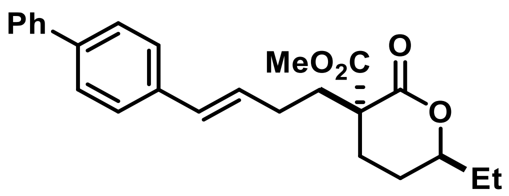

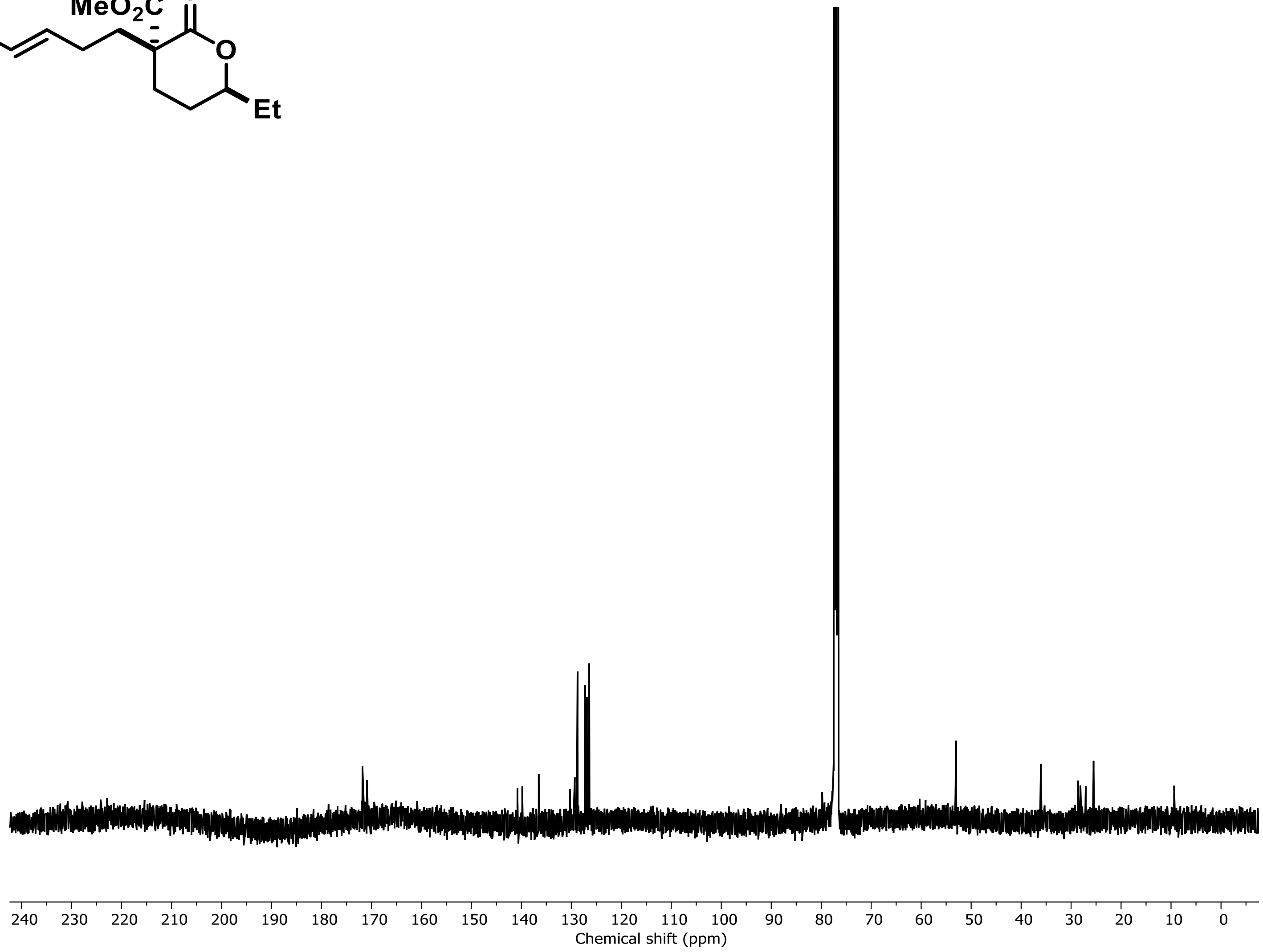

Rac–methyl (3_R_,6_S_)–6–ethyl–3–((_E_)–4–(4–fluorophenyl)but–3–en–1–yl)–2–oxotetrahydro–2_H_–pyran–3–carboxylate (1q)

^{1}H NMR (400 MHz, $CDCl_3$)

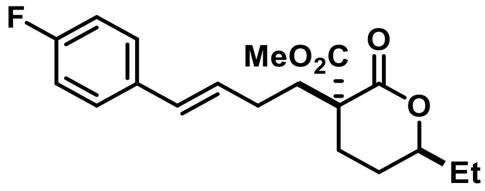

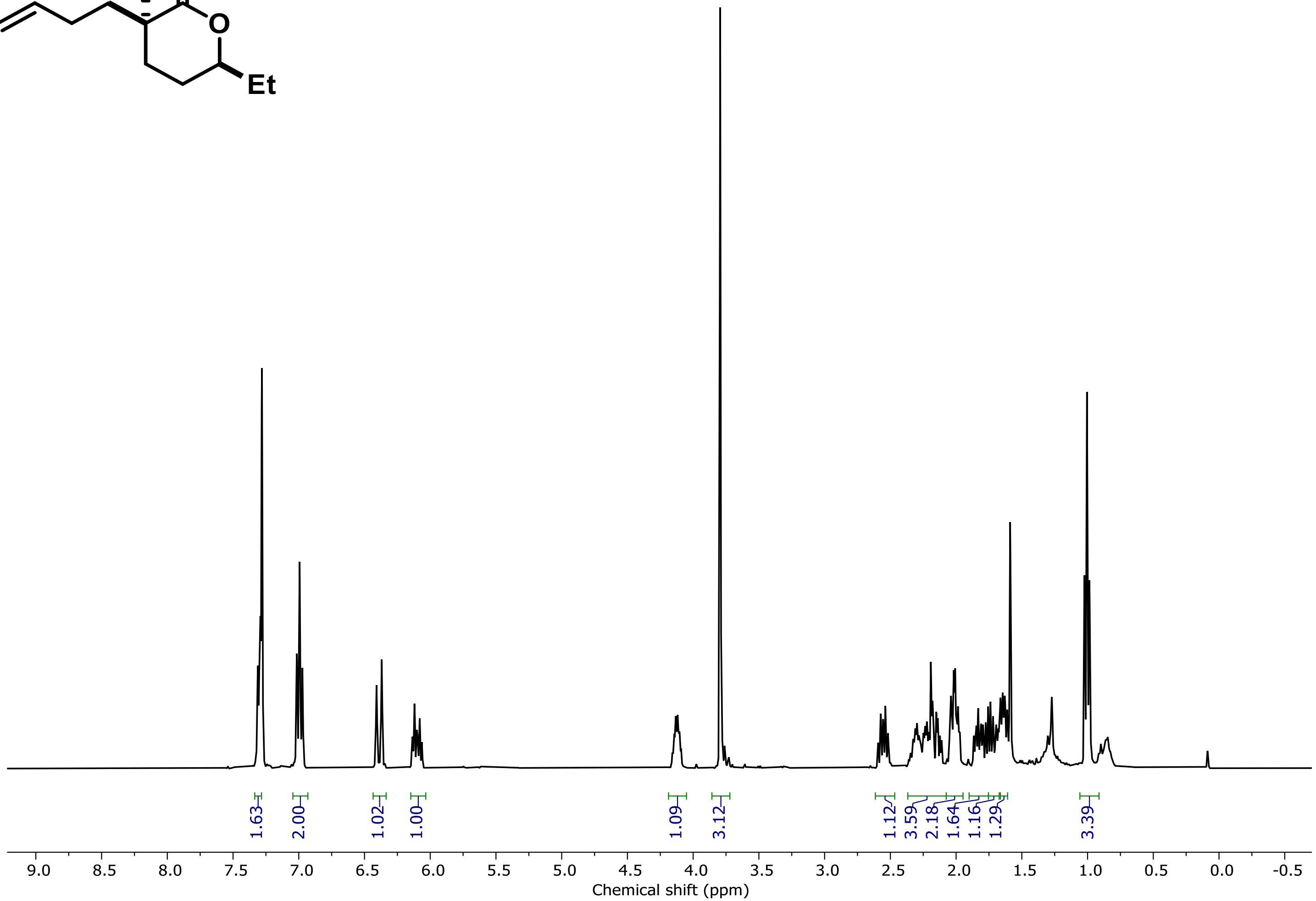

^{13}C NMR (101 MHz, $CDCl_3$)

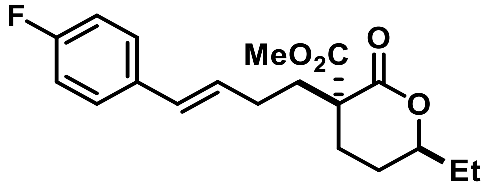

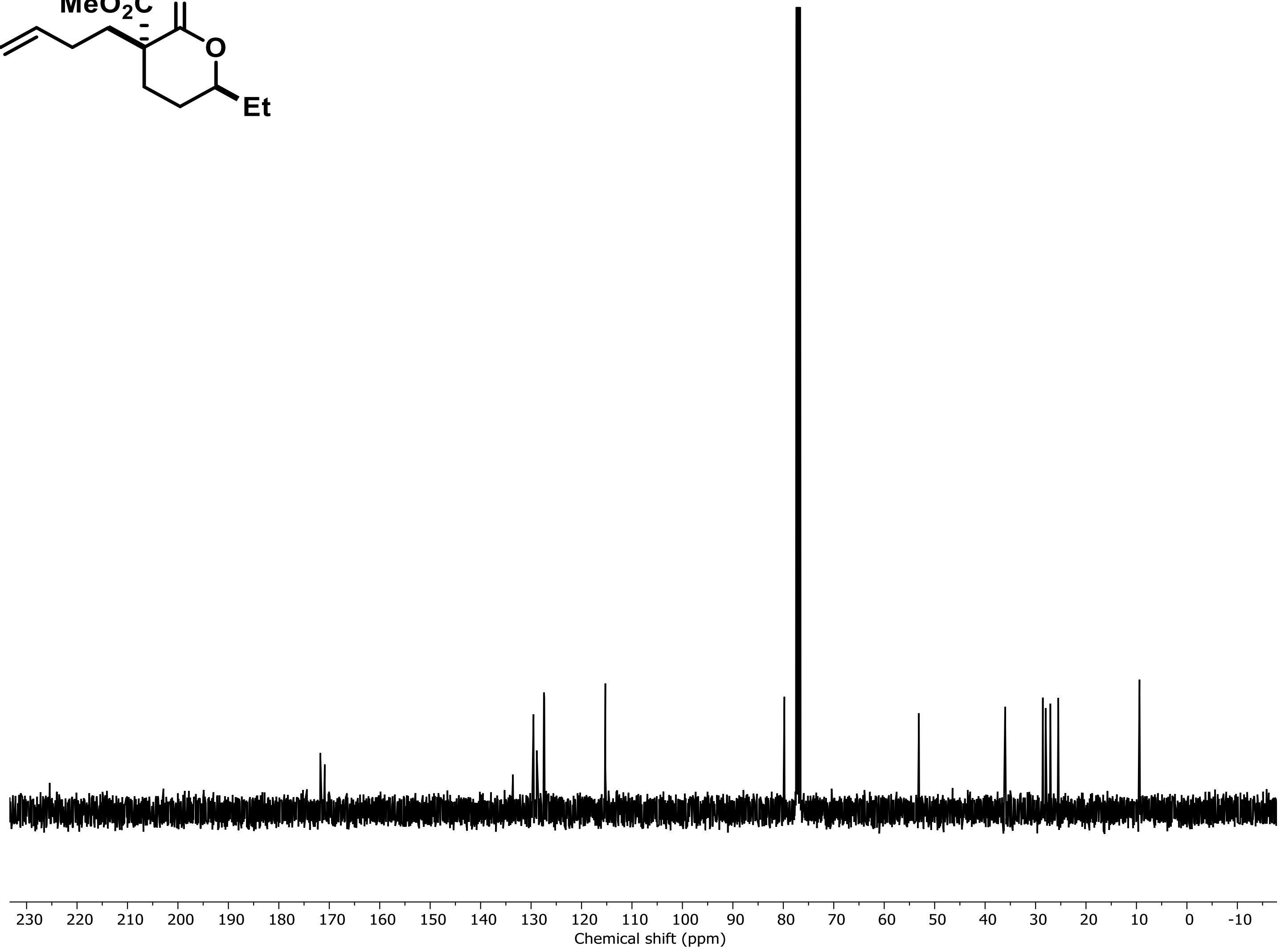

^{19}F NMR (471 MHz, $CDCl_3$)

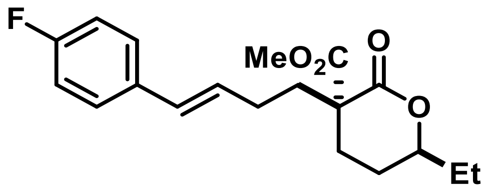

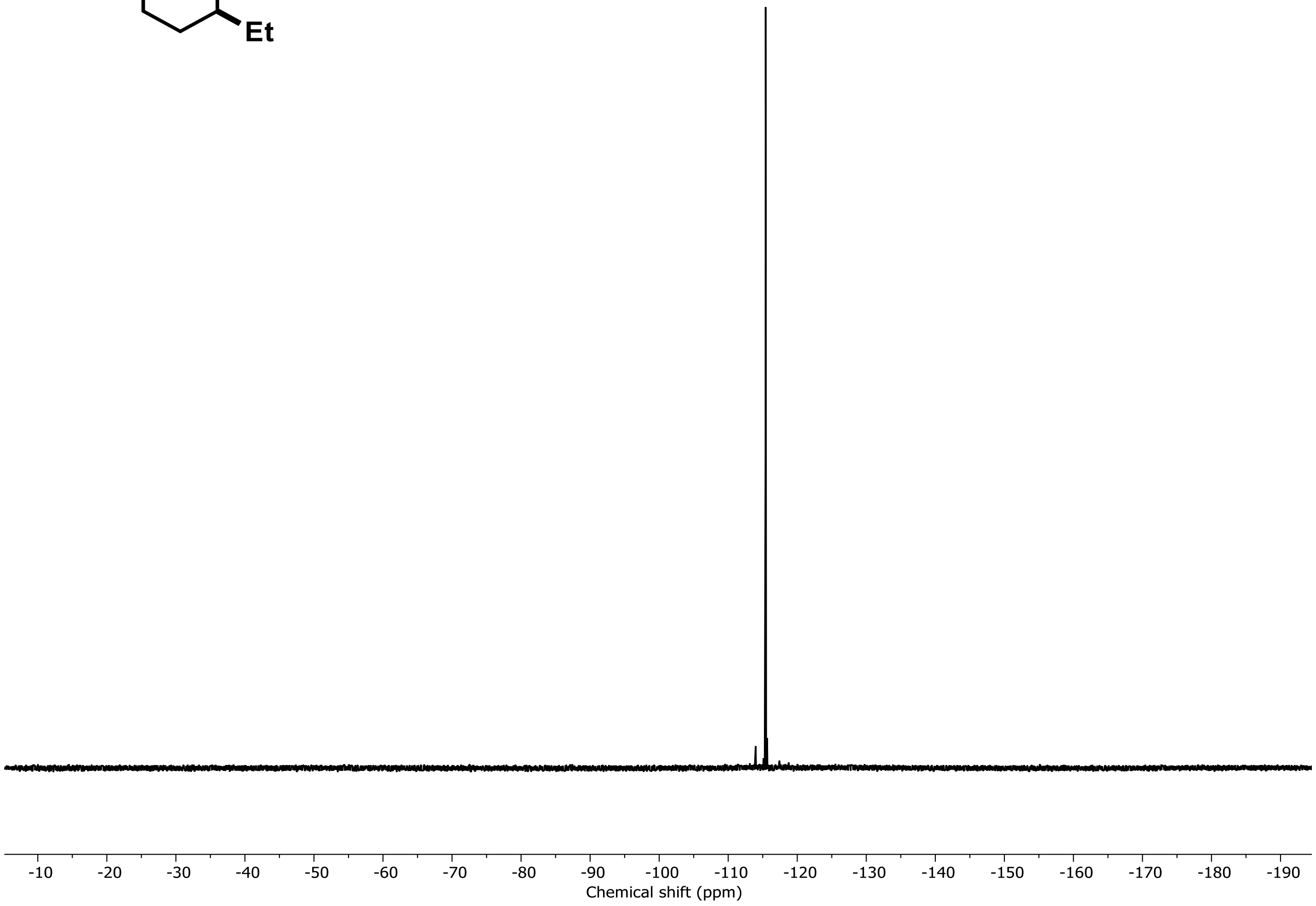

***Rac*–methyl (3*R*,6*S*)–3–((*E*)–4–(4–bromophenyl)but–3–en–1–yl)–6–ethyl–2–oxotetrahydro–2*H*–pyran–3–carboxylate (1r)**

^{1}H NMR (400 MHz, $CDCl_3$) 6.2:1 d.r.

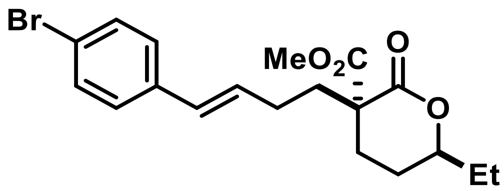

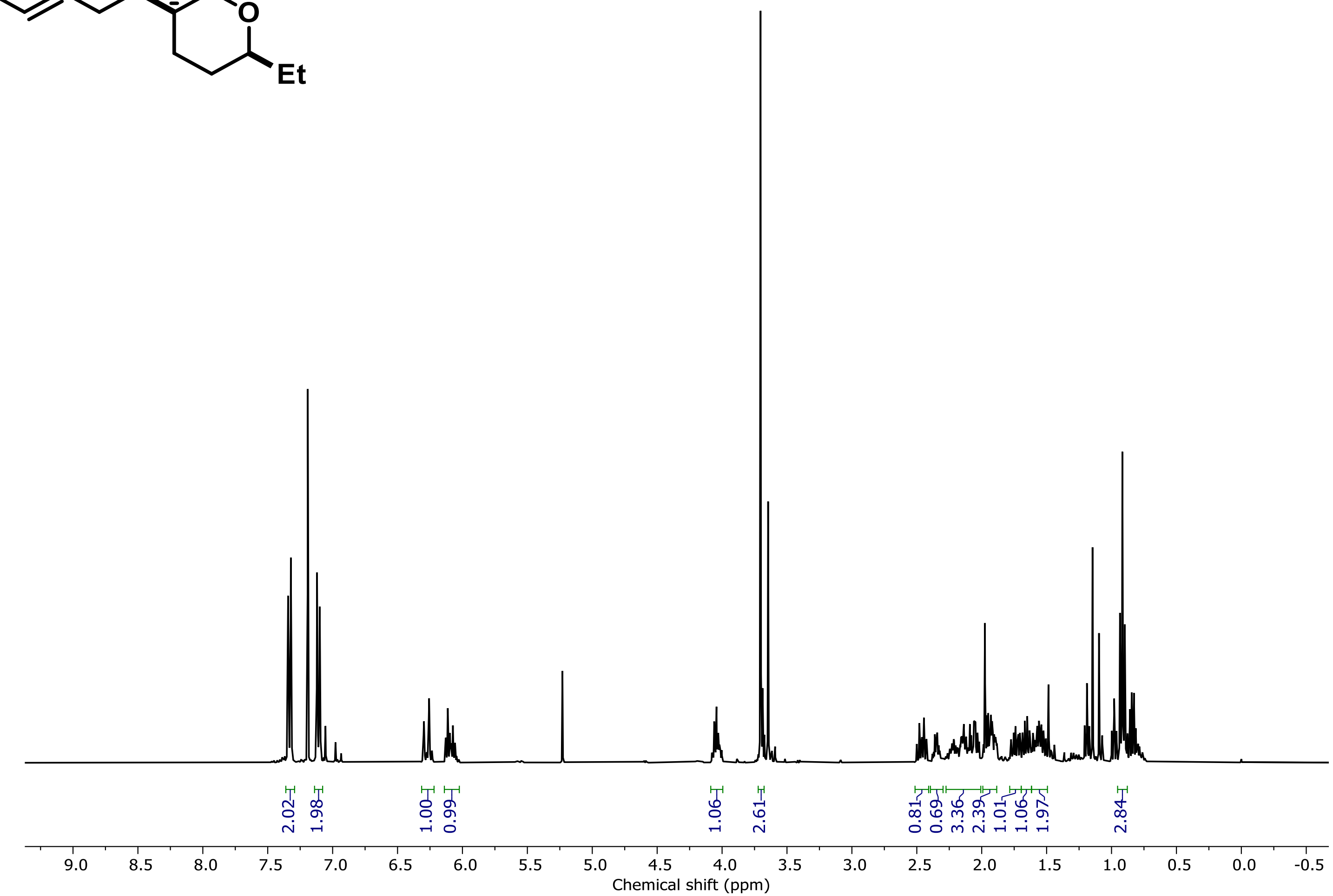

^{13}C NMR (101 MHz, $CDCl_3$) 6.2:1 d.r.

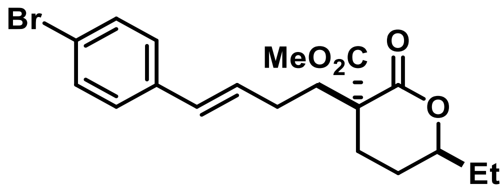

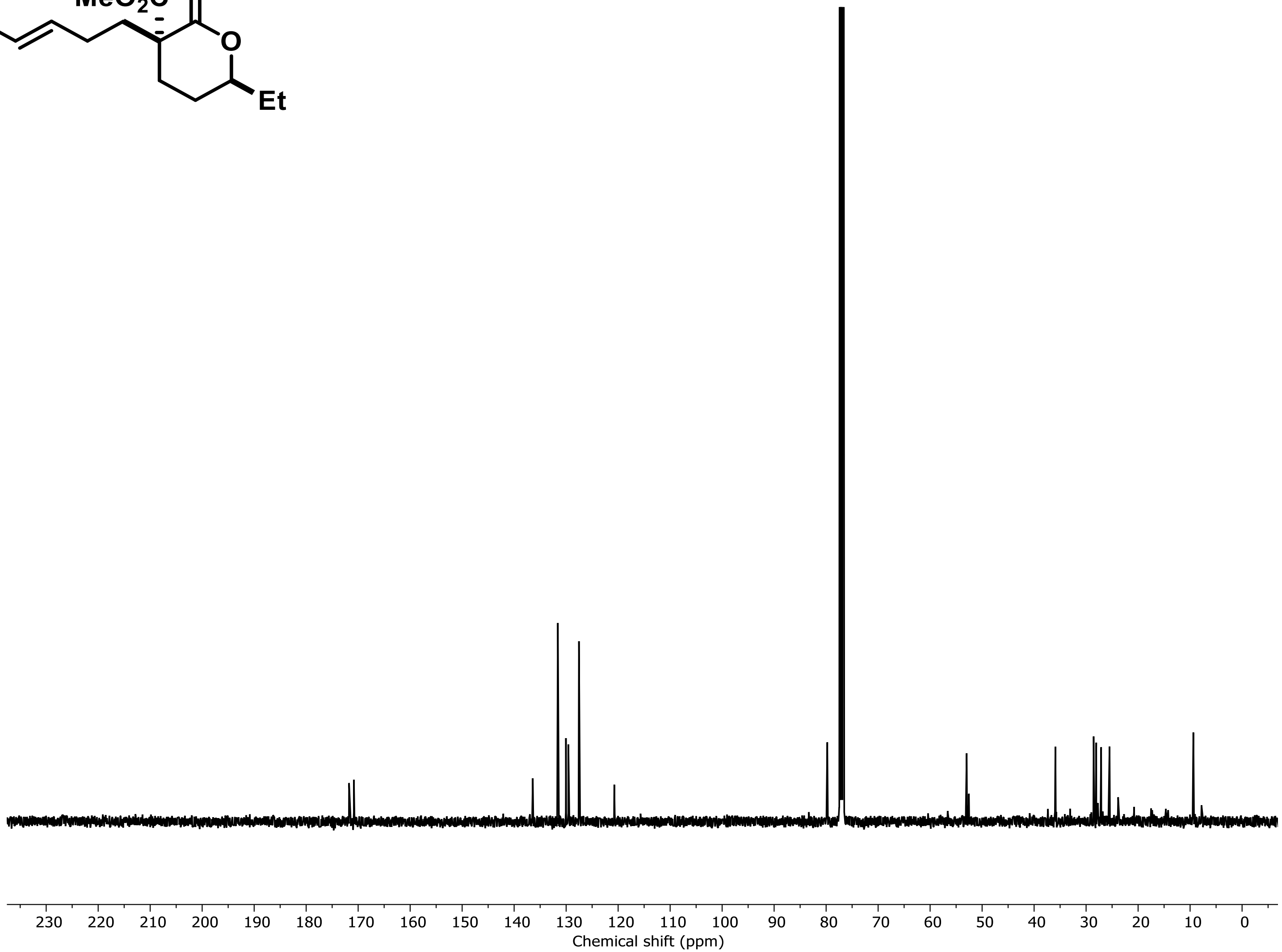

***Rac*–methyl (3*R*,6*S*)–6–ethyl–2–oxo–3–((*E*)–4–(4–(trifluoromethyl)phenyl)but–3–en–1–yl) tetrahydro–2*H*–pyran–3–carboxylate (1s)**

^{1}H NMR (400 MHz, $CDCl_3$)

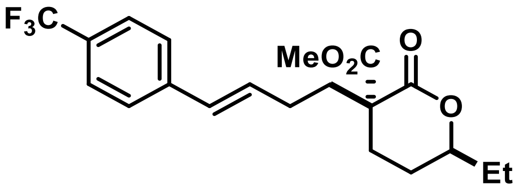

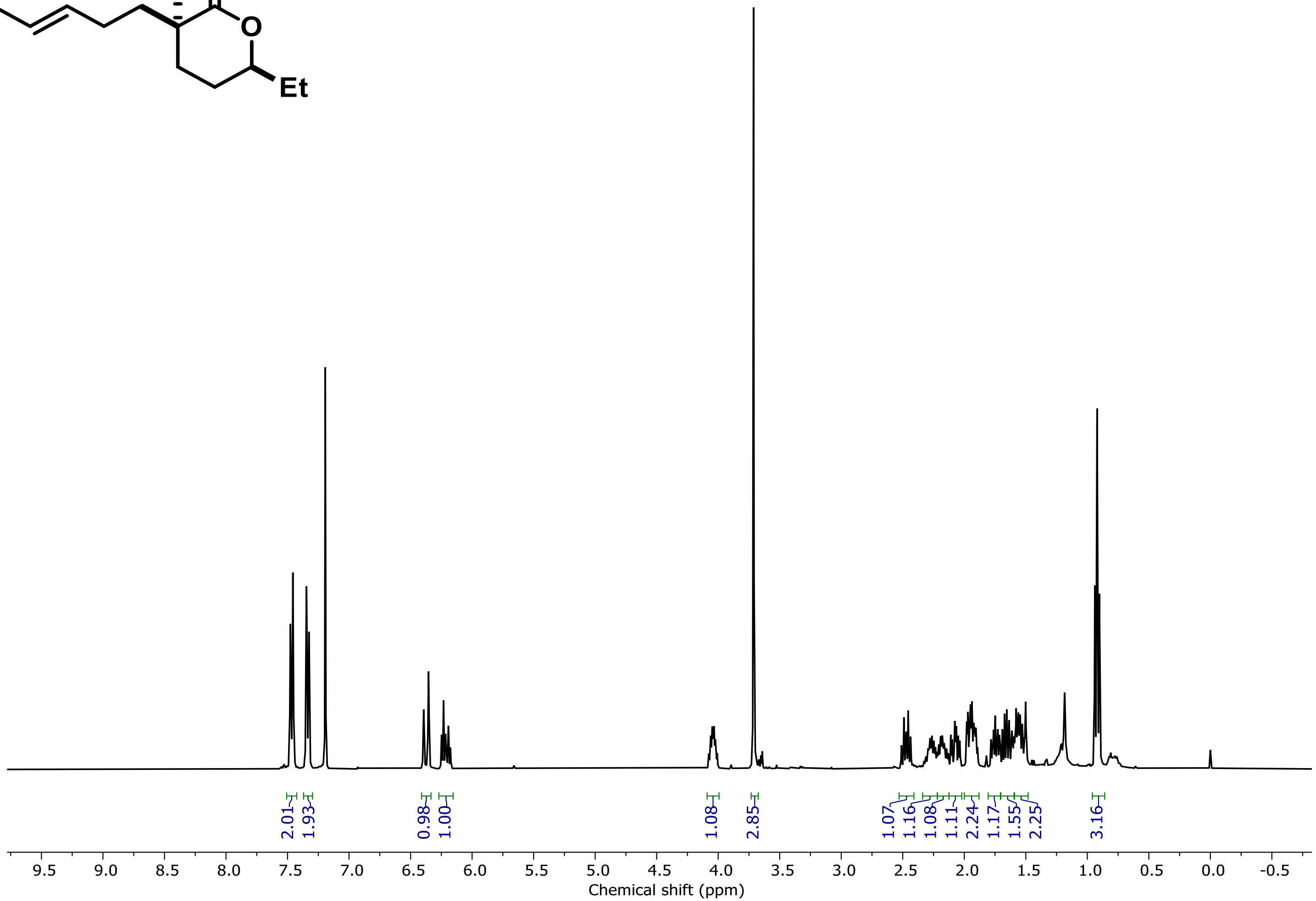

^{13}C NMR (101 MHz, $CDCl_3$)

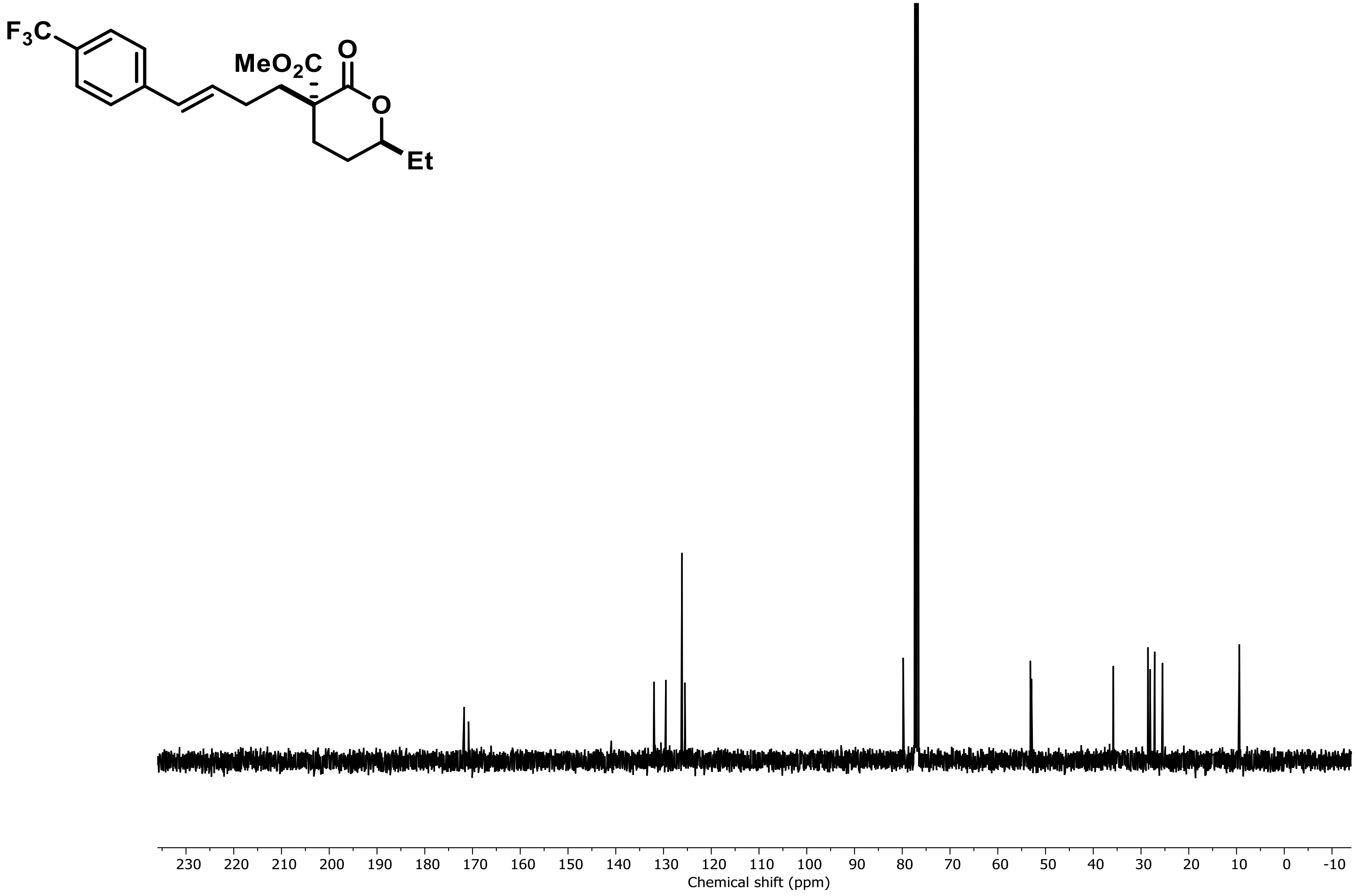

^{19}F NMR (471 MHz, $CDCl_3$)

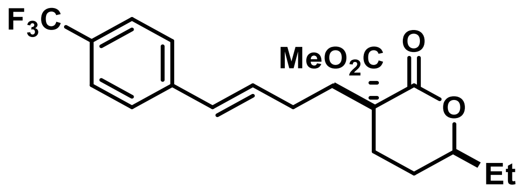

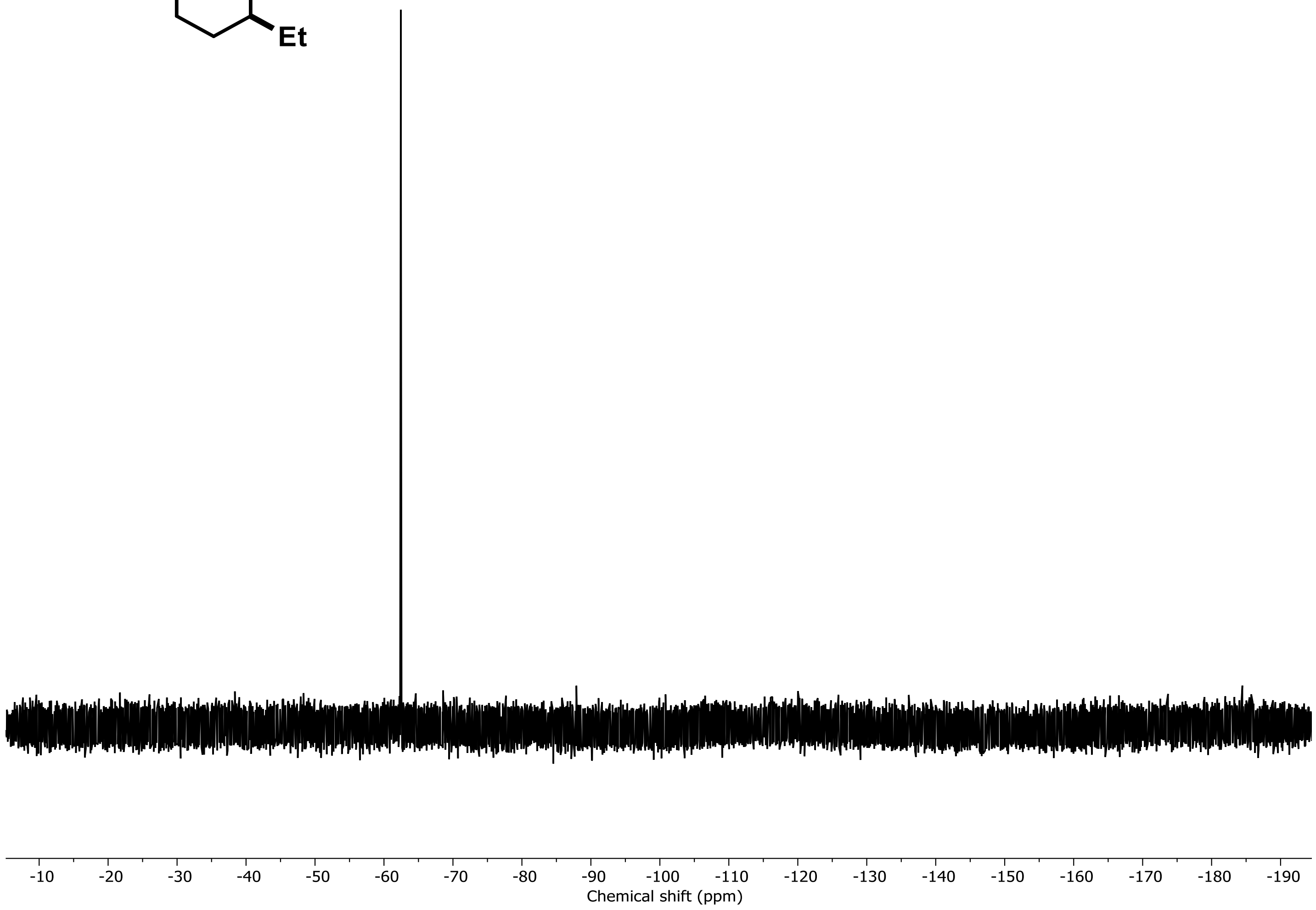

***Rac*–methyl (3*R*,6*S*)–6–ethyl–3–((*E*)–4–(4–methoxyphenyl)but–3–en–1–yl)–2–oxotetrahydro–2*H*–pyran–3–carboxylate (1t)**

^{1}H NMR (400 MHz, $CDCl_3$)

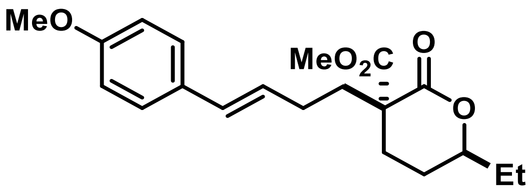

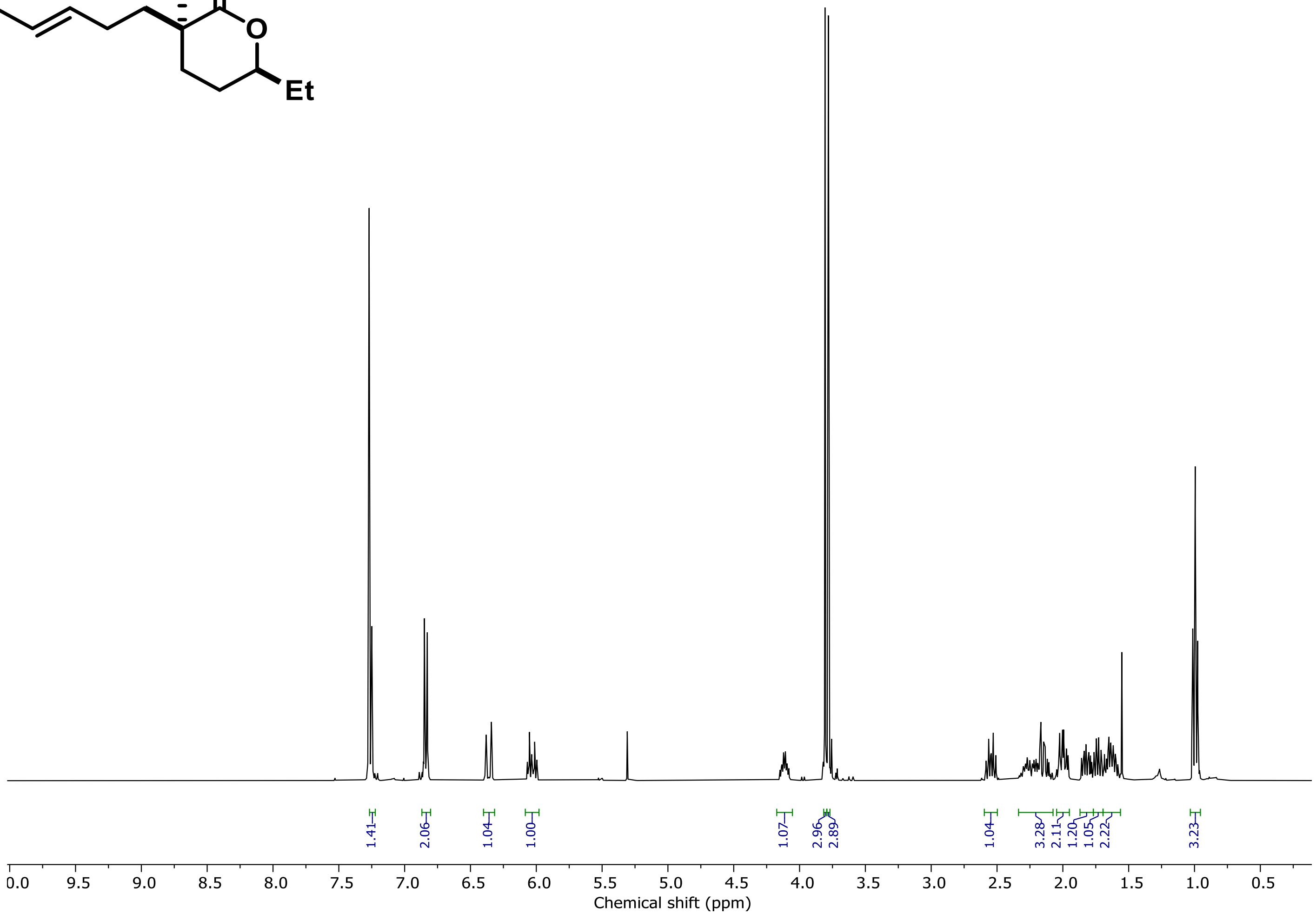

^{13}C NMR (101 MHz, $CDCl_3$)

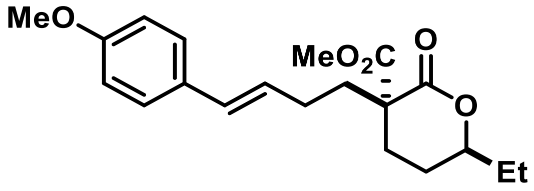

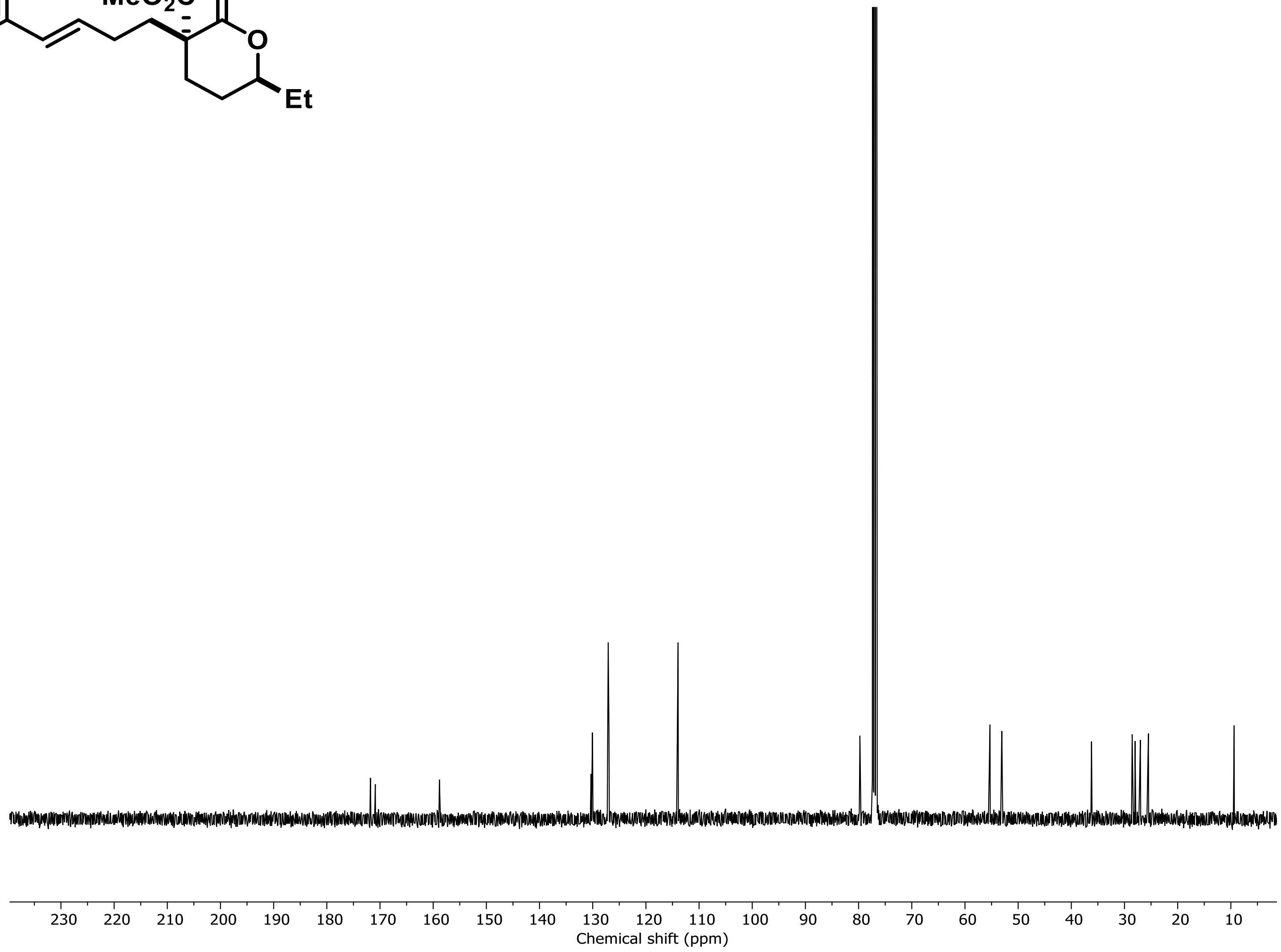

***Rac*-methyl (3*R*,6*S*)-3-(but-3-en-1-yl)-6-methyl-2-oxotetrahydro-2H-pyran-3-carboxylate (1w)**

^{1}H NMR (400 MHz, $CDCl_3$)

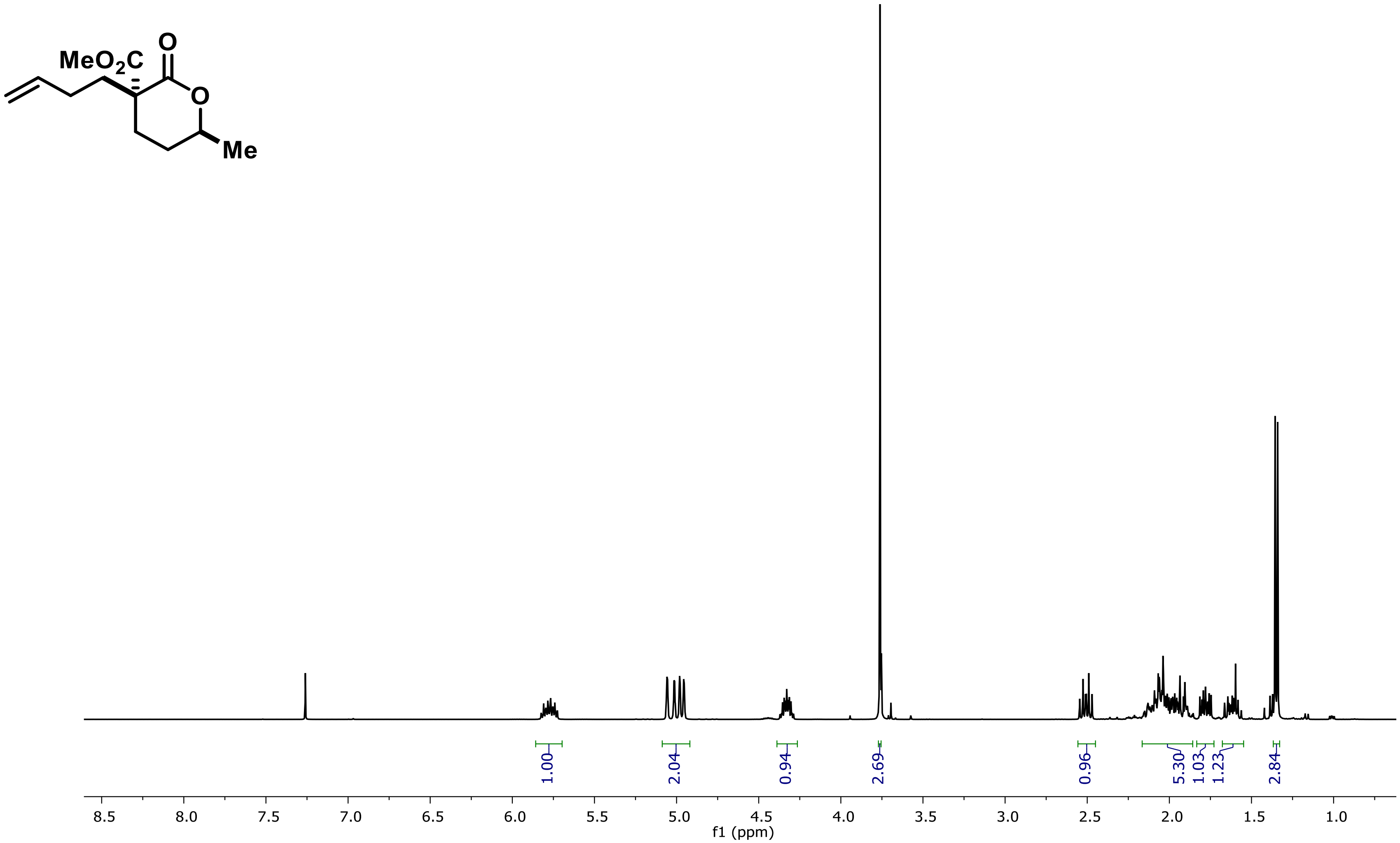

^{13}C NMR (101 MHz, $CDCl_3$)

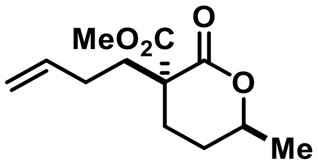

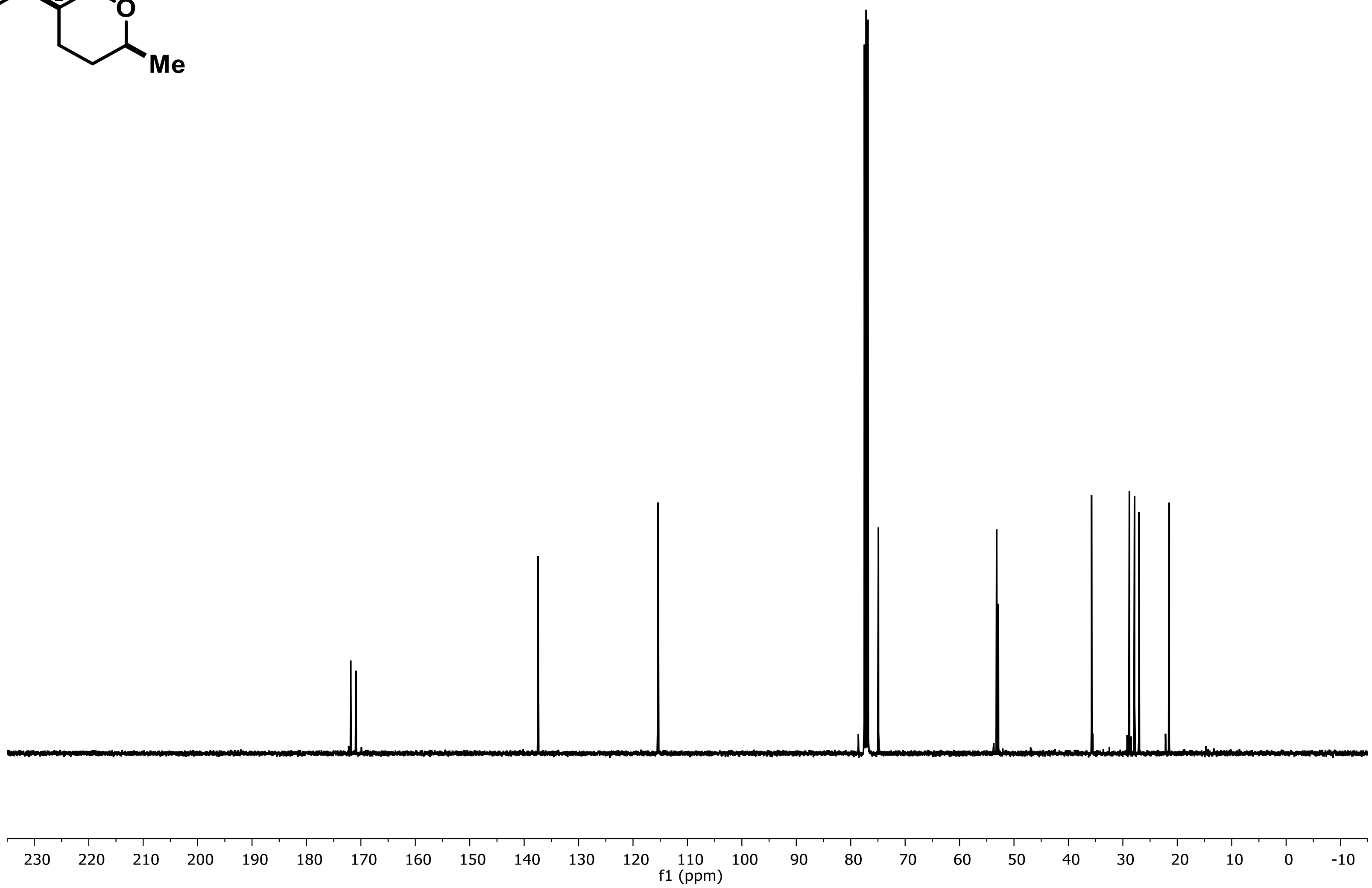

***Rac*-methyl (3*S*,6*S*)-6-methyl-2-oxo-3-(3-oxopropyl)tetrahydro-2H-pyran-3-carboxylate (S33)**

^{1}H NMR (400 MHz, $CDCl_3$)

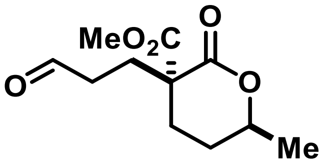

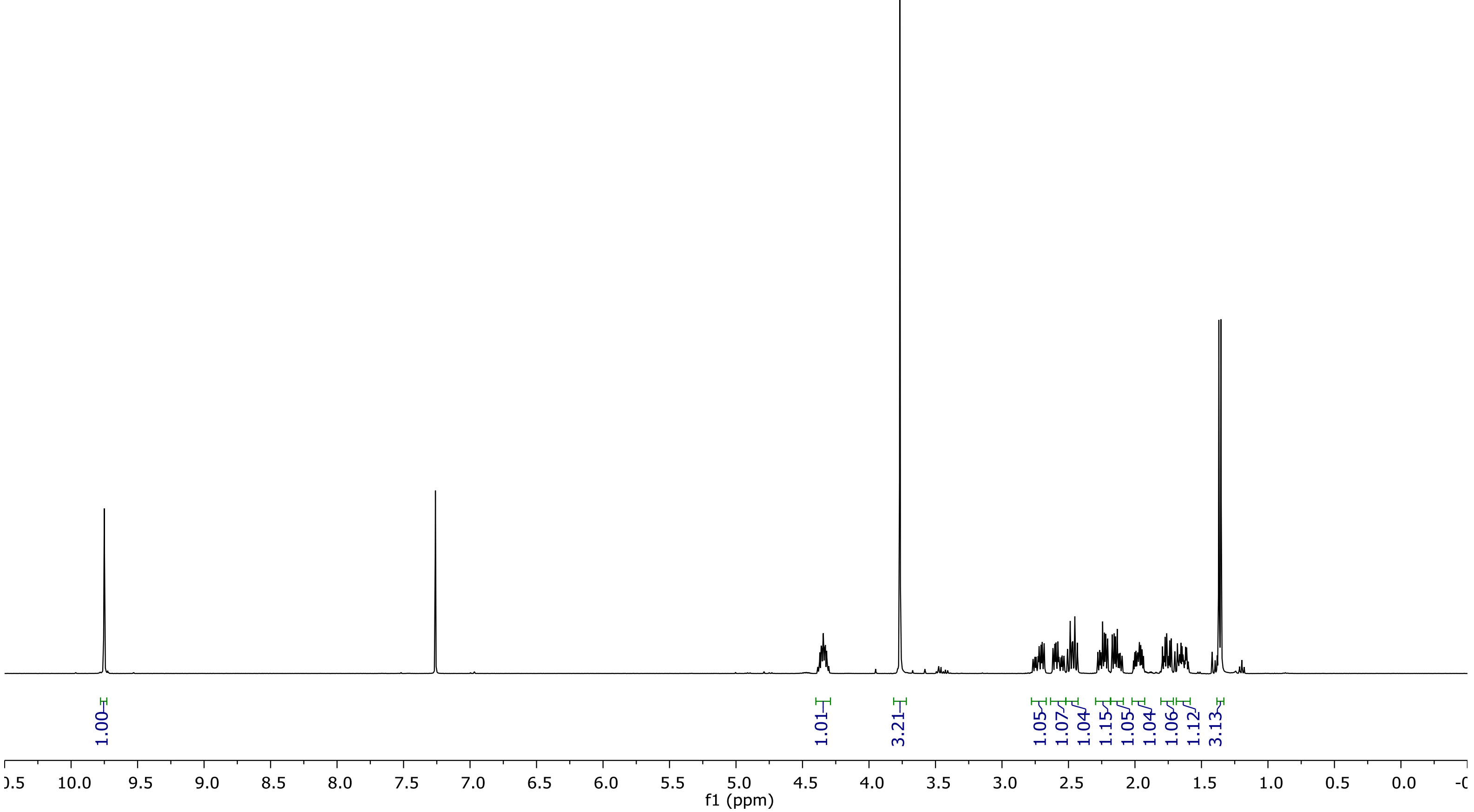

^{13}C NMR (101 MHz, $CDCl_3$)

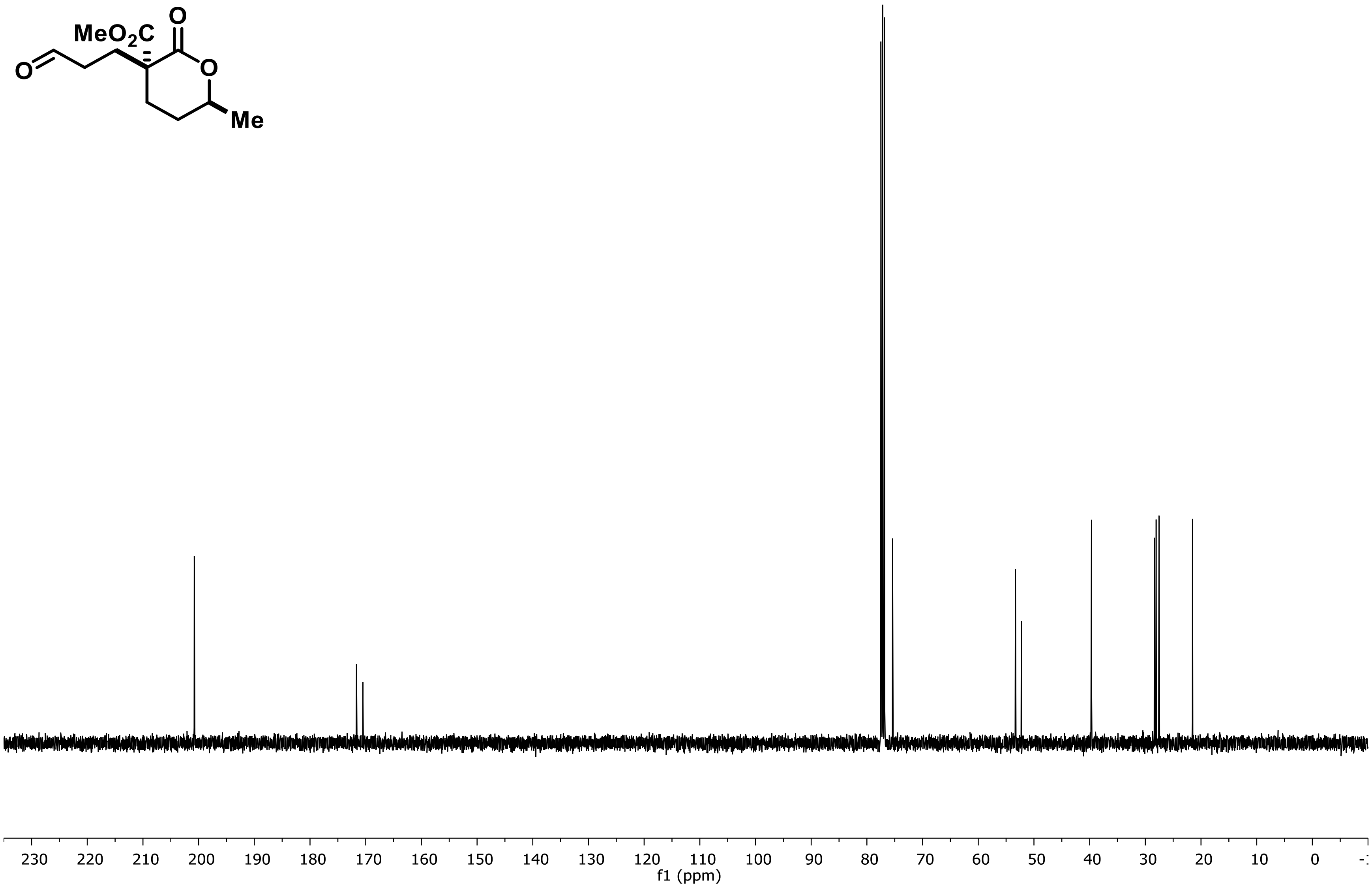

***Rac*-methyl (3R,6S)-6-methyl-2-oxo-3-(4-(thiophen-2-yl)but-3-en-1-yl)tetrahydro-2H-pyran-3-carboxylate (1u)** 3.9:1 mixture of diastereomers with 1.4:1 ratio of *E*:*Z* isomers

^{1}H NMR (400 MHz, $CDCl_3$)

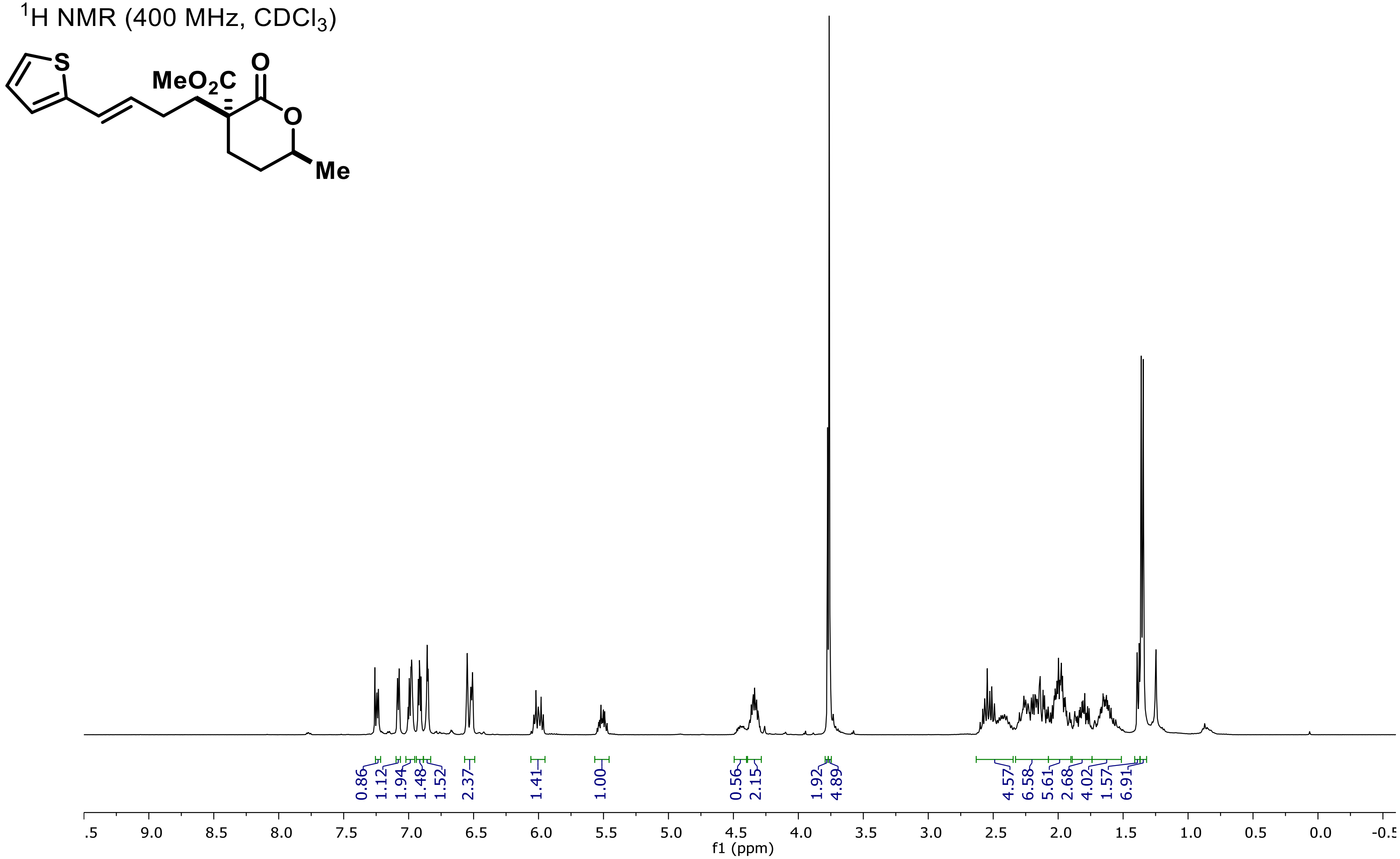

^{13}C NMR (101 MHz, $CDCl_3$)

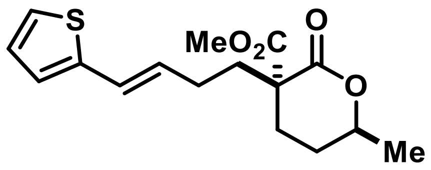

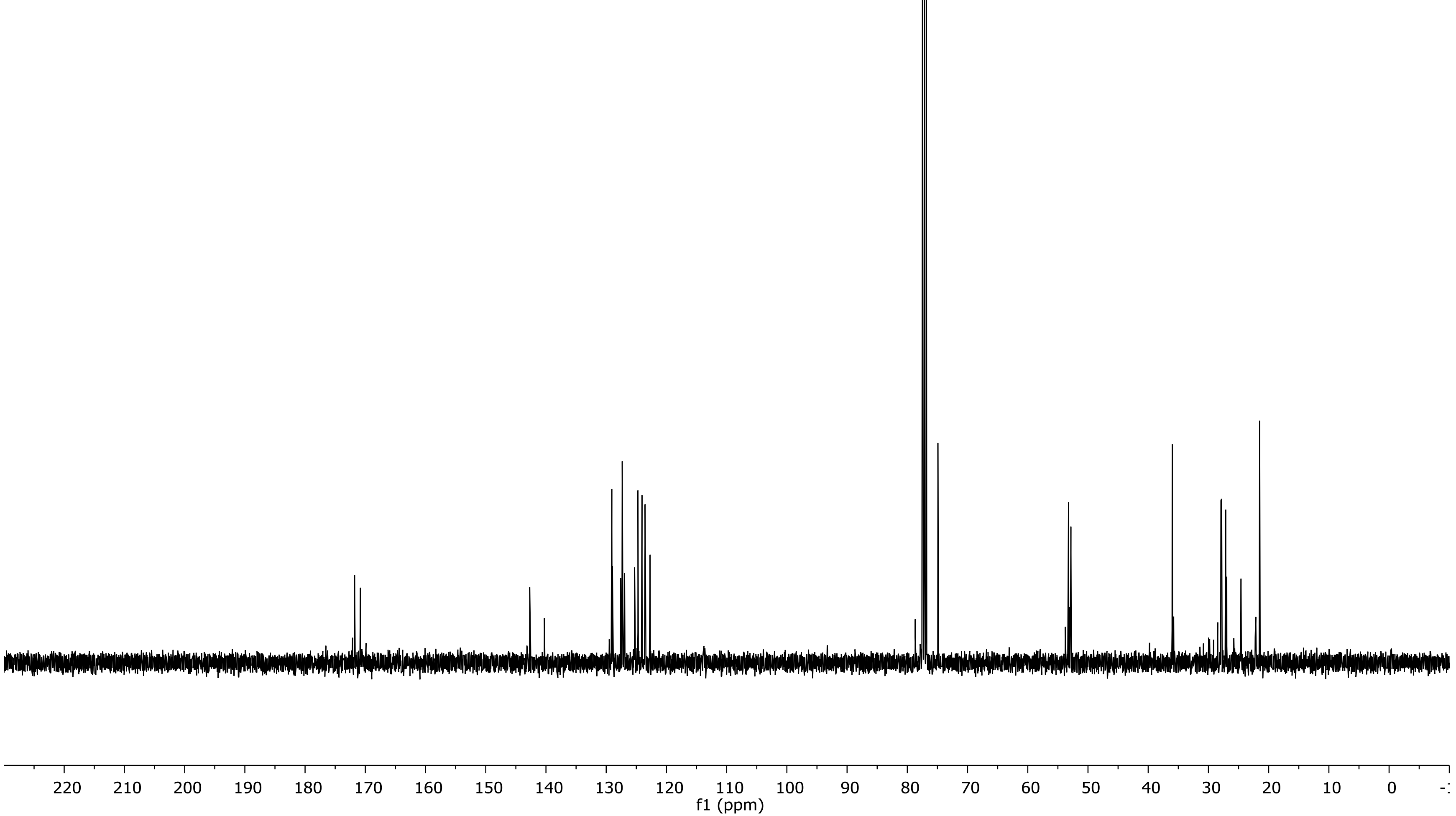

***Rac*-methyl (3*R*,6*S*)-6-methyl-2-oxo-3-(4-phenylpent-3-en-1-yl)tetrahydro-2H-pyran-3-carboxylate (1v)** mixture of diasteromers and alkene isomers

^{1}H NMR (400 MHz, $CDCl_3$)

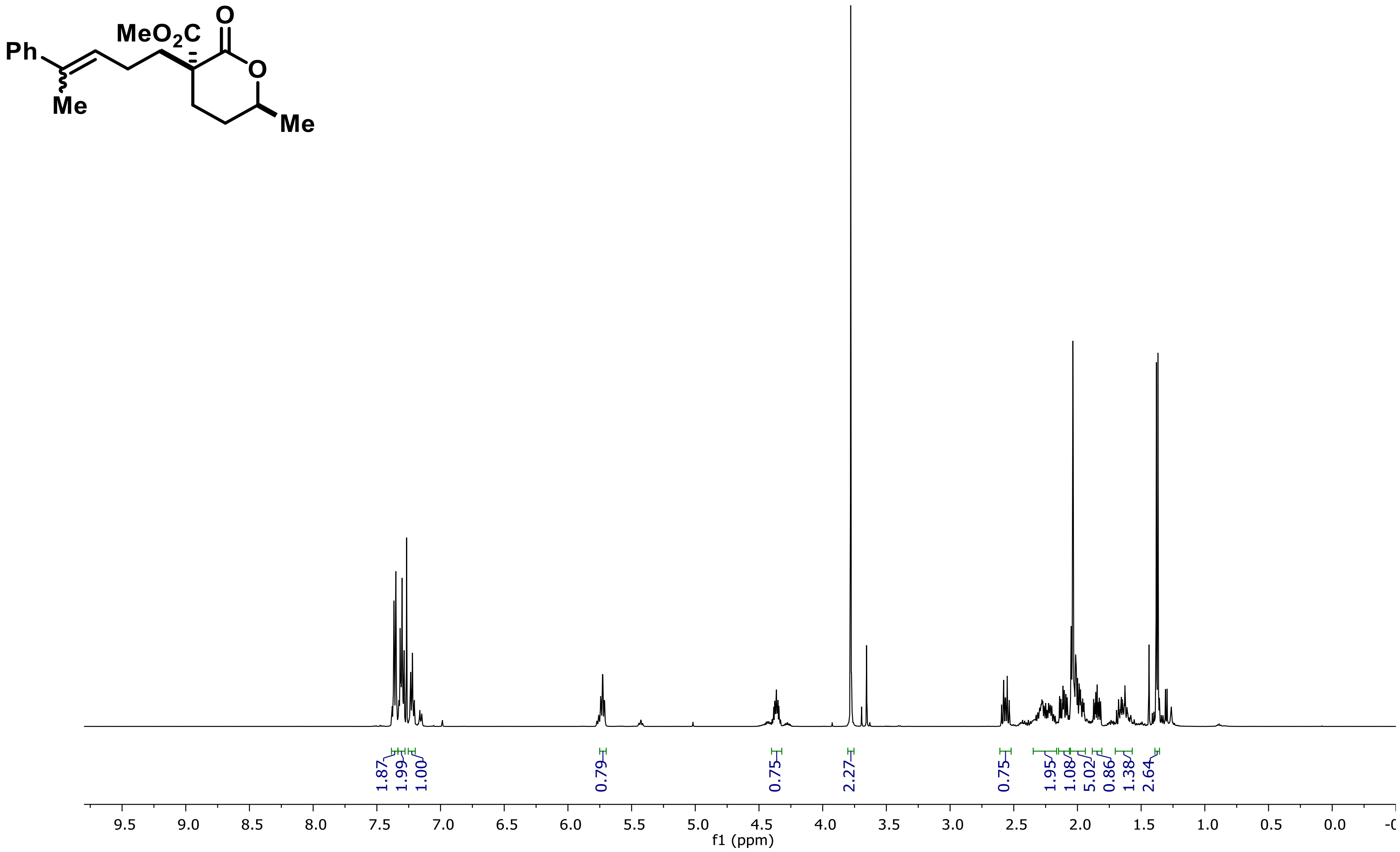

^{13}C NMR (126 MHz, $CDCl_3$)

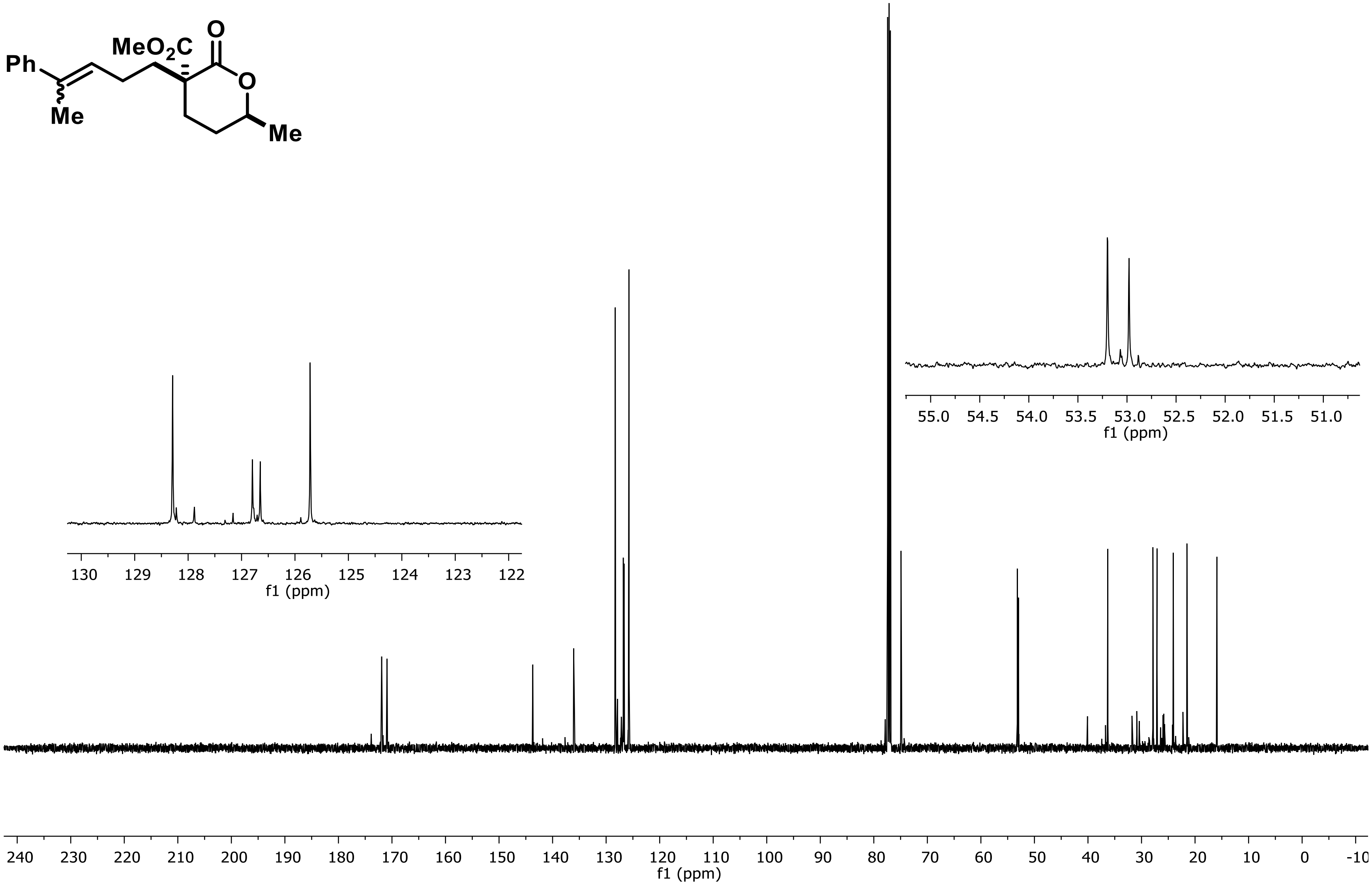

***Rac*-methyl (3*R*,6*S*)-6-methyl-3-(4-methylpent-3-en-1-yl)-2-oxotetrahydro-2H-pyran-3-carboxylate (1x)** 10:1 d.r.

^{1}H NMR (400 MHz, $CDCl_3$)

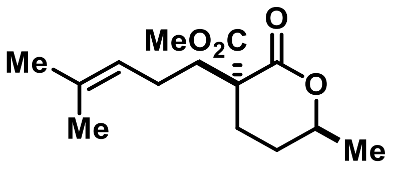

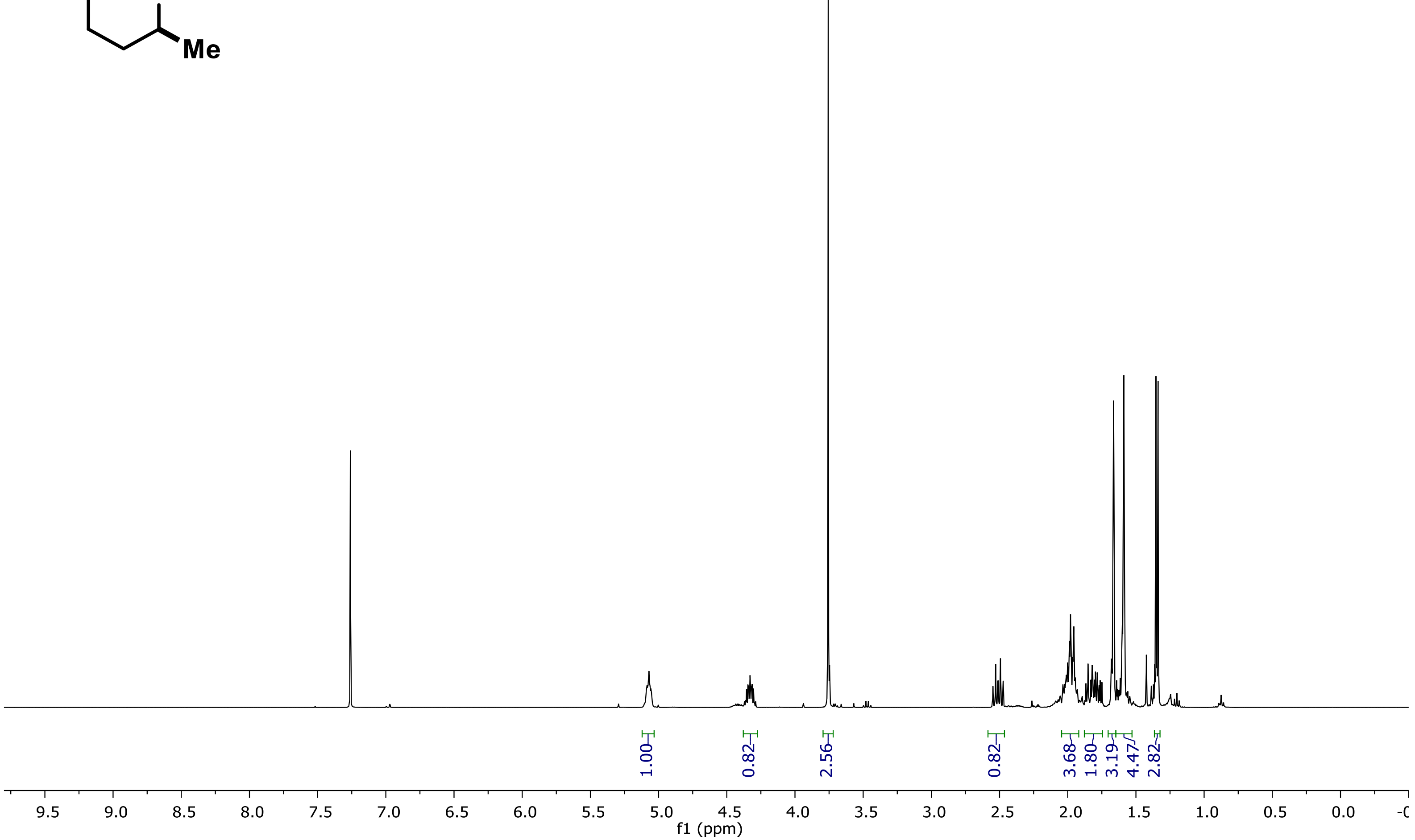

^{13}C NMR (101 MHz, $CDCl_3$)

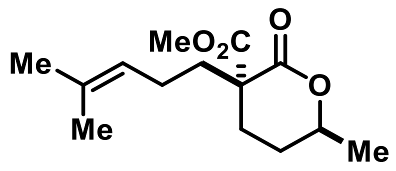

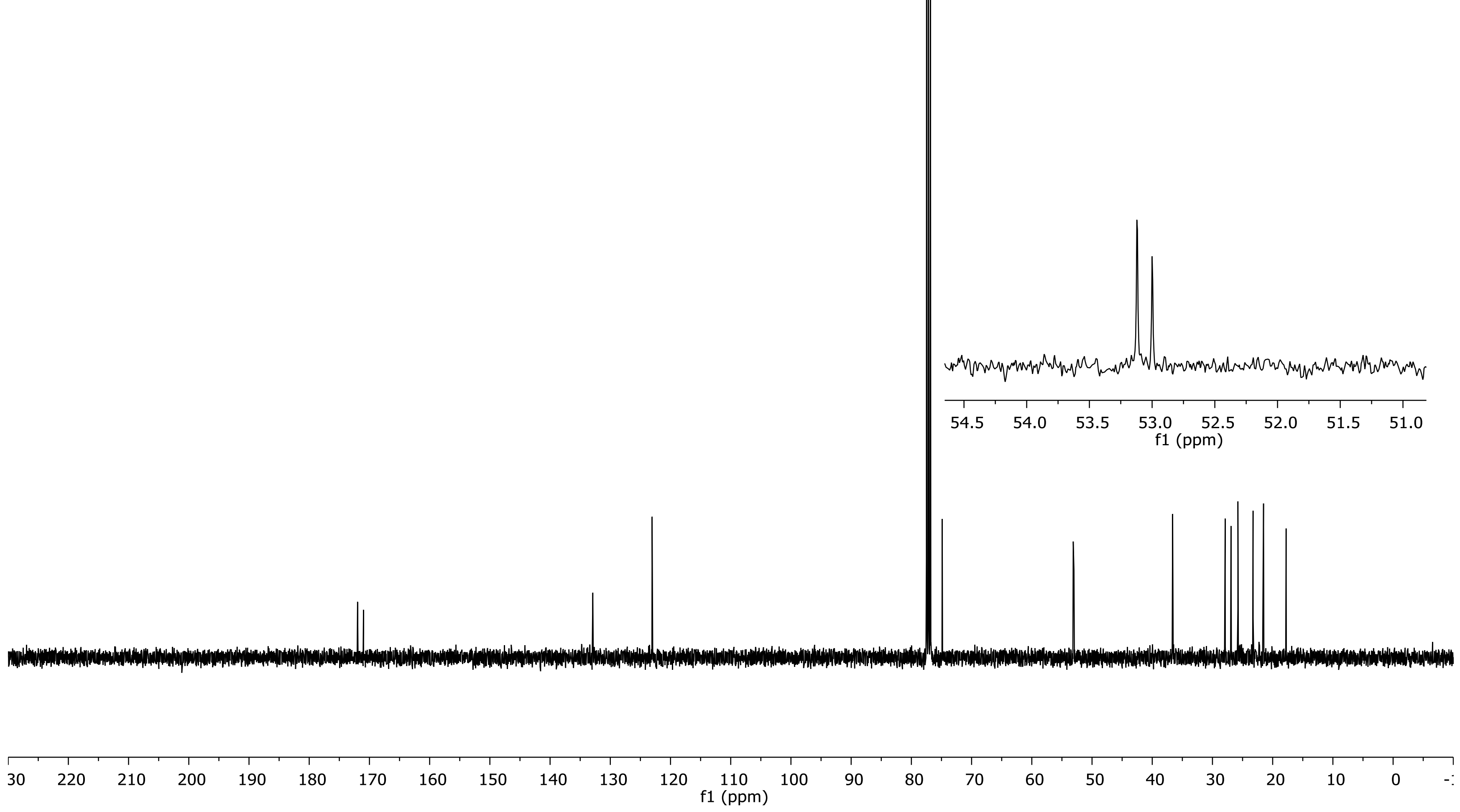

Dimethyl 2-(3-methylbut-3-en-1-yl)-2-(3-oxobutyl)malonate (S34)

^{1}H NMR (400 MHz, $CDCl_3$)

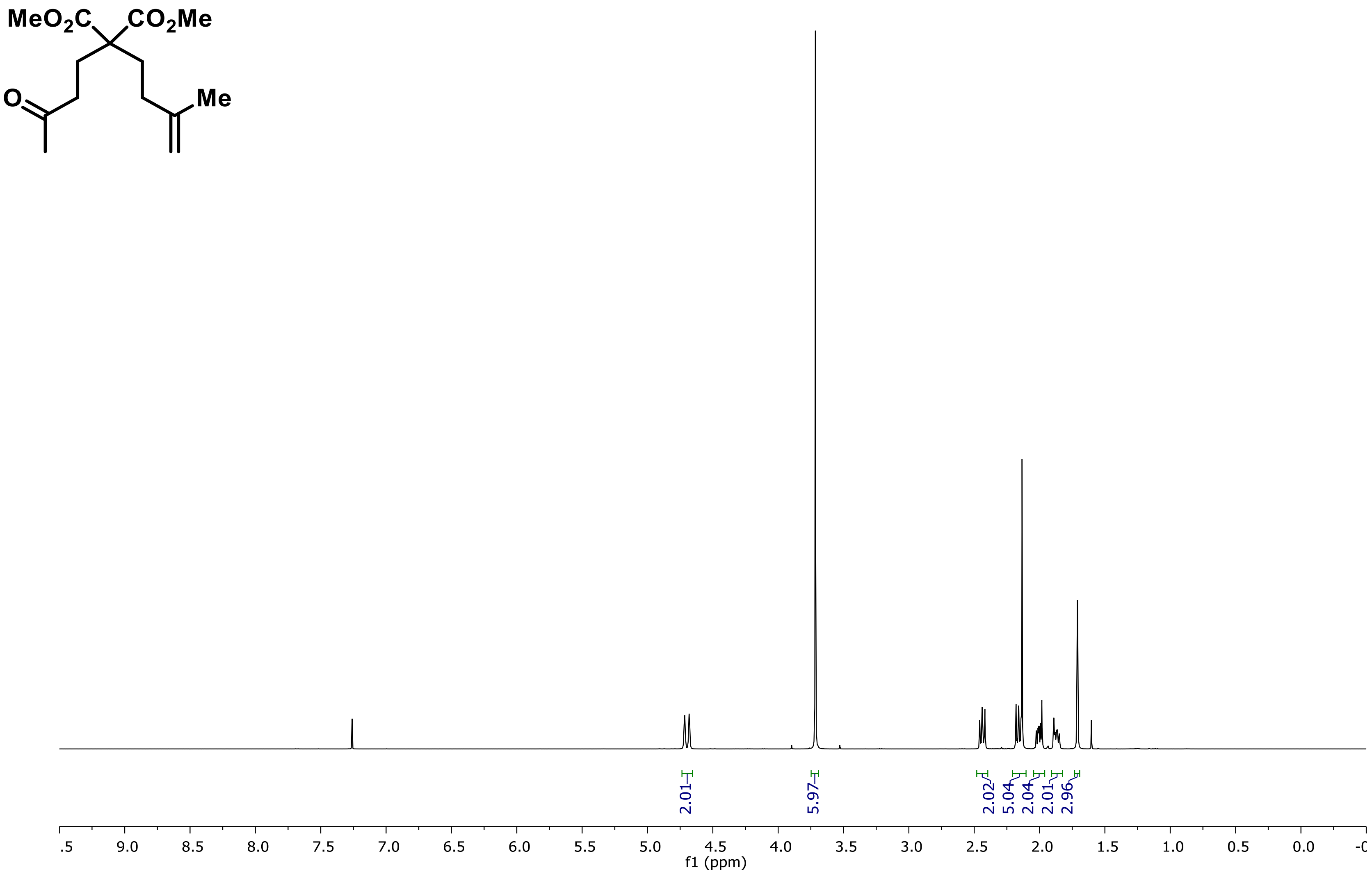

^{13}C NMR (101 MHz, $CDCl_3$)

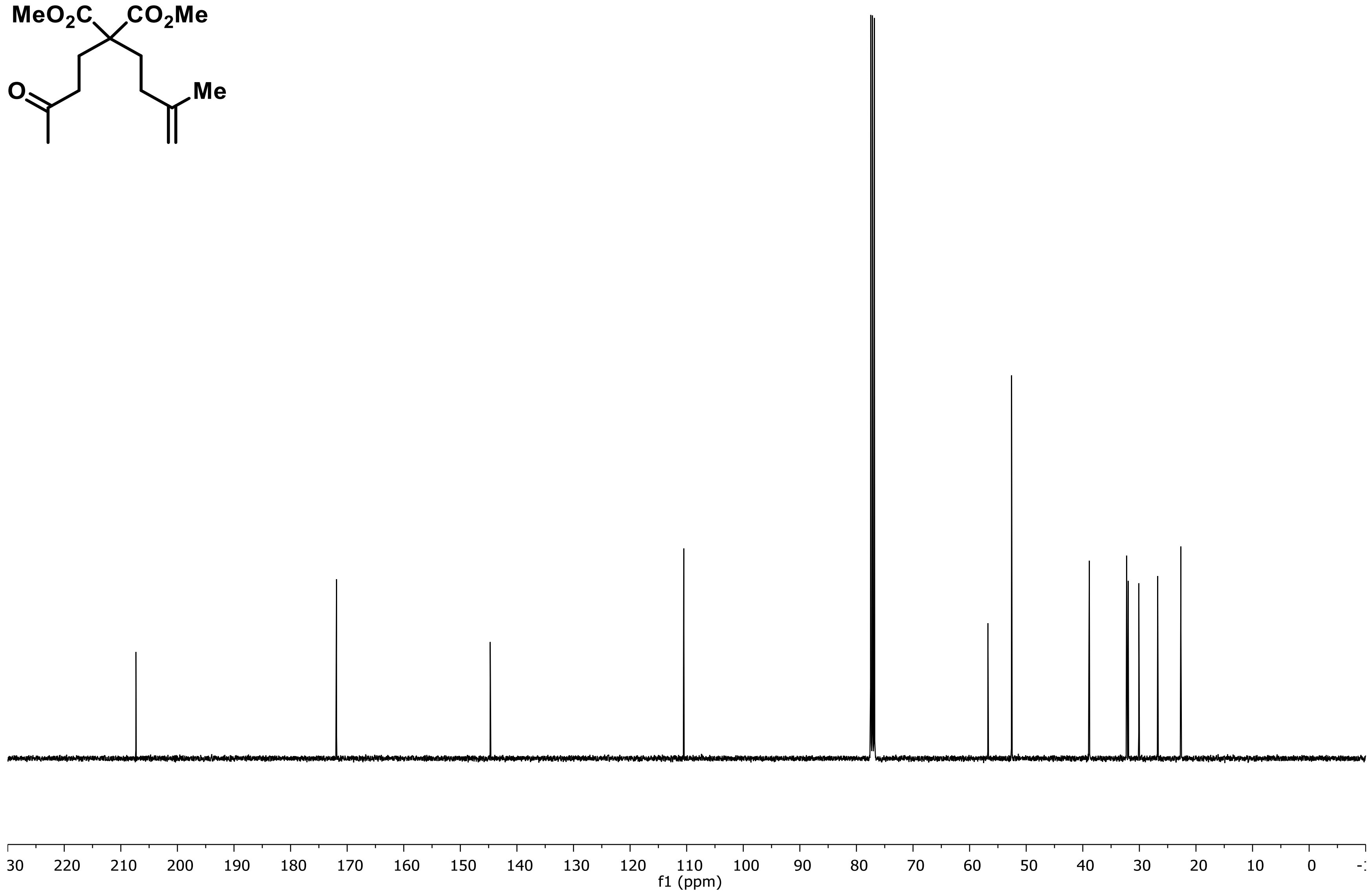

***Rac*-methyl (3*R*,6*S*)-6-methyl-3-(3-methylbut-3-en-1-yl)-2-oxotetrahydro-2H-pyran-3-carboxylate (S35)** 6.5:1 d.r.

^{1}H NMR (400 MHz, $CDCl_3$)

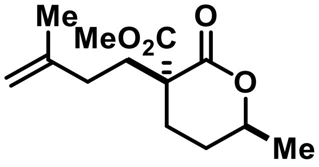

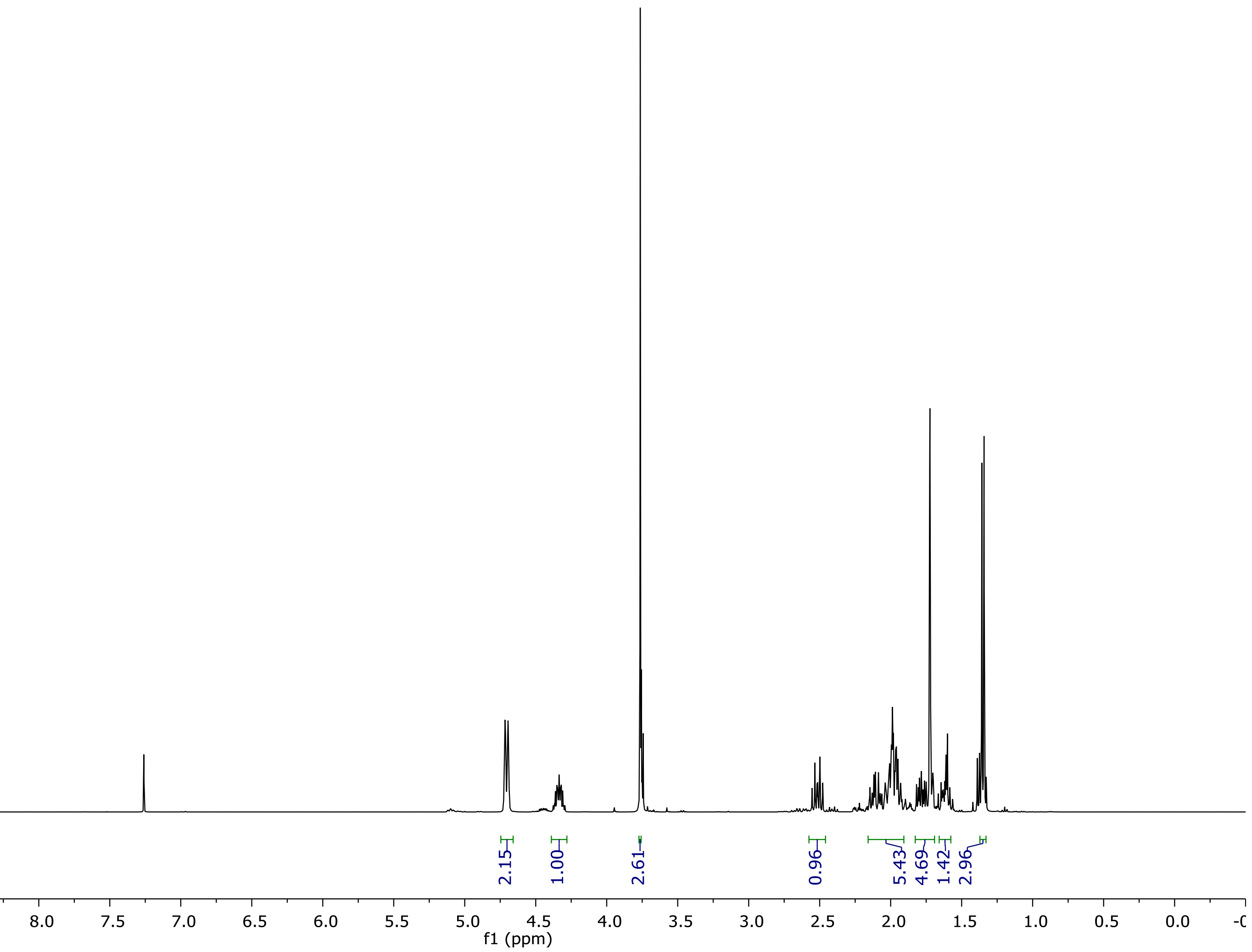

^{13}C NMR (101 MHz, $CDCl_3$)

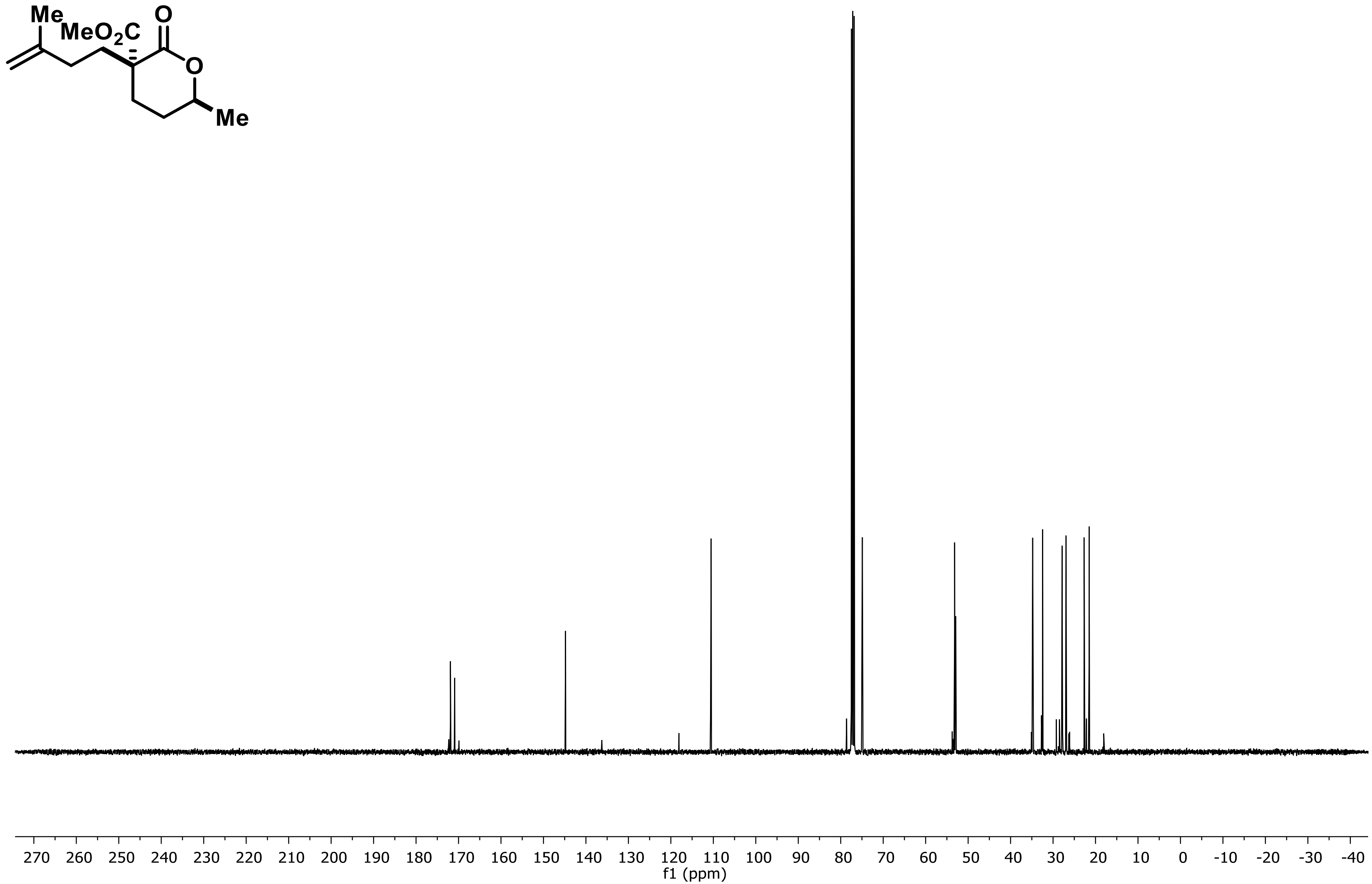

Dimethyl 2-(3-oxobutyl)-2-(4-phenylbut-3-yn-1-yl)malonate (S36)

^{1}H NMR (500 MHz, $CDCl_3$)

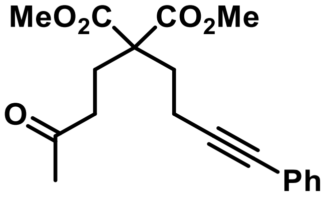

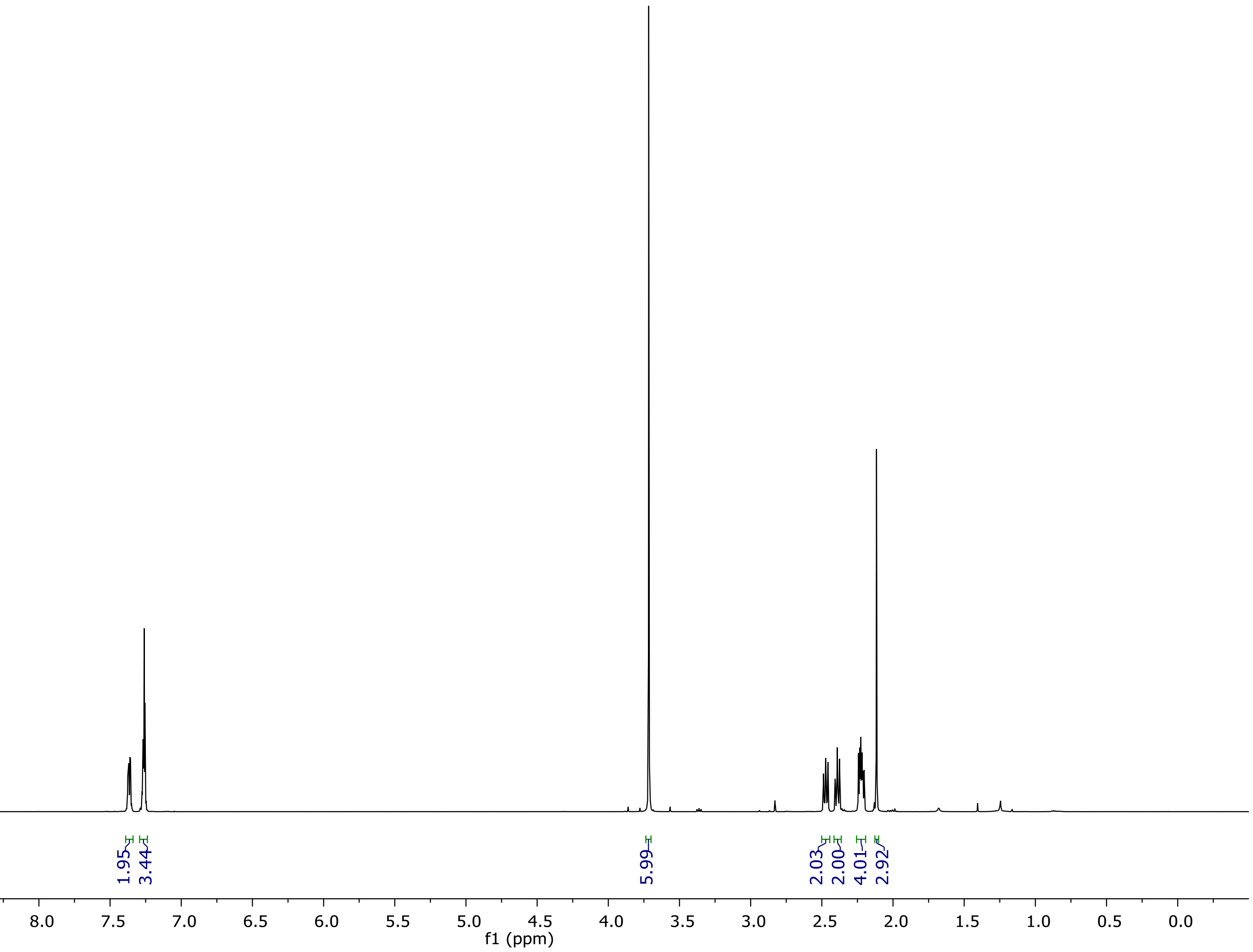

^{13}C NMR (126 MHz, $CDCl_3$)

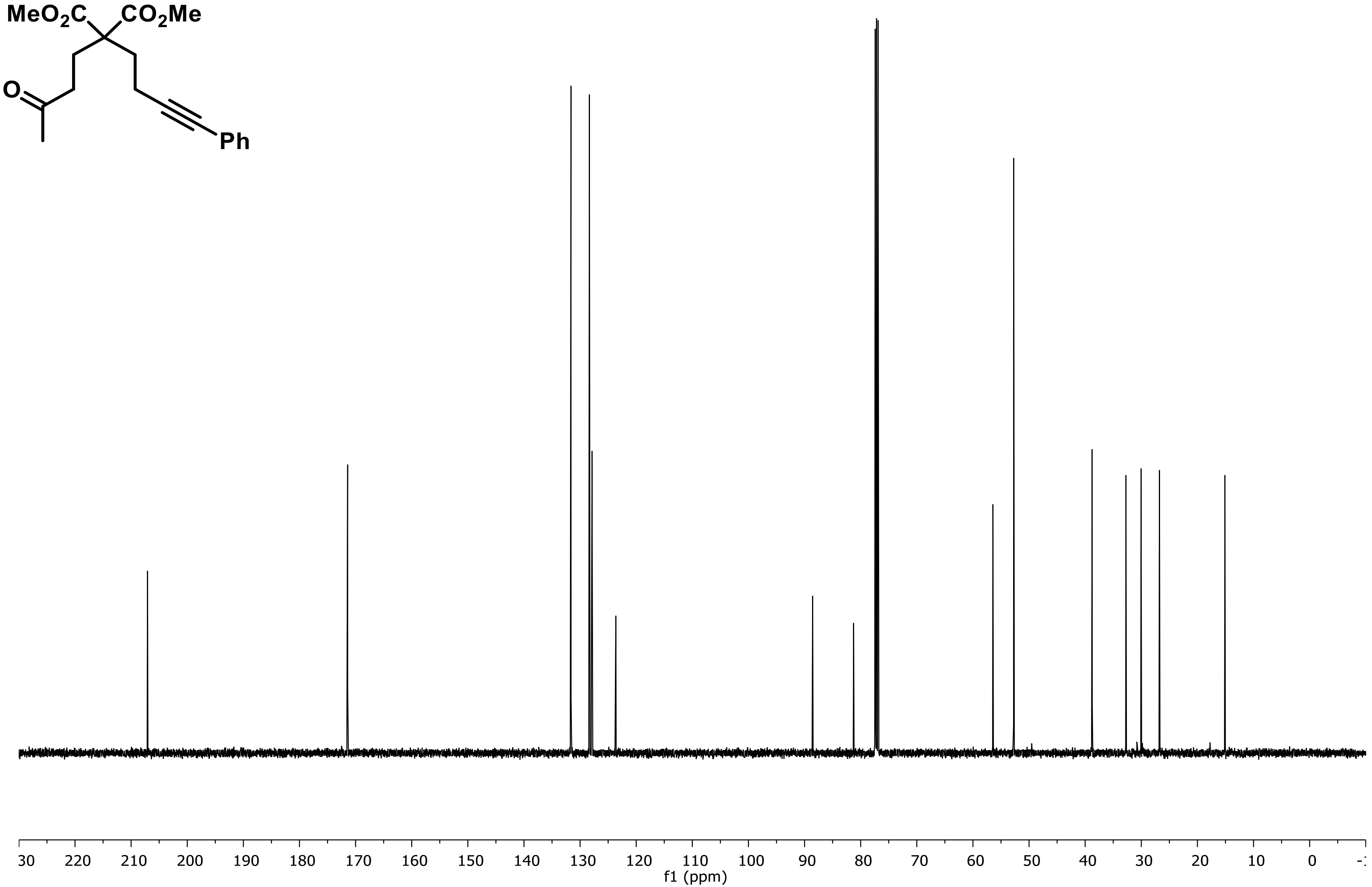

***Rac*-methyl (3*R*,6*S*)-6-methyl-2-oxo-3-(4-phenylbut-3-yn-1-yl)tetrahydro-2H-pyran-3-carboxylate (1y)**

^{1}H NMR (400 MHz, $CDCl_3$)

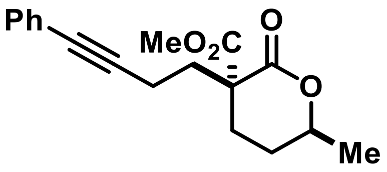

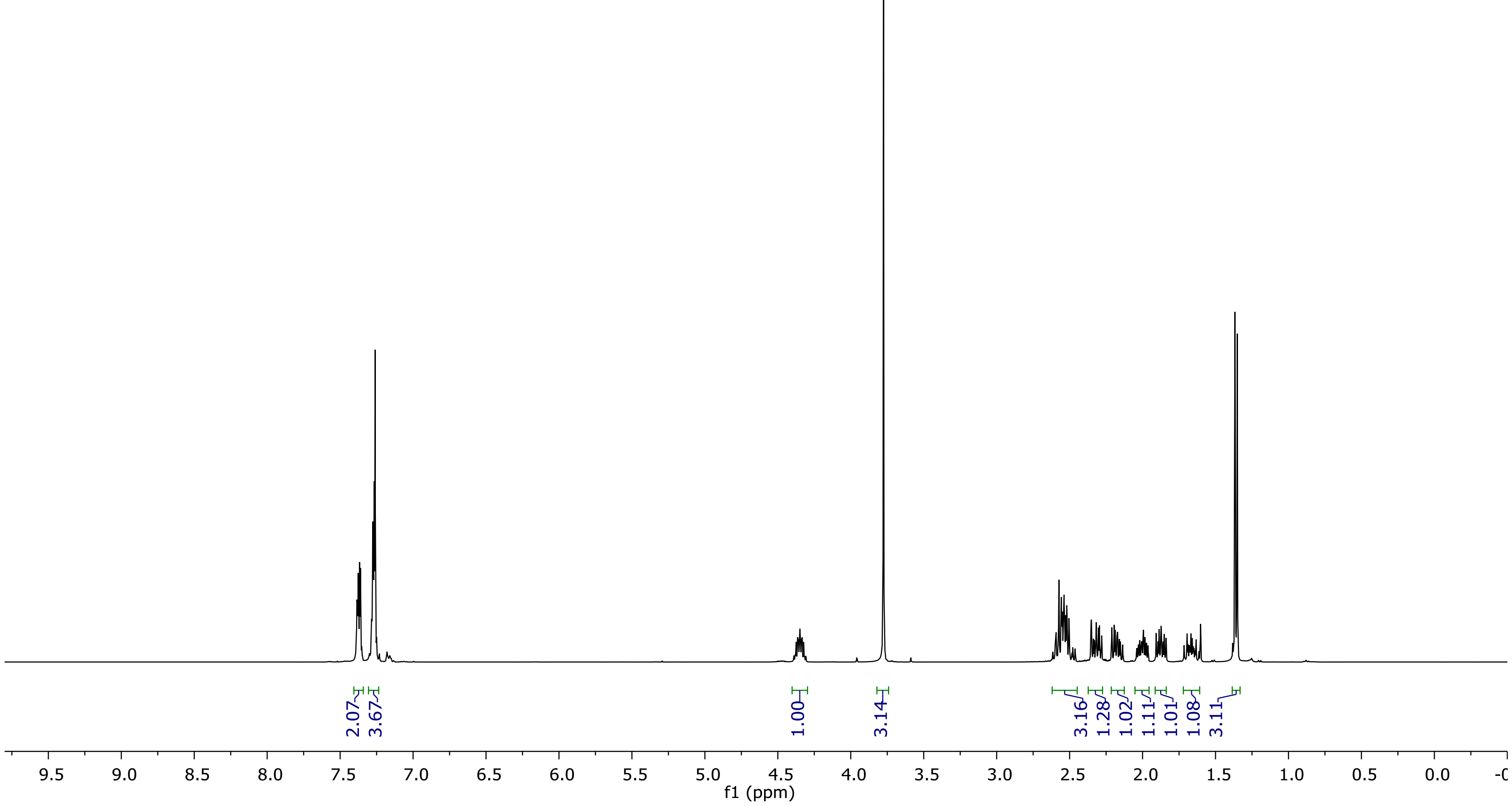

^{13}C NMR (101 MHz, $CDCl_3$)

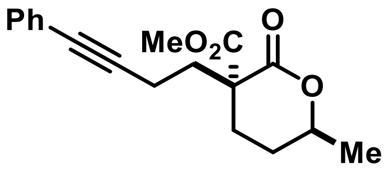

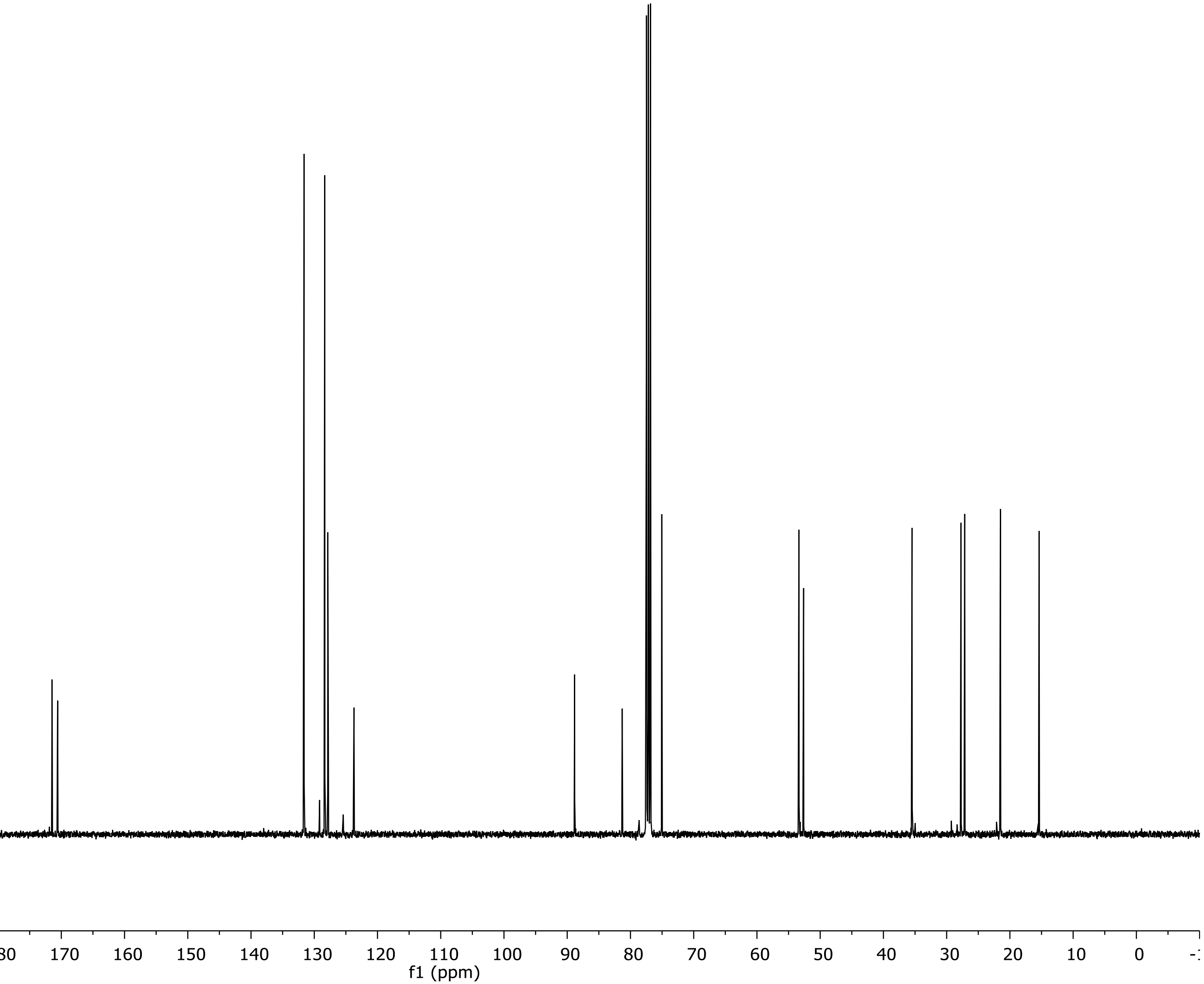

***Rac*–methyl (*R*)–2–benzyl–4–((3*S*,6*S*)–6–methyl–2–oxotetrahydro–2*H*–pyran–3–yl)butanoate (3a)**

^{1}H NMR (400 MHz, $CDCl_3$)

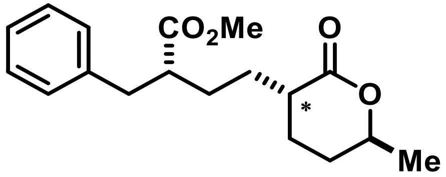

3:1 d.r.

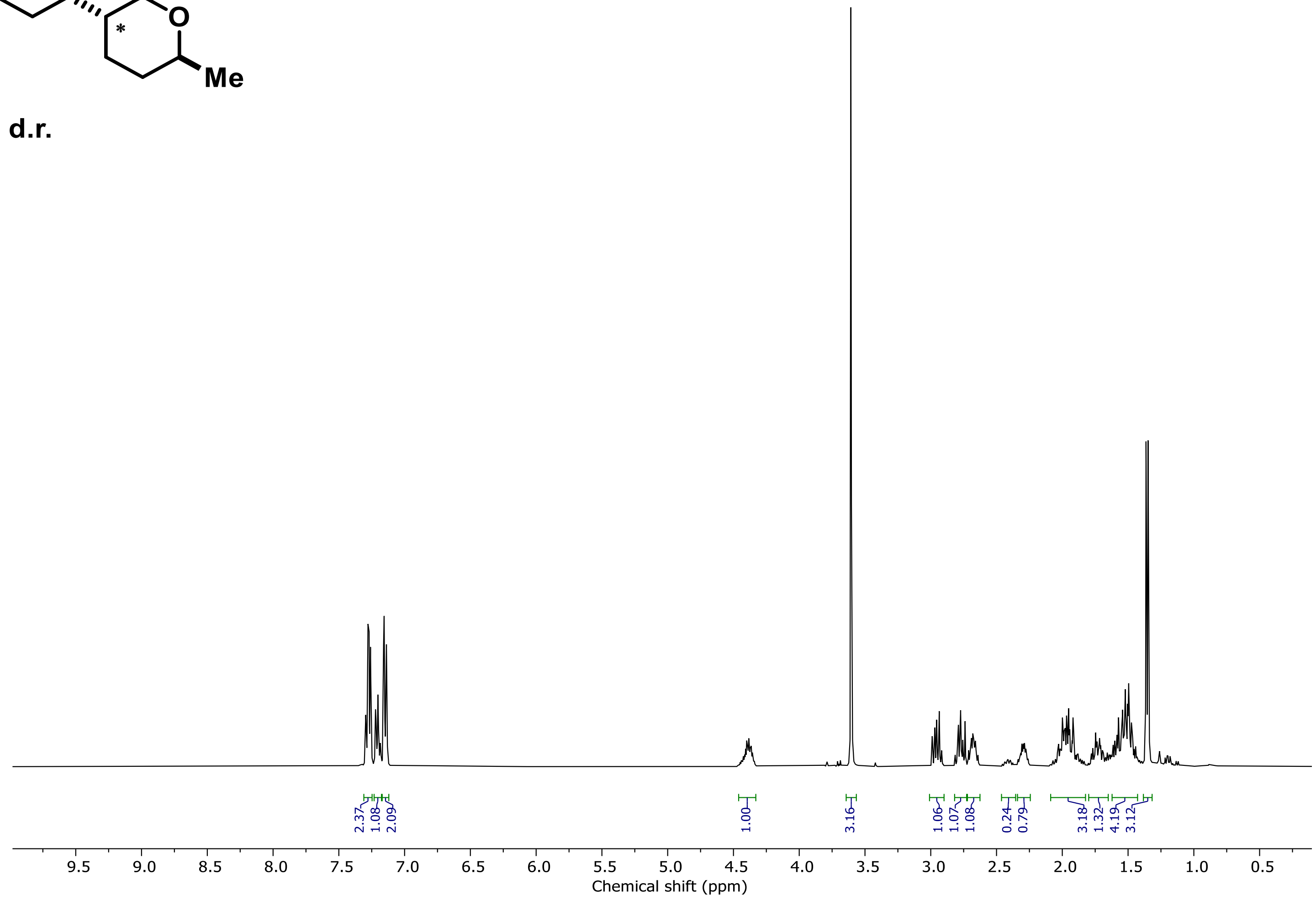

^{13}C NMR (101 MHz, $CDCl_3$)

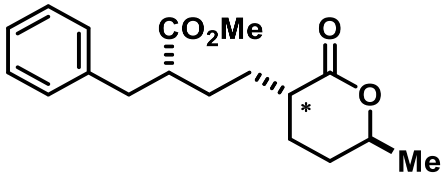

3:1 d.r.

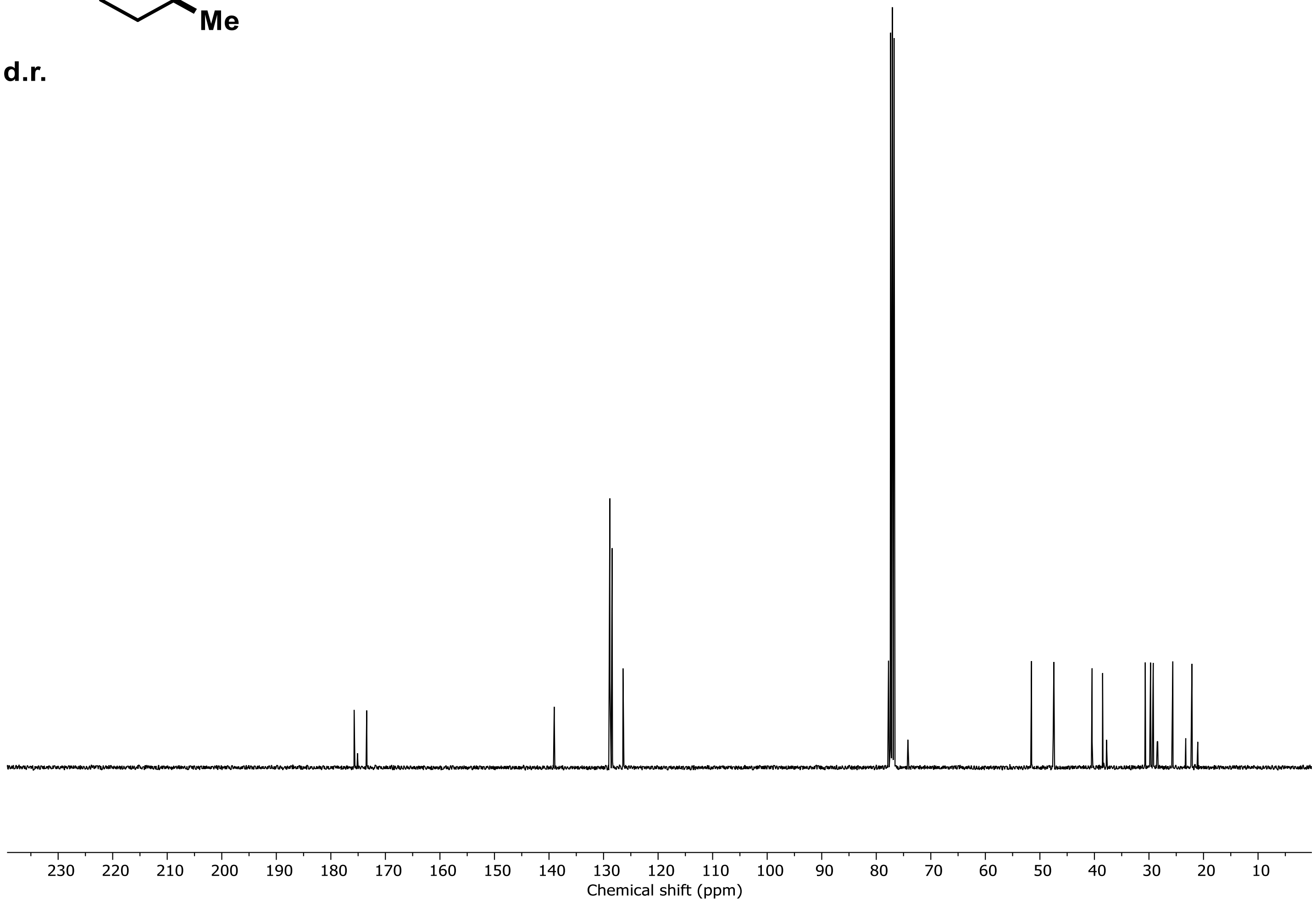

***Rac*–methyl (1*S*,3*S*)–3–benzyl–1–(3–hydroxybutyl)–2–oxocyclopentane–1–carboxylate (2a)**

^{1}H NMR (500 MHz, $CDCl_3$)

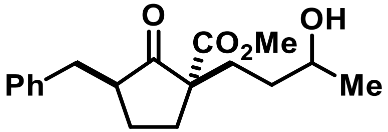

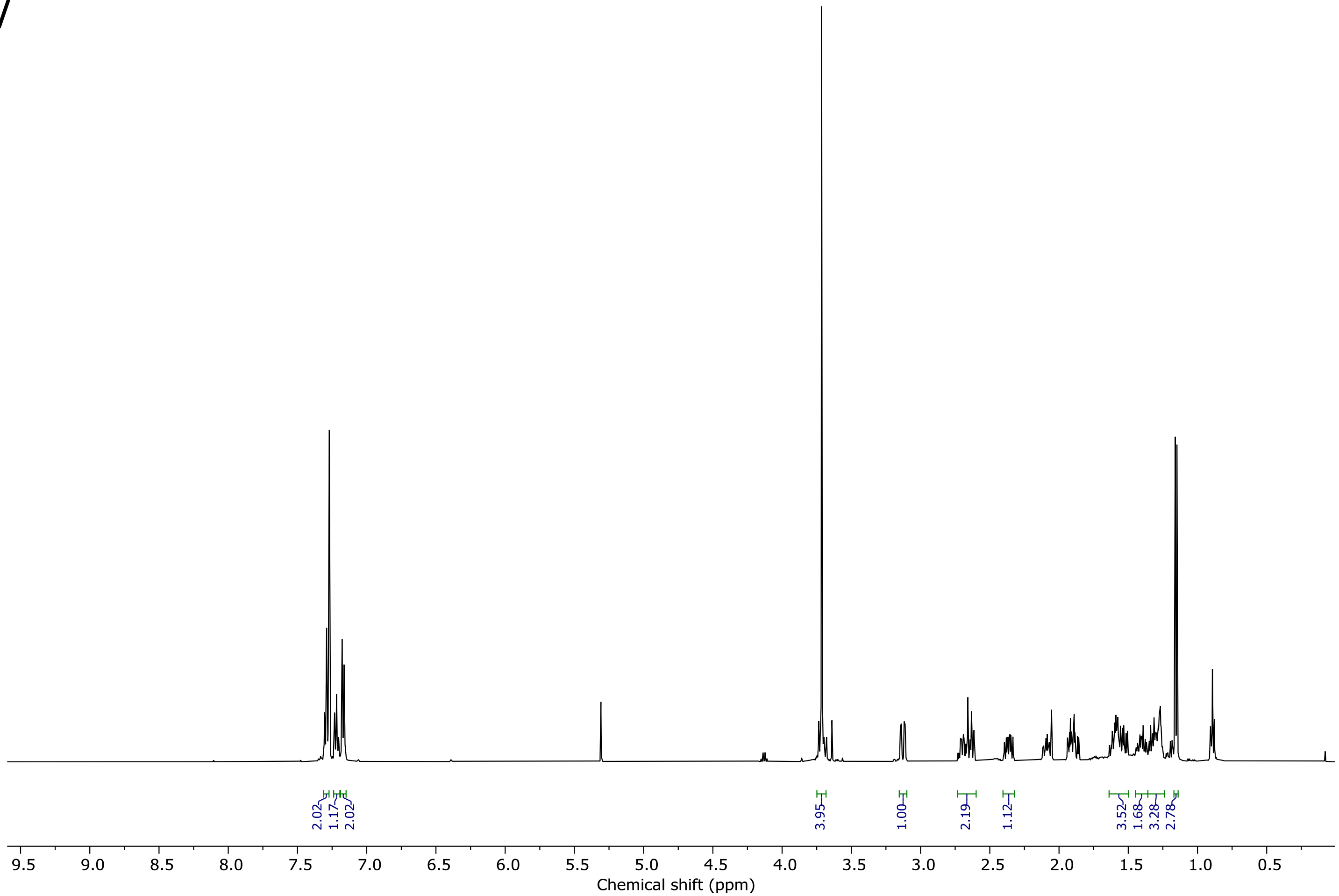

^{13}C NMR (126 MHz, $CDCl_3$)

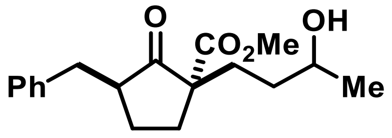

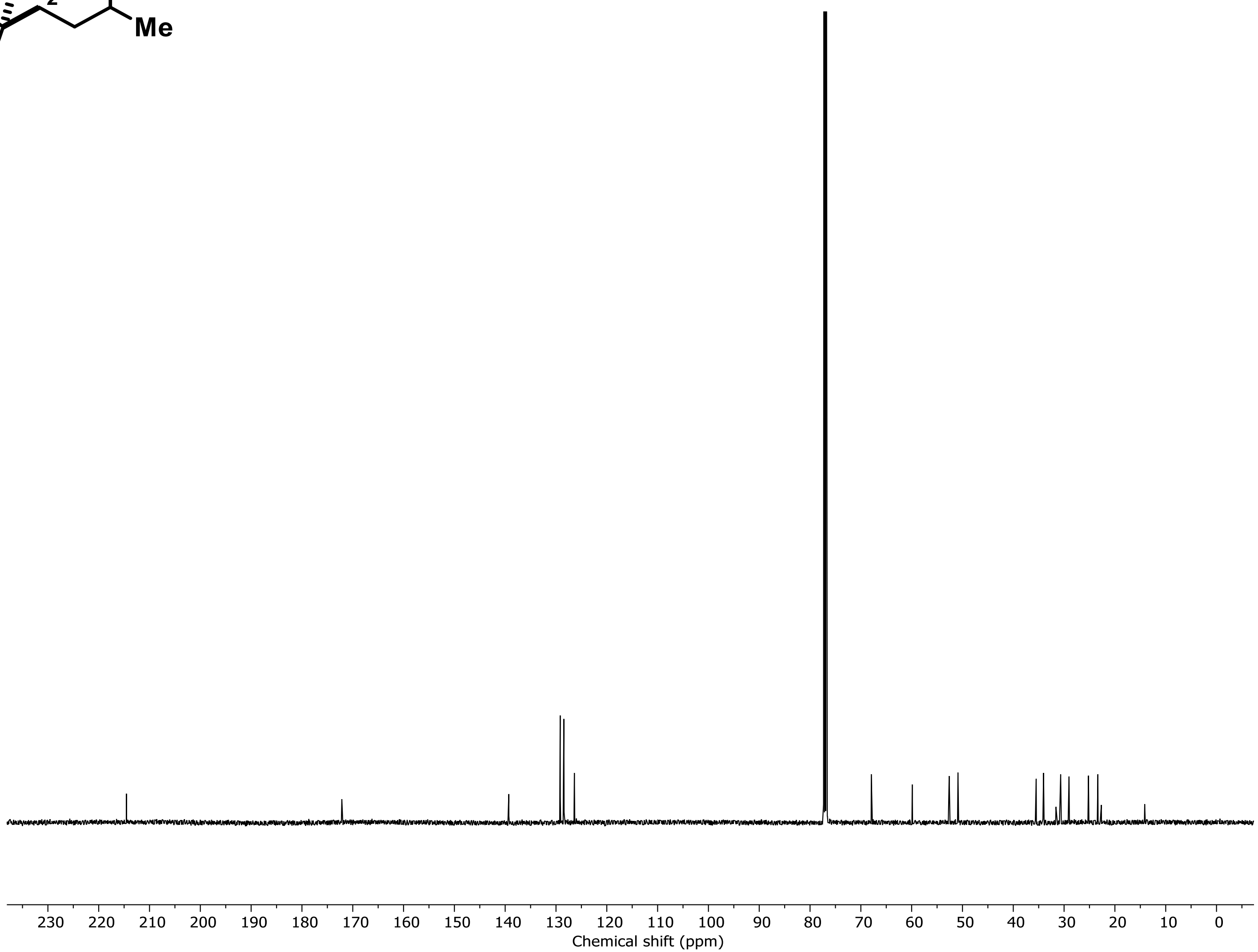

***Rac*–methyl (*R*)–2–benzyl–4–((3*S*,6*S*)–6–ethyl–2–oxotetrahydro–2*H*–pyran–3–yl)butanoate (3b)**

^{1}H NMR (500 MHz, $CDCl_3$)

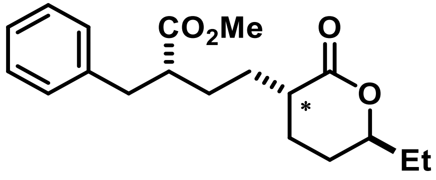

3:1 d.r.

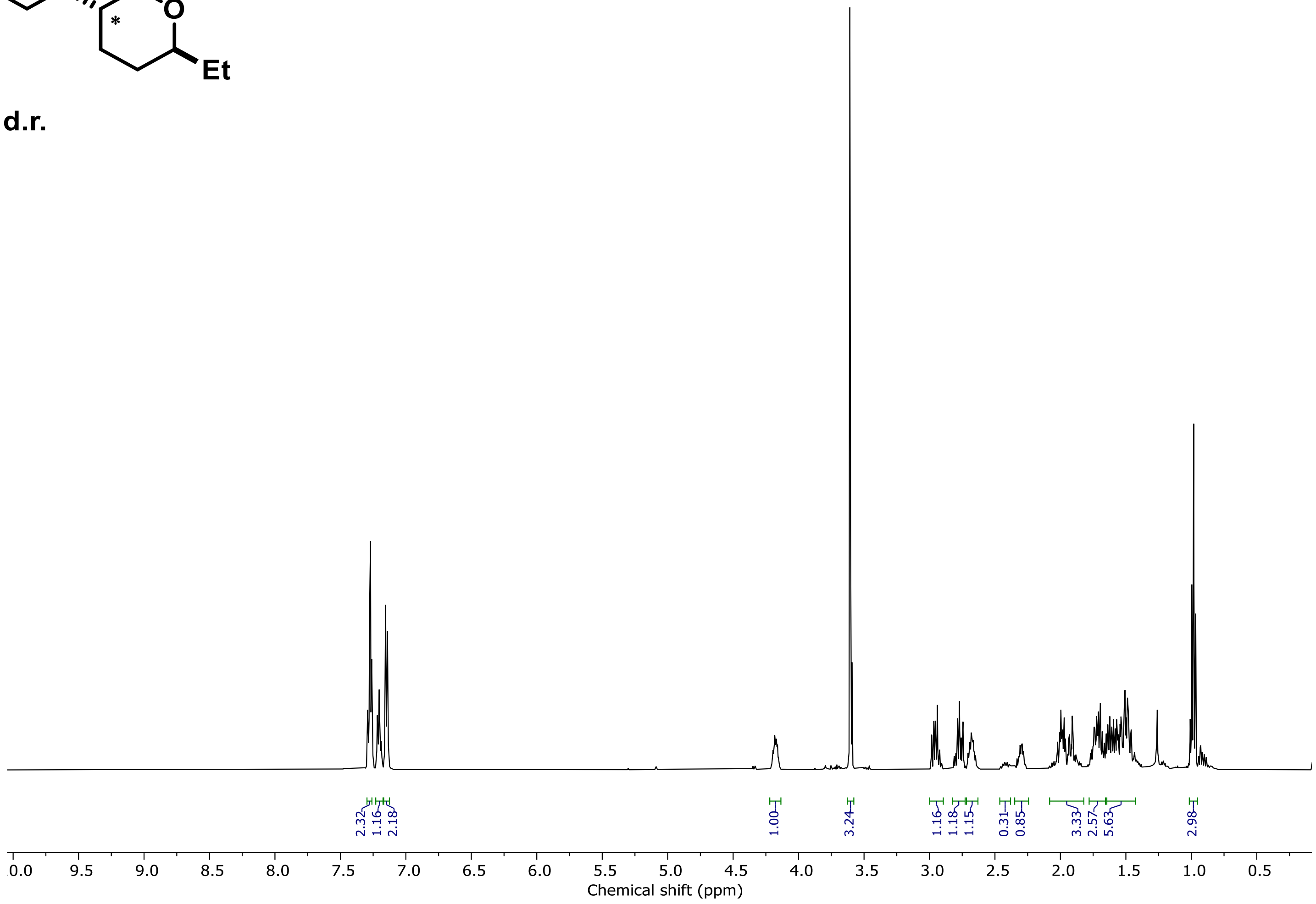

^{13}C NMR (126 MHz, $CDCl_3$)

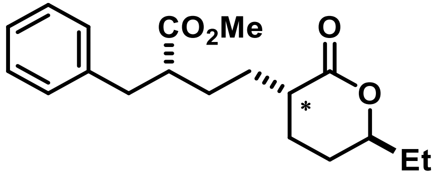

3:1 d.r.

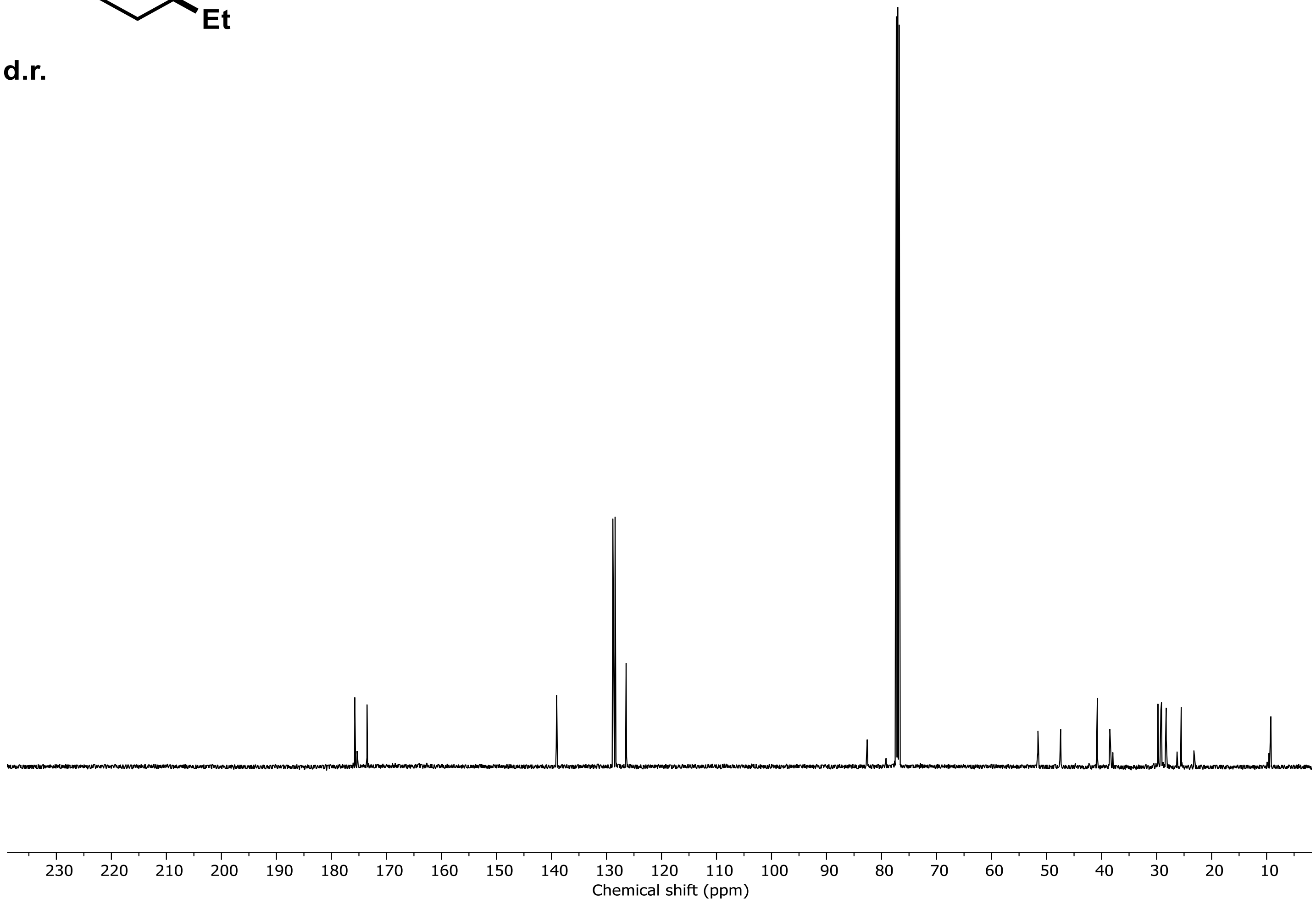

Rac–methyl (_R_)–2–benzyl–4–((3_S_,6_S_)–6–butyl–2–oxotetrahydro–2_H_–pyran–3–yl)butanoate (3c)

^{1}H NMR (400 MHz, $CDCl_3$)

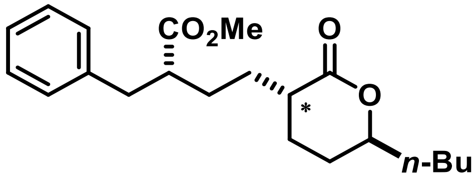

1.7:1 d.r.

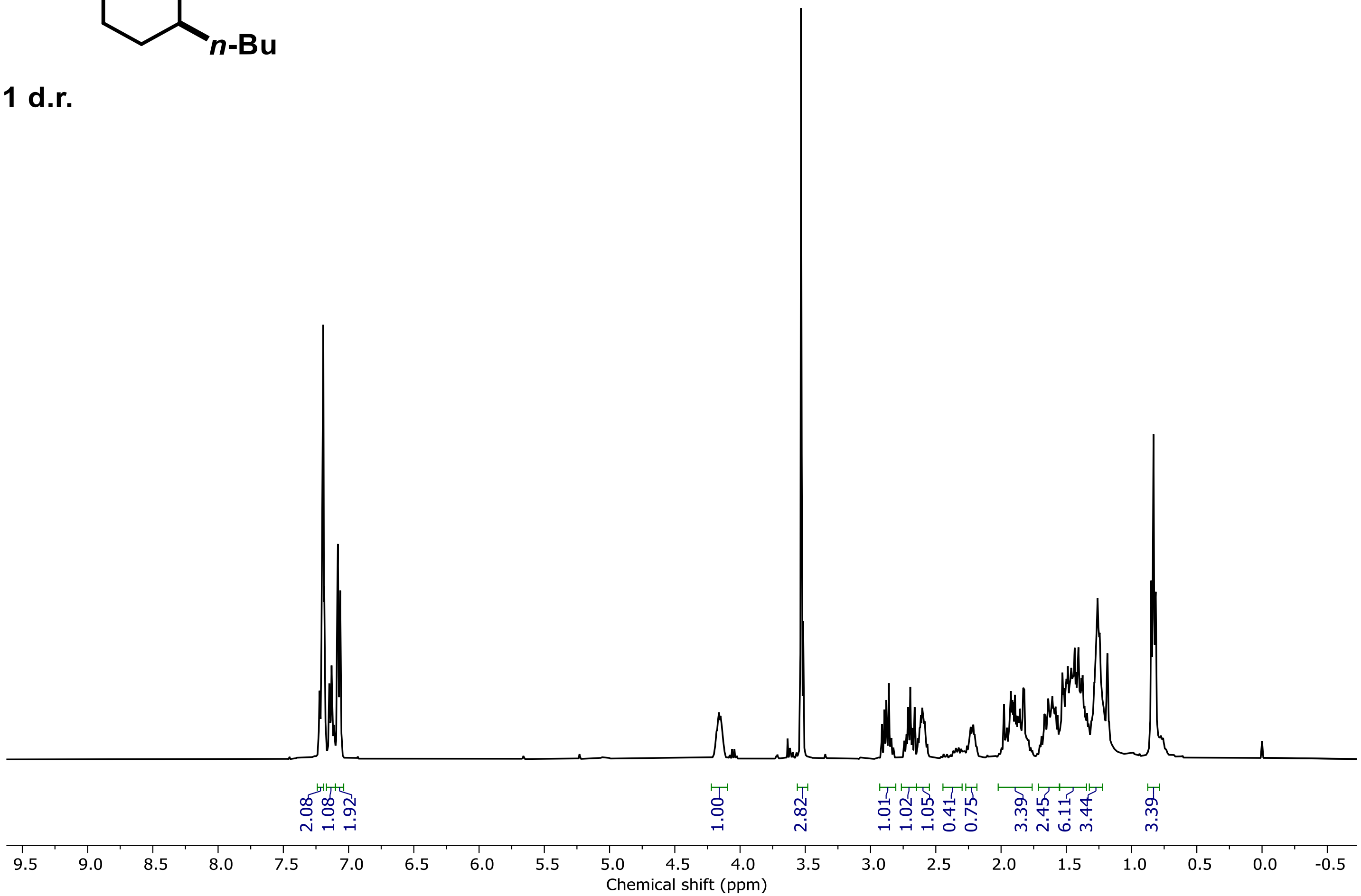

^{13}C NMR (101 MHz, $CDCl_3$)

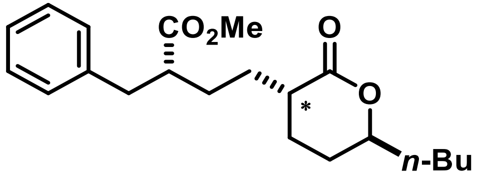

1.7:1 d.r.

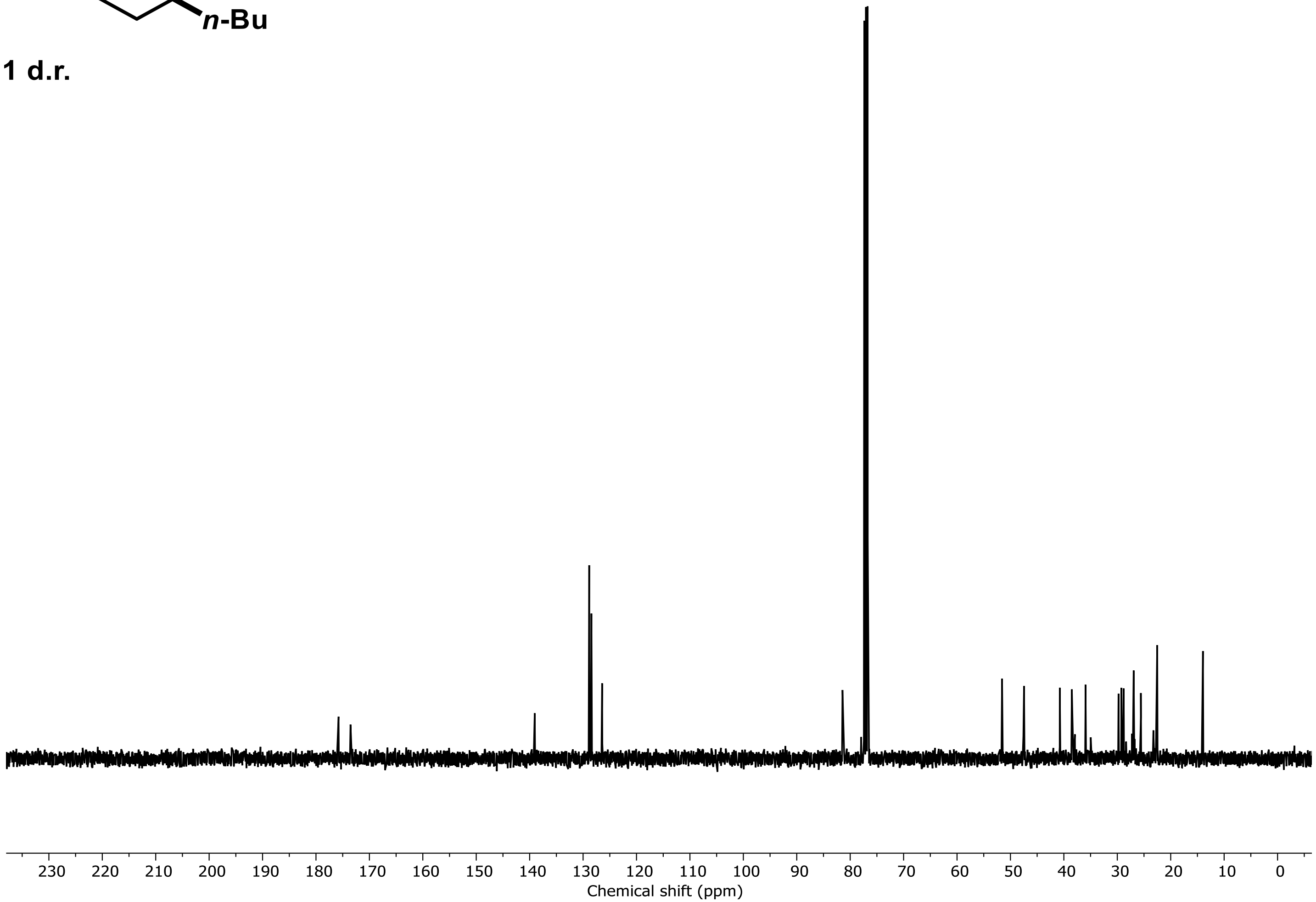

***Rac*–methyl (*R*)–2–benzyl–4–((3*S*,6*R*)–6–benzyl–2–oxotetrahydro–2*H*–pyran–3–yl)butanoate (3d)**

^{1}H NMR (500 MHz, $CDCl_3$)

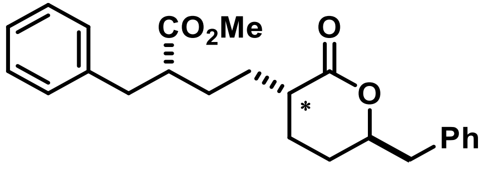

1:1 d.r.

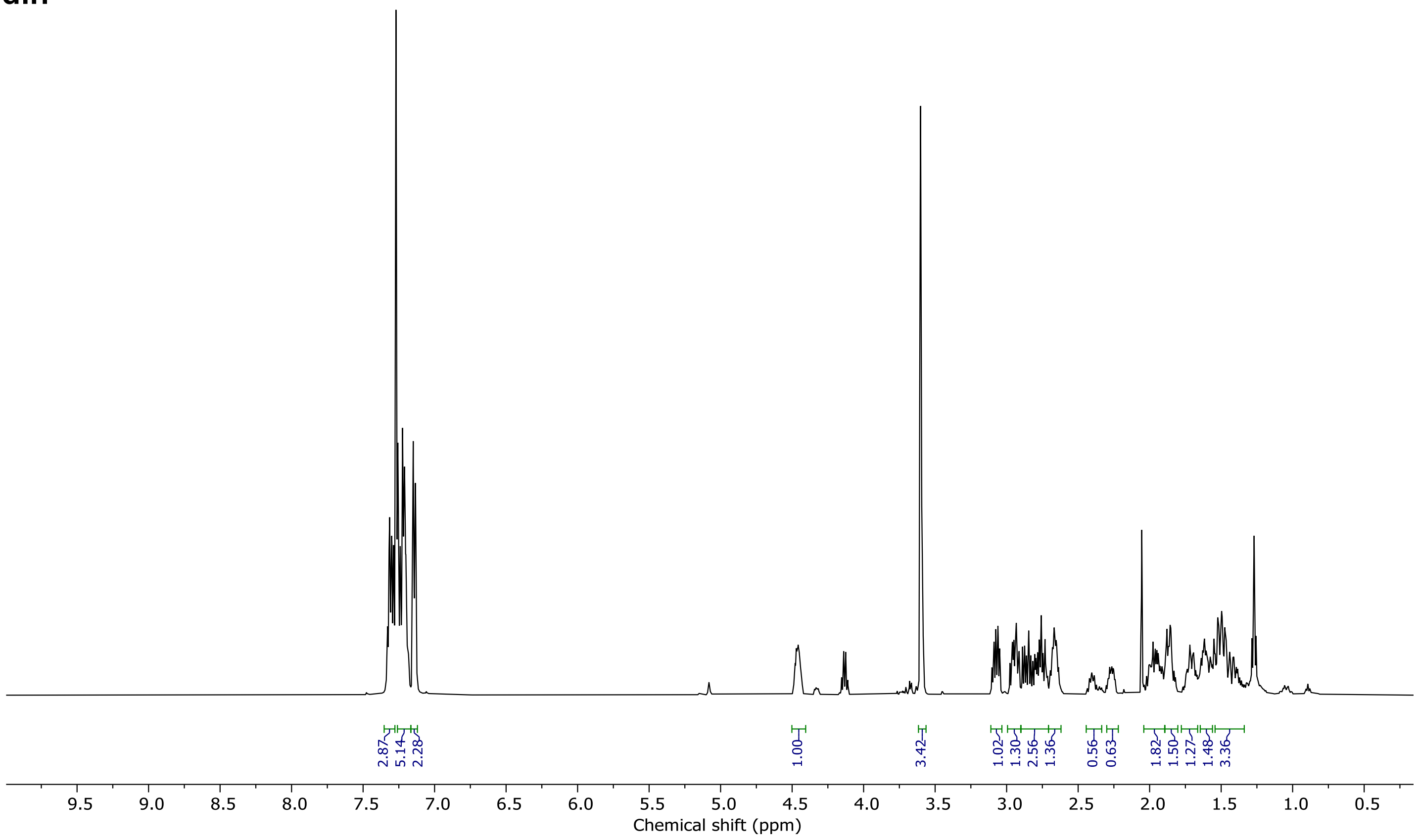

^{13}C NMR (126 MHz, $CDCl_3$)

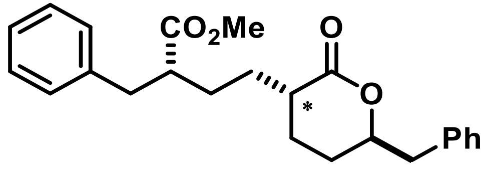

1:1 d.r.

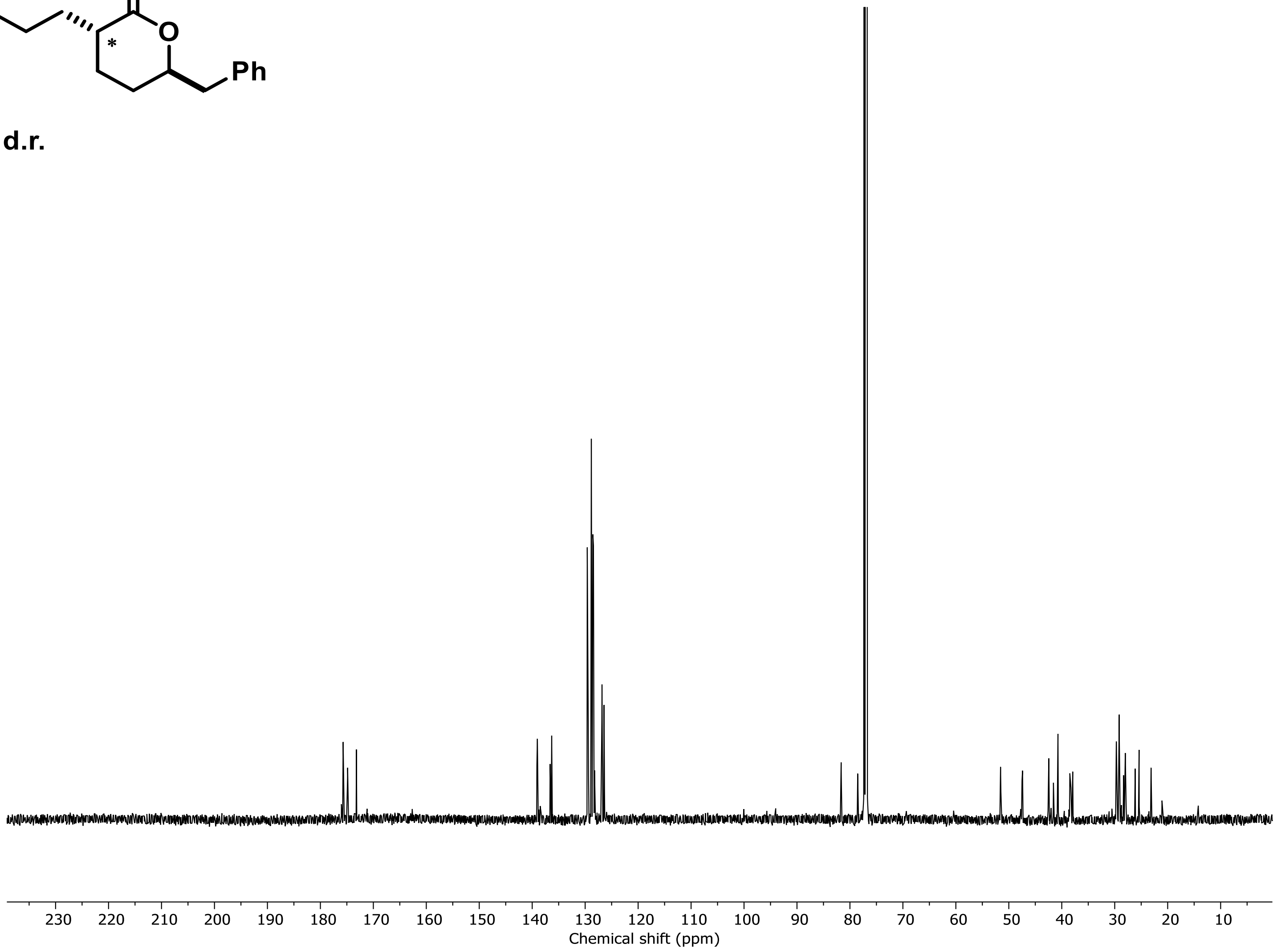

***Rac*–methyl (*R*)–2–benzyl–4–((3*S*,6*R*)–6–neopentyl–2–oxotetrahydro–2*H*–pyran–3–yl)butanoate (3e)**

^{1}H NMR (500 MHz, $CDCl_3$)

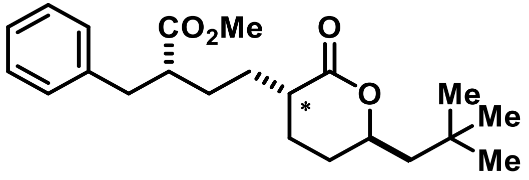

1.4:1 d.r.

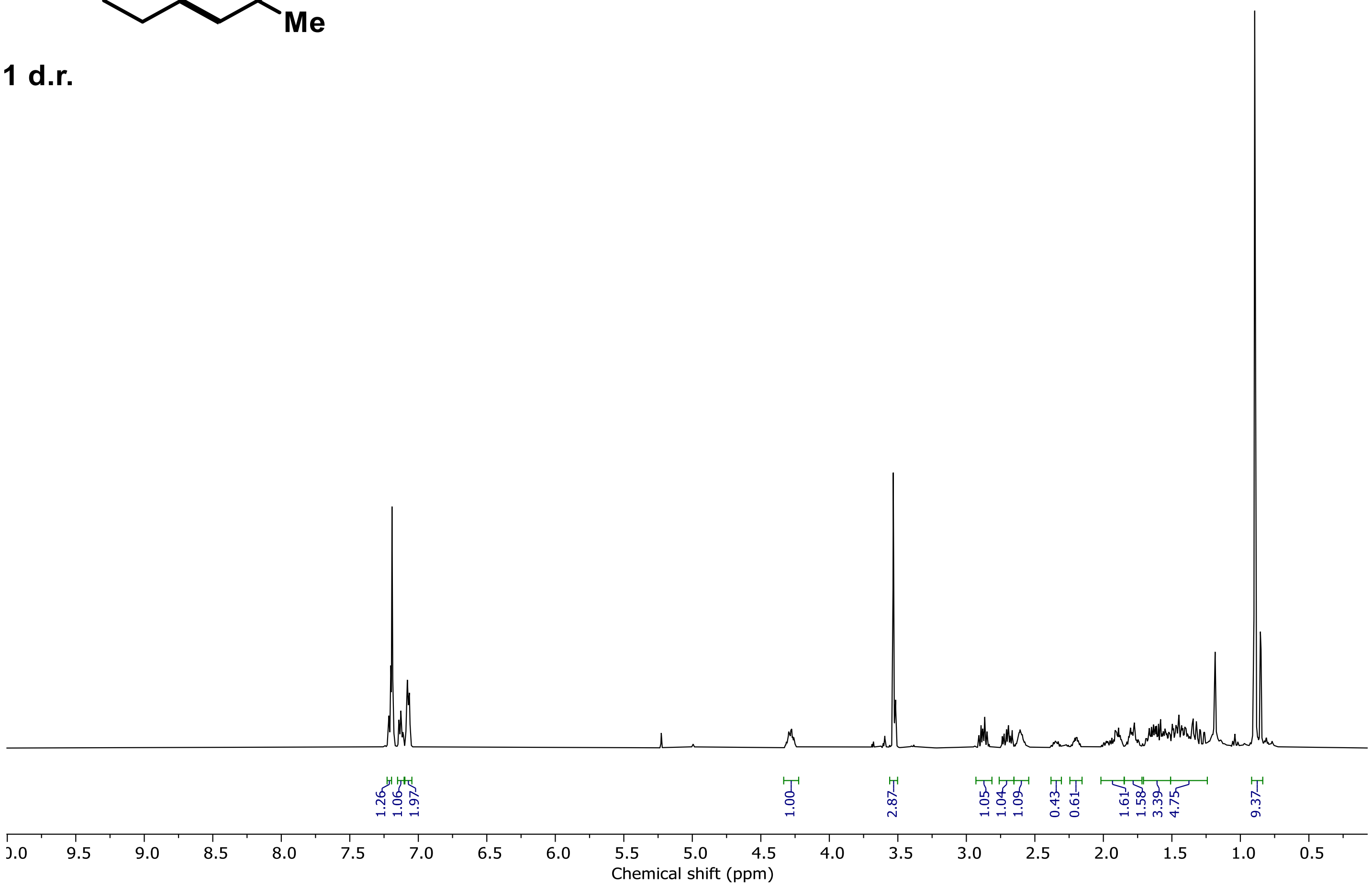

^{13}C NMR (126 MHz, $CDCl_3$)

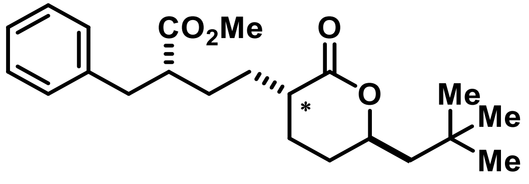

1.4:1 d.r.

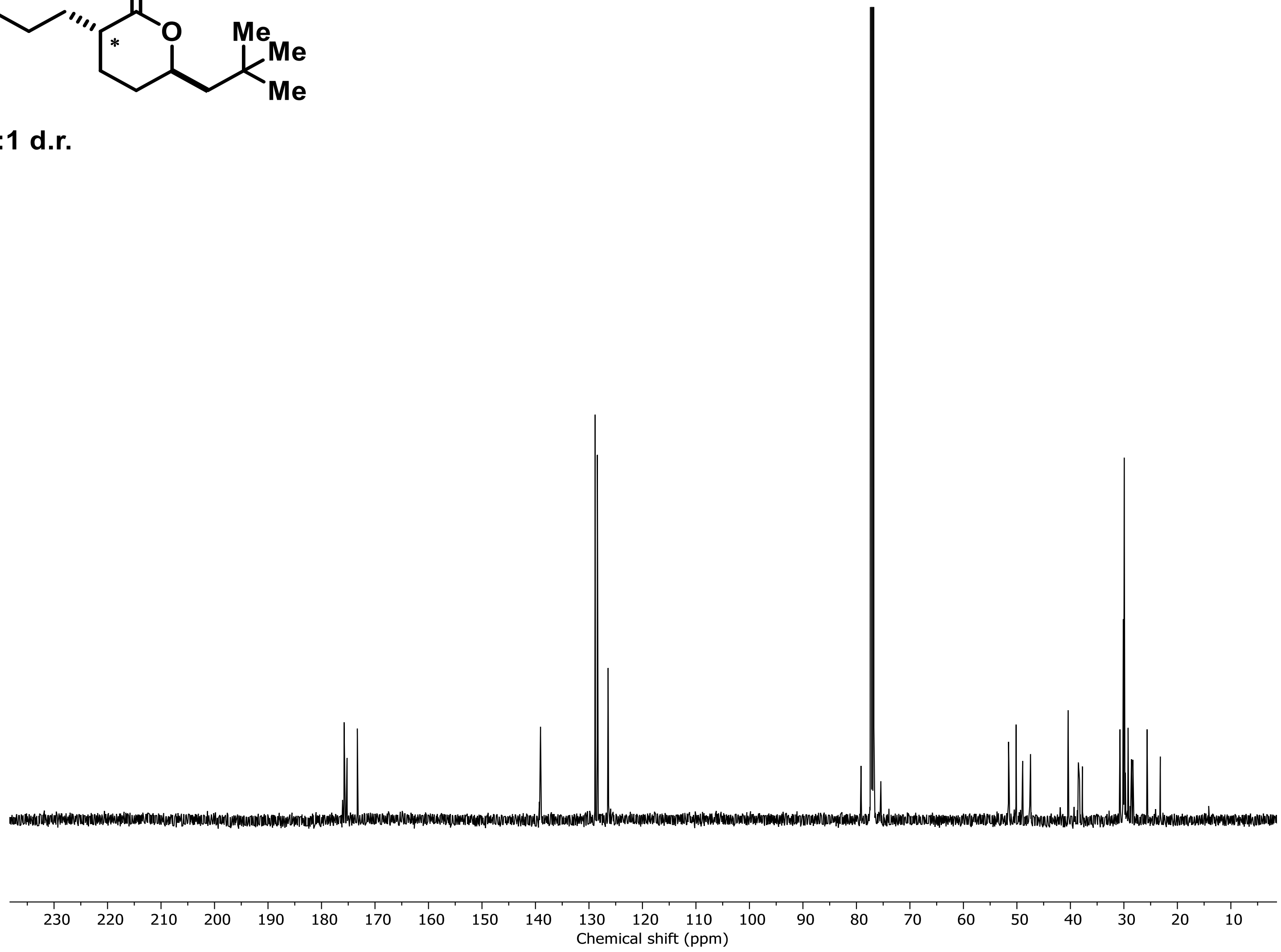

***Rac*–methyl (*R*)–2–benzyl–4–((3*S*,6*R*)–6–isopropyl–2–oxotetrahydro–2*H*–pyran–3–yl)butanoate (3f)**

^{1}H NMR (500 MHz, $CDCl_3$)

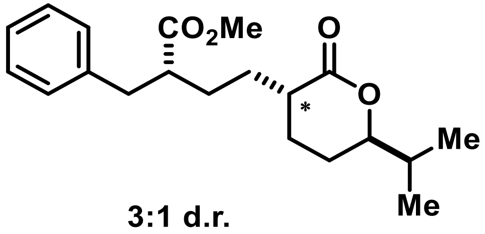

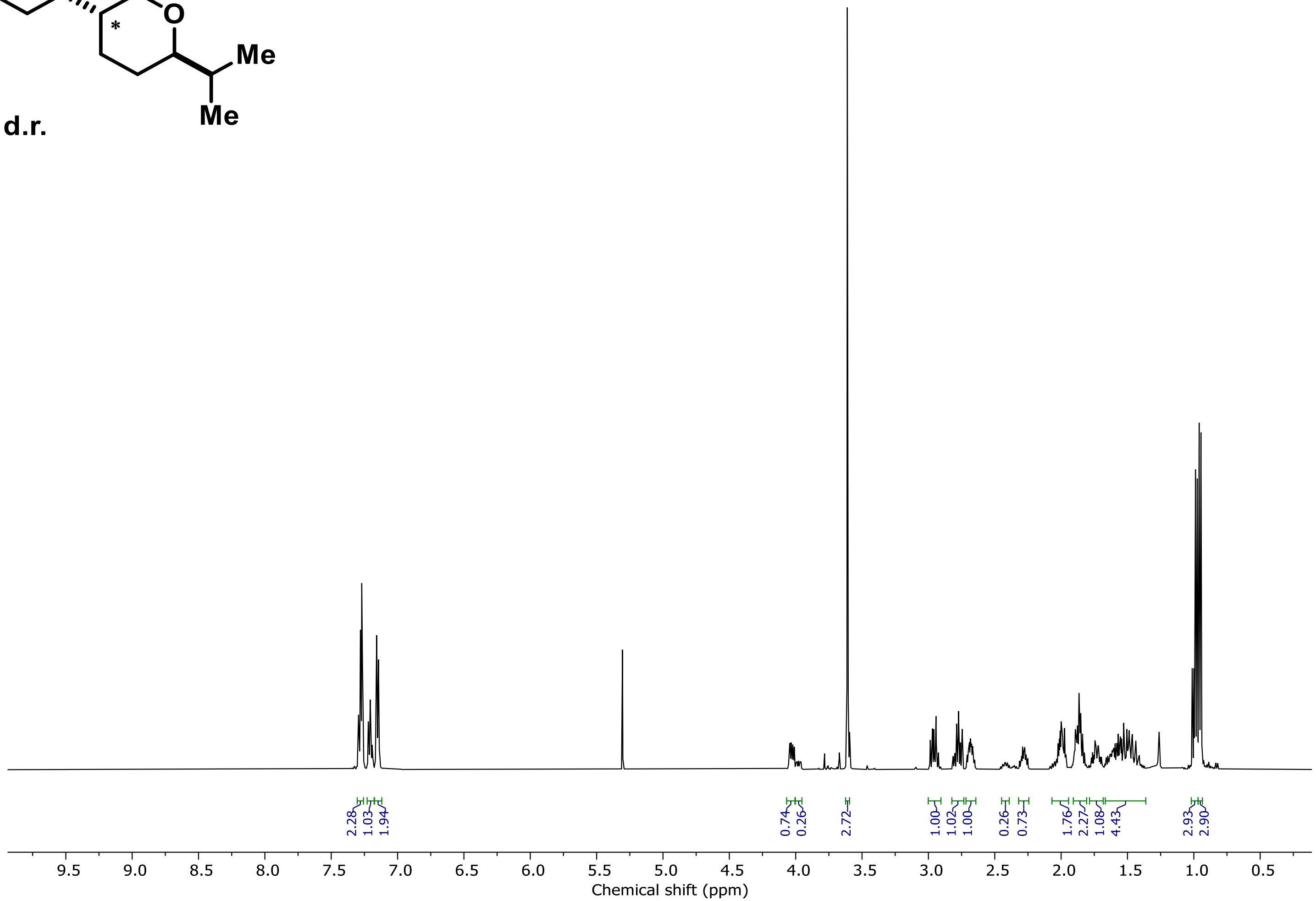

^{13}C NMR (126 MHz, $CDCl_3$)

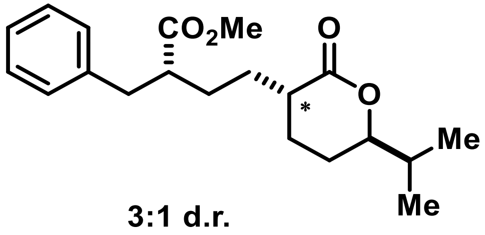

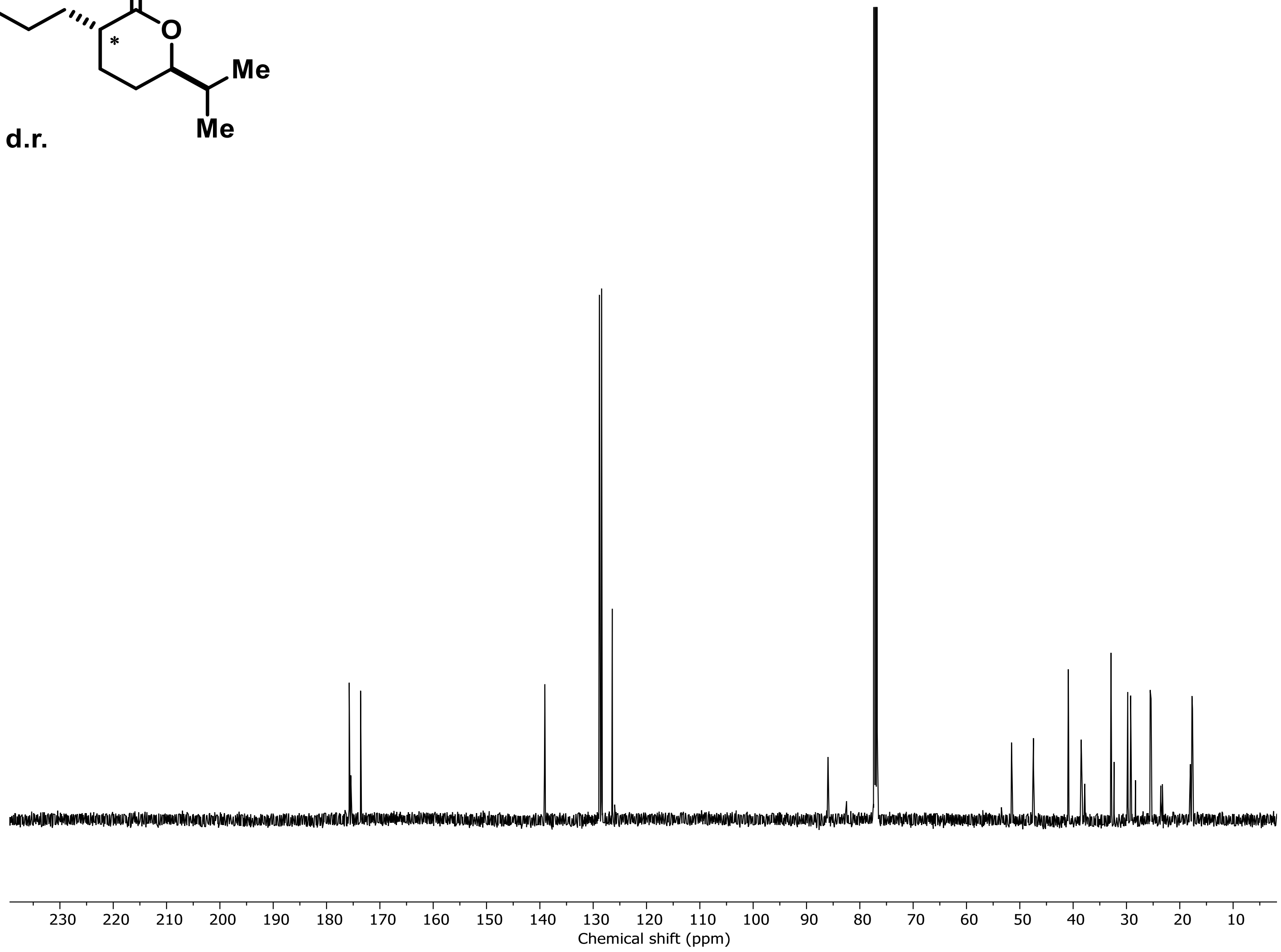

***Rac*–methyl (*R*)–4–((3*S*,6*R*)–6–benzhydryl–2–oxotetrahydro–2*H*–pyran–3–yl)–2–benzylbutanoate (3g)**

400 MHz, $CDCl_3$

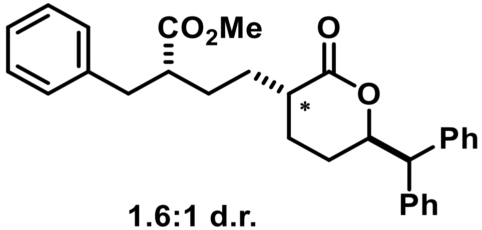

1.6:1 d.r.

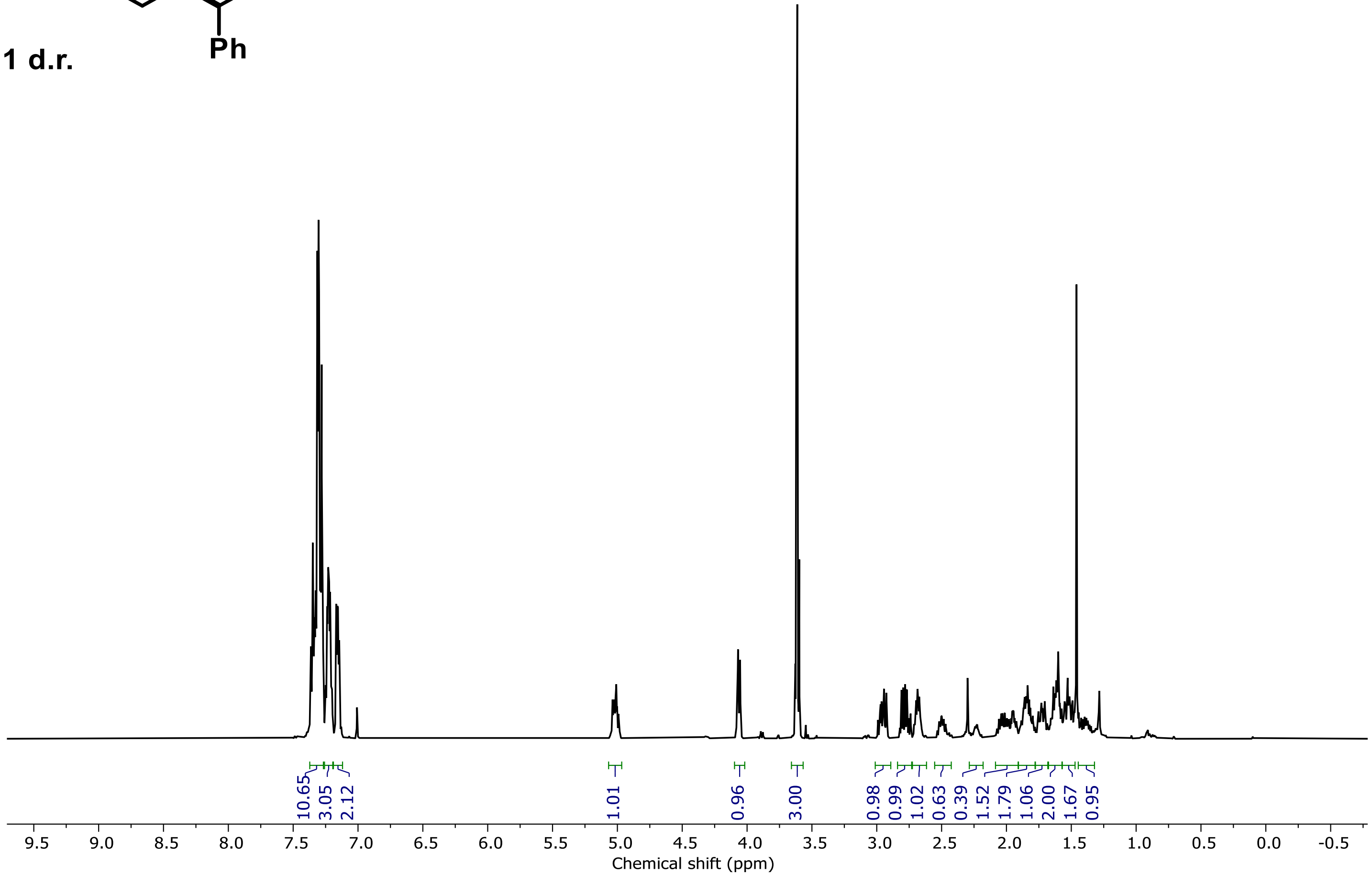

^{13}C NMR (101 MHz, $CDCl_3$)

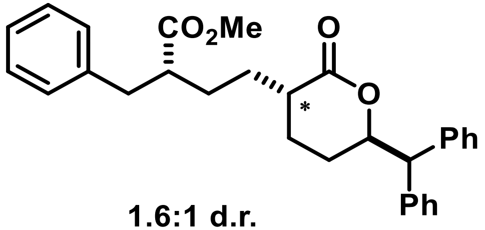

1.6:1 d.r.

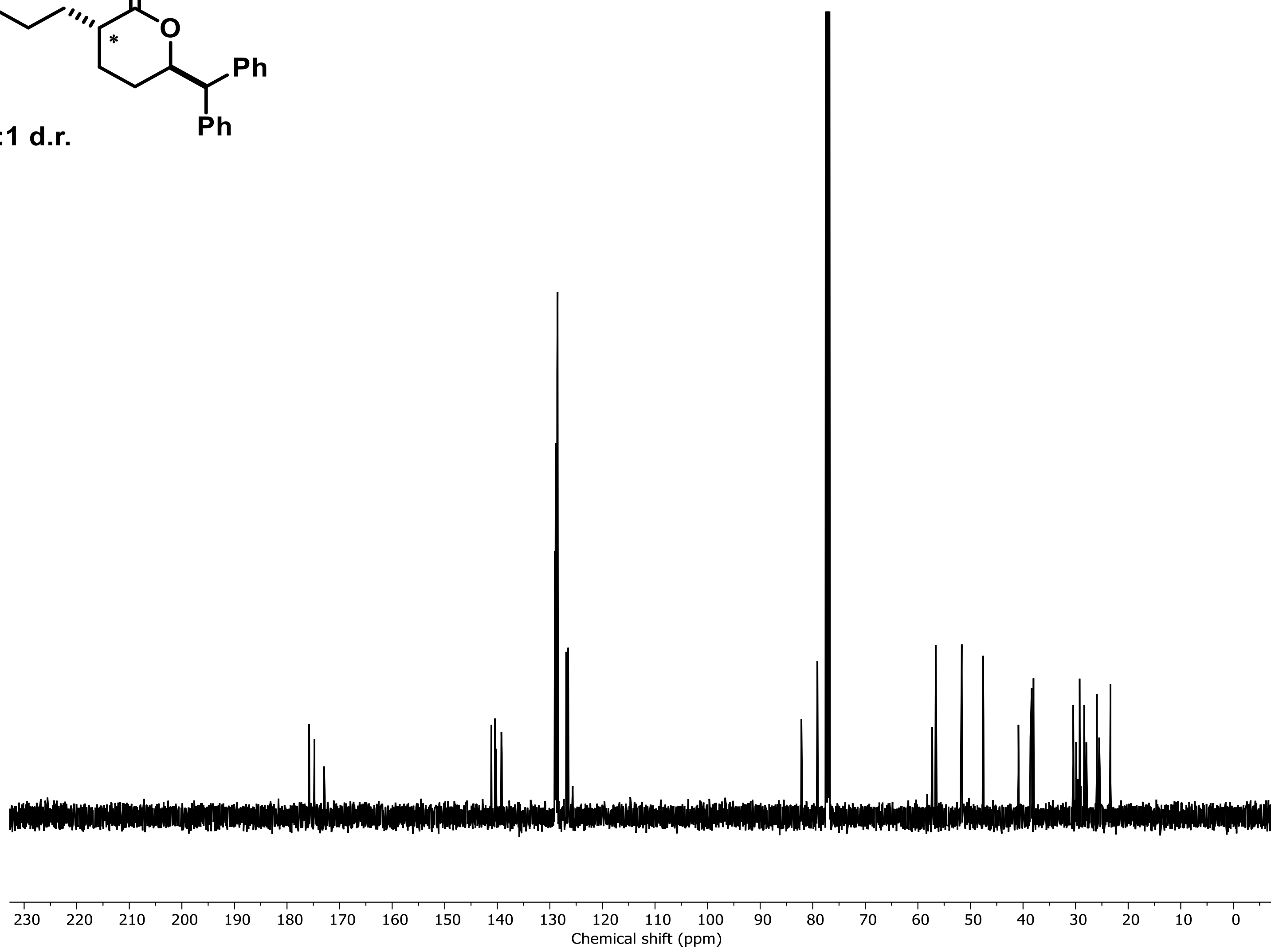

***Rac*–methyl (*R*)–2–benzyl–4–((3*S*,6*R*)–6–cyclohexyl–2–oxotetrahydro–2*H*–pyran–3–yl)butanoate (3h)**

^{1}H NMR (500 MHz, $CDCl_3$)

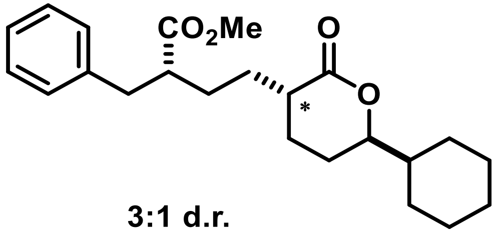

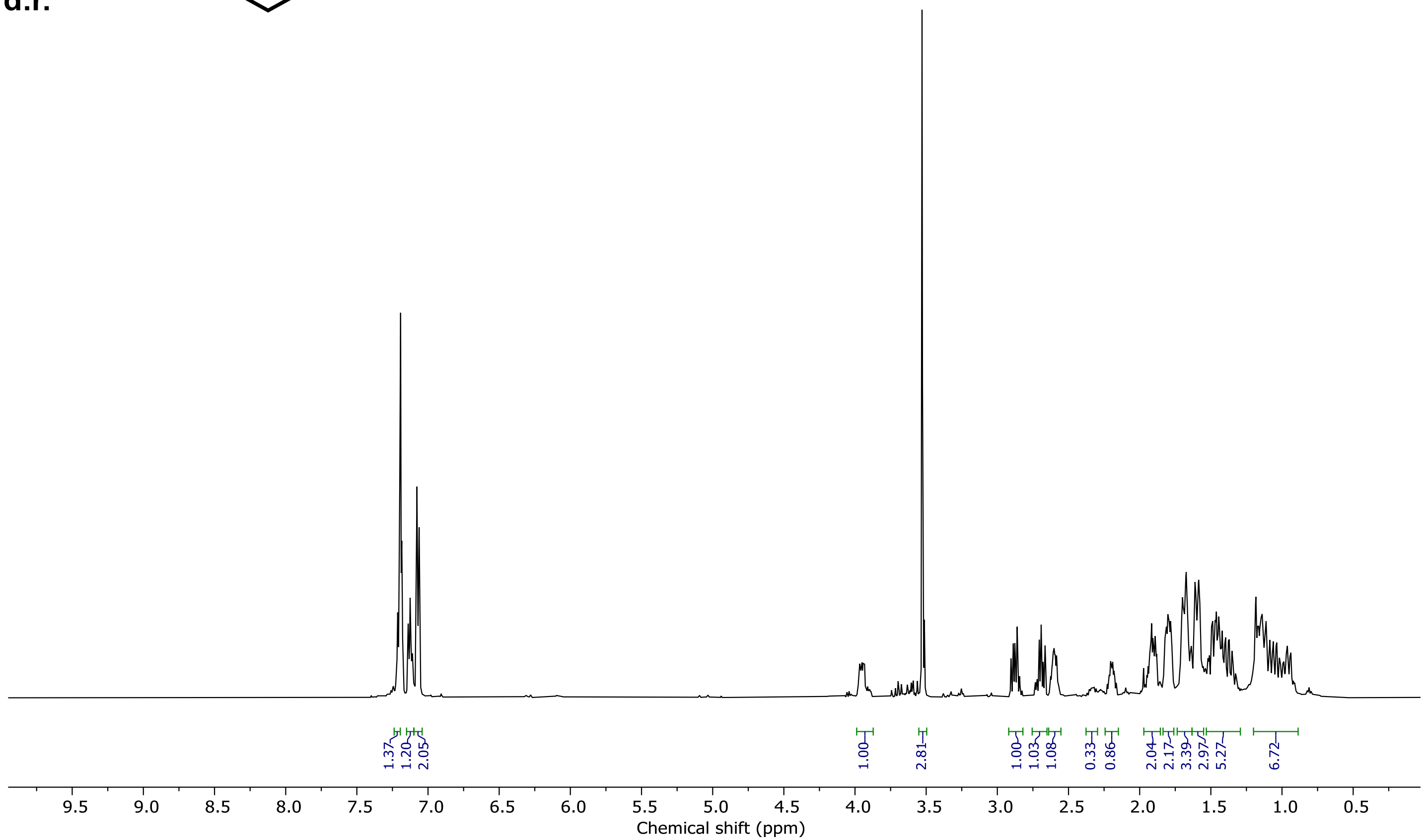

^{13}C NMR (126 MHz, $CDCl_3$)

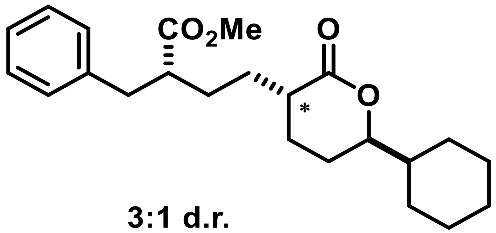

3:1 d.r.

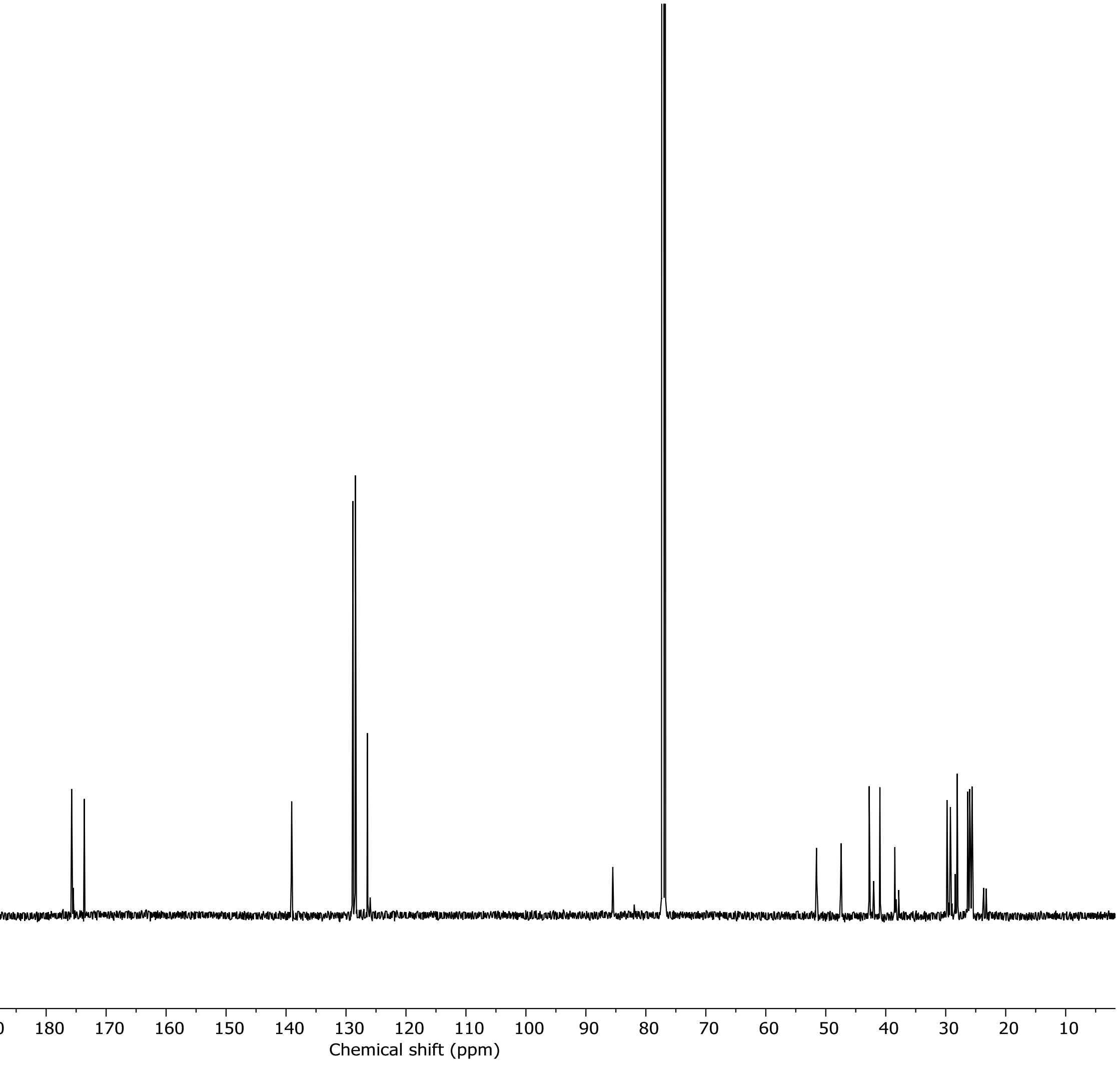

***Rac*–Methyl (*R*)–2–benzyl–4–((2*R*,5*S*)–6–oxooctahydro–2*H*,2'*H*–[2,4'–bipyran]–5–yl)butanoate (3i)**

^{1}H NMR (400 MHz, $CDCl_3$)

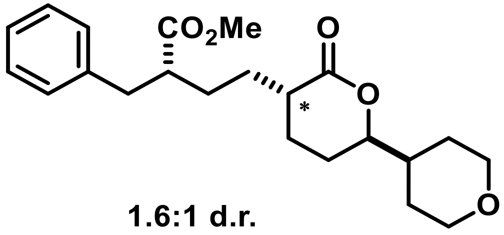

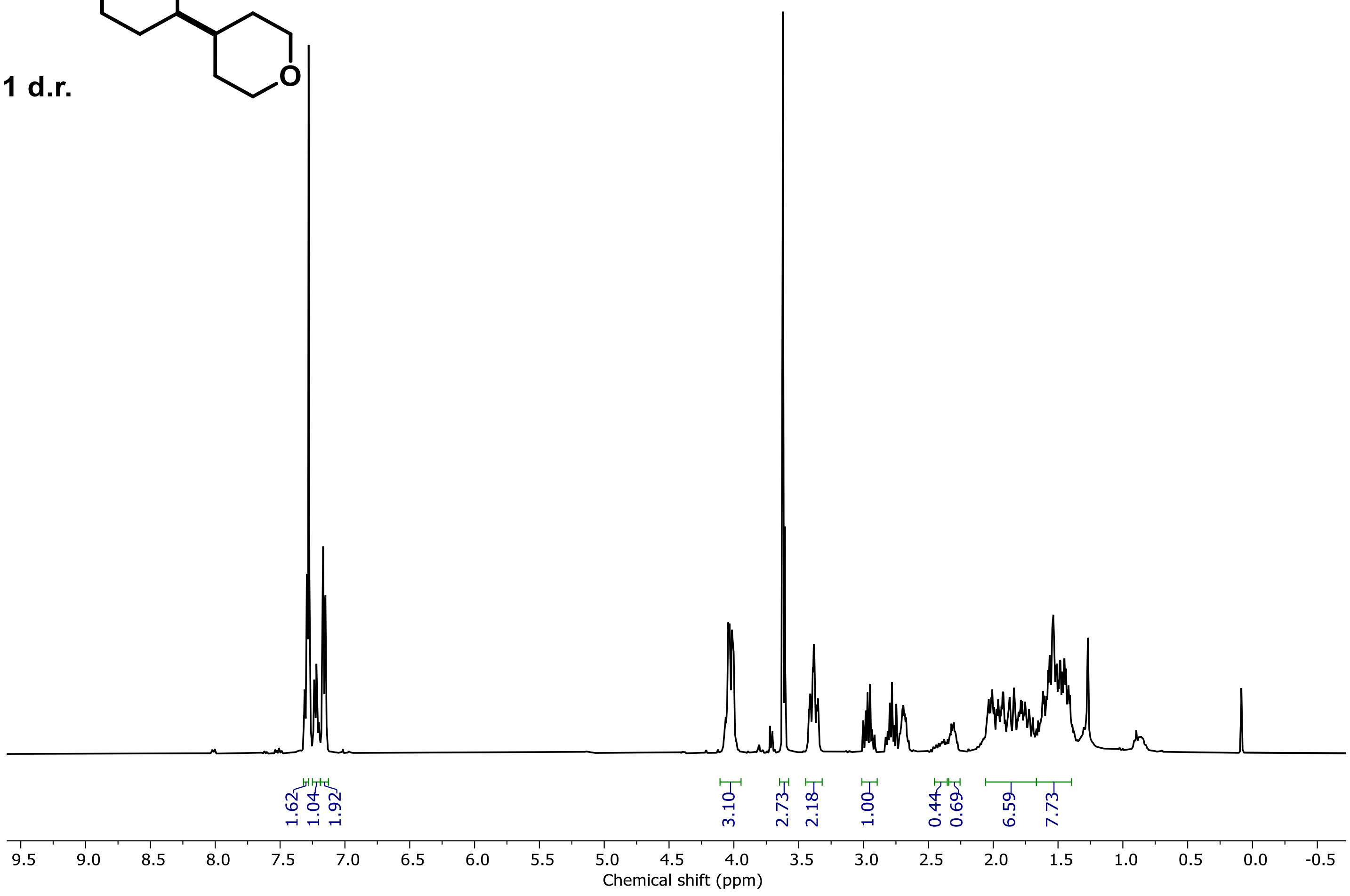

^{13}C NMR (101 MHz, $CDCl_3$)

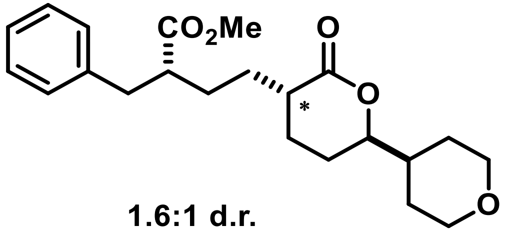

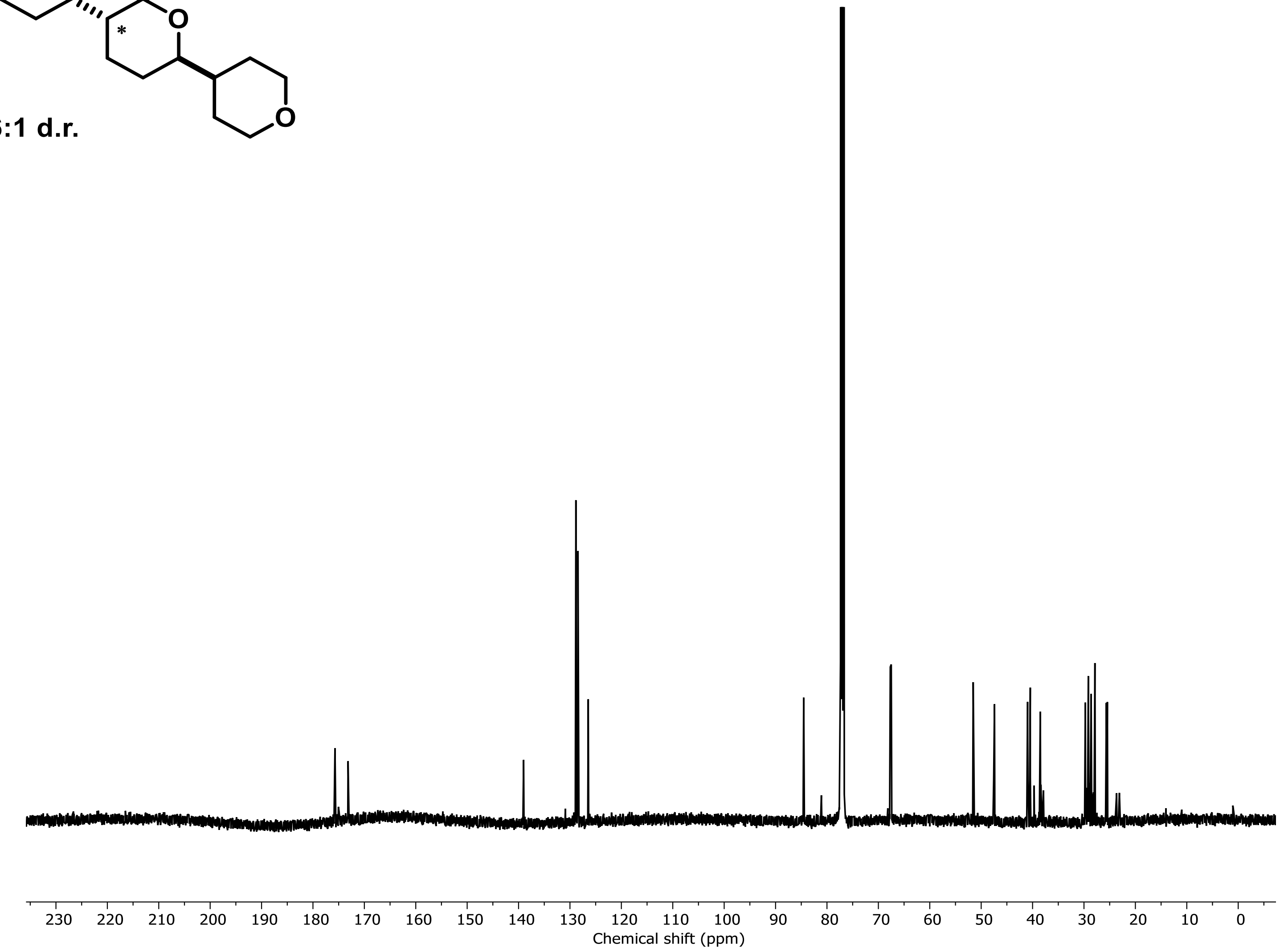

Rac–methyl (_R_)–2–benzyl–4–((3_S_,6_R_)–6–(tert–butyl)–2–oxotetrahydro–2_H_–pyran–3–yl)butanoate (3j)

^{1}H NMR (500 MHz, $CDCl_3$)

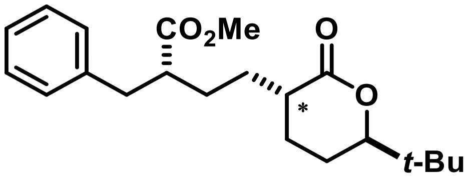

1.8:1 d.r.

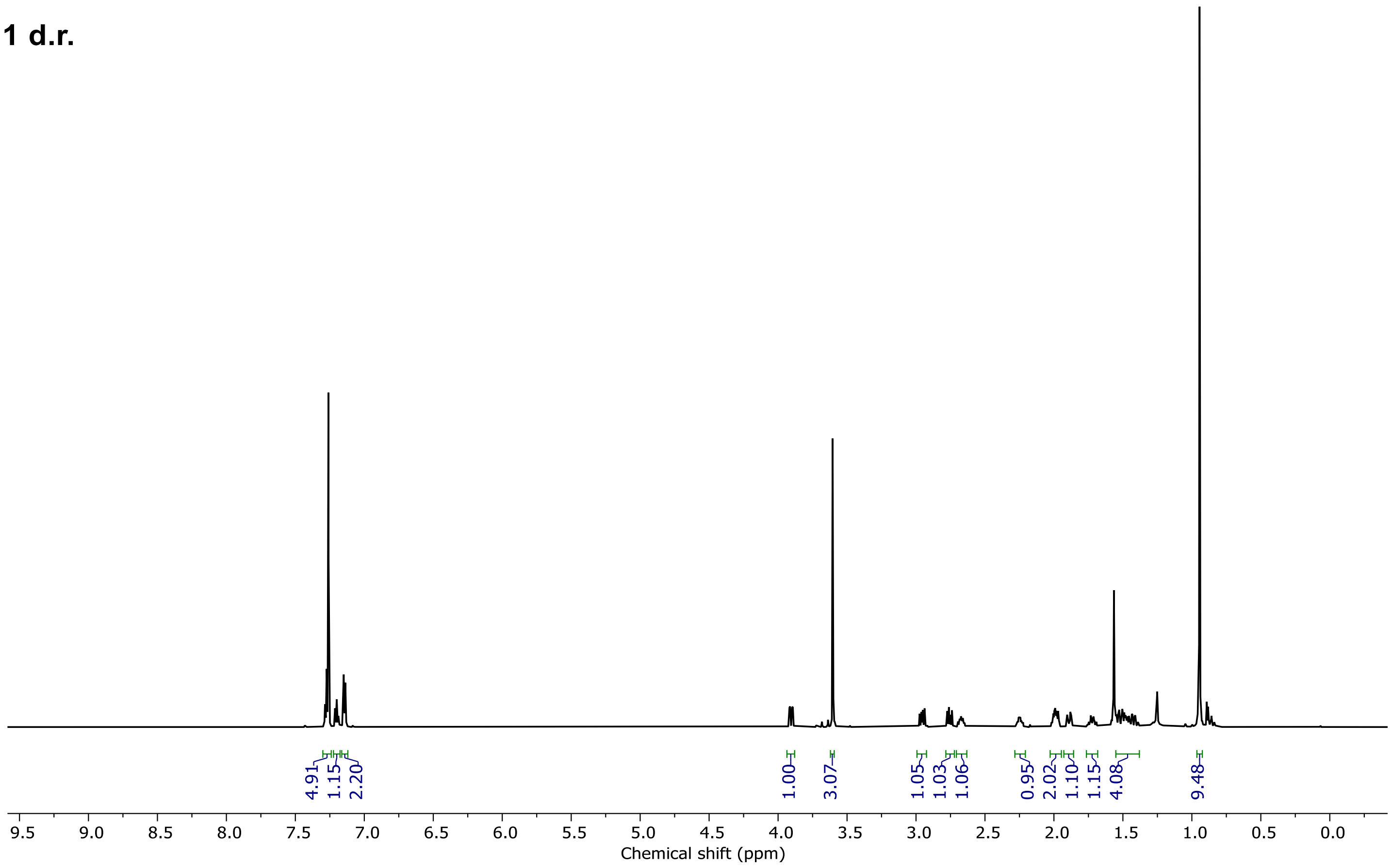

^{13}C NMR (126 MHz, $CDCl_3$)

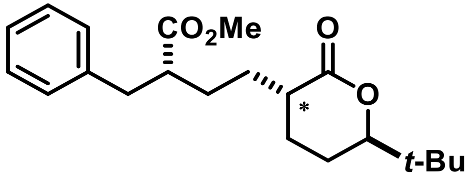

1.8:1 d.r.

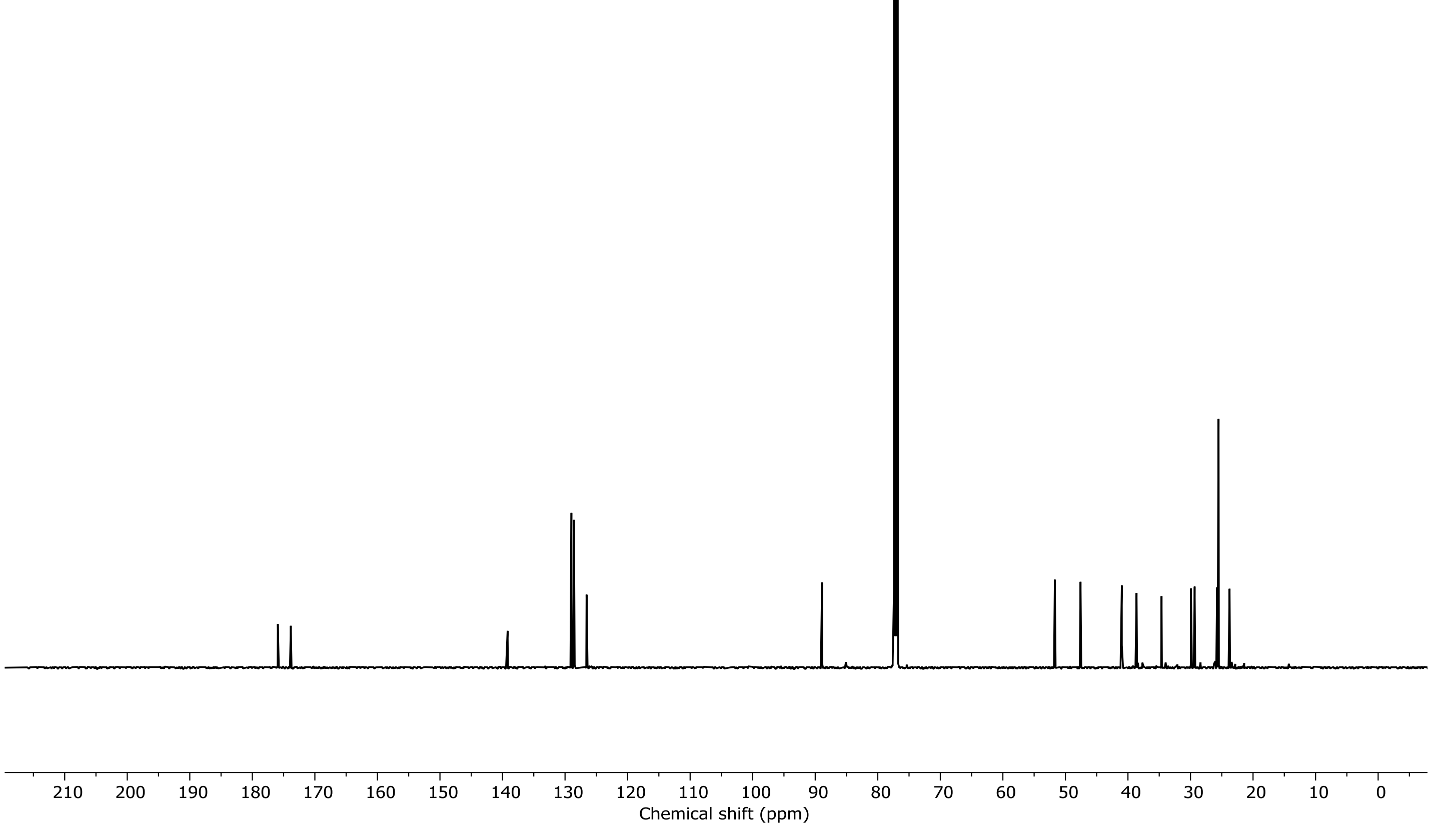

Rac–methyl (_R_)–4–((3_S_,6_S_)–6–ethyl–2–oxotetrahydro–2_H_–pyran–3–yl)–2–(2–fluorobenzyl) butanoate (3k)

^{1}H NMR (400 MHz, $CDCl_3$)

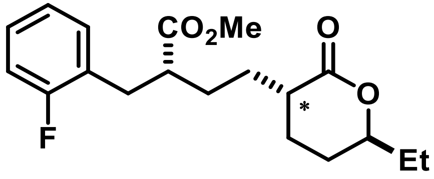

2.6:1 d.r.

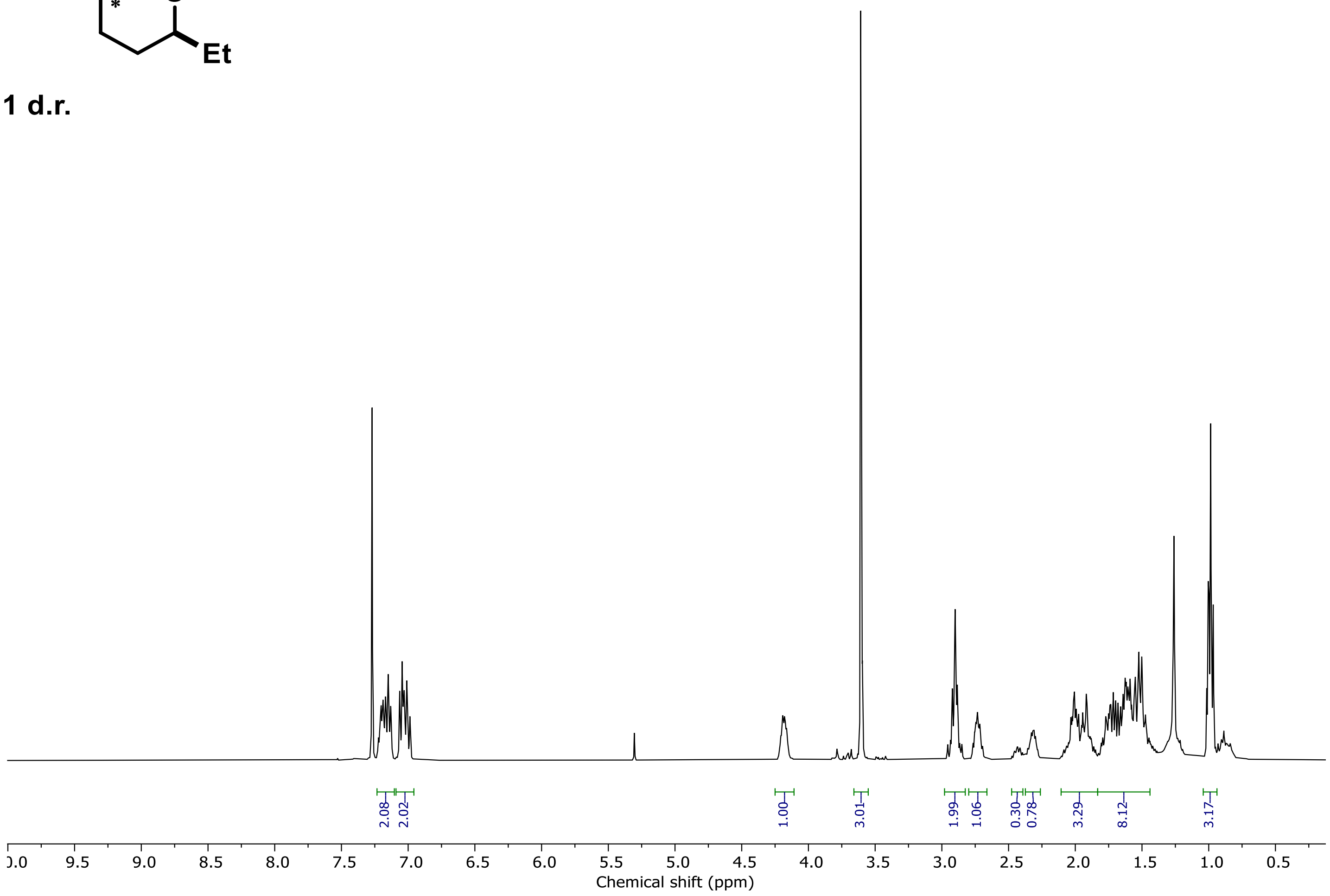

^{13}C NMR (101 MHz, $CDCl_3$)

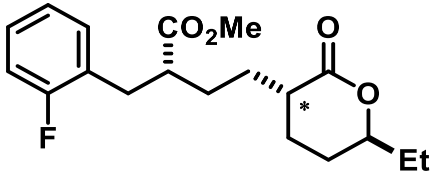

2.6:1 d.r.

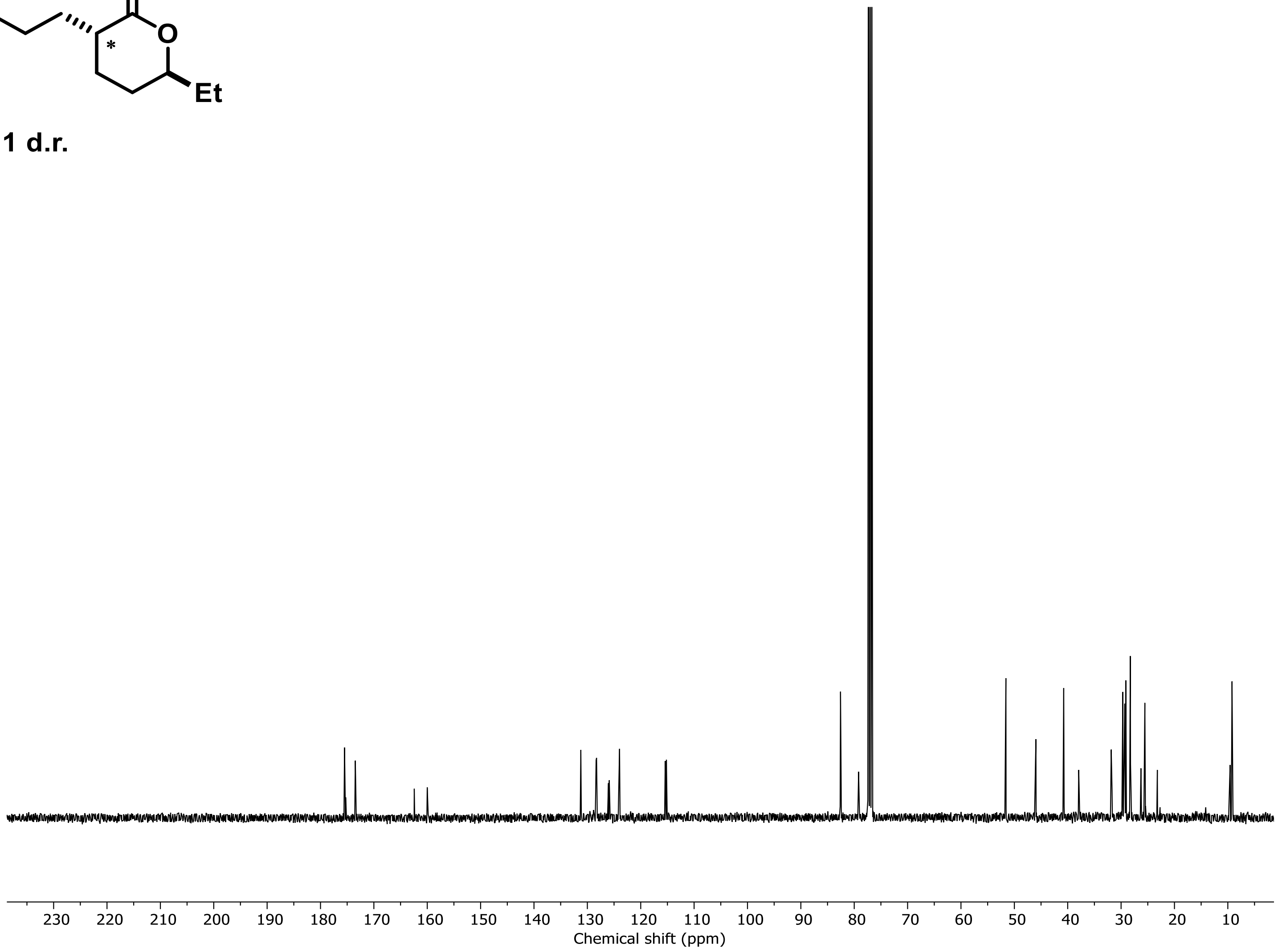

^{19}F NMR (376 MHz, $CDCl_3$)

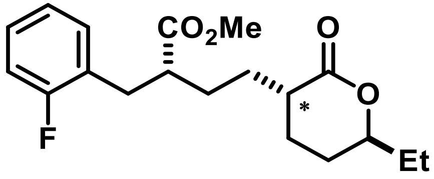

2.6:1 d.r.

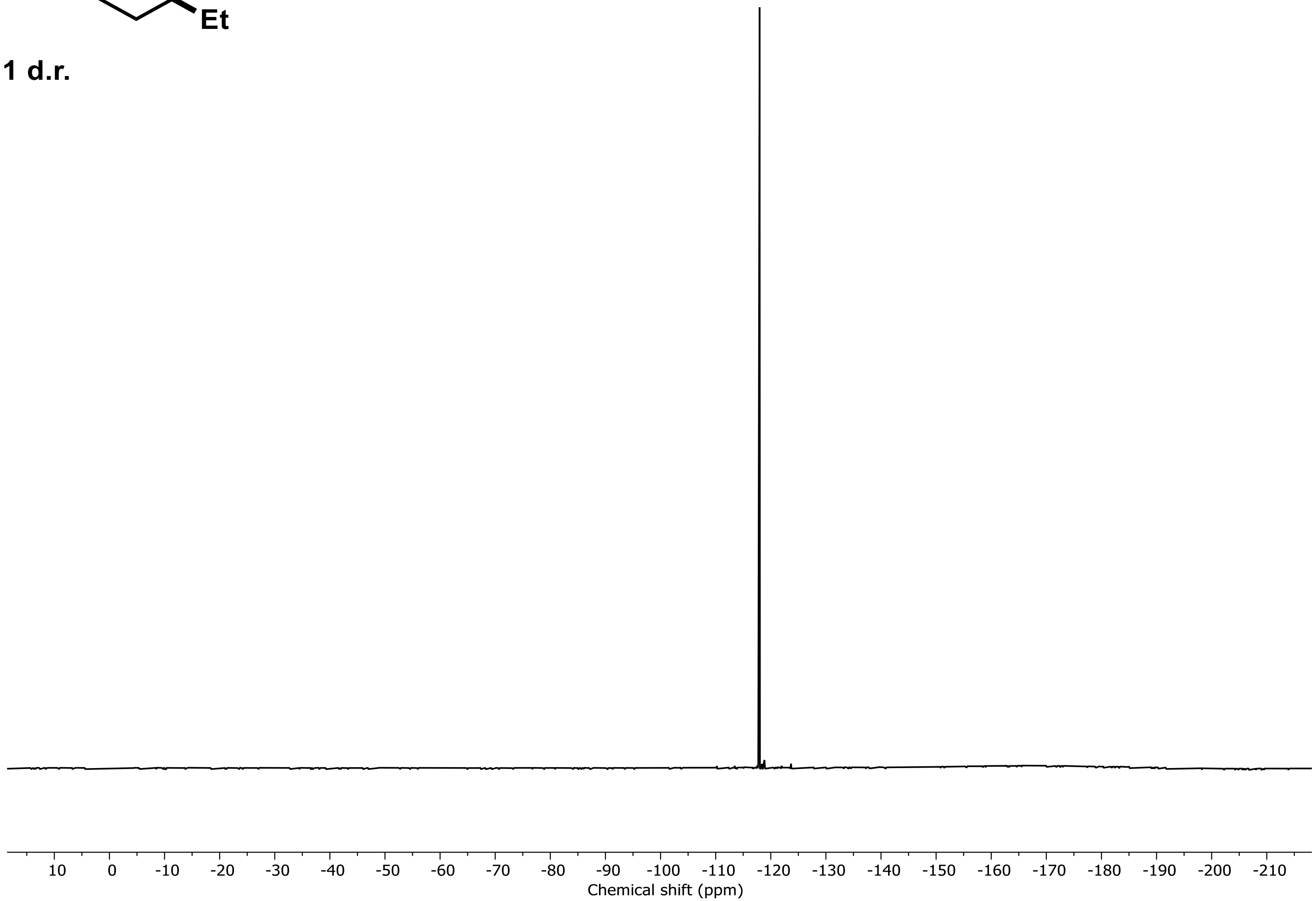

***Rac*–methyl (*R*)–4–((3*S*,6*S*)–6–ethyl–2–oxotetrahydro–2*H*–pyran–3–yl)–2–(2–methylbenzyl) butanoate (3l)**

^{1}H NMR (400 MHz, MeOD–d_4)

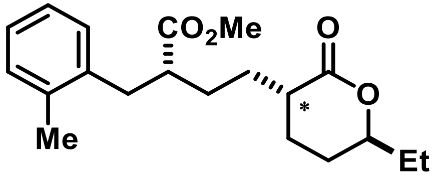

1.7:1 d.r.

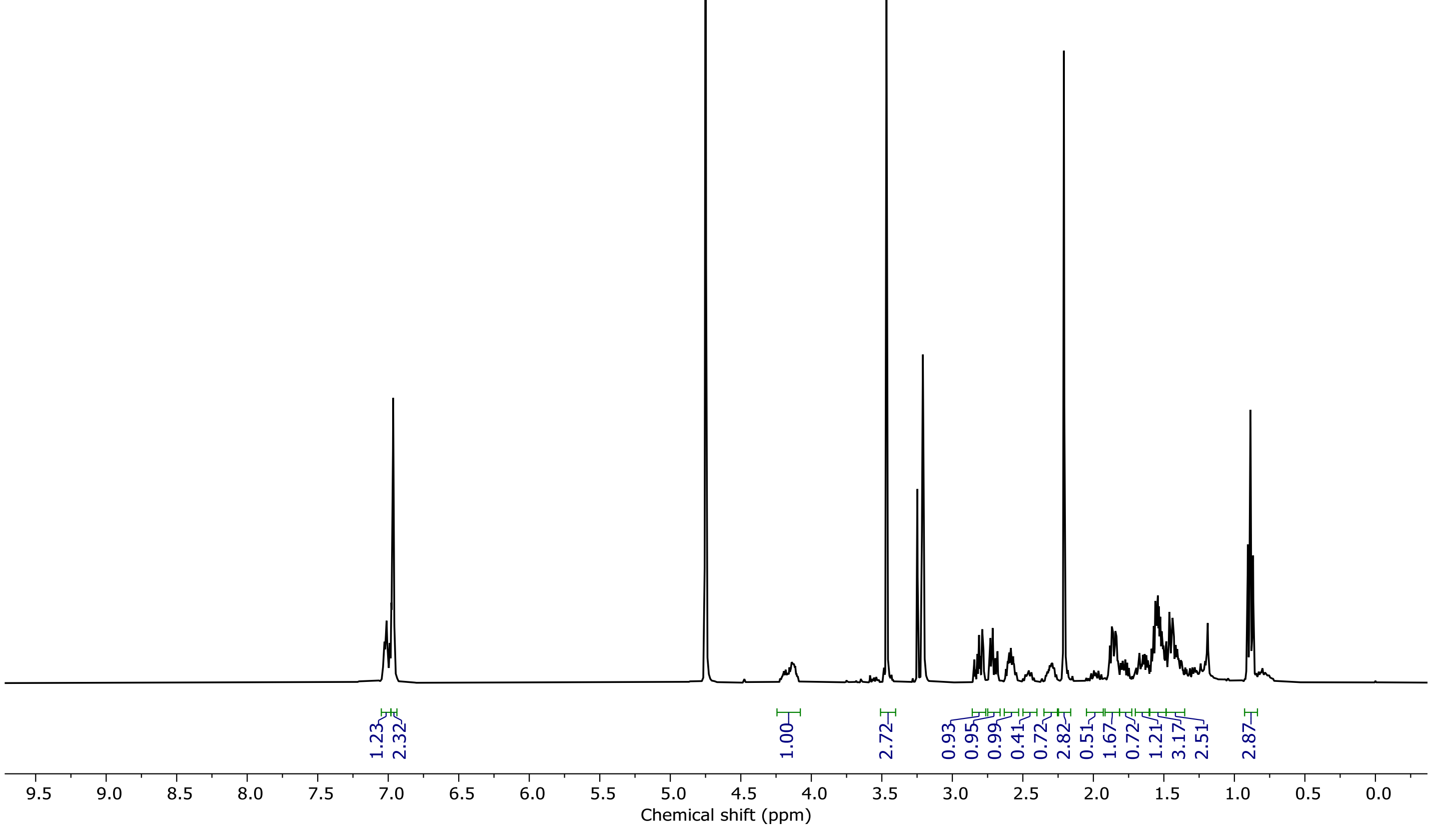

^{13}C NMR (400 MHz, MeOD–d_4)

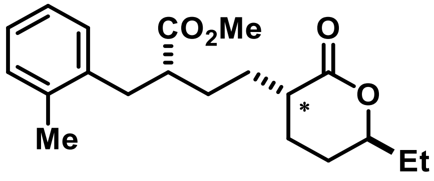

1.7:1 d.r.

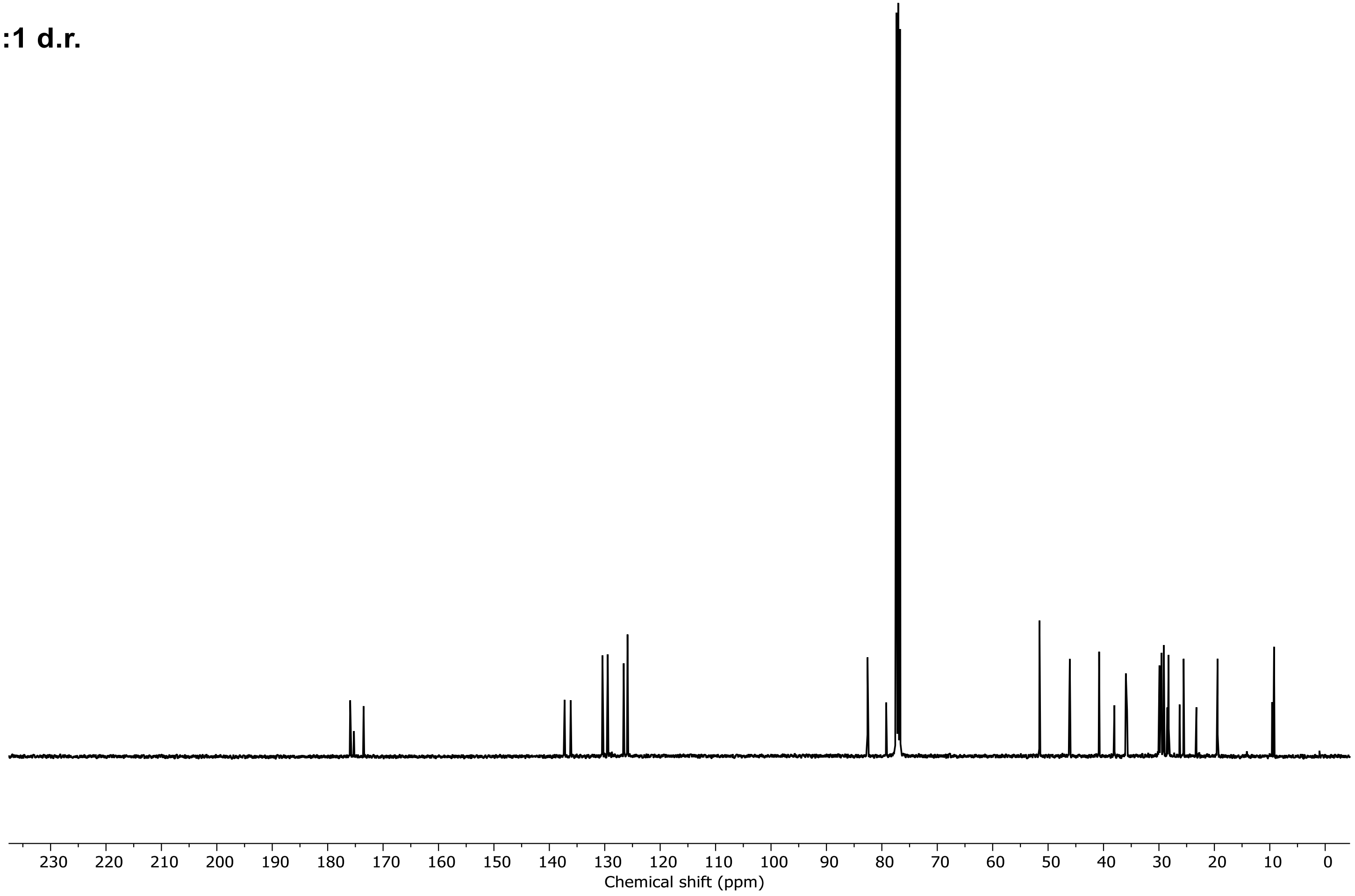

***Rac*–methyl (*R*)–4–((3*S*,6*S*)–6–ethyl–2–oxotetrahydro–2*H*–pyran–3–yl)–2–(3–methylbenzyl) butanoate (3m)**

^{1}H NMR (400 MHz, MeOD–d_4)

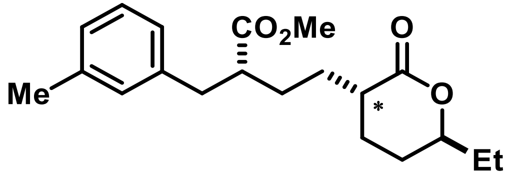

2:1 d.r.

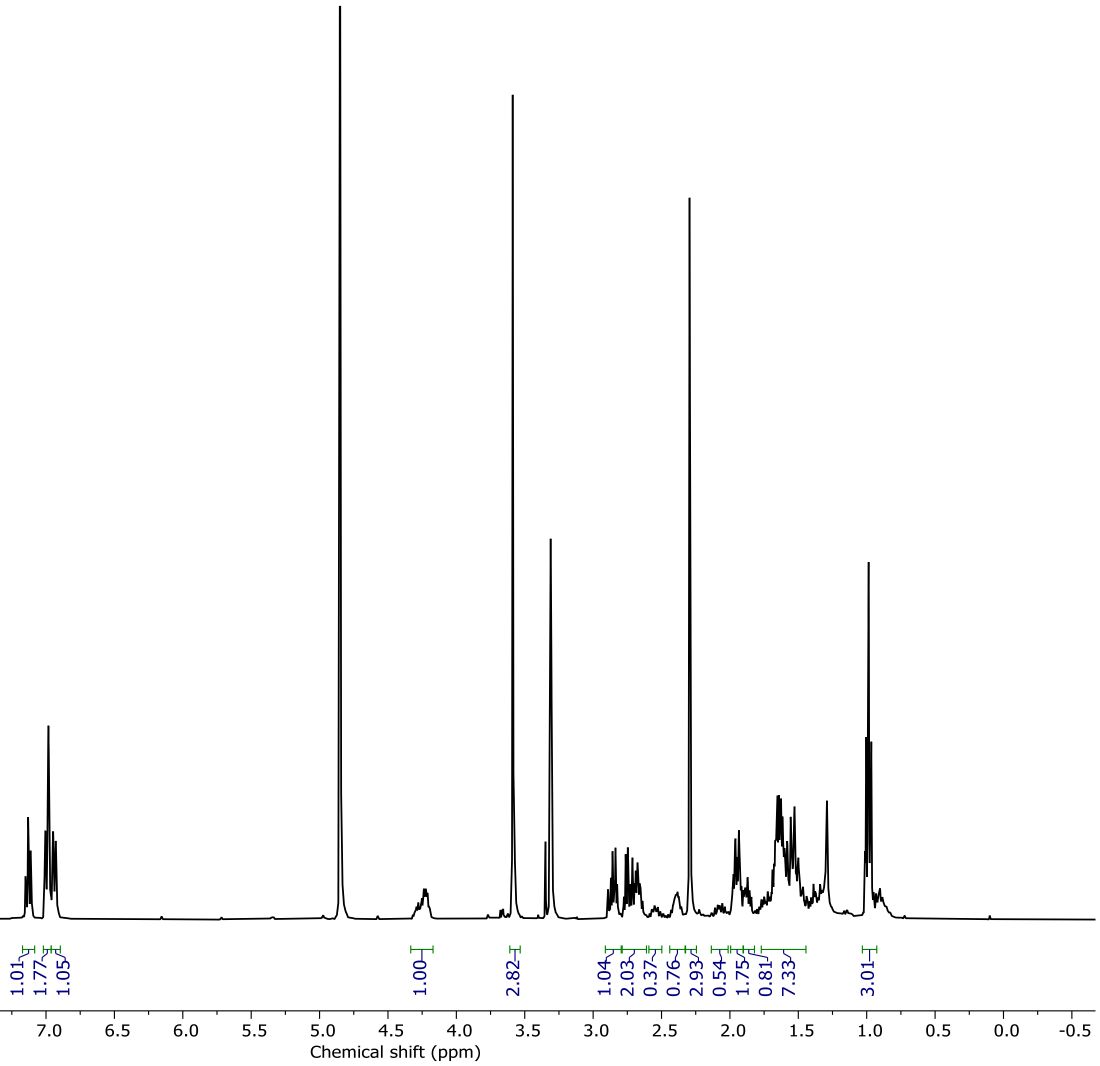

^{13}C NMR (400 MHz, MeOD–d_4)

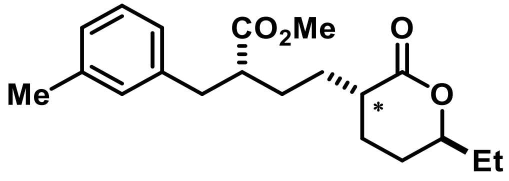

2:1 d.r.

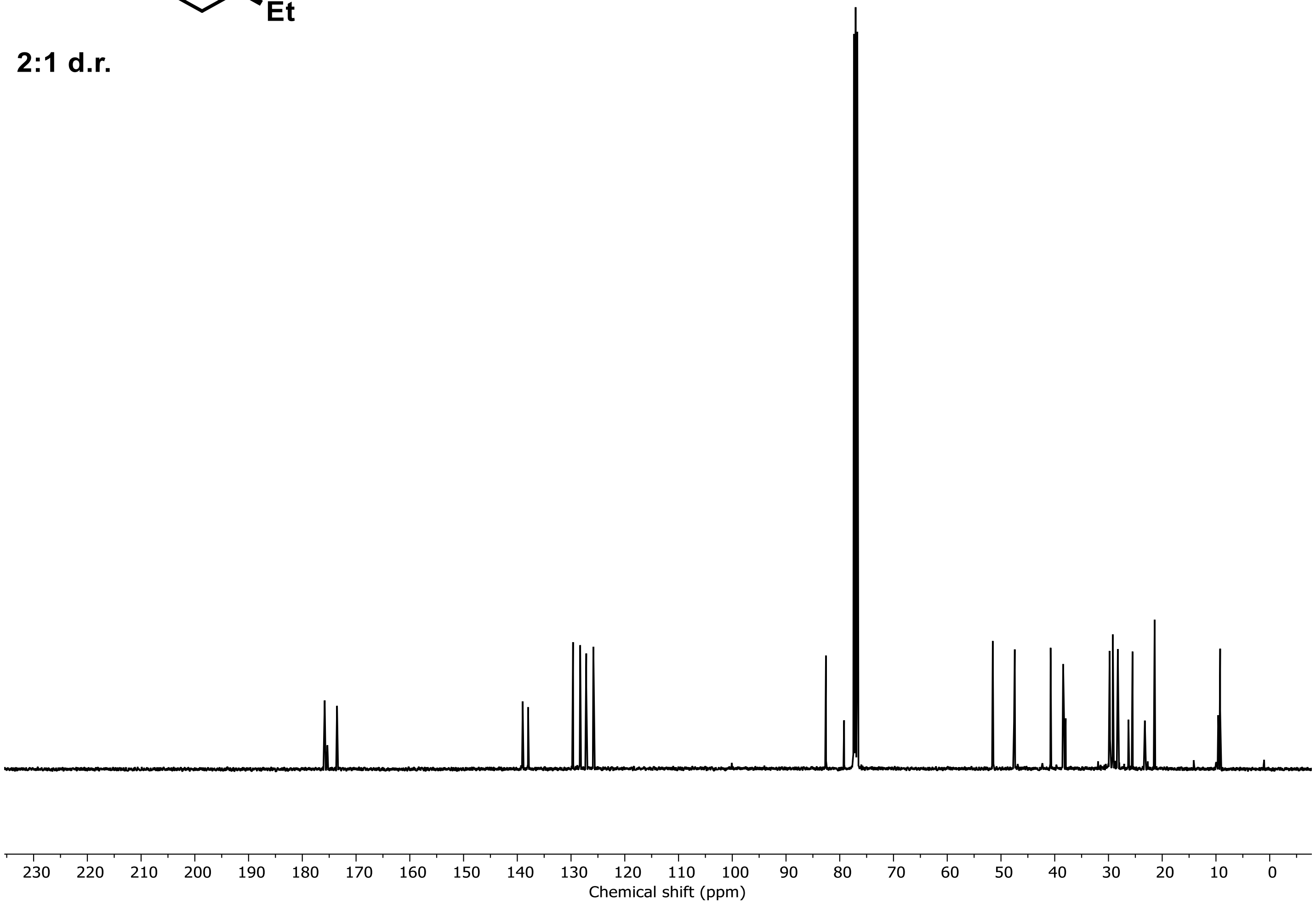

***Rac*–methyl (*R*)–2–(3–chlorobenzyl)–4–((3*S*,6*S*)–6–ethyl–2–oxotetrahydro–2*H*–pyran–3–yl) butanoate (3n)**

^{1}H NMR (400 MHz, $CDCl_3$)

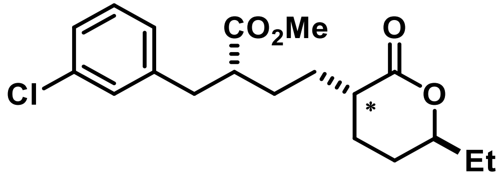

3.9:1 d.r.

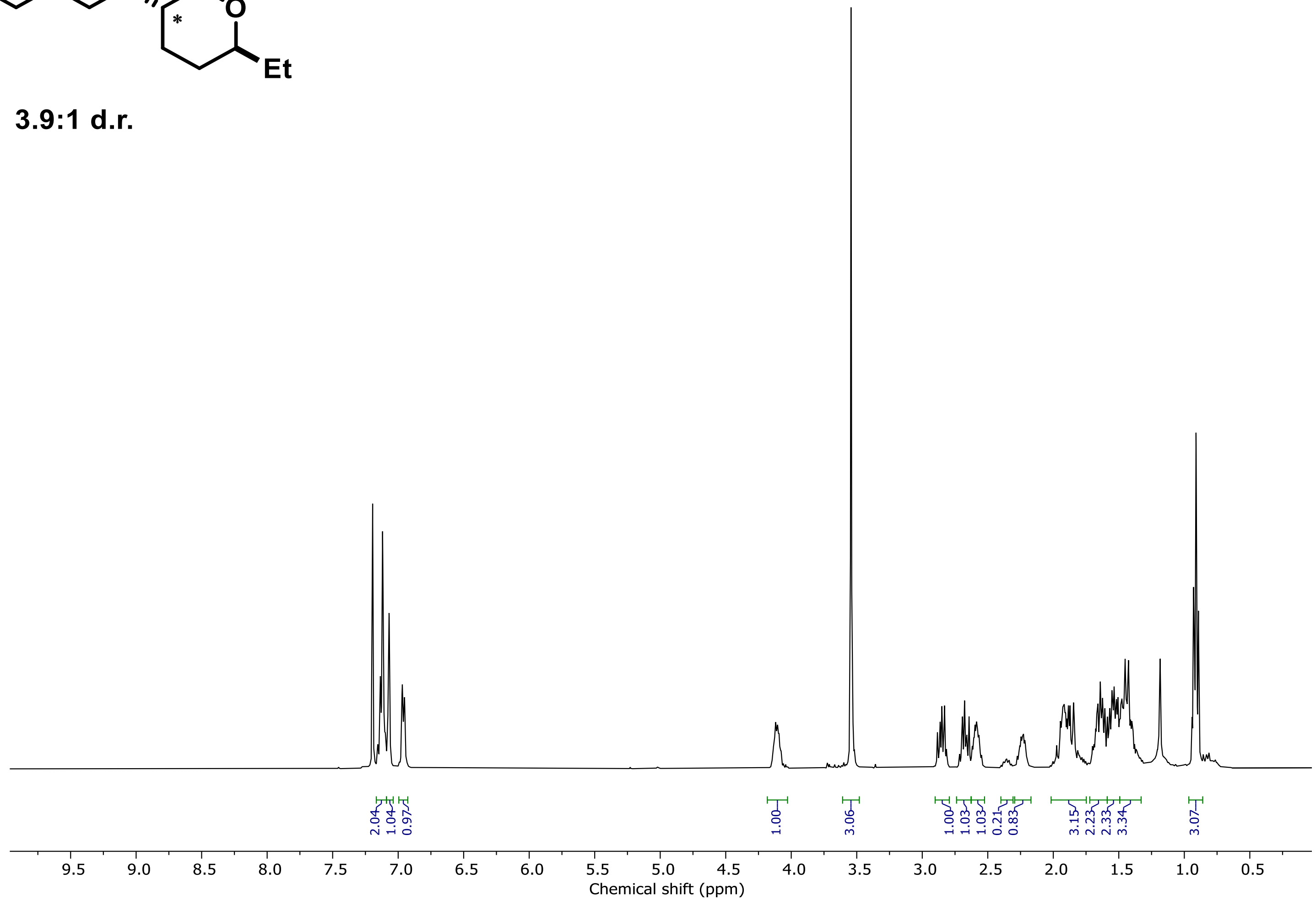

^{13}C NMR (101 MHz, $CDCl_3$)

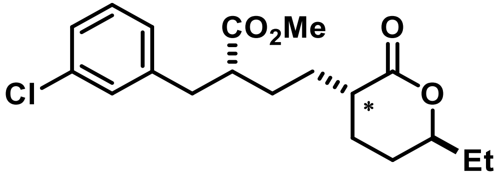

3.9:1 d.r.

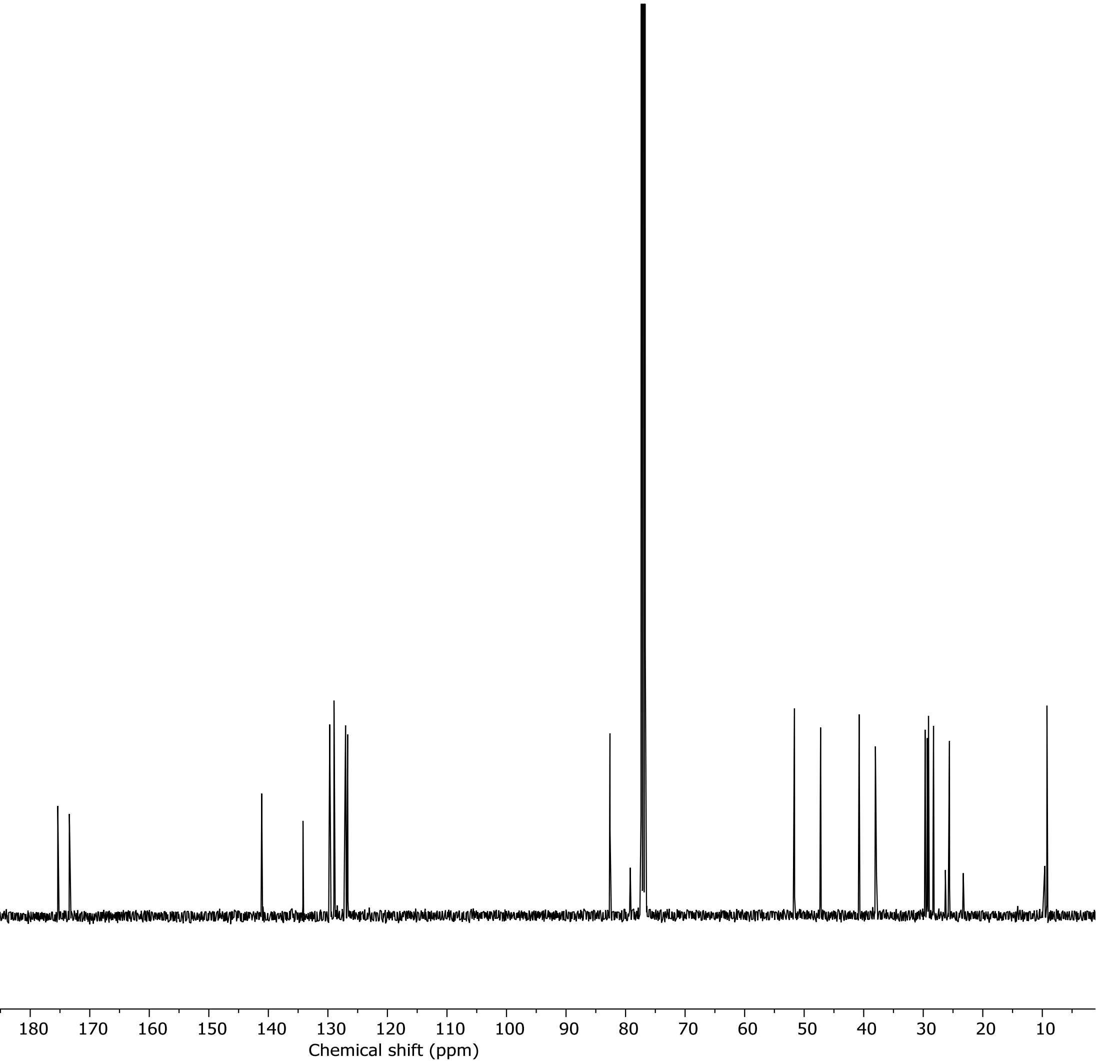

***Rac*–methyl (*R*)–4–((3*S*,6*S*)–6–ethyl–2–oxotetrahydro–2*H*–pyran–3–yl)–2–(naphthalen–1–ylmethyl)butanoate (3o)**

^{1}H NMR (400 MHz, $CDCl_3$)

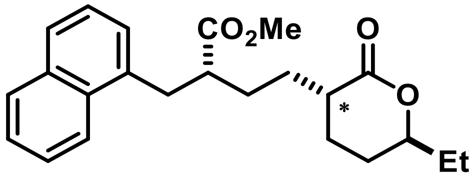

2.7:1 d.r.

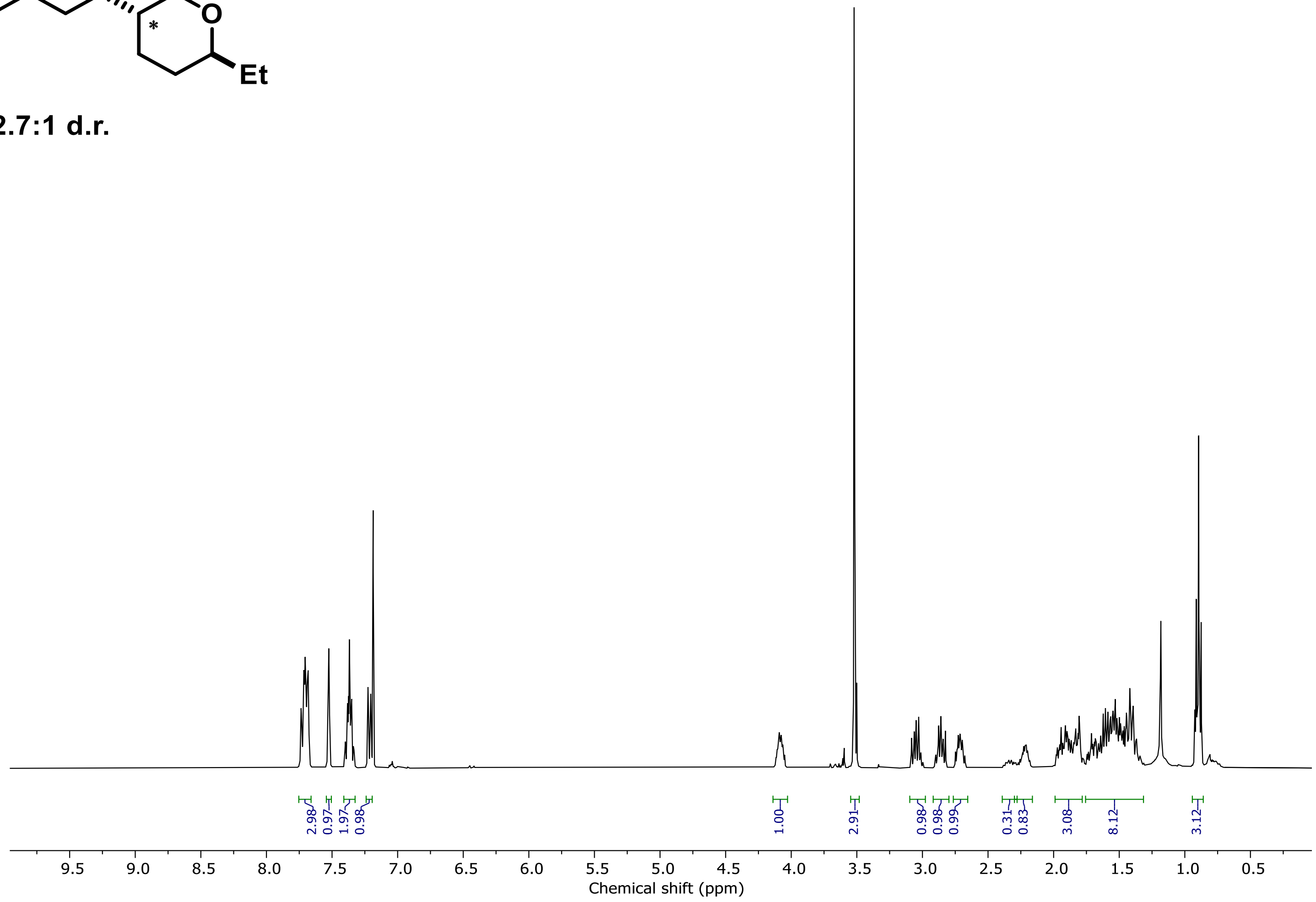

^{13}C NMR (101 MHz, $CDCl_3$)

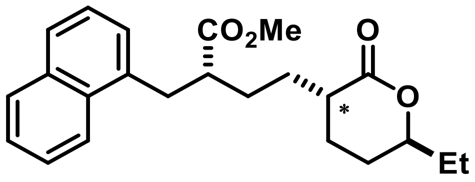

2.7:1 d.r.

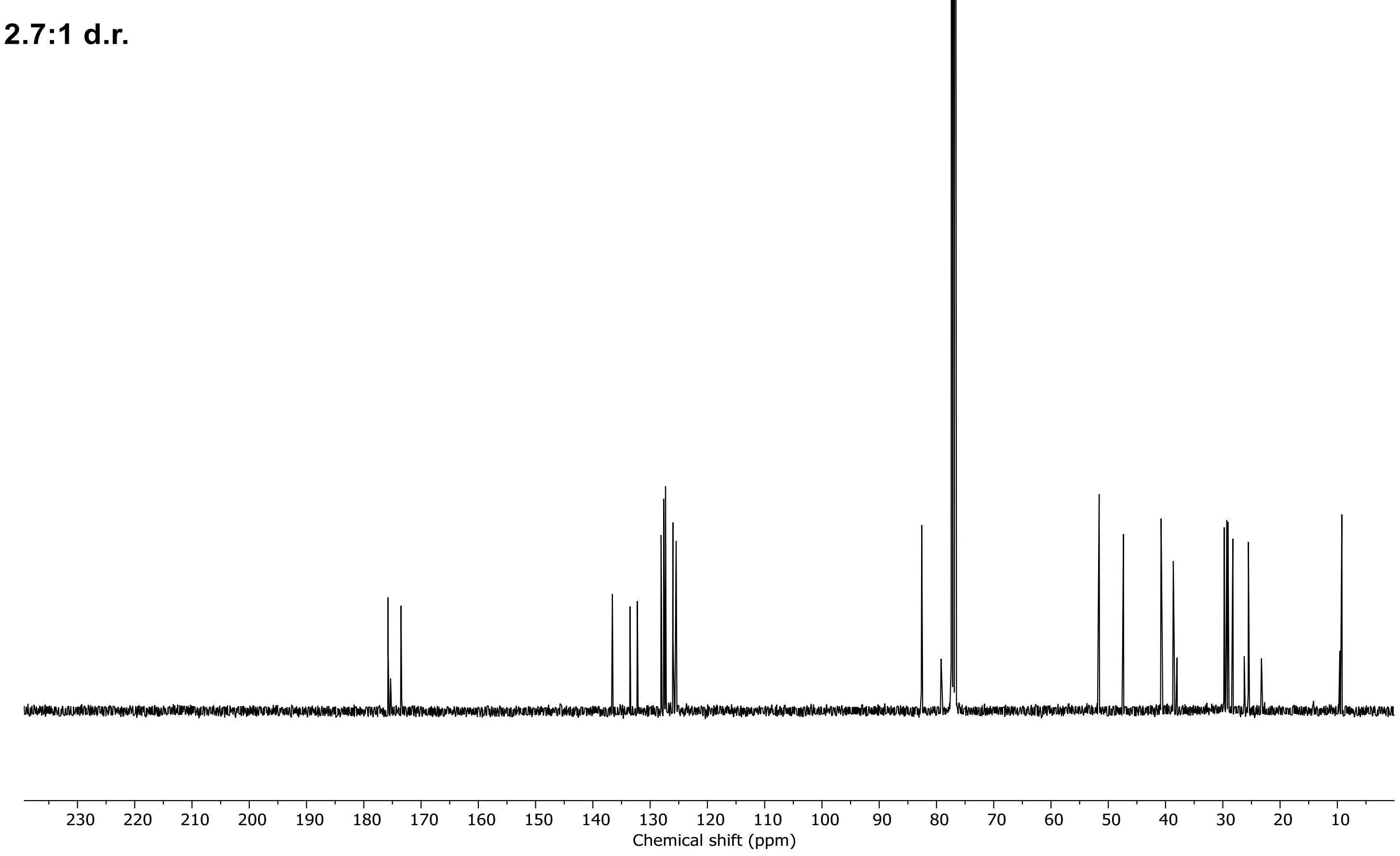

***Rac*–methyl (*R*)–2–([1,1'–biphenyl]–4–ylmethyl)–4–((3*S*,6*S*)–6–ethyl–2–oxotetrahydro–2*H*–pyran–3–yl)butanoate (3p)**

400 MHz, $CDCl_3$

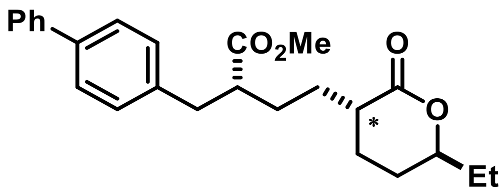

2:1 d.r.

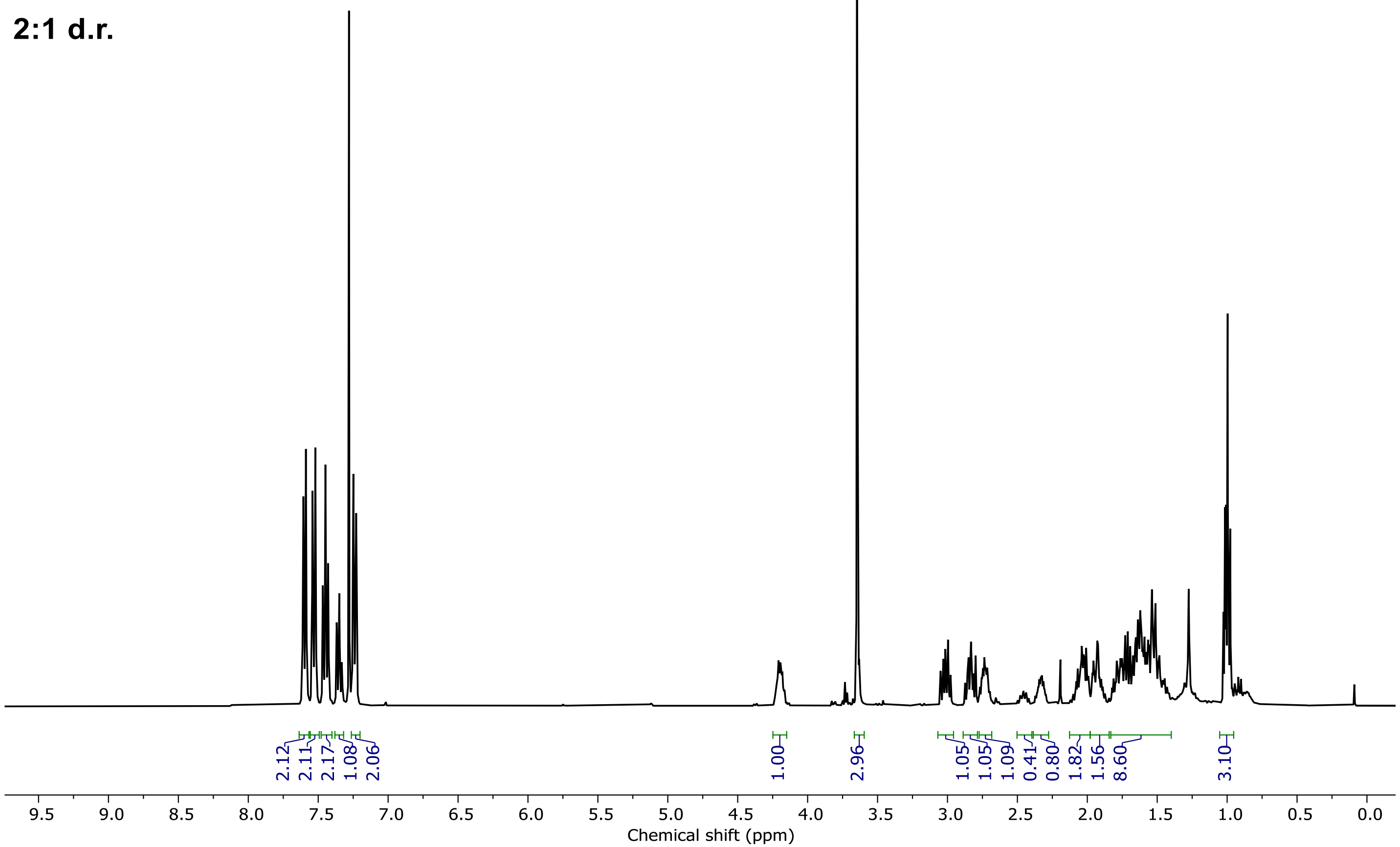

^{13}C NMR (101 MHz, $CDCl_3$)

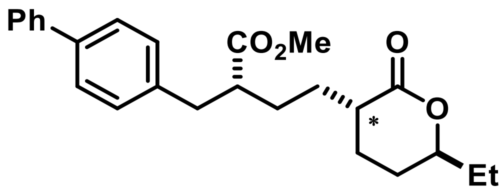

2:1 d.r.

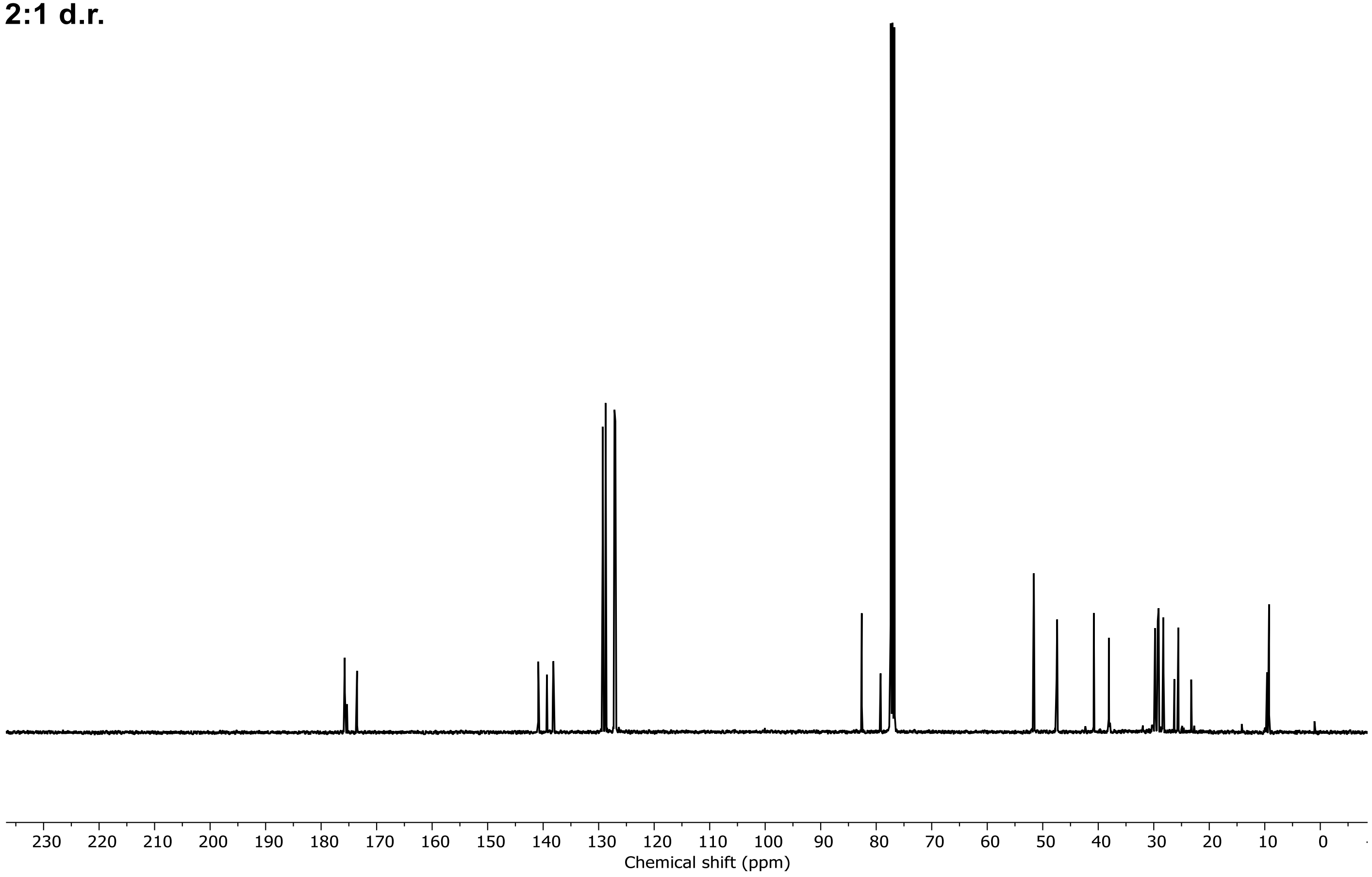

***Rac*–methyl (*R*)–4–((3*S*,6*S*)–6–ethyl–2–oxotetrahydro–2*H*–pyran–3–yl)–2–(4–fluorobenzyl) butanoate (3q)**

^{1}H NMR (400 MHz, $CDCl_3$)

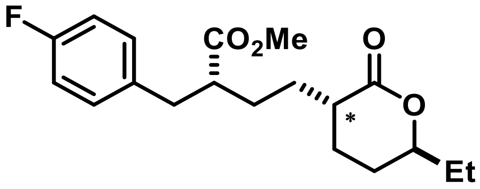

3.3:1 d.r.

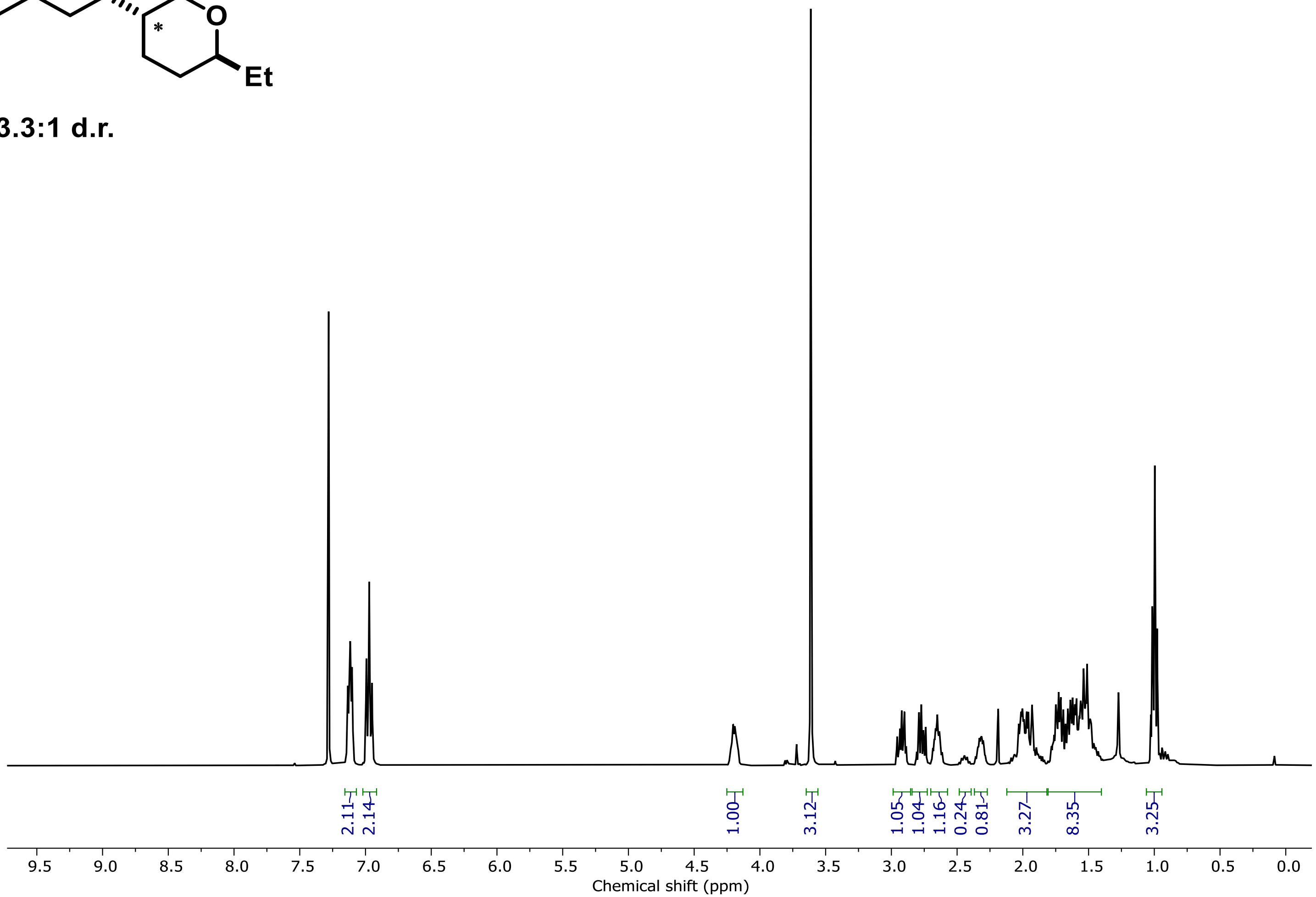

^{13}C NMR (101 MHz, $CDCl_3$)

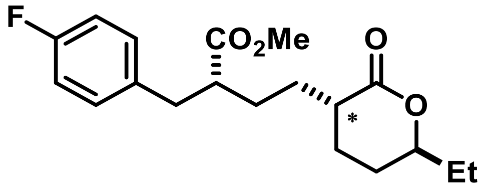

3.3:1 d.r.

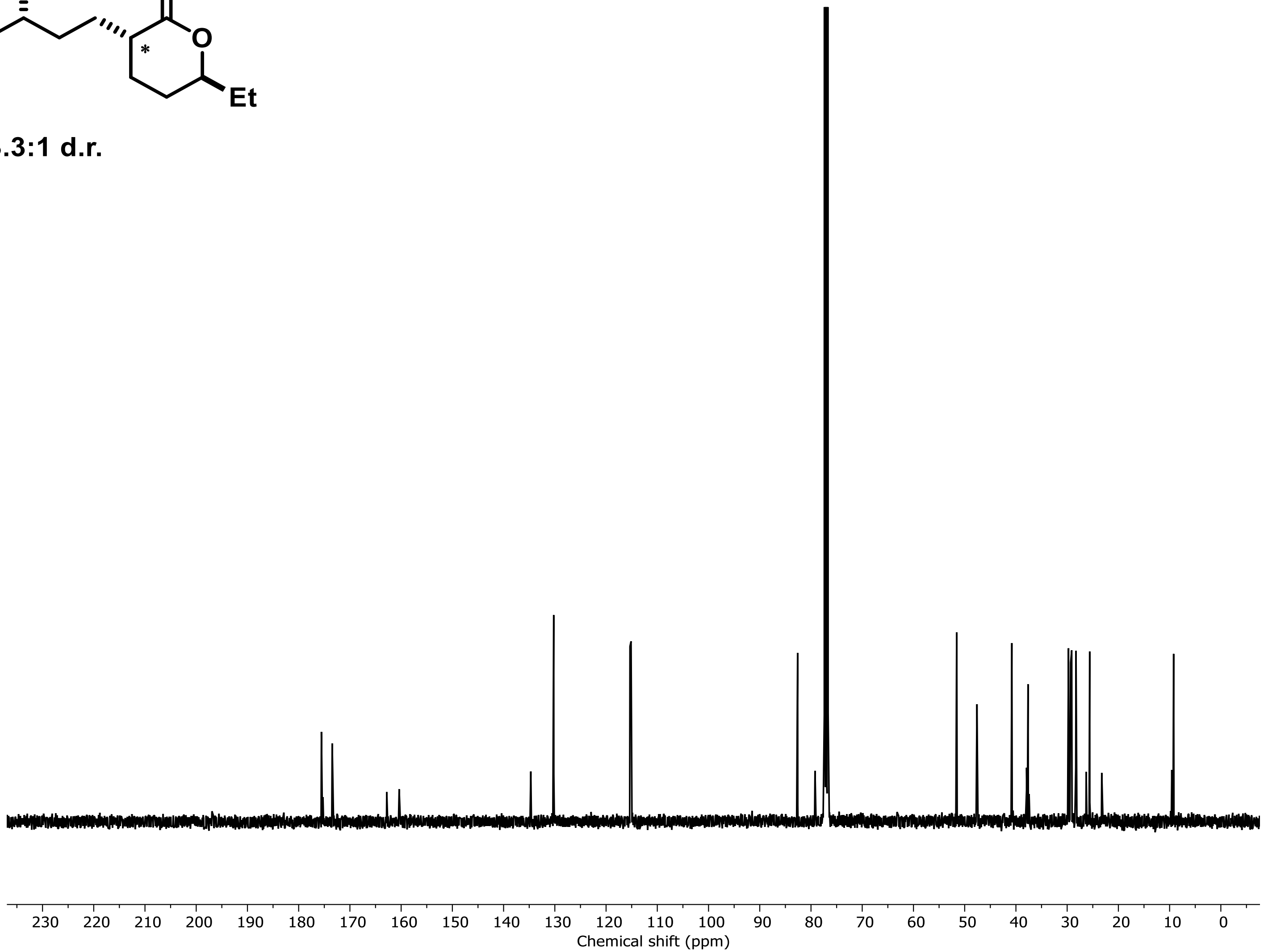

^{19}F NMR (471 MHz, $CDCl_3$)

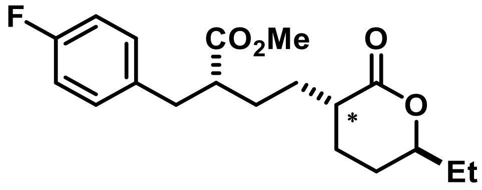

3.3:1 d.r.

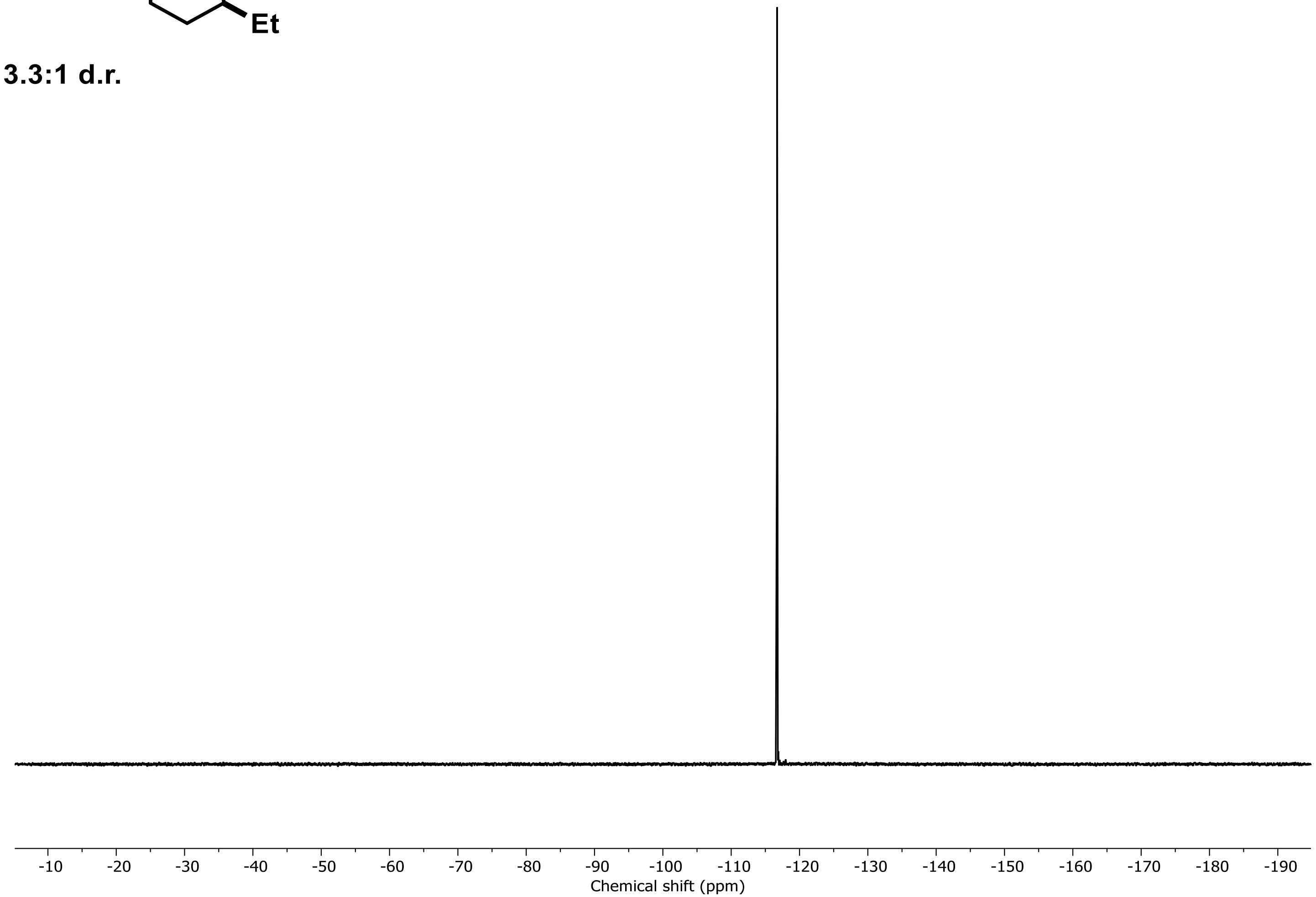

***Rac*–methyl (*R*)–2–(4–bromobenzyl)–4–((3*S*,6*S*)–6–ethyl–2–oxotetrahydro–2*H*–pyran–3–yl) butanoate (3r)**

^{1}H NMR (500 MHz, $CDCl_3$)

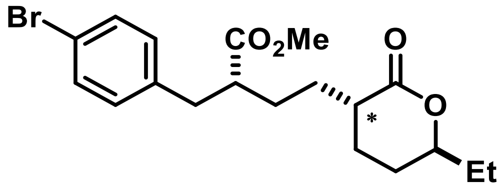

2.5:1 d.r.

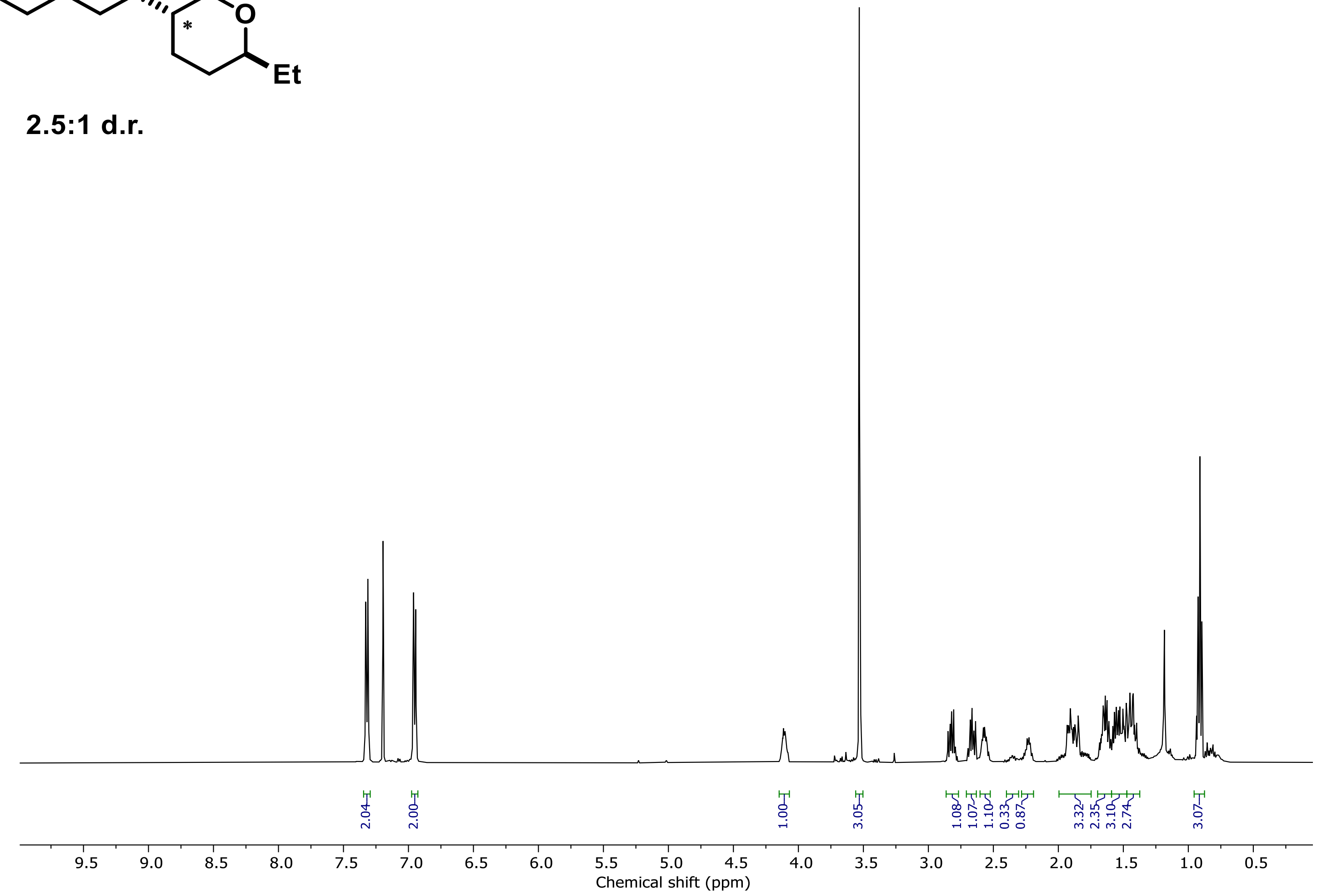

^{13}C NMR (126 MHz, $CDCl_3$)

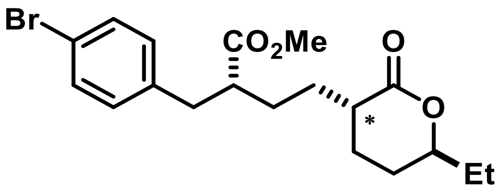

2.5:1 d.r.

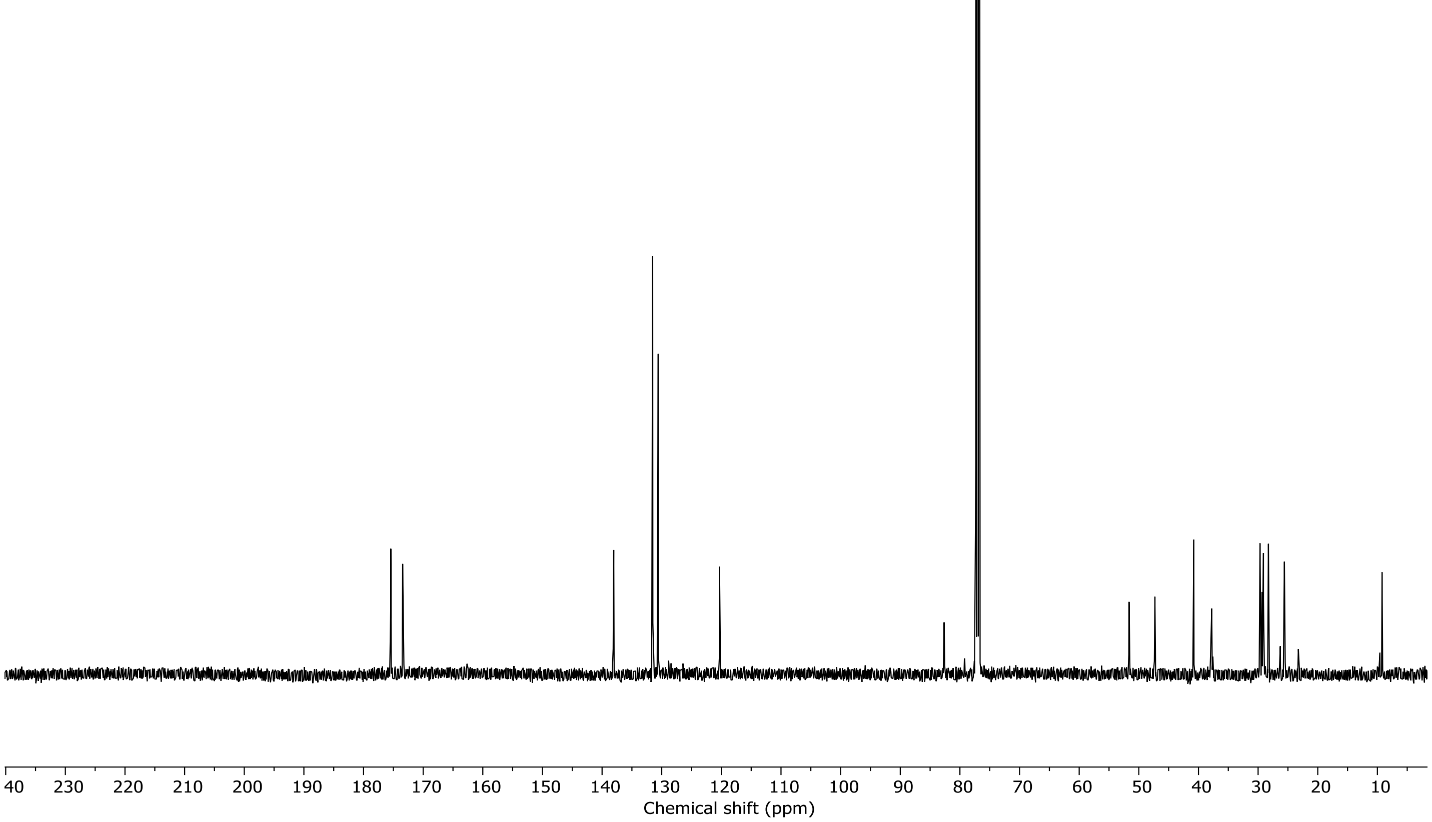

***Rac*–methyl (*R*)–4–((3*S*,6*S*)–6–ethyl–2–oxotetrahydro–2*H*–pyran–3–yl)–2–(4–(trifluoromethyl)benzyl)butanoate (3s)**

^{1}H NMR (400 MHz, $CDCl_3$)

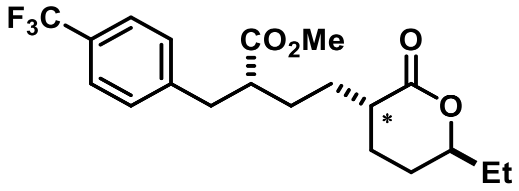

2.7:1 d.r.

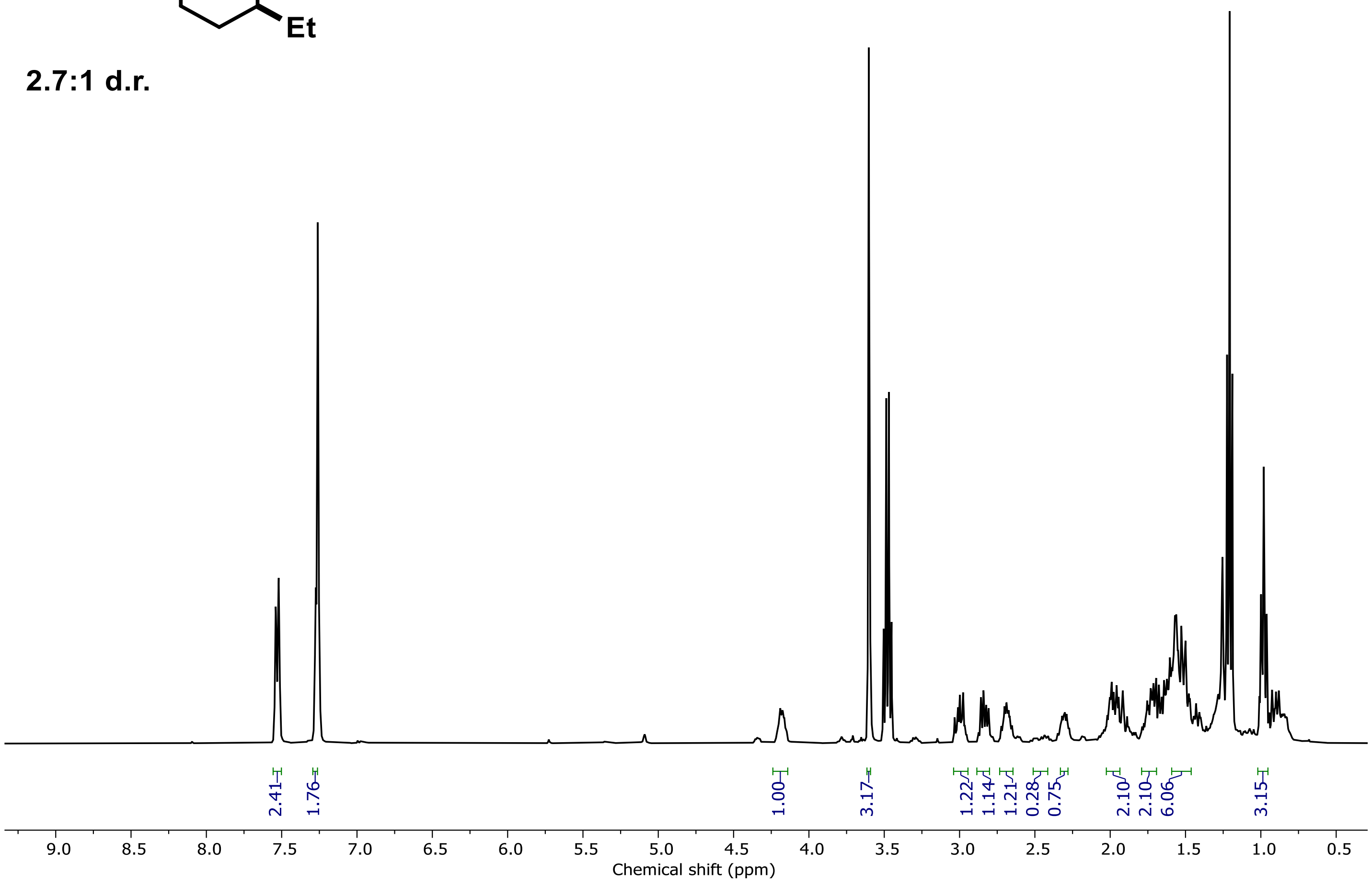

^{13}C NMR (101 MHz, $CDCl_3$)

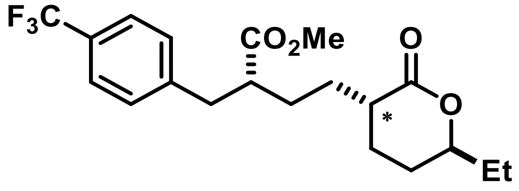

2.7:1 d.r.

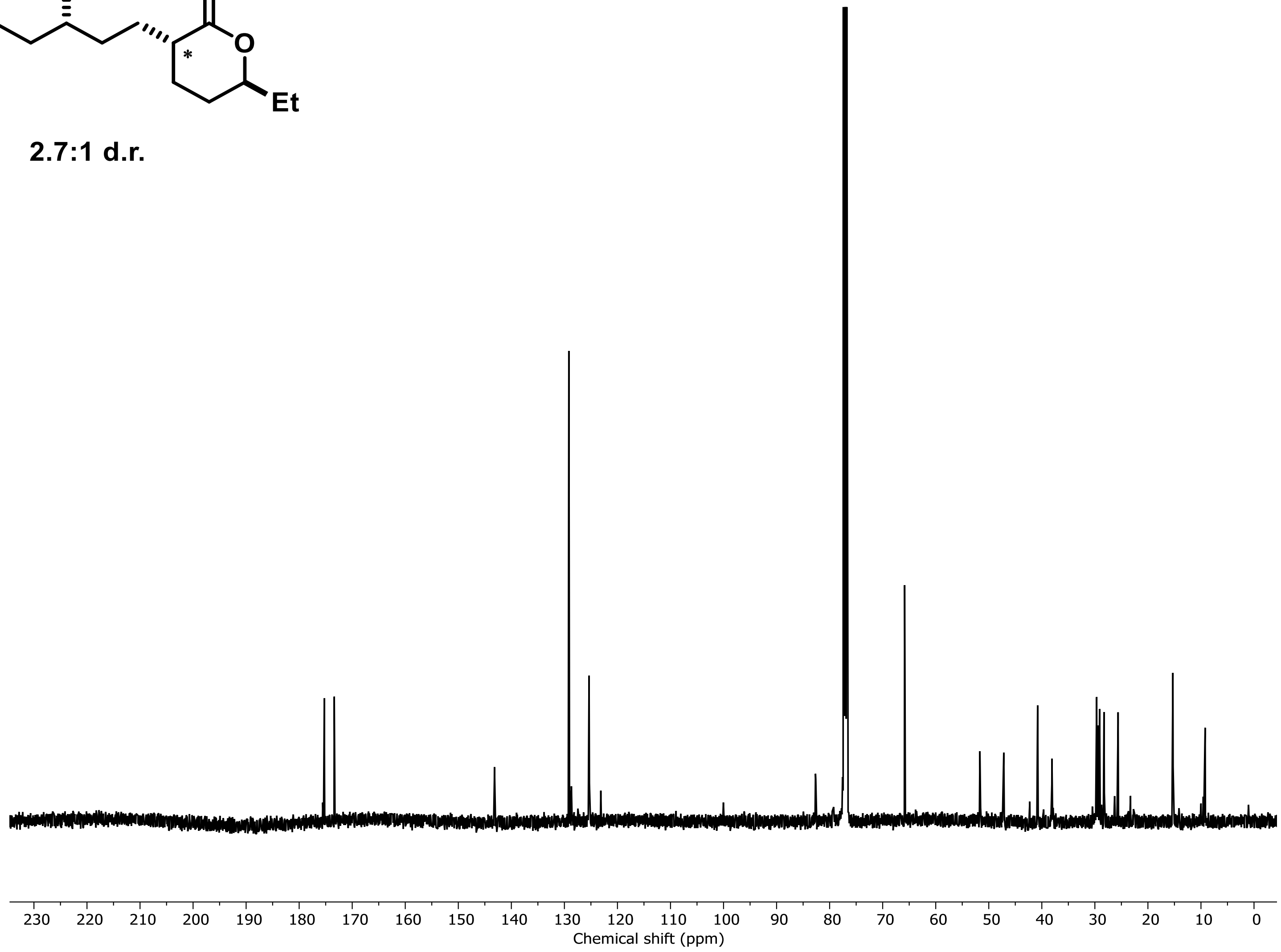

^{19}F NMR (471 MHz, $CDCl_3$)

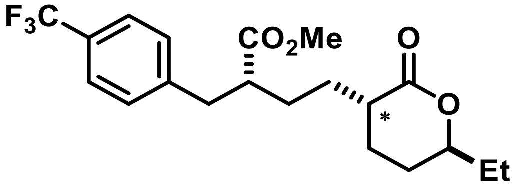

2.7:1 d.r.

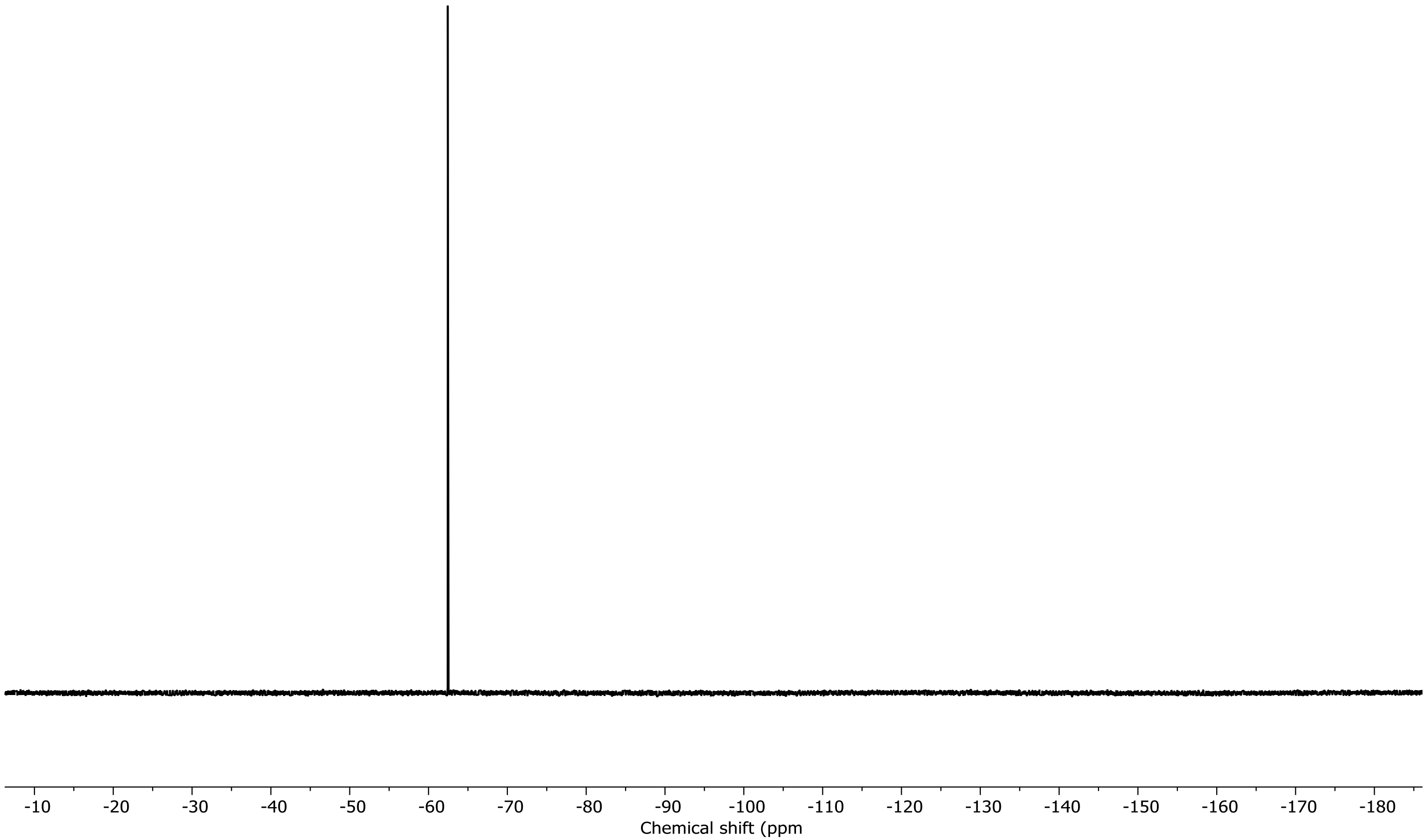

***Rac*–methyl (*R*)–4–((3*S*,6*S*)–6–ethyl–2–oxotetrahydro–2*H*–pyran–3–yl)–2–(4–methoxybenzyl) butanoate (3t)**

^{1}H NMR (400 MHz, $CDCl_3$)

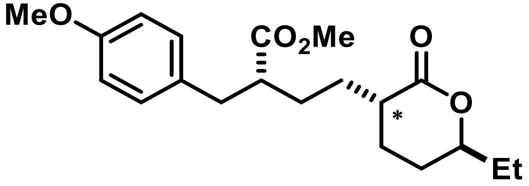

3.7:1 d.r.

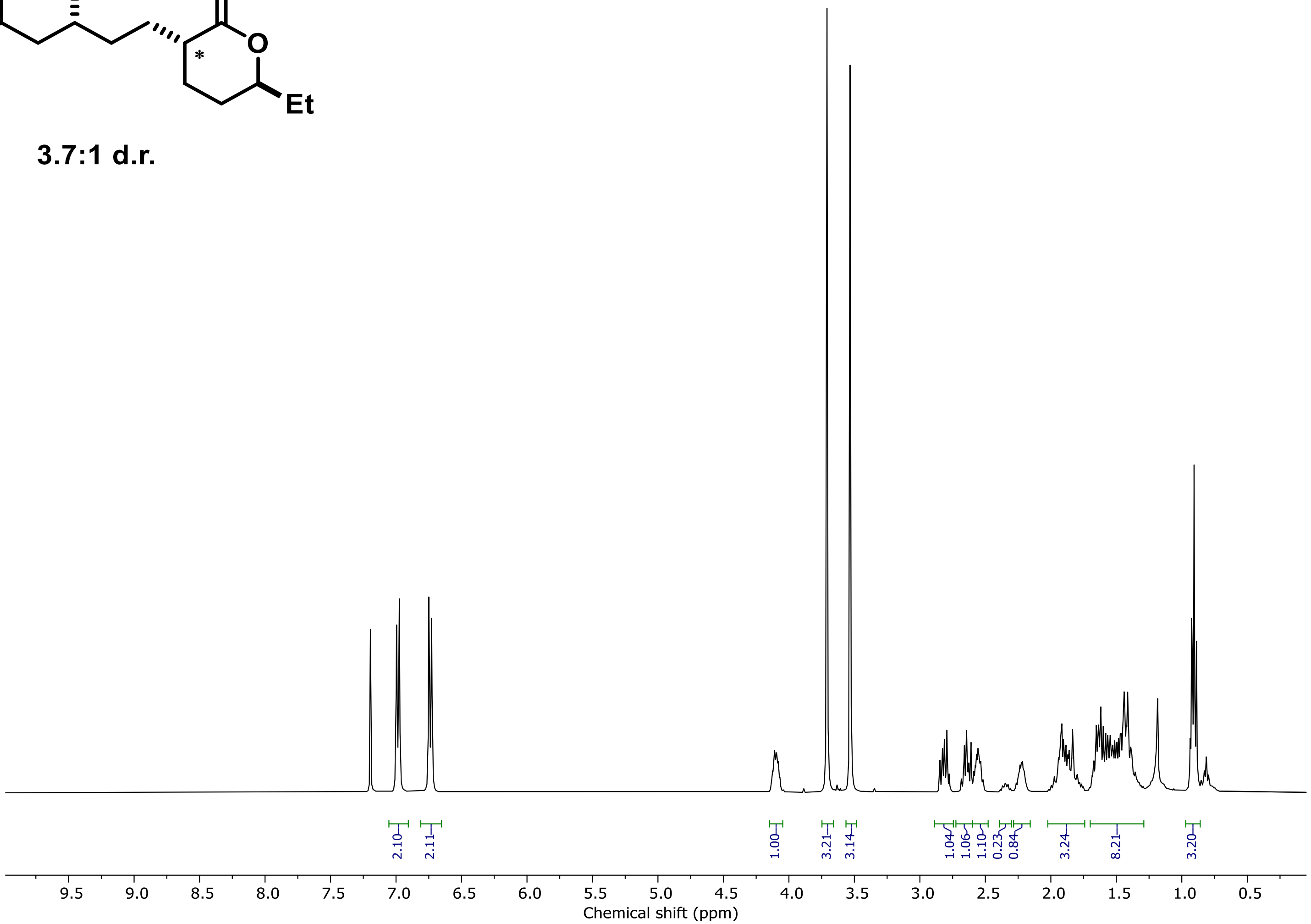

^{13}C NMR (101 MHz, $CDCl_3$)

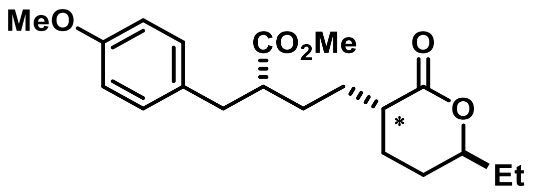

3.7:1 d.r.

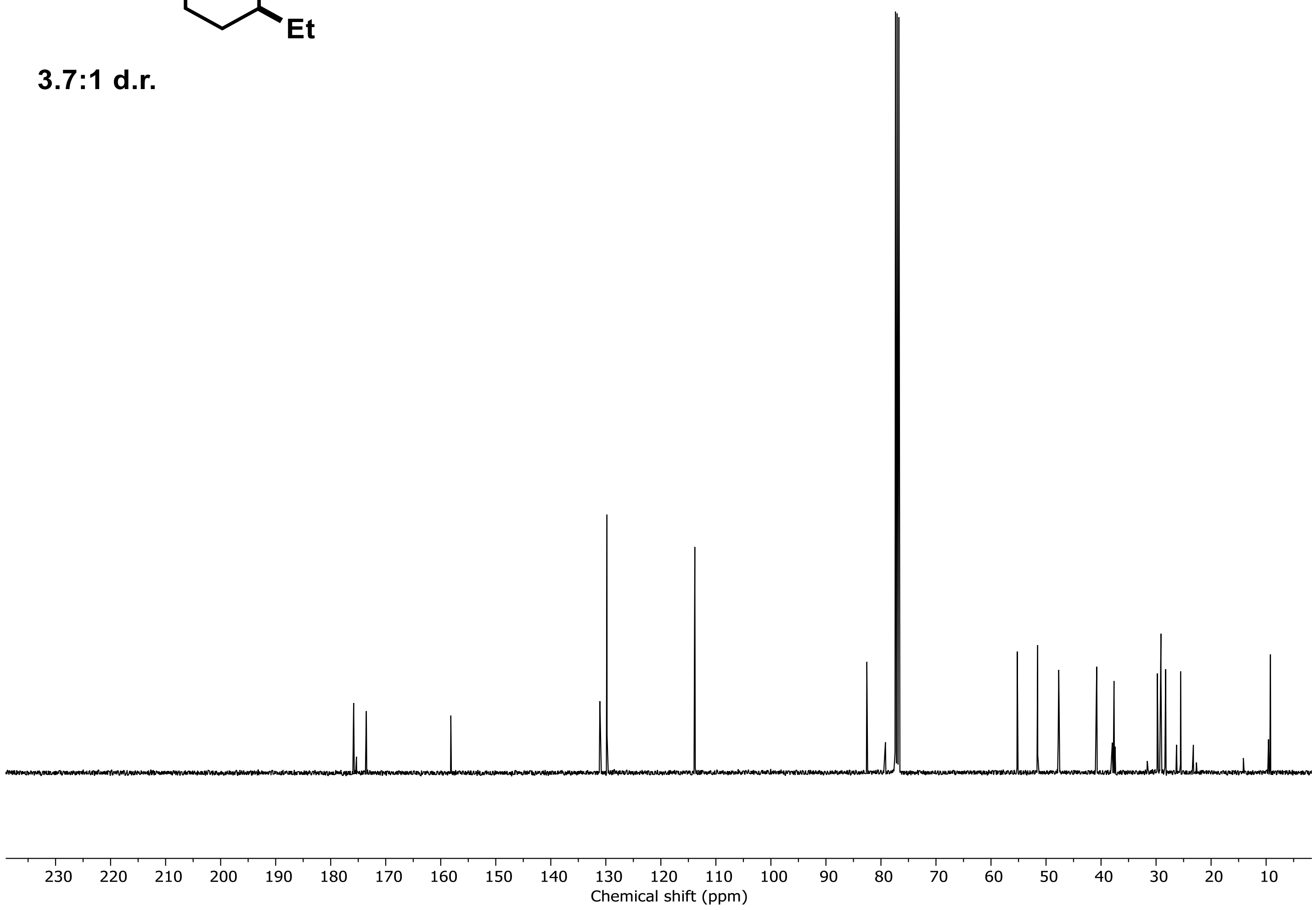

Rac-methyl (*R*)-4-((3*S*,6*S*)-6-methyl-2-oxotetrahydro-2H-pyran-3-yl)-2-(thiophen-2-ylmethyl)butanoate (3u)

^{1}H NMR (400 MHz, $CDCl_3$)

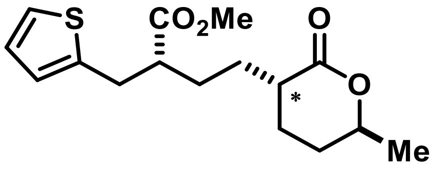

4.3:1 d.r.

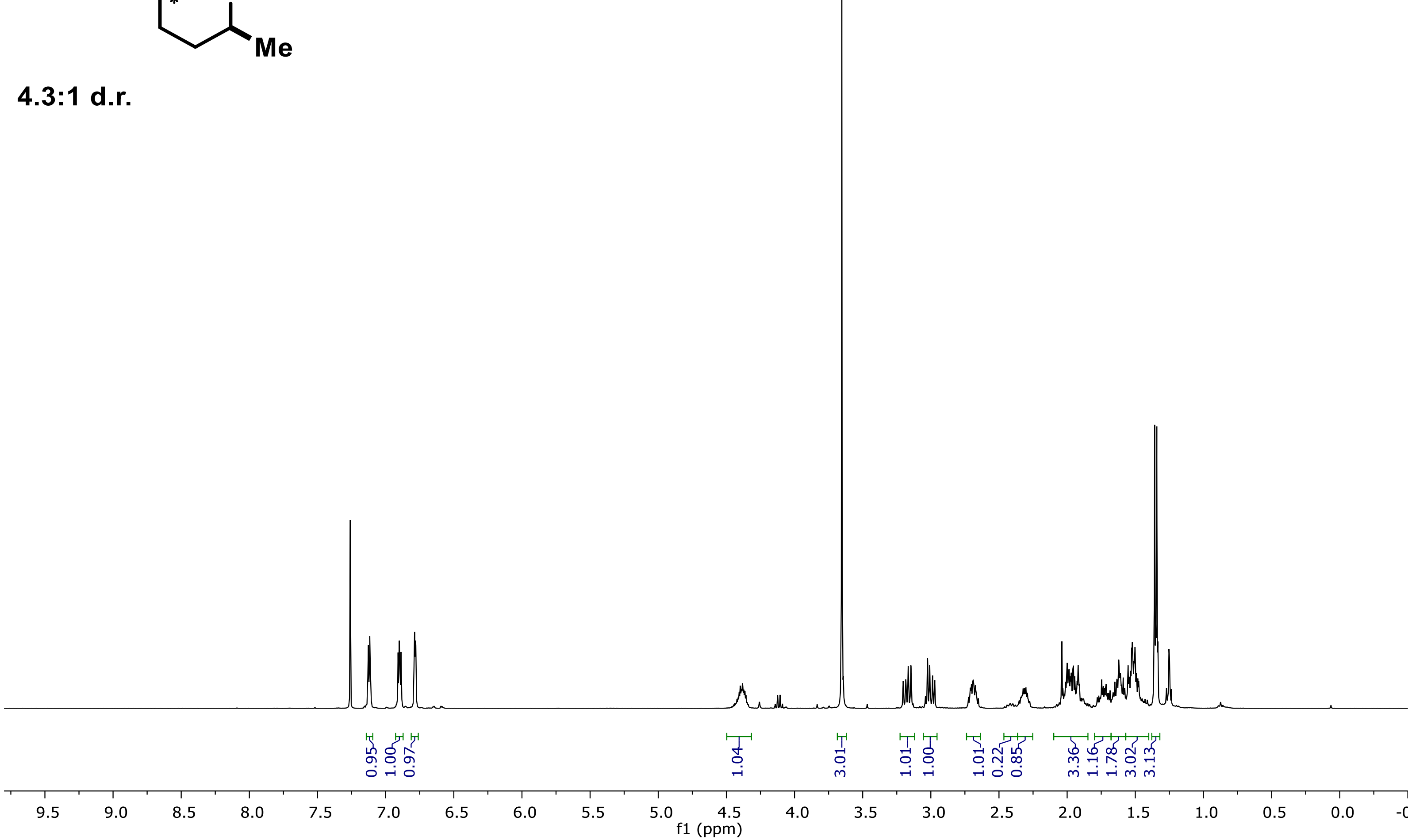

^{13}C NMR (101 MHz, $CDCl_3$)

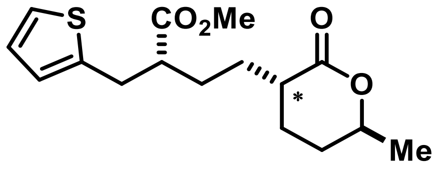

4.3:1 d.r.

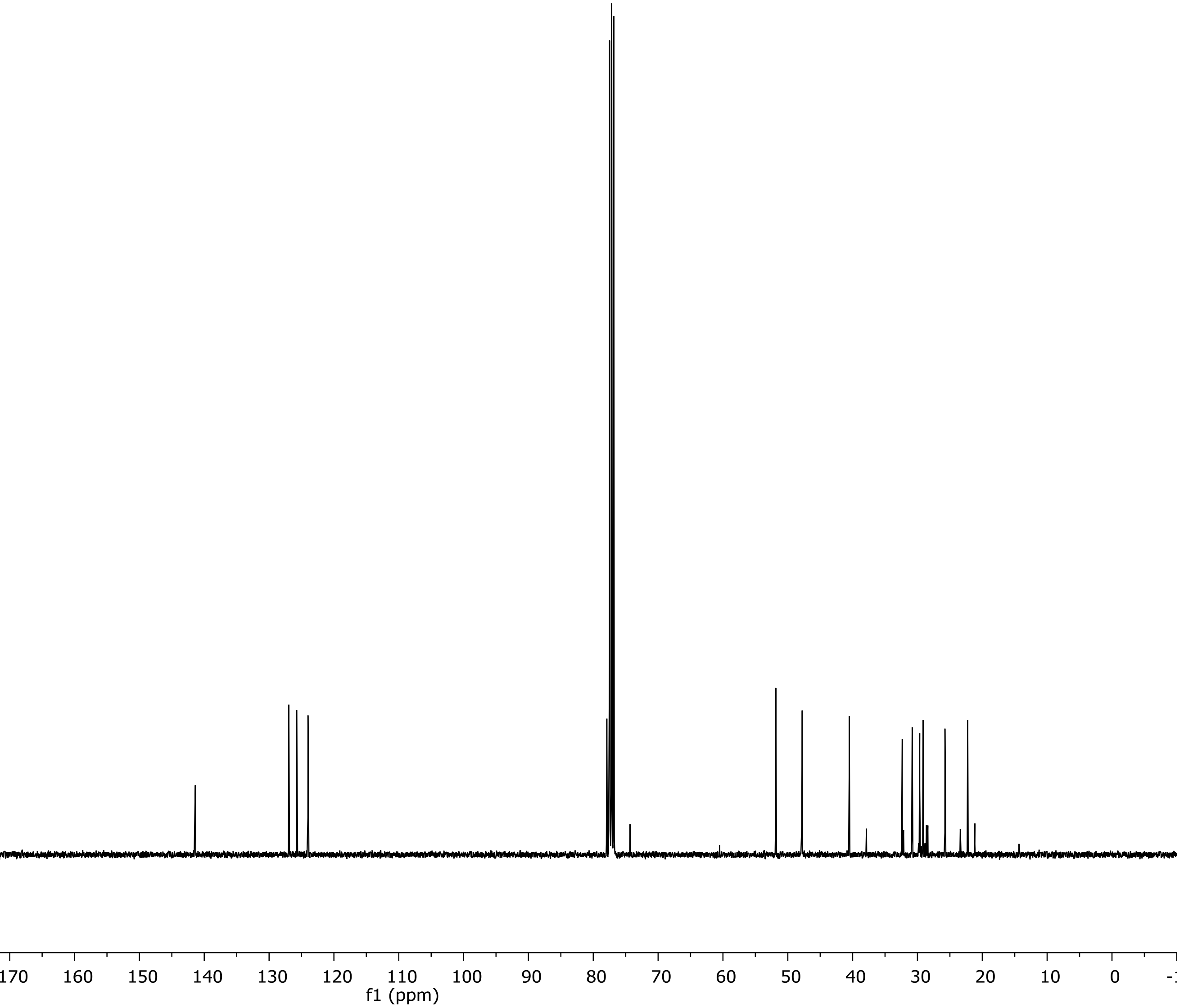

***Rac*-methyl 4-(6-methyl-2-oxotetrahydro-2H-pyran-3-yl)-2-(1-phenylethyl)butanoate (3v)**

^{1}H NMR (400 MHz, $CDCl_3$)

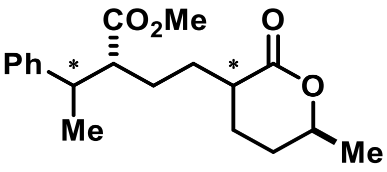

3.2:2.0:1.9:1 d.r.

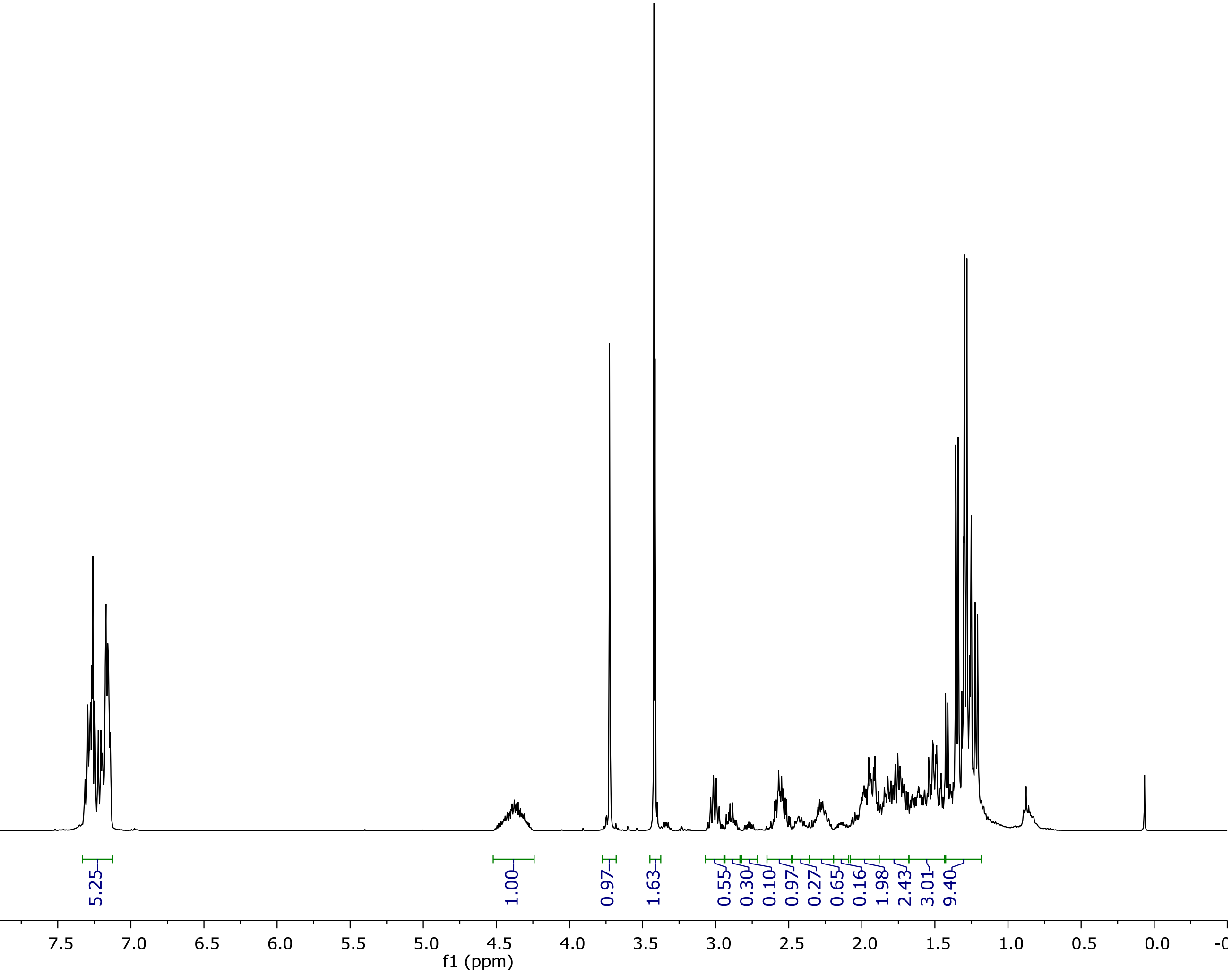

^{13}C NMR (101 MHz, $CDCl_3$)

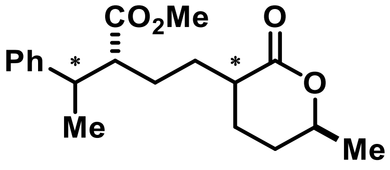

3.2:2.0:1.9:1 d.r.

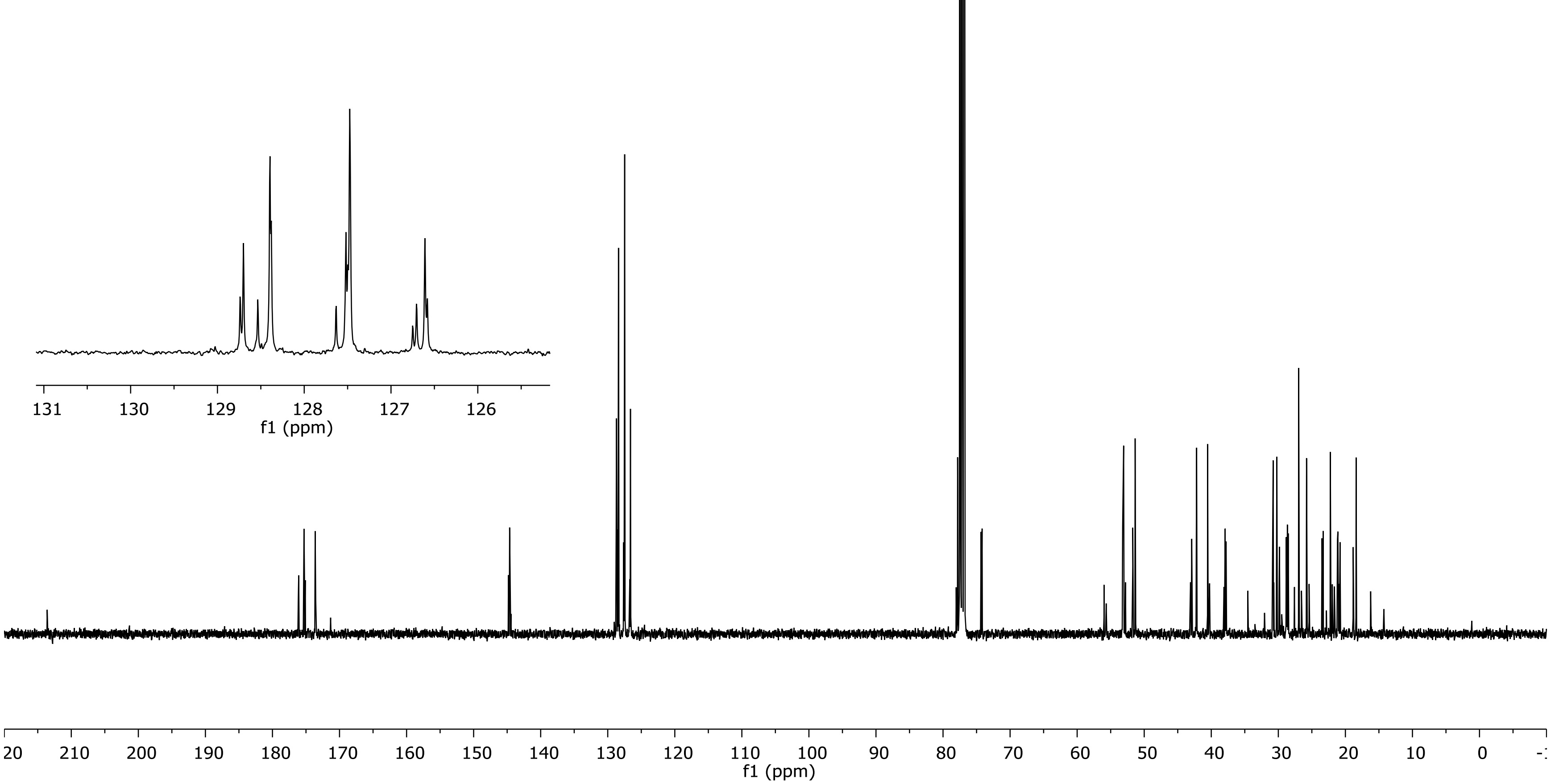

(*E*)–6–Methyl–3–(4–phenylbut–3–en–1–yl)tetrahydro–2*H*–pyran–2–one (S32)

^{1}H NMR (400 MHz, $CDCl_3$)

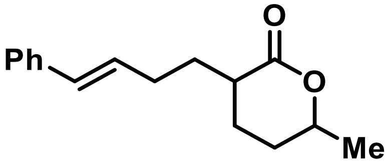

1.2:1 d.r.

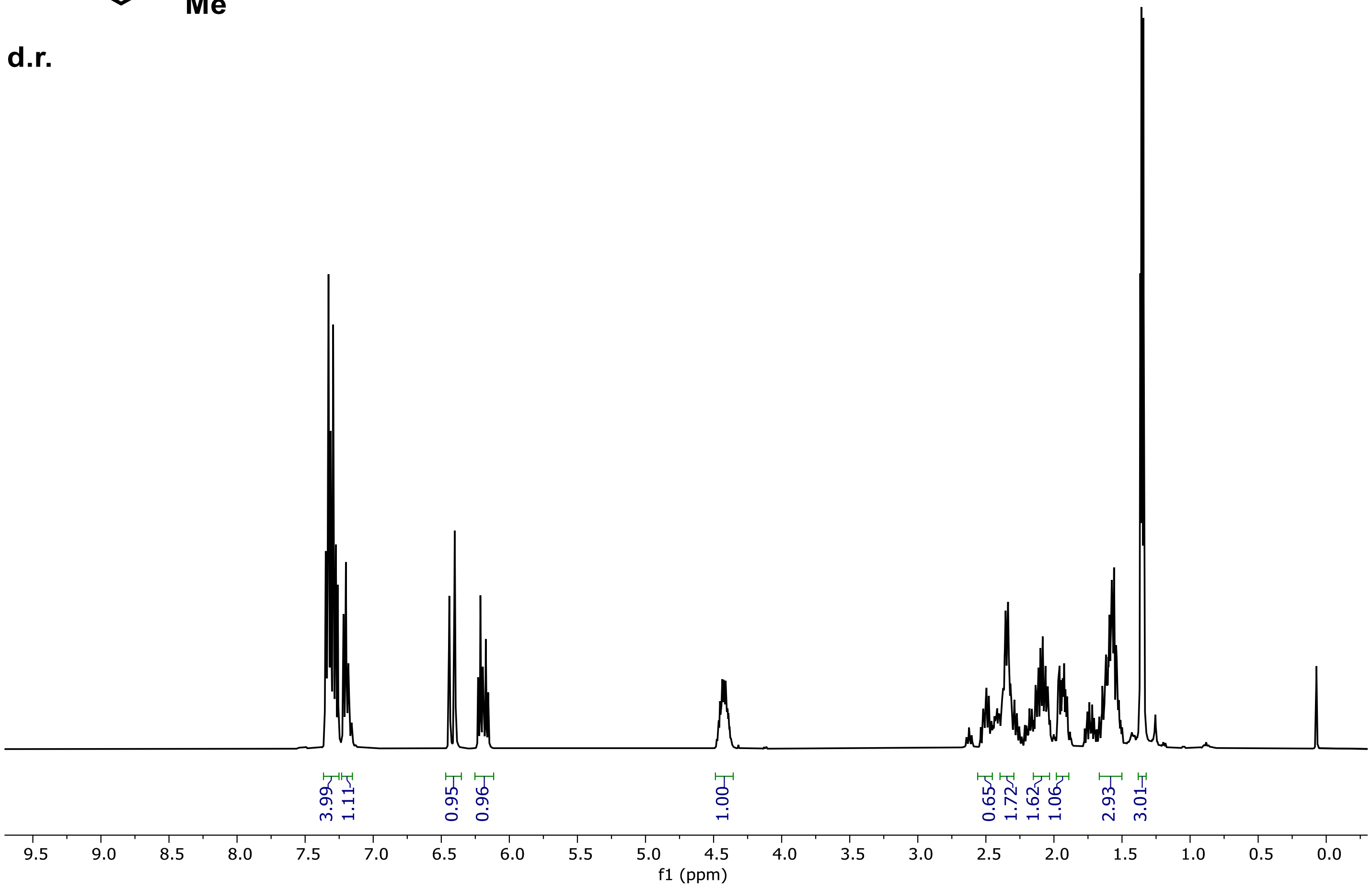

^{13}C NMR (101 MHz, $CDCl_3$)

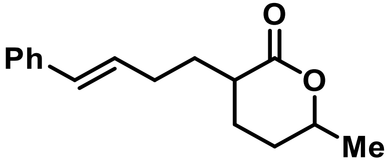

1.2:1 d.r.

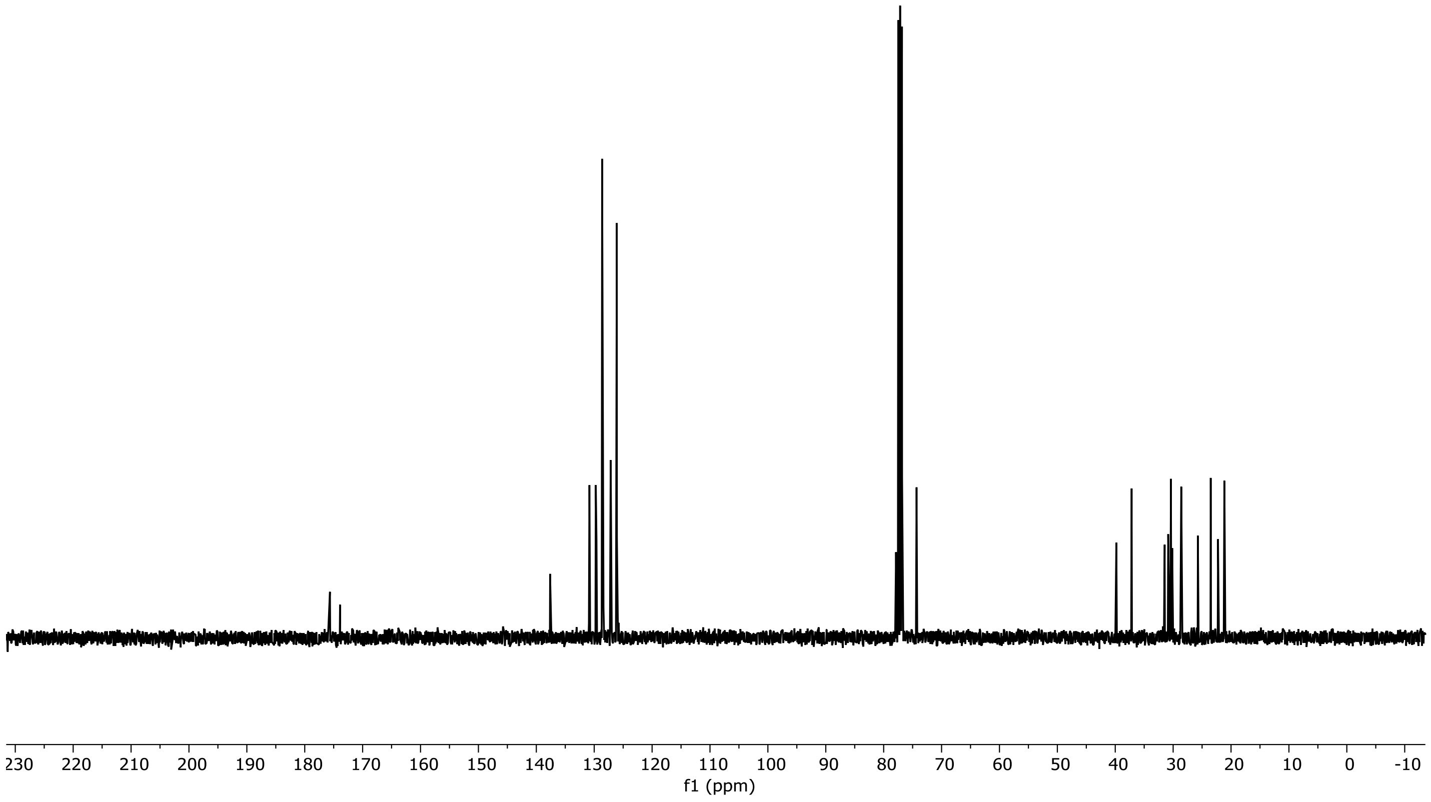

***Rac*–methyl (3*R*,6*S*)–6–methyl–2–oxo–3–((*E*)–4–phenylbut–3–en–1–yl)tetrahydro–2*H*–pyran–3–carboxylate–*^{13}C* (1a–*^{13}C*)**

^{1}H NMR (400 MHz, $CDCl_3$)

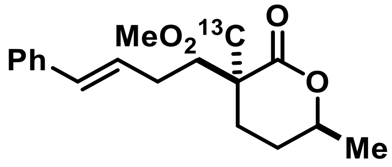

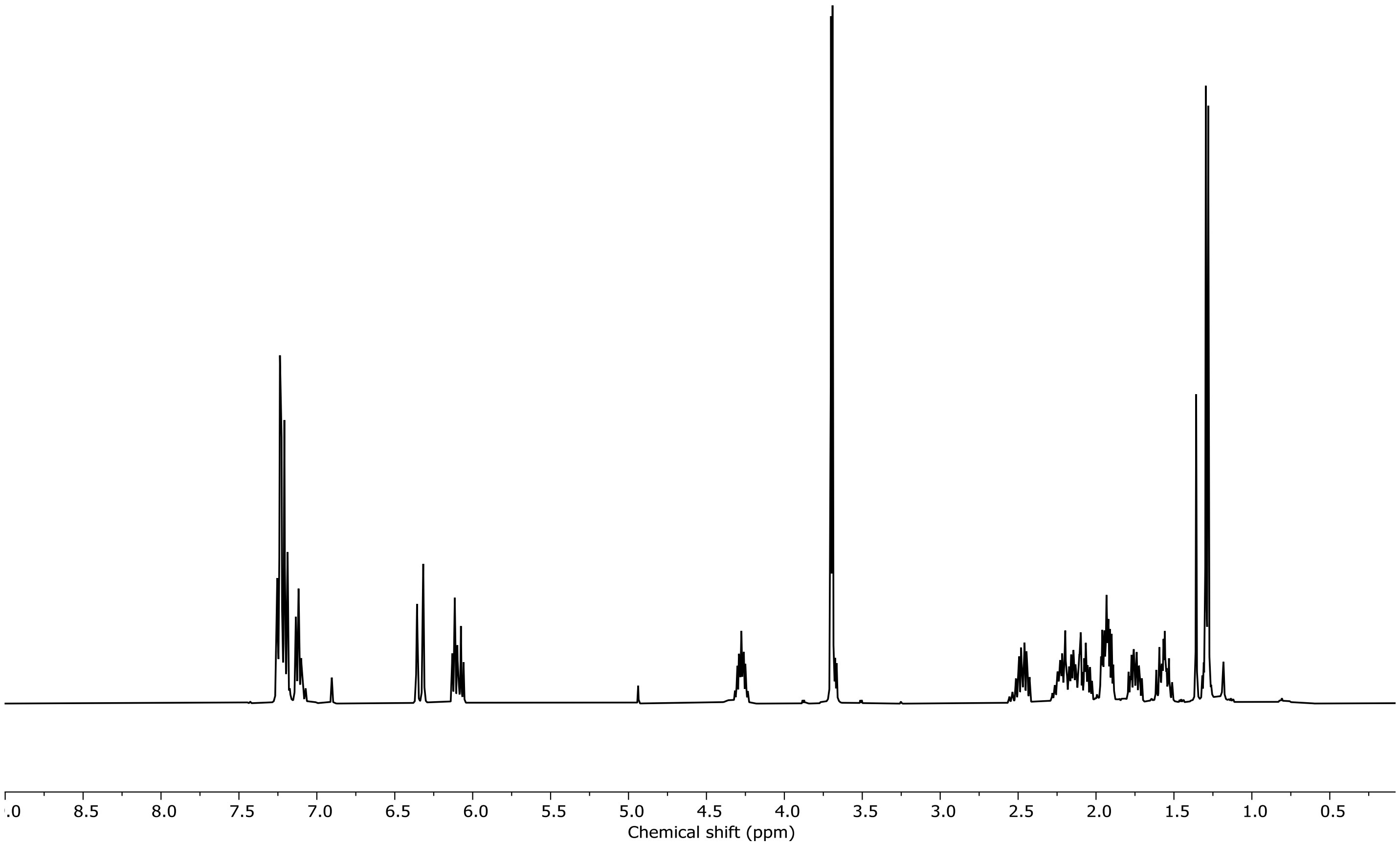

^{13}C NMR (101 MHz, $CDCl_3$)

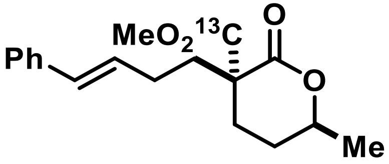

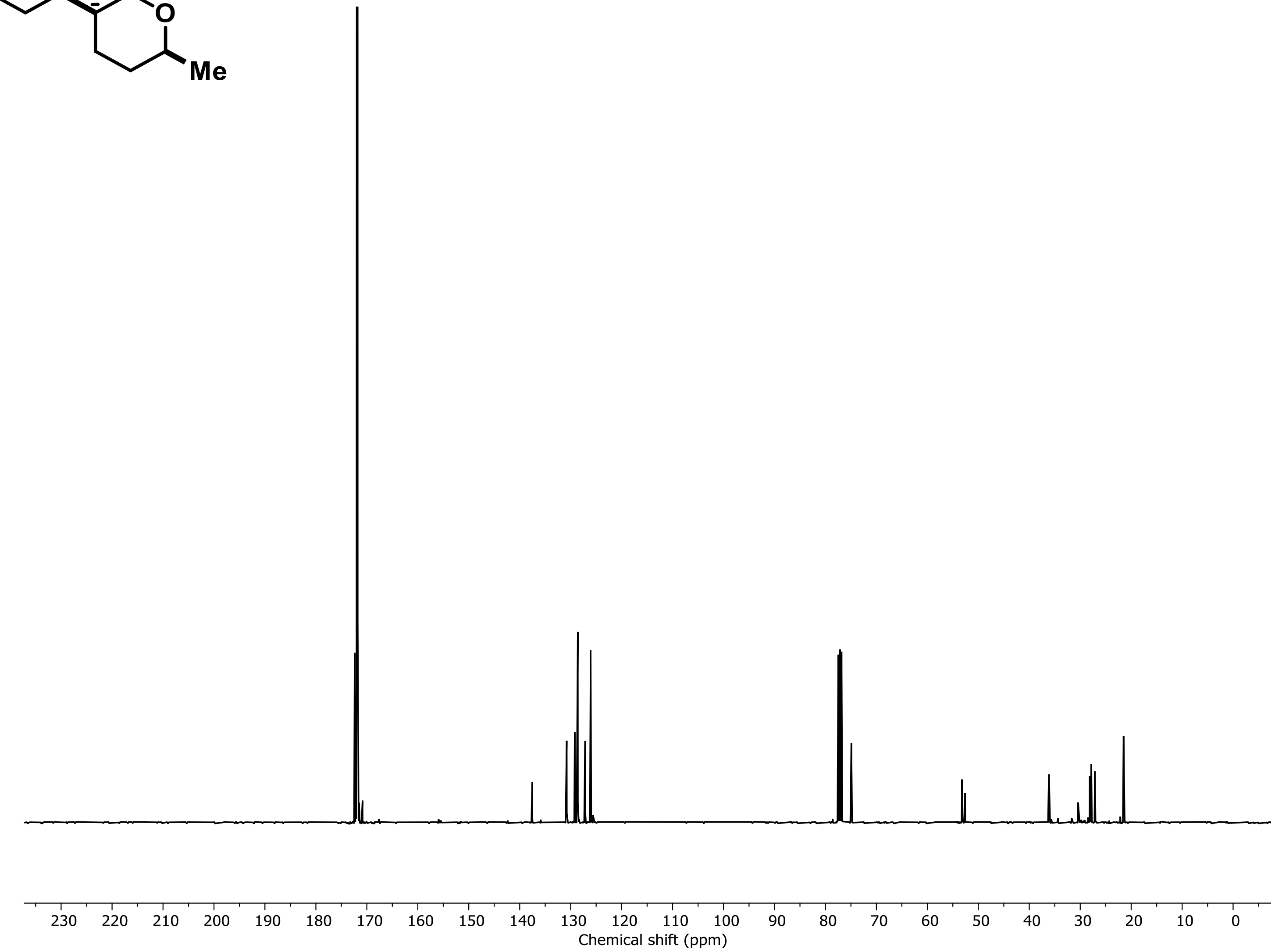

***Rac*–methyl (3*R*,6*R*)–6–methyl–2–oxo–3–((*E*)–4–phenylbut–3–en–1–yl)tetrahydro–2*H*–pyran–3–carboxylate–^{13}*C* (1a'–^{13}*C*)**

^{1}H NMR (400 MHz, $CDCl_3$)

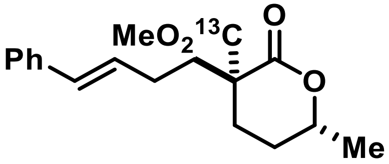

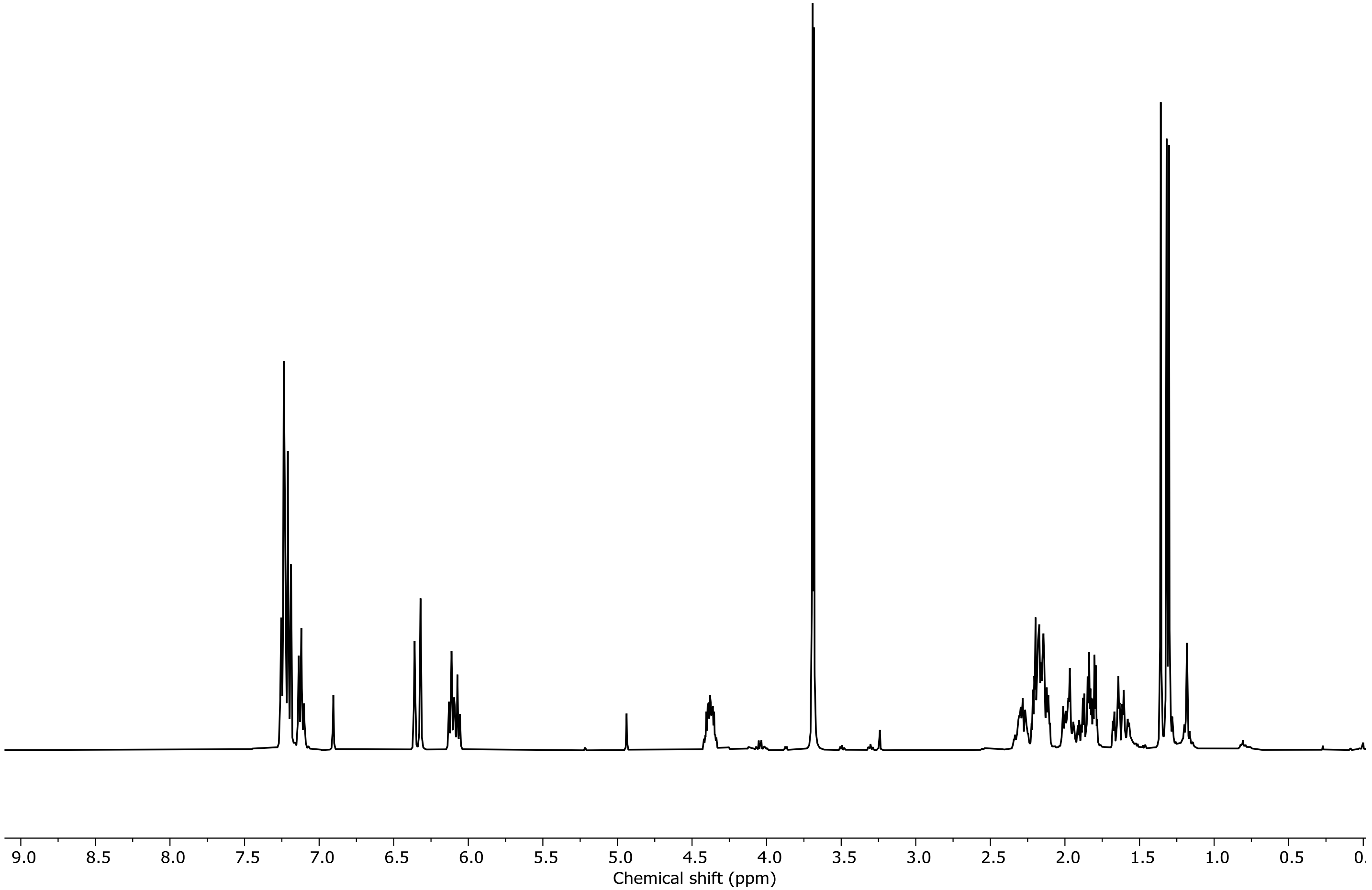

^{13}C NMR (101 MHz, $CDCl_3$)

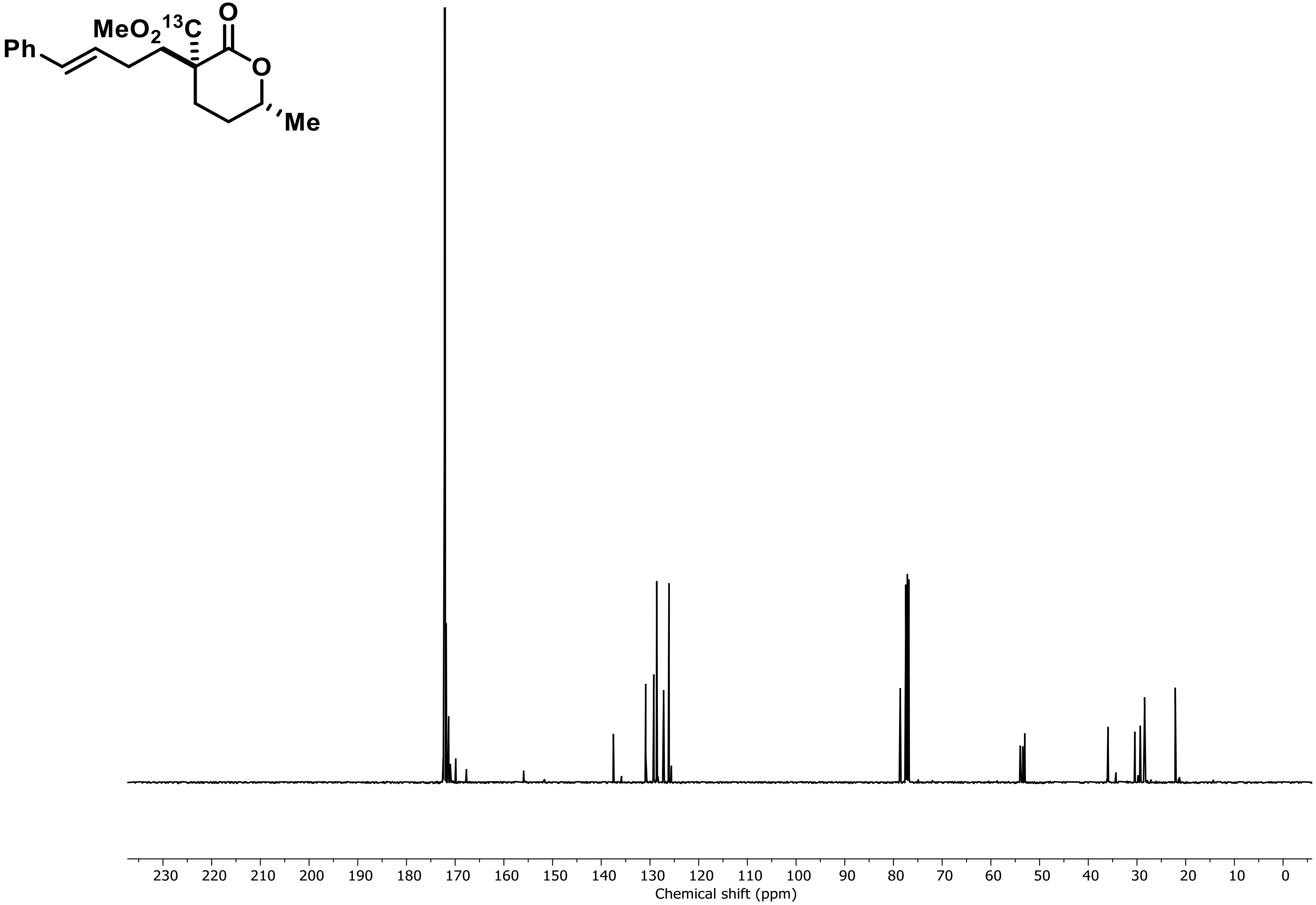

***Rac*-methyl (R)-2-benzyl-4-((3S,6S)-6-methyl-2-oxotetrahydro-2H-pyran-3-yl)butanoate-*^{13}C* (3a–*^{13}C*)**

^{1}H NMR (400 MHz, $CDCl_3$)

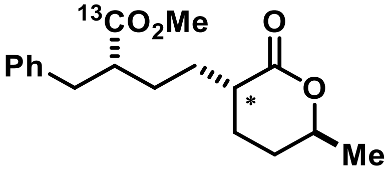

3:1 d.r.

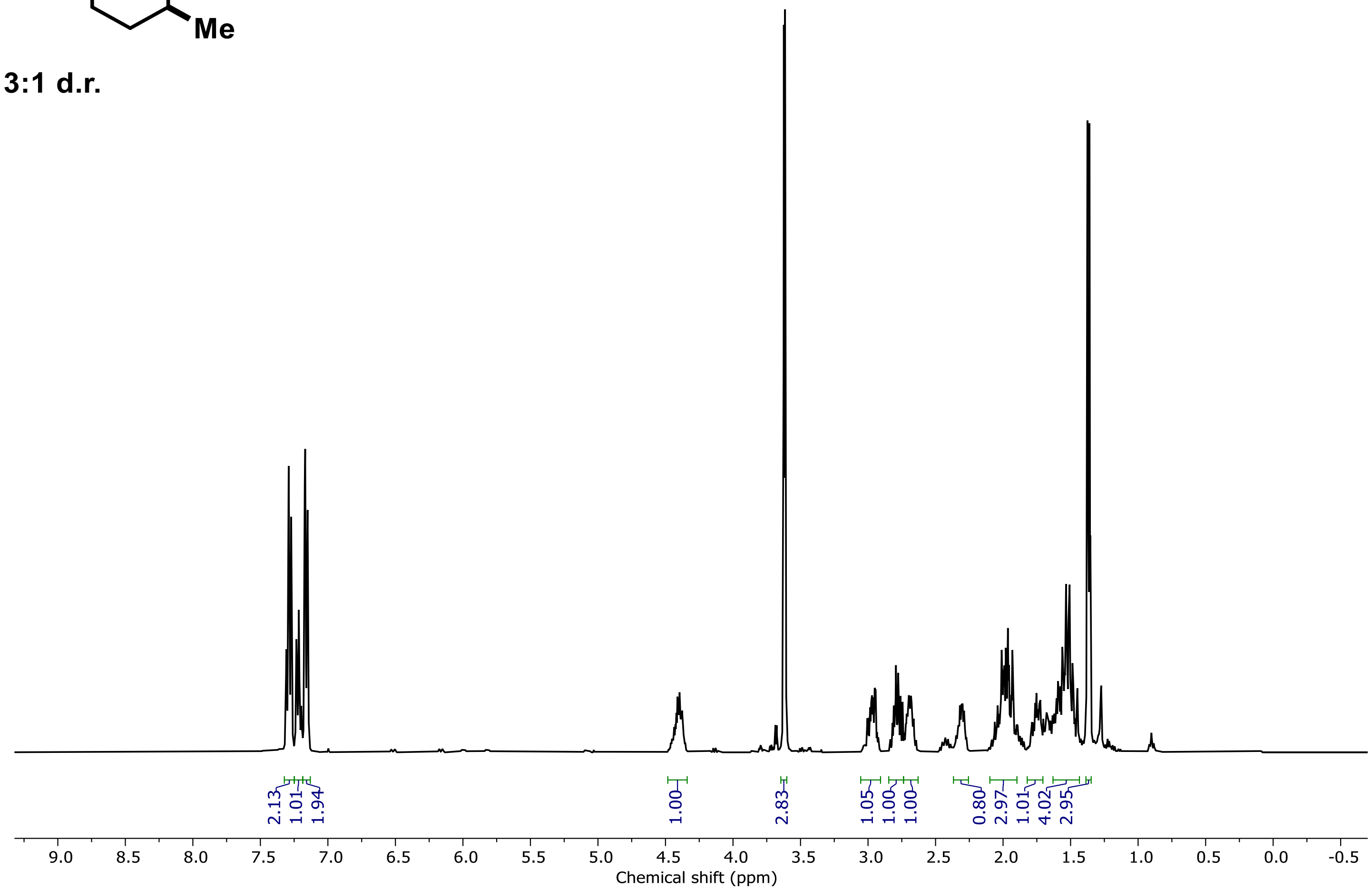

^{13}C NMR (101 MHz, $CDCl_3$)

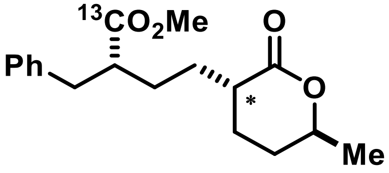

3:1 d.r.

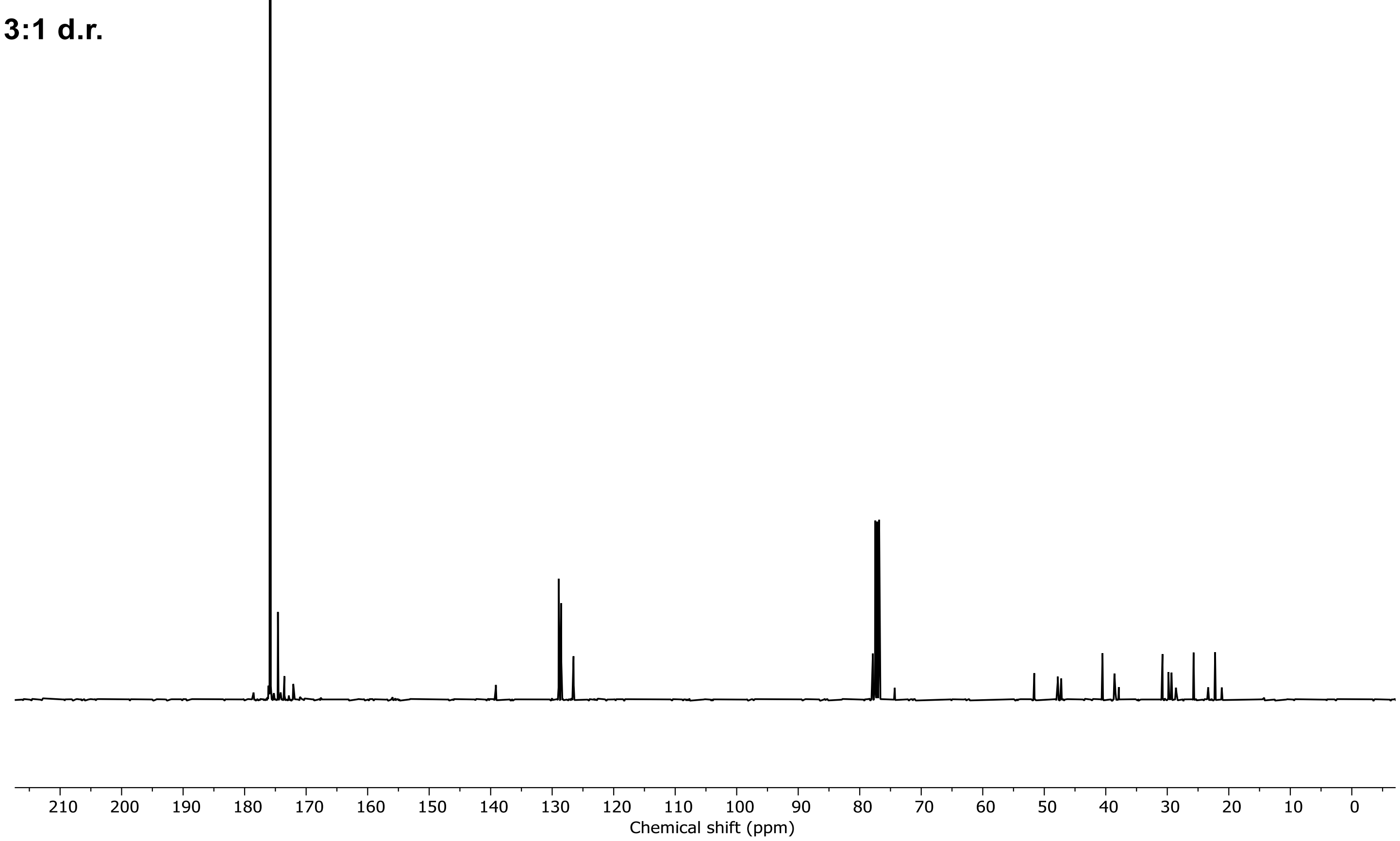

Dimethyl (*E*)–2–methyl–2–(4–phenylbut–3–en–1–yl)malonate (4)

^{1}H NMR (400 MHz, $CDCl_3$)

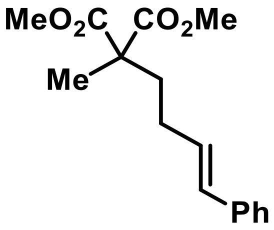

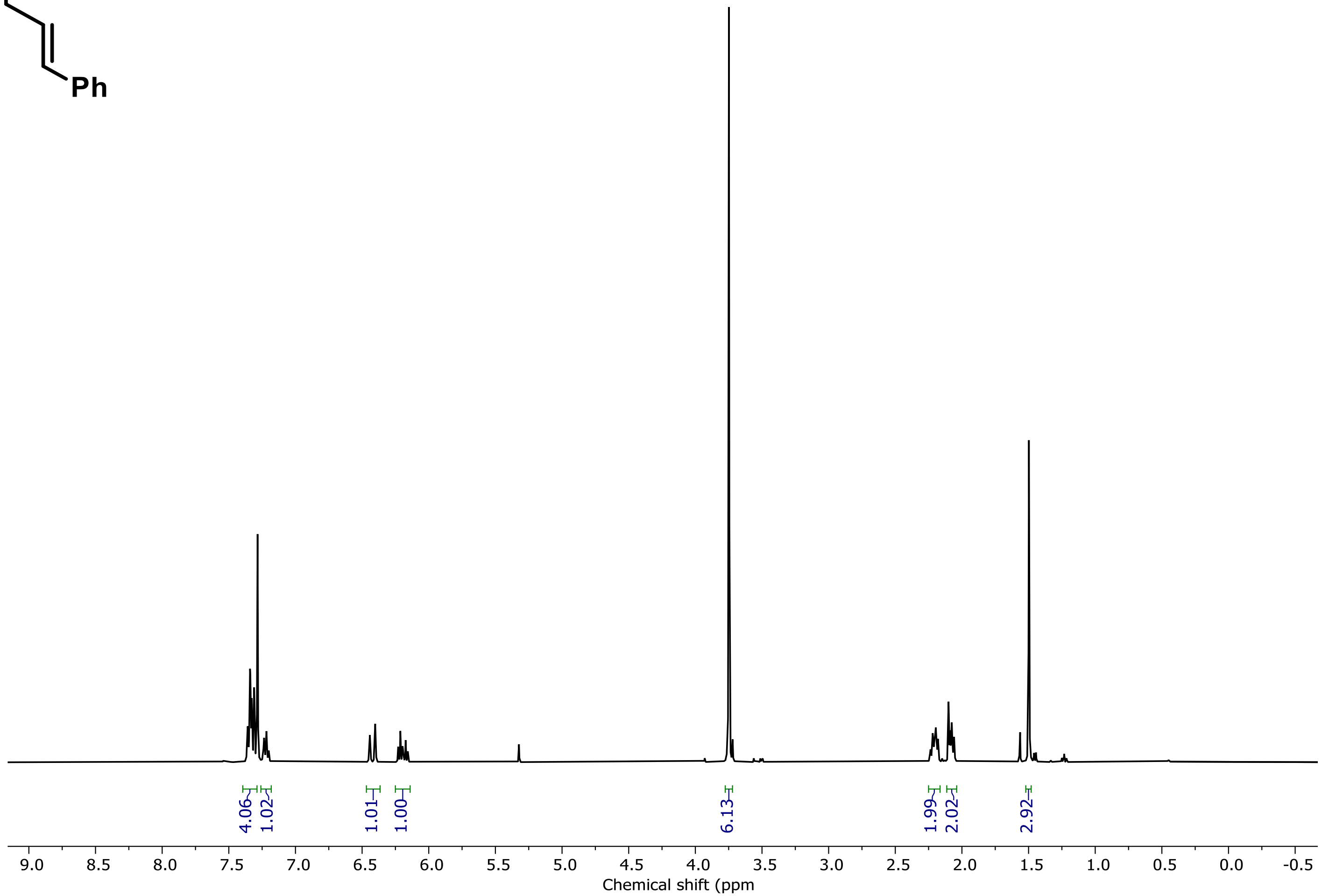

^{13}C NMR (101 MHz, $CDCl_3$)

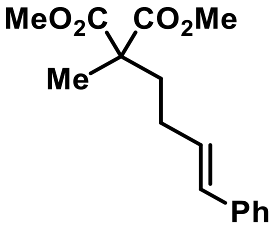

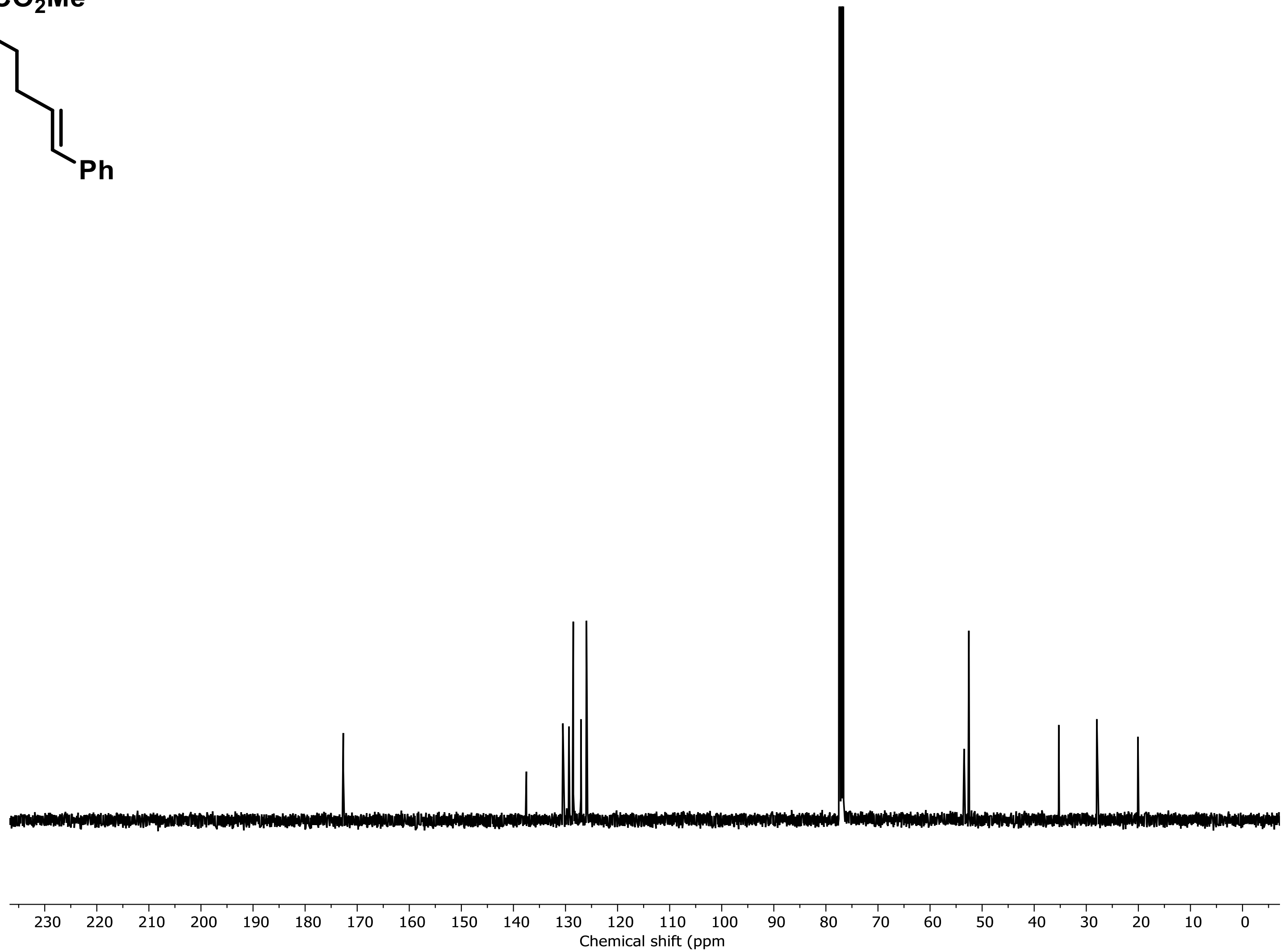

Methyl (E)-2,2-dimethyl-6-phenylhex-5-enoate (S37)

^{1}H NMR (400 MHz, $CDCl_3$)

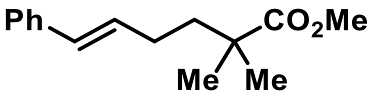

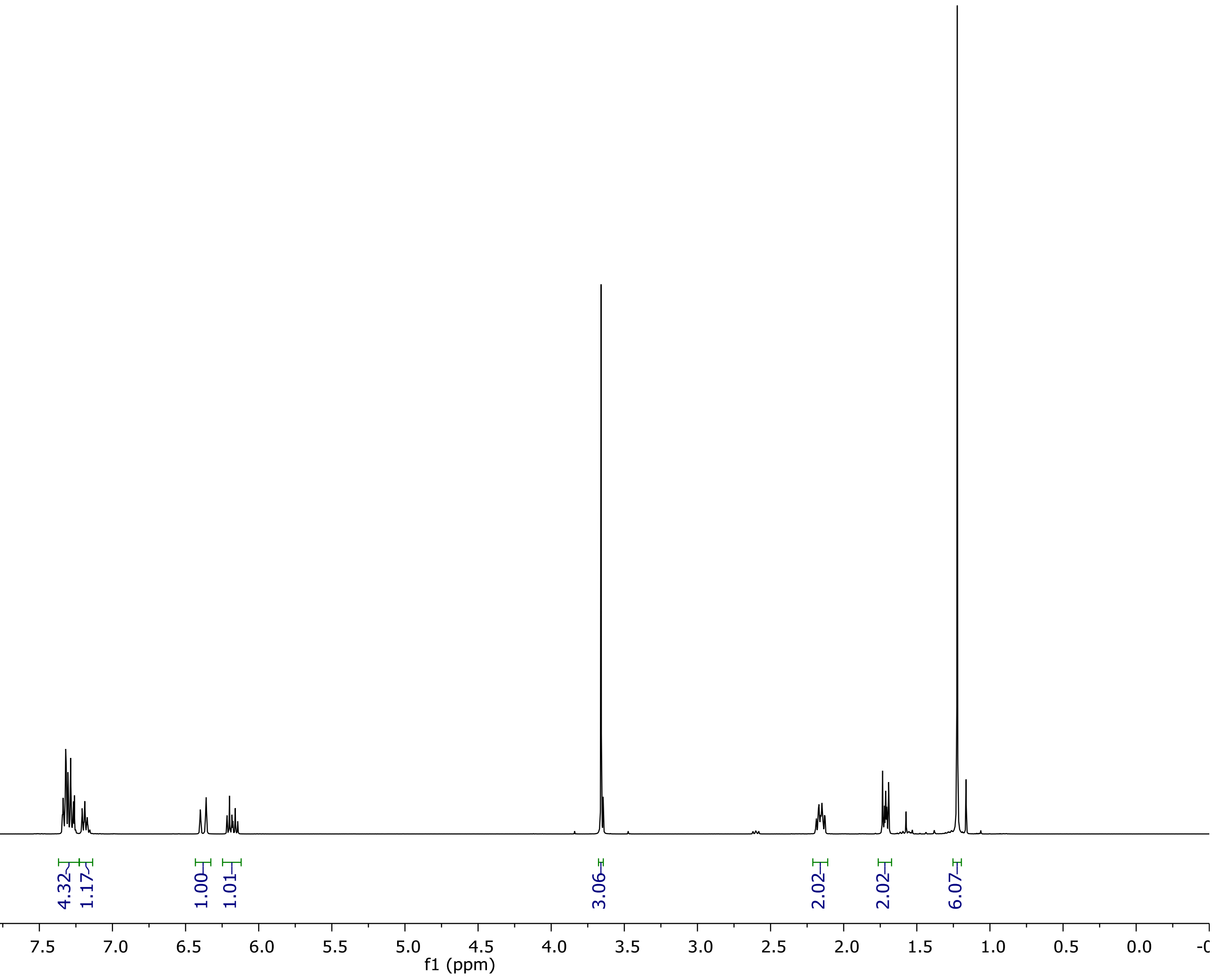

^{13}C NMR (101 MHz, $CDCl_3$)

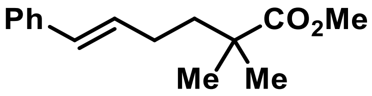

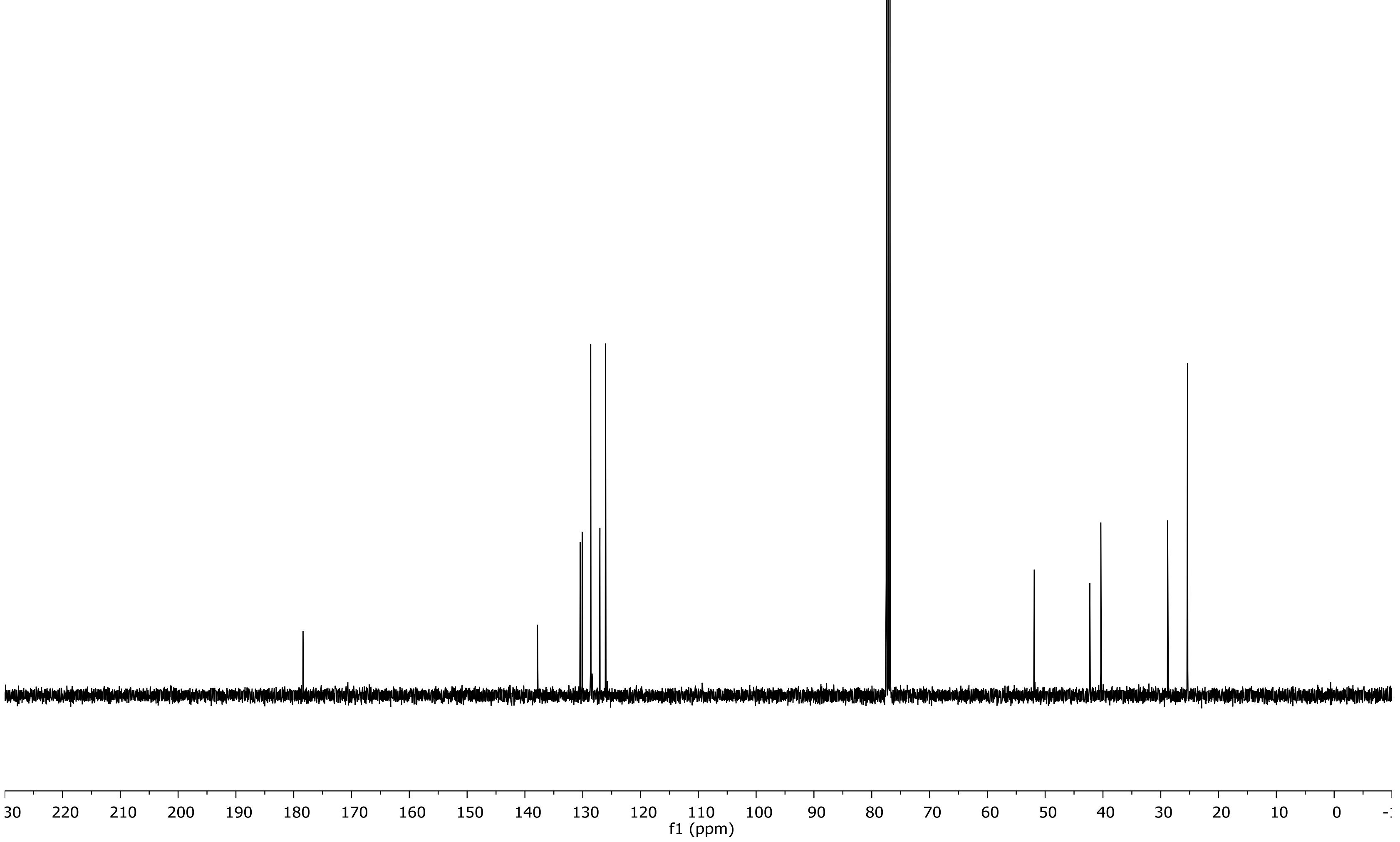

Methyl 3–benzyl–2–hydroxy–1–methylcyclopentane–1–carboxylate (5)

^{1}H NMR (400 MHz, $CDCl_3$)

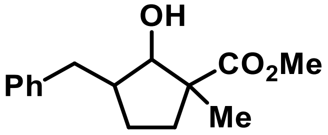

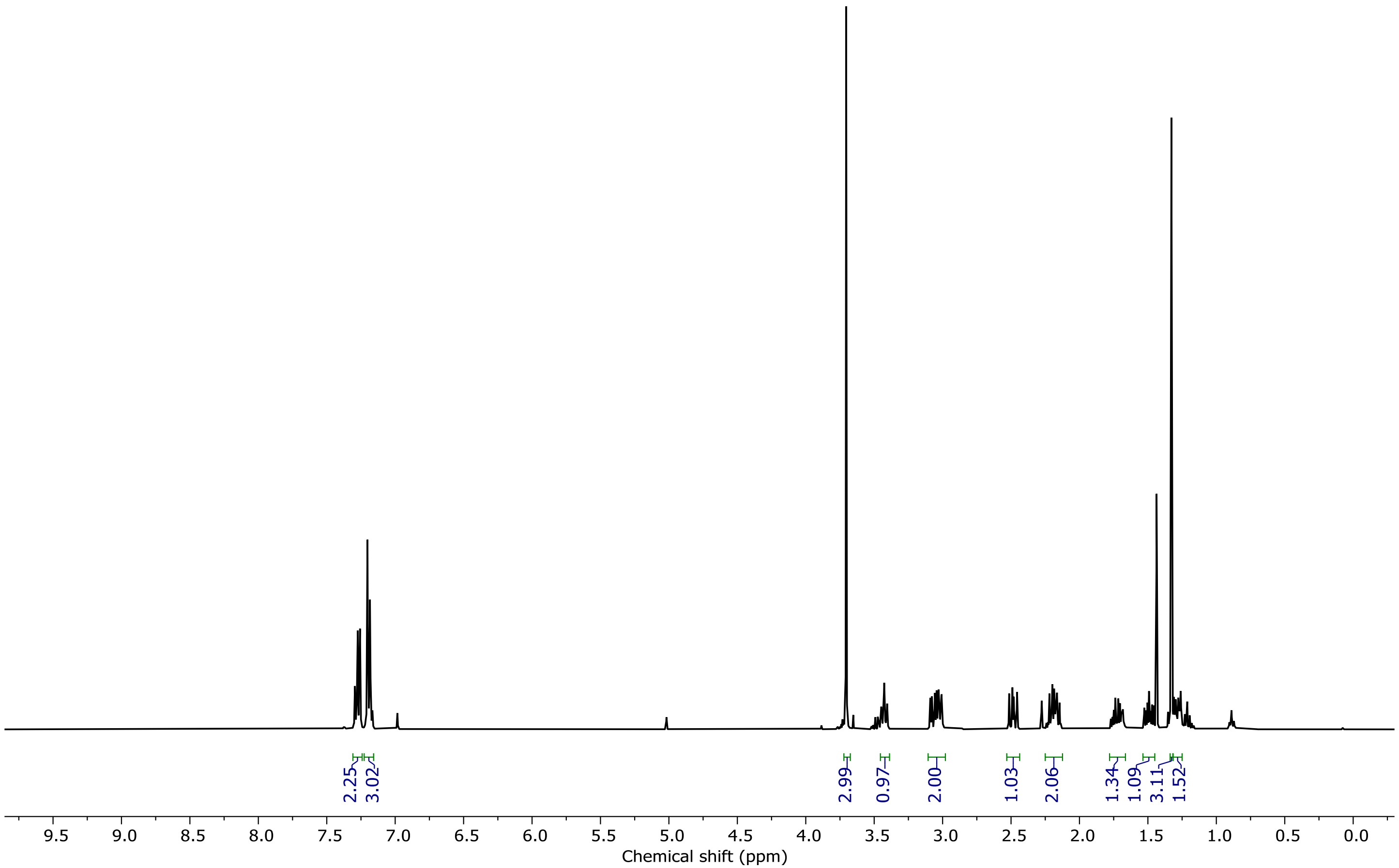

^{13}C NMR (101 MHz, $CDCl_3$)

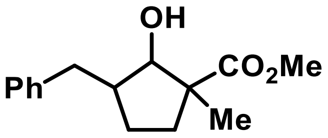

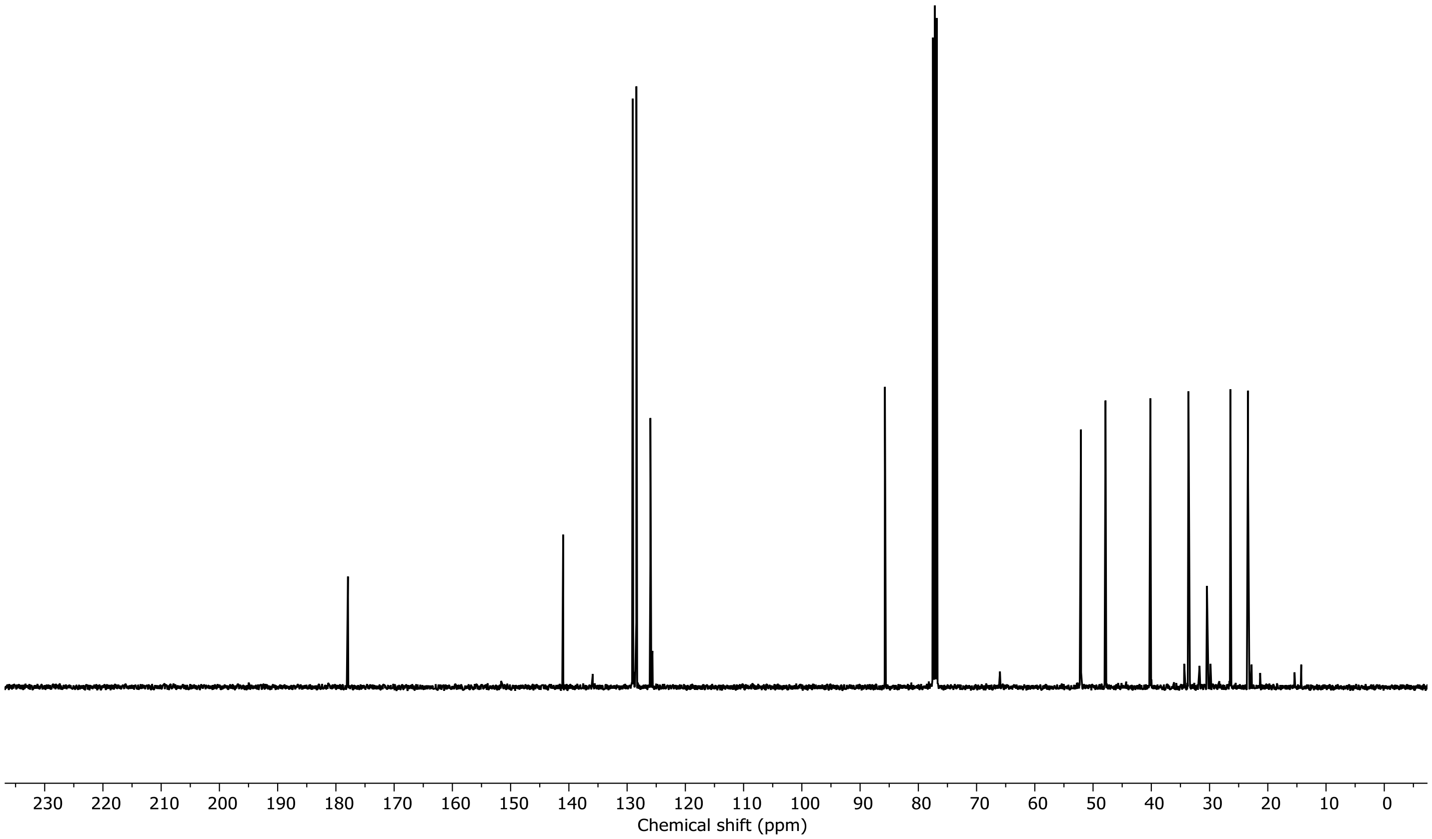

Supplement: Supplementary file 1 — ja2c05972_si_001.pdf [file ja2c05972_si_001.pdf]
